# Transcriptomic analysis of the human placenta reveals trophoblast dysfunction and augmented Wnt signalling associated with spontaneous preterm birth

**DOI:** 10.3389/fcell.2022.987740

**Published:** 2022-10-24

**Authors:** Khondoker M. Akram, Neha S. Kulkarni, Abbey Brook, Matthew D. Wyles, Dilly O. C. Anumba

**Affiliations:** ^1^ Academic Unit of Reproductive and Developmental Medicine, Department of Oncology and Metabolism, The University of Sheffield, Sheffield, United Kingdom; ^2^ Sheffield Institute for Translational Neuroscience (SITraN), University of Sheffield, Sheffield, United Kingdom

**Keywords:** preterm birth, transcriptomic analysis, trophoblast dysfunction, Wnt signalling, placenta, miRNAs

## Abstract

Preterm birth (PTB) is the leading cause of death in under-five children. Worldwide, annually, over 15 million babies are born preterm and 1 million of them die. The triggers and mechanisms of spontaneous PTB remain largely unknown. Most current therapies are ineffective and there is a paucity of reliable predictive biomarkers. Understanding the molecular mechanisms of spontaneous PTB is crucial for developing better diagnostics and therapeutics. To address this need, we conducted RNA-seq transcriptomic analysis, qRT-PCR and ELISA on fresh placental villous tissue from 20 spontaneous preterm and 20 spontaneous term deliveries, to identify genes and signalling pathways involved in the pathogenesis of PTB. Our differential gene expression, gene ontology and pathway analysis revealed several dysregulated genes (including *OCLN*, *OPTN*, *KRT7*, *WNT7A*, *RSPO4*, *BAMBI*, *NFATC4*, *SLC6A13*, *SLC6A17*, *SLC26A8* and *KLF8*) associated with altered trophoblast functions. We identified dysregulated Wnt, oxytocin and cellular senescence signalling pathways in preterm placentas, where augmented Wnt signalling could play a pivotal role in the pathogenesis of PTB due to its diverse biological functions. We also reported two novel targets (*ITPR2* and *MYLK2*) in the oxytocin signalling pathways for further study. Through bioinformatics analysis on DEGs, we identified four key miRNAs, - miR-524-5p, miR-520d-5p, miR-15a-5p and miR-424-5p - which were significantly downregulated in preterm placentas. These miRNAs may have regulatory roles in the aberrant gene expressions that we have observed in preterm placentas. We provide fresh molecular insight into the pathogenesis of spontaneous PTB which may drive further studies to develop new predictive biomarkers and therapeutics.

## Introduction

Preterm birth (PTB), defined as parturition before 37 completed gestational weeks, is the leading cause of death in neonates and children under-five years of age. Of the 15 million babies born preterm worldwide annually, more than one million die, accounting for about 16% of all child and 35% of newborn deaths (Paquette et al., 2018; Brockway et al., 2019; Chawanpaiboon et al., 2019). Although the risk of mortality is significantly higher in early PTBs (<34 weeks), the more frequently occurring late PTBs (>34 but <37 weeks) is also associated with higher risks of adverse outcomes compared to term babies (Teune et al., 2011). About 70% of preterm births occur spontaneously, whilst ∼30% are medically-indicated and physician-initiated aimed to mitigate maternal or fetal pregnancy complications, such as preeclampsia or fetal growth restriction (Goldenberg et al., 2008; Myatt et al., 2012; Eidem et al., 2015). Spontaneous PTB may occur with or without preceding preterm spontaneous rupture of membranes (PPROM) (Moutquin, 2003; Goldenberg et al., 2008; Vogel et al., 2018).

Spontaneous PTB has been proposed as a syndrome associated with multifactorial mechanistic risk factors, such as infection, decidual senescence, breach of maternal-fetal tolerance, cervical insufficiency, maternal stress, uterine vascular disorders, uterine overdistension, declined progesterone action or other unknown aetiologies (Romero et al., 2014). These risk factors are associated with multiple genetic and environmental predispositions. However, understanding the precise underlying molecular mechanism of spontaneous preterm labour remains elusive (Romero et al., 2014; Monangi et al., 2015).

Cumulative evidence suggests that parturition is driven by an orchestrated acute inflammatory response. Microarray analysis reveals that normal human parturition at term is associated with expression of multiple acute inflammatory genes within the chorioamniotic membrane, without any histological evidence of inflammation in the placenta or maternal systemic tissues. This inflammatory gene expression pattern has no temporal association with either the interval after rupture of membranes or the duration of labour (Haddad et al., 2006). Numerous targeted analyses have also identified overexpression of acute inflammatory genes and proteins including IL1β, IL6, IL8, CCL3, CCL4 and TLR-2 in the chorioamniotic membrane, amniotic fluid, myometrium and cervix during term labour (Elliott et al., 2001; Chan et al., 2002; Marvin et al., 2002; Maul et al., 2002; Kim et al., 2004). Although it is widely believed that term and preterm labours follow a common signalling pathway, there is emerging evidence that the initiation of preterm labour could be driven by underlying pathological, as yet unexplored, signalling (Haddad et al., 2006; Romero et al., 2014).

Unbiased global gene expression analysis of term and preterm placenta using Next Generation Sequencing (NGS) techniques may reveal differential gene expression and provide underlying mechanistic insight into preterm labour. The villous compartment at the maternal-fetal interface of the placenta is of interest in elucidating such differential gene expression. It consists of a diverse group of cells that play crucial roles in the maintenance of pregnancy across all trimesters (Pereira, 2018; Turco & Moffett, 2019). Histopathological studies on human preterm placentas have shown the morphological heterogeneity of trophoblasts as well as advanced villous maturation, features which may play roles in spontaneous PTB (Morgan et al., 2013; Khong et al., 2016; Morgan, 2016; Nijman et al., 2016). However, there is a limitation of studies profiling the transcriptome (the mRNA expressed from the genes) of the villous part of the placenta in spontaneous PTB. Over the last decade the majority (76%) of placental transcriptomic studies employed microarray techniques to explore conditions such as preeclampsia and chorioamnionitis which account for many medically-indicated PTB (Eidem et al., 2015; Paquette et al., 2018).

A recent NGS-based study on 11 term (6 placentas plus 5 published GEO data), 12 idiopathic spontaneous preterm (8 placentas plus 4 GEO data) and 9 infected preterm villous tissue samples (8 placenta plus 1 GEO data) identified 188 differentially expressed genes (DEGs) in spontaneous PTB compared to term, and 347 DEGs in idiopathic spontaneous PTB compared to infected PTB. In this study, spontaneous PTB showed involvement of aberrant IGF (Insulin-like growth factor) signalling, inflammation and placental maturity compared to infected PTB (Brockway et al., 2019). This study primarily focuses on elucidating the molecular difference between idiopathic spontaneous PTB without clinical infection and PTB with infection.

We hypothesised that trophoblast dysfunction plays key role in the pathogenesis of idiopathic spontaneous PTB. To test this hypothesis, we have conducted an NGS-based transcriptomic analysis (RNA-seq) on 20 idiopathic spontaneous preterm and 20 spontaneous term fresh placental villous tissue samples using the MinION Oxford Nanopore technique and identified 337 differentially upregulated and 564 downregulated genes in the preterm placenta. Several of these genes have been described to be associated with dysregulated trophoblast functions, including syncytiotrophoblast differentiation, cytotrophoblasts proliferation, migration, and dysregulated solute transport system. We also identified several dysregulated key signalling pathways including Wnt, oxytocin and cellular senescence in preterm placentas which may be involved in the pathogenesis of PTB. In addition, we employed qRT-PCR and bioinformatics analysis to identify a panel of C19MC cluster microRNAs (miRNAs) which may have regulatory roles on the key genes involved in preterm parturition. Taken together, our data provides fresh insight into the pathogenesis of spontaneous PTB, and identifies target molecules and pathways that can inform new PTB therapeutics and biomarkers.

## Materials and methods

### Participant recruitment

A total of 40 pregnant women (aged >16 years) who delivered at the Jessop Wing Maternity Hospital, Sheffield, United Kingdom during the period of April 2019 to December 2020 were recruited for the study (Table 1). Amongst them, 20 women delivered at term (>37 weeks of gestation) whilst 20 women delivered preterm (<37 weeks of gestation). Only women carrying singleton pregnancies and who delivered spontaneously were included. Women with multiple pregnancies or genital tract infections were excluded from the study.

**TABLE 1:** Clinical characteristics of the participants. Data is presented as medians with percentages or ranges in the parentheses. *p* values were calculated by either Mann-Whitney U test or ^#^Fisher’s exact test.

Participant criteria			
	Term	Preterm	*p* values
	*n* = 20	*n* = 20	
Maternal age, yrs (range)	31.1 (22.2–40.1)	27.5 (17.8–40.7)	0.0924
Maternal Race, n, (%)			
White	17 (85%)	18 (90%)	
Black	2 (10%)	0 (0%)	
Asian and others	1 (5%)	2 (10%)	
Gestational age, wks (range)	39.6 (37.4–41.1)	33.5 (24–36.6)	<0.0001
Subtypes of preterm births (%)			
Extremely preterm (<28 weeks)	–	3 (15%)	
Very preterm (28–31 weeks)	–	4 (20%)	
Moderate preterm (32–33 weeks)	–	4 (20%)	
Late preterm (34—< 37 weeks)	–	9 (45%)	
Spontaneous labour	100%	100%	
Membrane rupture (%)			
SROM	12 (60%)	1 (5%)	<0.0001#
PPROM	0 (0%)	10 (50%)	
AROM	7 (35%)	8 (40%)	
Others	1 (5%)	1 (5%)	
Mode of delivery (%)			
Vaginal (VD)	17 (85%)	15 (75%)	
Caesarean section (CS)	1 (5%)	3 (15%)	
Instrumental	2 (10%)	2 (10%)	
BMI at delivery, kg/m2 (Range)	27.1 (21.2–44.3)	27.5 (17.7–53)	0.6902
Smoking (%)			
Yes	3 (15%)	4 (20%)	
No	17 (85)	16 (80%)	
Newborn birth weight, kg	3.51	1.86	<0.0001
Newborn sex (%)			
Male	11 (55%)	11 (55%)	
Female	9 (45%)	9 (45%)	
Placenta gross weight, gm	673.9	409	<0.0001

Note: SROM, spontaneous rupture of membranes; PPROM, preterm premature rupture of the membranes; AROM, artificial rupture of membranes.

### Tissue harvesting

Written consents were taken before labour. The placentas were collected, and the tissue samples processed within 3 h of delivery. Clinical information was recorded on the case report form and electronically employing the REDCap data management system (REDCap Consortium, Vanderbilt). The gross weight of the whole placenta, along with the umbilical cord and amniotic membrane were recorded and tissue samples were harvested in a biosafety level-2 laminar flow hood under sterile environment. A 2 cm by 2 cm tissue flap of decidua basalis was removed from the pericentral lobe of the maternal surface of the placenta. Approximately 1 gm of villous tissue (devoid of decidua), was excised and was washed twice in sterile PBS (ThermoFisher, Cat. No- 10010056) to get rid of excess maternal blood. Tissue sample was then taken into a 2 ml cryovial (Corning, Cat. No-CLS430659) in 1.5 ml of RNA Later solution (Qiagen, Cat. No- 76106) and left to stand for 30 min at room temperature before storing it in the −80°C freezer for later use.

### RNA extraction for RNA-seq and qRT-PCR

Total RNA was extracted from the villous tissue (VT) samples using RNeasy Plus Mini Kit (QIAgen, Cat No- 74134), following the manufacturer’s instruction. Briefly, frozen tissues were thawed at room temperature and approximately 35 mg of tissue was taken from each sample in RLT Lysis buffer in a Sample Tube (Qiagen, Cat No- 990381) along with a 5 mm diameter stainless steel bead (Qiagen, Cat No- 69989). Tissue was completely homogenised by a TissueLyser LT homogeniser (Qiagen, Cat No- 85600) for 10 min at 50 Hz speed with 30 s rest in every 2 min. Tissue homogenate was centrifuged for 3 min at 18,000 x g and the supernatant was filtered through a gDNA eliminator spin column to remove genomic DNA. The flowthrough was used for RNA extraction using a mini spin column as per manufacturer’s protocol. Purified total RNA was resuspended in 50 µl of RNase-free water. The quality and quantity of the yield RNA was checked using Nanodrop ND1000, Qubit RNA HS Assay Kit (ThermoFisher, Cat No- Q32852) and Agilent 6000 Nano kit (Agilent, Cat No- 5067-1511) with 2100 Expert software. RNAs with an RIN value 7–9 and a 260/280 ratio >2.0 were used for downstream Next Generation RNA sequencing using MinION Oxford Nanopore Technology (Supplementary Figure S1). All RNA samples were stored in −80°C.

### Nanopore library preparation

Sequencing libraries were prepared using the Oxford Nanopore Technologies (ONT) PCR-cDNA Barcoding Kit (Oxford Nanopore, Cat No- SQK-PCB109). Briefly, 50 ng of total RNA from each sample was combined with 1 µl of VN primers (2 µM), 1 µl of dNTPs (10 mM) and water to a final volume of 9 µl. The mixture was incubated at 65°C for 5 min, then snap cooled on ice. A master mix was prepared containing 4 µl 5x RT Buffer (ThermoFisher, Cat No- EP0751), 1 µl RNaseOUT (Life Technologies, Cat No- 10777019), 1 µl nuclease free water, 2 µl Strand-Switching Primer (ONT, 10 µM). 8 µl of the master mix was combined with the RNA and incubated for 2 min at 42°C. 1 µl of Maxima H Minus Reverse Transcriptase (ThermoFisher, Cat No- EP0751) was added and incubated at 42°C for 90 mins, followed by heat inactivation at 85°C for 5 min. Two separate aliquots of 5 µl of the reverse transcribed RNA samples were PCR amplified and simultaneously barcoded with Barcode Primers (ONT, BP01-BP12, one barcode assigned to each sample/duplicate) using 2x LongAmp Taq Master Mix (NEB, Cat No- M0287).Following PCR, 1 µl of Exonuclease I (NEB, Cat No- M0293) was added to each sample followed by incubation at 37°C for 15 min and 80°C for 15 min. PCR reactions were then pooled for SPRI bead clean up using Agencourt AMPure XP beads (Beckman, Cat No- A63880). Samples with unique barcodes were pooled and combined with 1 µl of RAP (ONT) to add the sequencing adaptor and incubated for 5 min at room temperature. The libraries were then sequenced on an R9.4.1 flow-cell following standard ONT protocols.

### qRT-PCR

100 ng of total RNA from each sample was utilised for the one-step qRT-PCR using QuantiNova SYBR Green RT-PCR Kit (Qiagen, Cat. No- 208154) and QuantiTect Primer Assay primer probes (Qiagen, Cat. No- 249900) (Supplementary Table S1) following the manufacturer’s instruction. The qRT-PCR reactions were done on Rotor-Gene Q (Qiagen,Cat No- 9001550) following the thermocycling protocol as follows: reverse transcription at 50°C for 10 min followed by inactivation at 95°C for 2 min, then 40 cycles of PCR with denaturation at 95°C for 5 s and combined annealing/extension at 60°C for 10 s for each cycle. Melting curve analysis was performed for each qRT-PCR run. *GAPDH* was used as a housekeeping gene for normalisation of data which was expressed equally across all the 40 samples, and there was no significant difference in Cq values of *GAPDH* between the samples (mean Cq = 14.61 ± 0.45, *p* = 0.1274) (Supplementary Figure S2). The Cq values were used for calculation of relative gene expression (2^−ΔCq^, relative to *GAPDH*) in term and preterm groups (Livak & Schmittgen, 2001; Schmittgen & Livak, 2008). Data were presented as Log_10_ relative gene expression.

### Reverse transcription and qPCR for miRNA

Total RNA was extracted from frozen placenta villous tissue using miRNeasy Mini Kit (Qiagen, Cat No- 217004) following the manufacturer’s instruction as described above. 10 ng of RNA was reverse-transcribed into cDNA at 42°C for 1 h for each sample using the miRCURY LNA RT Kit (Qiagen, Cat No- 339340) following the manufacturer’s instruction. For qPCR, the template cDNA from each sample was diluted at 1 in 60 in nuclease-free water and 6 µl of this diluted cDNA was used for qPCR reaction using miRCURY LNA SYBR Green PCR kit (Qiagen, Cat No- 339346) and miRCURY LNA miRNA PCR Assay primers (Supplementary Table S2) following the manufacturer’s instruction. Each qPCR reaction volume was 20 µl and the thermocycler (Rotor Gene Q) protocol consists of an initial heat inactivation at 95°C for 2 min followed by 40 cycles of denaturation at 95°C for 10 s and a combined annealing/extension at 56°C for 1 min, and a final melting curve analysis (64°C–95°C). U6 snRNA primer was used as internal control and normalisation which was expressed equally across the samples (Supplementary Figure S3). The Cq values were used to calculate the relative miRNA expression (2^−ΔCq^, relative to U6 snRNA) and were presented as Log_10_ expression in each group.

### ELISA assay

A 1 cm^3^ villous tissue block was collected from each fresh placenta as above and snap-frozen in liquid nitrogen in a cryovial for 30-40 min before storing at −80°C for later use. For total protein extraction, 100 mg of tissue from the tissue block was taken in 1 ml of SigmaFAST/IgePal lysis buffer made up of 0.1% IgePal CA-630 (Sigma-Aldrich, Cat No- I3021) with SigmaFAST Protease Inhibitor Cocktail (Sigma-Aldrich, Cat No- S8830) (1 tablet in 100 ml PBS) in PBS in a Sample Tube along with a metal bead as above. Tissue was homogenised by TissueLyser LT for 10 min at 50 Hz at room temperature. Tissue lysate was then incubated at 4°C for 30 min on a roller followed by further homogenisation for 5 min by TissueLyser. Completely lysed tissue homogenate was then centrifuged at 16,000 x g for 20 min at 4°C. Supernatant was aliquoted and total protein concentration was determined by Qubit Protein Assay Kit (Thermo Fisher Scientific, Cat No- Q33211) following the manufacturer’s instruction, and stored at -80°C. Total protein at a concentration of 300 μg/ml was used for quantification of WNT7A protein in each sample by using Human WNT7A ELISA Kit following the manufacturer’s instruction (Fine Test, Cat No- EH2312).

### Bioinformatics analysis

Differential gene expression analysis:An in-house pipeline was generated for raw FASTQ files processing and analysing RNA-seq data to identify differentially expressed genes (DEGs). Sequencing data were demultiplexed by guppy v0.3.0, base calling software (Oxford Nanopore Technologies) and reads quality assessment was done by pycoQC v2.5.2 tool (Leonardi, 2019). The average PHRED quality of reads were between 10 and 11 which considered as good quality read in Nanopore sequencing technique. Reads with PHRED quality score below 8 was filtered out and not considered for subsequent data analysis. The quality-passed reads were mapped to the reference human genome (hg38) using minimap2- 2.17-r941 (Li, 2018). A range of 70%–95% reads were mapped onto the reference genome hg38. Post mapping, statistical analysis was carried out using the R packages (version 3.6.0) and Galaxy platform (Afgan et al., 2018) such as limma and edgeR to identify DEGs between preterm and term samples in 3 batches. To identify DEGs, a false discovery rate (FDR) (Adjusted *p* value) threshold of <0.05 and Log2 fold change (Log_2_FC) cut off ±1 was used.

Gene ontology (GO) and pathway analysis:To explore the underlying biological processes and signalling pathways, gene ontology (GO) and KEGG (Kyoto Encyclopedia of Genes and Genomes) signalling pathway analysis with upregulated and downregulated DEGs was conducted separately using g:Profiler tool (Reimand et al., 2019). GO terms and pathway enrichments were considered significant when an adjusted *p* value (P_adj_) was less than 0.05. A default g:SCS algorithm was used to compute multiple testing correction for *p*-values (Reimand et al., 2007).

Gene targets (hub-miRNAs) identification: The DEGs were further tested to obtain their miRNA targets using various bioinformatic tools. The obtained 3′UTR exons of the DEGs identified were considered. The appropriate 3′UTR regions for the DEGs were obtained from the UCSC Genome Browser (http://genome.ucsc.edu/index.html). An in-house custom Rscript was specifically developed to map the UTR exons of all the human genes to the DEGs. The custom script produced bed files which were then converted to fasta files using Bedtool ver2.29.2 (Quinlan & Hall, 2010). The mature.fasta file of only human miRNA sequences was obtained from miRbase database (Kozomara et al., 2019). Further, miRanda tool ver v3.3a (Enright et al., 2003) was used to obtain miRNA-mRNA target (DEGs), miRNA-mRNA binding score and binding energy. The obtained miRNA-mRNA targets were validated using the starBase database (Yang et al., 2011). We specifically wanted to identify hub-miRNA which are miRNAs that interact with more than one gene target, as they may have a more important role in gene regulation. The hub of miRNA with ≥3 gene targets was considered for further analysis.

### Statistical analysis

The statistical analysis on the sequencing data was conducted using the R statistical tool (version 3.6.0), R packages and Galaxy platform as specified in the aforementioned sections. Limma and edgeR were used to identify differentially expressed genes (DEGs) between preterm and term samples. An adjusted *p* value (P_adj_) threshold of <0.05 and Log2 fold change cut off ±1 was used for identification of significant DEGs. qRT-PCR and ELISA data were analysed using GraphPad PRISM (version 9.1.1). The Mann-Whitney U test was conducted between groups and a *p* value <0.05 was considered as statistically significant. Data are presented as median and interquartile ranges (IQR), with minima and maxima or mean ± standard deviation (SD).

### Role of funding source

The Funder did not play any role in the study design, data collection, data analyses, interpretation, or writing of the manuscript.

## Results

### Participant criteria

Of the 40 pregnant women recruited for the study as described above, 20 women were term and 20 were preterm with a median gestational age (GA) of 39.6 weeks and 33.5 weeks, respectively (*p* < 0.0001) (Table 1). Table 1 shows the race distribution of the women which was predominantly white (85% of term and 90% of preterm women). None of the subjects had diabetes mellitus or hypertension. Maternal demographics did not significantly differ between the term and preterm groups. Half of the women who experienced spontaneous preterm birth had experienced PPROM as expected (Moutquin, 2003; Goldenberg et al., 2008; Vogel et al., 2018). The median newborn birth weight was almost 50% lighter, and median placenta gross weight was ∼40% lighter, in PTB compared to term birth cases (*p* < 0.0001) (Table 1). The fresh preterm placentas were highly heterogeneous in their gross morphology at the time of tissue harvesting. There was a significant positive correlation between placenta gross weight and newborn birth weight in preterm (r = 0.715, *p* = 0.0004) and term birth (r = 0.574, *p* = 0.015) (Supplementary Figure S4) as previously reported (Soliman et al., 2013). Amongst the preterm group, 45% were late preterm and 35% were extremely preterm or very preterm births. The proportion of male and female babies was same in both groups which eliminated the downstream newborn gender bias in placental transcriptome analysis (Lien et al., 2021).

### Detection of differentially expressed genes in the preterm placenta

**FIGURE 1:**
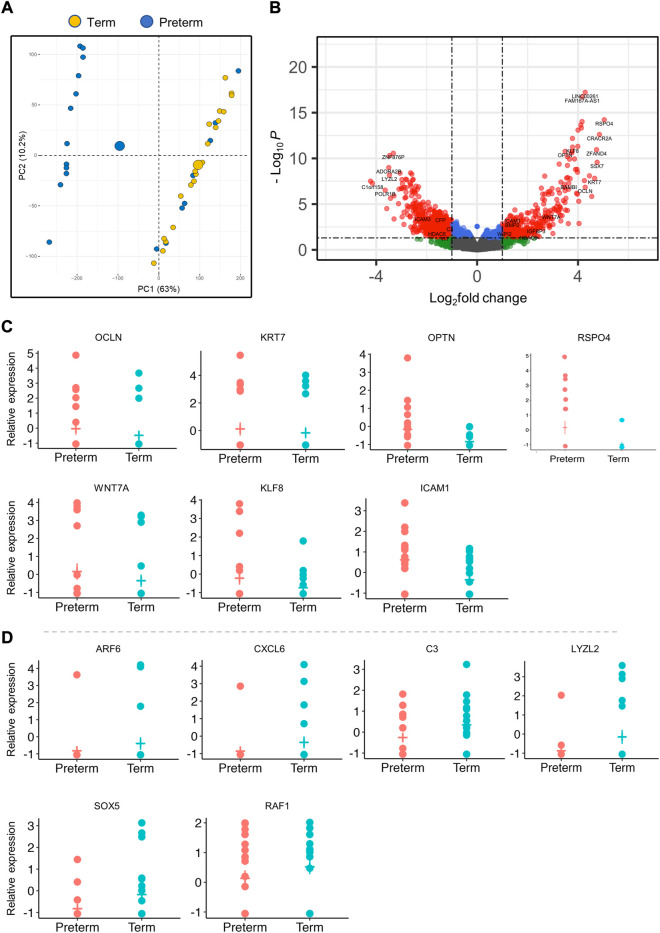
Differentially expressed genes (DEGs) in the placenta. **(A)**. PCA plot shows separation of preterm and term gene clusters (*n* = 40). **(B)**. The volcano plot shows the significant fold change differences between preterm and term placenta samples (*n* = 40, 20 term and 20 preterm). Each dot within the graph indicates an individual gene, with 564 downregulated genes on the left and 337 upregulated genes on the right. Significant DEGs are shown within the threshold lines on the graph. Genes observed Benjamini-Hochberg false-discovery rate (BH-FDR) < 0.05 and a fold change cut off ±1. **(C, D)**. A selected panel of significantly upregulated **(C)** and downregulated **(D)** gene expression in RNA-seq data. P_adj_ < 0.05 for all genes.

We employed the Next Generation RNA sequencing (RNA-seq) method to detect mRNA transcripts (genes) in placenta villous tissue samples using the Oxford Nanopore MinION technique. Good quality sequencing reads were obtained by the pycoQC tool (Leonardi, 2019). Using the minimap2 mapping tool, reads were mapped onto the reference human genome hg38; on average, 80% of the total reads were mapped successfully. Principal component analysis (PCA) was performed on all 40 samples, and a high variance was observed for the first two principal components (PC1 63%, PC2 10.2%). The PCA largely segregated the preterm group from the term group; however, there were few late preterm samples which clustered together with the term samples, possibly due to their gestational age proximity to term (Figure 1A).

**FIGURE 2:**
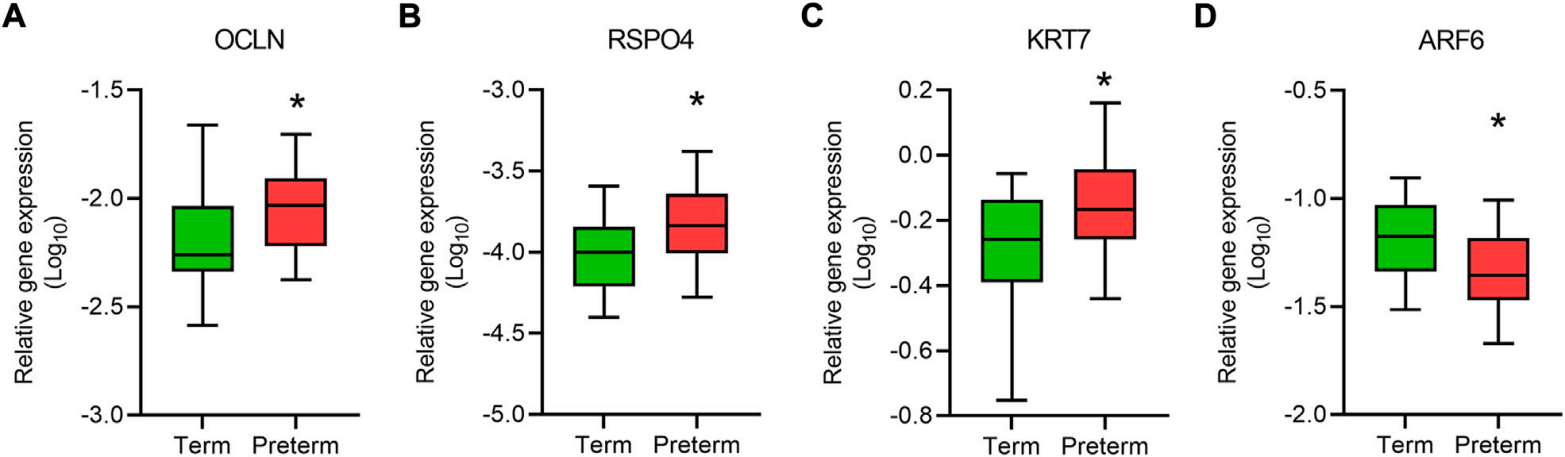
qRT-PCR validation of gene expression. Box plots show relative expression (relative to *GAPDH*) of a panel of genes, *OCLN* **(A)**, *RSPO4* **(B)**, *KRT7* **(C)** and *ARF6* **(D)** in term and preterm placentas. Data are presented as median and interquartile ranges (IQR) with minima and maxima. n = 20 term and 20 preterm, *p < 0.05, Mann-Whitney U test.

We conducted differential gene expression analysis on a total of 53,898 mapped transcripts using limma and edgeR R-packages (McCarthy et al., 2012) and significantly detected 901 DEGs (P_adj_ < 0.05), of which 337 (37.4%) genes were significantly upregulated and 564 (62.6%) genes were downregulated in the preterm placentas compared to term (Figure 1; Table 2, Supplementary Table S3). Amongst the DEGs, 26.1% genes were lacking annotation and 3.8% were lncRNAs (Long non-coding RNAs). The validity of transcriptome detection by RNA-seq was confirmed by detecting a panel of selected DEGs by qRT-PCR on the same cohort of samples which was commensurate with the RNA-seq data (Figure 2).

**TABLE 2:** Top 40 up and downregulated genes in the preterm placenta detected by DEG analysis on 20 term and 20 preterm placenta villous tissue samples with P_adj_ < 0.05.

Upregulated genes			Downregulated genes		
Genes	Log_2_FC	adj. *p* values	Genes	Log_2_FC	adj. *p* values
RSPO4	5.04	1.1E-10	FOXF2-DT	−4.23	3.4E-05
CRACR2A	4.85	1.7E-09	C1orf158	−4.15	5.5E-05
SSX7	4.76	6.9E-07	POLR1B	−3.64	2.2E-04
ZFAND4	4.74	5.0E-08	ADORA2B	−3.50	2.2E-06
KRT7	4.66	2.1E-05	LYZL2	−3.48	1.0E-05
CENPA	4.54	8.3E-04	HLA-DQA2	−3.47	1.5E-07
TECR	4.45	1.2E-05	HAPLN1	−3.43	1.3E-03
OCLN	4.29	1.3E-04	ZNF876P	−3.32	9.4E-08
LINC00261	4.28	3.3E-13	NCK2	−3.30	7.1E-04
PMP22	4.26	3.1E-05	APOB	−3.29	5.0E-03
FAM167A-AS1	4.17	5.1E-13	FANCM	−3.12	4.4E-03
EIF3M	4.16	1.3E-10	FOXA1	−3.10	1.8E-04
SIGIRR	4.13	4.2E-10	LMF1	−3.06	7.0E-06
SLC26A8	4.11	2.4E-10	BEST1	−3.00	1.6E-04
FAM234A	4.03	2.5E-03	DHRS4L1	−2.98	2.6E-05
RHBDL3	4.00	6.2E-10	SIKE1	−2.97	5.0E-03
SLC6A13	3.99	2.7E-08	ZPBP	−2.96	5.8E-05
SIT1	3.98	5.9E-06	OSTCP5	−2.90	5.0E-03
COA5	3.98	5.0E-04	MTND5P27	−2.90	5.0E-03
TMEM171	3.86	3.3E-07	CHRNA7	−2.89	1.3E-04
KLF8	3.78	2.9E-08	LINC01559	−2.83	2.4E-05
LINC00320	3.78	4.0E-09	FRMPD2B	−2.82	1.0E-03
PLEKHM2	3.76	6.7E-08	TPGS2	−2.82	3.4E-04
MIR563	3.76	8.8E-08	PRKX	−2.81	1.3E-04
GUSBP9	3.69	2.2E-05	KCTD9P1	−2.77	5.0E-03
LINC01122	3.66	3.3E-07	NLGN4X	−2.77	5.0E-03
BAMBI	3.63	5.5E-05	TRIM33	−2.71	5.0E-03
GRB7	3.62	5.0E-03	SMS	−2.66	4.9E-05
ABHD12B	3.59	1.8E-05	ARF6	−2.65	5.0E-03
TTLL7	3.58	1.3E-06	PRKX-AS1	−2.65	5.0E-03
SLC25A41	3.58	5.0E-03	GINS1	−2.64	6.7E-06
KCNQ5	3.57	1.9E-07	CSMD2-AS1	−2.63	5.9E-03
ZFP36L2	3.55	2.0E-05	PDIA2	−2.62	1.1E-04
RNU6-1254P	3.50	5.0E-03	UBA6	−2.60	7.1E-04
DPP9-AS1	3.49	2.4E-04	CATSPER2P1	−2.59	8.8E-06
OPTN	3.49	6.7E-08	KLHL31	−2.59	3.0E-04
TNRC18P3	3.47	1.3E-04	ECE1-AS1	−2.58	5.0E-03
RASSF8-AS1	3.46	6.8E-05	CMYA5	−2.56	2.8E-05
MAST4	3.42	2.1E-03	CCDC6	−2.56	1.2E-02
STXBP6	3.37	5.0E-03	USP49	−2.55	2.6E-04

### Gene ontology and signalling pathway analysis reveals diverse trophoblast dysfunctions in the preterm placenta

**FIGURE 3:**
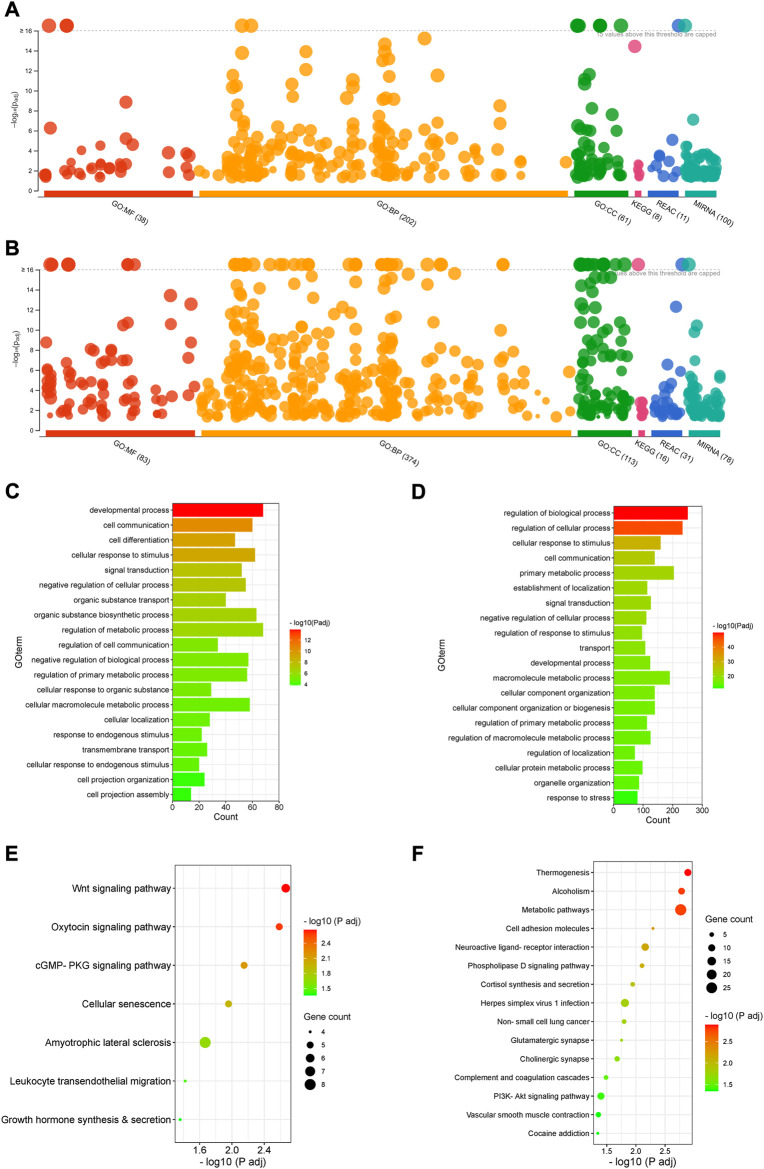
Gene ontology and signalling pathway analysis. **(A,B)**. Manhattan plots showing enrichment of GO terms, KEGG signalling pathways, Reactome and miRNA targets by upregulated **(A)** and downregulated DEGs **(B)** (P_adj_ < 0.05). **(C,D)**. GO enrichment with upregulated **(C)** and downregulated DEGs **(D)** (P_adj_ < 0.05). Selected top 20 GO terms are presented. **(E,F)**. KEGG signalling pathway enrichment with upregulated **(E)** and downregulated DEGs **(F)** (P_adj_ < 0.05).

Next, we conducted gene ontology (GO) and KEGG signalling pathway analysis with upregulated and downregulated DEGs separately using g:Profiler tool (Reimand et al., 2019). For the analysis, DEGs were ranked and the significance of GO and pathway enrichment was considered when an adjusted *p* value (P_adj_) was less than 0.05. A default g:SCS algorithm was used to compute multiple testing correction for *p*-values with a modified Benjamini-Hochberg FDR method (Reimand et al., 2007) (Figure 3).

Both upregulated and downregulated DEGs significantly enriched several GO terms with diverse biological processes predominantly associated with development, cell differentiation, cellular localisation, cell projection assembly, signal transduction, cell communication and metabolism (P_adj_ < 0.05) (Figures 3A–D, Supplementary Table S4).

The upregulated DEGs also significantly enriched several key signalling pathways in the preterm placenta, including Wnt, oxytocin, cellular senescence and cGMP-PKG signalling pathways which are pathologically relevant to PTB (P_adj_ < 0.05) (Figure 3E, Supplementary Table S5). The Wnt signalling was the top-hit signalling pathway in the preterm placenta (P_adj_ = 0.002) which was associated with 6 significantly upregulated genes (*RSPO4*, *WNT7A*, *BAMBI*, *DKK4*, *NFATC4*, *VANGL1*) (Figure 3E, Supplementary Table S5). Amongst them, the *RSPO4* (R-Spondin 4) was the topmost overexpressed gene in the preterm placenta compared to term (Log_2_FC = 5.04, P_adj_ = 1.1 × 10^−10^) (Table 2; Figures 1B,C). Our qRT-PCR analysis also confirmed its upregulation in preterm placentas (*p* < 0.05) (Figure 2B). *RSPO4* gene encodes R-Spondin 4, a member of the R-Spondin family of secreted proteins and acts as a ligand for the Wnt receptor Frizzled and augments Wnt signalling (Kamata et al., 2004; Nam et al., 2006; Carmon et al., 2014).

**FIGURE 4:**
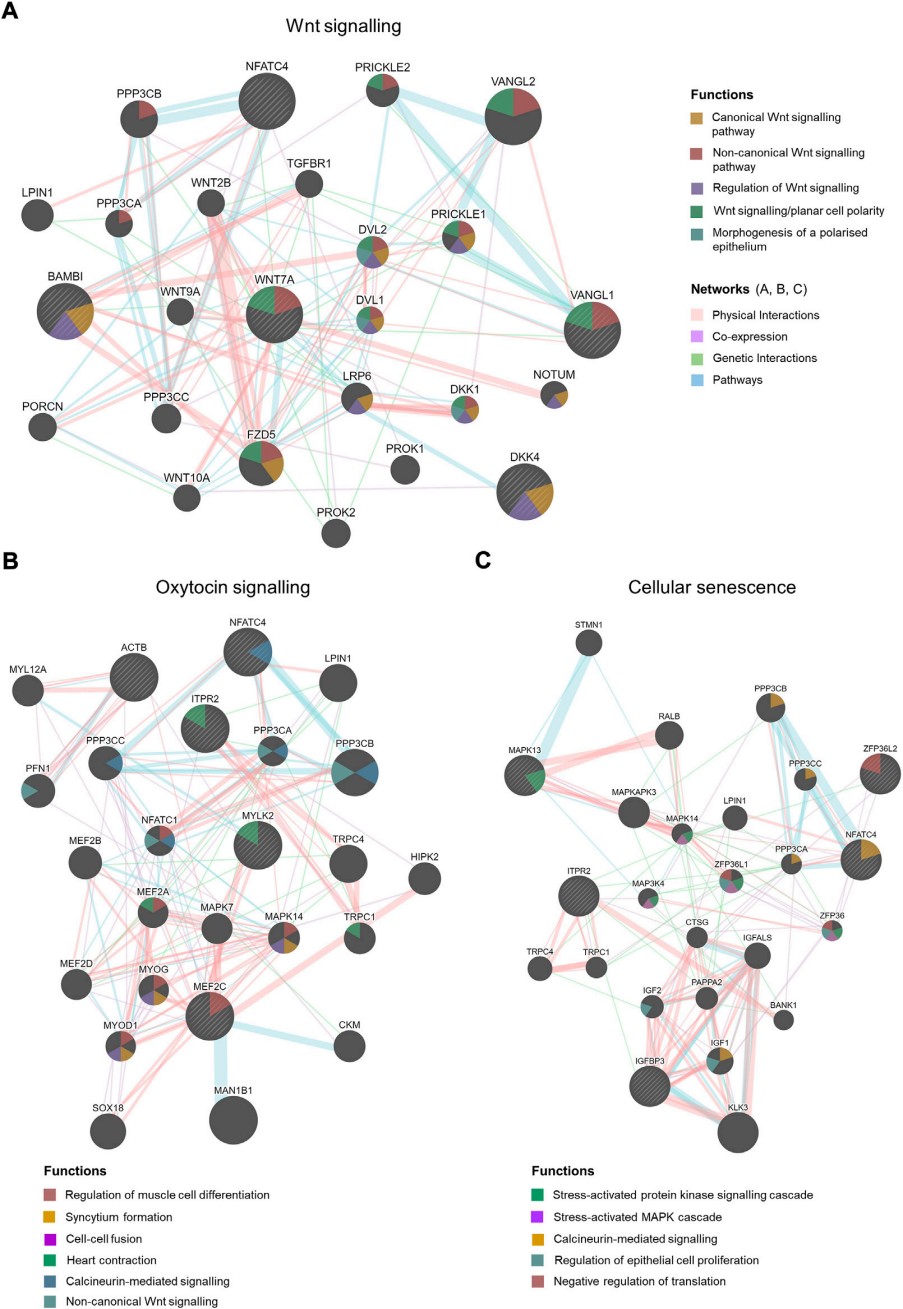
Gene interaction network analysis by Cytoscape plug-in GeneMANIA. Striped bigger nodes indicate genes identified by RNA-seq analysis. Five genes were input in the GeneMANIA to explore the interaction with other genes. Top 20 genes are shown in each network, Wnt signalling **(A)**, Oxytocin signalling **(B)** and Cellular senescence. **(C)** Coloured edges and their connections with other genes indicate nature of interactions. Colour-codes inside each node indicates their biological function as stated in the legends.

Coupled to *RSPO4*, we have identified a significant upregulation of another Wnt signalling ligand *WNT7A* (Log_2_FC = 2.94, P_adj_ = 0.005) in preterm placentas (Figures 1B,C). Gene interaction network analysis using Cytoscape plug-in GeneMANIA (Shannon et al., 2003; Warde-Farley et al., 2010) showed an association of aforementioned Wnt genes with the other 20 key genes that cumulatively regulate canonical and non-canonical Wnt/β-catenin signalling (Figure 4A). Notably, *WNT7A* gene has physical interactions with highest number of other Wnt genes including *WNT2B*, *WNT9A*, *WNT10A*, *FZD5* and *NOTUM* suggesting its major influences in Wnt signalling regulation.

**FIGURE 5:**
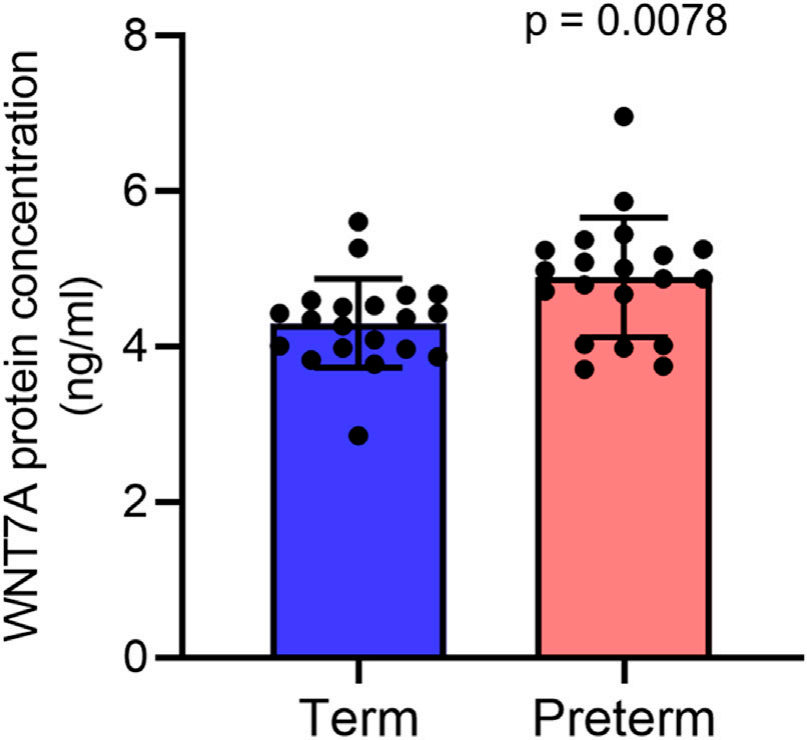
ELISA assay showing WNT7A protein concentrations (ng/ml) in 300 μg/ml total protein in term and preterm placenta VT samples. Data are presented as mean ± standard deviation. Each dot represents protein concentration from an individual subject. *n* = 20 term and 20 preterm. *p* value was determined by Mann-Whitney U test.

Our ELISA assay on villous tissue lysates from 20 term and 20 preterm placentas from the same cohort detected a 14% higher level of WNT7A protein expression in preterm placentas compared to term placentas (4.9 ± 0.8 ng/ml vs. 4.3 ± 0.6 ng/ml, *p* = 0.0078) (Figure 5). Together, our data suggests potential augmentation of Wnt signalling within the villous compartment of the preterm placenta.

The second-ranked signalling pathway was the oxytocin signalling pathway. This was significantly enriched in preterm placenta with 5 significantly upregulated DEGs (*ACTB*, *MEF2C*, *ITPR2*, *NFATC4*, *MYLK2*) (P_adj_ = 0.002). Amongst these, *MEF2C* and *NFATC4* are transcription factors which are activated by oxytocin. Gene interaction network analysis showed that these genes are involved with other network-genes which together regulate muscle cell differentiation (*MEF2C*), muscle contraction (*ITPR2*, *MYLK2*) and calcium-mediated signalling (*NFATC4*) (Figure 4B, Supplementary Table S5).

Trophoblast senescence is a physiological process that progresses gradually as pregnancy advances to term. Premature or accelerated cell senescence due to placental stresses has been implicated in various adverse pregnancies including preeclampsia, fetal growth restriction (FGR) and spontaneous PTB (Sultana et al., 2018). Our signalling pathway analysis identified a significant enrichment of cellular senescence within the preterm placenta villous tissue (P_adj_ = 0.011), which was associated with 5 significantly upregulated DEGs (*ZFP36L2*, *ITPR2*, *IGFBP3*, *NFATC4*, *MAPK13*) (Figure 3E, Supplementary Table S5). These genes, together with other 20 genes in the same network mediate stress-activated protein kinase signalling (*MAPK13*), calcineurin-mediated signalling (*NFATC4*) and negative regulation of translation (*ZFP36L2*) (Figure 4C).

In addition, 4 upregulated genes (*OCLN*, *ACTB*, *MAPK13*, *ICAM1*) significantly enriched leukocyte transendothelial migration signalling (P_adj_ = 0.037), suggesting a potential chemotaxis of inflammatory cells within the vicinity of preterm placenta villous tissue (Figure 3E, Supplementary Table S5). The other key signalling pathway significantly enriched in preterm placenta was the cGMP-PKG signalling pathway (P_adj_ = 0.007), which was associated with 5 upregulated DEGs (*MEF2C*, *ITPR2*, *NFATC4*, *MYLK2*, *ADRA1D*) (Figure 3E). The cGMP-PKG signalling pathway plays pivotal role in platelet activation and vascular smooth muscle contraction and relaxation (Tsai & Kass, 2009).

On the other hand, the downregulated DEGs enriched several important signalling pathways as well, including thermogenesis (P_adj_ = 0.001), metabolic pathways (P_adj_ = 0.001), cell adhesion (P_adj_ = 0.005), herpes simplex virus 1 infection (P_adj_ = 0.015) and PI3K-Akt signalling pathway (P_adj_ = 0.039) (Figure 3F, Supplementary Table S5). 4.8% (27 DEGs) of total downregulated genes were involved in the metabolic pathways suggesting dysregulation of a wide range of metabolic processes in the preterm placenta (Supplementary Table S5). Ten downregulated genes were associated with enrichment of thermogenesis signalling in the preterm placenta. Classically, cold temperature activates thermogenesis in adipocytes *via* a norepinephrine-dependent activation of β3-adrenergic signalling promoting browning of white adipose tissue (Tabuchi & Sul, 2021). However, the role of placental thermogenesis programme in PTB is unknown. Ten downregulated genes (*HLA-DQA2*, *ZNF286A*, *ZNF561*, *ZNF506*, *ZNF90*, *ZNF721*, *CFP*, *ZNF43*, *C5*, *STAT2*, *ZNF676*, *C3*, *ZNF320*) were involved in the enrichment of herpes simplex virus 1 infection. Most of these genes were from zinc finger protein families.

We also noted dysregulated gene expression in preterm placenta which are involved in organic substance transport. For instance, 40 upregulated DEGs including *OCLN*, *SLC26A8*, *SLC6A13*, *SLC25A41*, *OPTN*, *CAVIN1, SLC6A17*, *SLC26A1*, *SLC17A3*, *SLC4A4, SLC5A7*, *SLC16A1* significantly enriched GO term with biological function ‘Organic substance transport’ (P_adj_ = 1.57 × 10^−7^) (Figure 3C, Supplementary Table S4). Majority of these genes were the SLC (Solute linked carrier) gene family of sulfate transporters which are predominantly present in the cytotrophoblasts and syncytiotrophoblasts (Simmons et al., 2013). Of the SLC family genes, *SLC26A8, SLC6A13* and *SLC25A41* were amongst the highly overexpressed genes in the preterm placenta (Log_2_FC > 3.5, P_adj_ < 5 × 10^−3^) (Table 2). Other two genes under this GO term were *OCLN* and *OPTN* which were also amongst the top 50 highly expressed genes in the preterm placenta (Log_2_FC > 3.4, Padj <5 × 10^−4^) (Table 2). *OCLN* (Occludin) plays crucial roles in barrier function, cell polarity and immune defense mechanisms (Balda et al., 1996; Furuse et al., 1996; McCarthy et al., 1996; Hirase et al., 1997; Tsukita et al., 2001).

In addition, we identified two important differentially expressed transcription factors in preterm placenta with multifaceted biological functions, including *KLF8* and *FOXA1* (Table 2). KLF8 (Krüppel-like factor 8), which was upregulated in preterm placenta (Log_2_FC = 3.78, P_adj_ = 2.9 × 10^−8^) (Table 2), is primarily expressed within the syncytiotrophoblasts and vascular endothelial cells of the placenta, and its expression is promoted by the Wnt/β-catenin signalling (Yang et al., 2012; Yang et al., 2014). On the other hand *FOXA1* (Forkhead box A1) was downregulated in the preterm placenta (Log_2_FC = -3.10, P_adj_ = 1.8 × 10^−4^) (Table 2). Downregulation of FOXA1 in the placenta has recently been implicated in early-onset preeclampsia through enhancement of apoptosis and inhibition of migration and invasion of the trophoblastic cells (Zhu et al., 2020). We also noted a significant upregulation of *KRT7* (Keratin 7) in the preterm placenta compared to term placenta (Log_2_FC = 4.66, P_adj_ = 2.1 × 10^−5^) (Table 2; Figure 2C). KRT7 is predominantly expressed in the trophoblast cells (Simmons et al., 2013; Okae et al., 2018), and their upregulation is an indication of cytotrophoblastic overactivity and differentiation during preterm labour.

To determine if our detected differential gene expression in preterm placenta is solely due to variations in gestational ages (GA) between term and preterm groups or it has pathological links with PTB, we compared our DEGs with the previously identified GA-associated placental gene candidates (Supplementary Table S6) (Eidem et al., 2016) following an approach described previously (Lien et al., 2021). We intersected 37 GA-associated genes (Eidem et al., 2016) with our total 901 DEGs, which identified 3 GA-associated genes (*BMP2, DCTN1, APOB*) within our DEGs of preterm placentas. We removed these three genes from our PTB DEGs and conducted GO and signalling pathway enrichment analysis as above. Our re-analysis did not show any major alteration in signalling pathways with either upregulated or downregulated DEGs in preterm placenta compared to term placenta after removal of GA-linked genes (Supplementary Table S7). This evaluation suggests that the gene expression alteration that we detected in preterm placentas are more likely due to the underlying pathomechanism of preterm birth.

Taken together, our data provide molecular evidence of potential trophoblast dysfunction with dysregulated expression of key genes and signalling pathways including Wnt, cellular senescence and oxytocin signalling.

### Diversities in gene expression and signalling pathways between male and female placentas in preterm birth

**FIGURE 6:**
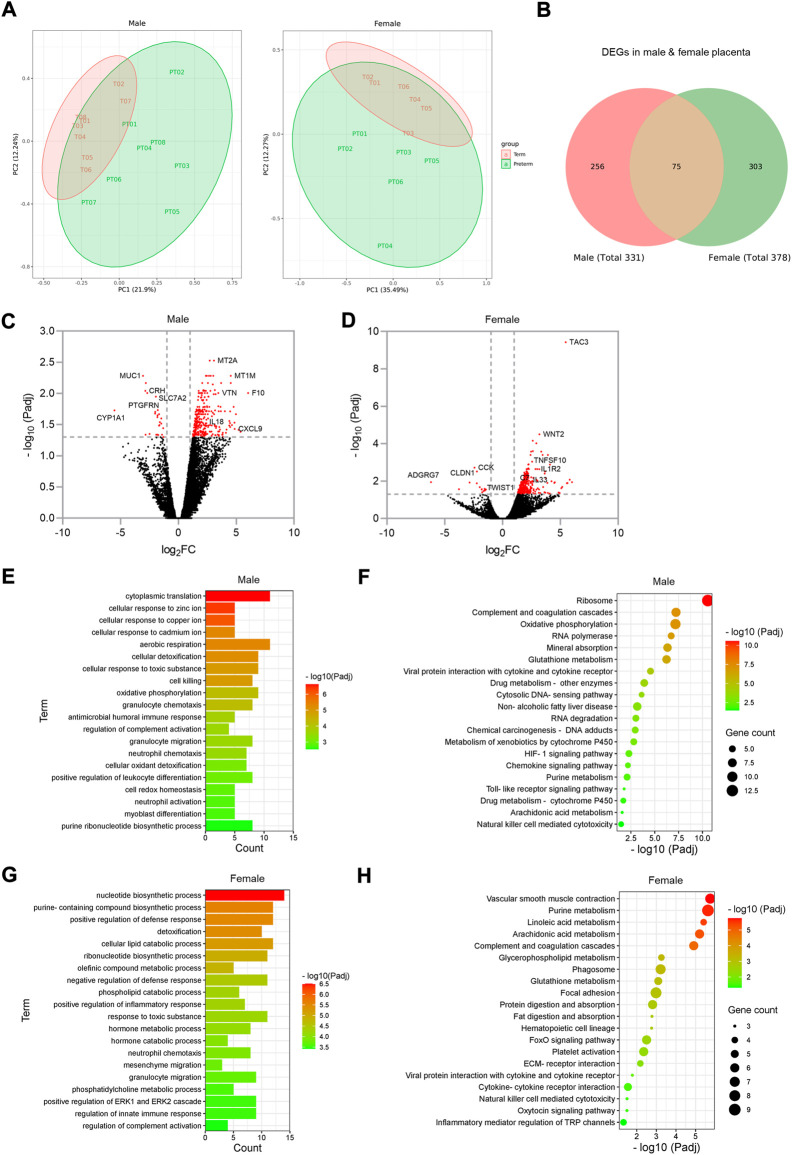
Sub-group gene expression and signalling pathway analysis on male and female placentas. **(A)**. PCA plots showing separation of term and preterm samples in male and female placenta. **(B)**. Venn diagram showing DEGs in male and female preterm placenta and common DEGs among both sexes. **(C,D)**. Volcano plots showing DEGs in male **(C)** and female **(D)** preterm placentas. Horizontal and vertical dotted lines indicate thresholds P_adj_ < 0.05 and Log_2_FC = +/−1. **(E–H)**. GO and KEGG signalling pathways for male (E, F respectively) and female (G, H respectively) preterm placentas. n = 16 males and 12 females (1:1 term vs. preterm).

The male fetus is affected by preterm birth slightly more frequently than the female. Although the underlying mechanism is unclear, pro-inflammatory signalling within the placenta is likely to be involved (McGregor et al., 1992; Challis et al., 2013; Peelen et al., 2016). To examine if the transcriptomic profile of placentas from male PTB varies from that of female PTB, we conducted differential gene expression and signalling pathway analysis. We had 11 male and 9 female births in each of our term and preterm groups (Table 1). Out of 22 male placentas, RNA-seq data from some preterm samples were grouped with data from term samples in our principal component analysis (PCA) (Data not shown). Similar feature was seen in the female placenta group (*n* = 18). Therefore, we selected 8 term and 8 preterm samples from male placenta group, and 6 term and 6 preterm samples from female placenta group, where term and preterm samples were clearly separated from each other in PCA (Figure 6A), which allowed us to conduct a sub-group differential gene expression analysis.

Our differential gene expression analysis by edgeR identified 331 DEGs in male preterm placenta and 378 DEGs in female preterm placenta compared to respective term groups (P_adj_ < 0.05); of them 75 DEGs were common between male and female placenta DEGs (Figure 6B; Table 3, Supplementary Table S8). In the male placenta group, 306 genes were significantly upregulated and 25 genes were downregulated (P_adj_ < 0.05) (Figure 6C). In the female placenta group, 362 genes were upregulated and 16 genes were downregulated (P_adj_ < 0.05) (Figure 6D; Table 3, Supplementary Table S8).

**TABLE 3:** Top 20 upregulated genes in male and female preterm placenta with top 15 common DEGs.

Male placenta			Female placenta		
Genes	Log_2_FC	adj. *p* values	Genes	Log_2_FC	adj. *p* values
F10	6.03	0.0098	RAPGEF4	6.02	0.0113
NCAM2	5.38	0.0412	IQCA1	5.82	0.0084
KNG1	5.20	0.0384	LRRC49	5.63	0.0128
LINC01205	4.97	0.0215	AGR2	5.57	0.0129
CXCL9	4.87	0.0370	TAC3	5.47	0.0000
CCDC141	4.85	0.0293	C8orf31	5.17	0.0205
MT1G	4.77	0.0164	LCN2	5.09	0.0238
CPA1	4.63	0.0321	TRG-AS1	4.95	0.0435
MT1M	4.53	0.0052	DMC1	4.85	0.0410
MT1L	4.53	0.0068	XAGE1B	4.52	0.0115
MKRN2OS	4.48	0.0345	XAGE1A	4.48	0.0125
STAC3	4.47	0.0194	SLC28A2	4.24	0.0102
VTRNA1-2	4.47	0.0299	ALDH1A1	4.10	0.0013
EIF4EBP3	4.41	0.0276	SNAR-H	4.08	0.0461
RHBDL2	4.40	0.0326	PLA2G4C	3.93	0.0431
REN	4.16	0.0380	LTF	3.93	0.0004
GZMB	4.11	0.0427	CHI3L1	3.89	0.0126
VTRNA1-1	4.02	0.0192	SLC43A3	3.76	0.0069
CXCL10	3.84	0.0427	LINC01116	3.72	0.0413
RASA4B	3.84	0.0495	HENMT1	3.68	0.0031

15 Common DEGs in male and female placenta				
		Male		Female	
Genes		Log_2_FC	adj. *p* values	Log_2_FC	adj. *p* values
VTRNA1-1		4.02	0.0192	3.16	0.0279
EPHX1		3.14	0.0321	3.18	0.0023
MT2A		3.07	0.0030	1.77	0.0118
RPL28		3.01	0.0052	1.53	0.0402
ICAM2		2.98	0.0204	2.35	0.0435
BUD31		2.97	0.0072	1.58	0.0383
NNMT		2.71	0.0030	2.56	0.0009
SNHG6		2.61	0.0112	1.71	0.0461
SPP1		2.58	0.0112	2.31	0.0097
VPS28		2.47	0.0160	1.98	0.0060
CINP		2.44	0.0189	2.02	0.0209
WDR41		2.44	0.0326	2.67	0.0068
MT1E		2.37	0.0091	1.65	0.0311
GSTM3		2.29	0.0164	2.05	0.0238
ETFB		2.18	0.0089	1.83	0.0050

Our GO and signalling pathway analysis using the upregulated DEGs by g:Profiler tool significantly enriched several GO terms with diverse biological processes and signalling pathways in both male and female preterm placenta (Figures 6E–H, Supplementary Tables S9, S10, S11, S12). Both male and female preterm placentas revealed involvement of DEGs in inflammation-associated biologicals processes, including granulocyte chemotaxis and migration, neutrophil chemotaxis and migration, leukocyte differentiation, regulation of innate immune response, and positive regulation of inflammatory response (P_adj_ < 0.001) (Figures 6E,G). Amongst the top other biological processes, aerobic respiration (P_adj_ = 4.8 × 10^−6^), oxidative phosphorylation (P_adj_ = 5.9 × 10^−5^), cellular oxidant detoxification (P_adj_ = 7.5 × 10^−4^) and cell redox homeostasis (P_adj_ = 1.3 × 10^−3^) processes were notable in the male preterm placentas; whereas, metabolic and catabolic process of lipids, phospholipids, olefinic compounds and hormones were prominent in the female preterm placentas (P_adj_ < 1.0 × 10^−4^) (Figures 6E,G).

On the other hand, the upregulated DEGs significantly enriched several common signalling pathways in both male and female preterm placentas, including arachidonic acid metabolism (P_adj_ < 0.02), glutathione metabolism (P_adj_ < 7.9 × 10^−4^), purine metabolism (P_adj_ < 8.0 × 10^−3^), natural killer cell mediated cytotoxicity (P_adj_ < 0.04), and complement and coagulation cascades signalling (P_adj_ < 1.2 × 10^−5^). However, oxidative phosphorylation (10 genes, P_adj_ = 6.8 × 10^−8^), HIF-1 signalling pathway (5 genes, P_adj_ = 0.005), chemokine signalling pathway (4 genes, P_adj_ = 0.006) and toll-like receptor signalling pathway (3 genes, P_adj_ = 0.016) were uniquely enriched in the male preterm placentas (Figure 6F, Supplementary Table S11). Whereas, in female preterm placenta, the uniquely enriched signalling pathways were vascular smooth muscle contraction (7 genes, P_adj_ = 1.78 × 10^−6^), platelet activation (6 genes, P_adj_ = 4.4 × 10^−3^), phagosome (7 genes, P_adj_ = 6.0 × 10^−4^), focal adhesion (8 genes, P_adj_ = 0.001), FoxO signalling pathway (6 genes, P_adj_ = 0.003) and oxytocin signalling pathway (3 genes, P_adj_ = 0.031) (Figure 6H, Supplementary Table S13).

In addition, our differential gene expression analysis identified two C-X-C motif chemokine ligands, *CXCL9* and *CXCL10* which were two of the top 20 highly expressed genes (Log_2_FC = 4.9 and Log_2_FC = 3.8 respectively, P_adj_ < 0.05) in the male preterm placenta that were involved in chemokine and toll-like receptor signalling pathways (Table 3). Whereas, phospholipase A2 gamma family gene *PLA2G4C* was the only gene amongst the top 20 highly expressed genes in the female preterm placenta (Log_2_FC = 3.9, P_adj_ < 0.05) that involved in both platelet activation and oxytocin signalling pathways.

Together, our data indicate that inflammation-associated signalling pathways are involved in both male and female preterm placentas, however, inflammation could be more prominent in male placenta compared to female placentas in preterm birth. Furthermore, oxidative phosphorylation and the HIF-1 signalling pathways may play key roles in preterm parturition in male birth. Whereas, vascular smooth muscle contraction, platelet activation and oxytocin signalling pathways could be key mechanisms in female preterm birth.

### miRNA expression in preterm placenta

**FIGURE 7:**
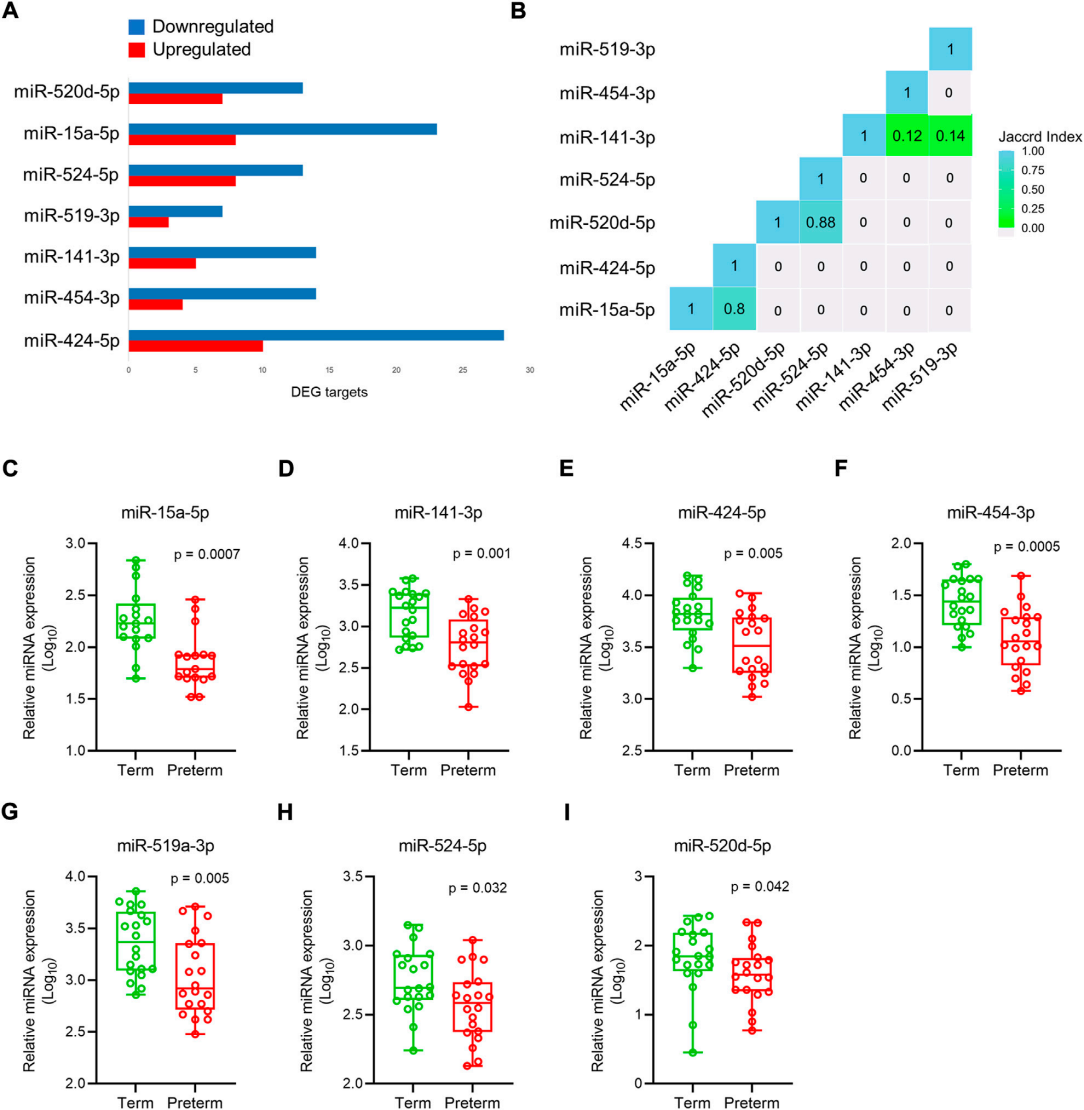
miRNAs expression in placenta. **(A)**. Selected 7 miRNAs and their upregulated and downregulated DEG targets in the preterm placenta. **(B)**. Jaccard index plot represents common genes targeted by selected miRNAs. Each block of colour represents the percentage of miRNAs having common gene targets. **(C–I)**. qRT-PCR validation of selected 7 miRNAs in the term and preterm placentas. The box and whisker plots depict qPCR analysis showing miRNA expression relative to U6 snRNA in term and preterm placenta villous tissue. Each ‘dot’ indicates a mean value of a duplicate qPCR run from an independent subject (*n* = 20 term and 20 preterm). Data are presented as median and interquartile ranges (IQR) with minima and maxima. *p* values were determined by the Mann-Whitney U test.

miRNAs modulate gene function by post-transcriptional and transcriptional regulation of target genes *via* base-pairing with their 3′UTR region (Anton et al., 2017; O'Brien et al., 2018; Sanders et al., 2015). Dysregulated miRNA expression has been implicated in the pathogenesis of PTB and preeclampsia (Pineles et al., 2007; Williams et al., 2012). To identify the miRNA targets within our DEG gene pool, we used miRbase database, validated using starBase (http://starbase.sysu.edu.cn/agoClipRNA.php?source=mRNA) bioinformatics tool, a database for exploring miRNA-mRNA interactions (Yang et al., 2011). We predicted putative targets of 378 miRNAs for 290 up-regulated gene targets, and 450 miRNAs for 498 down-regulated gene targets (Supplementary Table S13). Amongst them seven miRNAs were selected from the list based on their biological relevance and involvements in the adverse pregnancies (Figure 7A, Supplementary Table S13). The Jaccard index plot displays amongst 7 miRNAs, miR-524-5p and miR-520d-5p share 88% of gene targets in common, and miR-15a-5p and miR-424-5p share 80% of gene targets in common which potentially regulate those genes (Figure 7B).

The selected miRNA expressions were validated by qRT-PCR on 40 placenta villous tissue samples (same cohort). The primers (Supplementary Table S2) were specific to the target miRNAs without any primer dimers formation (Supplementary Figure S3A). Data were normalised against U6 snRNA which was expressed equally across the samples (Supplementary Figure S3B). We found all 7 miRNAs to be significantly downregulated in PTB placentas compared to term placentas (Figures 7C–I).

## Discussion

Our bulk transcriptomic study on 20 term and 20 preterm placentas from idiopathic spontaneous PTB reveals several differentially expressed genes which are associated with diverse trophoblast dysfunctions through activation of a number of key signalling pathways including Wnt, oxytocin, cell senescence and cGMP-PKG signalling pathways. The prevailing hypothesis is that the parturition processes in both term and preterm birth follow a common pathway that is predominantly driven by acute inflammatory responses, but that the triggering factors for spontaneous PTB remain elusive (Haddad et al., 2006; Romero et al., 2014). Our global differential gene expression analysis, GO and signalling pathway enrichment did not identify any key inflammatory genes in preterm placenta compared to term, consistent with the hypothesis that inflammation is involved in the common pathway of term and idiopathic spontaneous preterm labour.

However, our sub-group analysis on a selected small number of samples from male and female placentas revealed that several pro-inflammatory signalling pathways including arachidonic acid metabolism and natural killer cell mediated cytotoxicity were involved in both male and female preterm births, whereas chemokine signalling and Toll-like receptor signalling pathways were predominant features in male preterm placentas. Furthermore, our global gene expression analysis (irrespective of fetal sex) identified significant enrichment of leukocyte transendothelial migration signalling pathway driven by 4 upregulated genes (*OCLN, ACTB, MAPK13, ICAM1*) in preterm placenta, suggesting a possible local inflammatory response. Cumulatively, the presence of overt inflammation in preterm villous placenta and its involvement in the pathogenesis of spontaneous PTB cannot be precluded, as previously emphasised (Lien et al., 2020; Lien et al., 2021). In addition, despite the limitation of our sub-group analysis, we conclude that preterm births of male and female babies may be associated with different sets of additional signalling programmes, as also suggested previously (Lien et al., 2021), where male PTB follows oxidative phosphorylation and HIF-1 signalling pathways, and female PTB follows vascular smooth muscle contraction, platelet activation and oxytocin signalling pathways.

In a recent study, Lien et al. demonstrated that male PTB is associated with placental oxidative stress and xenobiotic metabolism pathways (Lien et al., 2021), which is in line with our findings. We have detected several upregulated genes in male preterm placenta which are involved in cellular oxidant detoxification and cell redox homeostasis in concert with activated HIF-1 signalling. Lien et al. also indicated that nutrient sensing and lipid, protein, and carbohydrate metabolism signalling are disrupted in female PTB placentas (Lien et al., 2021). We have also identified dysregulated metabolic and catabolic processes of lipids, phospholipids, olefinic compounds and hormones in the female preterm placentas. It is important to note that our study was conducted on a population where majority of the women were Caucasian White, whereas Lien et al. (Lien et al., 2021) conducted studies on entirely Black women; therefore, variation in our findings may be due to racial variation.

Our data analysis revealed a high level of biological variability in the samples within the same groups. In fact, transcriptome profiles of our eight late preterm samples had similarities with the term group as shown in our PCA. We decided to include all 40 samples in our ‘global’ differential gene expression analysis reflecting the natural distribution of transcriptome profiles in term and preterm placenta in our tested cohort. We have identified 901 DEGs in preterm placentas displaying the magnitude of complexity of underlying molecular mechanisms that lead to spontaneous PTB. However, we were able to identify several key genes which were highly differentially expressed in preterm placenta and were common in various key signalling pathways executing similar biological functions.

The maternal-fetal interface of the human haemochorial placenta is divided into two major components - the fetal part, and the decidual maternal part. The haemochorial placenta consists of (1) chorionic villi, containing an inner layer of villous cytotrophoblasts and outer layer of syncytiotrophoblasts; 2) villous core, containing fetal blood vessels, mesenchymal cells and fetal macrophages (Hofbauer cells) and 3) cytotrophoblasts column, that connect the villi with the decidua (Pereira, 2018). For our study, decidua was removed from the villous tissue, and maternal blood was washed out from the intervillous spaces as much as possible. Our data therefore mostly reflects the transcriptomic micro-milieu of the fetal part of the placenta. However, it should be noted that despite extensive tissue trimming and repeated wash, the villous tissue may have trace amount of maternal blood and decidual cells, which practically cannot be completely removed. In our cohort of participants, the sexes of the offspring were not different between the term and preterm groups, which largely removes the gender-bias previously observed in placental transcriptome profiles (Lien et al., 2021). The villous compartment, particularly cytotrophoblast cells, and a subpopulation of migratory/invasive cytotrophoblasts of the placenta play crucial roles in the maintenance of pregnancy, immune tolerance and fetal growth (Pereira, 2018; Turco & Moffett, 2019).

A histopathological study on 12 placentas suggested that premature accelerated differentiation of the villous cytotrophoblasts is associated with early PTB. This accelerated differentiation was also accompanied with accumulation of senescent syncytiotrophoblast and distal villous hypoplasia, although the underlying molecular mechanism was unexplored (Fitzgerald et al., 2011). Premature or accelerated cell senescence due to placental stresses has been implicated in various adverse pregnancies including preeclampsia, fetal growth restriction (FGR) and spontaneous PTB (Sultana et al., 2018). Here, we report significant enrichment of cellular senescence signalling pathway in preterm placentas, which was driven by five upregulated genes including *IGFBP3*, *NFATC4*, *MAPK13*. Cellular senescence is a state of irreversible and terminal arrest of cell proliferation which is crucially responsible for tissue and organ aging (Burton, 2009; Sultana et al., 2018). Trophoblast senescence is a natural process that progresses gradually as gestational age advances to term (Sultana et al., 2018). Therefore, our observation of cellular senescence at preterm gestations is more likely to be a pathological response, which albeit needs further validation by proteomic studies A number of signalling molecules mediate cellular senescence, however, in placenta mitogen-activated protein kinase (MAPK) mediated signalling plays a pivotal role in placental aging and labour (Behnia et al., 2015; Menon et al., 2016). Elevated levels of chronic stresses, such as oxidative, mitochondrial or endoplasmic reticulum stress, accelerate trophoblast senescence which may affect placental solute transport system resulting in adverse pregnancy outcomes (Burton, 2009). Indeed, several genes were significantly upregulated in our preterm placenta which were involved in stress response (39 genes), cell redox homeostasis (5 genes) and cellular oxidant detoxification (7 genes), as shown in our GO analysis, suggesting a potential dysregulation of oxidative stress responses in trophoblast in PTB.

Furthermore, our GO analysis identified several dysregulated biological processes in preterm villous placentas including organic substance transport (40 genes), cell differentiation (47 genes) and cell motility (17 genes). Out of 40 DEGs of the organic substance transport system, 10 genes were SLC family proteins, and amongst others *OCLN* and *OPTN* genes were highly upregulated in preterm placenta. Two SLC family proteins namely SLC13 and SLC26 act as sulfate transporters in the placenta. SLC26 gene family of sulfate transporters are predominantly present in the cytotrophoblasts, whereas the other sulfate transporter gene family SLC13 is predominantly localised to syncytiotrophoblasts (Simmons et al., 2013). We found a significant upregulation of *SLC26A8* and *SLC26A1* in the preterm villous placenta compared to the term. Sulfate is essential for fetal growth and development and is exclusively supplied by the maternal circulation *via* the placental sulfate transport system (Gaull et al., 1972; Loriette & Chatagner, 1978; Dawson, 2011). Dysregulation of sulfate transporter system may adversely affect fetal development and pregnancy outcome. We have also noted dysregulated expression of *SLC6A13* and *SLC6A17* in the preterm placenta which are involved in amino acids transport. SLC transporters serve as the ‘metabolic gate’ of cells and mediate the transport of a wide range of essential nutrients and metabolites. Their dysregulated expression has been implicated in a wide range of pathologies (Zhang et al., 2019). Our dysregulated SLC transcriptomes in preterm placenta warrants further study to elucidate their role in spontaneous PTB.

A recent *in vitro* study on human trophoblast cell line investigated the molecular mechanism of trophoblast dysfunction and showed that it occurs due to the transcription factor STOX1 (Storkheadbox 1) isoform, a proliferation/differentiation regulator (Nie et al., 2015), and imbalance leading to dysregulated expression of hundreds of genes including *ANXA1, CAPN6, KRT7, C19orf43, SLC6A8, HMGN1* and *ATG7*. This dysregulated gene expression was implicated in cytotrophoblast membrane repair and differentiation defects (Ducat et al., 2020). In our data, we provide transcriptomic evidence of trophoblast dysfunction in the context of PTB. We found dysregulated expressions of several genes including *OCLN, OPTN, KRT7, WNT7A, BAMBI, NFATC4, HDAC9, SLC6A13, SLC6A17* and *SLC26A8* genes in the villous tissue which have functional links to cell motility, differentiation, cell communication, cellular localisation and development (GO analysis).

*OCLN* (Occludin) gene was highly upregulated in our preterm placentas compared to term. OCLN is a tight-junction protein and plays crucial roles in barrier function, cell polarity and host defense mechanism (Balda et al., 1996; Furuse et al., 1996; McCarthy et al., 1996; Hirase et al., 1997; Tsukita et al., 2001). In the term placenta, occludin is predominantly localized at the apical part of the syncytium, and at the cell-cell junction between syncytium and villous cytotrophoblasts; however, the cytotrophoblasts that are located away from the villous stroma lack this protein (Marzioni et al., 2001). This spatial expression pattern of occludin suggests an important role of this protein in cytotrophoblast differentiation and villous syncytialisation. We therefore speculate that upregulation of *OCLN* may have a molecular link to accelerated differentiation of the villous cytotrophoblasts that was previously observed in the preterm placenta (Fitzgerald et al., 2011), which however needs to be verified by further proteomic and functional studies.

We also noted a significant upregulation of pan-trophoblast marker *KRT7* (Keratin 7) (Tsuchida et al., 2020) in the preterm placenta which was the fifth most highly expressed gene in our DEGs list. Upregulation of *KRT7* transcriptome could be a consequence of overactive trophoblasts, or due to their bulk increase, or increased syncytiotrophoblast differentiation within the villous compartment of preterm placenta. This feature was accompanied by a significant upregulation (Log_2_FC = 3.78) of *KLF8*, a pivotal transcription factor, that regulates cell cycle progression and cell invasion (Yang et al., 2014). During the third trimester KLF8 is primarily expressed within the syncytiotrophoblasts and vascular endothelial cells of the placenta (Yang et al., 2014). KLF8 expression is promoted by the Wnt signalling (Yang et al., 2012). Our differential gene expression and pathway analysis data suggest a potential upregulation of the Wnt signalling in the preterm placenta.

Wnt/β-catenin signalling plays pivotal roles in cell polarity, differentiation, proliferation, cell motility, organ morphogenesis and tissue homeostasis. It is regulated by 19 Wnt ligands on 10 frizzled (FZD) receptors, and by 4 R-Spondin family proteins (RSPO1-4) that augment FZD receptor activities (Logan & Nusse, 2004; Mulholland et al., 2005; Nam et al., 2006; de Lau et al., 2014). We have found a significant overexpression of *WNT7A* gene and protein as well as *RSPO4* in the villous compartment of the preterm placenta suggesting a potential overactivation of Wnt/β-catenin signalling. Furthermore, *WNT7A* gene has direct physical interactions with several other key Wnt-associated genes including *WNT2B, WNT9A, WNT10A* and *FZD5* suggesting its pivotal influences in Wnt signalling regulation. To date, the involvement of Wnt signalling in the preterm placenta has remained unclear. Here, for the first time, we report a potential upregulation of Wnt/β-catenin signalling within the villous tissue, which is more likely promoted by two pivotal secretory Wnt ligands, WNT7A and RSPO4. These two proteins could be potential therapeutic targets and biomarkers for spontaneous PTB. We also postulate that the upregulation of *KLF8*, *KRT7* and *OCLN* could be secondary to overactivation of Wnt/β-catenin signalling in the preterm placenta, which albeit needs verification by further studies.

Oxytocin (OT) signalling pathway was also significantly enriched with 5 upregulated genes in our preterm placenta. OT/Oxytocin receptor (OTR) system plays a central role in the mechanism of both term and preterm labour (Kim et al., 2017). OTR is highly expressed in the myometrium, and to a lesser extent in the amnion, chorion and decidua; and its expression is substantially increased at term (Wathes et al., 1999). Infection and inflammation can activate myometrial contraction *via* activation of the oxytocin signalling pathway, leading to both term and preterm labour (Kim et al., 2017). Currently, OTR antagonists are in clinical use as tocolytics for the treatment of preterm labour. However, these OTR antagonist tocolytics, such as atosiban, can only be given intravenously and they delay labour for no more than 48 h. Therefore, identifying novel targets in OT/OTR signalling system is crucial to developing new therapeutics that can be used to prevent spontaneous PTB. Here we identified 5 targets in oxytocin signalling (*ACTB, MEF2C, ITPR2, NFATC4, MYLK2*), of which *ITPR2* (Inositol 1,4,5-trisphosphate receptor type 2) and *MYLK2* (Myosin light chain kinase 2) genes modulate calcium-mediated signalling in myometrial contraction, and can be attractive targets for drug development.

Next, we attempted to explore miRNA targets for the DEGs in our preterm placentas by utilising bioinformatics prediction followed by qRT-PCR validation of a selected panel of miRNAs. Our preliminary assessment of RNA-seq data identified alteration of 3′UTR lengths in the transcriptomes of preterm placentas (data not shown). miRNAs bind with 3′UTR regions of mRNA and modulate gene function. miRNAs are small single-stranded, non-coding RNAs which regulate the expression of target genes at post-transcriptional and translational level (Bartel, 2004). Maternal serum miRNAs have been explored for biomarkers to predict PTB (Gray et al., 2017; Winger et al., 2017; Cook et al., 2019), preeclampsia (Wu et al., 2012) and SGA (Kim et al., 2020). Most of the miRNAs detected in trophoblasts originate from the large cluster C19MC on chromosome 19, which gives rise to 54 mature miRNAs. These miRNAs play an important role in cell proliferation, invasion, and differentiation (Donker et al., 2012). Our bioinformatics analysis on 901 DEGs and literature search identified seven miRNAs, which were all significantly downregulated in the preterm placenta compared to the term group. Amongst these, 4 miRNAs, eg. miR-524-5p and miR-520d-5p share 88% of gene targets (DEGs) in common, and miR-15a-5p and miR-424-5p share 80% of gene targets in common.

A previous qPCR-based study on placenta showed that miRNAs of C19MC, including miR-524-5p and miR-520d-5p were mostly downregulated in preterm pregnancies with PPROM but upregulated in PTB without PPROM (Hromadnikova et al., 2017). In our cohort of preterm placentas, 50% had PPROM. Another study on placentas showed downregulation of C19MC cluster miRNAs including miR-517-5p, miR-519d, miR-520a-5p, miR-525 in FGR and preeclampsia which were associated with disease severity (Hromadnikova et al., 2017). We postulate that miR-524-5p, miR-520d-5p, miR-15a-5p and miR-424-5p may influence differentially expressed genes that were identified in our preterm placentas. Further functional studies may illuminate the mRNA-miRNA interaction of these 4 miRNAs with the key DEGs identified in our study, and their role in the pathogenesis of PTB.

## Study limitation

One of the major limitations in spontaneous PTB research using human placenta samples is the lack of GA-matched healthy placenta controls. In our study, an ideal control would have been GA-matched preterm placentas obtained at planned caesarean section from non-labouring women who do not have any placental dysfunction disorders. However, such a study would be ethically and logistically impossible. Furthermore, there is a significant difference in placental transcriptomic profiles between spontaneously labouring and non-labouring placentas of same gestational ages. Therefore, to assess if our identified differentially expressed genes were due to variations in gestational ages between term and preterm placentas or due to underlying pathology, we removed previously published GA-associated placental genes (Eidem et al., 2016) from our DEG list, following a strategy described previously, for the downstream pathway analysis (Lien et al., 2021). In addition, our identified key signalling pathways including oxytocin and cellular senescence are expected to be elevated at term or late gestational ages, but we have detected that these signalling pathways are elevated in the earlier gestational ages of preterm placentas, suggesting a possible pathological link.

The second limitation of this study was our sub-group analysis on male and female placentas independently to compare gene expression profiles between term and preterm groups. In this sub-group analysis, we selected term and preterm samples for male or female placentas from the entire cohort which were separated from each other in Principal Component analysis. This manual selection of subjects was necessary to conduct differential gene expression analysis as some of the term and preterm samples in both male and female placentas appeared closely resembled in their PC1 and PC2. Despite this limitation, our sub-group analysis demonstrated that male and female fetuses use different molecular pathways during preterm birth, which is in line with previous finding (Lien et al., 2021). Although the selected number of male and female subjects were comparable with the Lien study, further study on a much larger cohort will be required to clarify the picture further given the high level of inherent biological variabilities in the subject population that we observed.

## Conclusion

In summary, we provide fresh transcriptomic evidence of trophoblast dysfunction in preterm birth characterised by aberrant cell motility, differentiation and micronutrient transport mediated by several key molecules and transcription factors including *OCLN*, *OPTN*, *KRT7*, *WNT7A*, *RSPO4*, *BAMBI*, *NFATC4*, *SLC6A13*, *SLC6A17*, *SLC26A8* and *KLF8*. The underpinning signalling pathways for the trophoblast dysfunction and preterm labour could be driven by Wnt, oxytocin and cellular senescence pathways, where augmented Wnt signalling could play a pivotal role due to its multifaceted functions on cell and tissue homeostasis. We also identified two novel potential therapeutic targets (ITPR2 and MYLK2) for modulation of oxytocin signalling to prevent preterm labour. Several of our identified target molecules could also be evaluated for biomarkers for early prediction of preterm birth. However, it is important to note that our inference and conclusion are made mostly based on transcriptomic findings, that will require confirmation by functional studies to better understand the pathogenesis of spontaneous PTB.

## Data Availability

The datasets presented in this study can be found in online repositories. The names of the repository/repositories and accession number(s) can be found below: GEO accession GSE211927: https://www.ncbi.nlm.nih.gov/geo/query/acc.cgi?acc=GSE211927.
