# Supplementary material for: Transcriptomic analysis of the human placenta reveals trophoblast dysfunction and augmented Wnt signalling associated with spontaneous preterm birth
Source: Front Cell Dev Biol. 2022 Oct 24;10:987740. doi: 10.3389/fcell.2022.987740 (PMC9638416; doi:10.3389/fcell.2022.987740)
Supplement: Supplementary file 1 [file DataSheet1.PDF]

Akram et al. Supplementary documents

SUPPLEMENTARY TABLES AND FIGURES:

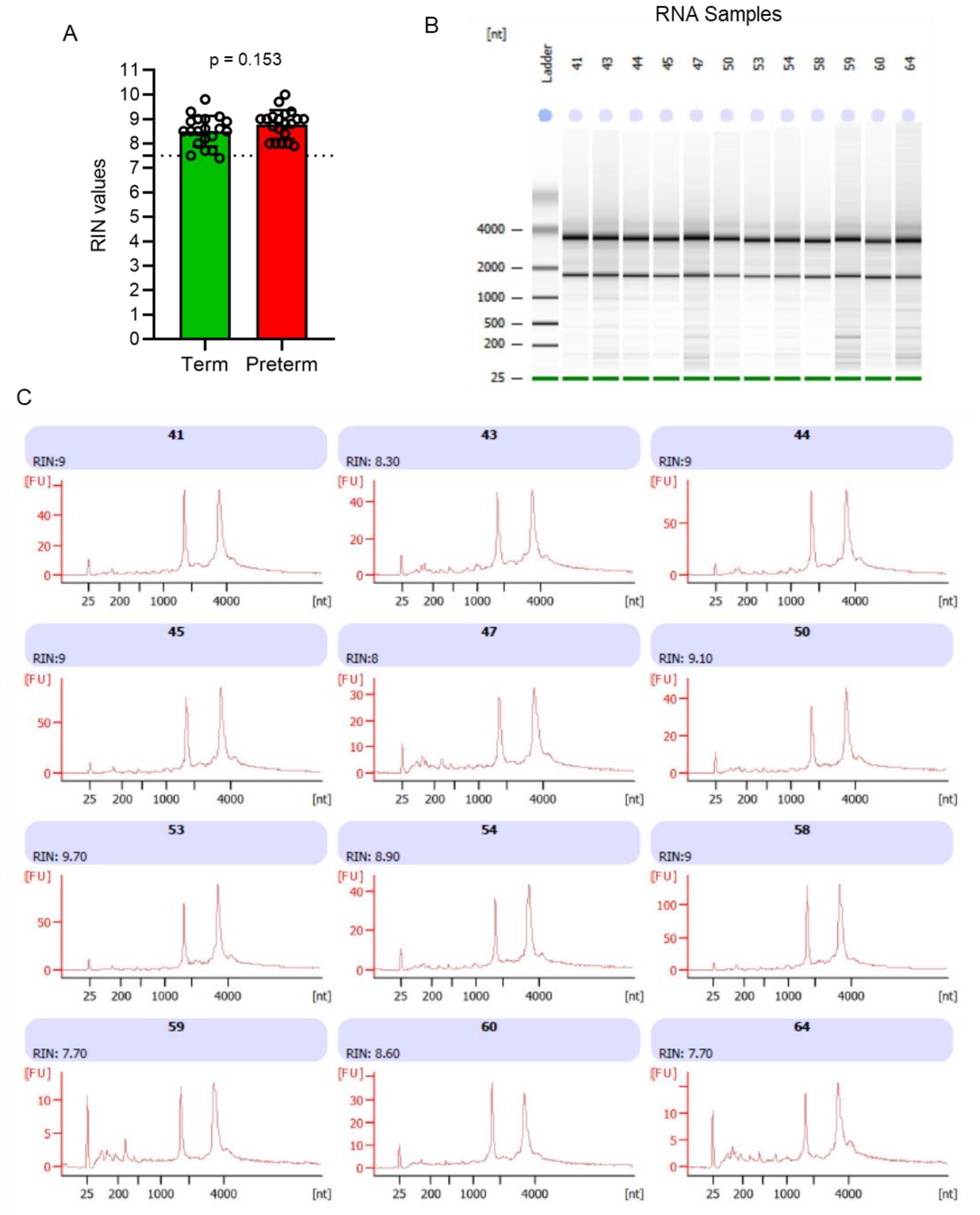

Figure S1: Quality of extracted RNA from placenta VT used for RNA sequencing and qRT-PCR. **A.** The mean RIN values were 8.5 and 8.9 for RNA extracted from term and preterm placenta respectively (p = 0.153), n = 20 for each group. Mann-Whitney U test. **B.** Agilent bioanalyzer gel image showing distinct 18s and 28s bands of RNA with minimal fragmentation. **C.** Agilent bioanalyzer histograms of representative 12 RNA samples.

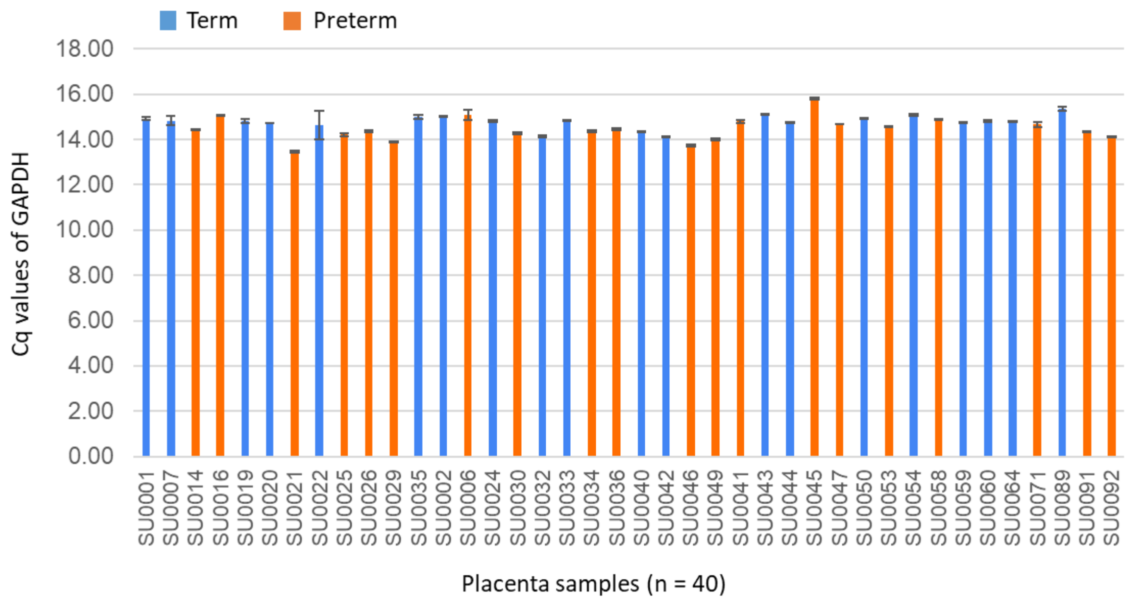

Figure S2: Cq values of *GAPDH*. Blue and orange bars represent term and preterm placentas respectively. Mean (±SD) Cq value = 14.61 ± 0.45 (n = 40). P = 0.1274. One-way repeated measure ANOVA test. Data are presented from two independent qRT-PCR runs for each sample.

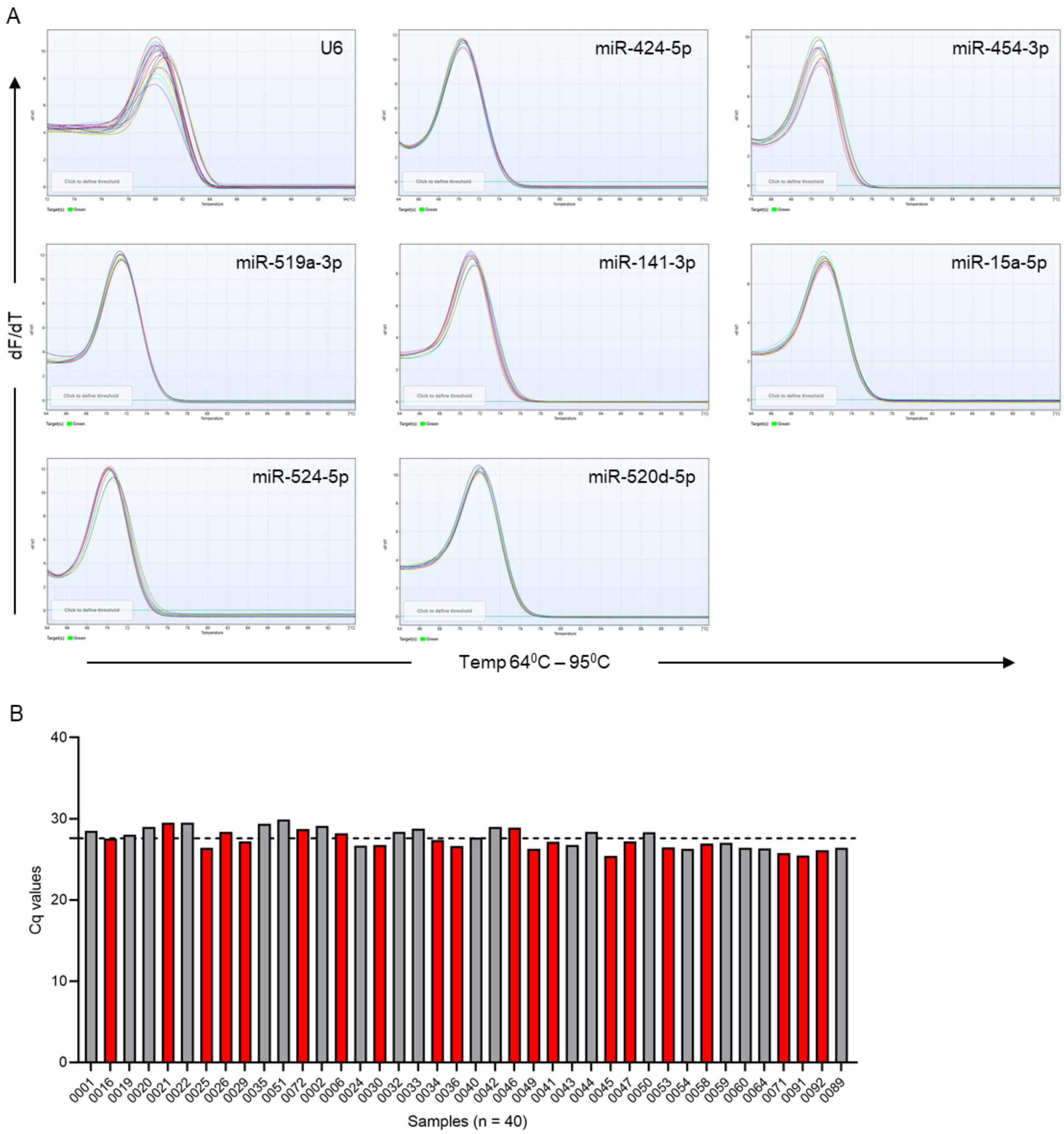

Figure S3. A. Melt curves of the miRNA primers and **B.** Cq values of U6 snRNA internal control used for qPCR for miRNA expression analysis. Dotted line indicates the mean Cq. Grey and Red bars represent term and preterm placentas respectively.

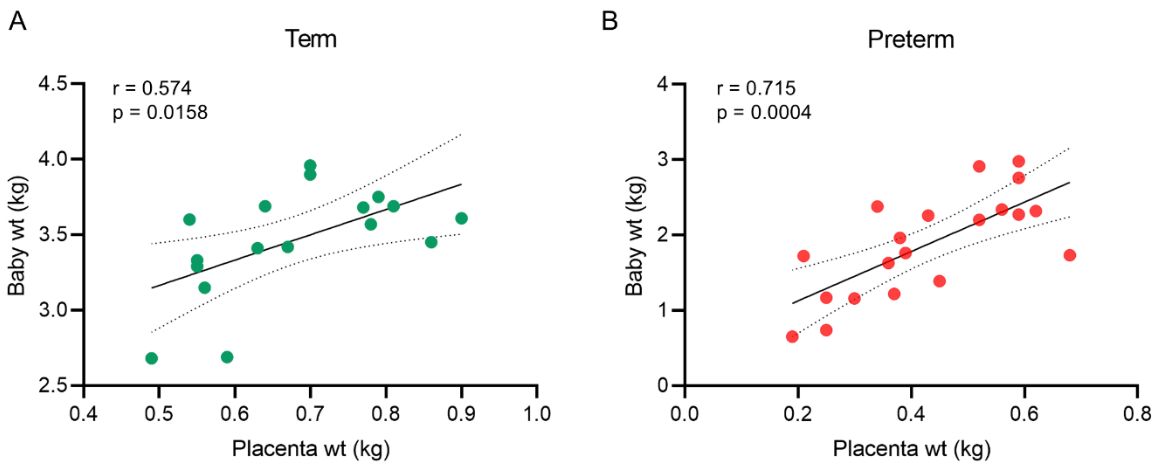

Figure S4: Correlation between placental gross weight and newborn baby weight. Pearson correlation analysis of gross weight of placentas and newborn babies in 17 term (A) and 20 preterm pregnancies (B). Dotted lines represent 95% CI bands of the best-fit lines (Solid).

Table S1: Primers for qRT-PCR validation of a selected panel of DEGs.

Genes	Primers	Cat No	Product code	Company
OCLN	Hs_OCLN_1_SG QuantiTect Primer Assay	249900	QT00081844	Qiagen
RSPO4	Hs_RSPO4_1_SG QuantiTect Primer Assay	249900	QT00057127	Qiagen
KRT7	Hs_KRT7_1_SG QuantiTect Primer Assay	249900	QT00063567	Qiagen
ARF6	Hs_ARF6_1_SG QuantiTect Primer Assay	249900	QT00236824	Qiagen
GAPDH	Hs_GAPDH_1_SG QuantiTect Primer Assay	249900	QT00079247	Qiagen

Table S2. Primers for miRNA validation.

miRNA panel	Primers	Cat No	Product code	Company
hsa-miR-520d-5p	hsa-miR-520d-5p miRCURY LNA miRNA PCR Assay	339306	YP00204684	Qiagen
hsa-miR-424-5p	hsa-miR-424-5p miRCURY LNA miRNA PCR Assay	339306	YP00204736	Qiagen
hsa-miR-454-3p	hsa-miR-454-3p miRCURY LNA miRNA PCR Assay	339306	YP00205663	Qiagen
hsa-miR-141-3p	hsa-miR-141-3p miRCURY LNA miRNA PCR Assay	339306	YP00204504	Qiagen
hsa-miR-15a-5p	hsa-miR-15a-5p miRCURY LNA miRNA PCR Assay	339306	YP00204066	Qiagen
hsa-miR-519a-3p	hsa-miR-519a-3p miRCURY LNA miRNA PCR Assay	339306	YP00205919	Qiagen
hsa-miR-524-5p	hsa-miR-524-5p miRCURY LNA miRNA PCR Assay	339306	YP00204135	Qiagen
U6 snRNA	U6 snRNA miRCURY LNA miRNA PCR Assay	339306	YP00203907	Qiagen

Table S3: Complete significantly identified DEGs table

Upregulated DEGs

background	hgnc_symbol	start_position	end_position	size	logFC	AveExpr	t	P.Value	adj.P.Val	B
ENSG00000002746	HECW1	43112629	43566001	4.53E+05	1.09E+00	-0.3856674	2.8116767	7.75E-03	0.0139114	-33.966245
ENSG00000004846	ABCB5	20615667	20777038	1.61E+05	2.69E+00	-0.6375058	5.88609737	8.11E-07	4.96E-04	-25.119831
ENSG00000006071	ABCC8	17392498	17476894	8.44E+04	2.70E+00	-0.2684128	4.36196253	9.49E-05	0.00502254	-29.618425
ENSG00000007384	RHBDF1	58059	76355	1.83E+04	2.81E+00	-0.351925	6.05794974	4.71E-07	0.00031749	-24.315545
ENSG00000007545	CRAMP1	1612337	1677908	6.56E+04	1.87E+00	-0.0514993	2.82225403	7.54E-03	0.01354543	-33.606976
ENSG00000008196	TFAP2B	50818723	50847619	2.89E+04	2.03E+00	-0.623528	2.97489461	0.00506781	0.00914598	-33.721388
ENSG00000010219	DYRK4	4562204	4615302	5.31E+04	1.66E+00	0.32454566	2.40082727	2.14E-02	0.037606	-33.548703
ENSG00000010379	SLC6A13	220621	262873	4.23E+04	3.99E+00	0.00913444	9.86209709	4.93E-12	2.66E-08	-12.2558
ENSG00000013588	GPRC5A	12891559	12917937	2.64E+04	1.19E+00	-0.4687317	2.43749013	1.96E-02	0.0345398	-34.853303
ENSG00000023734	STRAP	15882387	15903478	2.11E+04	2.46E+00	0.14898085	3.4442561	1.41E-03	5.02E-03	-31.242101
ENSG00000033011	ALG1	5033702	5087379	5.37E+04	1.29E+00	-0.3846344	2.56965692	1.42E-02	0.02527798	-34.527504
ENSG00000047365	ARAP2	35948221	36244514	2.96E+05	1.55E+00	0.43049073	3.22240394	2.61E-03	0.00502254	-31.426845
ENSG00000047644	WWC3	10015254	10144474	1.29E+05	2.53E+00	0.32353434	3.11533382	3.49E-03	0.00631378	-31.924089
ENSG00000048052	HDAC9	18086949	19002416	9.15E+05	2.04E+00	0.29493475	2.46887511	1.82E-02	0.03210252	-33.411281
ENSG00000048740	CELF2	10798397	11336675	5.38E+05	1.39E+00	-0.3854028	2.66163977	1.13E-02	0.02021567	-34.318768
ENSG00000050426	LETMD1	51047962	51060424	1.25E+04	1.54E+00	0.04882775	2.6462126	1.18E-02	0.02098771	-33.550803
ENSG00000056998	GYG2	2828822	2882820	5.40E+04	2.84E+00	-0.1318139	4.45341261	7.18E-05	0.00502254	-28.937204
ENSG00000064547	LPAR2	19623655	19628930	5.28E+03	1.14E+00	-0.5292913	2.8741227	6.60E-03	0.01186564	-33.924449
ENSG00000065308	TRAM2	52497408	52577060	7.97E+04	1.38E+00	-0.5189503	3.07810472	3.85E-03	0.00697219	-33.41769
ENSG00000065320	NTN1	9021510	9244000	2.22E+05	2.05E+00	-0.5098242	2.92305	5.81E-03	0.01046556	-33.765272
ENSG00000068912	ERLEC1	53787044	53818819	3.18E+04	2.85E+00	0.02931024	3.9052214	3.73E-04	0.00502254	-30.030114
ENSG00000069020	MAST4	66596361	67169595	5.73E+05	3.42E+00	-0.0722532	5.36986793	4.14E-06	0.00214684	-25.729136
ENSG00000071189	SNX13	17790761	17940501	1.50E+05	1.13E+00	1.17101664	2.36546175	2.32E-02	0.04077124	-33.148106
ENSG00000072422	RHOBTB1	60869438	61001440	1.32E+05	1.83E+00	-0.432785	2.84173403	0.0071719	0.01288934	-33.82687
ENSG00000073464	CLCN4	10156975	10237660	8.07E+04	1.17E+00	-0.6480313	3.68481912	7.10E-04	0.00502254	-31.885031
ENSG00000075624	ACTB	5526409	5563902	3.75E+04	3.22E+00	0.04992076	4.54291849	5.46E-05	0.00502254	-28.260388
ENSG00000080493	SLC4A4	71062667	71572087	5.09E+05	1.09E+00	-0.3821022	2.78767778	8.24E-03	0.01477854	-34.023182
ENSG00000081189	MEF2C	88717117	88904257	1.87E+05	3.00E+00	0.09433083	2.51209512	1.64E-02	0.02898925	-33.470127
ENSG00000084093	REST	56907876	56966808	5.89E+04	1.94E+00	-0.4570293	3.02905065	4.39E-03	7.94E-03	-33.499081
ENSG00000089063	TMEM230	5068232	5113103	4.49E+04	1.08E+00	-0.4183005	2.75544671	8.94E-03	0.01602	-34.107386

ENSG00000090339	ICAM1	10271093	10286615	1.55E+04	1.41E+00	0.12855596	4.02577757	0.00026107	0.00502254	-29.461049
ENSG00000095739	BAMBI	28677510	28682932	5.42E+03	3.63E+00	0.00647303	6.74782952	5.36E-08	5.46E-05	-21.716731
ENSG00000099625	CBARP	1228287	1238027	9.74E+03	1.22E+00	-0.5992459	2.51599772	1.62E-02	0.02872309	-34.782613
ENSG00000099797	TECR	14517085	14565980	4.89E+04	4.45E+00	-0.0246042	7.35421877	8.12E-09	1.22E-05	-19.763051
ENSG00000099864	PALM	708935	748329	3.94E+04	1.44E+00	-0.0115998	2.99708251	4.78E-03	0.00863093	-32.30985
ENSG00000100461	RBM23	22893204	22919182	2.60E+04	1.83E+00	-0.5393294	3.61577806	8.65E-04	0.00502254	-31.941961
ENSG00000100926	TM9SF1	24189149	24195687	6.54E+03	1.10E+00	-0.8171828	2.61067867	1.29E-02	0.02288957	-34.81005
ENSG00000100968	NFATC4	24365673	24379604	1.39E+04	1.97E+00	0.11126967	2.52821095	1.57E-02	0.02790363	-33.513151
ENSG00000101282	RSPO4	958452	1002311	4.39E+04	5.04E+00	-0.4043402	12.4034307	6.07E-15	1.09E-10	-5.7986081
ENSG00000101306	MYLK2	31819308	31834689	1.54E+04	1.81E+00	-0.3602751	2.6717826	0.01104346	0.01971778	-34.168672
ENSG00000101955	SRPX	38149336	38220924	7.16E+04	1.45E+00	-0.668301	3.53518148	1.09E-03	0.00502254	-32.318594
ENSG00000102349	KLF8	56232356	56291531	5.92E+04	3.78E+00	-0.4760678	9.8033054	5.81E-12	2.85E-08	-12.969619
ENSG00000103245	CIAO3	729760	741329	1.16E+04	1.35E+00	-0.5927298	2.64140689	1.19E-02	2.12E-02	-34.49405
ENSG00000104371	DKK4	42374063	42377229	3.17E+03	1.30E+00	-0.4831213	2.81074249	0.00776693	0.01394292	-34.04419
ENSG00000104679	R3HCC1	23270120	23296279	2.62E+04	1.01E+00	-0.8312084	3.45585239	1.36E-03	0.00502254	-32.718567
ENSG00000105364	MRPL4	10251901	10260055	8.15E+03	1.45E+00	-0.4658649	2.98418276	4.95E-03	0.00892787	-33.610563
ENSG00000105643	ARRDC2	18001132	18014102	1.30E+04	1.32E+00	-0.6319229	2.353735	2.39E-02	0.04187225	-35.206544
ENSG00000106633	GCK	44143213	44198170	5.50E+04	2.20E+00	0.16456229	2.97181402	5.11E-03	0.00921962	-32.436395
ENSG00000108298	RPL19	39200283	39204840	4.56E+03	2.89E+00	-0.0473579	4.5609455	5.16E-05	5.02E-03	-28.184447
ENSG00000108352	RAPGEFL1	40177010	40195656	1.86E+04	2.30E+00	0.13704183	2.75935757	0.00885577	0.01586901	-32.921248
ENSG00000108582	CPD	30378927	30469989	9.11E+04	1.16E+00	2.43093462	2.43814078	1.95E-02	0.03448854	-32.614185
ENSG00000108666	C17orf75	32324565	32350023	2.55E+04	2.24E+00	-0.4793164	3.92756114	3.49E-04	0.00502254	-31.081761
ENSG00000108733	PEX12	35574795	35578863	4.07E+03	1.65E+00	-0.4647494	2.90334516	6.11E-03	0.01100869	-33.65482
ENSG00000109099	PMP22	15229773	15272292	4.25E+04	4.26E+00	-0.5302545	6.96683464	2.70E-08	3.10E-05	-21.494922
ENSG00000111203	ITFG2	2812622	2859791	4.72E+04	1.44E+00	-0.5080197	3.0373496	4.29E-03	0.00776525	-33.49268
ENSG00000111224	PARP11	3791047	3873448	8.24E+04	3.32E+00	-0.2361429	6.84552286	3.95E-08	4.26E-05	-21.734646
ENSG00000111254	AKAP3	4615508	4649051	3.35E+04	1.75E+00	-0.6567744	3.51726343	1.15E-03	5.02E-03	-32.347319
ENSG00000111339	ART4	14825569	14843526	1.80E+04	2.30E+00	0.04146659	2.8462815	7.09E-03	0.01274241	-32.785062
ENSG00000111652	COPS7A	6724014	6731875	7.86E+03	1.07E+00	-0.7436582	2.68197962	1.08E-02	0.01922732	-34.575777
ENSG00000112053	SLC26A8	35943516	36024868	8.14E+04	4.11E+00	-0.3525301	11.881041	2.25E-14	2.43E-10	-7.1554431
ENSG00000112208	BAG2	57172326	57189833	1.75E+04	1.17E+00	-0.6515491	2.89816588	6.20E-03	0.0111571	-33.943974
ENSG00000114062	UBE3A	25333728	25439051	1.05E+05	1.56E+00	-0.6103266	2.44949091	1.90E-02	3.36E-02	-34.948004
ENSG00000114302	PRKAR2A	48744597	48847874	1.03E+05	2.33E+00	0.11631632	4.06543166	2.32E-04	0.00502254	-29.41612
ENSG00000115163	CENPA	26764289	26801067	3.68E+04	4.54E+00	-0.1223125	5.70442999	1.44E-06	0.00082618	-25.268477
ENSG00000115641	FHL2	105357712	105438513	8.08E+04	3.20E+00	-0.5158741	5.10628134	9.49E-06	0.00456462	-27.316029
ENSG00000115665	SLC5A7	107986523	108013994	2.75E+04	1.06E+00	-0.576599	3.08938186	3.74E-03	0.00676592	-33.403642

ENSG00000116205	TCEANC2	54053584	54112519	5.89E+04	1.52E+00	-0.4818029	2.53096649	1.56E-02	0.02772013	-34.648401
ENSG00000116771	AGMAT	15571699	15585051	1.34E+04	2.97E+00	-0.2262009	6.33343737	1.97E-07	0.00016125	-23.37747
ENSG00000116786	PLEKHM2	15684320	15734769	5.04E+04	3.76E+00	-0.1952066	9.4239206	1.70E-11	6.65E-08	-13.858088
ENSG00000117151	CTBS	84549611	84574480	2.49E+04	1.13E+00	-0.2733612	2.62665604	1.24E-02	0.02201388	-34.187951
ENSG00000117242	PINK1-AS	20642657	20652193	9.54E+03	2.55E+00	-0.6893892	6.36244792	1.80E-07	1.52E-04	-23.704044
ENSG00000118620	ZNF430	21020634	21060050	3.94E+04	1.22E+00	-0.8091472	3.31241611	0.00203477	0.00502254	-32.987248
ENSG00000119801	YPEL5	30146941	30160533	1.36E+04	1.73E+00	-0.5357694	3.07791486	3.85E-03	0.0069755	-33.431098
ENSG00000119862	LGALSL	64453969	64461381	7.41E+03	1.12E+00	-0.5490616	3.1005383	3.63E-03	0.00656776	-33.366441
ENSG00000120549	KIAA1217	23694746	24547848	8.53E+05	1.79E+00	0.43990027	2.41831369	2.05E-02	3.61E-02	-33.455593
ENSG00000120925	RNF170	42849637	42897290	4.77E+04	1.24E+00	-0.6065871	2.57934333	1.39E-02	0.02469181	-34.682799
ENSG00000121318	TAS2R10	10825317	10826358	1.04E+03	1.30E+00	-0.4152649	2.3861343	2.21E-02	0.03888829	-34.874924
ENSG00000122254	HS3ST2	22814162	22916338	1.02E+05	1.35E+00	-0.7595684	3.24914734	2.42E-03	0.00502254	-33.123984
ENSG00000122420	PTGFR	78303884	78540701	2.37E+05	2.24E+00	0.19580064	2.48044718	1.77E-02	0.03123468	-33.365887
ENSG00000122432	SPATA1	84506300	84566194	5.99E+04	3.32E+00	-0.1444503	3.69123989	6.96E-04	0.00502254	-31.363659
ENSG00000122477	LRRC39	100148448	100178273	29825	1.44E+00	-0.6993405	2.66652667	1.12E-02	0.01997516	-34.51637
ENSG00000122566	HNRNPA2B1	26171151	26201529	3.04E+04	1.03E+00	-0.7650099	2.53336082	1.55E-02	0.02756374	-34.874696
ENSG00000123104	ITPR2	26335352	26833194	4.98E+05	2.60E+00	0.06273382	5.89250926	7.95E-07	0.00049254	-24.026486
ENSG00000123179	EBPL	49660674	49691486	3.08E+04	2.39E+00	-0.6152066	4.58599498	4.78E-05	5.02E-03	-29.196762
ENSG00000123240	OPTN	13099449	13138308	3.89E+04	3.49E+00	-0.503171	9.41896398	1.73E-11	6.65E-08	-14.00239
ENSG00000124564	SLC17A3	25833066	25882286	4.92E+04	1.13E+00	-0.7373278	2.69620523	1.04E-02	0.01856437	-34.482628
ENSG00000125845	BMP2	6767686	6780246	1.26E+04	1.39E+00	-0.7515635	3.66056289	7.61E-04	0.00502254	-31.978395
ENSG00000127124	HIVEP3	41506365	42035925	529560	1.13E+00	-0.5356061	3.26954811	2.29E-03	0.00502254	-32.928447
ENSG00000128185	DGCR6L	20314238	20320080	5842	2.78E+00	-0.2284531	4.0802744	2.22E-04	0.00502254	-30.267589
ENSG00000128731	HERC2	28111040	28322179	211139	1.70E+00	-0.5559109	2.86336645	6.78E-03	0.01219727	-33.924164
ENSG00000128805	ARHGAP22	48446036	48656265	2.10E+05	2.41E+00	0.14456518	3.14244041	3.24E-03	0.00587254	-31.990098
ENSG00000130005	GAMT	1397026	1401570	4544	1.06E+00	-0.3090143	2.48061697	1.77E-02	0.03122295	-34.63071
ENSG00000130038	CRACR2A	3606633	3764819	1.58E+05	4.85E+00	-0.2040612	10.9574196	2.48E-13	1.67E-09	-9.5633281
ENSG00000131080	EDA2R	66595637	66639298	43661	2.23E+00	0.04642231	3.38167551	1.68E-03	0.00502254	-31.579645
ENSG00000131969	ABHD12B	50872053	50904970	3.29E+04	3.59E+00	-0.244307	7.21258452	1.26E-08	1.79E-05	-20.546769
ENSG00000132518	GUCY2D	8002615	8020342	17727	1.83E+00	0.26987804	3.84560304	4.44E-04	5.02E-03	-29.873958
ENSG00000132938	MTUS2	28820339	29505947	6.86E+05	1.09E+00	-0.8817446	3.24360235	2.46E-03	0.00502254	-33.32486
ENSG00000134461	ANKRD16	5861616	5889906	28290	1.45E+00	-0.5651256	3.01646755	0.00454009	0.00820348	-33.608715
ENSG00000134480	CCNH	87318416	87412930	94514	1.16E+00	-0.6395899	2.40826442	2.10E-02	0.03695982	-35.073208
ENSG00000134531	EMP1	13196723	13219941	23218	2.65E+00	-0.0973357	3.55423488	1.03E-03	0.00502254	-31.712182
ENSG00000134759	ELP2	36129444	36180557	51113	1.12E+00	-0.5704935	2.59911545	0.01322684	0.02354126	-34.614517
ENSG00000135226	UGT2B28	69280475	69295050	14575	1.98E+00	0.2637239	2.3913878	2.18E-02	3.84E-02	-33.684356

ENSG00000135447	PPP1R1A	54575387	54588659	13272	2.61E+00	0.21808187	2.96536878	5.20E-03	0.00937526	-32.379222
ENSG00000135480	KRT7	52232520	52252186	19666	4.66E+00	-0.0272437	7.14943305	1.53E-08	2.06E-05	-20.59191
ENSG00000136068	FLNB	58008398	58172251	163853	1.32E+00	0.27613097	4.1573326	1.76E-04	0.00502254	-29.011935
ENSG00000136367	ZFHX2	23520857	23556192	35335	3.16E+00	-0.0601533	5.45244675	3.19E-06	0.00170396	-25.96441
ENSG00000136936	XPA	97674909	97697340	22431	1.01E+00	0.11771226	3.059497	4.05E-03	7.32E-03	-32.184039
ENSG00000137078	SIT1	35649295	35650950	1655	3.98E+00	-0.4138133	7.65094927	3.26E-09	5.86E-06	-19.24112
ENSG00000137198	GMPR	16238587	16295549	56962	1.13E+00	1.15592928	2.48691551	0.01738617	0.03076832	-32.893954
ENSG00000137225	CAPN11	44158811	44184401	25590	1.03E+00	-0.5663601	2.9717975	0.00510935	0.00921971	-33.696087
ENSG00000137941	TTLL7	83865024	83999150	134126	3.58E+00	-0.2859085	8.21159559	5.95E-10	1.34E-06	-17.477047
ENSG00000137965	IFI44	78649796	78664078	14282	2.66E+00	-0.2129905	3.89604943	0.00038325	0.00502254	-30.803751
ENSG00000138760	SCARB2	76158737	76234536	75799	2.83E+00	-0.4641112	5.87926054	8.29E-07	0.00049649	-25.10366
ENSG00000139155	SLCO1C1	20695332	20753386	58054	2.94E+00	0.06646439	3.90023953	0.00037855	0.00502254	-30.166242
ENSG00000140990	NDUFB10	1959538	1961975	2437	1.09E+00	-0.709492	2.63234107	0.01218344	0.02171002	-34.603965
ENSG00000141314	RHBDL3	32265832	32324659	58827	4.00E+00	-0.3080733	11.383782	8.08E-14	6.22E-10	-8.4359943
ENSG00000141384	TAF4B	26226445	26391685	165240	1.07E+00	-0.3126294	2.68928092	0.01056996	0.01888423	-34.243852
ENSG00000141738	GRB7	39737927	39747291	9364	3.62E+00	-0.2221306	3.45575862	0.00136365	0.00502254	-31.675813
ENSG00000141748	ARL5C	39156894	39167484	10590	1.32E+00	-0.5761327	3.07702295	0.00386323	0.00699172	-33.435002
ENSG00000141933	TPGS1	507497	519654	12157	2.32E+00	-0.0507443	5.01756072	1.25E-05	0.00502254	-26.612223
ENSG00000142449	FBN3	8065402	8149592	84190	2.04E+00	0.25384249	2.60572183	0.01301305	0.02316535	-33.142553
ENSG00000145217	SLC26A1	979073	993440	14367	1.38E+00	-0.8014937	3.24067878	0.00247928	0.00502254	-33.243336
ENSG00000145536	ADAMTS16	5140330	5320304	179974	1.21E+00	-0.7063901	3.80502516	0.00050047	0.00502254	-31.563015
ENSG00000145685	LHFPL2	78485215	78770021	284806	2.17E+00	0.22769827	2.94283327	0.0055136	0.00994086	-32.46229
ENSG00000146556	WASH2P	113588550	113599043	10493	1.13E+00	-0.8236765	3.06682644	0.0039701	0.00718441	-33.668031
ENSG00000146674	IGFBP3	45912245	45921874	9629	2.34E+00	0.13829191	3.10495078	0.00358425	0.00649096	-32.134824
ENSG00000149100	EIF3M	32583798	32606264	22466	4.16E+00	-0.0581489	12.2202028	9.58E-15	1.29E-10	-6.1661392
ENSG00000151240	DIP2C	274190	689668	415478	2.24E+00	0.14499672	2.63013127	0.01225041	0.02182791	-33.20376
ENSG00000152518	ZFP36L2	43222402	43226606	4204	3.55E+00	-0.2733834	7.17364939	1.42E-08	1.96E-05	-20.674299
ENSG00000154764	WNT7A	13816258	13880071	63813	2.94E+00	-0.094867	4.33670932	0.00010248	0.00502254	-29.4485
ENSG00000155380	SLC16A1	112911847	112957593	45746	1.05E+00	-0.4814452	3.11603276	0.00347892	0.00630233	-33.30387
ENSG00000155761	SPAG17	117953590	118185228	231638	1.75E+00	-0.7858405	3.76734466	0.00055858	0.00502254	-31.778742
ENSG00000156711	MAPK13	36127809	36144524	16715	1.43E+00	0.18567591	2.94301411	0.00551098	0.00993647	-32.322071
ENSG00000157111	TMEM171	73120569	73131809	11240	3.86E+00	-0.5602708	8.76039488	1.17E-10	3.30E-07	-16.02512
ENSG00000157168	NRG1	31639222	32855666	1216444	1.94E+00	0.17416616	2.53217718	0.01558232	0.02764047	-33.446766
ENSG00000157211	CDCP2	54132968	54153770	20802	1.86E+00	-0.4723374	2.84398697	0.00713035	0.01281722	-33.963867
ENSG00000157954	WIPI2	5190196	5233840	43644	1.09E+00	-0.4096196	2.83766288	0.00724757	0.01302228	-33.918719
ENSG00000159339	PADI4	17308195	17364004	55809	1.14E+00	-0.6942581	2.36549108	0.02320332	0.04076977	-35.241998

ENSG00000160321	ZNF208	21932958	22010949	77991	2.15E+00	-0.5547149	3.51403776	0.0011566	0.00502254	-32.278452
ENSG00000162374	ELAVL4	50024029	50203772	179743	1.64E+00	-0.2560237	3.19134613	0.002837	0.0051474	-32.874931
ENSG00000162571	TTLL10	1173880	1197936	24056	2.44E+00	-0.6832642	4.19329152	0.00015814	0.00502254	-30.453579
ENSG00000162613	FUBP1	77944055	77979110	35055	2.30E+00	-0.2746967	2.8036593	0.00790924	0.01419601	-33.708761
ENSG00000162627	SNX7	98661701	98760500	98799	2.32E+00	-0.2493078	3.41812632	0.00151575	0.00502254	-32.173746
ENSG00000164631	ZNF12	6688433	6706947	18514	1.45E+00	-0.5702218	3.14795711	0.00319162	0.00578672	-33.254435
ENSG00000164669	INTS4P1	65141032	65234216	93184	1.29E+00	-0.571882	3.06243096	0.00401703	0.00726787	-33.476096
ENSG00000164746	C7orf57	48035511	48061304	25793	2.98E+00	0.12422879	3.85273629	0.00043526	0.00502254	-30.123326
ENSG00000164818	DNAAF5	726699	786475	59776	3.30E+00	-0.0131713	4.1600479	0.00017476	0.00502254	-29.788352
ENSG00000164877	MICALL2	1428465	1459470	31005	1.02E+00	-0.5811548	2.97815799	0.00502438	0.00906883	-33.694654
ENSG00000165269	AQP7	33383179	33402682	19503	1.62E+00	-0.5021163	2.34482828	0.02435246	0.04272064	-35.09294
ENSG00000165623	UCMA	13221766	13234374	12608	1.99E+00	-0.5148218	3.1026257	0.00360673	0.00653145	-33.346452
ENSG00000167733	HSD11B1L	5680604	5688523	7919	1.49E+00	-0.4702021	2.63897291	0.01198447	0.02136042	-34.399964
ENSG00000167770	OTUB1	63985853	64001811	15958	1.10E+00	-0.514401	3.1628655	0.00306528	0.00555878	-33.20062
ENSG00000167930	FAM234A	234521	272183	37662	4.03E+00	-0.187903	5.31545811	4.92E-06	0.00252381	-26.517547
ENSG00000167969	ECI1	2239402	2252300	12898	1.26E+00	-0.6642644	2.97081083	0.00512265	0.0092434	-33.772188
ENSG00000168291	PDHB	58427630	58433857	6227	2.19E+00	0.13624176	2.95853473	0.00529087	0.00954376	-32.490349
ENSG00000168806	LCMT2	43323649	43330582	6933	2.39E+00	0.03014532	2.99959031	0.00474776	0.00857413	-32.684958
ENSG00000168952	STXBP6	24809656	25050297	240641	3.37E+00	-0.1857218	4.14575087	0.00018243	0.00502254	-30.021089
ENSG00000171603	CLSTN1	9728926	9823984	95058	2.41E+00	0.26670683	3.04500699	0.00420824	0.00760922	-32.130469
ENSG00000171848	RRM2	10120698	10211725	91027	1.54E+00	-0.8541298	2.46667628	0.01825278	0.03226805	-35.130372
ENSG00000171873	ADRA1D	4220630	4249287	28657	1.57E+00	-0.8525651	4.00928492	0.00027419	0.00502254	-31.142356
ENSG00000172023	REG1B	79085023	79088019	2996	1.06E+00	-0.8254211	2.73172216	0.0094984	0.01699685	-34.55174
ENSG00000172236	TPSAB1	1240379	1242554	2175	1.18E+00	-0.6331166	2.55767316	0.01464321	0.02601061	-34.718749
ENSG00000172671	ZFAND4	45615500	45672780	57280	4.74E+00	-0.1971872	9.57137417	1.12E-11	5.03E-08	-13.413511
ENSG00000172732	MUS81	65857126	65867653	10527	1.04E+00	-0.5717328	2.65928616	0.01139352	0.0203327	-34.443509
ENSG00000172803	SNX32	65833834	65856896	23062	2.00E+00	0.16455001	2.45233257	0.01889056	0.03336819	-33.564976
ENSG00000172967	XKR3	16783412	16825411	41999	1.27E+00	-0.6546797	3.01558636	0.00455071	0.00822241	-33.684479
ENSG00000173218	VANGL1	115641970	115698224	56254	1.51E+00	-0.8558785	3.79597282	0.00051387	0.00502254	-31.760913
ENSG00000173253	DMRT2	1049858	1057552	7694	3.27E+00	0.03441284	5.9678947	6.26E-07	0.00040679	-24.053063
ENSG00000173486	FKBP2	64241003	64244132	3129	1.30E+00	-0.780002	4.0735234	0.00022645	0.00502254	-30.826164
ENSG00000173757	STAT5B	42199177	42276707	77530	2.64E+00	-0.6277189	5.10152864	9.63E-06	0.00458603	-27.632673
ENSG00000173805	HAP1	41717742	41734644	16902	3.01E+00	0.20766766	3.38322189	0.00167125	0.00502254	-31.345202
ENSG00000174021	GNG5	84498325	84506581	8256	1.03E+00	-0.7650099	2.53336082	0.01553753	0.02756374	-34.874696
ENSG00000174738	NR1D2	23945286	23980617	35331	2.88E+00	0.18166666	4.02192765	0.00026408	0.00502254	-29.562107
ENSG00000175730	BAK1P1	32690180	32690815	635	1.38E+00	-0.7988974	3.41922295	0.0015111	0.00502254	-32.679855

ENSG00000175984	DENND2C	114582848	114670422	87574	2.49E+00	-0.5964483	3.89424933	0.00038529	0.00502254	-31.280521
ENSG00000176022	B3GALT6	1232237	1235041	2804	1.64E+00	-0.4098136	3.03527439	0.00431875	0.00780774	-33.424391
ENSG00000176261	ZBTB8OS	32600172	32650903	50731	1.06E+00	-0.5454572	2.92639491	0.00575618	0.01037476	-33.801155
ENSG00000177138	FAM9B	9024232	9295682	271450	1.76E+00	-0.550271	2.45287838	0.01886593	0.03332904	-34.89865
ENSG00000177469	CAVIN1	42402449	42423256	20807	3.12E+00	-0.2817688	4.58353463	4.82E-05	0.00502254	-28.801072
ENSG00000177602	HASPIN	3723903	3726699	2796	2.68E+00	-0.2909795	7.33202104	8.70E-09	1.27E-05	-19.48815
ENSG00000178803	ADORA2A-AS1	24429206	24495074	65868	1.22E+00	-0.6466373	3.0367425	0.00430191	0.00777755	-33.591072
ENSG00000179454	KLHL28	44924324	45042322	117998	2.15E+00	0.12896897	2.99042597	0.00486425	0.00878302	-32.429359
ENSG00000180016	OR1E1	3397104	3398410	1306	2.42E+00	-0.1905928	3.26344357	0.00232907	0.00502254	-32.549309
ENSG00000180574	EIF2S3B	10505602	10523135	17533	3.25E+00	-0.6622907	8.32493791	4.24E-10	9.93E-07	-17.548121
ENSG00000181240	SLC25A41	6426037	6433779	7742	3.58E+00	-0.1548474	4.5742016	4.96E-05	0.00502254	-28.783118
ENSG00000181767	OR8H2	56103687	56107658	3971	2.19E+00	-0.5192936	3.60672584	0.00088813	0.00502254	-31.954668
ENSG00000182223	ZAR1	48490252	48494389	4137	1.25E+00	-0.3838989	2.63838632	0.01200195	0.02139086	-34.377762
ENSG00000182224	CYB5D1	7857746	7862282	4536	1.51E+00	-0.7622616	3.79840614	0.00051024	0.00502254	-31.596781
ENSG00000183347	GBP6	89364059	89388160	24101	1.73E+00	-0.495248	4.09158249	0.00021456	0.00502254	-30.568925
ENSG00000183508	TENT5C	117606048	117628389	22341	2.53E+00	0.08346389	3.21439887	0.00266414	0.00502254	-31.982531
ENSG00000183513	COA5	98599314	98608515	9201	3.98E+00	-0.2049147	5.88162802	8.23E-07	0.00049649	-24.724511
ENSG00000183935	HTR7P1	13000420	13004830	4410	1.38E+00	-0.4806339	3.01079311	0.00460894	0.00832593	-33.575412
ENSG00000184608	FAM167A-AS1	11368402	11438658	70256	4.17E+00	-0.5295548	14.8822395	1.89E-17	5.08E-13	0.03783567
ENSG00000185187	SIGIRR	405716	417455	11739	4.13E+00	-0.0593626	11.5912964	4.72E-14	4.24E-10	-7.8174925
ENSG00000185246	PRPF39	45084107	45116282	32175	2.37E+00	-0.1302735	3.44697627	0.00139778	0.00502254	-31.953711
ENSG00000185760	KCNQ5	72621792	73198853	577061	3.57E+00	-0.0246976	8.97055406	6.30E-11	1.89E-07	-14.809243
ENSG00000185904	LINC00839	42475480	42495337	19857	1.07E+00	-0.8831214	3.2571475	0.00236973	0.00502254	-33.292617
ENSG00000186094	AGBL4	48532854	50023954	1491100	1.38E+00	-0.5116855	2.64881294	0.01169477	0.02085579	-34.366544
ENSG00000186458	DEFB132	257724	261096	3372	1.52E+00	-0.3988161	2.46924788	0.01814053	0.03207592	-34.748195
ENSG00000187535	IFT140	1510427	1612072	101645	2.21E+00	0.28336549	2.63511953	0.01209971	0.02156225	-33.14205
ENSG00000187754	SSX7	52644061	52654900	10839	4.76E+00	-0.1867443	8.47721582	2.69E-10	6.91E-07	-16.633763
ENSG00000187833	C2orf78	73784189	73817147	32958	1.08E+00	-0.4265483	2.73129678	0.00950863	0.01701457	-34.168573
ENSG00000188171	ZNF626	20619939	20661596	41657	1.67E+00	-0.3945627	2.60874642	0.01291624	0.02299453	-34.441125
ENSG00000188408	MAGEB5	26216169	26218270	2101	1.24E+00	-0.8493627	3.2569403	0.00237108	0.00502254	-33.214164
ENSG00000188782	CATSPER4	26190561	26202968	12407	2.65E+00	-0.07474	3.65934261	0.00076359	0.00502254	-31.401509
ENSG00000189166	TNRC18P3	56991888	57009149	17261	3.47E+00	-0.2875445	6.42093684	1.50E-07	0.00013241	-23.049782
ENSG00000189196	LINC00994	64078361	64087363	9002	2.62E+00	-0.1615229	3.2188319	0.00263206	0.00502254	-32.638471
ENSG00000196277	GRM7	6770001	7741533	971532	2.28E+00	-0.1462576	2.40525018	0.02112894	0.03722079	-33.910347
ENSG00000197106	SLC6A17	110150494	110202202	51708	1.47E+00	-0.7295936	3.79022919	0.00052255	0.00502254	-31.608398
ENSG00000197185	SSXP1	52606535	52611758	5223	1.19E+00	-0.8336445	3.12306454	0.00341361	0.00618485	-33.534895

ENSG00000197599	CCDC154	1434383	1444556	10173	1.22E+00	-0.8091472	3.31241611	0.00203477	0.00502254	-32.987248
ENSG00000197822	OCLN	69492292	69558104	65812	4.29E+00	-0.2583802	6.43291857	1.44E-07	0.00012963	-22.997253
ENSG00000197905	TEAD4	2959330	3040676	81346	2.46E+00	-0.3019967	3.65459426	0.0007741	0.00502254	-31.604958
ENSG00000198153	ZNF849P	22685167	22686732	1565	1.22E+00	-0.8715698	3.38182366	0.00167778	0.00502254	-32.941338
ENSG00000198555		33976039	33987630	11591	1.31E+00	-0.7429584	2.33699274	0.02480146	0.04348566	-35.28933
ENSG00000199901		52786537	52786638	101	1.18E+00	-0.6837125	2.44263648	0.0193331	0.03412863	-35.00959
ENSG00000200601	RNA5SP50	73749517	73749623	106	2.72E+00	-0.1962058	3.72671326	0.00062854	0.00502254	-31.271101
ENSG00000201482	SNORD115-17	25201323	25201404	81	1.02E+00	-0.7080438	2.83152341	0.00736308	0.01322851	-34.121779
ENSG00000204536	CCHCR1	31142439	31158238	15799	2.74E+00	-0.2570184	4.13757245	0.00018696	0.00502254	-30.114087
ENSG00000205790	DPP9-AS1	4679282	4685948	6666	3.49E+00	-0.0615278	6.16093729	3.40E-07	0.00024459	-23.741584
ENSG00000205899	BHLHA9	1270444	1271815	1371	1.45E+00	-0.4182288	2.42459271	0.02018177	0.03559531	-34.854818
ENSG00000205976		7299774	7306592	6818	1.56E+00	-0.4907586	2.27912583	0.02835477	0.04945843	-35.182728
ENSG00000206559	ZCWPW2	28348721	28538122	189401	3.28E+00	-0.254165	4.86680837	2.00E-05	0.00502254	-27.928004
ENSG00000206560	ANKRD28	15667236	15859771	192535	2.81E+00	0.34649953	3.5087993	0.0011739	0.00502254	-30.880954
ENSG00000206579	XKR4	55102028	55542054	440026	2.19E+00	-0.5434473	4.51377976	5.97E-05	0.00502254	-29.405431
ENSG00000207815	MIR563	15873771	15873849	78	3.76E+00	-0.5407024	9.29660625	2.45E-11	8.81E-08	-14.32959
ENSG00000210140	MT-TC	5761	5826	65	2.69E+00	-0.0984808	4.12887522	0.0001919	0.00502254	-30.084685
ENSG00000212568	RNU6-1254P	66891188	66891291	103	3.50E+00	-0.0629766	4.61063445	4.43E-05	0.00502254	-28.649944
ENSG00000213967	ZNF726	23914876	23945159	30283	1.24E+00	0.19666641	3.05419409	0.00410638	0.00742753	-32.106059
ENSG00000214089	RPL9P21	44414649	44415153	504	1.56E+00	-0.7984072	3.52400939	0.00112434	0.00502254	-32.469121
ENSG00000214765	SEPTIN7P2	45723780	45768985	45205	2.88E+00	-0.3235049	6.09032548	4.25E-07	0.00029396	-24.186229
ENSG00000214860	EVPLL	18377778	18389647	11869	1.16E+00	-0.5601685	3.22896916	0.00256009	0.00502254	-33.039672
ENSG00000214946	TBC1D26	15732247	15749192	16945	1.41E+00	-0.5434225	2.87665772	0.00655247	0.01178984	-33.915931
ENSG00000214999		8079482	8081565	2083	1.20E+00	-0.7547602	2.7017274	0.01024466	0.01831398	-34.469092
ENSG00000215120	SOCS6P1	71527814	71530225	2411	1.64E+00	-0.7693051	3.33534117	0.00190952	0.00502254	-32.960796
ENSG00000215630	GUSBP9	71197646	71208130	10484	3.69E+00	-0.2269607	7.125876	1.65E-08	2.17E-05	-20.49674
ENSG00000215704	CELA2B	15465909	15491395	25486	1.40E+00	1.55373667	2.93578457	0.00561642	0.01012488	-31.699232
ENSG00000215717	TMEM167B	109090764	109096934	6170	2.53E+00	0.08443798	3.46894176	0.00131391	0.00502254	-31.229341
ENSG00000218868	CNN3P1	6534697	6535631	934	1.30E+00	-0.7193836	2.88309905	0.00644382	0.01159746	-34.002259
ENSG00000220154		79278105	79278427	322	1.20E+00	-0.7848816	2.55292094	0.01481422	0.0263083	-34.921466
ENSG00000220161	LINC02076	18411159	18414380	3221	1.55E+00	-0.4502699	3.37668156	0.00170203	0.00502254	-32.546421
ENSG00000223558	TRIM60P17	64085560	64086576	1016	1.10E+00	-0.821991	2.73320581	0.00946283	0.01693544	-34.543844
ENSG00000223601	EBLN1	22208814	22210021	1207	3.29E+00	-0.3689353	4.67850025	3.59E-05	0.00502254	-28.593503
ENSG00000224184	MIR3681HG	11833926	12702913	868987	1.07E+00	-0.6529669	2.50216411	0.01675821	0.0296765	-34.867463
ENSG00000224263	CYCSP12	11878830	11879129	299	1.10E+00	-0.8171828	2.61067867	0.01285473	0.02288957	-34.81005
ENSG00000224812	TMEM72-AS1	44793119	44959709	166590	1.46E+00	-0.5862969	2.70747033	0.0100977	0.01805367	-34.340365

ENSG00000224924	LINC00320	20651450	20803816	152366	3.78E+00	-0.4081903	10.592334	6.60E-13	3.95E-09	-10.771522
ENSG00000224972		6902670	6978859	76189	3.62E+00	-0.0251916	6.27296657	2.39E-07	0.00018665	-23.36361
ENSG00000225036	CDK4P1	105433994	105434821	827	1.31E+00	-0.7901403	3.5043679	0.00118873	0.00502254	-32.43635
ENSG00000226679		38221391	38223667	2276	3.96E+00	-0.3610983	7.88499123	1.60E-09	3.27E-06	-18.495215
ENSG00000227047		20499571	20501311	1740	3.94E+00	-0.1156659	5.95663904	6.49E-07	0.00041651	-24.468939
ENSG00000227685	LINC02088	20612912	20633234	20322	2.84E+00	0.11705914	3.24920534	0.00242197	0.00502254	-31.787305
ENSG00000228079		64086353	64088246	1893	1.36E+00	-0.7478791	3.21139523	0.00268608	0.00502254	-33.202196
ENSG00000228541		62463127	62464070	943	2.13E+00	0.09933591	2.95965115	0.00527536	0.00951673	-32.538984
ENSG00000228716	DHFR	80626226	80654983	28757	1.10E+00	-0.821991	2.73320581	0.00946283	0.01693544	-34.543844
ENSG00000228754	RPL13AP19	48745545	48746128	583	1.82E+00	-0.1247475	2.82270099	0.00753208	0.01353033	-33.551813
ENSG00000228828	TLK2P2	37818606	37820982	2376	2.58E+00	-0.2265959	3.8865239	0.00039414	0.00502254	-30.837087
ENSG00000229167		31571585	31577898	6313	1.38E+00	-0.489814	3.26754257	0.00230296	0.00502254	-32.925638
ENSG00000229180		66526088	66592397	66309	3.16E+00	-0.1850977	4.87824333	1.93E-05	0.00502254	-27.86187
ENSG00000229459		46261064	46294469	33405	1.96E+00	-0.5685325	3.29463347	0.00213728	0.00502254	-32.875972
ENSG00000230014	LINC00709	9275833	9287057	11224	3.23E+00	-0.2032264	4.40784987	8.25E-05	0.00502254	-29.313695
ENSG00000230368	FAM41C	868071	876903	8832	1.37E+00	0.45323234	3.09703627	0.00366132	0.00662919	-31.735883
ENSG00000230435		11406955	11414078	7123	1.25E+00	-0.3996243	2.523921	0.01589803	0.02819029	-34.632919
ENSG00000230501	ANKRD30BP3	45156775	45176925	20150	1.21E+00	-0.4419963	2.75587913	0.00893437	0.01600295	-34.134556
ENSG00000230725		25139652	25149372	9720	3.52E+00	-0.3778305	5.5502784	2.34E-06	0.00127663	-25.836636
ENSG00000230773		47924181	48314224	390043	1.30E+00	-0.7704665	3.58343301	0.00094932	0.00502254	-32.256385
ENSG00000231259	ANAPC1P2	87031815	87052992	21177	3.04E+00	-0.0660771	4.72493819	3.11E-05	0.00502254	-28.314075
ENSG00000232453	LINC02777	58882868	58931897	49029	3.07E+00	-0.3494998	4.67203263	3.67E-05	0.00502254	-28.560598
ENSG00000233723	LINC01122	58427799	59063766	635967	3.66E+00	0.02114573	8.74287849	1.23E-10	3.31E-07	-15.482999
ENSG00000233850		95025193	95026709	1516	3.48E+00	-0.2665566	5.22267379	6.58E-06	0.00328516	-26.835474
ENSG00000233973	LINC01360	73305609	73355253	49644	2.87E+00	0.02380063	3.09745442	0.00365721	0.00662219	-32.174554
ENSG00000234069	GAPDHP53	24445391	24446314	923	1.96E+00	0.26144012	2.50941911	0.01646677	0.0291719	-33.443826
ENSG00000234500		66511556	66545066	33510	1.03E+00	-0.4187004	2.39204022	0.02179875	0.03837189	-34.924318
ENSG00000235217	TSPY26P	32186477	32190527	4050	1.44E+00	-0.7721658	2.99522007	0.00480298	0.00867327	-33.847726
ENSG00000235563		53114576	53118609	4033	1.26E+00	-0.6752362	2.49792379	0.01693072	0.02997806	-34.885887
ENSG00000235636	NUS1P1	47512602	47513483	881	1.02E+00	-0.8031387	2.37142006	0.02288275	0.04022092	-35.349964
ENSG00000235885	LINC01828	67086446	67311439	224993	1.86E+00	-0.0786992	2.79245647	0.00813927	0.01460208	-32.85822
ENSG00000236168		23980585	23980734	149	1.21E+00	-0.7246928	2.82097736	0.00756552	0.01358904	-34.154988
ENSG00000236635	LINC02540	76521640	76593821	72181	2.48E+00	0.0505275	3.30605372	0.0020709	0.00502254	-31.665691
ENSG00000236834	LINC00421	19345049	19346749	1700	1.75E+00	-0.8423153	4.21687054	0.00014729	0.00502254	-30.51543
ENSG00000237402	CAMTA1-IT1	7368942	7370270	1328	1.47E+00	-0.772201	3.86962074	0.00041422	0.00502254	-31.400676
ENSG00000237445		13657311	13659910	2599	1.11E+00	-0.7407241	2.60441893	0.01305496	0.02323919	-34.73609

ENSG00000238158		3831933	3855737	23804	1.51E+00	-0.8558785	3.79597282	0.00051387	0.00502254	-31.760913
ENSG00000239264	TXNDC5	7881517	7910788	29271	2.05E+00	-0.5878327	4.36557055	9.39E-05	0.00502254	-29.840477
ENSG00000239589	LINC00879	94937980	95168351	230371	1.50E+00	-0.5324618	2.68738784	0.01062026	0.01897222	-34.37521
ENSG00000241832	CECR3	17256859	17266733	9874	2.40E+00	0.03287719	4.74023218	2.97E-05	0.00502254	-27.550688
ENSG00000244754	N4BP2L2	32432417	32538885	106468	1.62E+00	0.28781721	2.66505984	0.01123053	0.02004581	-32.957699
ENSG00000246695	RASSF8-AS1	25936683	25959765	23082	3.46E+00	0.00161486	6.65801638	7.11E-08	6.84E-05	-22.026665
ENSG00000247596	TWF2	52228612	52246788	18176	2.60E+00	-0.074455	5.50770988	2.68E-06	0.00144564	-25.211782
ENSG00000248238	LINC02438	19172335	19456994	284659	1.02E+00	-0.8863238	3.18945632	0.00285163	0.0051736	-33.476424
ENSG00000248347		47089572	47198861	109289	1.67E+00	-0.0592291	2.74996615	0.00906948	0.01624118	-33.690077
ENSG00000248508	SRP14-AS1	40039242	40076539	37297	1.04E+00	-0.7589164	2.85047946	0.00701185	0.01260758	-34.165969
ENSG00000248692	ADGRL3-AS1	62071752	62165554	93802	1.06E+00	-0.8254211	2.73172216	0.0094984	0.01699685	-34.55174
ENSG00000249028		1380060	1391502	11442	2.30E+00	-0.2699309	4.1939278	0.00015783	0.00502254	-30.116163
ENSG00000249840	GAPDHP76	11585246	11587135	1889	1.25E+00	-0.6870824	2.48459074	0.01748377	0.03093596	-34.919744
ENSG00000250381	UNC93B4	4143458	4150421	6963	1.05E+00	-0.5209789	2.73348071	0.00945626	0.01692479	-34.259249
ENSG00000250585	LINC00604	40240167	40267657	27490	1.29E+00	-0.86132	3.31825628	0.00200214	0.00502254	-33.085131
ENSG00000250629		38783580	38792754	9174	1.07E+00	-0.7309225	2.28142649	0.02820522	0.04920553	-35.446013
ENSG00000250899		3041437	3044950	3513	3.30E+00	-0.3248953	4.83644983	2.20E-05	0.00502254	-28.042459
ENSG00000251158		69898867	69903198	4331	1.61E+00	-0.5888333	3.24638725	0.00244077	0.00502254	-33.043043
ENSG00000251595	ABCA11P	425435	474129	48694	1.59E+00	-0.5890043	3.12747496	0.00337324	0.00611232	-33.312984
ENSG00000252368	RNA5SP43	37264677	37264786	109	1.57E+00	-0.6608821	2.68925498	0.01057065	0.01888484	-34.448974
ENSG00000253517	XRCC6P4	62855068	62857134	2066	2.76E+00	-0.2631556	4.02523037	0.0002615	0.00502254	-30.442792
ENSG00000253523		58031334	58032682	1348	2.05E+00	-0.4661524	3.65785666	0.00076686	0.00502254	-31.834977
ENSG00000253771	TPTE2P1	24924677	24968487	43810	1.98E+00	-0.7271529	4.37434725	9.14E-05	0.00502254	-29.926174
ENSG00000253837		23336171	23366125	29954	2.31E+00	-0.6118954	4.48128916	6.59E-05	0.00502254	-29.533355
ENSG00000254320		36321039	36321404	365	1.85E+00	0.23518418	2.39658893	0.02156597	0.03796833	-33.678135
ENSG00000254408	OR4A1P	49898267	49899186	919	1.75E+00	-0.7461352	3.76645958	0.00056002	0.00502254	-31.680173
ENSG00000254557		69713390	69719722	6332	4.04E+00	-0.1844835	3.46532553	0.00132738	0.00502254	-31.485743
ENSG00000254560	BBOX1-AS1	27047186	27220113	172927	1.20E+00	-0.681656	2.48434124	0.01749428	0.03095353	-34.95338
ENSG00000254934	LINC00678	27617626	27634627	17001	1.56E+00	-0.8002714	3.88306609	0.00039817	0.00502254	-31.484402
ENSG00000255277	ABCC6P2	14820792	14824702	3910	1.29E+00	-0.7003055	3.37558581	0.00170724	0.00502254	-32.751462
ENSG00000255323	LINC01495	22445673	22492073	46400	1.50E+00	-0.8239012	4.6167036	4.35E-05	0.00502254	-29.296328
ENSG00000255359	CCDC179	22846922	22860474	13552	1.38E+00	-0.7988974	3.41922295	0.0015111	0.00502254	-32.679855
ENSG00000255378	PRR23D2	7778591	7781413	2822	1.32E+00	-0.4220806	3.04959574	0.00415707	0.00751846	-33.289953
ENSG00000255413		13741809	13742781	972	1.68E+00	-0.1984122	2.44589688	0.01918325	0.03387409	-34.49687
ENSG00000255463		9631008	9631359	351	1.07E+00	-0.8831214	3.2571475	0.00236973	0.00502254	-33.292617
ENSG00000255660	RERG-AS1	15151923	15155283	3360	1.05E+00	-0.663399	3.10794063	0.00355554	0.00643984	-33.428017

ENSG00000256226		27105151	27161393	56242	2.67E+00	-0.5585961	5.41295367	3.62E-06	0.00191101	-26.618042
ENSG00000256232	LINC02387	31363481	31369301	5820	1.75E+00	-0.3157442	3.61580234	0.00086534	0.00502254	-31.758237
ENSG00000257522		29210986	29392048	181062	3.90E+00	-0.4756389	7.87608988	1.64E-09	3.27E-06	-18.561358
ENSG00000257534		54162065	54164452	2387	1.46E+00	-0.5878726	2.4882558	0.01733013	0.03067317	-34.821132
ENSG00000258334		49292631	49324576	31945	1.46E+00	-0.3883805	2.34793115	0.02417669	0.04241919	-35.013798
ENSG00000258657		24595723	24657774	62051	2.28E+00	-0.2303802	2.81592616	0.00766431	0.01376418	-33.672552
ENSG00000258747		44507385	44567467	60082	1.05E+00	-0.2787693	2.36435216	0.02326536	0.04087478	-34.869827
ENSG00000259126		44392531	44393716	1185	3.21E+00	-0.775528	6.2386927	2.66E-07	0.00019931	-24.125773
ENSG00000259168		27483035	27541991	58956	1.22E+00	-0.8091472	3.31241611	0.00203477	0.00502254	-32.987248
ENSG00000259380		38139595	38226897	87302	2.97E+00	-0.6815734	5.92772559	7.11E-07	0.00045101	-25.074795
ENSG00000259423		39167676	39180048	12372	1.02E+00	-0.8863238	3.18945632	0.00285163	0.0051736	-33.476424
ENSG00000259974	LINC00261	22547671	22578642	30971	4.28E+00	-0.6059603	15.4033719	6.10E-18	3.29E-13	1.13275534
ENSG00000260058		8309962	8357860	47898	2.33E+00	-0.39414	3.5315586	0.0011005	0.00502254	-32.056986
ENSG00000260406		43302096	43302772	676	1.11E+00	0.38573678	2.52443766	0.0158781	0.02815588	-33.194799
ENSG00000261020		22420022	22424731	4709	1.96E+00	-0.4269265	3.85187473	0.00043636	0.00502254	-31.213715
ENSG00000261189		7540451	7541338	887	1.52E+00	-0.3761233	2.61661429	0.01266749	0.02256137	-34.368843
ENSG00000261397	LINC01177	9441294	9444985	3691	2.71E+00	-0.5206614	4.61080824	4.43E-05	0.00502254	-29.050602
ENSG00000261405		33562649	33570935	8286	2.89E+00	0.18748959	3.27798578	0.0022377	0.00502254	-31.615819
ENSG00000261608	SLC25A1P4	35173316	35174093	777	1.61E+00	-0.4635231	2.4010091	0.02134192	0.03759107	-34.90956

Downregulated DEGs

background	hgnc_symbol	start_position	end_position	size	logFC	AveExpr	t	P.Value	adj.P.Val	B
ENSG00000002834	LASP1	38869859	38921770	51911	-1.6069648	-0.3792961	-2.8382377	7.24E-03	0.01300344	-33.906594
ENSG00000006638	TBXA2R	3594507	3606875	12368	-1.1212392	0.20972002	-3.4797608	1.27E-03	0.00502254	-31.0423
ENSG00000006788	MYH13	10300865	10373130	72265	-2.0621501	-0.5380914	-4.2132121	1.49E-04	0.00502254	-30.320348
ENSG00000007341	ST7L	112523514	112620825	97311	-1.1999352	1.67288505	-2.9376198	5.59E-03	1.01E-02	-31.66882
ENSG00000007516	BAIAP3	1333638	1349441	15803	-1.343169	0.10009046	-3.3516852	1.82E-03	0.00502254	-31.436613
ENSG00000009307	CSDE1	114716913	114758676	41763	-1.2615775	-0.5091305	-2.6913887	1.05E-02	1.88E-02	-34.329836
ENSG00000010318	PHF7	52410660	52423641	12981	-2.416158	-0.177488	-3.6589735	7.64E-04	0.00502254	-31.471007
ENSG00000011275	RNF216	5620047	5781696	161649	-1.3908561	1.17649007	-2.2919829	2.75E-02	4.81E-02	-33.295966
ENSG00000011295	TTC19	15999784	16045015	45231	-2.230486	-0.3434499	-3.2879365	2.18E-03	5.02E-03	-32.265109
ENSG00000011426	ANLN	36389821	36453791	63970	-1.6838075	-0.0100422	-2.762171	8.79E-03	1.58E-02	-32.988849

ENSG00000012232	EXTL3	28600469	28755599	155130	-1.5090308	0.22534787	-3.5190834	1.14E-03	5.02E-03	-30.944038
ENSG00000013016	EHD3	31234152	31269451	35299	-1.1745443	-0.8849472	-3.5434388	1.06E-03	0.00502254	-32.492839
ENSG00000015171	ZMYND11	134465	254637	120172	-1.080125	-0.4248339	-2.4676242	1.82E-02	3.22E-02	-34.763358
ENSG00000021762	OSBPL5	3087107	3166739	79632	-1.8095477	0.06515011	-2.7622985	8.79E-03	1.58E-02	-32.926215
ENSG00000021852	C8B	56929207	56966140	36933	-1.6945115	0.20239486	-2.3678049	2.31E-02	4.06E-02	-33.791952
ENSG00000033122	LRRC7	69567922	70151945	584023	-1.387939	-0.3035252	-3.9964461	0.00028485	0.00502254	-29.901285
ENSG00000033178	UBA6	67612652	67701155	88503	-2.5993754	-0.266243	-5.7555847	1.23E-06	0.00071381	-25.02856
ENSG00000034239	EFCAB1	48710789	48735311	24522	-1.5038099	0.00485168	-2.2859155	2.79E-02	0.04871578	-34.034809
ENSG00000040531	CTNS	3636459	3663103	26644	-1.7073731	-0.0021297	-2.4577698	1.86E-02	3.29E-02	-33.6692
ENSG00000042813	ZPBP	49850421	50121329	270908	-2.95978	-0.3064535	-6.7221714	5.81E-08	5.80E-05	-22.05129
ENSG00000050405	LIMA1	50175788	50283546	107758	-1.0557171	-0.0956296	-3.1206212	3.44E-03	0.00622551	-32.175978
ENSG00000051128	HOMER3	18929201	18941261	12060	-2.3182483	-0.5243679	-4.1998888	1.55E-04	0.00502254	-30.340987
ENSG00000052723	SIKE1	114769479	114780685	11206	-2.9734615	-0.3682415	-4.0427848	2.48E-04	0.00502254	-30.493489
ENSG00000054965	FAM168A	73400487	73598189	197702	-1.0049976	0.37967079	-2.9220856	5.82E-03	0.01049162	-32.368752
ENSG00000058453	CROCC	16740273	16972964	232691	-1.8130442	0.23268474	-2.4526976	0.01887408	0.03334235	-33.57723
ENSG00000061337	LZTS1	20246165	20303963	57798	-1.2279034	0.19181267	-3.7194207	6.42E-04	5.02E-03	-30.335837
ENSG00000062524	LTK	41503637	41513887	10250	-1.1843881	-0.1330196	-2.5664228	1.43E-02	2.55E-02	-33.567514
ENSG00000064989	CALCRL	187341964	187448460	106496	-1.0637892	0.26303205	-3.390135	1.64E-03	5.02E-03	-31.226445
ENSG00000066382	MPPED2	30384493	30586872	202379	-1.4268096	-0.0547254	-2.287906	2.78E-02	4.85E-02	-34.091416
ENSG00000068745	IP6K2	48688003	48740353	52350	-1.3984067	-0.2966418	-3.1249986	3.40E-03	6.15E-03	-33.160238
ENSG00000070444	MNT	2384073	2401104	17031	-1.0821875	0.33008164	-2.7188769	9.81E-03	1.76E-02	-32.892646
ENSG00000070748	CHAT	49609095	49667942	58847	-1.5798807	0.08345654	-2.4213829	2.03E-02	0.03586305	-33.745552
ENSG00000071051	NCK2	105744912	105894274	149362	-3.296572	0.01762208	-5.7540663	1.23E-06	7.14E-04	-24.770523
ENSG00000072954	TMEM38A	16661139	16690023	28884	-2.4283353	-0.2848457	-3.9228394	3.54E-04	0.00502254	-30.936889
ENSG00000075618	FSCN1	5592816	5606655	13839	-1.8973387	-0.1332632	-3.9276641	3.49E-04	5.02E-03	-30.191834
ENSG00000076344	RGS11	268301	275980	7679	-1.3111539	-0.6995634	-3.8725344	4.11E-04	5.02E-03	-31.373432
ENSG00000076662	ICAM3	10333776	10339661	5885	-2.1556147	-0.4800321	-4.1644239	1.72E-04	0.00502254	-30.361838
ENSG00000077092	RARB	25174332	25597932	423600	-1.1129243	0.13363738	-3.5504413	1.04E-03	5.02E-03	-30.840662
ENSG00000077800	FKBP6	73328161	73358637	30476	-1.2630543	-0.3051011	-2.4495089	1.90E-02	0.03359193	-34.701565

ENSG00000078061	ARAF	47561205	47571908	10703	-1.6728043	-0.8047408	-4.4406834	7.47E-05	0.00502254	-29.824533
ENSG00000078674	PCM1	17922840	18027975	105135	-1.5348974	1.67808063	-2.8490277	0.00703819	0.01265409	-31.878357
ENSG00000081665	ZNF506	19785839	19821751	35912	-2.3109622	-0.2109283	-3.6720744	0.00073609	0.00502254	-31.427059
ENSG00000084073	ZMPSTE24	40258041	40294180	36139	-1.0451524	-0.1260395	-3.8087054	4.95E-04	5.02E-03	-30.338228
ENSG00000084674	APOB	21001429	21044073	42644	-3.2935874	-0.4665203	-3.8637809	4.21E-04	0.00502254	-30.64835
ENSG00000087470	DNM1L	32679200	32745650	66450	-1.4784167	0.48159639	-3.4066304	0.00156537	0.00502254	-31.054781
ENSG00000088833	NSFL1C	1442162	1473842	31680	-1.5062507	0.02431034	-3.1748648	2.97E-03	0.0053812	-31.866909
ENSG00000088881	EBF4	2692874	2760108	67234	-1.4473514	-0.3533071	-2.3309549	2.52E-02	0.04404531	-35.042128
ENSG00000089472	HEPH	66162671	66268867	106196	-1.4948888	0.03543826	-2.524472	1.59E-02	0.02815446	-33.679581
ENSG00000090905	TNRC6A	24610209	24827632	217423	-1.5266357	0.3714986	-2.8991082	6.18E-03	1.11E-02	-32.412002
ENSG00000092148	HECTD1	31100112	31207804	107692	-1.3144689	-0.3726152	-2.5120235	1.64E-02	2.90E-02	-34.65922
ENSG00000094631	HDAC6	48801377	48824982	23605	-1.5875536	-0.6765629	-2.8434381	7.14E-03	0.01283495	-34.154826
ENSG00000097033	SH3GLB1	86704570	86748184	43614	-1.7803035	-0.1425344	-3.0055185	4.67E-03	0.00844172	-32.505583
ENSG00000099250	NRP1	33177492	33336262	158770	-2.010925	0.45165057	-2.4478603	1.91E-02	3.37E-02	-33.400529
ENSG00000099785	MARCHF2	8413270	8439017	25747	-1.1871308	-0.5530554	-2.8472221	7.07E-03	1.27E-02	-34.034861
ENSG00000099899	TRMT2A	20111875	20117392	5517	-1.7090582	0.21339716	-2.6100792	1.29E-02	2.29E-02	-33.174918
ENSG00000100483	VCPKMT	50108632	50116600	7968	-1.3905771	-0.6617382	-2.6915931	1.05E-02	1.88E-02	-34.534415
ENSG00000100731	PCNX1	70907405	71115382	207977	-1.0037322	0.20544725	-3.2409359	2.48E-03	0.00502254	-31.645227
ENSG00000100884	CPNE6	24070837	24078100	7263	-1.65694	0.17571211	-2.4435191	1.93E-02	3.41E-02	-33.637944
ENSG00000100911	PSME2	24143362	24147570	4208	-1.1745443	-0.8849472	-3.5434388	0.00106396	0.00502254	-32.492839
ENSG00000101003	GINS1	25407673	25452628	44955	-2.6381597	-0.3652026	-7.5970963	3.85E-09	6.69E-06	-19.123137
ENSG00000101236	RNF24	3927309	4015558	88249	-1.1549672	0.19120329	-3.3175915	2.01E-03	0.00502254	-31.540196
ENSG00000101307	SIRPB1	1561385	1620061	58676	-1.7801373	-0.0494532	-3.3304057	1.94E-03	0.00502254	-31.622576
ENSG00000102003	SYP	49187815	49200199	12384	-2.0862459	-0.6327805	-3.7523115	5.84E-04	5.02E-03	-31.73858
ENSG00000102098	SCML2	18239313	18354688	115375	-1.1582808	-0.590111	-2.9916548	0.00484847	0.00875483	-33.709864
ENSG00000102172	SMS	21940709	21994837	54128	-2.6611137	-0.5721323	-6.7922588	4.67E-08	4.93E-05	-22.345584
ENSG00000103227	LMF1	853634	981318	127684	-3.0565061	-0.2016405	-7.574124	4.13E-09	6.95E-06	-19.412594
ENSG00000103342	GSPT1	11868128	11916082	47954	-1.9893903	0.49404211	-3.224812	2.59E-03	5.02E-03	-31.508384
ENSG00000103512	NOMO1	14833721	14896157	62436	-1.6820124	0.43181227	-3.8122112	4.90E-04	0.00502254	-29.968751

ENSG00000104164	BLOC1S6	45587214	45615945	28731	-1.1918683	-0.8011381	-2.84251	7.16E-03	1.29E-02	-34.215506
ENSG00000104765	BNIP3L	26383054	26505636	122582	-1.9650444	0.27891964	-2.7828262	8.34E-03	0.01496075	-32.799922
ENSG00000105072	C19orf44	16496394	16521352	24958	-1.1494047	-0.0136152	-3.8289115	4.67E-04	5.02E-03	-30.126067
ENSG00000105676	ARMC6	19033575	19060311	26736	-1.8972232	0.09112929	-3.1976834	2.79E-03	0.00505965	-31.801752
ENSG00000105855	ITGB8	20330702	20415754	85052	-2.5018184	0.38880284	-3.1644121	3.05E-03	0.0055357	-31.701532
ENSG00000105953	OGDH	44606572	44709066	102494	-1.1925411	0.21201067	-3.4200884	1.51E-03	0.00502254	-31.152224
ENSG00000106355	LSM5	32485338	32495283	9945	-1.1498526	-0.0892015	-2.8006395	7.97E-03	0.01430574	-32.966616
ENSG00000106635	BCL7B	73536356	73557690	21334	-1.9957041	-0.2983777	-2.7963493	8.06E-03	0.01446031	-33.718962
ENSG00000106638	TBL2	73567537	73578791	11254	-1.0365421	0.1897663	-2.3590959	2.36E-02	0.04136373	-33.806736
ENSG00000106686	SPATA6L	4553386	4666674	113288	-2.1421582	-0.0724678	-5.2431083	6.17E-06	0.00313893	-26.323325
ENSG00000106804	C5	120952335	121050275	97940	-1.2266848	0.33374753	-3.5369007	1.08E-03	5.02E-03	-30.766564
ENSG00000107295	SH3GL2	17579066	17797124	218058	-2.4312009	-0.3622225	-4.7892607	2.55E-05	0.00502254	-28.332723
ENSG00000108091	CCDC6	59788747	59906556	117809	-2.5555068	0.19499742	-2.8608788	0.00682587	0.01227441	-32.496287
ENSG00000108839	ALOX12	6996049	7010754	14705	-1.6895528	-0.7516831	-4.4567545	7.11E-05	0.00502254	-29.76663
ENSG00000109685	NSD2	1871393	1982207	110814	-1.325745	2.39004837	-2.7142881	9.93E-03	0.01774983	-31.998516
ENSG00000110442	COMMD9	36269284	36289449	20165	-1.6202423	-0.3888721	-2.5625407	1.45E-02	0.02571128	-34.550527
ENSG00000110446	SLC15A3	60937060	60952653	15593	-1.8737783	0.2727281	-2.3984673	2.15E-02	0.03781	-33.658396
ENSG00000110514	MADD	47269161	47330031	60870	-1.1250762	-0.2194965	-2.5891672	1.36E-02	0.02411395	-34.257704
ENSG00000110619	CARS1	3000922	3057613	56691	-1.9413373	0.34116995	-3.8785361	4.04E-04	0.00502254	-29.801877
ENSG00000110799	VWF	5948877	6124770	175893	-2.4699855	0.47404261	-3.2312566	2.54E-03	0.00502254	-31.494236
ENSG00000111057	KRT18	52948871	52952906	4035	-1.0252948	-0.8516731	-2.5062528	1.66E-02	0.02939235	-35.056001
ENSG00000111262	KCNA1	4909905	4918256	8351	-1.7115288	0.16561172	-2.4688259	1.82E-02	0.03210525	-33.589652
ENSG00000111371	SLC38A1	46183063	46270017	86954	-2.0154199	-0.1423312	-4.5203993	5.85E-05	5.02E-03	-28.507222
ENSG00000112130	RNF8	37353979	37394734	40755	-1.0945937	-0.0985103	-3.6772788	7.25E-04	0.00502254	-30.663764
ENSG00000112144	CILK1	53001279	53061824	60545	-1.0481721	-0.6552655	-2.3595636	2.35E-02	0.04132115	-35.280312
ENSG00000112183	RBM24	17281361	17293871	12510	-2.0568083	0.2316612	-3.8279333	4.68E-04	5.02E-03	-29.972224
ENSG00000112337	SLC17A2	25912754	25930691	17937	-1.8038525	0.20920452	-2.3639502	2.33E-02	0.04091065	-33.753545
ENSG00000112773	TENT5A	81491439	81752774	261335	-1.2061213	-0.6882205	-2.4482813	1.91E-02	0.03368727	-35.040762
ENSG00000112877	CEP72	612340	667168	54828	-1.9706531	0.19575974	-3.2428057	0.00246487	0.00502254	-31.593769

ENSG00000113389	NPR3	32689070	32791724	102654	-1.5101655	0.00356563	-3.7970645	0.00051224	0.00502254	-30.257196
ENSG00000113492	AGXT2	34998101	35048135	50034	-1.1665316	-0.5923368	-2.3195476	0.02582801	0.04516215	-35.273726
ENSG00000115274	INO80B	74455087	74457944	2857	-2.4193416	-0.3531042	-5.0382029	1.17E-05	0.00502254	-27.667632
ENSG00000115286	NDUFS7	1383527	1395589	12062	-2.0800739	-0.3838519	-3.8173525	0.00048277	0.00502254	-31.276701
ENSG00000115386	REG1A	79120362	79123409	3047	-1.7029807	-0.7967334	-4.5206698	5.84E-05	0.00502254	-29.578575
ENSG00000115446	UNC50	98608579	98618515	9936	-1.6555006	-0.5130177	-4.8975926	1.82E-05	0.00502254	-27.20839
ENSG00000115561	CHMP3	86503430	86563479	60049	-1.181942	0.06719886	-3.4575154	0.00135692	0.00502254	-31.132831
ENSG00000115934		23181334	23251499	70165	-2.8616223	-0.523674	-5.5797219	2.14E-06	0.00118728	-25.869998
ENSG00000116171	SCP2	52927276	53051698	124422	-1.510576	-0.6618128	-2.8130934	0.00772023	0.01386185	-34.18137
ENSG00000116698	SMG7	183472216	183598246	126030	-1.1379389	0.39717982	-3.1174547	0.00346562	0.00627845	-31.872607
ENSG00000117118	SDHB	17018722	17054032	35310	-1.8783004	-0.4276198	-3.3286207	0.00194545	0.00502254	-32.726183
ENSG00000117600	PLPPR4	99264292	99309590	45298	-1.1603411	-0.6020953	-2.673848	0.01098657	0.01961801	-34.488198
ENSG00000117862	TXNDC12	52020131	52055191	35060	-1.4304867	-0.1531146	-4.3933588	8.63E-05	0.00502254	-28.732444
ENSG00000118007	STAG1	136336236	136752403	416167	-1.1098695	0.30743258	-3.3224354	0.0019791	0.00502254	-31.386802
ENSG00000118894	EEF2KMT	5084284	5097795	13511	-1.6251458	-0.6620473	-2.8212774	0.00755969	0.01357902	-34.165836
ENSG00000120697	ALG5	36949738	37000763	51025	-1.3074013	0.43866402	-3.2191948	0.00262945	0.00502254	-31.564412
ENSG00000120903	CHRNA2	27459756	27479883	20127	-2.1069546	-0.2802425	-4.6882134	3.49E-05	0.00502254	-28.60677
ENSG00000121905	HPCA	32885994	32898441	12447	-1.0374821	-0.8679836	-3.2698646	0.0022883	0.00502254	-33.211698
ENSG00000122547	EEPD1	36153254	36301538	148284	-1.9556586	0.3206969	-3.3816285	0.0016787	0.00502254	-31.232688
ENSG00000122584	NXPH1	8433609	8752961	319352	-2.2800279	0.28000323	-2.8634931	0.00677985	0.01219368	-32.515572
ENSG00000123243	ITIH5	7559270	7666998	107728	-1.063901	0.7053747	-2.5270912	0.01577612	0.02797872	-33.01056
ENSG00000123268	ATF1	50763710	50821162	57452	-1.0496272	0.15743366	-2.9282529	0.00572827	0.0103248	-32.486409
ENSG00000123329	ARHGAP9	57472264	57488814	16550	-2.4771951	-0.4384425	-4.8778241	1.94E-05	0.00502254	-28.228267
ENSG00000124356	STAMBP	73828916	73873659	44743	-1.4139264	0.02102812	-2.5618459	0.01449455	0.02575248	-33.407404
ENSG00000124486	USP9X	41085445	41236579	151134	-1.2233414	-0.4985556	-2.6404435	0.01194076	0.02128462	-34.50083
ENSG00000124615	MOCS1	39899578	39934551	34973	-1.8254093	0.12604169	-3.3061126	0.00207056	0.00502254	-31.562637
ENSG00000124688	MAD2L1BP	43629540	43640941	11401	-1.8875812	-0.1550624	-3.801524	0.00050562	0.00502254	-30.374172
ENSG00000124743	KLHL31	53647916	53665756	17840	-2.5876384	-0.1519912	-6.0849674	4.33E-07	0.0002952	-23.900785
ENSG00000124875	CXCL6	73836640	73849064	12424	-2.4767372	-0.6079821	-4.3247801	0.00010627	0.00502254	-30.051083

ENSG00000125630	POLR1B	112541915	112579818	37903	-3.6440126	-0.3789349	-6.2035276	2.98E-07	0.0002167	-23.929041
ENSG00000125648	SLC25A23	6436079	6465203	29124	-1.718818	-0.0539296	-2.6596673	0.0113827	0.02031405	-33.157512
ENSG00000125686	MED1	39404285	39451272	46987	-1.3528588	0.41285137	-3.5469231	0.00105346	0.00502254	-30.722527
ENSG00000125730	C3	6677704	6730562	52858	-1.0808077	0.04346431	-3.2866788	0.00218472	0.00502254	-31.599564
ENSG00000125775	SDCBP2	1309909	1329139	19230	-1.9935736	0.09285859	-2.8744122	0.00659074	0.01185752	-32.687714
ENSG00000125827	TMX4	7977346	8019805	42459	-1.1184381	-0.1476139	-3.1459633	0.00320888	0.00581763	-32.405948
ENSG00000125864	BFSP1	17493905	17569220	75315	-2.2399165	-0.24098	-3.6880506	0.00070292	0.00502254	-31.427617
ENSG00000125903	DEFB129	227258	229886	2628	-1.3510088	-0.8677303	-3.6045129	0.00089378	0.00502254	-32.311049
ENSG00000126759	CFP	47623172	47630305	7133	-1.4509165	0.21081952	-4.082527	0.00022044	0.00502254	-29.305445
ENSG00000128271	ADORA2A	24417879	24442357	24478	-1.3659771	-0.3608439	-2.6844249	0.01069944	0.01911114	-34.265048
ENSG00000128482	RNF112	19411125	19417276	6151	-1.6526726	0.3289579	-3.4342206	0.00144881	0.00502254	-31.102952
ENSG00000129467	ADCY4	24318349	24335093	16744	-2.1473009	-0.0197037	-3.1512798	0.00316304	0.0057351	-31.936046
ENSG00000129514	FOXA1	37589552	37596059	6507	-3.0988977	-0.1949487	-6.2885018	2.28E-07	0.00018034	-23.430232
ENSG00000129518	EAPP	34515938	34539711	23773	-1.7218773	0.33791044	-3.3290804	0.00194298	0.00502254	-31.33519
ENSG00000130032	PRRG3	151694607	151705924	11317	-1.0811439	0.30591766	-3.2381539	0.0024965	0.00502254	-31.617137
ENSG00000130167	TSPAN16	11296139	11326996	30857	-1.5294905	-0.0330572	-3.0806816	0.00382555	0.00692445	-32.229565
ENSG00000130270	ATP8B3	1782075	1812276	30201	-1.3006813	-0.5866975	-3.0476713	0.00417846	0.00755613	-33.556953
ENSG00000130475	FCHO1	17747718	17788568	40850	-2.4592419	-0.0347119	-4.6087881	4.46E-05	0.00502254	-28.228546
ENSG00000130997	POLN	2071918	2242121	170203	-1.9263378	0.23072092	-2.6357837	0.01207977	0.02152743	-33.149912
ENSG00000131013	PPIL4	149504495	149546043	41548	-1.2193801	0.42259068	-3.2404823	0.00248062	0.00502254	-31.546222
ENSG00000131634	TMEM204	1528688	1555580	26892	-2.5352567	-0.7203255	-7.0619271	2.01E-08	2.52E-05	-21.520831
ENSG00000131697	NPHP4	5862811	5992473	129662	-1.3263749	-0.4122378	-2.5634483	0.01443783	0.02565509	-34.547604
ENSG00000132153	DHX30	47802909	47850195	47286	-1.3369903	0.00725358	-3.9601001	0.00031724	0.00502254	-29.795408
ENSG00000132155	RAF1	12583601	12664125	80524	-1.0714768	0.32742079	-3.2277207	0.00256885	0.00502254	-31.647356
ENSG00000132773	TOE1	45340052	45343973	3921	-2.3940078	-0.2638322	-5.1969959	7.14E-06	0.00352848	-27.04205
ENSG00000133107	TRPC4	37632063	37869802	237739	-1.802204	0.04938048	-3.5505658	0.0010426	0.00502254	-31.243105
ENSG00000133246	PRAM1	8490056	8502640	12584	-1.8938677	0.0039213	-3.3509379	0.00182855	0.00502254	-31.690972
ENSG00000134056	MRPS36	69217760	69230158	12398	-1.1593312	-0.378757	-2.3875974	0.0220283	0.03875866	-34.932371
ENSG00000134138	MEIS2	36889204	37101299	212095	-2.1563123	-0.2318829	-3.1870271	0.00287055	0.00520775	-32.133402

ENSG00000134193	REG4	119794017	119811580	17563	-1.3283517	-0.6163866	-2.6702524	0.01108579	0.01979203	-34.537003
ENSG00000134249	ADAM30	119893533	119896515	2982	-1.7815171	-0.5370444	-2.9722522	0.00510323	0.00920929	-33.725485
ENSG00000134532	SOX5	23529504	24562544	1033040	-2.1452181	-0.4987697	-4.3230384	0.00010683	0.00502254	-29.79067
ENSG00000134760	DSG1	31318160	31359246	41086	-1.9949229	0.05814426	-3.1182987	0.00345775	0.00626439	-32.167222
ENSG00000134779	TPGS2	36777647	36829216	51569	-2.8183711	-0.5509984	-6.0325122	5.11E-07	0.00033568	-24.489033
ENSG00000134809	TIMM10	57528464	57530803	2339	-1.9609678	0.2712827	-2.7887182	0.0082174	0.01474029	-32.773468
ENSG00000135298	ADGRB3	68635282	69390571	755289	-2.1111077	0.21525368	-3.1345279	0.00330962	0.00599886	-31.925976
ENSG00000135605	TEC	48135783	48269838	134055	-2.0472119	-0.0456321	-3.5714384	0.00098239	0.00502254	-31.134293
ENSG00000135951	TSGA10	98997261	99154964	157703	-1.2266056	-0.1198226	-4.0069947	0.00027606	0.00502254	-29.718341
ENSG00000136143	SUCLA2	47745736	48037968	292232	-1.1119842	-0.2486798	-2.7753735	0.00850205	0.01524378	-33.677076
ENSG00000136213	CHST12	2403588	2448484	44896	-1.1683347	-0.7139777	-2.5553731	0.01472575	0.02615378	-34.899297
ENSG00000136247	ZDHHC4	6577434	6589374	11940	-1.9526182	-0.3684299	-2.5945019	0.01337804	0.02380409	-33.792804
ENSG00000136250	AOAH	36512941	36724549	211608	-1.7073584	0.11320203	-2.6349985	0.01210335	0.02156802	-33.278416
ENSG00000136267	DGKB	14145049	14974777	829728	-2.035999	-0.4501742	-3.828392	0.00046743	0.00502254	-31.321923
ENSG00000136327	NKX2-8	36580004	36582614	2610	-1.6728043	-0.8047408	-4.4406834	7.47E-05	0.00502254	-29.824533
ENSG00000136695	IL36RN	113058638	113065382	6744	-1.3828778	-0.7293134	-3.8879786	0.00039246	0.00502254	-31.341044
ENSG00000137221	TJAP1	43477523	43506556	29033	-1.5376531	-0.0073284	-3.2785185	0.00223442	0.00502254	-31.742915
ENSG00000137962	ARHGAP29	94148988	94275068	126080	-1.2246042	-0.3253262	-2.980057	0.00499928	0.00902381	-32.649825
ENSG00000137968	SLC44A5	75202131	75611116	408985	-2.4268839	-0.0421013	-4.6949021	3.42E-05	0.00502254	-28.147031
ENSG00000140265	ZSCAN29	43358172	43371043	12871	-1.2032103	-0.3909475	-2.561999	0.01448912	0.02574454	-34.546805
ENSG00000141127	PRPSAP2	18840085	18931287	91202	-1.6631478	-0.0129477	-3.3614781	0.0017757	0.00502254	-31.545258
ENSG00000141873	SLC39A3	2732204	2740028	7824	-1.9273537	0.51376714	-4.3859688	8.82E-05	0.00502254	-28.237159
ENSG00000141934	PLPP2	281040	291403	10363	-1.0979509	0.31313644	-2.8805126	0.00648724	0.01167483	-32.484759
ENSG00000143093	STRIP1	110031577	110074641	43064	-1.8301973	0.16192312	-2.5860574	0.01365893	0.02429266	-33.282531
ENSG00000143126	CELSR2	109249539	109275751	26212	-1.5295148	-0.7583008	-3.0170778	0.00453274	0.00819048	-33.81598
ENSG00000143924	EML4	42169353	42332548	163195	-1.7191367	0.06927001	-2.4309098	0.01988088	0.03507609	-33.758628
ENSG00000143942	CHAC2	53767804	53775196	7392	-1.6257607	-0.0579804	-2.7966743	0.00805194	0.01444878	-32.921154
ENSG00000144036	EXOC6B	72175984	72826041	650057	-1.3730707	2.75794116	-2.7923363	0.00814177	0.01460608	-31.714612
ENSG00000144061	NPHP1	110122311	110205066	82755	-1.0279789	0.2226339	-2.9816011	0.00497895	0.00898772	-32.310042

ENSG00000144161	ZC3H8	112211529	112255136	43607	-1.7634093	0.12942335	-2.3037871	0.0267881	0.04679994	-33.89775
ENSG00000144746	ARL6IP5	69084937	69106092	21155	-1.7624734	-0.6966512	-2.8373421	0.00725356	0.01303261	-34.225098
ENSG00000144868	TMEM108	133038391	133397775	359384	-1.2432914	0.37022353	-3.5440427	0.00106213	0.00502254	-30.745309
ENSG00000144959	NCEH1	172630249	172711218	80969	-1.2007208	0.47909162	-3.1398389	0.00326247	0.00591379	-31.748732
ENSG00000144962	SPATA16	172889357	173141235	251878	-1.0272509	0.22773969	-3.1758378	0.00295923	0.00536736	-31.827356
ENSG00000145681	HAPLN1	83637805	83720855	83050	-3.4344656	-0.1709194	-5.5544622	2.31E-06	0.00127274	-25.727014
ENSG00000146166	LGSN	63275951	63319983	44032	-1.0153005	-0.5081796	-2.431481	0.01985387	0.03502959	-34.918175
ENSG00000146592	CREB5	28299321	28825894	526573	-1.0358566	0.33203252	-3.1291903	0.00335766	0.0060843	-31.904107
ENSG00000146938	NLGN4X	5840637	6228867	388230	-2.7678822	-0.358887	-4.6365272	4.09E-05	0.00502254	-28.7933
ENSG00000146950	SHROOM2	9786429	9949443	163014	-1.2294517	-0.3828812	-2.5884427	0.01357905	0.02415616	-34.485463
ENSG00000147113	DIPK2B	45148373	45200901	52528	-1.6781528	0.01304023	-3.9944982	0.0002865	0.00502254	-29.923094
ENSG00000147488	ST18	52110839	52460959	350120	-1.6143529	-0.8392243	-3.8828438	0.00039843	0.00502254	-31.517819
ENSG00000148925	BTBD10	13388008	13463297	75289	-1.4012311	0.25521665	-3.9507104	0.00032617	0.00502254	-29.609604
ENSG00000149516	MS4A3	60056587	60071115	14528	-1.5963086	0.04801434	-2.6147266	0.01272676	0.0226647	-33.419903
ENSG00000150054	MPP7	28050993	28334486	283493	-1.4151169	0.44183292	-3.971475	0.00030673	0.00502254	-29.518022
ENSG00000150175	FRMPD2B	46870858	46894562	23704	-2.8248092	-0.6136581	-5.6299987	1.82E-06	0.00102348	-26.019892
ENSG00000150477	KIAA1328	36829106	37232172	403066	-1.7138871	0.16825259	-2.5422297	0.01520565	0.02698921	-33.461798
ENSG00000151033	LYZL2	30611779	30629762	17983	-3.4763526	-0.5121724	-7.4138972	6.76E-09	1.04E-05	-20.090782
ENSG00000151327	FAM177A1	35044907	35113130	68223	-1.3219832	-0.5147465	-3.5745157	0.0009738	0.00502254	-32.168565
ENSG00000151414	NEK7	198156994	198322420	165426	-1.0869505	0.36565252	-3.0113385	0.00460228	0.00831474	-32.170964
ENSG00000151490	PTPRO	15322257	15602175	279918	-1.4629621	-0.0004742	-4.2088329	0.0001509	0.00502254	-28.991252
ENSG00000151692	RNF144A	6917412	7068286	150874	-1.4480884	-0.7410689	-3.1784891	0.00293798	0.00532936	-33.380842
ENSG00000151746	BICD1	32106835	32383633	276798	-1.4653996	1.1815184	-2.8475903	0.00706435	0.01270029	-32.04377
ENSG00000152284	TCF7L1	85133392	85310387	176995	-1.1613255	0.25863823	-3.3745181	0.00171233	0.00502254	-31.319456
ENSG00000153012	LGI2	24998847	25030946	32099	-1.2240067	-0.5198189	-3.0554278	0.00409287	0.00740335	-33.47813
ENSG00000153201	RANBP2	108719482	108785809	66327	-2.14779	-0.7867344	-4.5339754	5.61E-05	0.00502254	-29.543778
ENSG00000153303	FRMD1	168053166	168101511	48345	-1.3185511	0.47409496	-3.1782735	0.0029397	0.00533231	-31.643188
ENSG00000153404	PLEKHG4B	92151	189972	97821	-1.2911274	-0.0348896	-3.4322547	0.00145684	0.00502254	-31.416327
ENSG00000154025	SLC5A10	18950345	19022595	72250	-1.2698491	-0.5569795	-3.3007022	0.00210176	0.00502254	-32.887519

ENSG00000154198	CYP4Z2P	46843095	46900437	57342	-2.3313262	-0.1766037	-4.5961232	4.63E-05	0.00502254	-28.66956
ENSG00000154451	GBP5	89258950	89272804	13854	-1.1265284	-0.300443	-2.5659707	0.01434896	0.02550305	-34.519036
ENSG00000154743	TSEN2	12484421	12541549	57128	-2.3694962	-0.1964427	-6.263905	2.46E-07	0.00018795	-23.181706
ENSG00000154814	OXNAD1	16265160	16350299	85139	-1.4880912	-0.3504638	-2.9457639	0.00547137	0.00986604	-33.623662
ENSG00000155026	RSPH10B	5925550	5970683	45133	-1.4021878	0.35357619	-3.7793548	0.00053938	0.00502254	-30.092327
ENSG00000156958	GALK2	49155656	49367869	212213	-1.4906696	1.22506435	-3.1331533	0.00332193	0.00602036	-31.325076
ENSG00000156973	PDE6D	231732433	231786272	53839	-1.1457878	0.44206422	-3.0131544	0.00458017	0.00827507	-32.092915
ENSG00000157330	C1orf158	12746200	12763699	17499	-4.1489917	-0.088402	-6.7475277	5.37E-08	5.46E-05	-21.641876
ENSG00000157379	DHRS1	24290598	24299780	9182	-2.2053091	0.28330657	-2.8060169	0.0078616	0.01411099	-32.671654
ENSG00000157613	CREB3L1	46277662	46321409	43747	-1.1316784	0.13709356	-3.3932235	0.0016252	0.00502254	-31.273416
ENSG00000157856	DRC1	26401920	26456711	54791	-2.3323496	-0.6214505	-4.3633265	9.45E-05	0.00502254	-29.938335
ENSG00000157933	SKI	2228319	2310213	81894	-2.4701182	-0.50407	-6.0449174	4.91E-07	0.00032675	-24.632783
ENSG00000158014	SLC30A2	26037252	26046118	8866	-1.6777221	-0.3715018	-2.6980168	0.01034065	0.0184813	-34.235515
ENSG00000158089	GALNT14	30910467	31155202	244735	-1.0542458	0.038803	-2.3989914	0.02144393	0.03776704	-33.869142
ENSG00000158125	XDH	31334321	31414742	80421	-1.9343858	0.29104216	-2.5655351	0.01436427	0.02552858	-33.293316
ENSG00000158315	RHBDL2	38885807	38941830	56023	-1.1942373	-0.2139052	-3.6316498	0.00082688	0.00502254	-30.850844
ENSG00000158406	H4C8	26277609	26285638	8029	-1.018271	0.52046387	-2.5330988	0.01554743	0.0275804	-33.133379
ENSG00000158825	CDA	20589086	20618903	29817	-2.2742357	-0.5963951	-4.9358077	1.62E-05	0.00502254	-28.188592
ENSG00000158828	PINK1	20633458	20651511	18053	-1.0251433	-0.804887	-2.5418192	0.01522087	0.02701444	-34.977835
ENSG00000159063	ALG8	78095244	78139660	44416	-1.2487434	0.4101617	-3.0673919	0.0039641	0.0071738	-31.979697
ENSG00000159692	CTBP1	1211445	1249953	38508	-1.9603048	0.62549046	-2.5117479	0.0163742	0.02901172	-33.123036
ENSG00000160058	BSDC1	32364633	32394731	30098	-1.2273039	-0.0270277	-4.1070891	0.00020483	0.00502254	-29.400665
ENSG00000160551	TAOK1	29390363	29551903	161540	-2.0088312	-0.0733269	-3.4551271	0.00136608	0.00502254	-31.550342
ENSG00000160785	SLC25A44	156193932	156212796	18864	-1.1749244	0.39617223	-3.2739634	0.00226262	0.00502254	-31.477803
ENSG00000161791	FMNL3	49636499	49708165	71666	-1.0654106	-0.6000952	-2.5166407	0.01618127	0.02867931	-34.851196
ENSG00000162368	CMPK1	47333790	47378839	45049	-1.2360923	-0.8791968	-3.57608	0.00096946	0.00502254	-32.397636
ENSG00000162402	USP24	55066359	55215364	149005	-2.1963175	-0.2713524	-3.9552403	0.00032183	0.00502254	-30.798304
ENSG00000162409	PRKAA2	56645314	56715335	70021	-1.0900146	-0.6530143	-2.5799912	0.01386404	0.02465419	-34.713903
ENSG00000162438	CTRC	15438442	15449242	10800	-1.0650883	0.18853787	-3.2949699	0.0021353	0.00502254	-31.531251

ENSG00000162520	SYNC	32679906	32703596	23690	-1.3124269	-0.4512049	-3.1616685	0.00307525	0.00557667	-33.168294
ENSG00000162521	RBBP4	32651142	32686211	35069	-1.5979781	0.7277129	-3.5195002	0.00113882	0.00502254	-30.527807
ENSG00000162614	NEXN	77888513	77943895	55382	-1.6319459	0.05723356	-2.5129033	0.01632845	0.02893543	-33.661741
ENSG00000162650	ATXN7L2	109483479	109492804	9325	-1.6897848	-0.7722206	-4.4741673	6.74E-05	0.00502254	-29.71561
ENSG00000162777	DENND2D	111185969	111204535	18566	-1.2530119	-0.0007457	-2.3215581	0.02570778	0.04498111	-34.020027
ENSG00000163029	SMC6	17663812	17800242	136430	-2.39399	0.53691632	-2.6650828	0.01122989	0.02004532	-32.875407
ENSG00000163297	ANTXR2	79901146	80125454	224308	-2.5177964	-0.4668187	-3.0102884	0.00461511	0.0083368	-33.168911
ENSG00000163508	EOMES	27715949	27722711	6762	-1.8117467	-0.8065536	-4.6390794	4.06E-05	0.00502254	-29.226331
ENSG00000164045	CDC25A	48157146	48188417	31271	-1.3471459	-0.0703213	-3.3727528	0.00172078	0.00502254	-31.604253
ENSG00000164080	RAD54L2	51538719	51668667	129948	-1.2467325	3.4470043	-2.3029473	0.02684014	0.04688022	-32.643968
ENSG00000164082	GRM2	51707068	51718613	11545	-1.7964977	0.07049924	-2.5642237	0.01441045	0.02560982	-33.372657
ENSG00000164182	NDUFAF2	60945177	61154531	209354	-1.2620979	-0.3545288	-2.8139398	0.00770348	0.01383362	-33.956683
ENSG00000164309	CMYA5	79689836	79800240	110404	-2.5573818	-0.6524216	-7.0085729	2.37E-08	2.78E-05	-21.666968
ENSG00000164619	BMPER	33904308	34156427	252119	-2.3940922	-0.0817975	-4.2771967	0.00012275	0.00502254	-29.588004
ENSG00000164663	USP49	41789896	41895375	105479	-2.5497572	-0.4432776	-6.1320913	3.73E-07	0.00026096	-24.125348
ENSG00000164808	SPIDR	47260878	47736306	475428	-2.2282128	-0.5991044	-4.5839648	4.81E-05	0.00502254	-29.266828
ENSG00000165097	KDM1B	18155329	18223854	68525	-1.2340193	-0.1514127	-3.3684866	0.00174137	0.00502254	-31.549114
ENSG00000165186	PTCHD1	23334849	23404374	69525	-1.5435586	0.02366423	-3.1373836	0.00328419	0.00595296	-32.047781
ENSG00000165355	FBXO33	39397669	39432500	34831	-1.19498	-0.6861542	-2.5364552	0.01542099	0.02736151	-34.913591
ENSG00000165525	NEMF	49782083	49852821	70738	-1.9254255	0.14820227	-2.6409318	0.01192627	0.02126021	-33.110269
ENSG00000165527	ARF6	49893082	49897054	3972	-2.6508025	-0.6059744	-4.0243902	0.00026215	0.00502254	-30.936527
ENSG00000165973	NELL1	20669551	21575686	906135	-1.5244954	-0.7240112	-3.5460709	0.00105602	0.00502254	-32.386809
ENSG00000166016	ABTB2	34150987	34358010	207023	-1.2164324	-0.6461699	-3.4383811	0.00143198	0.00502254	-32.602151
ENSG00000166200	COPS2	49106068	49155661	49593	-1.8277615	0.03532594	-3.0972544	0.00365917	0.00662553	-32.138746
ENSG00000166478	ZNF143	9460319	9528524	68205	-1.0196044	0.36692052	-2.7372566	0.00936634	0.01676609	-32.807371
ENSG00000166535	A2ML1	8822621	8887001	64380	-1.3183574	-0.4544065	-2.9893312	0.00487834	0.00880817	-33.554045
ENSG00000166960	CCDC178	32937402	33441101	503699	-1.0820514	0.17115838	-2.8839147	0.00643019	0.01157331	-32.63658
ENSG00000166986	MARS1	57475445	57517569	42124	-1.6363634	-0.0142614	-2.6915375	0.01051028	0.0187801	-33.17873
ENSG00000167460	TPM4	16067021	16103002	35981	-1.3845579	-0.6974491	-3.8262873	0.00047032	0.00502254	-31.504391

ENSG00000167699	GLOD4	757097	783390	26293	-1.7346719	0.24919787	-2.4995255	0.01686537	0.02986333	-33.406835
ENSG00000167771	RCOR2	63911230	63917164	5934	-1.5606923	-0.1534961	-3.9585077	0.00031874	0.00502254	-30.013892
ENSG00000167775	CD320	8302127	8308358	6231	-1.2665155	-0.1942515	-4.4222507	7.90E-05	0.00502254	-28.543262
ENSG00000167964	RAB26	2140803	2154165	13362	-1.4422169	-0.4023854	-2.5078406	0.01652978	0.02928257	-34.669211
ENSG00000167965	MLST8	2204248	2209453	5205	-1.438806	-0.6742665	-3.6312629	0.00082779	0.00502254	-32.052431
ENSG00000167978	SRRM2	2752626	2772538	19912	-1.2676204	-0.856895	-3.5133426	0.00115888	0.00502254	-32.55539
ENSG00000167995	BEST1	61950063	61965515	15452	-2.9997995	-0.7268676	-6.3507905	1.87E-07	0.00015501	-23.759759
ENSG00000168038	ULK4	41246599	41962130	715531	-1.4489201	0.3470748	-4.1879638	0.00016069	0.00502254	-28.914844
ENSG00000168122	ZNF355P	13095305	13113790	18485	-1.3250347	-0.0025014	-3.7361539	0.00061156	0.00502254	-30.39006
ENSG00000169122	FAM110B	57994509	58204279	209770	-1.1531052	-0.7737589	-2.7800872	0.00840047	0.01506467	-34.367988
ENSG00000170043	TRAPPC1	7930345	7932123	1778	-1.0438389	-0.7821572	-2.7572847	0.00890253	0.01594698	-34.446011
ENSG00000170425	ADORA2B	15945130	15975746	30616	-3.5020391	-0.4044251	-8.0318628	1.02E-09	2.21E-06	-17.93546
ENSG00000170581	STAT2	56341597	56360167	18570	-1.1838981	-0.4754512	-2.676748	0.01090714	0.0194789	-34.343921
ENSG00000170667	RASA4B	102479976	102517777	37801	-1.0669504	0.2014686	-3.2496798	0.00241882	0.00502254	-31.599238
ENSG00000170745	KCNS3	17877847	18361616	483769	-1.5103179	-0.8506908	-3.8560577	0.00043104	0.00502254	-31.598079
ENSG00000170965	PLAC1	134565838	134764322	198484	-1.2988693	0.39371825	-3.5642827	0.00100264	0.00502254	-30.669738
ENSG00000171148	TADA3	9779967	9793011	13044	-2.321771	-0.2795406	-6.4635003	1.31E-07	0.00011972	-22.610155
ENSG00000171467	ZNF318	43307134	43369647	62513	-1.1843721	-0.039644	-3.1838416	0.00289554	0.0052529	-32.136234
ENSG00000171469	ZNF561	9604680	9621236	16556	-2.4547251	-0.2885841	-3.6009309	0.00090299	0.00502254	-31.693934
ENSG00000171517	LPAR3	84811602	84893206	81604	-1.6915145	-0.3265594	-3.41818	0.00151553	0.00502254	-32.377298
ENSG00000171877	FRMD5	43870761	44195271	324510	-1.2242219	0.92689655	-2.6460377	0.01177581	0.02099616	-32.619527
ENSG00000171885	AQP4	26852043	26865771	13728	-1.7792127	-0.5780292	-3.8292457	0.00046627	0.00502254	-31.453188
ENSG00000171928	TVP23B	18781111	18806714	25603	-2.2779805	-0.1697951	-5.0067485	1.30E-05	0.00502254	-26.922534
ENSG00000172005	MAL	95025677	95053992	28315	-1.2212043	-0.2133101	-2.3488654	0.02412399	0.04233086	-34.304931
ENSG00000172171	TEFM	30897336	30906238	8902	-1.5946665	0.02891429	-3.0109619	0.00460688	0.00832277	-32.338484
ENSG00000172264	MACROD2	13995369	16053197	2057828	-2.1996224	-0.6067009	-4.0335389	0.00025511	0.00502254	-30.90998
ENSG00000172508	CARNS1	67414968	67425607	10639	-1.1348784	0.4603247	-2.8779018	0.00653135	0.01175342	-32.406823
ENSG00000173163	COMMD1	61888724	62147247	258523	-1.6126287	-0.4266432	-2.4632496	0.01840334	0.03253101	-34.774218
ENSG00000173402	DAG1	49468703	49535618	66915	-1.1420183	-0.4271003	-2.6565401	0.01147181	0.02047105	-34.379785

ENSG00000173404	INSM1	20368104	20370949	2845	-1.7413922	-0.8334739	-4.0864994	0.00021784	0.00502254	-30.913659
ENSG00000173852	DPY19L1	34928876	35038271	109395	-1.2274146	-0.4844499	-3.1812175	0.00291627	0.00529016	-33.133704
ENSG00000174206	C12orf66	64186316	64222296	35980	-1.5065397	0.13024077	-2.3985902	0.02146427	0.03780039	-33.701192
ENSG00000174226	SNX31	100572889	100663415	90526	-1.0080698	0.2101351	-3.3519616	0.00182335	0.00502254	-31.353203
ENSG00000174332	GLIS1	53506237	53738106	231869	-1.6967066	-0.1733682	-3.2313451	0.00254349	0.00502254	-31.943785
ENSG00000174348	PODN	53062052	53085501	23449	-1.5311797	-0.4835535	-2.4243153	0.02019508	0.03561761	-34.923421
ENSG00000174442	ZWILCH	66504959	66550130	45171	-1.0258967	0.3306509	-3.0595562	0.004048	0.00732317	-32.076552
ENSG00000174748	RPL15	23916591	23924374	7783	-1.1667123	0.26213364	-2.3983432	0.0214768	0.03781875	-33.623697
ENSG00000175344	CHRNA7	31923438	32173018	249580	-2.8899142	-0.2816439	-6.4101994	1.55E-07	0.00013262	-23.167872
ENSG00000175877	TMEM270	73861159	73865890	4731	-1.5947353	-0.8303188	-4.1877722	0.00016079	0.00502254	-30.609677
ENSG00000176095	IP6K1	49724294	49786542	62248	-1.6782852	0.40544338	-4.2308452	0.00014121	0.00502254	-28.77776
ENSG00000176533	GNG7	2511219	2702694	191475	-1.2284603	-0.2177858	-3.0047931	0.00468281	0.00845768	-32.56175
ENSG00000176593		58002061	58011232	9171	-1.1049772	0.35899552	-3.1556921	0.00312547	0.00566735	-31.809622
ENSG00000176749	CDK5R1	32486993	32491253	4260	-1.089636	0.20767192	-2.8421829	0.00716361	0.01287486	-32.673837
ENSG00000177084	POLE	132623753	132687376	63623	-1.0729207	0.3018682	-2.9129673	0.00596172	0.0107427	-32.44206
ENSG00000177119	ANO6	45215987	45482280	266293	-1.3912197	1.15361371	-2.9462087	0.00546499	0.00985519	-31.81669
ENSG00000177189	RPS6KA3	20149911	20267519	117608	-1.0374747	0.0919758	-2.8141512	0.0076993	0.01382658	-32.767644
ENSG00000177354	C10orf71	49299170	49327492	28322	-1.600086	0.11922472	-3.2469971	0.00243669	0.00502254	-31.711491
ENSG00000177374	HIC1	2054154	2063241	9087	-1.7269794	-0.0079014	-2.5801834	0.0138575	0.02464337	-33.491461
ENSG00000178175	ZNF366	72439903	72507410	67507	-1.3206096	-0.7295459	-3.2905632	0.00216143	0.00502254	-33.074887
ENSG00000178252	WDR6	49007062	49015953	8891	-1.6027682	0.04384888	-4.3966705	8.54E-05	0.00502254	-28.428515
ENSG00000178287	SPAG11A	7847876	7868867	20991	-2.3551008	0.46358281	-2.7173894	0.00984848	0.01761451	-32.810438
ENSG00000178567	EPM2AIP1	36985043	36993131	8088	-1.1149526	0.02265616	-2.3641928	0.02327406	0.04088872	-33.920873
ENSG00000178726	THBD	23045633	23049672	4039	-1.3802076	-0.6969082	-2.5196551	0.01606344	0.02847797	-34.959793
ENSG00000179094	PER1	8140472	8156506	16034	-1.1741132	-0.4085658	-2.5252354	0.01584738	0.02810417	-34.63175
ENSG00000179241	LDLRAD3	35943981	36232136	288155	-2.0403258	0.26709554	-2.7433395	0.00922316	0.01651253	-32.86814
ENSG00000179532	DNHD1	6497260	6593758	96498	-1.5361802	1.05019501	-3.1828208	0.00290359	0.00526732	-31.257078
ENSG00000180423	HARBI1	46602861	46617909	15048	-1.0607089	0.21343339	-3.6743077	0.00073136	0.00502254	-30.46781
ENSG00000180785	OR51E1	4643420	4655488	12068	-2.023869	-0.4860698	-3.9771833	0.00030159	0.00502254	-30.986093

ENSG00000180861	LINC01559	13371089	13387167	16078	-2.8290274	-0.5413236	-7.0806864	1.90E-08	2.43E-05	-20.561552
ENSG00000181826	RELL1	37590800	37686376	95576	-1.4632616	-0.4197116	-3.048917	0.0041646	0.00753183	-33.40605
ENSG00000182255	KCNA4	30009730	30017030	7300	-1.4958514	-0.488154	-2.5195569	0.01606726	0.02848381	-34.771319
ENSG00000182308	DCAF4L1	41981756	41986465	4709	-1.1564692	-0.6506576	-2.4554602	0.01874979	0.03312821	-35.059178
ENSG00000182903	ZNF721	425815	499156	73341	-1.5006614	0.10546618	-3.7895043	0.00052366	0.00502254	-30.207024
ENSG00000182986	ZNF320	52860851	52897693	36842	-1.0055411	0.28627441	-3.0083199	0.00463925	0.00837986	-32.245284
ENSG00000183011	NAA38	7856685	7885238	28553	-1.3066955	-0.6959879	-2.5232672	0.01592328	0.02823413	-34.945401
ENSG00000183019	MCEMP1	7676628	7679826	3198	-1.8348845	-0.0616643	-3.0629383	0.00401159	0.00725826	-32.21
ENSG00000183199	HSP90AB3P	87891843	87894015	2172	-1.1320087	0.47227976	-2.8382754	0.00723613	0.01300261	-32.486632
ENSG00000183458	PKD1P3	14911551	14935708	24157	-1.2979808	1.17622767	-2.651238	0.01162437	0.02073573	-32.510866
ENSG00000183597	TANGO2	20017014	20067164	50150	-1.5703133	0.20944763	-2.7716715	0.00858262	0.01538415	-32.84828
ENSG00000183873	SCN5A	38548057	38649687	101630	-1.5280899	-0.6916345	-3.2606453	0.00234706	0.00502254	-33.14828
ENSG00000183943	PRKX	3604340	3713649	109309	-2.8117619	-0.2492991	-6.4150692	1.53E-07	0.00013262	-22.908325
ENSG00000184115		110397406	110435166	37760	-1.1835115	-0.2780774	-2.5957236	0.01333785	0.02373415	-34.38178
ENSG00000185104	FAF1	50437028	50960267	523239	-1.3818326	0.32653336	-4.1349986	0.00018841	0.00502254	-29.079598
ENSG00000185261	KIAA0825	94150851	94618604	467753	-1.1715858	0.38915546	-3.1301943	0.00334858	0.00606804	-31.8454
ENSG00000185615	PDIA2	283164	287215	4051	-2.6221472	-0.7095327	-6.4964409	1.18E-07	0.00010979	-23.298825
ENSG00000185958	FAM186A	50326230	50396622	70392	-1.2870633	3.53349105	-2.6026481	0.01311212	0.0233394	-31.97028
ENSG00000185960	SHOX	624344	659411	35067	-1.5959765	-0.6230185	-2.708597	0.0100691	0.01800433	-34.413928
ENSG00000185988	PLK5	1508023	1536046	28023	-1.9297726	0.05054445	-3.3475181	0.00184602	0.00502254	-31.798394
ENSG00000186106	ANKRD46	100509752	100559784	50032	-1.1979089	0.31390186	-3.5511857	0.00104076	0.00502254	-30.740855
ENSG00000186212	SOWAHB	76894152	76898144	3992	-1.4869623	0.23910261	-2.7133006	0.00995051	0.01779347	-32.992199
ENSG00000186442	KRT3	52789685	52796117	6432	-1.3490695	-0.1932501	-3.9814146	0.00029783	0.00502254	-29.935531
ENSG00000186594	MIR22HG	1711493	1717174	5681	-1.1939621	0.44292274	-3.4867935	0.00124933	0.00502254	-30.864427
ENSG00000186603	HPDL	45326895	45328710	1815	-2.4382683	-0.757231	-5.9089844	7.55E-07	0.00047298	-25.188177
ENSG00000187266	EPOR	11377207	11384342	7135	-1.6087293	-0.0974377	-2.9663121	0.00518371	0.00935232	-32.618506
ENSG00000187607	ZNF286A	15699577	15720787	21210	-2.548504	-0.0053861	-5.0991408	9.70E-06	0.00458603	-26.647532
ENSG00000187642	PERM1	975204	982093	6889	-1.576588	-0.7060342	-3.0334175	0.00434014	0.00784589	-33.737509
ENSG00000187790	FANCM	45135930	45200890	64960	-3.116008	0.0125095	-5.122184	9.02E-06	0.00438177	-26.680666

ENSG00000188021	UBQLN2	56563627	56567868	4241	-1.8109146	-0.8300209	-4.0907866	0.00021507	0.00502254	-30.893137
ENSG00000188314	OR7D1P	9235144	9236076	932	-1.3878403	-0.7191698	-3.3673944	0.00174668	0.00502254	-32.867352
ENSG00000188610	FAM72B	121167646	121185539	17893	-1.0504794	-0.2867946	-2.6260165	0.01237601	0.02204807	-34.182456
ENSG00000188822	CNR2	23870515	23913362	42847	-1.7954399	-0.6893595	-3.541707	0.00106921	0.00502254	-32.301646
ENSG00000188984	AADACL3	12716110	12728760	12650	-1.976846	-0.704028	-3.6490989	0.00078643	0.00502254	-32.003304
ENSG00000189014	SHLD2P3	47689707	47730436	40729	-2.0050448	-0.0678754	-2.9767972	0.00504245	0.00910082	-32.471452
ENSG00000189057	FAM111B	59107185	59127412	20227	-2.0134863	0.1109513	-2.8842061	0.00642532	0.01156494	-32.588431
ENSG00000189283	FHIT	59747277	61251459	1504182	-1.2583399	-0.3217298	-2.5556548	0.01471562	0.02613664	-34.483526
ENSG00000196109	ZNF676	22179089	22215801	36712	-1.1138195	-0.7700987	-3.1765279	0.00295368	0.00535748	-33.337241
ENSG00000196199	MPHOSPH8	19633659	19673441	39782	-1.5746315	-0.7241608	-3.2254178	0.00258509	0.00502254	-33.250035
ENSG00000196364	PRSS29P	1260952	1263845	2893	-1.8327691	0.10994756	-3.4632922	0.00133501	0.00502254	-31.176141
ENSG00000196632	WNK3	54192823	54358642	165819	-1.5018054	-0.7640627	-4.2659631	0.000127	0.00502254	-30.282656
ENSG00000196876	SCN8A	51590266	51812864	222598	-1.2208559	-0.5458235	-3.3695966	0.00173599	0.00502254	-32.697867
ENSG00000196943	NOP9	24299850	24309124	9274	-1.4473183	-0.6828842	-2.8137941	0.00770636	0.01383833	-34.268433
ENSG00000197085	NPSR1-AS1	34346512	34871582	525070	-1.639412	-0.6711882	-2.9697758	0.00513663	0.00926833	-33.800464
ENSG00000197323	TRIM33	114392790	114511203	118413	-2.7080025	-0.5469886	-4.8799467	1.92E-05	0.00502254	-28.35833
ENSG00000197332		11639754	11686569	46815	-2.159901	-0.1241802	-3.7292127	0.000624	0.00502254	-31.156919
ENSG00000197429	IPP	45694324	45750653	56329	-1.5158381	0.20299895	-3.1091007	0.00354446	0.00641998	-32.029288
ENSG00000197503	LINC00477	24566964	24584168	17204	-2.2584874	-0.5267773	-3.9031541	0.00037532	0.00502254	-31.182943
ENSG00000197776	KLHDC1	49693105	49753150	60045	-1.3217941	1.59367993	-2.4284855	0.01999587	0.03527435	-32.866948
ENSG00000197879	MYO1C	1464186	1492686	28500	-1.2235242	1.6816356	-2.642064	0.01189276	0.02120186	-32.372685
ENSG00000197961	ZNF121	9560329	9584504	24175	-2.2340851	0.35564408	-3.4619453	0.00134009	0.00502254	-30.934496
ENSG00000198019	FCGR1B	121087345	121097161	9816	-1.4928255	-0.8070161	-4.4678025	6.87E-05	0.00502254	-29.749667
ENSG00000198053	SIRPA	1894167	1940592	46425	-1.7147974	0.49706821	-2.3702761	0.02294429	0.04032646	-33.499599
ENSG00000198155	ZNF876P	212610	255985	43375	-3.3209153	-0.4121344	-9.253114	2.78E-11	9.36E-08	-14.556974
ENSG00000198168	SVIP	22813799	22830299	16500	-1.402	-0.5869047	-2.7000569	0.01028777	0.01838949	-34.415105
ENSG00000198198	SZT2	43389882	43454247	64365	-1.1192306	0.18702901	-3.5696238	0.00098749	0.00502254	-30.759271
ENSG00000198271	KRTAP4-5	41148924	41149825	901	-1.5495956	-0.7453609	-3.961728	0.00031571	0.00502254	-31.142213
ENSG00000198521	ZNF43	21804946	21852125	47179	-1.2562246	-0.8305335	-3.7632385	0.00056529	0.00502254	-31.857472

ENSG00000198678	OR5BS1P	48559882	48562956	3074	-1.2143492	-0.6885538	-2.6274688	0.01233155	0.02197176	-34.66863
ENSG00000198691	ABCA4	93992834	94121148	128314	-1.2109171	1.76380714	-2.2765983	0.02851989	0.04973517	-33.141439
ENSG00000198865	CCDC152	42756818	42802439	45621	-1.1539268	0.43941944	-2.7886171	0.00821953	0.01474361	-32.611955
ENSG00000200550	RNU6-137P	42712740	42712847	107	-1.8230135	-0.2017348	-2.6401852	0.01194843	0.02129688	-34.093177
ENSG00000201358	RN7SKP193	50068568	50068873	305	-2.5386703	-0.5665011	-4.5293959	5.69E-05	0.00502254	-29.429834
ENSG00000202318		6234345	6234455	110	-1.4931062	-0.8139504	-4.4754589	6.71E-05	0.00502254	-29.728127
ENSG00000204175	GPRIN2	46549044	46555530	6486	-1.6153283	0.0693046	-2.3581796	0.02360423	0.04144986	-33.824026
ENSG00000204362	LINC02783	17189783	17197617	7834	-1.7873077	-0.09452	-3.2080104	0.00271101	0.00502254	-31.916195
ENSG00000205221	VIT	36696690	36814792	118102	-1.2828619	-0.2316791	-2.3549762	0.02378184	0.04175359	-34.827209
ENSG00000205293	LINC01602	57855500	57984126	128626	-1.5344176	-0.822954	-3.9036134	0.00037481	0.00502254	-31.45396
ENSG00000205452		33964547	33976346	11799	-1.4766567	-0.7079811	-3.2907623	0.00216024	0.00502254	-33.03551
ENSG00000205592	MUC19	40393395	40570832	177437	-2.2889742	-0.2252718	-4.0513819	0.00024191	0.00502254	-30.41391
ENSG00000205771	CATSPER2P1	43726918	43747094	20176	-2.5879183	-0.5590448	-7.4878458	5.38E-09	8.79E-06	-19.341443
ENSG00000205937	RNPS1	2253116	2268397	15281	-1.5485259	-0.4692136	-2.5203659	0.01603577	0.02842985	-34.672063
ENSG00000207193		16232231	16232330	99	-1.861903	0.1453379	-2.4785415	0.01774008	0.03137609	-33.515989
ENSG00000207431	RNU6-906P	146639700	146639769	69	-1.033436	0.52188473	-2.4088971	0.02094733	0.0369057	-33.411924
ENSG00000211611	IGKV6-21	89159751	89160366	615	-1.0790829	-0.6550503	-2.3035217	0.02680454	0.04682258	-35.373258
ENSG00000213228	RPL12P38	60433678	60435996	2318	-1.0577195	0.32131789	-3.0091162	0.00462947	0.00836247	-32.18093
ENSG00000213851		43410041	43410937	896	-1.8260024	0.2396333	-2.3999562	0.0213951	0.03768227	-33.673883
ENSG00000213988	ZNF90	20077994	20127076	49082	-1.7721974	0.22335446	-4.040154	0.00025014	0.00502254	-29.387913
ENSG00000214465	SMARCE1P6	26149204	26150731	1527	-1.4836597	-0.3833268	-2.4330783	0.01977853	0.03489895	-34.834666
ENSG00000214660	SLC29A4P2	63556598	63562588	5990	-1.9016433	-0.779694	-4.8801283	1.92E-05	0.00502254	-28.472775
ENSG00000214719		30576464	30672789	96325	-2.6400674	-0.278324	-6.6752308	6.74E-08	6.60E-05	-22.301257
ENSG00000214756	CSKMT	62665309	62668496	3187	-1.0745982	0.23400669	-3.4067782	0.00156472	0.00502254	-31.183236
ENSG00000215021	PHB2	6965327	6970780	5453	-1.8769477	-0.7894353	-4.6274522	4.21E-05	0.00502254	-29.25563
ENSG00000215045	GRID2IP	6497462	6551436	53974	-1.2345492	-0.676126	-3.0336472	0.00433749	0.00784135	-33.693968
ENSG00000215114	UBXN2B	58411359	58451501	40142	-1.3994629	-0.8626767	-3.7981802	0.00051057	0.00502254	-31.767366
ENSG00000215126	CBWD6	41131306	41199261	67955	-2.1137462	-0.1616486	-3.7969833	0.00051236	0.00502254	-30.765867
ENSG00000215190	LINC00680	57946074	57961501	15427	-1.3698143	-0.7275791	-2.9480959	0.00543799	0.00980683	-33.954801

ENSG00000215196	BASP1-AS1	17089296	17217047	127751	-2.4635647	-0.5840537	-5.3895503	3.89E-06	0.00203733	-26.775377
ENSG00000215252	GOLGA8B	34525095	34588503	63408	-1.4074814	-0.2935448	-2.573531	0.01408558	0.02504319	-34.446325
ENSG00000215386	MIR99AHG	15928296	16645467	717171	-1.586194	0.50582682	-4.1436288	0.00018359	0.00502254	-28.947723
ENSG00000215572	ESRRAP1	19560013	19561269	1256	-1.3001445	-0.8147667	-2.7660998	0.00870523	0.0156034	-34.409239
ENSG00000216364	MRPL42P2	16171606	16172031	425	-1.8023602	0.2847048	-2.2789138	0.02836859	0.04948094	-33.888637
ENSG00000217165	ANKRD18EP	39110321	39112952	2631	-1.1408361	-0.7184081	-2.3481905	0.02416204	0.04239488	-35.339555
ENSG00000219682		25140003	25141403	1400	-1.6743948	0.18800582	-2.6230175	0.01246831	0.02221029	-33.241268
ENSG00000220008	LINGO3	2289784	2292024	2240	-1.7029807	-0.7967334	-4.5206698	5.84E-05	0.00502254	-29.578575
ENSG00000223518	CSNK1A1P1	36798596	36818459	19863	-1.4830303	-0.7698131	-4.4305962	7.70E-05	0.00502254	-29.8062
ENSG00000223855		520391	525238	4847	-1.5930843	0.15907625	-2.363577	0.02330767	0.04094511	-33.84988
ENSG00000224042	MTND4P6	117263917	117264388	471	-1.0836912	0.34130881	-3.0958762	0.00367274	0.00664965	-31.978783
ENSG00000224295	OLFM5P	5518441	5524955	6514	-1.296918	-0.6966946	-3.3180198	0.00200345	0.00502254	-32.938882
ENSG00000224309	ANKRD30BP2	13038160	13067033	28873	-1.7767039	-0.1016763	-2.9571747	0.00530982	0.00957698	-32.591601
ENSG00000224336	FAM197Y1	9544917	9547147	2230	-1.2289181	-0.7789707	-2.6765647	0.01091215	0.01948719	-34.663919
ENSG00000224902	GAGE12H	49579983	49587301	7318	-1.3247023	-0.6669693	-2.627063	0.01234396	0.02199314	-34.686422
ENSG00000225014	KCTD9P1	20111824	20112990	1166	-2.7729704	-0.3255962	-4.9592449	1.50E-05	0.00502254	-27.894376
ENSG00000225045	MTND5P27	82815809	82817528	1719	-2.9008844	-0.3082577	-4.4315189	7.68E-05	0.00502254	-29.295589
ENSG00000225213		16721352	16748377	27025	-1.1316768	-0.7168382	-3.4224521	0.00149748	0.00502254	-32.657567
ENSG00000225526	MKRN2OS	12514934	12561059	46125	-1.0108498	0.52827815	-2.5755515	0.01401594	0.02492021	-33.018585
ENSG00000225559		138163440	138164637	1197	-1.0307868	0.61576606	-2.3290163	0.02526618	0.04424147	-33.490069
ENSG00000225766	DHRS4L1	24036453	24051028	14575	-2.9841372	-0.3221242	-7.046801	2.11E-08	2.58E-05	-20.990715
ENSG00000226258	GRM7-AS3	6631008	6805479	174471	-1.1303079	-0.171684	-3.0299308	0.00438058	0.00791819	-32.404928
ENSG00000226314	ZNF192P1	28161769	28169594	7825	-1.1850241	-0.4257966	-2.8415924	0.00717452	0.01289361	-33.905056
ENSG00000226401		63632608	63636617	4009	-1.3510088	-0.8677303	-3.6045129	0.00089378	0.00502254	-32.311049
ENSG00000226480	OR7H1P	9278448	9279369	921	-2.310227	-0.2990139	-3.077023	0.00386323	0.00699172	-33.263365
ENSG00000226521		20716481	20737127	20646	-2.1294702	-0.2254005	-3.2934188	0.00214446	0.00502254	-31.843715
ENSG00000226578		37775371	37784131	8760	-1.2697731	0.0493334	-3.1344016	0.00331075	0.0060007	-31.979166
ENSG00000227388		35772163	35790432	18269	-2.1515935	-0.2677081	-4.2628539	0.0001282	0.00502254	-29.250722
ENSG00000228277	UMLILO	73710302	73714527	4225	-2.2920018	-0.485115	-3.8696706	0.00041416	0.00502254	-31.299958

ENSG00000228486	C2orf92	97664217	97703064	38847	-1.8292775	-0.174881	-3.0309628	0.00436858	0.00789675	-32.445385
ENSG00000228903	RASA4CP	44026951	44041892	14941	-1.2360889	0.17187496	-3.4691857	0.00131301	0.00502254	-31.060937
ENSG00000229306		12999676	13016692	17016	-1.144769	0.17878564	-3.2527365	0.00239861	0.00502254	-31.688704
ENSG00000229424		36750687	36763081	12394	-2.0529406	0.29228091	-2.6858382	0.01066161	0.01904419	-32.980952
ENSG00000229492		17418697	17419828	1131	-2.9841733	-0.4181453	-6.3193447	2.06E-07	0.00016606	-23.667115
ENSG00000229558	SACS-AS1	23418971	23428869	9898	-1.9434959	-0.1091553	-3.063077	0.0040101	0.00725582	-32.321672
ENSG00000230333		11180902	11520175	339273	-1.7400157	0.13089941	-3.0568409	0.00407746	0.00737572	-32.158985
ENSG00000230650		110360608	110363737	3129	-1.0058818	0.19316515	-2.9944634	0.0048126	0.00869035	-32.277023
ENSG00000230662	TNPO1P2	18460391	18463055	2664	-2.2329079	-0.6967049	-5.0940406	9.86E-06	0.00461939	-27.767052
ENSG00000230699		911435	914948	3513	-1.6863573	0.07019524	-2.6535942	0.01155634	0.02061712	-33.237085
ENSG00000230787	PSAT1P3	79054945	79056055	1110	-1.6728043	-0.8047408	-4.4406834	7.47E-05	0.00502254	-29.824533
ENSG00000230790		11740997	11745301	4304	-2.3485929	-0.4398642	-3.970731	0.00030741	0.00502254	-30.776451
ENSG00000230946	HNRNPA1P68	100941017	100941995	978	-2.5274485	-0.4555144	-2.5963659	0.01331676	0.02369818	-33.942288
ENSG00000231105	ECE1-AS1	21293290	21299874	6584	-2.5836657	-0.5904828	-4.8600467	2.05E-05	0.00502254	-28.423658
ENSG00000231163	CSMD2-AS1	33868953	33893726	24773	-2.6266736	-0.3687178	-3.1436104	0.00322937	0.00585438	-32.961355
ENSG00000231205	ZNF826P	20340269	20424969	84700	-1.1510873	-0.4110197	-2.3915242	0.0218253	0.03841361	-34.926794
ENSG00000231755	CHODL-AS1	17835016	17885608	50592	-2.3072009	-0.7629814	-5.6805617	1.55E-06	0.00088154	-25.920711
ENSG00000231965		11175051	11175570	519	-1.2440566	-0.7444872	-2.905219	0.00608344	0.01095616	-34.070506
ENSG00000232268	OR52I1	4593750	4595013	1263	-1.3007515	-0.6357566	-2.5639252	0.01442099	0.02562769	-34.788806
ENSG00000232431		28788243	28792002	3759	-1.9395821	-0.5515203	-2.8926105	0.00628648	0.01131654	-33.909268
ENSG00000232729		74688864	74729001	40137	-1.8236901	-0.2179037	-2.9696305	0.0051386	0.00927157	-32.545213
ENSG00000232977	LINC00327	23465776	23487712	21936	-1.7659142	0.0086394	-2.6101319	0.01287211	0.02291975	-33.305684
ENSG00000233067	PTCHD1-AS	22191895	22235358	43463	-2.4262815	-0.7201387	-6.5201296	1.10E-07	0.00010369	-23.225331
ENSG00000233098	CCDC144NL-AS1	20868433	21002276	133843	-1.6277144	0.20376901	-3.1073204	0.00356148	0.00644994	-31.937577
ENSG00000233256		13415202	13423133	7931	-3.5826658	-0.1342623	-4.7240466	3.12E-05	0.00502254	-28.311597
ENSG00000233403		25878339	25893544	15205	-1.8224935	-0.5866285	-3.2474645	0.00243357	0.00502254	-33.030238
ENSG00000233670	PIRT	10822470	10838087	15617	-1.6318791	-0.4579555	-3.2639225	0.00232601	0.00502254	-32.918875
ENSG00000234174		113171535	113175360	3825	-1.026139	0.30200004	-3.0485868	0.00416827	0.00753796	-32.109382

ENSG00000234315	OSTCP5	45069977	45070429	452	-2.9025106	-0.0817965	-3.3849635	0.00166314	0.00502254	-32.192242
ENSG00000234338		64835280	64836882	1602	-1.827974	-0.4903944	-3.1078919	0.00355601	0.00644046	-33.346582
ENSG00000234429	ANKRD11P1	81194337	81201184	6847	-1.4123821	-0.695709	-2.891056	0.00631195	0.01136201	-34.084296
ENSG00000234541	CHEK2P5	38713195	38719229	6034	-1.1182254	-0.6649193	-2.4072289	0.02103022	0.03704932	-35.109326
ENSG00000234607		15969632	15970194	562	-1.5540897	0.15542568	-2.3796338	0.02244525	0.03947768	-33.801425
ENSG00000234736	FAM170B-AS1	49121839	49151547	29708	-1.4192258	-0.1702123	-3.209006	0.00270366	0.00502254	-31.885192
ENSG00000235084	CHCHD2P6	15604597	15605043	446	-1.2109106	0.25597297	-3.8780499	0.00040409	0.00502254	-29.864348
ENSG00000235258	NDUFB4P6	112286057	112286422	365	-1.1745443	-0.8849472	-3.5434388	0.00106396	0.00502254	-32.492839
ENSG00000235430	ZSWIM5P1	15768717	15771426	2709	-2.2322557	-0.6471262	-4.7844322	2.59E-05	0.00502254	-28.675473
ENSG00000235445		17983205	17983619	414	-1.3149452	-0.7157257	-3.8638994	0.00042124	0.00502254	-31.403214
ENSG00000235643	LINC01647	11609468	11613358	3890	-1.6862189	-0.1485918	-4.3982086	8.50E-05	0.00502254	-28.758831
ENSG00000235927	NEXN-AS1	77881348	77889539	8191	-1.1813684	-0.7104007	-2.4224132	0.02028653	0.03577657	-35.173733
ENSG00000236013		139976352	140093721	117369	-1.0386135	0.35060448	-2.9776534	0.00503108	0.0090806	-32.262891
ENSG00000236188	PRKX-AS1	3659487	3668192	8705	-2.64872	-0.4191042	-4.1009091	0.00020866	0.00502254	-30.408694
ENSG00000236341		56963886	56996757	32871	-2.4603852	-0.3916856	-4.7011811	3.35E-05	0.00502254	-28.713374
ENSG00000236377		32905662	32906584	922	-1.171433	0.03802761	-3.2656649	0.00231489	0.00502254	-31.712235
ENSG00000236442	ANKRD54P1	45151445	45151915	470	-1.0609317	-0.8667405	-2.404742	0.02115435	0.03726435	-35.283809
ENSG00000236908	LINC02827	3318718	3325343	6625	-1.0334342	-0.7668486	-2.904969	0.0060874	0.01096294	-34.054389
ENSG00000236965	OR52N3P	5800364	5801297	933	-1.069609	-0.7960102	-2.4105994	0.02086305	0.03675961	-35.264963
ENSG00000237400		36463023	36504513	41490	-1.238775	-0.4243619	-2.7356934	0.00940347	0.01683144	-34.187983
ENSG00000237470	DCLRE1CP1	15015370	15021863	6493	-1.1704991	0.36095081	-3.3189325	0.00199839	0.00502254	-31.348913
ENSG00000237521	OR7E24	9247344	9252625	5281	-1.5072034	-0.7384565	-3.9591866	0.0003181	0.00502254	-31.143105
ENSG00000237541	HLA-DQA2	32741391	32747198	5807	-3.4661221	-0.6628692	-9.0594501	4.86E-11	1.54E-07	-15.422644
ENSG00000237737	DCTN1-AS1	74385474	74393882	8408	-2.1041132	-0.4771211	-3.7456234	0.00059498	0.00502254	-31.530589
ENSG00000237799	CICP11	55736779	55739605	2826	-2.0794428	-0.7613757	-5.0300117	1.20E-05	0.00502254	-27.993184
ENSG00000237919	LRRC7-AS1	70013982	70031222	17240	-1.6369036	0.0659591	-2.2992084	0.02707297	0.0472731	-33.977296
ENSG00000239149	SNORA59A	12507246	12507397	151	-1.7204653	-0.2556279	-2.2971324	0.02720302	0.04749711	-34.896886
ENSG00000239627	RPL12P20	41389115	41389593	478	-1.2223107	-0.4453536	-4.1178547	0.00019834	0.00502254	-29.531437
ENSG00000240038	AMY2B	103553815	103579534	25719	-1.3510088	-0.8677303	-3.6045129	0.00089378	0.00502254	-32.311049

ENSG00000240230	COX19	898778	975549	76771	-1.3127584	-0.8416234	-3.7887471	0.00052482	0.00502254	-31.78625
ENSG00000241111		64067964	64103131	35167	-2.7630635	-0.3537927	-4.2261656	0.00014322	0.00502254	-30.005541
ENSG00000241549	GUSBP2	26871484	26956554	85070	-1.9365658	-0.5786466	-5.2280164	6.47E-06	0.00326066	-27.136099
ENSG00000242048		151806490	151810820	4330	-1.0157218	0.01532262	-3.7867145	0.00052794	0.00502254	-30.226484
ENSG00000242439		30830901	30831318	417	-1.2916296	-0.6091731	-3.7661531	0.00056052	0.00502254	-31.390774
ENSG00000243335	KCTD7	66628881	66649067	20186	-1.9656779	0.14318446	-2.773655	0.00853936	0.01530916	-32.803129
ENSG00000247157	LINC01252	11548030	11590369	42339	-1.0532671	0.25014362	-3.029674	0.00438358	0.00792333	-32.14572
ENSG00000247556	OIP5-AS1	41283990	41309737	25747	-1.2515974	0.25323709	-3.216853	0.00264633	0.00502254	-31.720348
ENSG00000248265		54058254	54122235	63981	-1.9128323	0.02895884	-3.567298	0.00099406	0.00502254	-31.199557
ENSG00000248461	LINC02119	38025568	38184717	159149	-1.2976204	-0.8535106	-3.7462784	0.00059385	0.00502254	-31.909533
ENSG00000248789	LINC02118	52008031	52080987	72956	-1.1633405	-0.7502376	-2.7983955	0.00801655	0.01438624	-34.335573
ENSG00000248843		3323977	3324762	785	-2.3187103	-0.1712984	-4.2853999	0.00011974	0.00502254	-29.592838
ENSG00000248918		50969660	50970187	527	-1.5672347	-0.744131	-3.4921095	0.00123069	0.00502254	-32.545205
ENSG00000249664		83012285	83013109	824	-1.1029314	-0.5420678	-2.8430467	0.00714766	0.01284749	-34.003366
ENSG00000249887		41924180	41924380	200	-1.3161791	-0.7140159	-2.6667585	0.01118299	0.01996426	-34.625942
ENSG00000249908	BRD9P2	767382	768930	1548	-1.7391418	-0.0010375	-2.5671073	0.01430907	0.02543384	-33.459719
ENSG00000249920	HNRNPA1P55	73938604	73939558	954	-2.1867089	0.06149339	-3.0827398	0.0038045	0.00688659	-32.102098
ENSG00000250050	MTND4P9	25718082	25719745	1663	-2.3425042	0.33480481	-2.8684838	0.00669279	0.01203832	-32.474206
ENSG00000250432	FAM242C	54085132	54125992	40860	-1.3698682	-0.5009876	-3.0457744	0.00419964	0.00759393	-33.467785
ENSG00000250462	LRRC37BP1	30629680	30637466	7786	-1.0712045	-0.7266335	-2.4691294	0.01814569	0.03208399	-35.113776
ENSG00000250597		34657606	34669432	11826	-2.7901225	-0.7247948	-7.0299248	2.22E-08	2.66E-05	-21.616614
ENSG00000250611		20037560	20037972	412	-1.2360923	-0.8791968	-3.57608	0.00096946	0.00502254	-32.397636
ENSG00000251314		95962001	96631085	669084	-1.1279081	0.56528526	-2.7000512	0.01028792	0.01838949	-32.725924
ENSG00000252206	RNU7-40P	56352387	56352450	63	-1.3459639	-0.6392396	-2.3402811	0.02461213	0.04316352	-35.279179
ENSG00000252515	RNU6-1171P	148739379	148739479	100	-1.0807545	0.30418883	-3.0321856	0.00435439	0.00787138	-32.135092
ENSG00000253230	MIR124-1HG	9899871	9906678	6807	-1.1793446	0.00187353	-2.5247073	0.01586771	0.02813931	-33.509494
ENSG00000253490	LINC02099	29748309	29798492	50183	-1.4436941	-0.1776023	-2.906052	0.00607024	0.01093276	-32.748768
ENSG00000253552	HOXA-AS2	27107777	27134302	26525	-1.2570949	-0.3849998	-2.8091896	0.00779792	0.01399715	-33.972074
ENSG00000253998	IGKV2-29	89234174	89234912	738	-1.1745443	-0.8849472	-3.5434388	0.00106396	0.00502254	-32.492839

ENSG00000254306		37600537	37625873	25336	-1.2830794	-0.6914836	-2.9064832	0.00606342	0.01092084	-34.054097
ENSG00000254701		70197255	70207745	10490	-1.9288478	-0.224028	-2.5106409	0.01641814	0.02908862	-34.242048
ENSG00000255328		327171	330122	2951	-1.8094047	-0.475594	-3.5769098	0.00096717	0.00502254	-32.068342
ENSG00000255524	NPIPB8	28637654	28658744	21090	-1.8569493	0.22758041	-2.7647574	0.00873501	0.01565626	-32.867893
ENSG00000255608		4838955	4842675	3720	-2.48096	-0.7462457	-6.2113936	2.90E-07	0.00021428	-24.215951
ENSG00000255947		61654665	61655702	1037	-2.3434691	-0.1495678	-3.748428	0.00059016	0.00502254	-31.206241
ENSG00000256146		5315961	5319347	3386	-1.3766205	-0.6275156	-3.7346899	0.00061416	0.00502254	-31.751547
ENSG00000256197	TSPAN9-IT1	3149797	3151476	1679	-2.0789431	0.14864364	-2.5603238	0.01454862	0.02584599	-33.292903
ENSG00000256642	LINC00273	34158585	34160036	1451	-1.4871313	-0.362613	-2.4206284	0.0203727	0.035925	-34.852886
ENSG00000256713	PGA5	61241175	61251444	10269	-1.7359078	-0.1954353	-2.8873569	0.00637294	0.01147141	-32.826246
ENSG00000257331	RACGAP1P1	45063473	45065351	1878	-1.1745443	-0.8849472	-3.5434388	0.00106396	0.00502254	-32.492839
ENSG00000257343		52746596	52748308	1712	-2.5046062	-0.4888785	-5.1256237	8.93E-06	0.00437417	-27.48676
ENSG00000257826		36061026	36067190	6164	-2.5759689	-0.162605	-4.8708043	1.98E-05	0.00502254	-27.801032
ENSG00000258137		54353661	54497688	144027	-1.0796031	-0.7812471	-2.3655667	0.02319921	0.04076387	-35.357988
ENSG00000258648	UBE2CP1	30683045	30683598	553	-1.9251703	-0.3389691	-3.7607198	0.00056944	0.00502254	-31.381443
ENSG00000259336		35099022	35169698	70676	-1.0460802	-0.6055939	-2.7850264	0.00829525	0.01487746	-34.23063
ENSG00000260599		22520995	22527949	6954	-1.3385734	-0.7546986	-3.4074697	0.0015617	0.00502254	-32.697597
ENSG00000260644	HERC2P5	32741314	32776196	34882	-1.2279711	-0.5618164	-2.2744655	0.02865989	0.04997609	-35.304392
ENSG00000260774		213898	217279	3381	-1.4470592	-0.6590308	-3.0852135	0.00377936	0.0068413	-33.508942
ENSG00000260840	LINC01964	85061213	85067347	6134	-1.3356142	-0.6820928	-3.2187843	0.0026324	0.00502254	-33.253842
ENSG00000261037		6019029	6022283	3254	-1.931048	-0.5468248	-3.1430647	0.00323414	0.00586283	-33.297784
ENSG00000261069		25087661	25088896	1235	-1.3465306	-0.5494648	-3.1312247	0.00333928	0.00605139	-33.323178
ENSG00000261211		6680309	6683633	3324	-1.6931868	-0.5569355	-3.6229002	0.0008479	0.00502254	-31.868746
ENSG00000261293		12093627	12095307	1680	-1.7771488	-0.6990731	-3.5663153	0.00099685	0.00502254	-32.313394
ENSG00000261340	LINC01616	29980113	29982392	2279	-1.1805331	-0.4011694	-2.4181908	0.02049091	0.03612754	-34.867017
ENSG00000261505		1317891	1322845	4954	-1.2863214	-0.4404857	-3.0859017	0.00377239	0.00682915	-33.351919
ENSG00000261509	TP53TG3B	33360274	33363478	3204	-1.5638235	-0.7303256	-3.9172473	0.00036005	0.00502254	-31.257921
ENSG00000261548	HLA-P	29800415	29802425	2010	-1.7029807	-0.7967334	-4.5206698	5.84E-05	0.00502254	-29.578575
ENSG00000261730	FOXF2-DT	1383790	1385066	1276	-4.2269098	-0.2596782	-6.9231971	3.10E-08	3.41E-05	-21.500823

ENSG00000262081	IL9RP4	80140	88570	8430	-1.1633405	-0.7502376	-2.7983955	0.00801655	0.01438624	-34.335573
ENSG00000262503		6927472	6928191	719	-1.1745443	-0.8849472	-3.5434388	0.00106396	0.00502254	-32.492839
ENSG00000262953	EIF4A1P9	2586330	2587866	1536	-2.0732607	0.16722324	-3.4304208	0.00146436	0.00502254	-31.151578
ENSG00000263244		9104848	9113181	8333	-1.4612543	-0.7174108	-2.7396168	0.00931054	0.01666732	-34.46565

Table S4: Gene Ontology (GO) analysis

GO terms with biological processes significantly enriched by upregulated DEGs

GO term_name	term_id	adjusted_p_value	term_size	query_size	intersection_size	intersections
developmental process	GO:0032502	1.26E-14	6326	213	68	RSPO4,CRACR2A,PMP22,SLC26A8,BAMBI,TTLL7,ZFP36L2,PARP11,DMRT2,ACTB,FHL2,ZFHX2,HAP1,MEF2C,WNT7A,NR1D2,SCARB2,CCHCR1,ABCC8,ABCB5,CATSPER4,EMP1,STAT5B,TWF2,TENT5C,WWC3,TEAD4,STRAP,CLSTN1,ARHGAP22,IGFBP3,TPGS1,RAPGEFL1,GRM7,EDA2R,IFT140,XKR4,LHFPL2,NTN1,FBN3,HDAC9,TFAP2B,UCMA,NFATC4,REST,NRG1,CRAMP1,RHOBTB1,MYLK2,KIAA1217,FAM9B,AKAP3,HERC2,ELAVL4,N4BP2L2,UBE3A,RRM2,VANGL1,SLC6A17,BHLHA9,PALM,ITFG2,ICAM1,BMP2,AGBL4,CIAO3,FLNB,DKK4
localization	GO:0051179	6.65E-14	6425	211	67	CRACR2A,CENPA,OCLN,SLC26A8,SLC6A13,PLEKHM2,BAMBI,GRB7,SLC25A41,KCNQ5,OPTN,STXBP6,PARP11,ACTB,CAVIN1,HAP1,MEF2C,WNT7A,SLCO1C1,ERLEC1,SCARB2,RHBDF1,CCHCR1,ABCC8,ABCB5,HASPIN,CATSPER4,STAT5B,TWF2,ITPR2,WWC3,TMEM167B,CLSTN1,IGFBP3,TPGS1,SNX7,GRM7,C17ORF75,IFT140,GCK,XKR4,NTN1,TXNDC5,HDAC9,TFAP2B,SNX32,REST,NRG1,RHOBTB1,MYLK2,AKAP3,SPAG17,HERC2,PEX12,AQP7,UBE3A,SLC6A17,SRPX,PALM,ICAM1,TBC1D26,BMP2,AGBL4,SLC26A1,TRAM2,ARRDC2,ARL5C
anatomical structure development	GO:0048856	1.00E-13	5718	213	63	RSPO4,CRACR2A,PMP22,SLC26A8,BAMBI,TTLL7,ZFP36L2,DMRT2,ACTB,FHL2,ZFHX2,HAP1,MEF2C,WNT7A,NR1D2,SCARB2,ABCC8,ABCB5,CATSPER4,EMP1,STAT5B,TWF2,TENT5C,TEAD4,STRAP,CLSTN1,ARHGAP22,IGFBP3,TPGS1,RAPGEFL1,GRM7,EDA2R,IFT140,XKR4,LHFPL2,NTN1,FBN3,HDAC9,TFAP2B,UCMA,NFATC4,REST,NRG1,CRAMP1,RHOBTB1,MYLK2,KIAA1217,FAM9B,AKAP3,ELAVL4,N4BP2L2,UBE3A,RRM2,VANGL1,SLC6A17,PALM,ITFG2,ICAM1,BMP2,AGBL4,CIAO3,FLNB,DKK4
multicellular organismal process	GO:0032501	7.82E-13	7474	216	72	RSPO4,CRACR2A,OCLN,PMP22,SIGIRR,SLC26A8,SLC6A13,BAMBI,TTLL7,ZFP36L2,PARP11,DMRT2,ACTB,FHL2,ZFHX2,HAP1,MEF2C,WNT7A,SLCO1C1,NR1D2,SCARB2,ABCC8,ABCB5,CATSPER4,STAT5B,TWF2,TENT5C,WWC3,TEAD4,STRAP,OR1E1,CLSTN1,ARHGAP22,IGFBP3,TPGS1,RAPGEFL1,GRM7,PTGFR,IFT140,OR8H2,LHFPL2,NTN1,HDAC9,TFAP2B,UCMA,NFATC4,REST,NRG1,CRAMP1,GUCY2D,MYLK2,KIAA1217,FAM9B,AKAP3,HERC2,ELAVL4,N4BP2L2,ADRA1D,UBE3A,RRM2,VANGL1,SLC6A17,PALM,ITFG2,MAPK13,BMP2,CELF2,AGBL4,CIAO3,FLNB,DKK4,TAS2R10

transport	GO:0006810	2.84E-12	4760	282	65	CRACR2A,OCLN,SLC26A8,SLC6A13,SLC25A41,KCNQ5,OPTN,STXBP6,PARP11,ACTB,CAVIN1,HAP1,MEF2C,WNT7A,SLCO1C1,ERLEC1,SCARB2,RHBDF1,CCHCR1,ABCC8,ABCB5,CATSPER4,STAT5B,ITPR2,TMEM167B,CLSTN1,SNX7,GRM7,C17ORF75,IFT140,GCK,XKR4,NTN1,TXNDC5,TFAP2B,SNX32,REST,NRG1,RHOBTB1,MYLK2,SPAG17,HERC2,PEX12,AQP7,UBE3A,SLC6A17,SRPX,TBC1D26,BMP2,AGBL4,SLC26A1,TRAM2,ARRDC2,ARL5C,CBARP,CLCN4,SLC17A3,SNX13,SLC4A4,HECW1,TMEM230,SLC5A7,SLC16A1,HNRNPA2B1,MICALL2
establishment of localization	GO:0051234	3.81E-12	4920	282	66	CRACR2A,CENPA,OCLN,SLC26A8,SLC6A13,SLC25A41,KCNQ5,OPTN,STXBP6,PARP11,ACTB,CAVIN1,HAP1,MEF2C,WNT7A,SLCO1C1,ERLEC1,SCARB2,RHBDF1,CCHCR1,ABCC8,ABCB5,CATSPER4,STAT5B,ITPR2,TMEM167B,CLSTN1,SNX7,GRM7,C17ORF75,IFT140,GCK,XKR4,NTN1,TXNDC5,TFAP2B,SNX32,REST,NRG1,RHOBTB1,MYLK2,SPAG17,HERC2,PEX12,AQP7,UBE3A,SLC6A17,SRPX,TBC1D26,BMP2,AGBL4,SLC26A1,TRAM2,ARRDC2,ARL5C,CBARP,CLCN4,SLC17A3,SNX13,SLC4A4,HECW1,TMEM230,SLC5A7,SLC16A1,HNRNPA2B1,MICALL2
signaling	GO:0023052	2.22E-11	6411	197	60	RSPO4,CRACR2A,PMP22,SIGIRR,SIT1,BAMBI,GRB7,ZFP36L2,OPTN,MAST4,FHL2,HAP1,MEF2C,WNT7A,NR1D2,ERLEC1,RHBDF1,ABCC8,HASPIN,STAT5B,PPP1R1A,ITPR2,WWC3,TEAD4,STRAP,OR1E1,CLSTN1,ARHGAP22,IGFBP3,PRKAR2A,TPGS1,RAPGEFL1,GRM7,PTGFR,EDA2R,IFT140,GCK,OR8H2,NTN1,TFAP2B,UCMA,NFATC4,REST,NRG1,GUCY2D,RHOBTB1,MYLK2,AKAP3,ELAVL4,ADRA1D,UBE3A,ARAP2,VANGL1,SRPX,PALM,ITFG2,LRRC39,MAPK13,ICAM1,BMP2
cell communication	GO:0007154	3.22E-11	6465	197	60	RSPO4,CRACR2A,PMP22,SIGIRR,SIT1,BAMBI,GRB7,ZFP36L2,OPTN,MAST4,FHL2,HAP1,MEF2C,WNT7A,NR1D2,ERLEC1,RHBDF1,ABCC8,HASPIN,STAT5B,PPP1R1A,ITPR2,WWC3,TEAD4,STRAP,OR1E1,CLSTN1,ARHGAP22,IGFBP3,PRKAR2A,TPGS1,RAPGEFL1,GRM7,PTGFR,EDA2R,IFT140,GCK,OR8H2,NTN1,TFAP2B,UCMA,NFATC4,REST,NRG1,GUCY2D,RHOBTB1,MYLK2,AKAP3,ELAVL4,ADRA1D,UBE3A,ARAP2,VANGL1,SRPX,PALM,ITFG2,LRRC39,MAPK13,ICAM1,BMP2
cell differentiation	GO:0030154	3.92E-10	4128	209	47	CRACR2A,PMP22,SLC26A8,BAMBI,TTLL7,ZFP36L2,PARP11,ACTB,FHL2,ZFHX2,HAP1,MEF2C,WNT7A,NR1D2,SCARB2,CCHCR1,ABCC8,ABCB5,CATSPER4,STAT5B,TWF2,TEAD4,STRAP,ARHGAP22,IGFBP3,TPGS1,GRM7,EDA2R,IFT140,NTN1,HDAC9,TFAP2B,UCMA,NFATC4,REST,NRG1,MYLK2,FAM9B,ELAVL4,N4BP2L2,UBE3A,ITFG2,ICAM1,BMP2,AGBL4,CIAO3,FLNB

cellular response to stimulus	GO:0051716	7.22E-10	7,346.00	197	62	RSPO4,CRACR2A,SIGIRR,SIT1,BAMBI,GRB7,ZFP36L2,OPTN,MAST4,ACTB,FHL2,HAP1,MEF2C,WNT7A,NR1D2,ERLEC1,RHBDF1,ABCC8,HASPIN,STAT5B,PPP1R1A,TWF2,ITPR2,WWC3,TEAD4,STRAP,OR1E1,ARHGAP22,IGFBP3,PRKAR2A,RAPGEFL1,GRM7,PTGFR,EDA2R,IFT140,GCK,OR8H2,NTN1,HDAC9,TFAP2B,UCMA,UGT2B28,NFATC4,REST,NRG1,GUCY2D,RHOBTB1,AKAP3,GBP6,HERC2,ELAVL4,ADRA1D,UBE3A,ARAP2,VANGL1,SRPX,PALM,ITFG2,LRRC39,MAPK13,ICAM1,BMP2
cellular developmental process	GO:0048869	7.93E-10	4210	209	47	CRACR2A,PMP22,SLC26A8,BAMBI,TTLL7,ZFP36L2,PARP11,ACTB,FHL2,ZFHX2,HAP1,MEF2C,WNT7A,NR1D2,SCARB2,CCHCR1,ABCC8,ABCB5,CATSPER4,STAT5B,TWF2,TEAD4,STRAP,ARHGAP22,IGFBP3,TPGS1,GRM7,EDA2R,IFT140,NTN1,HDAC9,TFAP2B,UCMA,NFATC4,REST,NRG1,MYLK2,FAM9B,ELAVL4,N4BP2L2,UBE3A,ITFG2,ICAM1,BMP2,AGBL4,CIAO3,FLNB
multicellular organism development	GO:0007275	2.66E-09	5114	213	52	RSPO4,CRACR2A,PMP22,BAMBI,TTLL7,ZFP36L2,DMRT2,ACTB,FHL2,ZFHX2,HAP1,MEF2C,WNT7A,NR1D2,SCARB2,ABCC8,ABCB5,STAT5B,TWF2,TENT5C,TEAD4,STRAP,CLSTN1,ARHGAP22,RAPGEFL1,GRM7,IFT140,LHFPL2,NTN1,HDAC9,TFAP2B,UCMA,NFATC4,REST,NRG1,CRAMP1,MYLK2,KIAA1217,AKAP3,ELAVL4,N4BP2L2,UBE3A,RRM2,VANGL1,SLC6A17,PALM,ITFG2,BMP2,AGBL4,CIAO3,FLNB,DKK4
organonitrogen compound metabolic process	GO:1901564	3.24E-09	6338	273	69	TECR,OCLN,EIF3M,RHBDL3,GRB7,ABHD12B,TTLL7,ZFP36L2,MAST4,PARP11,EIF2S3B,ACTB,HAP1,MEF2C,AGMAT,WNT7A,SLCO1C1,RPL19,ERLEC1,RHBDF1,HASPIN,STAT5B,TTLL10,LCMT2,IGFBP3,PRKAR2A,TPGS1,ART4,GCK,PDHB,HDAC9,REST,NRG1,GUCY2D,MYLK2,HERC2,DYRK4,PEX12,ELAVL4,B3GALT6,UBE3A,MRPL4,LRRC39,MAPK13,CELA2B,BMP2,AGBL4,SLC26A1,HS3ST2,FKBP2,ALG1,ZAR1,RNF170,ADAMTS16,GPRC5A,TPSAB1,BAG2,CCNH,CPD,PADI4,CTBS,GMPR,DHFR,OTUB1,SLC4A4,HECW1,WIPI2,COPS7A,GAMT
signal transduction	GO:0007165	2.70E-08	5925	197	52	RSPO4,CRACR2A,SIGIRR,SIT1,BAMBI,GRB7,ZFP36L2,OPTN,MAST4,FHL2,HAP1,MEF2C,WNT7A,NR1D2,ERLEC1,RHBDF1,HASPIN,STAT5B,PPP1R1A,ITPR2,WWC3,TEAD4,STRAP,OR1E1,ARHGAP22,IGFBP3,PRKAR2A,RAPGEFL1,GRM7,PTGFR,EDA2R,IFT140,OR8H2,NTN1,TFAP2B,UCMA,NFATC4,NRG1,GUCY2D,RHOBTB1,AKAP3,ADRA1D,UBE3A,ARAP2,VANGL1,SRPX,PALM,ITFG2,LRRC39,MAPK13,ICAM1,BMP2

negative regulation of cellular process	GO:0048523	3.55E-08	4818	262	55	OCLN,PMP22,SIGIRR,KLF8,BAMBI,GRB7,ZFP36L2,OPTN,STXBP6,FHL2,HAP1,MEF2C,WNT7A,NR1D2,ERLEC1,RHBDF1,ABCC8,STAT5B,TWF2,ITPR2,TENT5C,WWC3,STRAP,IGFBP3,PRKAR2A,GRM7,PTGFR,GCK,NTN1,TXNDC5,HDAC9,TFAP2B,UCMA,NFATC4,REST,NRG1,ELAVL4,N4BP2L2,UBE3A,SRPX,ZNF12,PALM,ITFG2,ICAM1,BMP2,AGBL4,DKK4,MAGEB5,CBARP,GPRC5A,BAG2,SNX13,DHFR,OTUB1,HECW1
biosynthetic process	GO:0009058	1.26E-07	5956	279	64	SSX7,TECR,EIF3M,SIGIRR,KLF8,BAMBI,GRB7,ZFP36L2,DMRT2,EIF2S3B,FHL2,ZFHX2,CAVIN1,MEF2C,AGMAT,WNT7A,RPL19,NR1D2,GYG2,STAT5B,WWC3,TEAD4,STRAP,LCMT2,FUBP1,EDA2R,GCK,PDHB,ZNF208,HDAC9,TFAP2B,NFATC4,REST,NRG1,GUCY2D,RBM23,ZNF626,ELAVL4,B3GALT6,N4BP2L2,UBE3A,RRM2,TCEANC2,MRPL4,BHLHA9,ZNF12,BMP2,SLC26A1,TRAM2,HS3ST2,ALG1,ZAR1,ZNF726,MAGEB5,ZNF430,CCNH,HIVEP3,ELP2,DHFR,WIPI2,TAF4B,SLC5A7,GAMT,HNRNPA2B1
organic substance transport	GO:0071702	1.57E-07	2689	282	40	OCLN,SLC26A8,SLC6A13,SLC25A41,OPTN,PARP11,ACTB,CAVIN1,HAP1,SLCO1C1,ERLEC1,SCARB2,RHBDF1,CCHCR1,ABCC8,CLSTN1,SNX7,GRM7,C17ORF75,GCK,XKR4,TFAP2B,SNX32,REST,HERC2,PEX12,AQP7,SLC6A17,TBC1D26,SLC26A1,TRAM2,ARRDC2,ARL5C,SLC17A3,SNX13,SLC4A4,SLC5A7,SLC16A1,HNRNPA2B1,MICALL2
organic substance biosynthetic process	GO:1901576	1.93E-07	5869	279	63	SSX7,TECR,EIF3M,SIGIRR,KLF8,BAMBI,GRB7,ZFP36L2,DMRT2,EIF2S3B,FHL2,ZFHX2,CAVIN1,MEF2C,AGMAT,WNT7A,RPL19,NR1D2,GYG2,STAT5B,WWC3,TEAD4,STRAP,LCMT2,FUBP1,EDA2R,GCK,PDHB,ZNF208,HDAC9,TFAP2B,NFATC4,REST,NRG1,GUCY2D,RBM23,ZNF626,ELAVL4,B3GALT6,N4BP2L2,UBE3A,RRM2,TCEANC2,MRPL4,BHLHA9,ZNF12,BMP2,SLC26A1,HS3ST2,ALG1,ZAR1,ZNF726,MAGEB5,ZNF430,CCNH,HIVEP3,ELP2,DHFR,WIPI2,TAF4B,SLC5A7,GAMT,HNRNPA2B1
cellular nitrogen compound biosynthetic process	GO:0044271	2.03E-07	4734	255	52	SSX7,TECR,EIF3M,SIGIRR,KLF8,BAMBI,GRB7,ZFP36L2,DMRT2,EIF2S3B,FHL2,ZFHX2,CAVIN1,MEF2C,AGMAT,WNT7A,RPL19,NR1D2,STAT5B,WWC3,TEAD4,STRAP,FUBP1,EDA2R,PDHB,ZNF208,HDAC9,TFAP2B,NFATC4,REST,NRG1,GUCY2D,RBM23,ZNF626,ELAVL4,N4BP2L2,UBE3A,RRM2,TCEANC2,MRPL4,BHLHA9,ZNF12,BMP2,SLC26A1,ZAR1,ZNF726,MAGEB5,ZNF430,CCNH,HIVEP3,ELP2,DHFR
macromolecule biosynthetic process	GO:0009059	3.27E-07	4880	266	54	SSX7,EIF3M,SIGIRR,KLF8,BAMBI,GRB7,ZFP36L2,DMRT2,EIF2S3B,FHL2,ZFHX2,CAVIN1,MEF2C,WNT7A,RPL19,NR1D2,GYG2,STAT5B,WWC3,TEAD4,STRAP,FUBP1,EDA2R,GCK,ZNF208,HDAC9,TFAP2B,NFATC4,REST,NRG1,RBM23,ZNF626,ELAVL4,B3GALT6,N4BP2L2,UBE3A,RRM2,TCEANC2,MRPL4,BHLHA9,ZNF12,BMP2,HS3ST2,ALG1,ZAR1,ZNF726,MAGEB5,ZNF430,CCNH,HIVEP3,ELP2,DHFR,WIPI2,TAF4B

positive regulation of biological process	GO:0048518	3.72E-07	6094	199	51	RSPO4,CRACR2A,OCLN,SLC6A13,PLEKHM2,BAMBI,GRB7,ZFP36L2,OPTN,DMRT2,EIF2S3B,ACTB,CAVIN1,HAP1,MEF2C,WNT7A,SLCO1C1,NR1D2,SCARB2,ABCC8,STAT5B,TWF2,TENT5C,TEAD4,CLSTN1,IGFBP3,SNX7,FUBP1,PTGFR,EDA2R,GCK,LHFPL2,ZNF208,NTN1,HDAC9,TFAP2B,NFATC4,REST,NRG1,RBM23,MYLK2,ELAVL4,N4BP2L2,ADRA1D,UBE3A,SRPX,PALM,MAPK13,ICAM1,BMP2,AGBL4
system development	GO:0048731	3.79E-07	4784	234	49	CRACR2A,PMP22,BAMBI,TTLL7,ZFP36L2,DMRT2,ACTB,FHL2,ZFHX2,HAP1,MEF2C,WNT7A,NR1D2,SCARB2,ABCC8,ABCB5,STAT5B,TWF2,TEAD4,STRAP,CLSTN1,ARHGAP22,RAPGEFL1,GRM7,IFT140,NTN1,HDAC9,TFAP2B,UCMA,NFATC4,REST,NRG1,MYLK2,KIAA1217,ELAVL4,N4BP2L2,UBE3A,VANGL1,SLC6A17,PALM,ITFG2,BMP2,AGBL4,CIAO3,FLNB,DKK4,ZNF430,CCDC154,ADAMTS16
regulation of metabolic process	GO:0019222	4.23E-07	7219	262	68	SSX7,OCLN,SIGIRR,KLF8,MIR563,BAMBI,GRB7,ZFP36L2,OPTN,DMRT2,EIF2S3B,ACTB,FHL2,ZFHX2,HAP1,MEF2C,WNT7A,SLCO1C1,NR1D2,SCARB2,RHBDF1,ABCC8,STAT5B,TENT5C,WWC3,TEAD4,STRAP,IGFBP3,PRKAR2A,SNX7,FUBP1,PTGFR,EDA2R,GCK,ZNF208,HDAC9,TFAP2B,SNX32,NFATC4,REST,NRG1,RBM23,MYLK2,ZNF626,ELAVL4,N4BP2L2,UBE3A,BHLHA9,ZNF12,MAPK13,BMP2,CELF2,AGBL4,CIAO3,ZAR1,ZNF726,MAGEB5,ZNF430,GPRC5A,BAG2,CCNH,LPAR2,HIVEP3,ELP2,DHFR,OTUB1,SLC4A4,HECW1
regulation of developmental process	GO:0050793	7.42E-07	2421	199	30	BAMBI,ZFP36L2,DMRT2,ZFHX2,HAP1,MEF2C,WNT7A,NR1D2,ABCC8,STAT5B,TWF2,WWC3,TEAD4,CLSTN1,IGFBP3,NTN1,HDAC9,TFAP2B,UCMA,NFATC4,REST,NRG1,RHOBTB1,ELAVL4,N4BP2L2,UBE3A,VANGL1,PALM,BMP2,AGBL4
regulation of biological quality	GO:0065008	8.10E-07	3741	141	31	OCLN,SLC6A13,SIT1,BAMBI,ZFP36L2,EIF2S3B,ACTB,HAP1,MEF2C,WNT7A,SLCO1C1,NR1D2,ABCC8,ABCB5,STAT5B,TWF2,ITPR2,TENT5C,STRAP,CLSTN1,ARHGAP22,PTGFR,GCK,XKR4,NTN1,HDAC9,TFAP2B,NFATC4,REST,NRG1,RHOBTB1
regulation of cellular metabolic process	GO:0031323	8.60E-07	5921	260	59	SSX7,OCLN,SIGIRR,KLF8,BAMBI,GRB7,ZFP36L2,OPTN,DMRT2,EIF2S3B,ACTB,FHL2,ZFHX2,HAP1,MEF2C,WNT7A,SLCO1C1,NR1D2,SCARB2,RHBDF1,STAT5B,TENT5C,WWC3,TEAD4,STRAP,IGFBP3,PRKAR2A,SNX7,FUBP1,EDA2R,GCK,ZNF208,HDAC9,TFAP2B,SNX32,NFATC4,REST,NRG1,RBM23,ZNF626,ELAVL4,N4BP2L2,UBE3A,BHLHA9,ZNF12,BMP2,CELF2,AGBL4,ZNF726,MAGEB5,ZNF430,GPRC5A,BAG2,CCNH,HIVEP3,ELP2,DHFR,OTUB1,SLC4A4

cellular biosynthetic process	GO:0044249	8.99E-07	5794	279	61	SSX7,TECR,EIF3M,SIGIRR,KLF8,BAMBI,GRB7,ZFP36L2,DMRT2,EIF2S3B,FHL2,ZFHX2,CAVIN1,MEF2C,AGMAT,WNT7A,RPL19,NR1D2,GYG2,STAT5B,WWC3,TEAD4,STRAP,FUBP1,EDA2R,GCK,PDHB,ZNF208,HDAC9,TFAP2B,NFATC4,REST,NRG1,GUCY2D,RBM23,ZNF626,ELAVL4,B3GALT6,N4BP2L2,UBE3A,RRM2,TCEANC2,MRPL4,BHLHA9,ZNF12,BMP2,SLC26A1,ALG1,ZAR1,ZNF726,MAGEB5,ZNF430,CCNH,HIVEP3,ELP2,DHFR,WIPI2,TAF4B,SLC5A7,GAMT,HNRNPA2B1
macromolecule localization	GO:0033036	9.37E-07	3147	157	30	CENPA,OCLN,PLEKHM2,OPTN,PARP11,ACTB,CAVIN1,HAP1,WNT7A,SLCO1C1,ERLEC1,SCARB2,RHBDF1,CCHCR1,ABCC8,HASPIN,STAT5B,TWF2,CLSTN1,SNX7,C17ORF75,IFT140,GCK,XKR4,TFAP2B,SNX32,REST,AKAP3,HERC2,PEX12
regulation of nitrogen compound metabolic process	GO:0051171	2.01917E-06	5529	262	56	SSX7,OCLN,SIGIRR,KLF8,BAMBI,GRB7,ZFP36L2,DMRT2,EIF2S3B,ACTB,FHL2,ZFHX2,HAP1,MEF2C,WNT7A,SLCO1C1,NR1D2,RHBDF1,STAT5B,TENT5C,WWC3,TEAD4,STRAP,IGFBP3,PRKAR2A,FUBP1,EDA2R,GCK,ZNF208,HDAC9,TFAP2B,NFATC4,REST,NRG1,RBM23,ZNF626,ELAVL4,N4BP2L2,UBE3A,BHLHA9,ZNF12,BMP2,CELF2,AGBL4,ZNF726,MAGEB5,ZNF430,GPRC5A,BAG2,CCNH,HIVEP3,ELP2,DHFR,OTUB1,SLC4A4,HECW1
protein localization	GO:0008104	2.4525E-06	2700	211	32	CENPA,OCLN,PLEKHM2,OPTN,PARP11,ACTB,CAVIN1,HAP1,WNT7A,ERLEC1,SCARB2,RHBDF1,CCHCR1,ABCC8,HASPIN,TWF2,CLSTN1,SNX7,C17ORF75,IFT140,GCK,TFAP2B,SNX32,REST,AKAP3,HERC2,PEX12,PALM,TBC1D26,TRAM2,ARRDC2,ARL5C
regulation of macromolecule metabolic process	GO:0060255	2.80012E-06	6710	262	63	SSX7,OCLN,SIGIRR,KLF8,MIR563,BAMBI,GRB7,ZFP36L2,OPTN,DMRT2,EIF2S3B,ACTB,FHL2,ZFHX2,HAP1,MEF2C,WNT7A,NR1D2,SCARB2,RHBDF1,ABCC8,STAT5B,TENT5C,WWC3,TEAD4,STRAP,IGFBP3,PRKAR2A,FUBP1,PTGFR,EDA2R,GCK,ZNF208,HDAC9,TFAP2B,NFATC4,REST,NRG1,RBM23,MYLK2,ZNF626,ELAVL4,N4BP2L2,UBE3A,BHLHA9,ZNF12,MAPK13,BMP2,CELF2,AGBL4,CIAO3,ZAR1,ZNF726,MAGEB5,ZNF430,GPRC5A,BAG2,CCNH,HIVEP3,ELP2,DHFR,OTUB1,HECW1
regulation of cell communication	GO:0010646	4.24333E-06	3313	197	34	RSPO4,CRACR2A,SIGIRR,BAMBI,OPTN,FHL2,HAP1,MEF2C,WNT7A,RHBDF1,ABCC8,WWC3,STRAP,CLSTN1,ARHGAP22,IGFBP3,GRM7,EDA2R,IFT140,GCK,TFAP2B,UCMA,NFATC4,REST,NRG1,GUCY2D,ELAVL4,ADRA1D,UBE3A,SRPX,PALM,ITFG2,ICAM1,BMP2
regulation of signaling	GO:0023051	4.71957E-06	3327	197	34	RSPO4,CRACR2A,SIGIRR,BAMBI,OPTN,FHL2,HAP1,MEF2C,WNT7A,RHBDF1,ABCC8,WWC3,STRAP,CLSTN1,ARHGAP22,IGFBP3,GRM7,EDA2R,IFT140,GCK,TFAP2B,UCMA,NFATC4,REST,NRG1,GUCY2D,ELAVL4,ADRA1D,UBE3A,SRPX,PALM,ITFG2,ICAM1,BMP2

protein metabolic process	GO:0019538	5.3393E-06	5389	268	55	OCLN,EIF3M,RHBDL3,GRB7,TTLL7,ZFP36L2,MAST4,PARP11,EIF2S3B,ACTB,HAP1,MEF2C,WNT7A,RPL19,ERLEC1,RHBDF1,HASPIN,TTLL10,IGFBP3,PRKAR2A,TPGS1,ART4,HDAC9,REST,NRG1,GUCY2D,MYLK2,HERC2,DYRK4,PEX12,ELAVL4,B3GALT6,UBE3A,MRPL4,LRRC39,MAPK13,CELA2B,BMP2,AGBL4,FKBP2,ALG1,ZAR1,RNF170,ADAMTS16,GPRC5A,TPSAB1,BAG2,CCNH,CPD,PADI4,DHFR,OTUB1,HECW1,WIPI2,COPS7A
negative regulation of biological process	GO:0048519	5.48755E-06	5844	262	57	OCLN,PMP22,SIGIRR,KLF8,MIR563,BAMBI,GRB7,ZFP36L2,OPTN,STXBP6,FHL2,HAP1,MEF2C,WNT7A,NR1D2,ERLEC1,RHBDF1,ABCC8,STAT5B,TWF2,ITPR2,TENT5C,WWC3,STRAP,IGFBP3,PRKAR2A,GRM7,PTGFR,GCK,NTN1,TXNDC5,HDAC9,TFAP2B,UCMA,NFATC4,REST,NRG1,ELAVL4,N4BP2L2,UBE3A,SRPX,ZNF12,PALM,ITFG2,ICAM1,BMP2,AGBL4,DKK4,ZAR1,MAGEB5,CBARP,GPRC5A,BAG2,SNX13,DHFR,OTUB1,HECW1
regulation of primary metabolic process	GO:0080090	5.84672E-06	5694	262	56	SSX7,OCLN,SIGIRR,KLF8,BAMBI,GRB7,ZFP36L2,DMRT2,EIF2S3B,ACTB,FHL2,ZFHX2,HAP1,MEF2C,WNT7A,NR1D2,SCARB2,RHBDF1,STAT5B,TENT5C,WWC3,TEAD4,STRAP,IGFBP3,PRKAR2A,FUBP1,EDA2R,GCK,ZNF208,HDAC9,TFAP2B,NFATC4,REST,NRG1,RBM23,ZNF626,ELAVL4,N4BP2L2,UBE3A,BHLHA9,ZNF12,BMP2,CELF2,AGBL4,ZNF726,MAGEB5,ZNF430,GPRC5A,BAG2,CCNH,HIVEP3,ELP2,DHFR,OTUB1,SLC4A4,HECW1
cellular response to organic substance	GO:0071310	7.37536E-06	2385	209	29	SIGIRR,BAMBI,ZFP36L2,OPTN,ACTB,HAP1,MEF2C,WNT7A,NR1D2,ERLEC1,ABCC8,STAT5B,TWF2,ITPR2,STRAP,PTGFR,EDA2R,GCK,HDAC9,TFAP2B,REST,GBP6,ELAVL4,UBE3A,PALM,MAPK13,ICAM1,BMP2,FLNB
regulation of localization	GO:0032879	9.6337E-06	2701	144	25	CRACR2A,OCLN,PLEKHM2,GRB7,KCNQ5,STXBP6,ACTB,CAVIN1,HAP1,MEF2C,WNT7A,ERLEC1,RHBDF1,ABCC8,CATSPER4,ITPR2,IGFBP3,GRM7,GCK,NTN1,HDAC9,TFAP2B,REST,NRG1,MYLK2
cellular macromolecule metabolic process	GO:0044260	1.26829E-05	5746	279	58	OCLN,EIF3M,GRB7,TTLL7,ZFP36L2,OPTN,MAST4,PARP11,EIF2S3B,ACTB,MEF2C,WNT7A,RPL19,ERLEC1,GYG2,RHBDF1,HASPIN,PPP1R1A,TENT5C,TTLL10,LCMT2,IGFBP3,PRKAR2A,TPGS1,ART4,GCK,HDAC9,REST,NRG1,GUCY2D,MYLK2,HERC2,DYRK4,PEX12,ELAVL4,B3GALT6,UBE3A,RRM2,MRPL4,LRRC39,MAPK13,BMP2,AGBL4,FKBP2,ALG1,ZAR1,RNF170,GPRC5A,BAG2,CCNH,PADI4,DHFR,OTUB1,HECW1,WIPI2,COPS7A,MUS81,HNRNPA2B1
movement of cell or subcellular component	GO:0006928	1.37437E-05	2074	148	22	SLC26A8,BAMBI,GRB7,DNAAF5,ACTB,CAVIN1,HAP1,MEF2C,WNT7A,RHBDF1,ABCC8,CATSPER4,STAT5B,WWC3,IGFBP3,TPGS1,IFT140,NTN1,HDAC9,NRG1,MYLK2,SPAG17
nitrogen compound transport	GO:0071705	1.39056E-05	2278	211	28	SLC6A13,SLC25A41,OPTN,PARP11,ACTB,CAVIN1,HAP1,ERLEC1,SCARB2,RHBDF1,CCHCR1,ABCC8,CLSTN1,SNX7,GRM7,C17ORF75,GCK,TFAP2B,SNX32,REST,HERC2,PEX12,AQP7,SLC6A17,TBC1D26,TRAM2,ARRDC2,ARL5C

cellular localization	GO:0051641	1.4341E-05	3117	157	28	CRACR2A,CENPA,OCLN,SLC6A13,PLEKHM2,OPTN,STXBP6,ACTB,HAP1,MEF2C,WNT7A,ERLEC1,SCARB2,CCHCR1,HASPIN,TWF2,ITPR2,CLSTN1,TPGS1,SNX7,C17ORF75,IFT140,NTN1,SNX32,NRG1,MYLK2,HERC2,PEX12
response to endogenous stimulus	GO:0009719	1.61306E-05	1551	197	22	BAMBI,ZFP36L2,ACTB,FHL2,MEF2C,WNT7A,NR1D2,ABCC8,STAT5B,ITPR2,STRAP,PTGFR,GCK,HDAC9,TFAP2B,REST,ELAVL4,UBE3A,PALM,MAPK13,ICAM1,BMP2
positive regulation of cellular process	GO:0048522	1.67206E-05	5561	199	45	RSPO4,CRACR2A,OCLN,SLC6A13,PLEKHM2,BAMBI,GRB7,ZFP36L2,OPTN,DMRT2,EIF2S3B,CAVIN1,HAP1,MEF2C,WNT7A,SLCO1C1,NR1D2,SCARB2,ABCC8,STAT5B,TWF2,TEAD4,CLSTN1,IGFBP3,SNX7,PTGFR,EDA2R,GCK,ZNF208,NTN1,HDAC9,TFAP2B,NFATC4,REST,NRG1,RBM23,ELAVL4,N4BP2L2,ADRA1D,UBE3A,SRPX,PALM,ICAM1,BMP2,AGBL4
intracellular signal transduction	GO:0035556	1.74884E-05	2639	197	29	CRACR2A,ZFP36L2,OPTN,MAST4,FHL2,MEF2C,WNT7A,HASPIN,PPP1R1A,ITPR2,WWC3,TEAD4,ARHGAP22,IGFBP3,PRKAR2A,RAPGEFL1,PTGFR,EDA2R,NTN1,NFATC4,NRG1,GUCY2D,RHOBTB1,ADRA1D,UBE3A,ITFG2,MAPK13,ICAM1,BMP2
cellular response to chemical stimulus	GO:0070887	2.21178E-05	3008	197	31	SIGIRR,BAMBI,ZFP36L2,OPTN,ACTB,HAP1,MEF2C,WNT7A,NR1D2,ERLEC1,ABCC8,STAT5B,TWF2,ITPR2,STRAP,PTGFR,EDA2R,GCK,NTN1,HDAC9,TFAP2B,UGT2B28,NFATC4,REST,GBP6,ELAVL4,UBE3A,PALM,MAPK13,ICAM1,BMP2
transmembrane transport	GO:0055085	2.21995E-05	1531	277	26	CRACR2A,OCLN,SLC26A8,SLC6A13,SLC25A41,KCNQ5,ACTB,HAP1,MEF2C,SLCO1C1,ABCC8,ABCB5,CATSPER4,ITPR2,PEX12,AQP7,SLC6A17,SLC26A1,TRAM2,CBARP,CLCN4,SLC17A3,SLC4A4,HECW1,SLC5A7,SLC16A1
regulation of cell differentiation	GO:0045595	2.34405E-05	1541	136	18	BAMBI,ZFP36L2,ZFHX2,HAP1,MEF2C,WNT7A,NR1D2,ABCC8,STAT5B,TWF2,IGFBP3,NTN1,HDAC9,TFAP2B,UCMA,NFATC4,REST,NRG1
cellular response to endogenous stimulus	GO:0071495	2.43847E-05	1310	197	20	BAMBI,ZFP36L2,ACTB,MEF2C,WNT7A,NR1D2,STAT5B,ITPR2,STRAP,PTGFR,GCK,HDAC9,TFAP2B,REST,ELAVL4,UBE3A,PALM,MAPK13,ICAM1,BMP2
heterocycle biosynthetic process	GO:0018130	2.58596E-05	4024	255	43	SSX7,TECR,SIGIRR,KLF8,BAMBI,DMRT2,FHL2,ZFHX2,CAVIN1,MEF2C,WNT7A,NR1D2,STAT5B,WWC3,TEAD4,STRAP,FUBP1,EDA2R,PDHB,ZNF208,HDAC9,TFAP2B,NFATC4,REST,NRG1,GUCY2D,RBM23,ZNF626,N4BP2L2,UBE3A,RRM2,TCEANC2,BHLHA9,ZNF12,BMP2,SLC26A1,ZNF726,MAGEB5,ZNF430,CCNH,HIVEP3,ELP2,DHFR
aromatic compound biosynthetic process	GO:0019438	2.7979E-05	4035	255	43	SSX7,TECR,SIGIRR,KLF8,BAMBI,DMRT2,FHL2,ZFHX2,CAVIN1,MEF2C,WNT7A,NR1D2,STAT5B,WWC3,TEAD4,STRAP,FUBP1,EDA2R,PDHB,ZNF208,HDAC9,TFAP2B,NFATC4,REST,NRG1,GUCY2D,RBM23,ZNF626,N4BP2L2,UBE3A,RRM2,TCEANC2,BHLHA9,ZNF12,BMP2,SLC26A1,ZNF726,MAGEB5,ZNF430,CCNH,HIVEP3,ELP2,DHFR

cell development	GO:0048468	2.95533E-05	2079	209	26	PMP22,SLC26A8,ACTB,FHL2,HAP1,MEF2C,WNT7A,SCARB2,ABCC8,CATSPER4,TWF2,TPGS1,GRM7,IFT140,NTN1,HDAC9,NFATC4,REST,NRG1,FAM9B,ELAVL4,UBE3A,ICAM1,BMP2,AGBL4,FLNB
nucleobase-containing compound biosynthetic process	GO:0034654	3.25632E-05	3953	252	42	SSX7,TECR,SIGIRR,KLF8,BAMBI,DMRT2,FHL2,ZFHX2,CAVIN1,MEF2C,WNT7A,NR1D2,STAT5B,WWC3,TEAD4,STRAP,FUBP1,EDA2R,PDHB,ZNF208,HDAC9,TFAP2B,NFATC4,REST,NRG1,GUCY2D,RBM23,ZNF626,N4BP2L2,UBE3A,RRM2,TCEANC2,BHLHA9,ZNF12,BMP2,SLC26A1,ZNF726,MAGEB5,ZNF430,CCNH,HIVEP3,ELP2
cellular protein metabolic process	GO:0044267	4.64086E-05	4796	268	49	OCLN,EIF3M,GRB7,TTLL7,ZFP36L2,MAST4,PARP11,EIF2S3B,ACTB,MEF2C,WNT7A,RPL19,ERLEC1,RHBDF1,HASPIN,TTLL10,IGFBP3,PRKAR2A,TPGS1,ART4,HDAC9,REST,NRG1,GUCY2D,MYLK2,HERC2,DYRK4,PEX12,ELAVL4,B3GALT6,UBE3A,MRPL4,LRRC39,MAPK13,BMP2,AGBL4,FKBP2,ALG1,ZAR1,RNF170,GPRC5A,BAG2,CCNH,PADI4,DHFR,OTUB1,HECW1,WIPI2,COPS7A
regulation of gene expression	GO:0010468	4.83306E-05	5388	255	51	SSX7,OCLN,SIGIRR,KLF8,MIR563,BAMBI,GRB7,ZFP36L2,DMRT2,EIF2S3B,FHL2,ZFHX2,MEF2C,WNT7A,NR1D2,ABCC8,STAT5B,TENT5C,WWC3,TEAD4,STRAP,FUBP1,PTGFR,EDA2R,ZNF208,HDAC9,TFAP2B,NFATC4,REST,NRG1,RBM23,MYLK2,ZNF626,ELAVL4,N4BP2L2,UBE3A,BHLHA9,ZNF12,MAPK13,BMP2,CELF2,CIAO3,ZAR1,ZNF726,MAGEB5,ZNF430,BAG2,CCNH,HIVEP3,ELP2,DHFR
secretion	GO:0046903	4.83323E-05	924	136	14	STXBP6,CAVIN1,HAP1,MEF2C,WNT7A,RHBDF1,ABCC8,STAT5B,TMEM167B,GRM7,GCK,TFAP2B,REST,NRG1
nervous system development	GO:0007399	5.92311E-05	2428	199	27	PMP22,TTLL7,ACTB,ZFHX2,HAP1,MEF2C,WNT7A,SCARB2,ABCC8,TWF2,STRAP,CLSTN1,RAPGEFL1,GRM7,IFT140,NTN1,HDAC9,TFAP2B,NFATC4,REST,NRG1,ELAVL4,UBE3A,SLC6A17,PALM,BMP2,AGBL4
generation of neurons	GO:0048699	6.69249E-05	1519	199	21	PMP22,ACTB,ZFHX2,HAP1,MEF2C,WNT7A,SCARB2,ABCC8,TWF2,STRAP,GRM7,IFT140,NTN1,HDAC9,NFATC4,REST,NRG1,ELAVL4,UBE3A,BMP2,AGBL4
plasma membrane bounded cell projection organization	GO:0120036	6.74907E-05	1479	282	25	OCLN,PMP22,DNAAF5,ACTB,HAP1,MEF2C,WNT7A,SCARB2,EMP1,TWF2,TPGS1,GRM7,IFT140,NTN1,NFATC4,SPAG17,ELAVL4,UBE3A,PALM,ICAM1,ADAMTS16,CLCN4,DHFR,HECW1,MICALL2
organic cyclic compound biosynthetic process	GO:1901362	7.72132E-05	4181	255	43	SSX7,TECR,SIGIRR,KLF8,BAMBI,DMRT2,FHL2,ZFHX2,CAVIN1,MEF2C,WNT7A,NR1D2,STAT5B,WWC3,TEAD4,STRAP,FUBP1,EDA2R,PDHB,ZNF208,HDAC9,TFAP2B,NFATC4,REST,NRG1,GUCY2D,RBM23,ZNF626,N4BP2L2,UBE3A,RRM2,TCEANC2,BHLHA9,ZNF12,BMP2,SLC26A1,ZNF726,MAGEB5,ZNF430,CCNH,HIVEP3,ELP2,DHFR

regulation of nucleobase-containing compound metabolic process	GO:0019219	7.73359E-05	3945	260	42	SSX7,SIGIRR,KLF8,BAMBI,ZFP36L2,DMRT2,FHL2,ZFHX2,MEF2C,WNT7A,NR1D2,STAT5B,TENT5C,WWC3,TEAD4,STRAP,FUBP1,EDA2R,GCK,ZNF208,HDAC9,TFAP2B,NFATC4,REST,NRG1,RBM23,ZNF626,ELAVL4,N4BP2L2,UBE3A,BHLHA9,ZNF12,BMP2,CELF2,ZNF726,MAGEB5,ZNF430,CCNH,HIVEP3,ELP2,OTUB1,SLC4A4
response to organic substance	GO:0010033	8.20835E-05	3009	197	30	CRACR2A,SIGIRR,BAMBI,ZFP36L2,OPTN,ACTB,FHL2,HAP1,MEF2C,WNT7A,NR1D2,ERLEC1,ABCC8,STAT5B,TWF2,ITPR2,STRAP,PTGFR,EDA2R,GCK,HDAC9,TFAP2B,REST,GBP6,ELAVL4,UBE3A,PALM,MAPK13,ICAM1,BMP2
plasma membrane bounded cell projection assembly	GO:0120031	9.54353E-05	548	239	14	OCLN,PMP22,DNAAF5,HAP1,EMP1,TWF2,TPGS1,IFT140,NTN1,SPAG17,PALM,ICAM1,ADAMTS16,CLCN4
cellular component organization	GO:0016043	9.65338E-05	7793	287	70	CRACR2A,CENPA,OCLN,PMP22,EIF3M,COA5,PLEKHM2,GRB7,TTLL7,RNU6-1254P,OPTN,MAST4,STXBP6,PARP11,DNAAF5,EIF2S3B,ACTB,HAP1,MEF2C,WNT7A,SCARB2,ABCC8,HASPIN,EMP1,TWF2,STRAP,CLSTN1,ARHGAP22,PRPF39,IGFBP3,TPGS1,SNX7,GRM7,C17ORF75,IFT140,XKR4,NTN1,HDAC9,NFATC4,REST,NRG1,RHOBTB1,SPAG17,PEX12,ELAVL4,UBE3A,RRM2,SRPX,PALM,ICAM1,BMP2,CELF2,TRAM2,CIAO3,FLNB,ADAMTS16,TPSAB1,CLCN4,EVPLL,PADI4,DHFR,NDUFB10,HECW1,WIPI2,COPS7A,SLC16A1,MUS81,HNRNPA2B1,MICALL2,XPA
regulation of macromolecule biosynthetic process	GO:0010556	0.00011208	3926	255	41	SSX7,SIGIRR,KLF8,BAMBI,GRB7,ZFP36L2,DMRT2,EIF2S3B,FHL2,ZFHX2,MEF2C,WNT7A,NR1D2,STAT5B,WWC3,TEAD4,STRAP,FUBP1,EDA2R,GCK,ZNF208,HDAC9,TFAP2B,NFATC4,REST,NRG1,RBM23,ZNF626,ELAVL4,N4BP2L2,UBE3A,BHLHA9,ZNF12,BMP2,ZNF726,MAGEB5,ZNF430,CCNH,HIVEP3,ELP2,DHFR
cell projection organization	GO:0030030	0.000114097	1521	262	24	OCLN,PMP22,DNAAF5,ACTB,HAP1,MEF2C,WNT7A,SCARB2,EMP1,TWF2,TPGS1,GRM7,IFT140,NTN1,NFATC4,SPAG17,ELAVL4,UBE3A,PALM,ICAM1,ADAMTS16,CLCN4,DHFR,HECW1
cell projection assembly	GO:0030031	0.000130057	562	239	14	OCLN,PMP22,DNAAF5,HAP1,EMP1,TWF2,TPGS1,IFT140,NTN1,SPAG17,PALM,ICAM1,ADAMTS16,CLCN4
regulation of response to stimulus	GO:0048583	0.000133739	3841	262	41	RSPO4,CRACR2A,OCLN,SIGIRR,BAMBI,OPTN,FHL2,HAP1,MEF2C,WNT7A,NR1D2,RHBDF1,ABCC8,STAT5B,WWC3,STRAP,ARHGAP22,IGFBP3,EDA2R,IFT140,TFAP2B,UCMA,NFATC4,REST,NRG1,GUCY2D,ADRA1D,UBE3A,SRPX,PALM,ITFG2,MAPK13,ICAM1,BMP2,DKK4,GPRC5A,SNX13,ELP2,DHFR,OTUB1,HECW1
regulation of RNA metabolic process	GO:0051252	0.000142323	3700	252	39	SSX7,SIGIRR,KLF8,BAMBI,ZFP36L2,DMRT2,FHL2,ZFHX2,MEF2C,WNT7A,NR1D2,STAT5B,TENT5C,WWC3,TEAD4,STRAP,FUBP1,EDA2R,ZNF208,HDAC9,TFAP2B,NFATC4,REST,NRG1,RBM23,ZNF626,ELAVL4,N4BP2L2,UBE3A,BHLHA9,ZNF12,BMP2,CELF2,ZNF726,MAGEB5,ZNF430,CCNH,HIVEP3,ELP2

response to chemical	GO:0042221	0.000144332	4316	136	29	CRACR2A,SIGIRR,BAMBI,ZFP36L2,OPTN,ACTB,FHL2,HAP1,MEF2C,WNT7A,NR1D2,ERLEC1,ABCC8,STAT5B,TWF2,ITPR2,STRAP,OR1E1,PTGFR,EDA2R,GCK,OR8H2,NTN1,HDAC9,TFAP2B,UGT2B28,NFATC4,REST,NRG1
regulation of plasma membrane bounded cell projection organization	GO:0120035	0.000203518	618	262	15	OCLN,PMP22,HAP1,WNT7A,SCARB2,TWF2,IFT140,NTN1,NFATC4,ELAVL4,UBE3A,PALM,ICAM1,ADAMTS16,HECW1
tissue development	GO:0009888	0.000220913	1912	250	26	BAMBI,DMRT2,ACTB,FHL2,MEF2C,WNT7A,NR1D2,EMP1,STAT5B,EDA2R,IFT140,NTN1,HDAC9,TFAP2B,NFATC4,NRG1,MYLK2,VANGL1,ICAM1,BMP2,FLNB,DKK4,CCDC154,ADAMTS16,EVPLL,HIVEP3
synapse organization	GO:0050808	0.000230214	422	190	11	ACTB,MEF2C,WNT7A,CLSTN1,ARHGAP22,NTN1,NFATC4,REST,NRG1,UBE3A,PALM
neurogenesis	GO:0022008	0.000238391	1639	199	21	PMP22,ACTB,ZFHX2,HAP1,MEF2C,WNT7A,SCARB2,ABCC8,TWF2,STRAP,GRM7,IFT140,NTN1,HDAC9,NFATC4,REST,NRG1,ELAVL4,UBE3A,BMP2,AGBL4
cellular component organization or biogenesis	GO:0071840	0.000261796	7994	287	70	CRACR2A,CENPA,OCLN,PMP22,EIF3M,COA5,PLEKHM2,GRB7,TTLL7,RNU6-1254P,OPTN,MAST4,STXBP6,PARP11,DNAAF5,EIF2S3B,ACTB,HAP1,MEF2C,WNT7A,SCARB2,ABCC8,HASPIN,EMP1,TWF2,STRAP,CLSTN1,ARHGAP22,PRPF39,IGFBP3,TPGS1,SNX7,GRM7,C17ORF75,IFT140,XKR4,NTN1,HDAC9,NFATC4,REST,NRG1,RHOBTB1,SPAG17,PEX12,ELAVL4,UBE3A,RRM2,SRPX,PALM,ICAM1,BMP2,CELF2,TRAM2,CIAO3,FLNB,ADAMTS16,TPSAB1,CLCN4,EVPLL,PADI4,DHFR,NDUFB10,HECW1,WIPI2,COPS7A,SLC16A1,MUS81,HNRNPA2B1,MICALL2,XPA
regulation of secretion	GO:0051046	0.000279199	605	136	11	STXBP6,HAP1,MEF2C,WNT7A,RHBDF1,ABCC8,GRM7,GCK,TFAP2B,REST,NRG1
macromolecule modification	GO:0043412	0.000280248	3856	268	41	OCLN,ABHD12B,TTLL7,MAST4,PARP11,ACTB,MEF2C,HASPIN,TTLL10,LCMT2,IGFBP3,PRKAR2A,TPGS1,ART4,HDAC9,REST,NRG1,GUCY2D,MYLK2,HERC2,DYRK4,PEX12,B3GALT6,UBE3A,ANKRD16,LRRC39,MAPK13,BMP2,AGBL4,FKBP2,ALG1,RNF170,GPRC5A,BAG2,CCNH,PADI4,ELP2,OTUB1,HECW1,WIPI2,COPS7A
neuron differentiation	GO:0030182	0.000281789	1364	199	19	PMP22,ACTB,ZFHX2,MEF2C,WNT7A,SCARB2,TWF2,STRAP,GRM7,IFT140,NTN1,HDAC9,NFATC4,REST,NRG1,ELAVL4,UBE3A,BMP2,AGBL4
regulation of cell projection organization	GO:0031344	0.000282364	634	262	15	OCLN,PMP22,HAP1,WNT7A,SCARB2,TWF2,IFT140,NTN1,NFATC4,ELAVL4,UBE3A,PALM,ICAM1,ADAMTS16,HECW1
regulation of transport	GO:0051049	0.000295857	1719	144	18	CRACR2A,OCLN,KCNQ5,STXBP6,ACTB,HAP1,MEF2C,WNT7A,ERLEC1,RHBDF1,ABCC8,CATSPER4,GRM7,GCK,TFAP2B,REST,NRG1,MYLK2

regulation of cellular biosynthetic process	GO:0031326	0.000316862	4081	255	41	SSX7,SIGIRR,KLF8,BAMBI,GRB7,ZFP36L2,DMRT2,EIF2S3B,FHL2,ZFHX2,MEF2C,WNT7A,NR1D2,STAT5B,WWC3,TEAD4,STRAP,FUBP1,EDA2R,GCK,ZNF208,HDAC9,TFAP2B,NFATC4,REST,NRG1,RBM23,ZNF626,ELAVL4,N4BP2L2,UBE3A,BHLHA9,ZNF12,BMP2,ZNF726,MAGEB5,ZNF430,CCNH,HIVEP3,ELP2,DHFR
localization of cell	GO:0051674	0.000354489	1645	136	17	SLC26A8,BAMBI,GRB7,ACTB,CAVIN1,MEF2C,WNT7A,RHBDF1,ABCC8,CATSPER4,STAT5B,WWC3,IGFBP3,TPGS1,NTN1,HDAC9,NRG1
cell motility	GO:0048870	0.000354489	1645	136	17	SLC26A8,BAMBI,GRB7,ACTB,CAVIN1,MEF2C,WNT7A,RHBDF1,ABCC8,CATSPER4,STAT5B,WWC3,IGFBP3,TPGS1,NTN1,HDAC9,NRG1
vesicle-mediated transport	GO:0016192	0.000397186	1597	141	17	CRACR2A,OPTN,STXBP6,ACTB,HAP1,WNT7A,SCARB2,TMEM167B,CLSTN1,SNX7,C17ORF75,XKR4,TXNDC5,SNX32,REST,NRG1,RHOBTB1
secretion by cell	GO:0032940	0.000427699	784	135	12	STXBP6,CAVIN1,HAP1,MEF2C,WNT7A,RHBDF1,ABCC8,TMEM167B,GRM7,GCK,TFAP2B,REST
transcription, DNA-templated	GO:0006351	0.000431349	3551	252	37	SSX7,SIGIRR,KLF8,BAMBI,DMRT2,FHL2,ZFHX2,CAVIN1,MEF2C,WNT7A,NR1D2,STAT5B,WWC3,TEAD4,STRAP,FUBP1,EDA2R,ZNF208,HDAC9,TFAP2B,NFATC4,REST,NRG1,RBM23,ZNF626,N4BP2L2,UBE3A,TCEANC2,BHLHA9,ZNF12,BMP2,ZNF726,MAGEB5,ZNF430,CCNH,HIVEP3,ELP2
nucleic acid-templated transcription	GO:0097659	0.000434338	3552	252	37	SSX7,SIGIRR,KLF8,BAMBI,DMRT2,FHL2,ZFHX2,CAVIN1,MEF2C,WNT7A,NR1D2,STAT5B,WWC3,TEAD4,STRAP,FUBP1,EDA2R,ZNF208,HDAC9,TFAP2B,NFATC4,REST,NRG1,RBM23,ZNF626,N4BP2L2,UBE3A,TCEANC2,BHLHA9,ZNF12,BMP2,ZNF726,MAGEB5,ZNF430,CCNH,HIVEP3,ELP2
regulation of biosynthetic process	GO:0009889	0.000466002	4141	255	41	SSX7,SIGIRR,KLF8,BAMBI,GRB7,ZFP36L2,DMRT2,EIF2S3B,FHL2,ZFHX2,MEF2C,WNT7A,NR1D2,STAT5B,WWC3,TEAD4,STRAP,FUBP1,EDA2R,GCK,ZNF208,HDAC9,TFAP2B,NFATC4,REST,NRG1,RBM23,ZNF626,ELAVL4,N4BP2L2,UBE3A,BHLHA9,ZNF12,BMP2,ZNF726,MAGEB5,ZNF430,CCNH,HIVEP3,ELP2,DHFR
RNA biosynthetic process	GO:0032774	0.000484878	3568	252	37	SSX7,SIGIRR,KLF8,BAMBI,DMRT2,FHL2,ZFHX2,CAVIN1,MEF2C,WNT7A,NR1D2,STAT5B,WWC3,TEAD4,STRAP,FUBP1,EDA2R,ZNF208,HDAC9,TFAP2B,NFATC4,REST,NRG1,RBM23,ZNF626,N4BP2L2,UBE3A,TCEANC2,BHLHA9,ZNF12,BMP2,ZNF726,MAGEB5,ZNF430,CCNH,HIVEP3,ELP2
regulation of signal transduction	GO:0009966	0.000579452	2929	197	28	RSPO4,CRACR2A,SIGIRR,BAMBI,OPTN,FHL2,HAP1,MEF2C,WNT7A,RHBDF1,WWC3,STRAP,ARHGAP22,IGFBP3,EDA2R,IFT140,TFAP2B,UCMA,NFATC4,NRG1,GUCY2D,ADRA1D,UBE3A,SRPX,PALM,ITFG2,ICAM1,BMP2
regulation of molecular function	GO:0065009	0.000606371	3017	262	34	CRACR2A,SIGIRR,BAMBI,ACTB,HAP1,MEF2C,SCARB2,ABCC8,PPP1R1A,DENND2C,ARHGAP22,IGFBP3,PRKAR2A,RAPGEFL1,GRM7,EDA2R,HDAC9,NFATC4,REST,NRG1,HERC2,ARAP2,PALM,TBC1D26,BMP2,DKK4,CBARP,GPRC5A,BAG2,CCNH,LPAR2,SNX13,DHFR,HECW1
cell junction organization	GO:0034330	0.000626927	687	190	13	OCLN,ACTB,MEF2C,WNT7A,ABCC8,CLSTN1,ARHGAP22,NTN1,NFATC4,REST,NRG1,UBE3A,PALM

regulation of cellular component organization	GO:0051128	0.000640484	2246	197	24	OCLN,PMP22,PLEKHM2,STXBP6,HAP1,MEF2C,WNT7A,SCARB2,ABCC8,TWF2,CLSTN1,ARHGAP22,IGFBP3,SNX7,IFT140,NTN1,NFATC4,NRG1,RHOBTB1,ELAVL4,UBE3A,PALM,ICAM1,BMP2
response to stress	GO:0006950	0.000771848	3780	263	39	CRACR2A,OCLN,SIGIRR,PLEKHM2,ZFP36L2,OPTN,ACTB,MEF2C,WNT7A,NR1D2,ERLEC1,ABCC8,STAT5B,ITPR2,PTGFR,EDA2R,HDAC9,NFATC4,REST,NRG1,GBP6,HERC2,ELAVL4,UBE3A,DEFB132,SRPX,ITFG2,MAPK13,BMP2,AGBL4,CIAO3,FLNB,TPSAB1,EVPLL,PADI4,GMPR,DHFR,OTUB1,WIPI2
cell-cell signaling	GO:0007267	0.000775663	1646	177	19	RSPO4,PMP22,BAMBI,HAP1,MEF2C,WNT7A,ABCC8,CLSTN1,TPGS1,GRM7,GCK,TFAP2B,NFATC4,REST,NRG1,MYLK2,ELAVL4,ADRA1D,VANGL1
export from cell	GO:0140352	0.000816952	840	277	17	STXBP6,CAVIN1,HAP1,MEF2C,WNT7A,RHBDF1,ABCC8,TMEM167B,GRM7,GCK,TFAP2B,REST,BMP2,CBARP,SLC17A3,SLC4A4,SLC16A1
regulation of secretion by cell	GO:1903530	0.000948285	547	135	10	STXBP6,HAP1,MEF2C,WNT7A,RHBDF1,ABCC8,GRM7,GCK,TFAP2B,REST
protein transport	GO:0015031	0.00095295	1829	211	22	OPTN,PARP11,CAVIN1,HAP1,ERLEC1,SCARB2,RHBDF1,CCHCR1,ABCC8,CLSTN1,SNX7,C17ORF75,GCK,TFAP2B,SNX32,REST,HERC2,PEX12,TBC1D26,TRAM2,ARRDC2,ARL5C
anatomical structure morphogenesis	GO:0009653	0.001124175	2685	234	29	PMP22,BAMBI,DMRT2,ACTB,FHL2,MEF2C,WNT7A,ABCC8,TWF2,TEAD4,ARHGAP22,IFT140,NTN1,FBN3,HDAC9,TFAP2B,NFATC4,NRG1,RHOBTB1,MYLK2,ELAVL4,UBE3A,VANGL1,PALM,BMP2,FLNB,DKK4,CCDC154,ADAMTS16
animal organ development	GO:0048513	0.001173945	3494	234	34	CRACR2A,BAMBI,ZFP36L2,ACTB,FHL2,ZFHX2,HAP1,MEF2C,WNT7A,NR1D2,ABCB5,STAT5B,TEAD4,IFT140,NTN1,HDAC9,TFAP2B,NFATC4,REST,NRG1,MYLK2,ELAVL4,N4BP2L2,UBE3A,VANGL1,SLC6A17,ITFG2,BMP2,CIAO3,FLNB,DKK4,ZNF430,CCDC154,ADAMTS16
enzyme linked receptor protein signaling pathway	GO:0007167	0.001259538	951	147	13	BAMBI,GRB7,HAP1,RHBDF1,STAT5B,STRAP,IGFBP3,TFAP2B,UCMA,NFATC4,NRG1,GUCY2D,AKAP3
regulation of transcription, DNA-templated	GO:0006355	0.001380279	3412	252	35	SSX7,SIGIRR,KLF8,BAMBI,DMRT2,FHL2,ZFHX2,MEF2C,WNT7A,NR1D2,STAT5B,WWC3,TEAD4,STRAP,FUBP1,EDA2R,ZNF208,HDAC9,TFAP2B,NFATC4,REST,NRG1,RBM23,ZNF626,N4BP2L2,UBE3A,BHLHA9,ZNF12,BMP2,ZNF726,MAGEB5,ZNF430,CCNH,HIVEP3,ELP2
regulation of nucleic acid-templated transcription	GO:1903506	0.001389595	3413	252	35	SSX7,SIGIRR,KLF8,BAMBI,DMRT2,FHL2,ZFHX2,MEF2C,WNT7A,NR1D2,STAT5B,WWC3,TEAD4,STRAP,FUBP1,EDA2R,ZNF208,HDAC9,TFAP2B,NFATC4,REST,NRG1,RBM23,ZNF626,N4BP2L2,UBE3A,BHLHA9,ZNF12,BMP2,ZNF726,MAGEB5,ZNF430,CCNH,HIVEP3,ELP2

regulation of RNA biosynthetic process	GO:2001141	0.001437059	3418	252	35	SSX7,SIGIRR,KLF8,BAMBI,DMRT2,FHL2,ZFHX2,MEF2C,WNT7A,NR1D2,STAT5B,WWC3,TEAD4,STRAP,FUBP1,EDA2R,ZNF208,HDAC9,TFAP2B,NFATC4,REST,NRG1,RBM23,ZNF626,N4BP2L2,UBE3A,BHLHA9,ZNF12,BMP2,ZNF726,MAGEB5,ZNF430,CCNH,HIVEP3,ELP2
locomotion	GO:0040011	0.001450734	1821	136	17	SLC26A8,BAMBI,GRB7,ACTB,CAVIN1,MEF2C,WNT7A,RHBDF1,ABCC8,CATSPER4,STAT5B,WWC3,IGFBP3,TPGS1,NTN1,HDAC9,NRG1
negative regulation of cellular metabolic process	GO:0031324	0.001486002	2493	136	20	OCLN,KLF8,GRB7,ZFP36L2,OPTN,FHL2,HAP1,MEF2C,NR1D2,TENT5C,WWC3,STRAP,IGFBP3,PRKAR2A,GCK,HDAC9,TFAP2B,NFATC4,REST,NRG1
cell death	GO:0008219	0.001674276	2057	136	18	PMP22,SLC6A13,OPTN,FHL2,MEF2C,WNT7A,EMP1,STAT5B,IGFBP3,PTGFR,EDA2R,XKR4,NTN1,TXNDC5,TFAP2B,NFATC4,REST,NRG1
regulation of transmembrane transport	GO:0034762	0.001692005	561	80	8	CRACR2A,OCLN,KCNQ5,ACTB,HAP1,MEF2C,ABCC8,CATSPER4
establishment of protein localization	GO:0045184	0.002560109	1942	211	22	OPTN,PARP11,CAVIN1,HAP1,ERLEC1,SCARB2,RHBDF1,CCHCR1,ABCC8,CLSTN1,SNX7,C17ORF75,GCK,TFAP2B,SNX32,REST,HERC2,PEX12,TBC1D26,TRAM2,ARRDC2,ARL5C
multi-organism reproductive process	GO:0044703	0.003007686	1054	167	14	SLC26A8,PARP11,ABCC8,CATSPER4,STAT5B,TEAD4,PRKAR2A,TPGS1,PTGFR,LHFPL2,FAM9B,AKAP3,HERC2,UBE3A
regulation of cellular component biogenesis	GO:0044087	0.003101391	940	136	12	OCLN,STXBP6,HAP1,MEF2C,WNT7A,TWF2,CLSTN1,SNX7,IFT140,NTN1,REST,NRG1
positive regulation of metabolic process	GO:0009893	0.003365421	3678	144	25	OCLN,BAMBI,ZFP36L2,OPTN,DMRT2,EIF2S3B,MEF2C,WNT7A,SLCO1C1,NR1D2,ABCC8,STAT5B,TENT5C,TEAD4,SNX7,FUBP1,PTGFR,GCK,ZNF208,TFAP2B,NFATC4,REST,NRG1,RBM23,MYLK2
cellular protein modification process	GO:0006464	0.003655776	3642	268	37	OCLN,TTLL7,MAST4,PARP11,ACTB,MEF2C,HASPIN,TTLL10,IGFBP3,PRKAR2A,TPGS1,ART4,HDAC9,REST,NRG1,GUCY2D,MYLK2,HERC2,DYRK4,PEX12,B3GALT6,UBE3A,LRRC39,MAPK13,BMP2,AGBL4,FKBP2,ALG1,RNF170,GPRC5A,BAG2,CCNH,PADI4,OTUB1,HECW1,WIPI2,COPS7A
protein modification process	GO:0036211	0.003655776	3642	268	37	OCLN,TTLL7,MAST4,PARP11,ACTB,MEF2C,HASPIN,TTLL10,IGFBP3,PRKAR2A,TPGS1,ART4,HDAC9,REST,NRG1,GUCY2D,MYLK2,HERC2,DYRK4,PEX12,B3GALT6,UBE3A,LRRC39,MAPK13,BMP2,AGBL4,FKBP2,ALG1,RNF170,GPRC5A,BAG2,CCNH,PADI4,OTUB1,HECW1,WIPI2,COPS7A
cellular response to growth factor stimulus	GO:0071363	0.00366823	654	197	12	BAMBI,ZFP36L2,HAP1,MEF2C,WNT7A,STAT5B,TWF2,STRAP,TFAP2B,ELAVL4,UBE3A,BMP2
muscle structure development	GO:0061061	0.003675683	616	209	12	FHL2,MEF2C,NR1D2,TEAD4,IGFBP3,HDAC9,NFATC4,REST,NRG1,MYLK2,BMP2,FLNB

negative regulation of secretion	GO:0051048	0.003817031	160	136	6	STXBP6,RHBDF1,ABCC8,GRM7,REST,NRG1
negative regulation of cell communication	GO:0010648	0.00390941	1347	262	20	SIGIRR,BAMBI,OPTN,FHL2,ABCC8,WWC3,STRAP,IGFBP3,UCMA,NFATC4,REST,NRG1,PALM,ITFG2,ICAM1,BMP2,DKK4,GPRC5A,SNX13,HECW1
negative regulation of signaling	GO:0023057	0.004042488	1350	262	20	SIGIRR,BAMBI,OPTN,FHL2,ABCC8,WWC3,STRAP,IGFBP3,UCMA,NFATC4,REST,NRG1,PALM,ITFG2,ICAM1,BMP2,DKK4,GPRC5A,SNX13,HECW1
regulation of synapse organization	GO:0050807	0.004303813	209	167	7	MEF2C,WNT7A,CLSTN1,ARHGAP22,NTN1,NFATC4,UBE3A
regulation of transcription by RNA polymerase II	GO:0006357	0.004894756	2529	252	28	KLF8,DMRT2,FHL2,ZFHX2,MEF2C,WNT7A,NR1D2,STAT5B,WWC3,TEAD4,STRAP,ZNF208,HDAC9,TFAP2B,NFATC4,REST,ZNF626,N4BP2L2,UBE3A,BHLHA9,ZNF12,BMP2,ZNF726,MAGEB5,ZNF430,CCNH,HIVEP3,ELP2
negative regulation of nitrogen compound metabolic process	GO:0051172	0.005082717	2330	258	27	OCLN,KLF8,GRB7,ZFP36L2,FHL2,HAP1,MEF2C,NR1D2,TENT5C,WWC3,STRAP,IGFBP3,PRKAR2A,HDAC9,TFAP2B,NFATC4,REST,NRG1,ELAVL4,N4BP2L2,ZNF12,BMP2,MAGEB5,GPRC5A,BAG2,DHFR,OTUB1
regulation of synapse structure or activity	GO:0050803	0.005190748	215	167	7	MEF2C,WNT7A,CLSTN1,ARHGAP22,NTN1,NFATC4,UBE3A
response to growth factor	GO:0070848	0.005552797	681	197	12	BAMBI,ZFP36L2,HAP1,MEF2C,WNT7A,STAT5B,TWF2,STRAP,TFAP2B,ELAVL4,UBE3A,BMP2
ion transport	GO:0006811	0.005761929	1544	277	22	CRACR2A,SLC26A8,SLC6A13,SLC25A41,KCNQ5,HAP1,MEF2C,SLCO1C1,ABCC8,CATSPER4,ITPR2,GRM7,GCK,SLC6A17,SLC26A1,CBARP,CLCN4,SLC17A3,SLC4A4,HECW1,SLC5A7,SLC16A1
response to hormone	GO:0009725	0.006426898	841	135	11	ZFP36L2,FHL2,MEF2C,WNT7A,NR1D2,ABCC8,STAT5B,PTGFR,GCK,HDAC9,REST
establishment of localization in cell	GO:0051649	0.007304024	2405	211	24	CRACR2A,CENPA,SLC6A13,ACTB,HAP1,MEF2C,WNT7A,ERLEC1,SCARB2,CCHCR1,ITPR2,SNX7,C17ORF75,IFT140,NTN1,SNX32,NRG1,MYLK2,HERC2,PEX12,TBC1D26,AGBL4,TRAM2,ARL5C
negative regulation of developmental process	GO:0051093	0.007831199	893	213	14	BAMBI,ZFP36L2,WNT7A,ABCC8,STAT5B,WWC3,NTN1,UCMA,NFATC4,REST,N4BP2L2,UBE3A,BMP2,DKK4
regulation of multicellular organismal process	GO:0051239	0.00800554	2623	144	20	SIGIRR,BAMBI,ZFP36L2,DMRT2,ZFHX2,HAP1,MEF2C,WNT7A,ABCC8,STAT5B,TWF2,WWC3,TEAD4,CLSTN1,NTN1,HDAC9,NFATC4,REST,NRG1,MYLK2
peptidyl-glutamic acid modification	GO:0018200	0.008103308	32	199	4	TTLL7,TTLL10,TPGS1,AGBL4
cell surface receptor signaling pathway	GO:0007166	0.008121796	2783	197	25	RSPO4,SIGIRR,BAMBI,GRB7,HAP1,MEF2C,WNT7A,RHBDF1,STAT5B,STRAP,IGFBP3,GRM7,EDA2R,IFT140,TFAP2B,UCMA,NFATC4,NRG1,GUCY2D,AKAP3,VANGL1,SRPX,ITFG2,ICAM1,BMP2

synaptic signaling	GO:0099536	0.008362063	736	158	11	PMP22,HAP1,MEF2C,WNT7A,CLSTN1,TPGS1,GRM7,NFATC4,NRG1,MYLK2,ELAVL4
ion transmembrane transport	GO:0034220	0.008623131	1116	86	10	CRACR2A,SLC26A8,SLC6A13,SLC25A41,KCNQ5,HAP1,MEF2C,ABCC8,CATSPER4,ITPR2
positive regulation of gene expression	GO:0010628	0.008631778	1117	197	15	OCLN,EIF2S3B,MEF2C,WNT7A,ABCC8,STAT5B,TENT5C,FUBP1,PTGFR,NFATC4,NRG1,MYLK2,ELAVL4,MAPK13,BMP2
transcription by RNA polymerase II	GO:0006366	0.009561702	2634	279	30	KLF8,DMRT2,FHL2,ZFHX2,MEF2C,WNT7A,NR1D2,STAT5B,WWC3,TEAD4,STRAP,ZNF208,HDAC9,TFAP2B,NFATC4,REST,ZNF626,N4BP2L2,UBE3A,BHLHA9,ZNF12,BMP2,ZNF726,MAGEB5,ZNF430,CCNH,HIVEP3,ELP2,TAF4B,HNRNPA2B1
response to organonitrogen compound	GO:0010243	0.009704082	1001	194	14	CRACR2A,ACTB,MEF2C,ERLEC1,ABCC8,STAT5B,ITPR2,GCK,HDAC9,ELAVL4,UBE3A,PALM,MAPK13,ICAM1
reproductive process	GO:0022414	0.010408693	1521	167	16	SLC26A8,PARP11,DMRT2,WNT7A,ABCC8,CATSPER4,STAT5B,TEAD4,PRKAR2A,TPGS1,PTGFR,LHFPL2,FAM9B,AKAP3,HERC2,UBE3A
inorganic ion transmembrane transport	GO:0098660	0.010613267	850	262	15	CRACR2A,SLC26A8,SLC6A13,KCNQ5,HAP1,ABCC8,CATSPER4,ITPR2,SLC6A17,SLC26A1,CBARP,CLCN4,SLC17A3,SLC4A4,HECW1
reproduction	GO:0000003	0.010668813	1524	167	16	SLC26A8,PARP11,DMRT2,WNT7A,ABCC8,CATSPER4,STAT5B,TEAD4,PRKAR2A,TPGS1,PTGFR,LHFPL2,FAM9B,AKAP3,HERC2,UBE3A
ventricular cardiac muscle cell differentiation	GO:0055012	0.010733714	15	136	3	FHL2,MEF2C,NRG1
muscle tissue development	GO:0060537	0.011620912	388	250	10	FHL2,MEF2C,NR1D2,HDAC9,TFAP2B,NRG1,MYLK2,BMP2,FLNB,HIVEP3
regulation of anatomical structure morphogenesis	GO:0022603	0.011822971	926	213	14	BAMBI,DMRT2,MEF2C,WNT7A,ABCC8,TWF2,NTN1,NFATC4,RHOBTB1,UBE3A,VANGL1,PALM,BMP2,DKK4
positive regulation of developmental process	GO:0051094	0.012468937	1271	136	13	BAMBI,DMRT2,HAP1,MEF2C,WNT7A,STAT5B,TWF2,TEAD4,CLSTN1,IGFBP3,NTN1,REST,NRG1
regulation of nervous system development	GO:0051960	0.014229479	438	135	8	HAP1,WNT7A,ABCC8,TWF2,CLSTN1,NTN1,NFATC4,REST
positive regulation of cellular component organization	GO:0051130	0.014486469	1021	197	14	OCLN,PLEKHM2,HAP1,WNT7A,SCARB2,ABCC8,TWF2,CLSTN1,SNX7,NTN1,NRG1,ELAVL4,PALM,BMP2

negative regulation of response to stimulus	GO:0048585	0.014924159	1604	262	21	SIGIRR,BAMBI,OPTN,FHL2,NR1D2,ABCC8,WWC3,STRAP,IGFBP3,UCMA,NFATC4,NRG1,PALM,ITFG2,ICAM1,BMP2,DKK4,GPRC5A,SNX13,OTUB1,HECW1
organic anion transport	GO:0015711	0.015215216	361	277	10	SLC26A8,SLC6A13,SLC25A41,SLCO1C1,GRM7,SLC6A17,SLC26A1,SLC17A3,SLC4A4,SLC16A1
inositol phosphate-mediated signaling	GO:0048016	0.017479603	56	136	4	FHL2,ITPR2,NFATC4,NRG1
negative regulation of signal transduction	GO:0009968	0.018076352	1242	262	18	SIGIRR,BAMBI,OPTN,FHL2,WWC3,STRAP,IGFBP3,UCMA,NFATC4,NRG1,PALM,ITFG2,ICAM1,BMP2,DKK4,GPRC5A,SNX13,HECW1
response to organic cyclic compound	GO:0014070	0.018268567	901	197	13	CRACR2A,ZFP36L2,ACTB,MEF2C,WNT7A,STAT5B,ITPR2,PTGFR,REST,ELAVL4,UBE3A,PALM,BMP2
organelle organization	GO:0006996	0.020069935	3805	287	38	CENPA,OCLN,COA5,PLEKHM2,GRB7,TTLL7,OPTN,MAST4,STXBP6,PARP11,DNAAF5,ACTB,HAP1,SCARB2,HASPIN,TWF2,TPGS1,SNX7,IFT140,HDAC9,RHOBTB1,SPAG17,PEX12,SRPX,PALM,TRAM2,FLNB,ADAMTS16,CLCN4,EVPLL,PADI4,NDUFB10,WIPI2,SLC16A1,MUS81,HNRNPA2B1,MICALL2,XPA
regulation of plasma membrane bounded cell projection assembly	GO:0120032	0.021105853	190	234	7	OCLN,HAP1,TWF2,IFT140,PALM,ICAM1,ADAMTS16
osteoblast differentiation	GO:0001649	0.021308668	226	197	7	BAMBI,FHL2,MEF2C,IGFBP3,UCMA,REST,BMP2
regulation of cell projection assembly	GO:0060491	0.022592169	192	234	7	OCLN,HAP1,TWF2,IFT140,PALM,ICAM1,ADAMTS16
negative regulation of cell differentiation	GO:0045596	0.02365456	657	197	11	BAMBI,ZFP36L2,WNT7A,ABCC8,STAT5B,NTN1,UCMA,NFATC4,REST,N4BP2L2,BMP2
cellular component assembly	GO:0022607	0.023827455	4556	107	22	CRACR2A,CENPA,OCLN,PMP22,EIF3M,COA5,GRB7,RNU6-1254P,STXBP6,DNAAF5,EIF2S3B,ACTB,HAP1,MEF2C,WNT7A,EMP1,TWF2,STRAP,CLSTN1,PRPF39,TPGS1,SNX7
response to nitrogen compound	GO:1901698	0.024741312	1088	194	14	CRACR2A,ACTB,MEF2C,ERLEC1,ABCC8,STAT5B,ITPR2,GCK,HDAC9,ELAVL4,UBE3A,PALM,MAPK13,ICAM1
circulatory system development	GO:0072359	0.024890154	1089	144	12	FHL2,MEF2C,WNT7A,ABCC8,ARHGAP22,IFT140,HDAC9,TFAP2B,NFATC4,REST,NRG1,MYLK2
maintenance of blood-brain barrier	GO:0035633	0.025208186	35	75	3	OCLN,ACTB,ABCC8
muscle organ development	GO:0007517	0.025486349	336	250	9	MEF2C,NR1D2,TEAD4,HDAC9,NRG1,MYLK2,BMP2,FLNB,HIVEP3

epithelium development	GO:0060429	0.026261659	1160	234	16	DMRT2,ACTB,MEF2C,WNT7A,STAT5B,IFT140,NTN1,TFAP2B,NFATC4,NRG1,VANGL1,ICAM1,BMP2,FLNB,DKK4,ADAMTS16
negative regulation of metabolic process	GO:0009892	0.026744896	3558	136	22	OCLN,SIGIRR,KLF8,MIR563,GRB7,ZFP36L2,OPTN,FHL2,HAP1,MEF2C,NR1D2,TENT5C,WWC3,STRAP,IGFBP3,PRKAR2A,GCK,HDAC9,TFAP2B,NFATC4,REST,NRG1
negative regulation of cellular biosynthetic process	GO:0031327	0.027237362	1581	136	14	KLF8,GRB7,ZFP36L2,FHL2,MEF2C,NR1D2,WWC3,STRAP,GCK,HDAC9,TFAP2B,NFATC4,REST,NRG1
chordate embryonic development	GO:0043009	0.027510076	661	199	11	DMRT2,TENT5C,TEAD4,IFT140,UCMA,KIAA1217,AKAP3,N4BP2L2,RRM2,BMP2,AGBL4
system process	GO:0003008	0.028235292	2243	277	26	OCLN,SLC6A13,ZFHX2,MEF2C,WNT7A,SLCO1C1,SCARB2,ABCC8,STRAP,OR1E1,GRM7,OR8H2,TFAP2B,NFATC4,GUCY2D,MYLK2,ELAVL4,ADRA1D,UBE3A,SLC6A17,CELF2,TAS2R10,ADAMTS16,SLC4A4,GAMT,SLC16A1
negative regulation of RNA metabolic process	GO:0051253	0.029171821	1395	197	16	KLF8,FHL2,MEF2C,NR1D2,TENT5C,WWC3,STRAP,HDAC9,TFAP2B,NFATC4,REST,NRG1,ELAVL4,N4BP2L2,ZNF12,BMP2
negative regulation of secretion by cell	GO:1903531	0.030324379	137	228	6	STXBP6,RHBDF1,ABCC8,GRM7,REST,CBARP
positive regulation of macromolecule metabolic process	GO:0010604	0.030350432	3378	144	22	OCLN,BAMBI,ZFP36L2,DMRT2,EIF2S3B,MEF2C,WNT7A,NR1D2,ABCC8,STAT5B,TENT5C,TEAD4,FUBP1,PTGFR,GCK,ZNF208,TFAP2B,NFATC4,REST,NRG1,RBM23,MYLK2
embryo development	GO:0009790	0.032982831	1088	199	14	DMRT2,MEF2C,WNT7A,TENT5C,TEAD4,IFT140,NTN1,UCMA,KIAA1217,AKAP3,N4BP2L2,RRM2,BMP2,AGBL4
negative regulation of neurogenesis	GO:0050768	0.033007888	138	135	5	WNT7A,ABCC8,NTN1,NFATC4,REST
negative regulation of biosynthetic process	GO:0009890	0.033311622	1610	136	14	KLF8,GRB7,ZFP36L2,FHL2,MEF2C,NR1D2,WWC3,STRAP,GCK,HDAC9,TFAP2B,NFATC4,REST,NRG1
regulation of neurogenesis	GO:0050767	0.035548254	358	135	7	HAP1,WNT7A,ABCC8,TWF2,NTN1,NFATC4,REST
negative regulation of macromolecule metabolic process	GO:0010605	0.035837126	3353	136	21	OCLN,SIGIRR,KLF8,MIR563,GRB7,ZFP36L2,OPTN,FHL2,HAP1,MEF2C,NR1D2,TENT5C,WWC3,STRAP,IGFBP3,PRKAR2A,HDAC9,TFAP2B,NFATC4,REST,NRG1
chemical synaptic transmission	GO:0007268	0.036160197	704	158	10	PMP22,HAP1,MEF2C,WNT7A,CLSTN1,TPGS1,GRM7,NFATC4,MYLK2,ELAVL4
anterograde trans-synaptic signaling	GO:0098916	0.036160197	704	158	10	PMP22,HAP1,MEF2C,WNT7A,CLSTN1,TPGS1,GRM7,NFATC4,MYLK2,ELAVL4
regulation of cell development	GO:0060284	0.036406041	499	135	8	HAP1,WNT7A,ABCC8,TWF2,NTN1,HDAC9,NFATC4,REST

positive regulation of multicellular organismal process	GO:0051240	0.036517483	1421	197	16	BAMBI,HAP1,MEF2C,WNT7A,ABCC8,STAT5B,TWF2,TEAD4,CLSTN1,NTN1,NFATC4,REST,NRG1,ADRA1D,MAPK13,BMP2
embryo development ending in birth or egg hatching	GO:0009792	0.036690818	682	199	11	DMRT2,TENT5C,TEAD4,IFT140,UCMA,KIAA1217,AKAP3,N4BP2L2,RRM2,BMP2,AGBL4
negative regulation of nervous system development	GO:0051961	0.039193765	143	135	5	WNT7A,ABCC8,NTN1,NFATC4,REST
trans-synaptic signaling	GO:0099537	0.039814225	712	158	10	PMP22,HAP1,MEF2C,WNT7A,CLSTN1,TPGS1,GRM7,NFATC4,MYLK2,ELAVL4
cellular component biogenesis	GO:0044085	0.040128579	4801	136	26	CRACR2A,CENPA,OCLN,PMP22,EIF3M,COA5,GRB7,RNU6-1254P,STXBP6,DNAAF5,EIF2S3B,ACTB,HAP1,MEF2C,WNT7A,EMP1,TWF2,STRAP,CLSTN1,PRPF39,TPGS1,SNX7,IFT140,NTN1,REST,NRG1
regulation of transmembrane transporter activity	GO:0022898	0.040817191	261	75	5	CRACR2A,ACTB,HAP1,MEF2C,ABCC8
cellular protein localization	GO:0034613	0.043727952	1954	287	24	CENPA,OCLN,OPTN,ACTB,HAP1,ERLEC1,SCARB2,CCHCR1,HASPIN,TWF2,CLSTN1,C17ORF75,IFT140,HERC2,PEX12,PALM,TBC1D26,TRAM2,ARL5C,SNX13,TM9SF1,WIPI2,MICALL2,XPA
cell population proliferation	GO:0008283	0.046630606	1940	199	19	PMP22,BAMBI,MEF2C,WNT7A,RHBDF1,ABCC8,STAT5B,IGFBP3,PTGFR,NTN1,TFAP2B,REST,NRG1,YPEL5,N4BP2L2,ADRA1D,SRPX,BMP2,AGBL4
cellular macromolecule localization	GO:0070727	0.047061044	1963	287	24	CENPA,OCLN,OPTN,ACTB,HAP1,ERLEC1,SCARB2,CCHCR1,HASPIN,TWF2,CLSTN1,C17ORF75,IFT140,HERC2,PEX12,PALM,TBC1D26,TRAM2,ARL5C,SNX13,TM9SF1,WIPI2,MICALL2,XPA
homeostatic process	GO:0042592	0.048531412	1774	128	14	OCLN,SIT1,ACTB,HAP1,MEF2C,NR1D2,ABCC8,STAT5B,ITPR2,STRAP,PTGFR,GCK,HDAC9,TFAP2B

GO terms with biological processes significantly enriched by downregulated DEGs

term_name	term_id	adjusted_p_value	term_size	query_size	intersection_size	intersections
biological_process	GO:0008150	1.84E-70	21028	494	366	POLR1B,ADORA2B,LYZL2,HLA-DQA2,HAPLN1,NCK2,APOB,FANCM,FOXA1,LMF1,BEST1,ZPBP,CHRNA7,TPGS2,PRKX,NLGN4X,TRIM33,SMS,ARF6,GINS1,PDIA2,UBA6,KLHL31,CCDC6,USP49,ZNF286A,TMEM204,ANTXR2,ITGB8,ARHGAP9,CXCL6,SKI,VWF,FCHO1,ZNF561,HPDL,SH3GL2,TMEM38A,SLC44A5,INO80B,BMPER,TOE1,SMC6,TSEN2,SPAG11A,DRC1,TADA3,HOMER3,ZNF506,TVP23B,CDA,BFSP1,ZNF121,TTC19,SPIDR,DHRS1,MACROD2,USP24,MEIS2,ICAM3,RANBP2,ADCY4,SOX5,SPATA6L,ADGRB3,CHRNA2,SYP,NDUFS7,MYH13,RBM24,TEC,LDLRAD3,DGKB,OR51E1,SLC38A1,FAM111B,NRP1,TAOK1,BCL7B,DSG1,SDCBP2,GSPT1,CEP72,KCTD7,BNIP3L,TIMM10,CTBP1,EEPD1,ZDHHC4,CARS1,XDH,PLK5,SLC39A3,POLN,NEMF,FSCN1,ARMC6,PRAM1,MAD2L1BP,SDHB,PHB2,SLC15A3,STRIP1,COPS2,MOCS1,RNU6-137P,CROCC,EOMES,UBQLN2,OSBPL5,SLC17A2,TRPC4,GRM2,CNR2,ADAM30,SH3GLB1,SIRPB1,AQP4,ZNF90,ZC3H8,ARL6IP5,INSM1,PGA5,HIC1,EAPP,SNORA59A,EML4,SLC25A23,SIRPA,KCNA1,TRMT2A,CTNS,AOAH,REG1A,GLIS1,C8B,LPAR3,ALOX12,ANLN,NOMO1,IP6K1,SLC30A2,ARAF,NKX2-8,PRPSAP2,CPNE6,UNC50,RNF112,MARS1,NEXN,PIRT,CHAC2,EEF2KMT,COMMD9,GPRIN2,ST18,COMMD1,EPOR,LASP1,WDR6,C10ORF71,RBBP4,MS4A3,SHOX,TEFM,HDAC6,MIR99AHG,CHAT,PERM1,MPHOSPH8,TANGO2,RCOR2,KRTAP4-5,RNPS1,PTCHD1,TJAP1,DNHD1,PCM1,PODN,CELSR2,SCN5A,TNRC6A,NELL1,SCP2,KCNS3,NPR3,EXTL3,OR7E24,C12ORF66,NSFL1C,EFCAB1,WNK3,ZNF721,KCNA4,HEPH,FCGR1B,GALK2,OXNAD1,LINC00273,DNM1L,BICD1,RELL1,PTPRO,CFP,ULK4,RNF144A,EBF4,NOP9,RAB26,MLST8,TXNDC12,MPP7,STAMBP,GOLGA8B,SVIP,BTBD10,UBXN2B,IP6K2,ANO6,RNF216,VCPKMT,TPM4,IL36RN,FAF1,THBD,EXOC6B,ADORA2A,MED1,DEFB129,AMY2B,KRT3,CDC25A,BAIAP3,DHX30,REG4,NPHP4,NSD2,KLHDC1,ZNF366,FRMD1,A2ML1,HECTD1,COX19,SYNC,RGS11,ALG5,NAA38,OR52I1,ATP8B3,PLAC1,PLEKHG4B,VIT,SLC5A10,SRRM2,CD320,FKBP6,NDUFAF2,CSDE1,FHIT,ZNF43,DENND2D,ALG8,RAD54L2,TMEM108,CMPK1,GRID2IP,KDM1B,SHROOM2,GNG7,LZTS1,DPY19L1,C5,TSGA10,ARHGAP29,FRMD5,LGI2,MYO1C,USP9X,MAL,SCN8A,PPIL4,ABTB2,OR5BS1P,ABCA4,TENT5A,ZSCAN29,NCEH1,ST7L,FBXO33,RHBDL2,MIR22HG,OGDH,BLOC1S6,MARCHF2,LTK,ZNF318,STAT2,CHMP3,MIR124-1HG,SLC25A44,EHD3,PSME2,PER1,CHST12,RPL15,AGXT2,TCF7L1,PLPPR4,MRPS36,SCML2,RNF24,LSM5,PDE6D,DAG1,SMG7,CARNS1,CREB3L1,GBP5,MADD,TBXA2R,SZT2,EPM2AIP1,ZNF676,RARB,SUCLA2,STAG1,PLPP2,RN

						F8,PRKAA2,CDK5R1,NEK7,MNT,PRRG3,C3,ZMYND11,IGKV6-21,CSKMT,POLE,RAF1,RASA4B,FMNL3,CTRC,ITIH5,CALCRL,HARBI1,LIMA1,GALNT14,ATF1,CILK1,ZMPSTE24,TRAPPC1,HPCA,RPS6KA3,TBL2,CREB5,NPHP1,SPATA16,ZWILCH,KRT18,PINK1,ZNF143,H4C8,LGSN,SNX31,ZNF320,FAM168A

cellular process	GO:0009987	2.40E-62	19329	494	340	POLR1B,ADORA2B,HLA-DQA2,HAPLN1,NCK2,APOB,FANCM,FOXA1,LMF1,BEST1,ZPBP,CHRNA7,TPGS2,PRKX,NLGN4X,TRIM33,SMS,ARF6,GINS1,PDIA2,UBA6,KLHL31,CCDC6,USP49,ZNF286A,TMEM204,ITGB8,ARHGAP9,CXCL6,SKI,VWF,FCHO1,ZNF561,HPDL,SH3GL2,TMEM38A,SLC44A5,INO80B,BMPER,TOE1,SMC6,TSEN2,DRC1,TADA3,HOMER3,ZNF506,TVP23B,CDA,BFSP1,ZNF121,TTC19,SPIDR,DHRS1,MACROD2,USP24,MEIS2,ICAM3,RANBP2,ADCY4,SOX5,ADGRB3,CHRNA2,SYP,NDUFS7,MYH13,RBM24,TEC,DGKB,OR51E1,SLC38A1,FAM111B,NRP1,TAOK1,BCL7B,DSG1,SDCBP2,GSPT1,CEP72,KCTD7,BNIP3L,TIMM10,CTBP1,EEPD1,ZDHHC4,CARS1,XDH,PLK5,SLC39A3,POLN,NEMF,FSCN1,ARMC6,PRAM1,MAD2L1BP,SDHB,PHB2,SLC15A3,STRIP1,COPS2,MOCS1,RNU6-137P,CROCC,EOMES,UBQLN2,OSBPL5,SLC17A2,TRPC4,GRM2,CNR2,SH3GLB1,SIRPB1,AQP4,ZNF90,ZC3H8,ARL6IP5,INSM1,HIC1,EAPP,SNORA59A,EML4,SLC25A23,SIRPA,KCNA1,TRMT2A,CTNS,AOAH,REG1A,GLIS1,C8B,LPAR3,ALOX12,ANLN,IP6K1,SLC30A2,ARAF,NKX2-8,PRPSAP2,CPNE6,UNC50,RNF112,MARS1,NEXN,PIRT,CHAC2,EEF2KMT,GPRIN2,ST18,COMMD1,EPOR,LASP1,WDR6,C10ORF71,RBBP4,MS4A3,SHOX,TEFM,HDAC6,CHAT,PERM1,MPHOSPH8,TANGO2,RCOR2,RNPS1,PTCHD1,TJAP1,DNHD1,PCM1,PODN,CELSR2,SCN5A,TNRC6A,NELL1,SCP2,KCNS3,NPR3,EXTL3,OR7E24,C12ORF66,NSFL1C,EFCAB1,WNK3,ZNF721,KCNA4,HEPH,FCGR1B,GALK2,DNM1L,BICD1,RELL1,PTPRO,ULK4,RNF144A,EBF4,NOP9,RAB26,MLST8,TXNDC12,MPP7,STAMBP,GOLGA8B,SVIP,BTBD10,UBXN2B,IP6K2,ANO6,RNF216,VCPKMT,TPM4,IL36RN,FAF1,THBD,EXOC6B,ADORA2A,MED1,KRT3,CDC25A,BAIAP3,DHX30,NPHP4,NSD2,KLHDC1,ZNF366,FRMD1,A2ML1,HECTD1,COX19,SYNC,RGS11,ALG5,NAA38,OR52I1,ATP8B3,PLEKHG4B,VIT,SLC5A10,SRRM2,CD320,FKBP6,NDUFAF2,CSDE1,FHIT,ZNF43,ALG8,RAD54L2,TMEM108,CMPK1,GRID2IP,KDM1B,SHROOM2,GNG7,LZTS1,DPY19L1,C5,ARHGAP29,FRMD5,LGI2,MYO1C,USP9X,MAL,SCN8A,PPIL4,ABTB2,OR5BS1P,ABCA4,TENT5A,ZSCAN29,NCEH1,ST7L,FBXO33,OGDH,BLOC1S6,MARCHF2,LTK,ZNF318,STAT2,CHMP3,SLC25A44,EHD3,PSME2,PER1,CHST12,RPL15,AGXT2,TCF7L1,PLPPR4,MRPS36,SCML2,RNF24,LSM5,DAG1,SMG7,CARNS1,CREB3L1,GBP5,MADD,TBXA2R,SZT2,EPM2AIP1,ZNF676,RARB,SUCLA2,STAG1,PLPP2,RNF8,PRKAA2,CDK5R1,NEK7,MNT,C3,ZMYND11,CSKMT,POLE,RAF1,RASA4B,FMNL3,CTRC,ITIH5,CALCRL,HARBI1,LIMA1,GALNT14,ATF1,CILK1,ZMPSTE24,TRAPPC1,HPCA,RPS6KA3,TBL2,CREB5,NPHP1,SPATA16,ZWILCH,KRT18,PINK1,ZNF143,H4C8,LGSN,SNX31,ZNF320,FAM168A

biological regulation	GO:0065007	2.65E-55	12859	494	266	ADORA2B,HLA-DQA2,NCK2,APOB,FANCM,FOXA1,BEST1,CHRNA7,PRKX,NLGN4X,TRIM33,ARF6,KLHL31,ZNF286A,TMEM204,ITGB8,ARHGAP9,CXCL6,SKI,VWF,FCHO1,ZNF561,SH3GL2,TMEM38A,BMPER,SMC6,DRC1,TADA3,HOMER3,ZNF506,CDA,ZNF121,SPIDR,MEIS2,RANBP2,ADCY4,SOX5,ADGRB3,CHRNA2,SYP,RBM24,TEC,LDLRAD3,DGKB,OR51E1,SLC38A1,NRP1,TAOK1,BCL7B,DSG1,SDCBP2,GSPT1,CEP72,KCTD7,BNIP3L,CTBP1,EEPD1,XDH,PLK5,SLC39A3,FSCN1,PRAM1,MAD2L1BP,PHB2,SLC15A3,STRIP1,COPS2,CROCC,EOMES,UBQLN2,TRPC4,GRM2,CNR2,SH3GLB1,SIRPB1,AQP4,ZNF90,ZC3H8,ARL6IP5,INSM1,HIC1,EAPP,SLC25A23,SIRPA,KCNA1,CTNS,AOAH,REG1A,GLIS1,C8B,LPAR3,ALOX12,ANLN,IP6K1,SLC30A2,ARAF,NKX2-8,PRPSAP2,CPNE6,RNF112,MARS1,NEXN,PIRT,COMMD9,ST18,COMMD1,EPOR,WDR6,C10ORF71,RBBP4,MS4A3,SHOX,TEFM,HDAC6,MIR99AHG,CHAT,PERM1,MPHOSPH8,RCOR2,RNPS1,PTCHD1,PCM1,PODN,CELSR2,SCN5A,TNRC6A,NELL1,SCP2,KCNS3,NPR3,EXTL3,OR7E24,C12ORF66,NSFL1C,EFCAB1,WNK3,ZNF721,KCNA4,HEPH,FCGR1B,LINC00273,DNM1L,BICD1,RELL1,PTPRO,CFP,ULK4,RNF144A,EBF4,RAB26,MLST8,TXNDC12,MPP7,STAMBP,SVIP,BTBD10,UBXN2B,IP6K2,ANO6,RNF216,VCPKMT,IL36RN,FAF1,THBD,ADORA2A,MED1,CDC25A,BAIAP3,NPHP4,NSD2,ZNF366,FRMD1,A2ML1,HECTD1,COX19,RGS11,NAA38,OR52I1,ATP8B3,PLEKHG4B,VIT,CD320,FKBP6,NDUFAF2,CSDE1,FHIT,ZNF43,DENND2D,TMEM108,GRID2IP,KDM1B,GNG7,LZTS1,C5,ARHGAP29,FRMD5,MYO1C,USP9X,MAL,SCN8A,PPIL4,OR5BS1P,ABCA4,TENT5A,ZSCAN29,NCEH1,ST7L,FBXO33,MIR22HG,BLOC1S6,MARCHF2,LTK,ZNF318,STAT2,CHMP3,MIR124-1HG,SLC25A44,EHD3,PSME2,PER1,AGXT2,TCF7L1,PLPPR4,SCML2,LSM5,PDE6D,DAG1,SMG7,CREB3L1,GBP5,MADD,TBXA2R,SZT2,EPM2AIP1,ZNF676,RARB,PLPP2,RNF8,PRKAA2,CDK5R1,NEK7,MNT,C3,ZMYND11,RAF1,RASA4B,FMNL3,CTRC,ITIH5,CALCRL,LIMA1,ATF1,CILK1,ZMPSTE24,HPCA,RPS6KA3,TBL2,CREB5,NPHP1,ZWILCH,KRT18,PINK1,ZNF143,H4C8,ZNF320,FAM168A

regulation of biological process	GO:0050789	1.08E-50	12142	494	251	ADORA2B,HLA-DQA2,NCK2,APOB,FANCM,FOXA1,BEST1,CHRNA7,PRKX,NLGN4X,TRIM33,ARF6,KLHL31,ZNF286A,TMEM204,ITGB8,ARHGAP9,CXCL6,SKI,VWF,FCHO1,ZNF561,SH3GL2,TMEM38A,BMPER,SMC6,DRC1,TADA3,HOMER3,ZNF506,CDA,ZNF121,SPIDR,MEIS2,RANBP2,ADCY4,SOX5,ADGRB3,CHRNA2,SYP,RBM24,TEC,LDLRAD3,DGKB,OR51E1,NRP1,TAOK1,BCL7B,SDCBP2,GSPT1,CEP72,KCTD7,BNIP3L,CTBP1,EEPD1,XDH,PLK5,FSCN1,PRAM1,MAD2L1BP,PHB2,SLC15A3,STRIP1,COPS2,CROCC,EOMES,UBQLN2,GRM2,CNR2,SH3GLB1,SIRPB1,AQP4,ZNF90,ZC3H8,ARL6IP5,INSM1,HIC1,EAPP,SLC25A23,SIRPA,KCNA1,CTNS,AOAH,REG1A,GLIS1,C8B,LPAR3,ALOX12,ANLN,IP6K1,SLC30A2,ARAF,NKX2-8,CPNE6,RNF112,MARS1,NEXN,PIRT,ST18,COMMD1,EPOR,WDR6,C10ORF71,RBBP4,MS4A3,SHOX,HDAC6,MIR99AHG,PERM1,MPHOSPH8,RCOR2,RNPS1,PTCHD1,PCM1,PODN,CELSR2,SCN5A,TNRC6A,NELL1,SCP2,KCNS3,NPR3,EXTL3,OR7E24,C12ORF66,NSFL1C,EFCAB1,WNK3,ZNF721,KCNA4,FCGR1B,LINC00273,DNM1L,BICD1,RELL1,PTPRO,CFP,ULK4,RNF144A,EBF4,RAB26,MLST8,TXNDC12,MPP7,STAMBP,SVIP,BTBD10,UBXN2B,IP6K2,ANO6,RNF216,IL36RN,FAF1,THBD,ADORA2A,MED1,CDC25A,BAIAP3,NPHP4,NSD2,ZNF366,FRMD1,A2ML1,HECTD1,RGS11,NAA38,OR52I1,PLEKHG4B,VIT,CD320,FKBP6,NDUFAF2,CSDE1,FHIT,ZNF43,TMEM108,GRID2IP,KDM1B,GNG7,LZTS1,C5,ARHGAP29,FRMD5,MYO1C,USP9X,MAL,SCN8A,PPIL4,OR5BS1P,ABCA4,TENT5A,ZSCAN29,NCEH1,ST7L,FBXO33,MIR22HG,BLOC1S6,MARCHF2,LTK,ZNF318,STAT2,CHMP3,MIR124-1HG,SLC25A44,EHD3,PSME2,PER1,AGXT2,TCF7L1,PLPPR4,SCML2,LSM5,DAG1,SMG7,CREB3L1,GBP5,MADD,TBXA2R,SZT2,EPM2AIP1,ZNF676,RARB,PLPP2,RNF8,PRKAA2,CDK5R1,NEK7,MNT,C3,ZMYND11,RAF1,RASA4B,FMNL3,ITIH5,CALCRL,LIMA1,ATF1,CILK1,ZMPSTE24,HPCA,RPS6KA3,TBL2,CREB5,NPHP1,ZWILCH,KRT18,PINK1,ZNF143,H4C8,ZNF320,FAM168A

regulation of cellular process	GO:0050794	5.91E-47	11067	494	233	ADORA2B,HLA-DQA2,NCK2,APOB,FANCM,FOXA1,CHRNA7,PRKX,NLGN4X,TRIM33,ARF6,KLHL31,ZNF286A,TMEM204,ITGB8,ARHGAP9,CXCL6,SKI,VWF,FCHO1,ZNF561,SH3GL2,TMEM38A,BMPER,SMC6,DRC1,TADA3,HOMER3,ZNF506,CDA,ZNF121,SPIDR,MEIS2,RANBP2,ADCY4,SOX5,ADGRB3,CHRNA2,SYP,RBM24,TEC,DGKB,OR51E1,NRP1,TAOK1,BCL7B,SDCBP2,GSPT1,CEP72,BNIP3L,CTBP1,XDH,PLK5,FSCN1,PRAM1,MAD2L1BP,PHB2,SLC15A3,COPS2,CROCC,EOMES,UBQLN2,GRM2,CNR2,SH3GLB1,SIRPB1,AQP4,ZNF90,ZC3H8,ARL6IP5,INSM1,HIC1,EAPP,SLC25A23,SIRPA,KCNA1,CTNS,REG1A,GLIS1,LPAR3,ALOX12,ANLN,SLC30A2,ARAF,NKX2-8,CPNE6,RNF112,MARS1,NEXN,PIRT,ST18,COMMD1,EPOR,WDR6,C10ORF71,RBBP4,MS4A3,SHOX,HDAC6,PERM1,MPHOSPH8,RCOR2,RNPS1,PTCHD1,PCM1,PODN,CELSR2,SCN5A,TNRC6A,NELL1,SCP2,KCNS3,NPR3,EXTL3,OR7E24,C12ORF66,NSFL1C,EFCAB1,WNK3,ZNF721,KCNA4,FCGR1B,DNM1L,BICD1,RELL1,PTPRO,ULK4,RNF144A,EBF4,RAB26,MLST8,TXNDC12,MPP7,STAMBP,SVIP,BTBD10,UBXN2B,IP6K2,ANO6,IL36RN,FAF1,THBD,ADORA2A,MED1,CDC25A,BAIAP3,NPHP4,NSD2,ZNF366,FRMD1,A2ML1,HECTD1,RGS11,NAA38,OR52I1,PLEKHG4B,VIT,CD320,NDUFAF2,CSDE1,FHIT,ZNF43,TMEM108,GRID2IP,KDM1B,GNG7,LZTS1,C5,ARHGAP29,FRMD5,MYO1C,USP9X,MAL,SCN8A,PPIL4,OR5BS1P,ABCA4,TENT5A,ZSCAN29,NCEH1,ST7L,FBXO33,BLOC1S6,MARCHF2,LTK,ZNF318,STAT2,CHMP3,EHD3,PSME2,PER1,AGXT2,TCF7L1,PLPPR4,SCML2,DAG1,SMG7,CREB3L1,GBP5,MADD,TBXA2R,SZT2,EPM2AIP1,ZNF676,RARB,PLPP2,RNF8,PRKAA2,CDK5R1,NEK7,MNT,C3,ZMYND11,RAF1,RASA4B,ITIH5,CALCRL,LIMA1,ATF1,CILK1,ZMPSTE24,HPCA,RPS6KA3,TBL2,CREB5,NPHP1,ZWILCH,KRT18,PINK1,ZNF143,H4C8,ZNF320,FAM168A

response to stimulus	GO:0050896	1.70E-34	8815	487	184	ADORA2B,HLA-DQA2,NCK2,APOB,FANCM,FOXA1,BEST1,CHRNA7,PRKX,NLGN4X,TRIM33,ARF6,PDIA2,UBA6,KLHL31,TMEM204,ITGB8,ARHGAP9,CXCL6,SKI,VWF,FCHO1,SH3GL2,TMEM38A,INO80B,BMPER,SMC6,SPAG11A,TADA3,HOMER3,CDA,SPIDR,MACROD2,MEIS2,ADCY4,SOX5,ADGRB3,CHRNA2,SYP,MYH13,RBM24,TEC,DGKB,OR51E1,NRP1,TAOK1,BCL7B,DSG1,SDCBP2,BNIP3L,EEPD1,XDH,PLK5,POLN,PRAM1,MAD2L1BP,PHB2,SLC15A3,COPS2,EOMES,UBQLN2,GRM2,CNR2,SH3GLB1,SIRPB1,AQP4,ZC3H8,ARL6IP5,HIC1,SLC25A23,SIRPA,KCNA1,CTNS,AOAH,REG1A,C8B,LPAR3,ALOX12,SLC30A2,ARAF,CPNE6,RNF112,MARS1,NEXN,PIRT,ST18,COMMD1,EPOR,C10ORF71,HDAC6,PERM1,PTCHD1,CELSR2,SCN5A,TNRC6A,NPR3,OR7E24,C12ORF66,WNK3,FCGR1B,DNM1L,BICD1,RELL1,PTPRO,CFP,ULK4,MLST8,TXNDC12,STAMBP,SVIP,IP6K2,ANO6,RNF216,IL36RN,FAF1,THBD,ADORA2A,MED1,DEFB129,CDC25A,BAIAP3,REG4,NPHP4,NSD2,ZNF366,FRMD1,RGS11,OR52I1,PLEKHG4B,CD320,NDUFAF2,FHIT,TMEM108,SHROOM2,GNG7,C5,ARHGAP29,MYO1C,USP9X,MAL,ABTB2,OR5BS1P,ABCA4,TENT5A,NCEH1,BLOC1S6,MARCHF2,LTK,STAT2,PER1,TCF7L1,PLPPR4,LSM5,PDE6D,DAG1,CREB3L1,GBP5,MADD,TBXA2R,SZT2,EPM2AIP1,RARB,PLPP2,RNF8,PRKAA2,CDK5R1,MNT,C3,ZMYND11,IGKV6-21,POLE,RAF1,RASA4B,CALCRL,ATF1,CILK1,ZMPSTE24,HPCA,RPS6KA3,TBL2,NPHP1,ZWILCH,KRT18,PINK1

localization	GO:0051179	1.14E-30	6425	492	149	ADORA2B,NCK2,APOB,BEST1,CHRNA7,PRKX,NLGN4X,ARF6,PDIA2,ANTXR2,ITGB8,CXCL6,SKI,FCHO1,SH3GL2,TMEM38A,SLC44A5,BMPER,DRC1,HOMER3,TVP23B,SPIDR,DHRS1,ICAM3,RANBP2,CHRNA2,SYP,NDUFS7,LDLRAD3,SLC38A1,NRP1,SDCBP2,CEP72,KCTD7,BNIP3L,TIMM10,EEPD1,ZDHHC4,XDH,SLC39A3,NEMF,FSCN1,PRAM1,PHB2,SLC15A3,CROCC,UBQLN2,OSBPL5,SLC17A2,TRPC4,GRM2,CNR2,SH3GLB1,SIRPB1,AQP4,ARL6IP5,INSM1,EML4,SLC25A23,SIRPA,KCNA1,CTNS,ANLN,SLC30A2,CPNE6,UNC50,NEXN,PIRT,COMMD9,COMMD1,LASP1,HDAC6,CHAT,TANGO2,PCM1,PODN,CELSR2,SCN5A,SCP2,KCNS3,NPR3,C12ORF66,NSFL1C,EFCAB1,WNK3,KCNA4,HEPH,DNM1L,BICD1,PTPRO,ULK4,NOP9,RAB26,MPP7,SVIP,UBXN2B,IP6K2,ANO6,FAF1,EXOC6B,ADORA2A,MED1,BAIAP3,NPHP4,NSD2,HECTD1,ATP8B3,SLC5A10,CD320,NDUFAF2,TMEM108,SHROOM2,C5,FRMD5,MYO1C,USP9X,MAL,SCN8A,ABCA4,OGDH,BLOC1S6,MARCHF2,CHMP3,SLC25A44,EHD3,PER1,PLPPR4,DAG1,SMG7,CREB3L1,GBP5,TBXA2R,SZT2,PRKAA2,CDK5R1,C3,RAF1,FMNL3,CALCRL,LIMA1,CILK1,ZMPSTE24,TRAPPC1,HPCA,NPHP1,ZWILCH,KRT18,PINK1,SNX31
cellular response to stimulus	GO:0051716	2.48E-30	7346	487	159	ADORA2B,NCK2,APOB,FANCM,FOXA1,CHRNA7,PRKX,NLGN4X,TRIM33,ARF6,PDIA2,UBA6,KLHL31,TMEM204,ITGB8,ARHGAP9,CXCL6,SKI,VWF,FCHO1,SH3GL2,TMEM38A,INO80B,BMPER,SMC6,TADA3,HOMER3,CDA,SPIDR,MACROD2,ADCY4,SOX5,ADGRB3,CHRNA2,SYP,MYH13,RBM24,TEC,DGKB,OR51E1,NRP1,TAOK1,BCL7B,SDCBP2,BNIP3L,EEPD1,XDH,PLK5,POLN,PRAM1,MAD2L1BP,PHB2,SLC15A3,COPS2,UBQLN2,GRM2,CNR2,SH3GLB1,SIRPB1,AQP4,ARL6IP5,HIC1,SLC25A23,SIRPA,KCNA1,CTNS,REG1A,LPAR3,ARAF,CPNE6,RNF112,MARS1,PIRT,ST18,COMMD1,EPOR,C10ORF71,HDAC6,PTCHD1,CELSR2,SCN5A,TNRC6A,NPR3,OR7E24,C12ORF66,WNK3,FCGR1B,DNM1L,BICD1,RELL1,PTPRO,ULK4,MLST8,TXNDC12,STAMBP,SVIP,IP6K2,ANO6,IL36RN,FAF1,ADORA2A,MED1,CDC25A,BAIAP3,NPHP4,NSD2,ZNF366,FRMD1,RGS11,OR52I1,PLEKHG4B,CD320,NDUFAF2,FHIT,TMEM108,SHROOM2,GNG7,C5,ARHGAP29,MYO1C,USP9X,MAL,ABTB2,OR5BS1P,ABCA4,NCEH1,BLOC1S6,LTK,STAT2,PER1,TCF7L1,PLPPR4,DAG1,CREB3L1,GBP5,MADD,TBXA2R,SZT2,RARB,PLPP2,RNF8,PRKAA2,CDK5R1,MNT,C3,ZMYND11,POLE,RAF1,RASA4B,CALCRL,CILK1,ZMPSTE24,HPCA,RPS6KA3,TBL2,NPHP1,ZWILCH,KRT18,PINK1

positive regulation of biological process	GO:0048518	1.60E-29	6094	488	142	ADORA2B,HLA-DQA2,NCK2,APOB,FANCM,FOXA1,CHRNA7,ARF6,ITGB8,SKI,VWF,FCHO1,BMPER,SMC6,TADA3,SPIDR,MEIS2,RANBP2,SOX5,ADGRB3,RBM24,TEC,NRP1,TAOK1,KCTD7,BNIP3L,CTBP1,EEPD1,XDH,PLK5,FSCN1,PRAM1,MAD2L1BP,PHB2,SLC15A3,CROCC,EOMES,UBQLN2,SH3GLB1,SIRPB1,ZC3H8,ARL6IP5,INSM1,HIC1,EAPP,SIRPA,KCNA1,CTNS,REG1A,GLIS1,C8B,LPAR3,ANLN,SLC30A2,ARAF,NKX2-8,CPNE6,RNF112,MARS1,PIRT,ST18,COMMD1,C10ORF71,SHOX,HDAC6,MPHOSPH8,RNPS1,PCM1,SCN5A,TNRC6A,NELL1,SCP2,EXTL3,NSFL1C,WNK3,ZNF721,DNM1L,BICD1,RELL1,CFP,RNF144A,MLST8,MPP7,STAMBP,SVIP,BTBD10,UBXN2B,IP6K2,ANO6,FAF1,THBD,ADORA2A,MED1,CDC25A,BAIAP3,NPHP4,NSD2,FRMD1,HECTD1,VIT,CD320,FKBP6,ZNF43,TMEM108,KDM1B,C5,FRMD5,MYO1C,USP9X,MAL,TENT5A,FBXO33,BLOC1S6,MARCHF2,LTK,ZNF318,EHD3,PSME2,PER1,AGXT2,DAG1,CREB3L1,GBP5,MADD,TBXA2R,EPM2AIP1,RARB,RNF8,PRKAA2,CDK5R1,NEK7,C3,RAF1,CALCRL,ATF1,ZMPSTE24,HPCA,RPS6KA3,CREB5,NPHP1,PINK1,ZNF143

metabolic process	GO:0008152	5.03E-27	14361	494	231	POLR1B,ADORA2B,LYZL2,NCK2,APOB,FANCM,FOXA1,LMF1,CHRNA7,TPGS2,PRKX,TRIM33,SMS,GINS1,UBA6,KLHL31,USP49,ZNF286A,ITGB8,CXCL6,SKI,ZNF561,HPDL,SH3GL2,SLC44A5,INO80B,BMPER,TOE1,SMC6,TSEN2,TADA3,HOMER3,ZNF506,CDA,ZNF121,SPIDR,MACROD2,USP24,MEIS2,RANBP2,ADCY4,SOX5,NDUFS7,RBM24,TEC,LDLRAD3,DGKB,FAM111B,NRP1,TAOK1,GSPT1,BNIP3L,CTBP1,EEPD1,ZDHHC4,CARS1,XDH,PLK5,POLN,NEMF,SDHB,PHB2,COPS2,MOCS1,RNU6-137P,EOMES,UBQLN2,OSBPL5,SLC17A2,GRM2,CNR2,ADAM30,SH3GLB1,AQP4,ZNF90,ZC3H8,ARL6IP5,INSM1,PGA5,HIC1,EAPP,SNORA59A,SLC25A23,SIRPA,TRMT2A,CTNS,AOAH,GLIS1,LPAR3,ALOX12,IP6K1,ARAF,NKX2-8,PRPSAP2,CPNE6,RNF112,MARS1,CHAC2,EEF2KMT,ST18,COMMD1,WDR6,RBBP4,SHOX,TEFM,HDAC6,MIR99AHG,CHAT,PERM1,MPHOSPH8,RCOR2,RNPS1,CELSR2,TNRC6A,NELL1,SCP2,NPR3,EXTL3,NSFL1C,WNK3,ZNF721,GALK2,LINC00273,DNM1L,BICD1,PTPRO,ULK4,RNF144A,EBF4,NOP9,RAB26,MLST8,STAMBP,SVIP,BTBD10,UBXN2B,IP6K2,RNF216,VCPKMT,IL36RN,FAF1,ADORA2A,MED1,AMY2B,CDC25A,NSD2,KLHDC1,ZNF366,A2ML1,HECTD1,ALG5,SRRM2,CD320,FKBP6,CSDE1,FHIT,ZNF43,ALG8,CMPK1,KDM1B,LZTS1,DPY19L1,C5,USP9X,PPIL4,ABCA4,TENT5A,ZSCAN29,NCEH1,FBXO33,RHBDL2,MIR22HG,OGDH,MARCHF2,LTK,ZNF318,STAT2,CHMP3,MIR124-1HG,SLC25A44,EHD3,PSME2,PER1,CHST12,RPL15,AGXT2,TCF7L1,PLPPR4,MRPS36,SCML2,RNF24,LSM5,DAG1,SMG7,CARNS1,CREB3L1,GBP5,MADD,EPM2AIP1,ZNF676,RARB,SUCLA2,PLPP2,RNF8,PRKAA2,CDK5R1,NEK7,MNT,C3,ZMYND11,CSKMT,POLE,RAF1,CTRC,ITIH5,CALCRL,HARBI1,LIMA1,GALNT14,ATF1,CILK1,ZMPSTE24,HPCA,RPS6KA3,CREB5,PINK1,ZNF143,H4C8,LGSN,ZNF320,FAM168A

positive regulation of cellular process	GO:0048522	1.29E-26	5561	494	131	ADORA2B,HLA-DQA2,NCK2,APOB,FANCM,FOXA1,CHRNA7,ARF6,SKI,VWF,FCHO1,BMPER,SMC6,TADA3,SPIDR,MEIS2,RANBP2,SOX5,ADGRB3,RBM24,TEC,NRP1,TAOK1,BNIP3L,CTBP1,XDH,PLK5,FSCN1,MAD2L1BP,PHB2,SLC15A3,CROCC,EOMES,UBQLN2,SH3GLB1,SIRPB1,ZC3H8,ARL6IP5,INSM1,HIC1,EAPP,SIRPA,KCNA1,CTNS,REG1A,GLIS1,LPAR3,ANLN,SLC30A2,ARAF,NKX2-8,CPNE6,RNF112,MARS1,PIRT,ST18,COMMD1,C10ORF71,SHOX,HDAC6,MPHOSPH8,RNPS1,PCM1,SCN5A,TNRC6A,NELL1,SCP2,EXTL3,NSFL1C,WNK3,ZNF721,DNM1L,BICD1,RELL1,RNF144A,MLST8,MPP7,STAMBP,SVIP,BTBD10,UBXN2B,IP6K2,ANO6,FAF1,ADORA2A,MED1,CDC25A,BAIAP3,NPHP4,NSD2,FRMD1,HECTD1,VIT,CD320,ZNF43,TMEM108,KDM1B,FRMD5,MYO1C,USP9X,MAL,FBXO33,BLOC1S6,MARCHF2,LTK,ZNF318,EHD3,PSME2,PER1,AGXT2,DAG1,CREB3L1,GBP5,MADD,EPM2AIP1,RARB,RNF8,PRKAA2,CDK5R1,NEK7,C3,RAF1,CALCRL,ATF1,HPCA,RPS6KA3,CREB5,NPHP1,PINK1,ZNF143,FAM168A
cell communication	GO:0007154	2.68E-25	6465	487	139	ADORA2B,NCK2,FOXA1,CHRNA7,PRKX,NLGN4X,TRIM33,ARF6,KLHL31,TMEM204,ITGB8,ARHGAP9,CXCL6,SKI,VWF,FCHO1,SH3GL2,TMEM38A,BMPER,TADA3,HOMER3,CDA,ADCY4,ADGRB3,CHRNA2,SYP,MYH13,TEC,DGKB,OR51E1,NRP1,TAOK1,BCL7B,SDCBP2,BNIP3L,XDH,PLK5,PRAM1,MAD2L1BP,PHB2,SLC15A3,COPS2,UBQLN2,GRM2,CNR2,SH3GLB1,SIRPB1,ARL6IP5,HIC1,SIRPA,KCNA1,CTNS,REG1A,LPAR3,ARAF,CPNE6,MARS1,PIRT,ST18,EPOR,C10ORF71,HDAC6,CHAT,PTCHD1,CELSR2,SCN5A,TNRC6A,NPR3,OR7E24,C12ORF66,WNK3,FCGR1B,DNM1L,BICD1,RELL1,PTPRO,ULK4,RAB26,MLST8,TXNDC12,STAMBP,ANO6,IL36RN,FAF1,ADORA2A,MED1,BAIAP3,NPHP4,ZNF366,FRMD1,RGS11,OR52I1,PLEKHG4B,CD320,NDUFAF2,FHIT,TMEM108,GRID2IP,GNG7,LZTS1,C5,ARHGAP29,MYO1C,USP9X,MAL,SCN8A,OR5BS1P,ABCA4,NCEH1,BLOC1S6,LTK,STAT2,PER1,TCF7L1,PLPPR4,DAG1,CREB3L1,MADD,TBXA2R,SZT2,RARB,PLPP2,PRKAA2,CDK5R1,MNT,C3,ZMYND11,RAF1,RASA4B,CALCRL,CILK1,ZMPSTE24,HPCA,RPS6KA3,TBL2,NPHP1,ZWILCH,KRT18,PINK1

multicellular organismal process	GO:0032501	5.79E-25	7474	484	150	POLR1B,ADORA2B,HAPLN1,NCK2,APOB,FOXA1,BEST1,ZPBP,CHRNA7,PRKX,NLGN4X,ARF6,GINS1,UBA6,TMEM204,ITGB8,CXCL6,SKI,VWF,SH3GL2,TMEM38A,BMPER,SPAG11A,DRC1,HOMER3,BFSP1,MACROD2,MEIS2,SOX5,SPATA6L,ADGRB3,CHRNA2,MYH13,RBM24,TEC,DGKB,OR51E1,SLC38A1,NRP1,TAOK1,DSG1,SDCBP2,TIMM10,XDH,PLK5,SLC39A3,ARMC6,PHB2,COPS2,CROCC,EOMES,TRPC4,GRM2,CNR2,AQP4,ZC3H8,ARL6IP5,INSM1,PGA5,SIRPA,KCNA1,CTNS,GLIS1,LPAR3,ALOX12,ANLN,IP6K1,NKX2-8,PRPSAP2,CPNE6,RNF112,NEXN,PIRT,GPRIN2,EPOR,SHOX,HDAC6,KRTAP4-5,PTCHD1,PCM1,CELSR2,SCN5A,NELL1,NPR3,OR7E24,WNK3,DNM1L,PTPRO,ULK4,RAB26,ANO6,RNF216,TPM4,IL36RN,THBD,ADORA2A,MED1,DHX30,NPHP4,NSD2,HECTD1,ALG5,OR52I1,PLAC1,VIT,FKBP6,CSDE1,TMEM108,KDM1B,SHROOM2,LZTS1,C5,TSGA10,LGI2,USP9X,MAL,SCN8A,OR5BS1P,ABCA4,NCEH1,OGDH,BLOC1S6,LTK,SLC25A44,EHD3,PER1,PLPPR4,PDE6D,DAG1,CREB3L1,GBP5,TBXA2R,SZT2,RARB,RNF8,PRKAA2,CDK5R1,MNT,C3,POLE,RAF1,FMNL3,CALCRL,LIMA1,ATF1,ZMPSTE24,HPCA,RPS6KA3,NPHP1,SPATA16

organic substance metabolic process	GO:0071704	7.68E-25	13835	494	221	POLR1B,ADORA2B,NCK2,APOB,FANCM,FOXA1,LMF1,CHRNA7,TPGS2,PRKX,TRIM33,SMS,GINS1,UBA6,KLHL31,USP49,ZNF286A,ITGB8,CXCL6,SKI,ZNF561,HPDL,SH3GL2,SLC44A5,INO80B,BMPER,TOE1,SMC6,TSEN2,TADA3,HOMER3,ZNF506,CDA,ZNF121,SPIDR,MACROD2,USP24,MEIS2,RANBP2,ADCY4,SOX5,RBM24,TEC,LDLRAD3,DGKB,FAM111B,NRP1,TAOK1,GSPT1,BNIP3L,CTBP1,EEPD1,ZDHHC4,CARS1,XDH,PLK5,POLN,NEMF,SDHB,PHB2,COPS2,MOCS1,RNU6-137P,EOMES,UBQLN2,OSBPL5,GRM2,ADAM30,SH3GLB1,AQP4,ZNF90,ZC3H8,ARL6IP5,INSM1,PGA5,HIC1,EAPP,SNORA59A,SIRPA,TRMT2A,CTNS,AOAH,GLIS1,ALOX12,IP6K1,ARAF,NKX2-8,PRPSAP2,CPNE6,RNF112,MARS1,CHAC2,EEF2KMT,ST18,COMMD1,WDR6,RBBP4,SHOX,TEFM,HDAC6,MIR99AHG,CHAT,PERM1,MPHOSPH8,RCOR2,RNPS1,CELSR2,TNRC6A,NELL1,SCP2,EXTL3,NSFL1C,WNK3,ZNF721,GALK2,LINC00273,DNM1L,BICD1,PTPRO,ULK4,RNF144A,EBF4,NOP9,RAB26,MLST8,STAMBP,SVIP,UBXN2B,IP6K2,RNF216,VCPKMT,IL36RN,FAF1,ADORA2A,MED1,AMY2B,CDC25A,NSD2,KLHDC1,ZNF366,A2ML1,HECTD1,ALG5,SRRM2,CD320,FKBP6,CSDE1,FHIT,ZNF43,ALG8,CMPK1,KDM1B,DPY19L1,C5,USP9X,PPIL4,ABCA4,TENT5A,ZSCAN29,NCEH1,FBXO33,RHBDL2,MIR22HG,OGDH,MARCHF2,LTK,ZNF318,STAT2,MIR124-1HG,SLC25A44,EHD3,PSME2,PER1,CHST12,RPL15,AGXT2,TCF7L1,PLPPR4,MRPS36,SCML2,RNF24,LSM5,DAG1,SMG7,CARNS1,CREB3L1,GBP5,MADD,EPM2AIP1,ZNF676,RARB,SUCLA2,PLPP2,RNF8,PRKAA2,CDK5R1,NEK7,MNT,C3,ZMYND11,CSKMT,POLE,RAF1,CTRC,ITIH5,CALCRL,HARBI1,LIMA1,GALNT14,ATF1,CILK1,ZMPSTE24,HPCA,RPS6KA3,CREB5,PINK1,ZNF143,H4C8,LGSN,ZNF320,FAM168A

signaling	GO:0023052	4.77E-24	6411	487	136	ADORA2B,NCK2,FOXA1,CHRNA7,PRKX,NLGN4X,TRIM33,ARF6,KLHL31,TMEM204,ITGB8,ARHGAP9,CXCL6,SKI,VWF,FCHO1,SH3GL2,TMEM38A,BMPER,TADA3,HOMER3,CDA,ADCY4,ADGRB3,CHRNA2,SYP,TEC,DGKB,OR51E1,NRP1,TAOK1,BCL7B,SDCBP2,BNIP3L,XDH,PLK5,PRAM1,MAD2L1BP,PHB2,SLC15A3,COPS2,UBQLN2,GRM2,CNR2,SIRPB1,ARL6IP5,HIC1,SIRPA,KCNA1,CTNS,REG1A,LPAR3,ARAF,CPNE6,PIRT,ST18,EPOR,C10ORF71,HDAC6,CHAT,PTCHD1,CELSR2,SCN5A,NPR3,OR7E24,C12ORF66,WNK3,FCGR1B,DNM1L,BICD1,RELL1,PTPRO,ULK4,RAB26,MLST8,TXNDC12,STAMBP,ANO6,IL36RN,FAF1,ADORA2A,MED1,BAIAP3,NPHP4,ZNF366,FRMD1,RGS11,OR52I1,PLEKHG4B,CD320,NDUFAF2,FHIT,TMEM108,GRID2IP,GNG7,LZTS1,C5,ARHGAP29,MYO1C,USP9X,MAL,SCN8A,OR5BS1P,ABCA4,NCEH1,BLOC1S6,LTK,STAT2,EHD3,PER1,TCF7L1,PLPPR4,DAG1,CREB3L1,MADD,TBXA2R,SZT2,RARB,PLPP2,PRKAA2,CDK5R1,MNT,C3,ZMYND11,RAF1,RASA4B,CALCRL,CILK1,ZMPSTE24,HPCA,RPS6KA3,TBL2,NPHP1,ZWILCH,KRT18,PINK1

cellular metabolic process	GO:0044237	5.75E-24	12839	494	209	POLR1B,ADORA2B,NCK2,APOB,FANCM,FOXA1,LMF1,CHRNA7,TPGS2,PRKX,TRIM33,SMS,GINS1,UBA6,KLHL31,USP49,ZNF286A,ITGB8,SKI,ZNF561,HPDL,SH3GL2,SLC44A5,INO80B,BMPER,TOE1,SMC6,TSEN2,TADA3,ZNF506,CDA,ZNF121,SPIDR,MACROD2,USP24,MEIS2,RANBP2,ADCY4,SOX5,NDUFS7,RBM24,TEC,DGKB,FAM111B,NRP1,TAOK1,GSPT1,BNIP3L,CTBP1,EEPD1,ZDHHC4,CARS1,XDH,PLK5,POLN,NEMF,SDHB,PHB2,COPS2,MOCS1,RNU6-137P,EOMES,UBQLN2,OSBPL5,SLC17A2,SH3GLB1,ZNF90,ZC3H8,ARL6IP5,INSM1,HIC1,EAPP,SNORA59A,SLC25A23,SIRPA,TRMT2A,CTNS,AOAH,GLIS1,ALOX12,IP6K1,ARAF,NKX2-8,PRPSAP2,RNF112,MARS1,CHAC2,EEF2KMT,ST18,COMMD1,WDR6,RBBP4,SHOX,TEFM,HDAC6,CHAT,PERM1,MPHOSPH8,RCOR2,RNPS1,CELSR2,TNRC6A,NELL1,SCP2,EXTL3,NSFL1C,WNK3,ZNF721,GALK2,DNM1L,BICD1,PTPRO,ULK4,RNF144A,EBF4,NOP9,MLST8,STAMBP,SVIP,BTBD10,UBXN2B,IP6K2,RNF216,VCPKMT,FAF1,ADORA2A,MED1,CDC25A,NSD2,KLHDC1,ZNF366,A2ML1,HECTD1,ALG5,SRRM2,CD320,FKBP6,CSDE1,FHIT,ZNF43,ALG8,CMPK1,KDM1B,LZTS1,DPY19L1,C5,USP9X,PPIL4,ABCA4,TENT5A,ZSCAN29,NCEH1,FBXO33,OGDH,MARCHF2,LTK,ZNF318,STAT2,CHMP3,SLC25A44,EHD3,PSME2,PER1,CHST12,RPL15,AGXT2,TCF7L1,PLPPR4,MRPS36,SCML2,RNF24,LSM5,DAG1,SMG7,CARNS1,CREB3L1,MADD,EPM2AIP1,ZNF676,RARB,SUCLA2,PLPP2,RNF8,PRKAA2,CDK5R1,NEK7,MNT,C3,ZMYND11,CSKMT,POLE,RAF1,CTRC,ITIH5,CALCRL,HARBI1,GALNT14,ATF1,CILK1,ZMPSTE24,HPCA,RPS6KA3,CREB5,PINK1,ZNF143,H4C8,LGSN,ZNF320,FAM168A

primary metabolic process	GO:0044238	5.96E-23	12572	494	204	POLR1B,ADORA2B,NCK2,APOB,FANCM,FOXA1,LMF1,CHRNA7,TPGS2,PRKX,TRIM33,SMS,GINS1,UBA6,KLHL31,USP49,ZNF286A,ITGB8,SKI,ZNF561,HPDL,SH3GL2,SLC44A5,INO80B,BMPER,TOE1,SMC6,TSEN2,TADA3,ZNF506,CDA,ZNF121,SPIDR,MACROD2,USP24,MEIS2,RANBP2,ADCY4,SOX5,RBM24,TEC,LDLRAD3,DGKB,FAM111B,NRP1,TAOK1,GSPT1,BNIP3L,CTBP1,EEPD1,ZDHHC4,CARS1,XDH,PLK5,POLN,NEMF,SDHB,PHB2,COPS2,MOCS1,RNU6-137P,EOMES,UBQLN2,OSBPL5,ADAM30,ZNF90,ZC3H8,ARL6IP5,INSM1,PGA5,HIC1,EAPP,SNORA59A,SIRPA,TRMT2A,CTNS,AOAH,GLIS1,ALOX12,IP6K1,ARAF,NKX2-8,PRPSAP2,CPNE6,RNF112,MARS1,EEF2KMT,ST18,COMMD1,WDR6,SHOX,TEFM,HDAC6,CHAT,PERM1,MPHOSPH8,RCOR2,RNPS1,CELSR2,TNRC6A,NELL1,SCP2,EXTL3,NSFL1C,WNK3,ZNF721,GALK2,BICD1,PTPRO,ULK4,RNF144A,EBF4,NOP9,RAB26,MLST8,STAMBP,SVIP,UBXN2B,IP6K2,RNF216,VCPKMT,FAF1,ADORA2A,MED1,AMY2B,CDC25A,NSD2,KLHDC1,ZNF366,A2ML1,HECTD1,ALG5,SRRM2,FKBP6,CSDE1,FHIT,ZNF43,ALG8,CMPK1,KDM1B,DPY19L1,C5,USP9X,PPIL4,ABCA4,TENT5A,ZSCAN29,NCEH1,FBXO33,RHBDL2,OGDH,MARCHF2,LTK,ZNF318,STAT2,SLC25A44,PSME2,PER1,CHST12,RPL15,AGXT2,TCF7L1,PLPPR4,MRPS36,SCML2,RNF24,LSM5,DAG1,SMG7,CARNS1,CREB3L1,MADD,EPM2AIP1,ZNF676,RARB,SUCLA2,PLPP2,RNF8,PRKAA2,CDK5R1,NEK7,MNT,C3,ZMYND11,CSKMT,POLE,RAF1,CTRC,ITIH5,HARBI1,LIMA1,GALNT14,ATF1,CILK1,ZMPSTE24,HPCA,RPS6KA3,CREB5,PINK1,ZNF143,H4C8,LGSN,ZNF320,FAM168A

negative regulation of biological process	GO:0048519	7.63E-23	5844	489	127	ADORA2B,NCK2,FOXA1,CHRNA7,NLGN4X,TRIM33,ARF6,KLHL31,ITGB8,SKI,SH3GL2,TMEM38A,BMPER,HOMER3,CDA,MEIS2,ADGRB3,RBM24,NRP1,TAOK1,GSPT1,BNIP3L,CTBP1,XDH,PLK5,MAD2L1BP,PHB2,COPS2,EOMES,UBQLN2,CNR2,AQP4,ZC3H8,ARL6IP5,INSM1,HIC1,EAPP,SIRPA,CTNS,AOAH,GLIS1,ALOX12,IP6K1,ARAF,NKX2-8,ST18,COMMD1,WDR6,RBBP4,HDAC6,MIR99AHG,MPHOSPH8,RCOR2,RNPS1,PCM1,PODN,TNRC6A,NELL1,NPR3,C12ORF66,NSFL1C,WNK3,LINC00273,BICD1,PTPRO,TXNDC12,STAMBP,SVIP,BTBD10,UBXN2B,IP6K2,RNF216,IL36RN,THBD,ADORA2A,MED1,NPHP4,NSD2,ZNF366,A2ML1,HECTD1,RGS11,NAA38,FKBP6,NDUFAF2,CSDE1,FHIT,GRID2IP,KDM1B,LZTS1,C5,FRMD5,USP9X,TENT5A,ST7L,MIR22HG,LTK,ZNF318,STAT2,MIR124-1HG,PER1,SCML2,LSM5,DAG1,SMG7,CREB3L1,TBXA2R,SZT2,RARB,RNF8,PRKAA2,CDK5R1,MNT,C3,ZMYND11,RAF1,RASA4B,ITIH5,CALCRL,LIMA1,ZMPSTE24,HPCA,RPS6KA3,ZWILCH,KRT18,PINK1,H4C8
cellular macromolecule metabolic process	GO:0044260	2.50E-22	5746	478	123	NCK2,APOB,FANCM,CHRNA7,TPGS2,PRKX,TRIM33,GINS1,UBA6,KLHL31,USP49,SKI,SH3GL2,INO80B,BMPER,SMC6,TADA3,SPIDR,MACROD2,USP24,RANBP2,RBM24,TEC,FAM111B,NRP1,TAOK1,GSPT1,BNIP3L,CTBP1,EEPD1,ZDHHC4,CARS1,XDH,PLK5,POLN,NEMF,PHB2,COPS2,MOCS1,UBQLN2,SH3GLB1,ARL6IP5,INSM1,SIRPA,TRMT2A,AOAH,ARAF,RNF112,MARS1,EEF2KMT,ST18,COMMD1,WDR6,RBBP4,HDAC6,MPHOSPH8,RCOR2,RNPS1,TNRC6A,NELL1,EXTL3,NSFL1C,WNK3,PTPRO,ULK4,RNF144A,MLST8,STAMBP,SVIP,UBXN2B,RNF216,VCPKMT,FAF1,ADORA2A,CDC25A,NSD2,KLHDC1,A2ML1,HECTD1,ALG5,FKBP6,CSDE1,FHIT,ALG8,KDM1B,DPY19L1,C5,USP9X,PPIL4,TENT5A,NCEH1,FBXO33,OGDH,MARCHF2,LTK,STAT2,EHD3,PSME2,PER1,CHST12,RPL15,RNF24,LSM5,DAG1,SMG7,MADD,EPM2AIP1,RNF8,PRKAA2,CDK5R1,NEK7,C3,CSKMT,POLE,RAF1,ITIH5,CALCRL,GALNT14,ATF1,CILK1,ZMPSTE24,HPCA,RPS6KA3

establishment of localization	GO:0051234	4.98E-22	4920	492	114	ADORA2B,APOB,BEST1,CHRNA7,NLGN4X,ARF6,ANTXR2,FCHO1,SH3GL2,TMEM38A,SLC44A5,HOMER3,TVP23B,SPIDR,DHRS1,ICAM3,RANBP2,CHRNA2,SYP,NDUFS7,LDLRAD3,SLC38A1,NRP1,SDCBP2,KCTD7,BNIP3L,TIMM10,EEPD1,ZDHHC4,XDH,SLC39A3,NEMF,PRAM1,PHB2,SLC15A3,CROCC,UBQLN2,OSBPL5,SLC17A2,TRPC4,GRM2,SH3GLB1,SIRPB1,AQP4,ARL6IP5,EML4,SLC25A23,SIRPA,KCNA1,CTNS,SLC30A2,CPNE6,UNC50,PIRT,COMMD9,COMMD1,LASP1,HDAC6,CHAT,TANGO2,PCM1,CELSR2,SCN5A,SCP2,KCNS3,NPR3,NSFL1C,WNK3,KCNA4,HEPH,DNM1L,BICD1,NOP9,RAB26,SVIP,UBXN2B,IP6K2,ANO6,EXOC6B,ADORA2A,MED1,BAIAP3,NSD2,ATP8B3,SLC5A10,CD320,NDUFAF2,TMEM108,SHROOM2,MYO1C,MAL,SCN8A,ABCA4,BLOC1S6,MARCHF2,CHMP3,SLC25A44,EHD3,PER1,PLPPR4,SMG7,CREB3L1,CDK5R1,C3,RAF1,CALCRL,LIMA1,CILK1,ZMPSTE24,TRAPPC1,HPCA,KRT18,PINK1,SNX31
signal transduction	GO:0007165	5.94E-22	5925	487	126	ADORA2B,NCK2,FOXA1,CHRNA7,PRKX,NLGN4X,TRIM33,ARF6,KLHL31,TMEM204,ITGB8,ARHGAP9,CXCL6,SKI,VWF,FCHO1,SH3GL2,TMEM38A,BMPER,TADA3,HOMER3,CDA,ADCY4,ADGRB3,CHRNA2,SYP,TEC,DGKB,OR51E1,NRP1,TAOK1,BCL7B,SDCBP2,BNIP3L,XDH,PLK5,PRAM1,MAD2L1BP,PHB2,SLC15A3,COPS2,UBQLN2,GRM2,CNR2,SIRPB1,ARL6IP5,HIC1,SIRPA,KCNA1,CTNS,REG1A,LPAR3,ARAF,PIRT,ST18,EPOR,C10ORF71,HDAC6,PTCHD1,CELSR2,NPR3,OR7E24,C12ORF66,WNK3,FCGR1B,DNM1L,BICD1,RELL1,PTPRO,ULK4,MLST8,TXNDC12,STAMBP,ANO6,IL36RN,FAF1,ADORA2A,MED1,BAIAP3,NPHP4,ZNF366,FRMD1,RGS11,OR52I1,PLEKHG4B,CD320,FHIT,TMEM108,GNG7,C5,ARHGAP29,MYO1C,USP9X,MAL,OR5BS1P,ABCA4,NCEH1,LTK,STAT2,PER1,TCF7L1,PLPPR4,DAG1,CREB3L1,MADD,TBXA2R,SZT2,RARB,PLPP2,PRKAA2,CDK5R1,MNT,C3,ZMYND11,RAF1,RASA4B,CALCRL,CILK1,ZMPSTE24,HPCA,RPS6KA3,TBL2,NPHP1,ZWILCH,KRT18,PINK1

regulation of metabolic process	GO:0019222	6.08E-22	7219	494	143	ADORA2B,NCK2,APOB,FANCM,FOXA1,CHRNA7,TRIM33,KLHL31,ZNF286A,ITGB8,CXCL6,SKI,ZNF561,SH3GL2,BMPER,TADA3,HOMER3,ZNF506,CDA,ZNF121,SPIDR,MEIS2,RANBP2,SOX5,RBM24,TEC,LDLRAD3,NRP1,TAOK1,GSPT1,BNIP3L,CTBP1,XDH,PHB2,COPS2,EOMES,UBQLN2,CNR2,SH3GLB1,AQP4,ZNF90,ZC3H8,ARL6IP5,INSM1,HIC1,EAPP,SLC25A23,SIRPA,CTNS,GLIS1,LPAR3,IP6K1,ARAF,NKX2-8,MARS1,ST18,COMMD1,WDR6,SHOX,HDAC6,MIR99AHG,PERM1,MPHOSPH8,RCOR2,RNPS1,CELSR2,TNRC6A,NELL1,SCP2,NPR3,WNK3,ZNF721,LINC00273,DNM1L,PTPRO,RNF144A,EBF4,RAB26,MLST8,SVIP,BTBD10,RNF216,IL36RN,FAF1,ADORA2A,MED1,CDC25A,NSD2,ZNF366,A2ML1,HECTD1,CD320,FKBP6,CSDE1,FHIT,ZNF43,KDM1B,LZTS1,C5,USP9X,PPIL4,TENT5A,ZSCAN29,FBXO33,MIR22HG,MARCHF2,LTK,ZNF318,STAT2,MIR124-1HG,SLC25A44,PSME2,PER1,AGXT2,TCF7L1,SCML2,LSM5,DAG1,SMG7,CREB3L1,GBP5,MADD,EPM2AIP1,ZNF676,RARB,RNF8,PRKAA2,CDK5R1,NEK7,MNT,C3,ZMYND11,RAF1,ITIH5,ATF1,ZMPSTE24,HPCA,RPS6KA3,CREB5,PINK1,ZNF143,ZNF320,FAM168A
organonitrogen compound metabolic process	GO:1901564	1.11E-21	6338	478	129	ADORA2B,NCK2,APOB,FANCM,LMF1,CHRNA7,TPGS2,PRKX,TRIM33,SMS,UBA6,KLHL31,USP49,ITGB8,SKI,HPDL,SH3GL2,SLC44A5,BMPER,TADA3,CDA,MACROD2,USP24,RANBP2,ADCY4,RBM24,TEC,LDLRAD3,FAM111B,NRP1,TAOK1,GSPT1,BNIP3L,CTBP1,ZDHHC4,CARS1,XDH,PLK5,NEMF,PHB2,COPS2,MOCS1,UBQLN2,OSBPL5,ADAM30,ARL6IP5,INSM1,PGA5,SIRPA,CTNS,ARAF,PRPSAP2,RNF112,MARS1,CHAC2,EEF2KMT,ST18,COMMD1,HDAC6,CHAT,RCOR2,TNRC6A,NELL1,EXTL3,NSFL1C,WNK3,PTPRO,ULK4,RNF144A,RAB26,MLST8,STAMBP,SVIP,UBXN2B,RNF216,VCPKMT,FAF1,ADORA2A,MED1,CDC25A,NSD2,KLHDC1,A2ML1,HECTD1,ALG5,CD320,FKBP6,CSDE1,FHIT,ALG8,CMPK1,KDM1B,DPY19L1,C5,USP9X,PPIL4,NCEH1,FBXO33,RHBDL2,OGDH,MARCHF2,LTK,STAT2,SLC25A44,PSME2,PER1,CHST12,RPL15,AGXT2,RNF24,DAG1,CARNS1,MADD,SUCLA2,PLPP2,RNF8,PRKAA2,CDK5R1,NEK7,C3,CSKMT,RAF1,CTRC,ITIH5,GALNT14,CILK1,ZMPSTE24,HPCA,RPS6KA3

negative regulation of cellular process	GO:0048523	2.34E-21	4818	489	111	NCK2,FOXA1,CHRNA7,NLGN4X,TRIM33,ARF6,KLHL31,SKI,SH3GL2,TMEM38A,BMPER,HOMER3,CDA,MEIS2,RBM24,NRP1,TAOK1,BNIP3L,CTBP1,XDH,PLK5,MAD2L1BP,PHB2,COPS2,EOMES,UBQLN2,CNR2,AQP4,ZC3H8,ARL6IP5,INSM1,HIC1,EAPP,SIRPA,CTNS,GLIS1,ALOX12,ARAF,NKX2-8,ST18,COMMD1,WDR6,RBBP4,HDAC6,MPHOSPH8,RCOR2,RNPS1,PCM1,PODN,TNRC6A,NELL1,NPR3,C12ORF66,NSFL1C,WNK3,BICD1,PTPRO,TXNDC12,STAMBP,SVIP,BTBD10,UBXN2B,IP6K2,IL36RN,THBD,ADORA2A,MED1,NPHP4,NSD2,ZNF366,A2ML1,HECTD1,RGS11,NAA38,NDUFAF2,FHIT,GRID2IP,KDM1B,LZTS1,C5,FRMD5,USP9X,TENT5A,ST7L,LTK,ZNF318,STAT2,PER1,SCML2,DAG1,CREB3L1,TBXA2R,SZT2,RARB,RNF8,PRKAA2,CDK5R1,MNT,C3,ZMYND11,RAF1,RASA4B,ITIH5,LIMA1,ZMPSTE24,HPCA,RPS6KA3,ZWILCH,KRT18,PINK1,H4C8
regulation of response to stimulus	GO:0048583	4.70E-20	3841	494	96	ADORA2B,NCK2,FOXA1,CHRNA7,NLGN4X,TRIM33,ARF6,KLHL31,TMEM204,ARHGAP9,CXCL6,SKI,VWF,FCHO1,BMPER,HOMER3,SPIDR,SYP,TEC,NRP1,TAOK1,XDH,PRAM1,MAD2L1BP,PHB2,SLC15A3,UBQLN2,GRM2,CNR2,ARL6IP5,HIC1,SLC25A23,SIRPA,CTNS,AOAH,C8B,LPAR3,ALOX12,ARAF,C10ORF71,HDAC6,SCN5A,C12ORF66,FCGR1B,DNM1L,BICD1,RELL1,PTPRO,CFP,ULK4,MLST8,TXNDC12,STAMBP,SVIP,ANO6,RNF216,IL36RN,FAF1,THBD,ADORA2A,MED1,NPHP4,NSD2,ZNF366,FRMD1,RGS11,PLEKHG4B,NDUFAF2,TMEM108,GNG7,C5,ARHGAP29,MYO1C,MAL,STAT2,PER1,TCF7L1,DAG1,CREB3L1,GBP5,MADD,TBXA2R,SZT2,RNF8,PRKAA2,CDK5R1,MNT,C3,ZMYND11,RAF1,RASA4B,CALCRL,ZMPSTE24,RPS6KA3,PINK1,FAM168A

nitrogen compound metabolic process	GO:0006807	1.36E-19	12062	494	191	POLR1B,ADORA2B,NCK2,APOB,FANCM,FOXA1,LMF1,CHRNA7,TPGS2,PRKX,TRIM33,SMS,GINS1,UBA6,KLHL31,USP49,ZNF286A,ITGB8,SKI,ZNF561,HPDL,SH3GL2,SLC44A5,INO80B,BMPER,TOE1,SMC6,TSEN2,TADA3,ZNF506,CDA,ZNF121,SPIDR,MACROD2,USP24,MEIS2,RANBP2,ADCY4,SOX5,RBM24,TEC,LDLRAD3,FAM111B,NRP1,TAOK1,GSPT1,BNIP3L,CTBP1,EEPD1,ZDHHC4,CARS1,XDH,PLK5,POLN,NEMF,PHB2,COPS2,MOCS1,RNU6-137P,EOMES,UBQLN2,OSBPL5,ADAM30,ZNF90,ZC3H8,ARL6IP5,INSM1,PGA5,HIC1,EAPP,SNORA59A,SIRPA,TRMT2A,CTNS,GLIS1,ARAF,NKX2-8,PRPSAP2,RNF112,MARS1,CHAC2,EEF2KMT,ST18,COMMD1,WDR6,SHOX,TEFM,HDAC6,CHAT,PERM1,MPHOSPH8,RCOR2,RNPS1,CELSR2,TNRC6A,NELL1,EXTL3,NSFL1C,WNK3,ZNF721,BICD1,PTPRO,ULK4,RNF144A,EBF4,NOP9,RAB26,MLST8,STAMBP,SVIP,UBXN2B,RNF216,VCPKMT,FAF1,ADORA2A,MED1,CDC25A,NSD2,KLHDC1,ZNF366,A2ML1,HECTD1,ALG5,SRRM2,CD320,FKBP6,CSDE1,FHIT,ZNF43,ALG8,CMPK1,KDM1B,DPY19L1,C5,USP9X,PPIL4,TENT5A,ZSCAN29,NCEH1,FBXO33,RHBDL2,OGDH,MARCHF2,LTK,ZNF318,STAT2,SLC25A44,PSME2,PER1,CHST12,RPL15,AGXT2,TCF7L1,SCML2,RNF24,LSM5,DAG1,SMG7,CARNS1,CREB3L1,MADD,ZNF676,RARB,SUCLA2,PLPP2,RNF8,PRKAA2,CDK5R1,NEK7,MNT,C3,ZMYND11,CSKMT,POLE,RAF1,CTRC,ITIH5,HARBI1,GALNT14,ATF1,CILK1,ZMPSTE24,HPCA,RPS6KA3,CREB5,PINK1,ZNF143,H4C8,LGSN,ZNF320,FAM168A
transport	GO:0006810	2.19E-19	4760	492	107	ADORA2B,APOB,BEST1,CHRNA7,NLGN4X,ARF6,ANTXR2,FCHO1,SH3GL2,TMEM38A,SLC44A5,HOMER3,TVP23B,DHRS1,ICAM3,RANBP2,CHRNA2,SYP,NDUFS7,LDLRAD3,SLC38A1,NRP1,SDCBP2,KCTD7,BNIP3L,TIMM10,EEPD1,ZDHHC4,XDH,SLC39A3,NEMF,PRAM1,PHB2,SLC15A3,UBQLN2,OSBPL5,SLC17A2,TRPC4,GRM2,SH3GLB1,SIRPB1,AQP4,ARL6IP5,SLC25A23,SIRPA,KCNA1,CTNS,SLC30A2,CPNE6,UNC50,PIRT,COMMD9,COMMD1,LASP1,HDAC6,CHAT,TANGO2,PCM1,CELSR2,SCN5A,SCP2,KCNS3,NPR3,WNK3,KCNA4,HEPH,DNM1L,BICD1,NOP9,RAB26,SVIP,IP6K2,ANO6,EXOC6B,ADORA2A,MED1,BAIAP3,ATP8B3,SLC5A10,CD320,NDUFAF2,TMEM108,SHROOM2,MYO1C,SCN8A,ABCA4,BLOC1S6,MARCHF2,CHMP3,SLC25A44,EHD3,PER1,PLPPR4,SMG7,CREB3L1,CDK5R1,C3,RAF1,CALCRL,LIMA1,CILK1,ZMPSTE24,TRAPPC1,HPCA,KRT18,PINK1,SNX31

regulation of cellular metabolic process	GO:0031323	7.47E-19	5921	494	121	ADORA2B,NCK2,FANCM,FOXA1,CHRNA7,TRIM33,KLHL31,ZNF286A,SKI,ZNF561,SH3GL2,BMPER,TADA3,ZNF506,CDA,ZNF121,SPIDR,MEIS2,RANBP2,SOX5,RBM24,TEC,NRP1,TAOK1,GSPT1,BNIP3L,CTBP1,XDH,PHB2,COPS2,EOMES,UBQLN2,SH3GLB1,ZNF90,ZC3H8,ARL6IP5,INSM1,HIC1,EAPP,SLC25A23,SIRPA,CTNS,GLIS1,ARAF,NKX2-8,MARS1,ST18,COMMD1,WDR6,SHOX,HDAC6,PERM1,MPHOSPH8,RCOR2,RNPS1,CELSR2,TNRC6A,NELL1,SCP2,WNK3,ZNF721,DNM1L,PTPRO,RNF144A,EBF4,MLST8,SVIP,BTBD10,FAF1,ADORA2A,MED1,CDC25A,NSD2,ZNF366,A2ML1,HECTD1,CSDE1,FHIT,ZNF43,KDM1B,LZTS1,C5,USP9X,PPIL4,TENT5A,ZSCAN29,FBXO33,MARCHF2,LTK,ZNF318,STAT2,PSME2,PER1,AGXT2,TCF7L1,SCML2,DAG1,SMG7,CREB3L1,MADD,EPM2AIP1,ZNF676,RARB,RNF8,PRKAA2,CDK5R1,NEK7,MNT,C3,ZMYND11,RAF1,ITIH5,ATF1,ZMPSTE24,HPCA,RPS6KA3,CREB5,PINK1,ZNF143,ZNF320,FAM168A
developmental process	GO:0032502	2.56E-18	6326	489	124	POLR1B,HAPLN1,NCK2,APOB,FOXA1,ZPBP,CHRNA7,PRKX,NLGN4X,ARF6,GINS1,UBA6,TMEM204,ITGB8,SKI,SH3GL2,BMPER,SMC6,SPAG11A,DRC1,BFSP1,MACROD2,MEIS2,SOX5,SPATA6L,ADGRB3,SYP,RBM24,TEC,NRP1,TAOK1,BCL7B,SDCBP2,CTBP1,XDH,PLK5,SLC39A3,ARMC6,PHB2,STRIP1,COPS2,EOMES,TRPC4,ZC3H8,INSM1,KCNA1,CTNS,GLIS1,LPAR3,ALOX12,ANLN,NKX2-8,PRPSAP2,CPNE6,RNF112,NEXN,GPRIN2,EPOR,SHOX,HDAC6,KRTAP4-5,PTCHD1,PCM1,CELSR2,SCN5A,NELL1,NPR3,DNM1L,BICD1,PTPRO,ULK4,RAB26,ANO6,TPM4,ADORA2A,MED1,KRT3,BAIAP3,DHX30,NPHP4,NSD2,HECTD1,ALG5,PLAC1,VIT,FKBP6,CSDE1,TMEM108,SHROOM2,LZTS1,C5,TSGA10,LGI2,USP9X,MAL,SCN8A,OGDH,BLOC1S6,LTK,STAT2,PLPPR4,SCML2,DAG1,CREB3L1,TBXA2R,SZT2,RARB,RNF8,CDK5R1,MNT,C3,POLE,RAF1,FMNL3,CALCRL,ATF1,ZMPSTE24,HPCA,RPS6KA3,NPHP1,SPATA16,KRT18,PINK1,H4C8

macromolecule metabolic process	GO:0043170	4.54E-18	12328	494	190	POLR1B,ADORA2B,NCK2,APOB,FANCM,FOXA1,LMF1,CHRNA7,TPGS2,PRKX,TRIM33,GINS1,UBA6,KLHL31,USP49,ZNF286A,ITGB8,CXCL6,SKI,ZNF561,SH3GL2,INO80B,BMPER,TOE1,SMC6,TSEN2,TADA3,HOMER3,ZNF506,ZNF121,SPIDR,MACROD2,USP24,MEIS2,RANBP2,SOX5,RBM24,TEC,LDLRAD3,FAM111B,NRP1,TAOK1,GSPT1,BNIP3L,CTBP1,EEPD1,ZDHHC4,CARS1,XDH,PLK5,POLN,NEMF,PHB2,COPS2,MOCS1,RNU6-137P,EOMES,UBQLN2,GRM2,ADAM30,SH3GLB1,AQP4,ZNF90,ZC3H8,ARL6IP5,INSM1,PGA5,HIC1,EAPP,SNORA59A,SIRPA,TRMT2A,AOAH,GLIS1,ARAF,NKX2-8,RNF112,MARS1,EEF2KMT,ST18,COMMD1,WDR6,RBBP4,SHOX,TEFM,HDAC6,MIR99AHG,PERM1,MPHOSPH8,RCOR2,RNPS1,CELSR2,TNRC6A,NELL1,EXTL3,NSFL1C,WNK3,ZNF721,LINC00273,DNM1L,BICD1,PTPRO,ULK4,RNF144A,EBF4,NOP9,RAB26,MLST8,STAMBP,SVIP,UBXN2B,RNF216,VCPKMT,IL36RN,FAF1,ADORA2A,MED1,CDC25A,NSD2,KLHDC1,ZNF366,A2ML1,HECTD1,ALG5,SRRM2,FKBP6,CSDE1,FHIT,ZNF43,ALG8,KDM1B,DPY19L1,C5,USP9X,PPIL4,TENT5A,ZSCAN29,NCEH1,FBXO33,RHBDL2,MIR22HG,OGDH,MARCHF2,LTK,ZNF318,STAT2,MIR124-1HG,EHD3,PSME2,PER1,CHST12,RPL15,TCF7L1,SCML2,RNF24,LSM5,DAG1,SMG7,CREB3L1,GBP5,MADD,EPM2AIP1,ZNF676,RARB,RNF8,PRKAA2,CDK5R1,NEK7,MNT,C3,ZMYND11,CSKMT,POLE,RAF1,CTRC,ITIH5,CALCRL,HARBI1,GALNT14,ATF1,CILK1,ZMPSTE24,HPCA,RPS6KA3,CREB5,PINK1,ZNF143,H4C8,ZNF320,FAM168A

cellular component organization	GO:0016043	2.15E-17	7793	489	139	POLR1B,HLA-DQA2,NCK2,APOB,FANCM,FOXA1,ZPBP,CHRNA7,NLGN4X,ARF6,UBA6,CCDC6,USP49,FCHO1,SH3GL2,TMEM38A,INO80B,SMC6,DRC1,CDA,BFSP1,TTC19,SPIDR,DHRS1,ADGRB3,SYP,NDUFS7,DGKB,NRP1,TAOK1,DSG1,GSPT1,CEP72,KCTD7,BNIP3L,TIMM10,XDH,PLK5,FSCN1,MAD2L1BP,PHB2,STRIP1,RNU6-137P,CROCC,UBQLN2,OSBPL5,SH3GLB1,AQP4,INSM1,EML4,KCNA1,LPAR3,ANLN,NKX2-8,CPNE6,RNF112,NEXN,GPRIN2,RBBP4,HDAC6,MPHOSPH8,TANGO2,TJAP1,PCM1,CELSR2,SCP2,KCNS3,EXTL3,NSFL1C,WNK3,KCNA4,DNM1L,BICD1,PTPRO,ULK4,MLST8,MPP7,GOLGA8B,SVIP,UBXN2B,IP6K2,ANO6,TPM4,FAF1,EXOC6B,ADORA2A,KRT3,BAIAP3,DHX30,NPHP4,NSD2,COX19,ATP8B3,VIT,NDUFAF2,CSDE1,RAD54L2,TMEM108,KDM1B,SHROOM2,LZTS1,FRMD5,LGI2,MYO1C,USP9X,MAL,ABCA4,ST7L,BLOC1S6,LTK,STAT2,CHMP3,EHD3,TCF7L1,PLPPR4,DAG1,CREB3L1,GBP5,SZT2,STAG1,RNF8,PRKAA2,CDK5R1,NEK7,C3,ZMYND11,RAF1,FMNL3,LIMA1,ATF1,CILK1,ZMPSTE24,HPCA,RPS6KA3,NPHP1,ZWILCH,KRT18,PINK1,H4C8
cellular biosynthetic process	GO:0044249	2.96E-17	5794	493	116	POLR1B,ADORA2B,NCK2,APOB,FANCM,FOXA1,TRIM33,SMS,GINS1,ZNF286A,SKI,ZNF561,SLC44A5,TADA3,ZNF506,CDA,ZNF121,MEIS2,ADCY4,SOX5,RBM24,DGKB,FAM111B,GSPT1,CTBP1,ZDHHC4,CARS1,POLN,NEMF,PHB2,COPS2,MOCS1,EOMES,ZNF90,ZC3H8,INSM1,HIC1,EAPP,SIRPA,CTNS,GLIS1,ALOX12,IP6K1,NKX2-8,PRPSAP2,MARS1,CHAC2,ST18,COMMD1,RBBP4,SHOX,TEFM,HDAC6,CHAT,PERM1,MPHOSPH8,RCOR2,RNPS1,CELSR2,TNRC6A,SCP2,EXTL3,ZNF721,EBF4,SVIP,IP6K2,FAF1,ADORA2A,MED1,NSD2,ZNF366,ALG5,CSDE1,ZNF43,ALG8,CMPK1,KDM1B,DPY19L1,USP9X,PPIL4,ZSCAN29,ZNF318,STAT2,PER1,CHST12,RPL15,AGXT2,TCF7L1,SCML2,CARNS1,CREB3L1,EPM2AIP1,ZNF676,RARB,SUCLA2,PLPP2,RNF8,PRKAA2,CDK5R1,NEK7,MNT,C3,ZMYND11,POLE,RAF1,GALNT14,ATF1,ZMPSTE24,HPCA,RPS6KA3,CREB5,PINK1,ZNF143,H4C8,LGSN,ZNF320

protein metabolic process	GO:0019538	7.32E-17	5389	478	108	NCK2,APOB,FANCM,LMF1,CHRNA7,TPGS2,PRKX,TRIM33,UBA6,KLHL31,USP49,SKI,SH3GL2,BMPER,TADA3,MACROD2,USP24,RANBP2,RBM24,TEC,LDLRAD3,FAM111B,NRP1,TAOK1,GSPT1,BNIP3L,CTBP1,ZDHHC4,CARS1,XDH,PLK5,NEMF,PHB2,COPS2,MOCS1,UBQLN2,ADAM30,ARL6IP5,INSM1,PGA5,SIRPA,ARAF,RNF112,MARS1,EEF2KMT,ST18,COMMD1,HDAC6,RCOR2,TNRC6A,NELL1,EXTL3,NSFL1C,WNK3,PTPRO,ULK4,RNF144A,RAB26,MLST8,STAMBP,SVIP,UBXN2B,RNF216,VCPKMT,FAF1,ADORA2A,CDC25A,NSD2,KLHDC1,A2ML1,HECTD1,ALG5,FKBP6,CSDE1,FHIT,ALG8,KDM1B,DPY19L1,C5,USP9X,PPIL4,NCEH1,FBXO33,RHBDL2,OGDH,MARCHF2,LTK,STAT2,PSME2,PER1,CHST12,RPL15,RNF24,DAG1,MADD,RNF8,PRKAA2,CDK5R1,NEK7,C3,CSKMT,RAF1,CTRC,ITIH5,GALNT14,CILK1,ZMPSTE24,RPS6KA3
cellular component organization or biogenesis	GO:0071840	7.65E-17	7994	489	140	POLR1B,HLA-DQA2,NCK2,APOB,FANCM,FOXA1,ZPBP,CHRNA7,NLGN4X,ARF6,UBA6,CCDC6,USP49,FCHO1,SH3GL2,TMEM38A,INO80B,SMC6,DRC1,CDA,BFSP1,TTC19,SPIDR,DHRS1,ADGRB3,SYP,NDUFS7,DGKB,NRP1,TAOK1,DSG1,GSPT1,CEP72,KCTD7,BNIP3L,TIMM10,XDH,PLK5,FSCN1,MAD2L1BP,PHB2,STRIP1,RNU6-137P,CROCC,UBQLN2,OSBPL5,SH3GLB1,AQP4,INSM1,EML4,KCNA1,LPAR3,ANLN,NKX2-8,CPNE6,RNF112,NEXN,GPRIN2,RBBP4,HDAC6,MPHOSPH8,TANGO2,TJAP1,PCM1,CELSR2,SCP2,KCNS3,EXTL3,NSFL1C,WNK3,KCNA4,DNM1L,BICD1,PTPRO,ULK4,NOP9,MLST8,MPP7,GOLGA8B,SVIP,UBXN2B,IP6K2,ANO6,TPM4,FAF1,EXOC6B,ADORA2A,KRT3,BAIAP3,DHX30,NPHP4,NSD2,COX19,ATP8B3,VIT,NDUFAF2,CSDE1,RAD54L2,TMEM108,KDM1B,SHROOM2,LZTS1,FRMD5,LGI2,MYO1C,USP9X,MAL,ABCA4,ST7L,BLOC1S6,LTK,STAT2,CHMP3,EHD3,TCF7L1,PLPPR4,DAG1,CREB3L1,GBP5,SZT2,STAG1,RNF8,PRKAA2,CDK5R1,NEK7,C3,ZMYND11,RAF1,FMNL3,LIMA1,ATF1,CILK1,ZMPSTE24,HPCA,RPS6KA3,NPHP1,ZWILCH,KRT18,PINK1,H4C8

organic substance biosynthetic process	GO:1901576	8.08E-17	5869	493	116	POLR1B,ADORA2B,NCK2,APOB,FANCM,FOXA1,TRIM33,SMS,GINS1,ZNF286A,SKI,ZNF561,SLC44A5,TADA3,ZNF506,CDA,ZNF121,MEIS2,RANBP2,ADCY4,SOX5,RBM24,DGKB,FAM111B,GSPT1,CTBP1,ZDHHC4,CARS1,POLN,NEMF,PHB2,COPS2,MOCS1,EOMES,ZNF90,ZC3H8,INSM1,HIC1,EAPP,CTNS,GLIS1,ALOX12,IP6K1,NKX2-8,PRPSAP2,MARS1,CHAC2,ST18,COMMD1,RBBP4,SHOX,TEFM,HDAC6,CHAT,PERM1,MPHOSPH8,RCOR2,RNPS1,CELSR2,TNRC6A,SCP2,EXTL3,ZNF721,EBF4,SVIP,IP6K2,FAF1,ADORA2A,MED1,NSD2,ZNF366,ALG5,CSDE1,ZNF43,ALG8,CMPK1,KDM1B,DPY19L1,USP9X,PPIL4,ZSCAN29,ZNF318,STAT2,PER1,CHST12,RPL15,AGXT2,TCF7L1,SCML2,CARNS1,CREB3L1,EPM2AIP1,ZNF676,RARB,SUCLA2,PLPP2,RNF8,PRKAA2,CDK5R1,NEK7,MNT,C3,ZMYND11,POLE,RAF1,GALNT14,ATF1,ZMPSTE24,HPCA,RPS6KA3,CREB5,PINK1,ZNF143,H4C8,LGSN,ZNF320
biosynthetic process	GO:0009058	8.57E-17	5956	493	117	POLR1B,ADORA2B,NCK2,APOB,FANCM,FOXA1,TRIM33,SMS,GINS1,ZNF286A,SKI,ZNF561,SLC44A5,TADA3,ZNF506,CDA,ZNF121,MEIS2,RANBP2,ADCY4,SOX5,RBM24,DGKB,FAM111B,GSPT1,CTBP1,ZDHHC4,CARS1,POLN,NEMF,PHB2,COPS2,MOCS1,EOMES,ZNF90,ZC3H8,INSM1,HIC1,EAPP,SIRPA,CTNS,GLIS1,ALOX12,IP6K1,NKX2-8,PRPSAP2,MARS1,CHAC2,ST18,COMMD1,RBBP4,SHOX,TEFM,HDAC6,CHAT,PERM1,MPHOSPH8,RCOR2,RNPS1,CELSR2,TNRC6A,SCP2,EXTL3,ZNF721,EBF4,SVIP,IP6K2,FAF1,ADORA2A,MED1,NSD2,ZNF366,ALG5,CSDE1,ZNF43,ALG8,CMPK1,KDM1B,DPY19L1,USP9X,PPIL4,ZSCAN29,ZNF318,STAT2,PER1,CHST12,RPL15,AGXT2,TCF7L1,SCML2,CARNS1,CREB3L1,EPM2AIP1,ZNF676,RARB,SUCLA2,PLPP2,RNF8,PRKAA2,CDK5R1,NEK7,MNT,C3,ZMYND11,POLE,RAF1,GALNT14,ATF1,ZMPSTE24,HPCA,RPS6KA3,CREB5,PINK1,ZNF143,H4C8,LGSN,ZNF320
regulation of primary metabolic process	GO:0080090	2.47E-16	5694	494	113	ADORA2B,NCK2,APOB,FANCM,FOXA1,CHRNA7,TRIM33,KLHL31,ZNF286A,SKI,ZNF561,SH3GL2,BMPER,TADA3,ZNF506,CDA,ZNF121,SPIDR,MEIS2,RANBP2,SOX5,RBM24,TEC,LDLRAD3,NRP1,TAOK1,GSPT1,CTBP1,XDH,PHB2,COPS2,EOMES,UBQLN2,ZNF90,ZC3H8,ARL6IP5,INSM1,HIC1,EAPP,SIRPA,GLIS1,ARAF,NKX2-8,MARS1,ST18,COMMD1,SHOX,HDAC6,PERM1,MPHOSPH8,RCOR2,RNPS1,CELSR2,TNRC6A,NELL1,SCP2,WNK3,ZNF721,PTPRO,RNF144A,EBF4,RAB26,MLST8,SVIP,FAF1,ADORA2A,MED1,CDC25A,NSD2,ZNF366,A2ML1,HECTD1,CSDE1,FHIT,ZNF43,KDM1B,C5,USP9X,PPIL4,TENT5A,ZSCAN29,FBXO33,MARCHF2,ZNF318,STAT2,PSME2,PER1,TCF7L1,SCML2,DAG1,CREB3L1,MADD,EPM2AIP1,ZNF676,RARB,RNF8,PRKAA2,CDK5R1,NEK7,MNT,C3,ZMYND11,RAF1,ITIH5,ATF1,ZMPSTE24,HPCA,RPS6KA3,CREB5,PINK1,ZNF143,ZNF320,FAM168A

regulation of macromolecule metabolic process	GO:0060255	2.84E-16	6710	494	125	ADORA2B,NCK2,APOB,FANCM,FOXA1,CHRNA7,TRIM33,KLHL31,ZNF286A,ITGB8,CXCL6,SKI,ZNF561,SH3GL2,BMPER,TADA3,HOMER3,ZNF506,ZNF121,SPIDR,MEIS2,SOX5,RBM24,TEC,LDLRAD3,NRP1,TAOK1,GSPT1,CTBP1,XDH,PHB2,COPS2,EOMES,UBQLN2,AQP4,ZNF90,ZC3H8,ARL6IP5,INSM1,HIC1,EAPP,SIRPA,GLIS1,ARAF,NKX2-8,MARS1,ST18,COMMD1,SHOX,HDAC6,MIR99AHG,PERM1,MPHOSPH8,RCOR2,RNPS1,CELSR2,TNRC6A,NELL1,WNK3,ZNF721,LINC00273,DNM1L,PTPRO,RNF144A,EBF4,RAB26,MLST8,SVIP,RNF216,IL36RN,FAF1,ADORA2A,MED1,CDC25A,NSD2,ZNF366,A2ML1,HECTD1,FKBP6,CSDE1,FHIT,ZNF43,KDM1B,C5,USP9X,PPIL4,TENT5A,ZSCAN29,FBXO33,MIR22HG,MARCHF2,ZNF318,STAT2,MIR124-1HG,PSME2,PER1,TCF7L1,SCML2,LSM5,DAG1,SMG7,CREB3L1,GBP5,MADD,EPM2AIP1,ZNF676,RARB,RNF8,PRKAA2,CDK5R1,NEK7,MNT,C3,ZMYND11,RAF1,ITIH5,ATF1,ZMPSTE24,HPCA,RPS6KA3,CREB5,PINK1,ZNF143,ZNF320,FAM168A
regulation of localization	GO:0032879	4.60E-16	2701	487	72	ADORA2B,APOB,BEST1,PRKX,ARF6,TMEM38A,BMPER,DRC1,HOMER3,SPIDR,SYP,NRP1,CEP72,KCTD7,BNIP3L,EEPD1,PRAM1,PHB2,CROCC,UBQLN2,GRM2,SH3GLB1,SIRPB1,ARL6IP5,INSM1,SLC25A23,SIRPA,KCNA1,SLC30A2,NEXN,PIRT,COMMD1,HDAC6,PCM1,PODN,CELSR2,SCN5A,SCP2,KCNS3,NSFL1C,EFCAB1,WNK3,KCNA4,DNM1L,BICD1,PTPRO,ULK4,RAB26,SVIP,UBXN2B,ANO6,ADORA2A,BAIAP3,NSD2,HECTD1,NDUFAF2,C5,FRMD5,MYO1C,SCN8A,CHMP3,EHD3,PER1,DAG1,TBXA2R,PRKAA2,CDK5R1,C3,RAF1,ZMPSTE24,HPCA,PINK1
regulation of nitrogen compound metabolic process	GO:0051171	7.18E-16	5529	494	110	ADORA2B,NCK2,FANCM,FOXA1,CHRNA7,TRIM33,KLHL31,ZNF286A,SKI,ZNF561,SH3GL2,BMPER,TADA3,ZNF506,CDA,ZNF121,SPIDR,MEIS2,SOX5,RBM24,TEC,LDLRAD3,NRP1,TAOK1,GSPT1,CTBP1,XDH,PHB2,COPS2,EOMES,UBQLN2,ZNF90,ZC3H8,ARL6IP5,INSM1,HIC1,EAPP,SIRPA,GLIS1,ARAF,NKX2-8,MARS1,ST18,COMMD1,SHOX,HDAC6,PERM1,MPHOSPH8,RCOR2,RNPS1,CELSR2,TNRC6A,NELL1,WNK3,ZNF721,PTPRO,RNF144A,EBF4,RAB26,MLST8,SVIP,FAF1,ADORA2A,MED1,CDC25A,NSD2,ZNF366,A2ML1,HECTD1,CSDE1,FHIT,ZNF43,KDM1B,C5,USP9X,PPIL4,TENT5A,ZSCAN29,FBXO33,MARCHF2,ZNF318,STAT2,PSME2,PER1,AGXT2,TCF7L1,SCML2,DAG1,CREB3L1,MADD,ZNF676,RARB,RNF8,PRKAA2,CDK5R1,NEK7,MNT,C3,ZMYND11,RAF1,ITIH5,ATF1,ZMPSTE24,HPCA,RPS6KA3,CREB5,PINK1,ZNF143,ZNF320,FAM168A

anatomical structure development	GO:0048856	1.30E-15	5718	489	111	POLR1B,HAPLN1,NCK2,APOB,FOXA1,ZPBP,CHRNA7,PRKX,NLGN4X,ARF6,GINS1,UBA6,TMEM204,ITGB8,SKI,SH3GL2,BMPER,DRC1,BFSP1,MACROD2,MEIS2,SOX5,ADGRB3,RBM24,TEC,NRP1,TAOK1,SDCBP2,XDH,PLK5,SLC39A3,ARMC6,PHB2,STRIP1,COPS2,EOMES,TRPC4,ZC3H8,INSM1,KCNA1,CTNS,LPAR3,ALOX12,ANLN,NKX2-8,PRPSAP2,CPNE6,RNF112,NEXN,GPRIN2,EPOR,SHOX,HDAC6,PTCHD1,PCM1,CELSR2,SCN5A,NELL1,NPR3,DNM1L,BICD1,PTPRO,ULK4,RAB26,ANO6,ADORA2A,MED1,KRT3,DHX30,NPHP4,NSD2,HECTD1,ALG5,PLAC1,VIT,CSDE1,TMEM108,SHROOM2,LZTS1,C5,LGI2,USP9X,MAL,SCN8A,OGDH,BLOC1S6,LTK,STAT2,PLPPR4,SCML2,DAG1,CREB3L1,TBXA2R,SZT2,RARB,RNF8,CDK5R1,MNT,C3,POLE,RAF1,FMNL3,CALCRL,ATF1,ZMPSTE24,HPCA,RPS6KA3,NPHP1,KRT18,PINK1,H4C8
cellular protein metabolic process	GO:0044267	1.68E-15	4796	478	98	NCK2,FANCM,CHRNA7,TPGS2,PRKX,TRIM33,UBA6,KLHL31,USP49,SKI,SH3GL2,BMPER,TADA3,MACROD2,USP24,RANBP2,RBM24,TEC,NRP1,TAOK1,GSPT1,BNIP3L,CTBP1,ZDHHC4,CARS1,XDH,PLK5,NEMF,PHB2,COPS2,MOCS1,UBQLN2,ARL6IP5,INSM1,SIRPA,ARAF,RNF112,MARS1,EEF2KMT,ST18,COMMD1,HDAC6,RCOR2,TNRC6A,NELL1,EXTL3,NSFL1C,WNK3,PTPRO,ULK4,RNF144A,MLST8,STAMBP,SVIP,UBXN2B,RNF216,VCPKMT,FAF1,ADORA2A,CDC25A,NSD2,KLHDC1,A2ML1,HECTD1,ALG5,FKBP6,CSDE1,FHIT,ALG8,KDM1B,DPY19L1,C5,USP9X,PPIL4,NCEH1,FBXO33,OGDH,MARCHF2,LTK,STAT2,PSME2,PER1,RPL15,RNF24,DAG1,MADD,RNF8,PRKAA2,CDK5R1,NEK7,C3,CSKMT,RAF1,ITIH5,GALNT14,CILK1,ZMPSTE24,RPS6KA3
regulation of signaling	GO:0023051	2.58E-15	3327	461	77	ADORA2B,NCK2,FOXA1,CHRNA7,NLGN4X,TRIM33,ARF6,KLHL31,TMEM204,ARHGAP9,SKI,VWF,BMPER,HOMER3,SYP,DGKB,NRP1,TAOK1,XDH,MAD2L1BP,PHB2,SLC15A3,UBQLN2,GRM2,CNR2,ARL6IP5,HIC1,SIRPA,CTNS,LPAR3,ARAF,C10ORF71,HDAC6,C12ORF66,DNM1L,BICD1,RELL1,PTPRO,ULK4,RAB26,MLST8,TXNDC12,STAMBP,IL36RN,FAF1,ADORA2A,MED1,BAIAP3,NPHP4,ZNF366,FRMD1,RGS11,PLEKHG4B,NDUFAF2,TMEM108,GRID2IP,GNG7,LZTS1,ARHGAP29,MYO1C,MAL,STAT2,EHD3,PER1,TCF7L1,PLPPR4,DAG1,CREB3L1,MADD,SZT2,PRKAA2,CDK5R1,MNT,C3,ZMYND11,RAF1,RASA4B

regulation of biological quality	GO:0065008	2.99E-15	3741	477	84	ADORA2B,NCK2,APOB,FOXA1,CHRNA7,NLGN4X,ARF6,CXCL6,VWF,SH3GL2,TMEM38A,TADA3,ADGRB3,CHRNA2,SYP,RBM24,TEC,DGKB,SLC38A1,NRP1,TAOK1,DSG1,KCTD7,BNIP3L,XDH,SLC39A3,PHB2,CROCC,TRPC4,GRM2,CNR2,SH3GLB1,AQP4,ZC3H8,ARL6IP5,SLC25A23,KCNA1,CTNS,LPAR3,ALOX12,IP6K1,SLC30A2,COMMD9,COMMD1,HDAC6,CHAT,CELSR2,SCN5A,TNRC6A,SCP2,NPR3,WNK3,HEPH,PTPRO,MLST8,ANO6,THBD,ADORA2A,MED1,BAIAP3,NPHP4,COX19,ATP8B3,NDUFAF2,TMEM108,GRID2IP,LZTS1,USP9X,SCN8A,ABCA4,TENT5A,BLOC1S6,SLC25A44,EHD3,DAG1,TBXA2R,PRKAA2,CDK5R1,RAF1,FMNL3,CTRC,LIMA1,ZMPSTE24,HPCA
organelle organization	GO:0006996	3.60E-15	3805	489	86	POLR1B,NCK2,FANCM,FOXA1,ZPBP,ARF6,CCDC6,USP49,TMEM38A,INO80B,SMC6,DRC1,BFSP1,TTC19,SYP,NDUFS7,NRP1,TAOK1,CEP72,BNIP3L,TIMM10,PLK5,FSCN1,MAD2L1BP,PHB2,STRIP1,CROCC,UBQLN2,SH3GLB1,EML4,ANLN,RNF112,NEXN,RBBP4,HDAC6,MPHOSPH8,TANGO2,TJAP1,PCM1,CELSR2,SCP2,NSFL1C,DNM1L,BICD1,ULK4,MLST8,GOLGA8B,UBXN2B,TPM4,KRT3,BAIAP3,DHX30,NPHP4,NSD2,COX19,ATP8B3,NDUFAF2,CSDE1,RAD54L2,TMEM108,KDM1B,SHROOM2,FRMD5,MYO1C,BLOC1S6,STAT2,CHMP3,EHD3,TCF7L1,DAG1,STAG1,RNF8,PRKAA2,CDK5R1,NEK7,ZMYND11,RAF1,FMNL3,LIMA1,CILK1,ZMPSTE24,NPHP1,ZWILCH,KRT18,PINK1,H4C8
system development	GO:0048731	7.20E-15	4784	489	98	POLR1B,HAPLN1,NCK2,APOB,FOXA1,CHRNA7,PRKX,NLGN4X,ARF6,UBA6,TMEM204,ITGB8,SKI,SH3GL2,BMPER,DRC1,BFSP1,MACROD2,MEIS2,SOX5,ADGRB3,RBM24,NRP1,TAOK1,SDCBP2,XDH,PLK5,SLC39A3,ARMC6,PHB2,COPS2,EOMES,TRPC4,ZC3H8,INSM1,KCNA1,CTNS,LPAR3,ALOX12,ANLN,NKX2-8,PRPSAP2,CPNE6,RNF112,NEXN,GPRIN2,EPOR,SHOX,HDAC6,PTCHD1,PCM1,CELSR2,SCN5A,NELL1,NPR3,PTPRO,ULK4,RAB26,ANO6,ADORA2A,MED1,DHX30,NPHP4,NSD2,HECTD1,PLAC1,VIT,CSDE1,TMEM108,SHROOM2,LZTS1,C5,LGI2,USP9X,MAL,SCN8A,OGDH,BLOC1S6,LTK,PLPPR4,DAG1,CREB3L1,TBXA2R,SZT2,RARB,RNF8,CDK5R1,C3,POLE,RAF1,FMNL3,CALCRL,ATF1,ZMPSTE24,HPCA,RPS6KA3,NPHP1,H4C8
regulation of cell communication	GO:0010646	7.31E-15	3313	461	76	ADORA2B,NCK2,FOXA1,CHRNA7,NLGN4X,TRIM33,ARF6,KLHL31,TMEM204,ARHGAP9,SKI,VWF,BMPER,HOMER3,SYP,DGKB,NRP1,TAOK1,XDH,MAD2L1BP,PHB2,SLC15A3,UBQLN2,GRM2,CNR2,ARL6IP5,HIC1,SIRPA,CTNS,LPAR3,ARAF,C10ORF71,HDAC6,C12ORF66,DNM1L,BICD1,RELL1,PTPRO,ULK4,RAB26,MLST8,TXNDC12,STAMBP,IL36RN,FAF1,ADORA2A,MED1,BAIAP3,NPHP4,ZNF366,FRMD1,RGS11,PLEKHG4B,NDUFAF2,TMEM108,GRID2IP,GNG7,LZTS1,ARHGAP29,MYO1C,MAL,STAT2,PER1,TCF7L1,PLPPR4,DAG1,CREB3L1,MADD,SZT2,PRKAA2,CDK5R1,MNT,C3,ZMYND11,RAF1,RASA4B

multicellular organism development	GO:0007275	2.33E-14	5114	489	101	POLR1B,HAPLN1,NCK2,APOB,FOXA1,CHRNA7,PRKX,NLGN4X,ARF6,GINS1,UBA6,TMEM204,ITGB8,SKI,SH3GL2,BMPER,DRC1,BFSP1,MACROD2,MEIS2,SOX5,ADGRB3,RBM24,NRP1,TAOK1,SDCBP2,XDH,PLK5,SLC39A3,ARMC6,PHB2,COPS2,EOMES,TRPC4,ZC3H8,INSM1,KCNA1,CTNS,LPAR3,ALOX12,ANLN,NKX2-8,PRPSAP2,CPNE6,RNF112,NEXN,GPRIN2,EPOR,SHOX,HDAC6,PTCHD1,PCM1,CELSR2,SCN5A,NELL1,NPR3,PTPRO,ULK4,RAB26,ANO6,ADORA2A,MED1,DHX30,NPHP4,NSD2,HECTD1,ALG5,PLAC1,VIT,CSDE1,TMEM108,SHROOM2,LZTS1,C5,LGI2,USP9X,MAL,SCN8A,OGDH,BLOC1S6,LTK,PLPPR4,DAG1,CREB3L1,TBXA2R,SZT2,RARB,RNF8,CDK5R1,MNT,C3,POLE,RAF1,FMNL3,CALCRL,ATF1,ZMPSTE24,HPCA,RPS6KA3,NPHP1,H4C8
nervous system development	GO:0007399	3.84E-14	2428	478	64	HAPLN1,NCK2,APOB,FOXA1,CHRNA7,NLGN4X,ARF6,UBA6,SKI,SH3GL2,MACROD2,MEIS2,SOX5,ADGRB3,NRP1,TAOK1,SDCBP2,PLK5,COPS2,EOMES,TRPC4,INSM1,KCNA1,CTNS,LPAR3,NKX2-8,CPNE6,RNF112,NEXN,GPRIN2,EPOR,HDAC6,PTCHD1,PCM1,CELSR2,SCN5A,NELL1,PTPRO,ULK4,ADORA2A,MED1,DHX30,NPHP4,HECTD1,VIT,TMEM108,SHROOM2,LZTS1,LGI2,USP9X,MAL,SCN8A,OGDH,BLOC1S6,LTK,PLPPR4,DAG1,SZT2,RARB,CDK5R1,C3,ATF1,HPCA,RPS6KA3
macromolecule localization	GO:0033036	6.46E-14	3147	492	75	APOB,ARF6,PDIA2,HOMER3,TVP23B,SPIDR,DHRS1,RANBP2,NRP1,CEP72,BNIP3L,TIMM10,EEPD1,ZDHHC4,PRAM1,PHB2,SLC15A3,CROCC,OSBPL5,SH3GLB1,ARL6IP5,KCNA1,CTNS,UNC50,COMMD1,HDAC6,TANGO2,PCM1,CELSR2,SCP2,C12ORF66,NSFL1C,WNK3,DNM1L,BICD1,RAB26,MPP7,SVIP,UBXN2B,ANO6,FAF1,EXOC6B,ADORA2A,MED1,BAIAP3,NPHP4,NSD2,HECTD1,ATP8B3,NDUFAF2,SHROOM2,MYO1C,USP9X,MAL,ABCA4,BLOC1S6,CHMP3,EHD3,PLPPR4,DAG1,SMG7,GBP5,SZT2,PRKAA2,CDK5R1,C3,RAF1,CALCRL,LIMA1,HPCA,NPHP1,ZWILCH,KRT18,PINK1,SNX31
regulation of signal transduction	GO:0009966	3.01E-13	2929	461	68	ADORA2B,NCK2,FOXA1,CHRNA7,NLGN4X,TRIM33,ARF6,KLHL31,TMEM204,ARHGAP9,SKI,VWF,BMPER,HOMER3,SYP,NRP1,TAOK1,XDH,MAD2L1BP,PHB2,SLC15A3,UBQLN2,GRM2,ARL6IP5,HIC1,SIRPA,CTNS,LPAR3,ARAF,C10ORF71,HDAC6,C12ORF66,DNM1L,BICD1,RELL1,PTPRO,ULK4,MLST8,TXNDC12,STAMBP,IL36RN,FAF1,ADORA2A,MED1,NPHP4,ZNF366,FRMD1,RGS11,PLEKHG4B,TMEM108,GNG7,ARHGAP29,MYO1C,MAL,STAT2,PER1,TCF7L1,DAG1,CREB3L1,MADD,SZT2,PRKAA2,CDK5R1,MNT,C3,ZMYND11,RAF1,RASA4B

response to chemical	GO:0042221	5.38E-13	4316	487	88	NCK2,APOB,FOXA1,CHRNA7,TRIM33,ARF6,TMEM204,ITGB8,CXCL6,SKI,SH3GL2,TMEM38A,BMPER,TADA3,CDA,SPIDR,MEIS2,SOX5,CHRNA2,SYP,OR51E1,NRP1,DSG1,BNIP3L,XDH,PLK5,PHB2,UBQLN2,GRM2,CNR2,AQP4,ZC3H8,ARL6IP5,SLC25A23,SIRPA,KCNA1,REG1A,SLC30A2,CPNE6,RNF112,MARS1,NEXN,ST18,EPOR,HDAC6,SCN5A,OR7E24,WNK3,DNM1L,PTPRO,SVIP,IP6K2,ANO6,IL36RN,THBD,ADORA2A,MED1,BAIAP3,ZNF366,OR52I1,NDUFAF2,TMEM108,C5,MYO1C,USP9X,ABTB2,OR5BS1P,NCEH1,LTK,STAT2,PER1,DAG1,CREB3L1,GBP5,TBXA2R,EPM2AIP1,RARB,PRKAA2,CDK5R1,RAF1,RASA4B,CALCRL,ATF1,HPCA,RPS6KA3,TBL2,KRT18,PINK1
response to stress	GO:0006950	1.53E-12	3780	494	81	ADORA2B,NCK2,FANCM,CHRNA7,PDIA2,UBA6,KLHL31,CXCL6,VWF,INO80B,SMC6,SPAG11A,SPIDR,MACROD2,MYH13,RBM24,TEC,DGKB,TAOK1,BNIP3L,EEPD1,XDH,PLK5,POLN,PHB2,SLC15A3,UBQLN2,CNR2,SH3GLB1,AQP4,ARL6IP5,HIC1,SLC25A23,SIRPA,AOAH,C8B,ALOX12,RNF112,MARS1,PIRT,COMMD1,HDAC6,TNRC6A,C12ORF66,WNK3,RELL1,CFP,ULK4,TXNDC12,SVIP,ANO6,RNF216,IL36RN,THBD,ADORA2A,MED1,DEFB129,NSD2,C5,MYO1C,BLOC1S6,MARCHF2,STAT2,PER1,DAG1,CREB3L1,GBP5,TBXA2R,SZT2,RNF8,PRKAA2,C3,ZMYND11,POLE,RAF1,CALCRL,ZMPSTE24,RPS6KA3,TBL2,PINK1,FAM168A
protein localization	GO:0008104	1.94E-12	2700	492	66	APOB,ARF6,PDIA2,HOMER3,TVP23B,SPIDR,DHRS1,RANBP2,NRP1,CEP72,BNIP3L,TIMM10,ZDHHC4,PRAM1,PHB2,SLC15A3,CROCC,SH3GLB1,ARL6IP5,KCNA1,CTNS,UNC50,COMMD1,HDAC6,TANGO2,PCM1,CELSR2,SCP2,C12ORF66,NSFL1C,WNK3,DNM1L,BICD1,RAB26,MPP7,SVIP,UBXN2B,FAF1,EXOC6B,ADORA2A,MED1,BAIAP3,NPHP4,NSD2,HECTD1,NDUFAF2,SHROOM2,MYO1C,USP9X,MAL,BLOC1S6,CHMP3,EHD3,DAG1,GBP5,SZT2,PRKAA2,CDK5R1,RAF1,CALCRL,HPCA,NPHP1,ZWILCH,KRT18,PINK1,SNX31
macromolecule modification	GO:0043412	2.00E-12	3856	487	81	NCK2,FANCM,CHRNA7,TPGS2,PRKX,TRIM33,UBA6,KLHL31,USP49,SKI,SH3GL2,BMPER,TADA3,MACROD2,USP24,RANBP2,TEC,NRP1,TAOK1,GSPT1,CTBP1,ZDHHC4,XDH,PLK5,PHB2,COPS2,INSM1,SIRPA,TRMT2A,ARAF,RNF112,EEF2KMT,COMMD1,WDR6,HDAC6,MPHOSPH8,RCOR2,EXTL3,WNK3,PTPRO,ULK4,RNF144A,MLST8,STAMBP,SVIP,RNF216,VCPKMT,ADORA2A,CDC25A,NSD2,KLHDC1,HECTD1,ALG5,FKBP6,ALG8,KDM1B,DPY19L1,USP9X,PPIL4,NCEH1,FBXO33,OGDH,MARCHF2,LTK,STAT2,PER1,RNF24,DAG1,MADD,RNF8,PRKAA2,CDK5R1,NEK7,C3,CSKMT,RAF1,GALNT14,CILK1,ZMPSTE24,RPS6KA3,PINK1

cellular protein modification process	GO:0006464	2.65E-12	3642	487	78	NCK2,FANCM,CHRNA7,TPGS2,PRKX,TRIM33,UBA6,KLHL31,USP49,SKI,SH3GL2,BMPER,TADA3,MACROD2,USP24,RANBP2,TEC,NRP1,TAOK1,GSPT1,CTBP1,ZDHHC4,XDH,PLK5,PHB2,COPS2,INSM1,SIRPA,ARAF,RNF112,EEF2KMT,COMMD1,HDAC6,RCOR2,EXTL3,WNK3,PTPRO,ULK4,RNF144A,MLST8,STAMBP,SVIP,RNF216,VCPKMT,ADORA2A,CDC25A,NSD2,KLHDC1,HECTD1,ALG5,FKBP6,ALG8,KDM1B,DPY19L1,USP9X,PPIL4,NCEH1,FBXO33,OGDH,MARCHF2,LTK,STAT2,PER1,RNF24,DAG1,MADD,RNF8,PRKAA2,CDK5R1,NEK7,C3,CSKMT,RAF1,GALNT14,CILK1,ZMPSTE24,RPS6KA3,PINK1
protein modification process	GO:0036211	2.65E-12	3642	487	78	NCK2,FANCM,CHRNA7,TPGS2,PRKX,TRIM33,UBA6,KLHL31,USP49,SKI,SH3GL2,BMPER,TADA3,MACROD2,USP24,RANBP2,TEC,NRP1,TAOK1,GSPT1,CTBP1,ZDHHC4,XDH,PLK5,PHB2,COPS2,INSM1,SIRPA,ARAF,RNF112,EEF2KMT,COMMD1,HDAC6,RCOR2,EXTL3,WNK3,PTPRO,ULK4,RNF144A,MLST8,STAMBP,SVIP,RNF216,VCPKMT,ADORA2A,CDC25A,NSD2,KLHDC1,HECTD1,ALG5,FKBP6,ALG8,KDM1B,DPY19L1,USP9X,PPIL4,NCEH1,FBXO33,OGDH,MARCHF2,LTK,STAT2,PER1,RNF24,DAG1,MADD,RNF8,PRKAA2,CDK5R1,NEK7,C3,CSKMT,RAF1,GALNT14,CILK1,ZMPSTE24,RPS6KA3,PINK1
cellular localization	GO:0051641	4.90E-12	3117	492	71	ADORA2B,NLGN4X,ARF6,PDIA2,TMEM38A,HOMER3,SPIDR,DHRS1,RANBP2,SYP,NRP1,SDCBP2,CEP72,BNIP3L,TIMM10,ZDHHC4,NEMF,PRAM1,PHB2,CROCC,OSBPL5,SH3GLB1,ARL6IP5,EML4,SLC25A23,KCNA1,SLC30A2,UNC50,COMMD1,HDAC6,PCM1,SCN5A,SCP2,C12ORF66,NSFL1C,WNK3,DNM1L,BICD1,NOP9,RAB26,SVIP,UBXN2B,EXOC6B,ADORA2A,MED1,BAIAP3,NPHP4,HECTD1,NDUFAF2,TMEM108,SHROOM2,MYO1C,MAL,BLOC1S6,CHMP3,EHD3,DAG1,SMG7,GBP5,SZT2,CDK5R1,RAF1,CILK1,ZMPSTE24,TRAPPC1,HPCA,NPHP1,ZWILCH,KRT18,PINK1,SNX31
negative regulation of metabolic process	GO:0009892	7.44E-12	3558	487	76	NCK2,FOXA1,CHRNA7,TRIM33,KLHL31,ITGB8,SKI,SH3GL2,HOMER3,CDA,MEIS2,RBM24,GSPT1,CTBP1,XDH,PHB2,COPS2,EOMES,UBQLN2,AQP4,ZC3H8,INSM1,HIC1,EAPP,SIRPA,CTNS,GLIS1,IP6K1,WDR6,HDAC6,MIR99AHG,MPHOSPH8,RCOR2,RNPS1,TNRC6A,NELL1,NPR3,LINC00273,PTPRO,SVIP,RNF216,IL36RN,ADORA2A,MED1,NSD2,ZNF366,A2ML1,FKBP6,CSDE1,FHIT,KDM1B,LZTS1,C5,USP9X,TENT5A,MIR22HG,ZNF318,MIR124-1HG,PER1,SCML2,LSM5,SMG7,CREB3L1,RARB,RNF8,PRKAA2,CDK5R1,MNT,C3,ZMYND11,RAF1,ITIH5,ZMPSTE24,HPCA,RPS6KA3,PINK1
regulation of cellular component organization	GO:0051128	7.90E-12	2246	487	58	NCK2,CHRNA7,ARF6,CDA,SPIDR,ADGRB3,DGKB,NRP1,TAOK1,GSPT1,BNIP3L,PLK5,FSCN1,MAD2L1BP,CROCC,UBQLN2,SH3GLB1,INSM1,LPAR3,ANLN,CPNE6,NEXN,HDAC6,MPHOSPH8,EXTL3,DNM1L,BICD1,PTPRO,ULK4,MLST8,MPP7,SVIP,IP6K2,ANO6,FAF1,NPHP4,LZTS1,MYO1C,ST7L,LTK,STAT2,CHMP3,EHD3,DAG1,GBP5,PRKAA2,CDK5R1,NEK7,C3,RAF1,LIMA1,ATF1,ZMPSTE24,HPCA,RPS6KA3,NPHP1,ZWILCH,PINK1

cellular response to chemical stimulus	GO:0070887	1.77E-11	3008	487	68	NCK2,APOB,FOXA1,CHRNA7,TRIM33,ARF6,TMEM204,ITGB8,CXCL6,SKI,SH3GL2,TMEM38A,BMPER,TADA3,SPIDR,SOX5,SYP,NRP1,BNIP3L,XDH,PLK5,PHB2,CNR2,AQP4,ARL6IP5,SLC25A23,SIRPA,KCNA1,CPNE6,RNF112,MARS1,ST18,EPOR,HDAC6,SCN5A,WNK3,DNM1L,PTPRO,IP6K2,ANO6,IL36RN,MED1,BAIAP3,ZNF366,NDUFAF2,TMEM108,C5,MYO1C,USP9X,ABTB2,NCEH1,LTK,STAT2,PER1,DAG1,CREB3L1,GBP5,TBXA2R,RARB,PRKAA2,CDK5R1,RAF1,RASA4B,CALCRL,HPCA,TBL2,KRT18,PINK1
phosphate-containing compound metabolic process	GO:0006796	2.82E-11	2742	487	64	ADORA2B,NCK2,CHRNA7,PRKX,KLHL31,SH3GL2,SLC44A5,BMPER,TADA3,CDA,RANBP2,ADCY4,TEC,DGKB,NRP1,TAOK1,CTBP1,XDH,PLK5,PHB2,COPS2,MOCS1,OSBPL5,SLC17A2,INSM1,SIRPA,IP6K1,ARAF,PRPSAP2,HDAC6,CHAT,SCP2,WNK3,GALK2,PTPRO,ULK4,MLST8,BTBD10,IP6K2,ADORA2A,CDC25A,FHIT,CMPK1,PPIL4,NCEH1,OGDH,LTK,STAT2,PLPPR4,DAG1,SMG7,MADD,SUCLA2,PLPP2,PRKAA2,CDK5R1,NEK7,C3,RAF1,CILK1,ZMPSTE24,HPCA,RPS6KA3,PINK1
positive regulation of metabolic process	GO:0009893	2.97E-11	3678	494	77	ADORA2B,NCK2,APOB,FANCM,FOXA1,CHRNA7,ITGB8,SKI,TADA3,SPIDR,MEIS2,RANBP2,RBM24,TEC,NRP1,TAOK1,BNIP3L,CTBP1,XDH,PHB2,EOMES,UBQLN2,SH3GLB1,ZC3H8,ARL6IP5,EAPP,GLIS1,ARAF,NKX2-8,MARS1,ST18,COMMD1,SHOX,HDAC6,TNRC6A,SCP2,WNK3,ZNF721,RNF144A,MLST8,SVIP,BTBD10,FAF1,MED1,NSD2,HECTD1,ZNF43,KDM1B,C5,USP9X,TENT5A,FBXO33,MARCHF2,LTK,ZNF318,PSME2,PER1,AGXT2,DAG1,CREB3L1,GBP5,MADD,EPM2AIP1,RARB,RNF8,PRKAA2,CDK5R1,NEK7,C3,RAF1,ATF1,ZMPSTE24,RPS6KA3,CREB5,PINK1,ZNF143,FAM168A
phosphorus metabolic process	GO:0006793	3.98E-11	2763	487	64	ADORA2B,NCK2,CHRNA7,PRKX,KLHL31,SH3GL2,SLC44A5,BMPER,TADA3,CDA,RANBP2,ADCY4,TEC,DGKB,NRP1,TAOK1,CTBP1,XDH,PLK5,PHB2,COPS2,MOCS1,OSBPL5,SLC17A2,INSM1,SIRPA,IP6K1,ARAF,PRPSAP2,HDAC6,CHAT,SCP2,WNK3,GALK2,PTPRO,ULK4,MLST8,BTBD10,IP6K2,ADORA2A,CDC25A,FHIT,CMPK1,PPIL4,NCEH1,OGDH,LTK,STAT2,PLPPR4,DAG1,SMG7,MADD,SUCLA2,PLPP2,PRKAA2,CDK5R1,NEK7,C3,RAF1,CILK1,ZMPSTE24,HPCA,RPS6KA3,PINK1
positive regulation of cellular metabolic process	GO:0031325	4.80E-11	3176	494	70	ADORA2B,NCK2,FANCM,FOXA1,CHRNA7,SKI,TADA3,SPIDR,MEIS2,RANBP2,RBM24,TEC,NRP1,TAOK1,BNIP3L,CTBP1,XDH,PHB2,EOMES,UBQLN2,SH3GLB1,ZC3H8,ARL6IP5,EAPP,GLIS1,ARAF,NKX2-8,MARS1,ST18,COMMD1,SHOX,HDAC6,TNRC6A,WNK3,ZNF721,RNF144A,MLST8,SVIP,BTBD10,FAF1,MED1,NSD2,HECTD1,ZNF43,KDM1B,USP9X,FBXO33,MARCHF2,LTK,ZNF318,PSME2,PER1,AGXT2,DAG1,CREB3L1,MADD,EPM2AIP1,RARB,RNF8,PRKAA2,CDK5R1,NEK7,C3,RAF1,ATF1,RPS6KA3,CREB5,PINK1,ZNF143,FAM168A

negative regulation of cellular metabolic process	GO:0031324	5.51E-11	2493	487	60	NCK2,FOXA1,CHRNA7,TRIM33,KLHL31,SKI,SH3GL2,CDA,MEIS2,RBM24,CTBP1,XDH,PHB2,COPS2,EOMES,UBQLN2,ZC3H8,INSM1,HIC1,EAPP,SIRPA,CTNS,GLIS1,WDR6,HDAC6,MPHOSPH8,RCOR2,RNPS1,TNRC6A,NELL1,PTPRO,SVIP,ADORA2A,MED1,NSD2,ZNF366,A2ML1,FHIT,KDM1B,LZTS1,C5,USP9X,TENT5A,ZNF318,PER1,SCML2,CREB3L1,RARB,RNF8,PRKAA2,CDK5R1,MNT,C3,ZMYND11,RAF1,ITIH5,ZMPSTE24,HPCA,RPS6KA3,PINK1
macromolecule biosynthetic process	GO:0009059	6.63E-11	4880	493	91	POLR1B,NCK2,APOB,FANCM,FOXA1,TRIM33,GINS1,ZNF286A,SKI,ZNF561,TADA3,ZNF506,ZNF121,MEIS2,SOX5,RBM24,FAM111B,GSPT1,CTBP1,ZDHHC4,CARS1,POLN,NEMF,PHB2,COPS2,EOMES,ZNF90,ZC3H8,INSM1,HIC1,EAPP,GLIS1,NKX2-8,MARS1,ST18,COMMD1,RBBP4,SHOX,TEFM,HDAC6,PERM1,MPHOSPH8,RCOR2,RNPS1,CELSR2,TNRC6A,EXTL3,ZNF721,EBF4,SVIP,FAF1,ADORA2A,MED1,NSD2,ZNF366,ALG5,CSDE1,ZNF43,ALG8,KDM1B,DPY19L1,USP9X,PPIL4,ZSCAN29,ZNF318,STAT2,PER1,CHST12,RPL15,TCF7L1,SCML2,CREB3L1,EPM2AIP1,ZNF676,RARB,RNF8,CDK5R1,NEK7,MNT,ZMYND11,POLE,RAF1,GALNT14,ATF1,ZMPSTE24,RPS6KA3,CREB5,PINK1,ZNF143,H4C8,ZNF320
cellular nitrogen compound biosynthetic process	GO:0044271	8.49E-11	4734	493	89	POLR1B,ADORA2B,NCK2,FOXA1,TRIM33,SMS,ZNF286A,SKI,ZNF561,TADA3,ZNF506,CDA,ZNF121,MEIS2,ADCY4,SOX5,RBM24,GSPT1,CTBP1,CARS1,POLN,NEMF,PHB2,COPS2,EOMES,ZNF90,ZC3H8,INSM1,HIC1,EAPP,SIRPA,GLIS1,NKX2-8,PRPSAP2,MARS1,CHAC2,ST18,COMMD1,SHOX,TEFM,HDAC6,PERM1,MPHOSPH8,RCOR2,RNPS1,CELSR2,TNRC6A,ZNF721,EBF4,FAF1,ADORA2A,MED1,NSD2,ZNF366,CSDE1,ZNF43,CMPK1,KDM1B,USP9X,PPIL4,ZSCAN29,ZNF318,STAT2,PER1,RPL15,AGXT2,TCF7L1,SCML2,CARNS1,CREB3L1,ZNF676,RARB,SUCLA2,RNF8,CDK5R1,NEK7,MNT,ZMYND11,POLE,RAF1,ATF1,ZMPSTE24,HPCA,RPS6KA3,CREB5,PINK1,ZNF143,H4C8,ZNF320
cell cycle	GO:0007049	1.07E-10	1713	486	48	FANCM,FOXA1,ARF6,GINS1,TADA3,TTC19,MEIS2,TAOK1,GSPT1,CEP72,CTBP1,PLK5,MAD2L1BP,PHB2,CROCC,SH3GLB1,INSM1,EML4,ANLN,RNF112,WDR6,RBBP4,MS4A3,PCM1,NSFL1C,STAMBP,GOLGA8B,UBXN2B,MED1,CDC25A,FKBP6,LZTS1,USP9X,ZNF318,CHMP3,PSME2,MADD,STAG1,RNF8,CDK5R1,NEK7,MNT,ZMYND11,POLE,ZMPSTE24,RPS6KA3,ZWILCH,KRT18

organic cyclic compound biosynthetic process	GO:1901362	1.10E-10	4181	493	82	POLR1B,ADORA2B,NCK2,APOB,FOXA1,TRIM33,ZNF286A,SKI,ZNF561,TADA3,ZNF506,CDA,ZNF121,MEIS2,ADCY4,SOX5,CTBP1,POLN,PHB2,COPS2,MOCS1,EOMES,ZNF90,ZC3H8,INSM1,HIC1,EAPP,CTNS,GLIS1,NKX2-8,PRPSAP2,MARS1,ST18,COMMD1,SHOX,TEFM,HDAC6,PERM1,MPHOSPH8,RCOR2,RNPS1,CELSR2,SCP2,ZNF721,EBF4,FAF1,ADORA2A,MED1,NSD2,ZNF366,ZNF43,CMPK1,KDM1B,USP9X,PPIL4,ZSCAN29,ZNF318,STAT2,PER1,TCF7L1,SCML2,CREB3L1,ZNF676,RARB,SUCLA2,RNF8,PRKAA2,CDK5R1,NEK7,MNT,ZMYND11,POLE,RAF1,ATF1,ZMPSTE24,HPCA,RPS6KA3,CREB5,PINK1,ZNF143,H4C8,ZNF320
response to external stimulus	GO:0009605	1.69E-10	2752	479	62	ADORA2B,APOB,BEST1,ITGB8,CXCL6,SPAG11A,CDA,MACROD2,MEIS2,MYH13,DGKB,NRP1,BNIP3L,PHB2,SLC15A3,CNR2,SH3GLB1,AQP4,SIRPA,KCNA1,AOAH,REG1A,C8B,ALOX12,MARS1,NEXN,TNRC6A,C12ORF66,DNM1L,PTPRO,CFP,ANO6,RNF216,IL36RN,THBD,ADORA2A,MED1,DEFB129,REG4,SHROOM2,C5,MYO1C,ABCA4,TENT5A,BLOC1S6,MARCHF2,STAT2,PER1,LSM5,DAG1,GBP5,TBXA2R,SZT2,PRKAA2,CDK5R1,C3,ZMYND11,RAF1,CALCRL,ZMPSTE24,RPS6KA3,TBL2
intracellular signal transduction	GO:0035556	1.90E-10	2639	487	61	ADORA2B,NCK2,CHRNA7,ARF6,KLHL31,ARHGAP9,VWF,TMEM38A,BMPER,HOMER3,ADCY4,TEC,DGKB,NRP1,TAOK1,SDCBP2,XDH,PLK5,MAD2L1BP,PHB2,SLC15A3,GRM2,ARL6IP5,HIC1,SIRPA,CTNS,LPAR3,ARAF,PIRT,C10ORF71,NPR3,C12ORF66,WNK3,DNM1L,RELL1,ULK4,MLST8,TXNDC12,STAMBP,FRMD1,RGS11,PLEKHG4B,FHIT,ARHGAP29,LTK,PER1,DAG1,CREB3L1,MADD,TBXA2R,SZT2,PRKAA2,ZMYND11,RAF1,RASA4B,CILK1,ZMPSTE24,HPCA,RPS6KA3,ZWILCH,PINK1
cellular response to stress	GO:0033554	2.36E-10	1852	494	50	ADORA2B,NCK2,FANCM,PDIA2,UBA6,KLHL31,INO80B,SMC6,SPIDR,MACROD2,MYH13,RBM24,TAOK1,BNIP3L,EEPD1,XDH,PLK5,POLN,PHB2,UBQLN2,SH3GLB1,ARL6IP5,HIC1,SLC25A23,SIRPA,RNF112,MARS1,COMMD1,HDAC6,TNRC6A,C12ORF66,WNK3,RELL1,ULK4,TXNDC12,SVIP,NSD2,PER1,DAG1,CREB3L1,SZT2,RNF8,PRKAA2,ZMYND11,POLE,ZMPSTE24,RPS6KA3,TBL2,PINK1,FAM168A

regulation of gene expression	GO:0010468	4.41E-10	5388	493	95	ADORA2B,NCK2,APOB,FOXA1,CHRNA7,TRIM33,ZNF286A,ITGB8,CXCL6,SKI,ZNF561,SH3GL2,TADA3,HOMER3,ZNF506,ZNF121,MEIS2,SOX5,RBM24,LDLRAD3,GSPT1,CTBP1,XDH,PHB2,COPS2,EOMES,AQP4,ZNF90,ZC3H8,INSM1,HIC1,EAPP,SIRPA,GLIS1,NKX2-8,MARS1,ST18,COMMD1,SHOX,HDAC6,MIR99AHG,PERM1,MPHOSPH8,RCOR2,RNPS1,CELSR2,TNRC6A,NELL1,ZNF721,LINC00273,DNM1L,EBF4,RNF216,IL36RN,FAF1,ADORA2A,MED1,NSD2,ZNF366,FKBP6,CSDE1,ZNF43,KDM1B,C5,USP9X,PPIL4,TENT5A,ZSCAN29,MIR22HG,ZNF318,STAT2,MIR124-1HG,PER1,TCF7L1,SCML2,LSM5,SMG7,CREB3L1,GBP5,ZNF676,RARB,RNF8,PRKAA2,CDK5R1,MNT,C3,ZMYND11,RAF1,ATF1,ZMPSTE24,RPS6KA3,CREB5,PINK1,ZNF143,ZNF320
neurogenesis	GO:0022008	4.43E-10	1639	452	44	NCK2,FOXA1,CHRNA7,NLGN4X,ARF6,UBA6,SKI,SH3GL2,SOX5,ADGRB3,NRP1,TAOK1,PLK5,COPS2,EOMES,TRPC4,INSM1,KCNA1,LPAR3,NKX2-8,CPNE6,RNF112,NEXN,GPRIN2,HDAC6,PCM1,CELSR2,PTPRO,ULK4,ADORA2A,MED1,NPHP4,TMEM108,LZTS1,USP9X,OGDH,BLOC1S6,LTK,PLPPR4,DAG1,SZT2,RARB,CDK5R1,C3
response to organic substance	GO:0010033	5.18E-10	3009	486	65	NCK2,APOB,FOXA1,CHRNA7,TRIM33,ARF6,TMEM204,ITGB8,CXCL6,SKI,SH3GL2,TMEM38A,BMPER,TADA3,CDA,SPIDR,MEIS2,SOX5,SYP,NRP1,DSG1,XDH,PLK5,PHB2,UBQLN2,GRM2,CNR2,AQP4,ARL6IP5,SIRPA,REG1A,MARS1,ST18,EPOR,HDAC6,SVIP,IP6K2,IL36RN,THBD,ADORA2A,MED1,BAIAP3,ZNF366,NDUFAF2,TMEM108,MYO1C,USP9X,LTK,STAT2,PER1,DAG1,CREB3L1,GBP5,TBXA2R,EPM2AIP1,RARB,PRKAA2,CDK5R1,RAF1,CALCRL,ATF1,HPCA,RPS6KA3,TBL2,KRT18
generation of neurons	GO:0048699	6.16E-10	1519	452	42	NCK2,FOXA1,CHRNA7,NLGN4X,ARF6,UBA6,SKI,SH3GL2,SOX5,ADGRB3,NRP1,TAOK1,PLK5,COPS2,EOMES,INSM1,KCNA1,LPAR3,NKX2-8,CPNE6,RNF112,NEXN,GPRIN2,HDAC6,PCM1,CELSR2,PTPRO,ULK4,ADORA2A,MED1,NPHP4,TMEM108,LZTS1,USP9X,OGDH,BLOC1S6,LTK,PLPPR4,DAG1,SZT2,CDK5R1,C3
negative regulation of nitrogen compound metabolic process	GO:0051172	7.21E-10	2330	478	55	NCK2,FOXA1,CHRNA7,TRIM33,KLHL31,SKI,SH3GL2,CDA,MEIS2,RBM24,CTBP1,XDH,PHB2,COPS2,EOMES,ZC3H8,INSM1,HIC1,EAPP,SIRPA,GLIS1,HDAC6,MPHOSPH8,RCOR2,RNPS1,TNRC6A,NELL1,PTPRO,SVIP,ADORA2A,MED1,NSD2,ZNF366,A2ML1,FHIT,KDM1B,C5,USP9X,TENT5A,ZNF318,PER1,SCML2,CREB3L1,RARB,RNF8,PRKAA2,CDK5R1,MNT,C3,ZMYND11,RAF1,ITIH5,ZMPSTE24,HPCA,RPS6KA3

positive regulation of macromolecule metabolic process	GO:0010604	8.88E-10	3378	494	70	ADORA2B,NCK2,APOB,FANCM,FOXA1,CHRNA7,ITGB8,SKI,TADA3,SPIDR,MEIS2,RBM24,TEC,NRP1,TAOK1,CTBP1,XDH,PHB2,EOMES,UBQLN2,ZC3H8,ARL6IP5,EAPP,GLIS1,ARAF,NKX2-8,MARS1,ST18,COMMD1,SHOX,HDAC6,TNRC6A,WNK3,ZNF721,RNF144A,MLST8,SVIP,FAF1,MED1,NSD2,HECTD1,ZNF43,KDM1B,C5,USP9X,TENT5A,FBXO33,MARCHF2,ZNF318,PSME2,PER1,DAG1,CREB3L1,GBP5,MADD,EPM2AIP1,RARB,RNF8,PRKAA2,CDK5R1,NEK7,C3,RAF1,ATF1,ZMPSTE24,RPS6KA3,CREB5,PINK1,ZNF143,FAM168A
heterocycle biosynthetic process	GO:0018130	9.10E-10	4024	493	78	POLR1B,ADORA2B,NCK2,FOXA1,TRIM33,ZNF286A,SKI,ZNF561,TADA3,ZNF506,CDA,ZNF121,MEIS2,ADCY4,SOX5,CTBP1,POLN,PHB2,COPS2,MOCS1,EOMES,ZNF90,ZC3H8,INSM1,HIC1,EAPP,GLIS1,NKX2-8,PRPSAP2,MARS1,ST18,COMMD1,SHOX,TEFM,HDAC6,PERM1,MPHOSPH8,RCOR2,RNPS1,CELSR2,ZNF721,EBF4,FAF1,ADORA2A,MED1,NSD2,ZNF366,ZNF43,CMPK1,KDM1B,USP9X,PPIL4,ZSCAN29,ZNF318,STAT2,PER1,TCF7L1,SCML2,CREB3L1,ZNF676,RARB,SUCLA2,RNF8,CDK5R1,NEK7,MNT,ZMYND11,POLE,RAF1,ATF1,ZMPSTE24,HPCA,RPS6KA3,CREB5,PINK1,ZNF143,H4C8,ZNF320
negative regulation of macromolecule metabolic process	GO:0010605	9.28E-10	3353	487	69	NCK2,FOXA1,CHRNA7,TRIM33,KLHL31,ITGB8,SKI,SH3GL2,HOMER3,MEIS2,RBM24,GSPT1,CTBP1,XDH,PHB2,COPS2,EOMES,UBQLN2,AQP4,ZC3H8,INSM1,HIC1,EAPP,SIRPA,GLIS1,HDAC6,MIR99AHG,MPHOSPH8,RCOR2,RNPS1,TNRC6A,NELL1,LINC00273,PTPRO,SVIP,RNF216,IL36RN,ADORA2A,MED1,NSD2,ZNF366,A2ML1,FKBP6,CSDE1,FHIT,KDM1B,C5,USP9X,TENT5A,MIR22HG,ZNF318,MIR124-1HG,PER1,SCML2,LSM5,SMG7,CREB3L1,RARB,RNF8,PRKAA2,CDK5R1,MNT,C3,ZMYND11,RAF1,ITIH5,ZMPSTE24,RPS6KA3,PINK1
aromatic compound biosynthetic process	GO:0019438	1.04E-09	4035	493	78	POLR1B,ADORA2B,NCK2,FOXA1,TRIM33,ZNF286A,SKI,ZNF561,TADA3,ZNF506,CDA,ZNF121,MEIS2,ADCY4,SOX5,CTBP1,POLN,PHB2,COPS2,EOMES,ZNF90,ZC3H8,INSM1,HIC1,EAPP,CTNS,GLIS1,NKX2-8,PRPSAP2,MARS1,ST18,COMMD1,SHOX,TEFM,HDAC6,PERM1,MPHOSPH8,RCOR2,RNPS1,CELSR2,ZNF721,EBF4,FAF1,ADORA2A,MED1,NSD2,ZNF366,ZNF43,CMPK1,KDM1B,USP9X,PPIL4,ZSCAN29,ZNF318,STAT2,PER1,TCF7L1,SCML2,CREB3L1,ZNF676,RARB,SUCLA2,RNF8,CDK5R1,NEK7,MNT,ZMYND11,POLE,RAF1,ATF1,ZMPSTE24,HPCA,RPS6KA3,CREB5,PINK1,ZNF143,H4C8,ZNF320
regulation of cellular protein metabolic process	GO:0032268	1.08E-09	2309	487	55	NCK2,FANCM,CHRNA7,KLHL31,SKI,SH3GL2,BMPER,TADA3,RBM24,TEC,NRP1,TAOK1,GSPT1,CTBP1,XDH,PHB2,UBQLN2,ARL6IP5,INSM1,SIRPA,ARAF,ST18,COMMD1,HDAC6,TNRC6A,NELL1,WNK3,PTPRO,RNF144A,MLST8,SVIP,FAF1,ADORA2A,CDC25A,A2ML1,HECTD1,CSDE1,FHIT,C5,PPIL4,FBXO33,MARCHF2,STAT2,PSME2,PER1,DAG1,MADD,PRKAA2,CDK5R1,C3,RAF1,ITIH5,ZMPSTE24,RPS6KA3,PINK1

regulation of biosynthetic process	GO:0009889	1.41E-09	4141	493	79	ADORA2B,NCK2,APOB,FOXA1,TRIM33,ZNF286A,SKI,ZNF561,TADA3,ZNF506,ZNF121,MEIS2,RANBP2,SOX5,RBM24,GSPT1,CTBP1,PHB2,COPS2,EOMES,ZNF90,ZC3H8,INSM1,HIC1,EAPP,SIRPA,CTNS,GLIS1,NKX2-8,MARS1,ST18,COMMD1,SHOX,HDAC6,PERM1,MPHOSPH8,RCOR2,CELSR2,TNRC6A,SCP2,ZNF721,EBF4,SVIP,FAF1,ADORA2A,MED1,NSD2,ZNF366,CSDE1,ZNF43,KDM1B,USP9X,PPIL4,ZSCAN29,ZNF318,STAT2,PER1,AGXT2,TCF7L1,SCML2,CREB3L1,EPM2AIP1,ZNF676,RARB,RNF8,CDK5R1,NEK7,MNT,C3,ZMYND11,RAF1,ATF1,ZMPSTE24,HPCA,RPS6KA3,CREB5,PINK1,ZNF143,ZNF320
regulation of protein metabolic process	GO:0051246	1.46E-09	2472	487	57	NCK2,FANCM,CHRNA7,KLHL31,SKI,SH3GL2,BMPER,TADA3,RBM24,TEC,LDLRAD3,NRP1,TAOK1,GSPT1,CTBP1,XDH,PHB2,UBQLN2,ARL6IP5,INSM1,SIRPA,ARAF,ST18,COMMD1,HDAC6,TNRC6A,NELL1,WNK3,PTPRO,RNF144A,RAB26,MLST8,SVIP,FAF1,ADORA2A,CDC25A,A2ML1,HECTD1,CSDE1,FHIT,C5,PPIL4,FBXO33,MARCHF2,STAT2,PSME2,PER1,DAG1,MADD,PRKAA2,CDK5R1,C3,RAF1,ITIH5,ZMPSTE24,RPS6KA3,PINK1
positive regulation of nitrogen compound metabolic process	GO:0051173	2.11E-09	2978	494	64	ADORA2B,NCK2,FANCM,FOXA1,CHRNA7,SKI,TADA3,SPIDR,MEIS2,RBM24,TEC,NRP1,TAOK1,CTBP1,XDH,PHB2,EOMES,UBQLN2,ZC3H8,ARL6IP5,EAPP,GLIS1,ARAF,NKX2-8,MARS1,ST18,COMMD1,SHOX,HDAC6,TNRC6A,WNK3,ZNF721,RNF144A,MLST8,SVIP,FAF1,MED1,NSD2,HECTD1,ZNF43,KDM1B,USP9X,FBXO33,MARCHF2,ZNF318,PSME2,PER1,AGXT2,DAG1,CREB3L1,MADD,RARB,RNF8,PRKAA2,CDK5R1,NEK7,C3,RAF1,ATF1,RPS6KA3,CREB5,PINK1,ZNF143,FAM168A
nucleobase-containing compound biosynthetic process	GO:0034654	2.83E-09	3953	493	76	POLR1B,ADORA2B,NCK2,FOXA1,TRIM33,ZNF286A,SKI,ZNF561,TADA3,ZNF506,CDA,ZNF121,MEIS2,ADCY4,SOX5,CTBP1,POLN,PHB2,COPS2,EOMES,ZNF90,ZC3H8,INSM1,HIC1,EAPP,GLIS1,NKX2-8,PRPSAP2,MARS1,ST18,COMMD1,SHOX,TEFM,HDAC6,PERM1,MPHOSPH8,RCOR2,RNPS1,CELSR2,ZNF721,EBF4,FAF1,ADORA2A,MED1,NSD2,ZNF366,ZNF43,CMPK1,KDM1B,USP9X,PPIL4,ZSCAN29,ZNF318,STAT2,PER1,TCF7L1,SCML2,CREB3L1,ZNF676,RARB,RNF8,CDK5R1,NEK7,MNT,ZMYND11,POLE,RAF1,ATF1,ZMPSTE24,HPCA,RPS6KA3,CREB5,PINK1,ZNF143,H4C8,ZNF320
cation transport	GO:0006812	3.44E-09	1174	487	37	BEST1,CHRNA7,TMEM38A,SLC44A5,HOMER3,NDUFS7,SLC38A1,SLC39A3,PHB2,SLC15A3,SLC17A2,TRPC4,GRM2,SLC25A23,KCNA1,CTNS,SLC30A2,PIRT,COMMD9,COMMD1,SCN5A,KCNS3,WNK3,KCNA4,HEPH,DNM1L,ANO6,ADORA2A,SLC5A10,SHROOM2,SCN8A,EHD3,PER1,CALCRL,ZMPSTE24,HPCA,PINK1

regulation of cellular biosynthetic process	GO:0031326	4.95E-09	4081	493	77	ADORA2B,NCK2,FOXA1,TRIM33,ZNF286A,SKI,ZNF561,TADA3,ZNF506,ZNF121,MEIS2,SOX5,RBM24,GSPT1,CTBP1,PHB2,COPS2,EOMES,ZNF90,ZC3H8,INSM1,HIC1,EAPP,SIRPA,CTNS,GLIS1,NKX2-8,MARS1,ST18,COMMD1,SHOX,HDAC6,PERM1,MPHOSPH8,RCOR2,CELSR2,TNRC6A,SCP2,ZNF721,EBF4,SVIP,FAF1,ADORA2A,MED1,NSD2,ZNF366,CSDE1,ZNF43,KDM1B,USP9X,PPIL4,ZSCAN29,ZNF318,STAT2,PER1,AGXT2,TCF7L1,SCML2,CREB3L1,EPM2AIP1,ZNF676,RARB,RNF8,CDK5R1,NEK7,MNT,C3,ZMYND11,RAF1,ATF1,ZMPSTE24,HPCA,RPS6KA3,CREB5,PINK1,ZNF143,ZNF320
phosphorylation	GO:0016310	5.60E-09	1841	487	47	NCK2,CHRNA7,PRKX,KLHL31,SH3GL2,BMPER,TADA3,RANBP2,TEC,DGKB,NRP1,TAOK1,CTBP1,XDH,PLK5,PHB2,COPS2,INSM1,SIRPA,IP6K1,ARAF,HDAC6,WNK3,GALK2,PTPRO,ULK4,MLST8,BTBD10,IP6K2,ADORA2A,CDC25A,CMPK1,PPIL4,OGDH,LTK,STAT2,DAG1,MADD,PRKAA2,CDK5R1,NEK7,C3,RAF1,CILK1,ZMPSTE24,RPS6KA3,PINK1
cellular developmental process	GO:0048869	5.61E-09	4210	489	78	POLR1B,NCK2,APOB,FOXA1,ZPBP,CHRNA7,PRKX,NLGN4X,ARF6,UBA6,TMEM204,ITGB8,SKI,SH3GL2,SMC6,BFSP1,MEIS2,SOX5,ADGRB3,RBM24,NRP1,TAOK1,BCL7B,CTBP1,XDH,PLK5,ARMC6,COPS2,EOMES,TRPC4,ZC3H8,INSM1,KCNA1,GLIS1,LPAR3,ANLN,NKX2-8,CPNE6,RNF112,NEXN,GPRIN2,HDAC6,PCM1,CELSR2,NELL1,DNM1L,PTPRO,ULK4,TPM4,ADORA2A,MED1,KRT3,NPHP4,HECTD1,FKBP6,TMEM108,LZTS1,USP9X,MAL,OGDH,BLOC1S6,LTK,PLPPR4,DAG1,CREB3L1,SZT2,RARB,RNF8,CDK5R1,MNT,C3,RAF1,ATF1,ZMPSTE24,RPS6KA3,NPHP1,SPATA16,H4C8
organic substance transport	GO:0071702	6.51E-09	2689	492	59	APOB,BEST1,ARF6,SLC44A5,HOMER3,TVP23B,DHRS1,RANBP2,SLC38A1,BNIP3L,TIMM10,EEPD1,ZDHHC4,PHB2,SLC15A3,OSBPL5,SLC17A2,TRPC4,GRM2,SH3GLB1,AQP4,ARL6IP5,SLC25A23,CTNS,UNC50,COMMD1,HDAC6,TANGO2,PCM1,SCP2,DNM1L,RAB26,SVIP,ANO6,EXOC6B,ADORA2A,MED1,BAIAP3,ATP8B3,SLC5A10,CD320,NDUFAF2,MYO1C,ABCA4,BLOC1S6,CHMP3,SLC25A44,EHD3,PLPPR4,SMG7,CDK5R1,C3,RAF1,CALCRL,LIMA1,HPCA,KRT18,PINK1,SNX31
neuron differentiation	GO:0030182	6.60E-09	1364	473	39	NCK2,FOXA1,CHRNA7,NLGN4X,ARF6,UBA6,SH3GL2,ADGRB3,NRP1,TAOK1,PLK5,COPS2,EOMES,INSM1,LPAR3,NKX2-8,CPNE6,RNF112,NEXN,GPRIN2,HDAC6,CELSR2,PTPRO,ULK4,ADORA2A,MED1,NPHP4,TMEM108,LZTS1,USP9X,OGDH,BLOC1S6,LTK,PLPPR4,DAG1,SZT2,CDK5R1,C3,ATF1

regulation of transport	GO:0051049	7.29E-09	1719	487	45	ADORA2B,BEST1,ARF6,TMEM38A,HOMER3,SYP,NRP1,KCTD7,BNIP3L,EEPD1,PRAM1,PHB2,UBQLN2,GRM2,SH3GLB1,SIRPB1,ARL6IP5,SIRPA,KCNA1,PIRT,COMMD1,PCM1,SCN5A,SCP2,KCNS3,WNK3,KCNA4,DNM1L,BICD1,RAB26,SVIP,ANO6,ADORA2A,BAIAP3,NDUFAF2,MYO1C,SCN8A,CHMP3,EHD3,PER1,CDK5R1,C3,ZMPSTE24,HPCA,PINK1
cellular catabolic process	GO:0044248	7.90E-09	2188	446	49	APOB,UBA6,USP49,CDA,USP24,RBM24,GSPT1,BNIP3L,XDH,NEMF,PHB2,UBQLN2,SH3GLB1,AOAH,ARAF,CHAC2,COMMD1,WDR6,HDAC6,RNPS1,TNRC6A,NELL1,SCP2,NSFL1C,DNM1L,RNF144A,SVIP,UBXN2B,RNF216,FAF1,KLHDC1,HECTD1,CSDE1,FHIT,LZTS1,USP9X,TENT5A,FBXO33,MARCHF2,CHMP3,SLC25A44,PSME2,AGXT2,LSM5,SMG7,CARNS1,RNF8,PRKAA2,CDK5R1
negative regulation of response to stimulus	GO:0048585	8.92E-09	1604	465	42	NCK2,NLGN4X,TRIM33,KLHL31,SKI,BMPER,HOMER3,NRP1,XDH,MAD2L1BP,PHB2,UBQLN2,CNR2,HIC1,SIRPA,AOAH,ALOX12,C12ORF66,BICD1,PTPRO,TXNDC12,STAMBP,SVIP,IL36RN,THBD,ADORA2A,NPHP4,ZNF366,RGS11,NDUFAF2,C5,STAT2,PER1,DAG1,CREB3L1,SZT2,PRKAA2,MNT,ZMYND11,RAF1,RASA4B,CALCRL
regulation of response to stress	GO:0080134	1.31E-08	1273	494	38	ADORA2B,NCK2,KLHL31,CXCL6,SPIDR,TAOK1,XDH,UBQLN2,CNR2,ARL6IP5,HIC1,SLC25A23,SIRPA,AOAH,ALOX12,HDAC6,RELL1,ULK4,TXNDC12,SVIP,ANO6,RNF216,THBD,ADORA2A,MED1,NSD2,STAT2,PER1,CREB3L1,GBP5,TBXA2R,RNF8,C3,ZMYND11,CALCRL,ZMPSTE24,PINK1,FAM168A
regulation of intracellular signal transduction	GO:1902531	1.47E-08	1688	487	44	ADORA2B,NCK2,CHRNA7,ARF6,KLHL31,ARHGAP9,VWF,BMPER,HOMER3,NRP1,TAOK1,XDH,MAD2L1BP,PHB2,SLC15A3,GRM2,ARL6IP5,HIC1,SIRPA,CTNS,LPAR3,ARAF,C10ORF71,C12ORF66,DNM1L,RELL1,ULK4,MLST8,TXNDC12,STAMBP,FRMD1,PLEKHG4B,ARHGAP29,PER1,DAG1,CREB3L1,MADD,SZT2,PRKAA2,ZMYND11,RAF1,RASA4B,ZMPSTE24,PINK1
regulation of nucleobase-containing compound metabolic process	GO:0019219	2.04E-08	3945	494	74	ADORA2B,NCK2,FOXA1,TRIM33,ZNF286A,SKI,ZNF561,TADA3,ZNF506,CDA,ZNF121,SPIDR,MEIS2,SOX5,RBM24,CTBP1,PHB2,COPS2,EOMES,ZNF90,ZC3H8,INSM1,HIC1,EAPP,GLIS1,NKX2-8,MARS1,ST18,COMMD1,SHOX,HDAC6,PERM1,MPHOSPH8,RCOR2,RNPS1,CELSR2,TNRC6A,ZNF721,EBF4,FAF1,ADORA2A,MED1,NSD2,ZNF366,ZNF43,KDM1B,USP9X,PPIL4,TENT5A,ZSCAN29,ZNF318,STAT2,PER1,TCF7L1,SCML2,CREB3L1,ZNF676,RARB,RNF8,PRKAA2,CDK5R1,NEK7,MNT,ZMYND11,RAF1,ATF1,ZMPSTE24,HPCA,RPS6KA3,CREB5,PINK1,ZNF143,ZNF320,FAM168A
ion transport	GO:0006811	2.49E-08	1544	305	32	BEST1,CHRNA7,TMEM38A,SLC44A5,HOMER3,CHRNA2,NDUFS7,SLC38A1,SLC39A3,PHB2,SLC15A3,SLC17A2,TRPC4,GRM2,ARL6IP5,SLC25A23,KCNA1,CTNS,SLC30A2,PIRT,COMMD9,COMMD1,LASP1,SCN5A,KCNS3,WNK3,KCNA4,HEPH,DNM1L,IP6K2,ANO6,ADORA2A

macromolecule catabolic process	GO:0009057	3.88E-08	1341	487	38	APOB,UBA6,USP49,USP24,RBM24,GSPT1,BNIP3L,NEMF,UBQLN2,SH3GLB1,AOAH,ARAF,COMMD1,HDAC6,RNPS1,TNRC6A,NELL1,NSFL1C,RNF144A,RAB26,SVIP,UBXN2B,RNF216,FAF1,KLHDC1,HECTD1,CSDE1,FHIT,USP9X,TENT5A,FBXO33,MARCHF2,PSME2,LSM5,SMG7,RNF8,ZMPSTE24,PINK1
cell differentiation	GO:0030154	3.93E-08	4128	489	75	POLR1B,NCK2,APOB,FOXA1,ZPBP,CHRNA7,PRKX,NLGN4X,ARF6,UBA6,TMEM204,ITGB8,SKI,SH3GL2,BFSP1,MEIS2,SOX5,ADGRB3,RBM24,NRP1,TAOK1,BCL7B,CTBP1,XDH,PLK5,ARMC6,COPS2,EOMES,TRPC4,ZC3H8,INSM1,KCNA1,GLIS1,LPAR3,ANLN,NKX2-8,CPNE6,RNF112,NEXN,GPRIN2,HDAC6,PCM1,CELSR2,NELL1,PTPRO,ULK4,TPM4,ADORA2A,MED1,KRT3,NPHP4,HECTD1,FKBP6,TMEM108,LZTS1,USP9X,MAL,OGDH,BLOC1S6,LTK,PLPPR4,DAG1,CREB3L1,SZT2,RARB,RNF8,CDK5R1,C3,RAF1,ATF1,ZMPSTE24,RPS6KA3,NPHP1,SPATA16,H4C8
catabolic process	GO:0009056	4.91E-08	2547	446	52	APOB,UBA6,USP49,CDA,USP24,RBM24,GSPT1,BNIP3L,XDH,NEMF,PHB2,UBQLN2,SH3GLB1,AOAH,ARAF,CHAC2,COMMD1,WDR6,HDAC6,RNPS1,TNRC6A,NELL1,SCP2,NSFL1C,DNM1L,RNF144A,RAB26,SVIP,UBXN2B,RNF216,FAF1,KLHDC1,HECTD1,CSDE1,FHIT,LZTS1,USP9X,TENT5A,NCEH1,FBXO33,OGDH,MARCHF2,CHMP3,SLC25A44,PSME2,AGXT2,LSM5,SMG7,CARNS1,RNF8,PRKAA2,CDK5R1
organic substance catabolic process	GO:1901575	5.35E-08	2081	445	46	APOB,UBA6,USP49,CDA,USP24,RBM24,GSPT1,BNIP3L,XDH,NEMF,UBQLN2,SH3GLB1,AOAH,ARAF,CHAC2,COMMD1,HDAC6,RNPS1,TNRC6A,NELL1,SCP2,NSFL1C,RNF144A,RAB26,SVIP,UBXN2B,RNF216,FAF1,KLHDC1,HECTD1,CSDE1,FHIT,USP9X,TENT5A,NCEH1,FBXO33,OGDH,MARCHF2,SLC25A44,PSME2,AGXT2,LSM5,SMG7,CARNS1,RNF8,PRKAA2
cellular protein localization	GO:0034613	6.00E-08	1954	492	47	ARF6,PDIA2,HOMER3,SPIDR,DHRS1,RANBP2,NRP1,CEP72,BNIP3L,TIMM10,ZDHHC4,PRAM1,PHB2,CROCC,SH3GLB1,ARL6IP5,KCNA1,UNC50,COMMD1,HDAC6,PCM1,SCP2,C12ORF66,NSFL1C,WNK3,DNM1L,BICD1,RAB26,SVIP,UBXN2B,EXOC6B,MED1,NPHP4,HECTD1,MYO1C,MAL,EHD3,DAG1,GBP5,SZT2,CDK5R1,HPCA,NPHP1,ZWILCH,KRT18,PINK1,SNX31
cytoskeleton organization	GO:0007010	6.71E-08	1435	486	39	NCK2,ARF6,CCDC6,DRC1,BFSP1,NRP1,TAOK1,CEP72,FSCN1,STRIP1,CROCC,EML4,ANLN,NEXN,HDAC6,PCM1,NSFL1C,BICD1,ULK4,MLST8,GOLGA8B,UBXN2B,TPM4,KRT3,NPHP4,SHROOM2,FRMD5,MYO1C,CHMP3,DAG1,STAG1,PRKAA2,CDK5R1,NEK7,RAF1,FMNL3,LIMA1,NPHP1,KRT18
cellular macromolecule localization	GO:0070727	7.00E-08	1963	492	47	ARF6,PDIA2,HOMER3,SPIDR,DHRS1,RANBP2,NRP1,CEP72,BNIP3L,TIMM10,ZDHHC4,PRAM1,PHB2,CROCC,SH3GLB1,ARL6IP5,KCNA1,UNC50,COMMD1,HDAC6,PCM1,SCP2,C12ORF66,NSFL1C,WNK3,DNM1L,BICD1,RAB26,SVIP,UBXN2B,EXOC6B,MED1,NPHP4,HECTD1,MYO1C,MAL,EHD3,DAG1,GBP5,SZT2,CDK5R1,HPCA,NPHP1,ZWILCH,KRT18,PINK1,SNX31

neuron development	GO:0048666	1.02E-07	1104	473	33	NCK2,CHRNA7,ARF6,UBA6,SH3GL2,ADGRB3,NRP1,TAOK1,PLK5,INSM1,LPAR3,NKX2-8,CPNE6,NEXN,GPRIN2,HDAC6,CELSR2,PTPRO,ULK4,ADORA2A,NPHP4,TMEM108,LZTS1,USP9X,OGDH,BLOC1S6,LTK,PLPPR4,DAG1,SZT2,CDK5R1,C3,ATF1
cellular macromolecule catabolic process	GO:0044265	1.07E-07	1135	487	34	UBA6,USP49,USP24,RBM24,GSPT1,BNIP3L,NEMF,UBQLN2,ARAF,COMMD1,HDAC6,RNPS1,TNRC6A,NELL1,NSFL1C,RNF144A,SVIP,UBXN2B,RNF216,FAF1,KLHDC1,HECTD1,CSDE1,FHIT,USP9X,TENT5A,FBXO33,MARCHF2,PSME2,LSM5,SMG7,RNF8,ZMPSTE24,PINK1
organic cyclic compound metabolic process	GO:1901360	1.23E-07	8395	494	121	POLR1B,ADORA2B,NCK2,APOB,FANCM,FOXA1,TRIM33,GINS1,USP49,ZNF286A,SKI,ZNF561,HPDL,INO80B,TOE1,SMC6,TSEN2,TADA3,ZNF506,CDA,ZNF121,SPIDR,MACROD2,MEIS2,ADCY4,SOX5,RBM24,TAOK1,GSPT1,CTBP1,EEPD1,CARS1,XDH,POLN,PHB2,COPS2,MOCS1,RNU6-137P,EOMES,OSBPL5,ZNF90,ZC3H8,INSM1,HIC1,EAPP,SNORA59A,TRMT2A,CTNS,GLIS1,ALOX12,NKX2-8,PRPSAP2,MARS1,ST18,COMMD1,WDR6,SHOX,TEFM,HDAC6,PERM1,MPHOSPH8,RCOR2,RNPS1,CELSR2,TNRC6A,SCP2,ZNF721,BICD1,EBF4,NOP9,FAF1,ADORA2A,MED1,NSD2,ZNF366,SRRM2,CD320,FKBP6,CSDE1,FHIT,ZNF43,CMPK1,KDM1B,USP9X,PPIL4,TENT5A,ZSCAN29,OGDH,ZNF318,STAT2,PER1,TCF7L1,SCML2,LSM5,SMG7,CREB3L1,ZNF676,RARB,SUCLA2,RNF8,PRKAA2,CDK5R1,NEK7,MNT,ZMYND11,CSKMT,POLE,RAF1,CTRC,HARBI1,LIMA1,ATF1,ZMPSTE24,HPCA,RPS6KA3,CREB5,PINK1,ZNF143,H4C8,ZNF320,FAM168A
central nervous system development	GO:0007417	1.28E-07	1040	478	32	HAPLN1,NLGN4X,UBA6,SKI,SH3GL2,MACROD2,MEIS2,NRP1,EOMES,TRPC4,KCNA1,CTNS,EPOR,PTCHD1,PCM1,CELSR2,SCN5A,ADORA2A,MED1,DHX30,VIT,TMEM108,SHROOM2,MAL,OGDH,DAG1,SZT2,RARB,CDK5R1,C3,HPCA,RPS6KA3
regulation of molecular function	GO:0065009	1.40E-07	3017	487	60	ADORA2B,NCK2,FOXA1,CHRNA7,ARHGAP9,SKI,RANBP2,ADGRB3,NRP1,TAOK1,KCTD7,XDH,PHB2,EOMES,GRM2,CNR2,ARL6IP5,SIRPA,KCNA1,PRPSAP2,PIRT,ST18,COMMD1,TEFM,HDAC6,NPR3,WNK3,DNM1L,BICD1,PTPRO,MLST8,VCPKMT,FAF1,ADORA2A,CDC25A,A2ML1,RGS11,PLEKHG4B,DENND2D,C5,ARHGAP29,LTK,EHD3,PSME2,PDE6D,DAG1,MADD,TBXA2R,SZT2,EPM2AIP1,CDK5R1,NEK7,C3,RAF1,RASA4B,ITIH5,ZMPSTE24,HPCA,RPS6KA3,PINK1
negative regulation of cell communication	GO:0010648	1.43E-07	1347	461	36	NCK2,NLGN4X,TRIM33,KLHL31,SKI,BMPER,HOMER3,NRP1,XDH,MAD2L1BP,PHB2,UBQLN2,CNR2,HIC1,SIRPA,C12ORF66,BICD1,PTPRO,TXNDC12,STAMBP,IL36RN,NPHP4,ZNF366,RGS11,NDUFAF2,GRID2IP,STAT2,PER1,DAG1,CREB3L1,SZT2,PRKAA2,MNT,ZMYND11,RAF1,RASA4B

negative regulation of signaling	GO:0023057	1.52E-07	1350	461	36	NCK2,NLGN4X,TRIM33,KLHL31,SKI,BMPER,HOMER3,NRP1,XDH,MAD2L1BP,PHB2,UBQLN2,CNR2,HIC1,SIRPA,C12ORF66,BICD1,PTPRO,TXNDC12,STAMBP,IL36RN,NPHP4,ZNF366,RGS11,NDUFAF2,GRID2IP,STAT2,PER1,DAG1,CREB3L1,SZT2,PRKAA2,MNT,ZMYND11,RAF1,RASA4B
transcription, DNA-templated	GO:0006351	1.58E-07	3551	493	67	POLR1B,NCK2,FOXA1,TRIM33,ZNF286A,SKI,ZNF561,TADA3,ZNF506,ZNF121,MEIS2,SOX5,CTBP1,PHB2,COPS2,EOMES,ZNF90,ZC3H8,INSM1,HIC1,EAPP,GLIS1,NKX2-8,MARS1,ST18,COMMD1,SHOX,TEFM,HDAC6,PERM1,MPHOSPH8,RCOR2,RNPS1,CELSR2,ZNF721,EBF4,FAF1,ADORA2A,MED1,NSD2,ZNF366,ZNF43,KDM1B,USP9X,PPIL4,ZSCAN29,ZNF318,STAT2,PER1,TCF7L1,SCML2,CREB3L1,ZNF676,RARB,RNF8,CDK5R1,MNT,ZMYND11,RAF1,ATF1,ZMPSTE24,RPS6KA3,CREB5,PINK1,ZNF143,H4C8,ZNF320
nucleic acid-templated transcription	GO:0097659	1.60E-07	3552	493	67	POLR1B,NCK2,FOXA1,TRIM33,ZNF286A,SKI,ZNF561,TADA3,ZNF506,ZNF121,MEIS2,SOX5,CTBP1,PHB2,COPS2,EOMES,ZNF90,ZC3H8,INSM1,HIC1,EAPP,GLIS1,NKX2-8,MARS1,ST18,COMMD1,SHOX,TEFM,HDAC6,PERM1,MPHOSPH8,RCOR2,RNPS1,CELSR2,ZNF721,EBF4,FAF1,ADORA2A,MED1,NSD2,ZNF366,ZNF43,KDM1B,USP9X,PPIL4,ZSCAN29,ZNF318,STAT2,PER1,TCF7L1,SCML2,CREB3L1,ZNF676,RARB,RNF8,CDK5R1,MNT,ZMYND11,RAF1,ATF1,ZMPSTE24,RPS6KA3,CREB5,PINK1,ZNF143,H4C8,ZNF320
RNA biosynthetic process	GO:0032774	1.93E-07	3568	493	67	POLR1B,NCK2,FOXA1,TRIM33,ZNF286A,SKI,ZNF561,TADA3,ZNF506,ZNF121,MEIS2,SOX5,CTBP1,PHB2,COPS2,EOMES,ZNF90,ZC3H8,INSM1,HIC1,EAPP,GLIS1,NKX2-8,MARS1,ST18,COMMD1,SHOX,TEFM,HDAC6,PERM1,MPHOSPH8,RCOR2,RNPS1,CELSR2,ZNF721,EBF4,FAF1,ADORA2A,MED1,NSD2,ZNF366,ZNF43,KDM1B,USP9X,PPIL4,ZSCAN29,ZNF318,STAT2,PER1,TCF7L1,SCML2,CREB3L1,ZNF676,RARB,RNF8,CDK5R1,MNT,ZMYND11,RAF1,ATF1,ZMPSTE24,RPS6KA3,CREB5,PINK1,ZNF143,H4C8,ZNF320
metal ion transport	GO:0030001	1.99E-07	880	477	29	BEST1,CHRNA7,TMEM38A,HOMER3,SLC38A1,SLC39A3,SLC17A2,TRPC4,SLC25A23,KCNA1,SLC30A2,COMMD9,COMMD1,SCN5A,KCNS3,WNK3,KCNA4,HEPH,DNM1L,ANO6,ADORA2A,SLC5A10,SHROOM2,SCN8A,EHD3,PER1,CALCRL,ZMPSTE24,HPCA
cell surface receptor signaling pathway	GO:0007166	2.09E-07	2783	465	55	ADORA2B,NCK2,FOXA1,CHRNA7,NLGN4X,TRIM33,TMEM204,ITGB8,CXCL6,SKI,FCHO1,BMPER,HOMER3,CDA,ADGRB3,CHRNA2,TEC,NRP1,BCL7B,XDH,PRAM1,PHB2,GRM2,SIRPB1,HIC1,ST18,EPOR,PTCHD1,CELSR2,FCGR1B,PTPRO,STAMBP,ANO6,IL36RN,FAF1,ADORA2A,MED1,NPHP4,TMEM108,C5,MYO1C,USP9X,MAL,NCEH1,LTK,STAT2,TCF7L1,CREB3L1,MADD,PRKAA2,CDK5R1,C3,ZMYND11,RAF1,CALCRL

cell death	GO:0008219	2.32E-07	2057	487	47	NCK2,FOXA1,CHRNA7,ARF6,NRP1,TAOK1,BCL7B,BNIP3L,PLK5,PHB2,SH3GLB1,ZC3H8,ARL6IP5,HIC1,ALOX12,ARAF,RNF112,HDAC6,RNPS1,WNK3,DNM1L,TXNDC12,STAMBP,BTBD10,IP6K2,ANO6,RNF216,FAF1,ADORA2A,MED1,NAA38,FHIT,MAL,LTK,CHMP3,CREB3L1,MADD,RARB,PRKAA2,CDK5R1,MNT,ZMYND11,RAF1,ZMPSTE24,RPS6KA3,KRT18,PINK1
regulation of phosphate metabolic process	GO:0019220	2.58E-07	1367	487	37	ADORA2B,NCK2,CHRNA7,KLHL31,SH3GL2,BMPER,TADA3,CDA,RANBP2,TEC,NRP1,TAOK1,XDH,PHB2,INSM1,SIRPA,ARAF,HDAC6,SCP2,WNK3,PTPRO,MLST8,BTBD10,ADORA2A,CDC25A,PPIL4,LTK,STAT2,DAG1,SMG7,MADD,PRKAA2,CDK5R1,C3,RAF1,HPCA,PINK1
regulation of phosphorus metabolic process	GO:0051174	2.68E-07	1369	487	37	ADORA2B,NCK2,CHRNA7,KLHL31,SH3GL2,BMPER,TADA3,CDA,RANBP2,TEC,NRP1,TAOK1,XDH,PHB2,INSM1,SIRPA,ARAF,HDAC6,SCP2,WNK3,PTPRO,MLST8,BTBD10,ADORA2A,CDC25A,PPIL4,LTK,STAT2,DAG1,SMG7,MADD,PRKAA2,CDK5R1,C3,RAF1,HPCA,PINK1
plasma membrane bounded cell projection organization	GO:0120036	2.88E-07	1479	474	38	NCK2,CHRNA7,ARF6,UBA6,SH3GL2,DRC1,ADGRB3,NRP1,TAOK1,PLK5,FSCN1,CROCC,LPAR3,ANLN,NKX2-8,CPNE6,NEXN,GPRIN2,HDAC6,PCM1,CELSR2,PTPRO,ULK4,ANO6,ADORA2A,TMEM108,LZTS1,USP9X,BLOC1S6,LTK,EHD3,PLPPR4,DAG1,SZT2,CDK5R1,LIMA1,ATF1,CILK1
cell projection organization	GO:0030030	3.00E-07	1521	483	39	NCK2,CHRNA7,ARF6,UBA6,SH3GL2,DRC1,ADGRB3,NRP1,TAOK1,PLK5,FSCN1,CROCC,LPAR3,ANLN,NKX2-8,CPNE6,NEXN,GPRIN2,HDAC6,PCM1,CELSR2,PTPRO,ULK4,ANO6,ADORA2A,TMEM108,LZTS1,USP9X,BLOC1S6,LTK,EHD3,PLPPR4,DAG1,SZT2,CDK5R1,LIMA1,ATF1,CILK1,NPHP1
anatomical structure morphogenesis	GO:0009653	3.22E-07	2685	487	55	POLR1B,APOB,FOXA1,ZPBP,CHRNA7,PRKX,ITGB8,SKI,SH3GL2,BMPER,MEIS2,SOX5,ADGRB3,NRP1,TAOK1,XDH,SLC39A3,PHB2,STRIP1,EOMES,LPAR3,NKX2-8,CPNE6,NEXN,HDAC6,CELSR2,SCN5A,DNM1L,BICD1,PTPRO,ADORA2A,MED1,NSD2,HECTD1,TMEM108,SHROOM2,LZTS1,C5,USP9X,STAT2,PLPPR4,SCML2,DAG1,CREB3L1,TBXA2R,SZT2,RARB,CDK5R1,C3,FMNL3,CALCRL,ZMPSTE24,NPHP1,KRT18,PINK1
movement of cell or subcellular component	GO:0006928	3.94E-07	2074	474	46	NCK2,APOB,PRKX,ARF6,ITGB8,CXCL6,SKI,BMPER,DRC1,NRP1,FSCN1,PHB2,CNR2,AQP4,INSM1,SIRPA,ANLN,NEXN,HDAC6,DNHD1,PCM1,PODN,CELSR2,SCN5A,EFCAB1,DNM1L,BICD1,PTPRO,ULK4,ANO6,NPHP4,TMEM108,SHROOM2,C5,FRMD5,MYO1C,USP9X,OGDH,BLOC1S6,DAG1,TBXA2R,CDK5R1,RAF1,FMNL3,LIMA1,CILK1

cellular nitrogen compound metabolic process	GO:0034641	4.50E-07	8767	494	123	POLR1B,ADORA2B,NCK2,FANCM,FOXA1,CHRNA7,TRIM33,SMS,GINS1,USP49,ZNF286A,ITGB8,SKI,ZNF561,INO80B,TOE1,SMC6,TSEN2,TADA3,ZNF506,CDA,ZNF121,SPIDR,MACROD2,MEIS2,ADCY4,SOX5,RBM24,TAOK1,GSPT1,CTBP1,EEPD1,CARS1,XDH,POLN,NEMF,PHB2,COPS2,RNU6-137P,EOMES,ZNF90,ZC3H8,ARL6IP5,INSM1,HIC1,EAPP,SNORA59A,SIRPA,TRMT2A,CTNS,GLIS1,NKX2-8,PRPSAP2,MARS1,CHAC2,ST18,COMMD1,WDR6,SHOX,TEFM,HDAC6,PERM1,MPHOSPH8,RCOR2,RNPS1,CELSR2,TNRC6A,ZNF721,BICD1,EBF4,NOP9,FAF1,ADORA2A,MED1,NSD2,ZNF366,SRRM2,FKBP6,CSDE1,FHIT,ZNF43,CMPK1,KDM1B,USP9X,PPIL4,TENT5A,ZSCAN29,OGDH,ZNF318,STAT2,PER1,RPL15,AGXT2,TCF7L1,SCML2,LSM5,SMG7,CARNS1,CREB3L1,ZNF676,RARB,SUCLA2,PLPP2,RNF8,PRKAA2,CDK5R1,NEK7,MNT,ZMYND11,CSKMT,POLE,RAF1,HARBI1,ATF1,ZMPSTE24,HPCA,RPS6KA3,CREB5,PINK1,ZNF143,H4C8,ZNF320,FAM168A
regulation of multicellular organismal process	GO:0051239	4.65E-07	2623	489	54	ADORA2B,FOXA1,CHRNA7,PRKX,NLGN4X,ARF6,ITGB8,CXCL6,SKI,TMEM38A,BMPER,HOMER3,MEIS2,SOX5,ADGRB3,NRP1,XDH,PHB2,AQP4,ZC3H8,SIRPA,KCNA1,LPAR3,ALOX12,IP6K1,RNF112,HDAC6,PCM1,SCN5A,NELL1,NPR3,PTPRO,ANO6,RNF216,IL36RN,THBD,ADORA2A,MED1,NSD2,TMEM108,C5,SLC25A44,EHD3,PER1,DAG1,CREB3L1,GBP5,TBXA2R,RARB,CDK5R1,C3,CALCRL,ZMPSTE24,H4C8
protein phosphorylation	GO:0006468	5.85E-07	1544	487	39	NCK2,CHRNA7,PRKX,KLHL31,SH3GL2,BMPER,TADA3,TEC,NRP1,TAOK1,CTBP1,XDH,PLK5,PHB2,COPS2,INSM1,SIRPA,ARAF,HDAC6,WNK3,PTPRO,ULK4,MLST8,ADORA2A,CDC25A,PPIL4,LTK,STAT2,DAG1,MADD,PRKAA2,CDK5R1,NEK7,C3,RAF1,CILK1,ZMPSTE24,RPS6KA3,PINK1
proteolysis	GO:0006508	6.02E-07	1755	487	42	UBA6,USP49,USP24,LDLRAD3,FAM111B,XDH,NEMF,COPS2,UBQLN2,ADAM30,ARL6IP5,PGA5,ARAF,ST18,COMMD1,HDAC6,NSFL1C,RNF144A,STAMBP,SVIP,UBXN2B,RNF216,FAF1,ADORA2A,KLHDC1,A2ML1,HECTD1,FHIT,C5,USP9X,FBXO33,RHBDL2,PSME2,DAG1,RNF8,C3,RAF1,CTRC,ITIH5,ZMPSTE24,RPS6KA3,PINK1
regulation of macromolecule biosynthetic process	GO:0010556	6.51E-07	3926	493	70	NCK2,FOXA1,TRIM33,ZNF286A,SKI,ZNF561,TADA3,ZNF506,ZNF121,MEIS2,SOX5,RBM24,GSPT1,CTBP1,PHB2,COPS2,EOMES,ZNF90,ZC3H8,INSM1,HIC1,EAPP,GLIS1,NKX2-8,MARS1,ST18,COMMD1,SHOX,HDAC6,PERM1,MPHOSPH8,RCOR2,CELSR2,TNRC6A,ZNF721,EBF4,SVIP,FAF1,ADORA2A,MED1,NSD2,ZNF366,CSDE1,ZNF43,KDM1B,USP9X,PPIL4,ZSCAN29,ZNF318,STAT2,PER1,TCF7L1,SCML2,CREB3L1,EPM2AIP1,ZNF676,RARB,RNF8,CDK5R1,NEK7,MNT,ZMYND11,RAF1,ATF1,ZMPSTE24,RPS6KA3,CREB5,PINK1,ZNF143,ZNF320

programmed cell death	GO:0012501	7.04E-07	1908	487	44	NCK2,FOXA1,ARF6,NRP1,TAOK1,BCL7B,BNIP3L,PLK5,PHB2,SH3GLB1,ZC3H8,ARL6IP5,HIC1,ALOX12,ARAF,HDAC6,RNPS1,WNK3,DNM1L,TXNDC12,STAMBP,IP6K2,ANO6,RNF216,FAF1,ADORA2A,MED1,NAA38,FHIT,MAL,LTK,CHMP3,CREB3L1,MADD,RARB,PRKAA2,CDK5R1,MNT,ZMYND11,RAF1,ZMPSTE24,RPS6KA3,KRT18,PINK1
positive regulation of response to stimulus	GO:0048584	7.19E-07	2111	458	45	ADORA2B,NCK2,FOXA1,CHRNA7,SKI,VWF,FCHO1,BMPER,SPIDR,TEC,NRP1,TAOK1,XDH,PRAM1,PHB2,SLC15A3,UBQLN2,ARL6IP5,HIC1,CTNS,C8B,LPAR3,C10ORF71,HDAC6,DNM1L,RELL1,CFP,MLST8,ANO6,FAF1,THBD,ADORA2A,MED1,NSD2,FRMD1,TMEM108,C5,MYO1C,MAL,GBP5,MADD,TBXA2R,RNF8,C3,RAF1
regulation of RNA metabolic process	GO:0051252	8.71E-07	3700	493	67	NCK2,FOXA1,TRIM33,ZNF286A,SKI,ZNF561,TADA3,ZNF506,ZNF121,MEIS2,SOX5,RBM24,CTBP1,PHB2,COPS2,EOMES,ZNF90,ZC3H8,INSM1,HIC1,EAPP,GLIS1,NKX2-8,MARS1,ST18,COMMD1,SHOX,HDAC6,PERM1,MPHOSPH8,RCOR2,RNPS1,CELSR2,TNRC6A,ZNF721,EBF4,FAF1,ADORA2A,MED1,NSD2,ZNF366,ZNF43,KDM1B,USP9X,PPIL4,TENT5A,ZSCAN29,ZNF318,STAT2,PER1,TCF7L1,SCML2,CREB3L1,ZNF676,RARB,RNF8,CDK5R1,MNT,ZMYND11,RAF1,ATF1,ZMPSTE24,RPS6KA3,CREB5,PINK1,ZNF143,ZNF320
apoptotic process	GO:0006915	9.96E-07	1857	487	43	NCK2,FOXA1,ARF6,NRP1,TAOK1,BCL7B,BNIP3L,PLK5,PHB2,SH3GLB1,ZC3H8,ARL6IP5,HIC1,ALOX12,ARAF,RNPS1,WNK3,DNM1L,TXNDC12,STAMBP,IP6K2,ANO6,RNF216,FAF1,ADORA2A,MED1,NAA38,FHIT,MAL,LTK,CHMP3,CREB3L1,MADD,RARB,PRKAA2,CDK5R1,MNT,ZMYND11,RAF1,ZMPSTE24,RPS6KA3,KRT18,PINK1
neuron projection development	GO:0031175	1.02284E-06	953	473	29	NCK2,CHRNA7,ARF6,UBA6,SH3GL2,ADGRB3,NRP1,TAOK1,PLK5,LPAR3,NKX2-8,CPNE6,NEXN,GPRIN2,HDAC6,CELSR2,PTPRO,ULK4,ADORA2A,TMEM108,LZTS1,USP9X,BLOC1S6,LTK,PLPPR4,DAG1,SZT2,CDK5R1,ATF1
negative regulation of signal transduction	GO:0009968	1.04482E-06	1242	461	33	NCK2,NLGN4X,TRIM33,KLHL31,SKI,BMPER,HOMER3,NRP1,XDH,MAD2L1BP,PHB2,UBQLN2,HIC1,SIRPA,C12ORF66,BICD1,PTPRO,TXNDC12,STAMBP,IL36RN,NPHP4,ZNF366,RGS11,STAT2,PER1,DAG1,CREB3L1,SZT2,PRKAA2,MNT,ZMYND11,RAF1,RASA4B
cell-cell signaling	GO:0007267	1.04586E-06	1646	487	40	ADORA2B,FOXA1,CHRNA7,NLGN4X,CXCL6,SKI,CHRNA2,SYP,DGKB,NRP1,BCL7B,GRM2,CNR2,HIC1,KCNA1,LPAR3,CPNE6,CHAT,PTCHD1,CELSR2,SCN5A,PTPRO,RAB26,ADORA2A,BAIAP3,NPHP4,NDUFAF2,TMEM108,GRID2IP,LZTS1,BLOC1S6,TCF7L1,PLPPR4,DAG1,PRKAA2,CDK5R1,C3,RAF1,RPS6KA3,PINK1

heterocycle metabolic process	GO:0046483	1.05441E-06	8099	494	115	POLR1B,ADORA2B,NCK2,FANCM,FOXA1,TRIM33,GINS1,USP49,ZNF286A,SKI,ZNF561,INO80B,TOE1,SMC6,TSEN2,TADA3,ZNF506,CDA,ZNF121,SPIDR,MACROD2,MEIS2,ADCY4,SOX5,RBM24,TAOK1,GSPT1,CTBP1,EEPD1,CARS1,XDH,POLN,PHB2,COPS2,MOCS1,RNU6-137P,EOMES,ZNF90,ZC3H8,INSM1,HIC1,EAPP,SNORA59A,TRMT2A,GLIS1,ALOX12,NKX2-8,PRPSAP2,MARS1,ST18,COMMD1,WDR6,SHOX,TEFM,HDAC6,PERM1,MPHOSPH8,RCOR2,RNPS1,CELSR2,TNRC6A,ZNF721,BICD1,EBF4,NOP9,FAF1,ADORA2A,MED1,NSD2,ZNF366,SRRM2,CD320,FKBP6,CSDE1,FHIT,ZNF43,CMPK1,KDM1B,USP9X,PPIL4,TENT5A,ZSCAN29,OGDH,ZNF318,STAT2,PER1,TCF7L1,SCML2,LSM5,SMG7,CREB3L1,ZNF676,RARB,SUCLA2,RNF8,PRKAA2,CDK5R1,NEK7,MNT,ZMYND11,CSKMT,POLE,RAF1,CTRC,HARBI1,ATF1,ZMPSTE24,HPCA,RPS6KA3,CREB5,PINK1,ZNF143,H4C8,ZNF320,FAM168A
regulation of catalytic activity	GO:0050790	1.16412E-06	2314	487	49	ADORA2B,NCK2,CHRNA7,ARHGAP9,RANBP2,ADGRB3,NRP1,TAOK1,XDH,PHB2,GRM2,CNR2,ARL6IP5,SIRPA,PRPSAP2,ST18,TEFM,HDAC6,NPR3,DNM1L,BICD1,PTPRO,MLST8,FAF1,ADORA2A,CDC25A,A2ML1,RGS11,PLEKHG4B,DENND2D,C5,ARHGAP29,LTK,PSME2,PDE6D,DAG1,MADD,TBXA2R,SZT2,EPM2AIP1,CDK5R1,NEK7,C3,RAF1,RASA4B,ITIH5,HPCA,RPS6KA3,PINK1
cell motility	GO:0048870	1.18063E-06	1645	469	39	NCK2,APOB,PRKX,ARF6,ITGB8,CXCL6,SKI,BMPER,DRC1,NRP1,FSCN1,PHB2,CNR2,INSM1,SIRPA,ANLN,NEXN,HDAC6,PCM1,PODN,CELSR2,EFCAB1,DNM1L,PTPRO,ULK4,ANO6,NPHP4,SHROOM2,C5,FRMD5,MYO1C,USP9X,OGDH,DAG1,TBXA2R,CDK5R1,RAF1,FMNL3,LIMA1
localization of cell	GO:0051674	1.18063E-06	1645	469	39	NCK2,APOB,PRKX,ARF6,ITGB8,CXCL6,SKI,BMPER,DRC1,NRP1,FSCN1,PHB2,CNR2,INSM1,SIRPA,ANLN,NEXN,HDAC6,PCM1,PODN,CELSR2,EFCAB1,DNM1L,PTPRO,ULK4,ANO6,NPHP4,SHROOM2,C5,FRMD5,MYO1C,USP9X,OGDH,DAG1,TBXA2R,CDK5R1,RAF1,FMNL3,LIMA1
regulation of transcription, DNA-templated	GO:0006355	1.44468E-06	3412	493	63	NCK2,FOXA1,TRIM33,ZNF286A,SKI,ZNF561,TADA3,ZNF506,ZNF121,MEIS2,SOX5,CTBP1,PHB2,COPS2,EOMES,ZNF90,ZC3H8,INSM1,HIC1,EAPP,GLIS1,NKX2-8,MARS1,ST18,COMMD1,SHOX,HDAC6,PERM1,MPHOSPH8,RCOR2,CELSR2,ZNF721,EBF4,FAF1,ADORA2A,MED1,NSD2,ZNF366,ZNF43,KDM1B,USP9X,PPIL4,ZSCAN29,ZNF318,STAT2,PER1,TCF7L1,SCML2,CREB3L1,ZNF676,RARB,RNF8,CDK5R1,MNT,ZMYND11,RAF1,ATF1,ZMPSTE24,RPS6KA3,CREB5,PINK1,ZNF143,ZNF320

regulation of nucleic acid-templated transcription	GO:1903506	1.46124E-06	3413	493	63	NCK2,FOXA1,TRIM33,ZNF286A,SKI,ZNF561,TADA3,ZNF506,ZNF121,MEIS2,SOX5,CTBP1,PHB2,COPS2,EOMES,ZNF90,ZC3H8,INSM1,HIC1,EAPP,GLIS1,NKX2-8,MARS1,ST18,COMMD1,SHOX,HDAC6,PERM1,MPHOSPH8,RCOR2,CELSR2,ZNF721,EBF4,FAF1,ADORA2A,MED1,NSD2,ZNF366,ZNF43,KDM1B,USP9X,PPIL4,ZSCAN29,ZNF318,STAT2,PER1,TCF7L1,SCML2,CREB3L1,ZNF676,RARB,RNF8,CDK5R1,MNT,ZMYND11,RAF1,ATF1,ZMPSTE24,RPS6KA3,CREB5,PINK1,ZNF143,ZNF320
regulation of RNA biosynthetic process	GO:2001141	1.54678E-06	3418	493	63	NCK2,FOXA1,TRIM33,ZNF286A,SKI,ZNF561,TADA3,ZNF506,ZNF121,MEIS2,SOX5,CTBP1,PHB2,COPS2,EOMES,ZNF90,ZC3H8,INSM1,HIC1,EAPP,GLIS1,NKX2-8,MARS1,ST18,COMMD1,SHOX,HDAC6,PERM1,MPHOSPH8,RCOR2,CELSR2,ZNF721,EBF4,FAF1,ADORA2A,MED1,NSD2,ZNF366,ZNF43,KDM1B,USP9X,PPIL4,ZSCAN29,ZNF318,STAT2,PER1,TCF7L1,SCML2,CREB3L1,ZNF676,RARB,RNF8,CDK5R1,MNT,ZMYND11,RAF1,ATF1,ZMPSTE24,RPS6KA3,CREB5,PINK1,ZNF143,ZNF320
cellular aromatic compound metabolic process	GO:0006725	1.62939E-06	8159	494	115	POLR1B,ADORA2B,NCK2,FANCM,FOXA1,TRIM33,GINS1,USP49,ZNF286A,SKI,ZNF561,HPDL,INO80B,TOE1,SMC6,TSEN2,TADA3,ZNF506,CDA,ZNF121,SPIDR,MACROD2,MEIS2,ADCY4,SOX5,RBM24,TAOK1,GSPT1,CTBP1,EEPD1,CARS1,XDH,POLN,PHB2,COPS2,RNU6-137P,EOMES,ZNF90,ZC3H8,INSM1,HIC1,EAPP,SNORA59A,TRMT2A,CTNS,GLIS1,NKX2-8,PRPSAP2,MARS1,ST18,COMMD1,WDR6,SHOX,TEFM,HDAC6,PERM1,MPHOSPH8,RCOR2,RNPS1,CELSR2,TNRC6A,ZNF721,BICD1,EBF4,NOP9,FAF1,ADORA2A,MED1,NSD2,ZNF366,SRRM2,CD320,FKBP6,CSDE1,FHIT,ZNF43,CMPK1,KDM1B,USP9X,PPIL4,TENT5A,ZSCAN29,OGDH,ZNF318,STAT2,PER1,TCF7L1,SCML2,LSM5,SMG7,CREB3L1,ZNF676,RARB,SUCLA2,RNF8,PRKAA2,CDK5R1,NEK7,MNT,ZMYND11,CSKMT,POLE,RAF1,CTRC,HARBI1,ATF1,ZMPSTE24,HPCA,RPS6KA3,CREB5,PINK1,ZNF143,H4C8,ZNF320,FAM168A
negative regulation of nucleobase-containing compound metabolic process	GO:0045934	1.68915E-06	1496	477	37	NCK2,FOXA1,TRIM33,SKI,CDA,MEIS2,RBM24,CTBP1,PHB2,COPS2,EOMES,ZC3H8,INSM1,HIC1,EAPP,GLIS1,HDAC6,MPHOSPH8,RCOR2,RNPS1,MED1,NSD2,ZNF366,KDM1B,USP9X,TENT5A,ZNF318,PER1,SCML2,CREB3L1,RARB,RNF8,CDK5R1,MNT,ZMYND11,ZMPSTE24,HPCA
regulation of protein localization	GO:0032880	1.78552E-06	829	487	27	ARF6,SPIDR,CEP72,BNIP3L,CROCC,SH3GLB1,COMMD1,HDAC6,PCM1,CELSR2,NSFL1C,WNK3,DNM1L,BICD1,SVIP,UBXN2B,ADORA2A,BAIAP3,NSD2,HECTD1,NDUFAF2,MYO1C,DAG1,PRKAA2,CDK5R1,HPCA,PINK1

homeostatic process	GO:0042592	2.70117E-06	1774	487	41	APOB,FOXA1,CHRNA7,CXCL6,SH3GL2,TMEM38A,TAOK1,KCTD7,SLC39A3,CROCC,TRPC4,GRM2,AQP4,ZC3H8,SLC25A23,KCNA1,LPAR3,ALOX12,IP6K1,SLC30A2,COMMD9,COMMD1,SCN5A,NPR3,WNK3,HEPH,ANO6,ADORA2A,MED1,BAIAP3,NPHP4,COX19,NDUFAF2,ABCA4,SLC25A44,TBXA2R,PRKAA2,RAF1,CTRC,LIMA1,PINK1
negative regulation of RNA metabolic process	GO:0051253	3.03557E-06	1395	475	35	NCK2,FOXA1,TRIM33,SKI,MEIS2,RBM24,CTBP1,PHB2,COPS2,EOMES,ZC3H8,INSM1,HIC1,EAPP,GLIS1,HDAC6,MPHOSPH8,RCOR2,RNPS1,MED1,NSD2,ZNF366,KDM1B,USP9X,TENT5A,ZNF318,PER1,SCML2,CREB3L1,RARB,RNF8,CDK5R1,MNT,ZMYND11,ZMPSTE24
synaptic signaling	GO:0099536	3.23747E-06	736	487	25	ADORA2B,CHRNA7,NLGN4X,CHRNA2,SYP,DGKB,GRM2,CNR2,KCNA1,LPAR3,CPNE6,CHAT,PTCHD1,RAB26,ADORA2A,BAIAP3,TMEM108,GRID2IP,LZTS1,BLOC1S6,PLPPR4,DAG1,CDK5R1,RPS6KA3,PINK1
small molecule metabolic process	GO:0044281	3.50144E-06	1779	490	41	ADORA2B,APOB,SMS,HPDL,CDA,MACROD2,RANBP2,ADCY4,CARS1,XDH,SDHB,OSBPL5,CTNS,AOAH,ALOX12,IP6K1,PRPSAP2,MARS1,SCP2,GALK2,IP6K2,CD320,FHIT,CMPK1,NCEH1,OGDH,SLC25A44,AGXT2,MRPS36,CARNS1,EPM2AIP1,SUCLA2,PLPP2,PRKAA2,C3,CTRC,LIMA1,ZMPSTE24,HPCA,PINK1,LGSN
cell cycle process	GO:0022402	5.36025E-06	1194	485	32	FANCM,GINS1,TTC19,TAOK1,GSPT1,CEP72,PLK5,MAD2L1BP,PHB2,CROCC,SH3GLB1,INSM1,EML4,ANLN,RNF112,WDR6,PCM1,NSFL1C,STAMBP,GOLGA8B,UBXN2B,MED1,CDC25A,LZTS1,CHMP3,PSME2,STAG1,CDK5R1,NEK7,POLE,ZMPSTE24,ZWILCH
microtubule-based process	GO:0007017	5.4597E-06	898	474	27	APOB,DRC1,TAOK1,CEP72,CROCC,AQP4,EML4,HDAC6,DNHD1,PCM1,CELSR2,NSFL1C,EFCAB1,BICD1,ULK4,GOLGA8B,UBXN2B,NPHP4,TMEM108,BLOC1S6,CHMP3,DAG1,STAG1,PRKAA2,CDK5R1,NEK7,CILK1
nucleobase-containing compound metabolic process	GO:0006139	5.60114E-06	7940	494	111	POLR1B,ADORA2B,NCK2,FANCM,FOXA1,TRIM33,GINS1,USP49,ZNF286A,SKI,ZNF561,INO80B,TOE1,SMC6,TSEN2,TADA3,ZNF506,CDA,ZNF121,SPIDR,MACROD2,MEIS2,ADCY4,SOX5,RBM24,TAOK1,GSPT1,CTBP1,EEPD1,CARS1,XDH,POLN,PHB2,COPS2,RNU6-137P,EOMES,ZNF90,ZC3H8,INSM1,HIC1,EAPP,SNORA59A,TRMT2A,GLIS1,NKX2-8,PRPSAP2,MARS1,ST18,COMMD1,WDR6,SHOX,TEFM,HDAC6,PERM1,MPHOSPH8,RCOR2,RNPS1,CELSR2,TNRC6A,ZNF721,BICD1,EBF4,NOP9,FAF1,ADORA2A,MED1,NSD2,ZNF366,SRRM2,FKBP6,CSDE1,FHIT,ZNF43,CMPK1,KDM1B,USP9X,PPIL4,TENT5A,ZSCAN29,OGDH,ZNF318,STAT2,PER1,TCF7L1,SCML2,LSM5,SMG7,CREB3L1,ZNF676,RARB,SUCLA2,RNF8,PRKAA2,CDK5R1,NEK7,MNT,ZMYND11,CSKMT,POLE,RAF1,HARBI1,ATF1,ZMPSTE24,HPCA,RPS6KA3,CREB5,PINK1,ZNF143,H4C8,ZNF320,FAM168A

ion transmembrane transport	GO:0034220	5.88402E-06	1116	260	22	BEST1,CHRNA7,TMEM38A,CHRNA2,NDUFS7,SLC39A3,PHB2,SLC15A3,SLC17A2,TRPC4,ARL6IP5,SLC25A23,KCNA1,CTNS,SLC30A2,PIRT,COMMD1,LASP1,SCN5A,KCNS3,WNK3,KCNA4
locomotion	GO:0040011	6.09749E-06	1821	469	40	NCK2,APOB,PRKX,ARF6,ITGB8,CXCL6,SKI,BMPER,DRC1,NRP1,FSCN1,PHB2,CNR2,INSM1,SIRPA,ANLN,NEXN,HDAC6,PCM1,PODN,CELSR2,EFCAB1,DNM1L,PTPRO,ULK4,ANO6,ADORA2A,NPHP4,SHROOM2,C5,FRMD5,MYO1C,USP9X,OGDH,DAG1,TBXA2R,CDK5R1,RAF1,FMNL3,LIMA1
cellular protein catabolic process	GO:0044257	6.50356E-06	821	487	26	UBA6,USP49,USP24,BNIP3L,NEMF,UBQLN2,ARAF,COMMD1,HDAC6,NELL1,NSFL1C,RNF144A,SVIP,UBXN2B,RNF216,FAF1,KLHDC1,HECTD1,FHIT,USP9X,FBXO33,MARCHF2,PSME2,RNF8,ZMPSTE24,PINK1
organonitrogen compound catabolic process	GO:1901565	6.75467E-06	1335	487	34	APOB,UBA6,USP49,CDA,USP24,BNIP3L,XDH,NEMF,UBQLN2,ARAF,CHAC2,COMMD1,HDAC6,NELL1,NSFL1C,RNF144A,RAB26,SVIP,UBXN2B,RNF216,FAF1,KLHDC1,HECTD1,FHIT,USP9X,FBXO33,MARCHF2,SLC25A44,PSME2,AGXT2,CARNS1,RNF8,ZMPSTE24,PINK1
regulation of protein modification process	GO:0031399	7.24732E-06	1477	487	36	NCK2,FANCM,CHRNA7,KLHL31,SKI,SH3GL2,BMPER,TADA3,TEC,NRP1,TAOK1,CTBP1,XDH,PHB2,INSM1,SIRPA,ARAF,COMMD1,HDAC6,WNK3,PTPRO,MLST8,SVIP,ADORA2A,CDC25A,PPIL4,FBXO33,STAT2,DAG1,MADD,PRKAA2,CDK5R1,C3,RAF1,ZMPSTE24,PINK1
trans-synaptic signaling	GO:0099537	7.93894E-06	712	487	24	ADORA2B,CHRNA7,NLGN4X,CHRNA2,SYP,DGKB,GRM2,CNR2,KCNA1,LPAR3,CPNE6,CHAT,PTCHD1,RAB26,ADORA2A,BAIAP3,TMEM108,GRID2IP,LZTS1,BLOC1S6,PLPPR4,DAG1,RPS6KA3,PINK1
cellular response to organic substance	GO:0071310	8.27212E-06	2385	486	48	NCK2,APOB,FOXA1,TRIM33,ARF6,TMEM204,ITGB8,CXCL6,SKI,SH3GL2,TMEM38A,BMPER,TADA3,SPIDR,SOX5,SYP,NRP1,XDH,PLK5,PHB2,AQP4,ARL6IP5,SIRPA,MARS1,ST18,EPOR,HDAC6,IL36RN,MED1,BAIAP3,ZNF366,NDUFAF2,TMEM108,MYO1C,USP9X,LTK,STAT2,PER1,DAG1,CREB3L1,GBP5,TBXA2R,RARB,PRKAA2,RAF1,CALCRL,TBL2,KRT18
regulation of cellular response to stress	GO:0080135	8.36238E-06	648	494	23	NCK2,KLHL31,SPIDR,TAOK1,XDH,UBQLN2,ARL6IP5,HIC1,SLC25A23,SIRPA,HDAC6,RELL1,ULK4,TXNDC12,SVIP,NSD2,PER1,CREB3L1,RNF8,ZMYND11,ZMPSTE24,PINK1,FAM168A
regulation of phosphorylation	GO:0042325	9.00907E-06	1223	458	31	NCK2,CHRNA7,KLHL31,SH3GL2,BMPER,TADA3,RANBP2,TEC,NRP1,TAOK1,XDH,PHB2,INSM1,SIRPA,ARAF,HDAC6,WNK3,PTPRO,MLST8,BTBD10,ADORA2A,CDC25A,PPIL4,LTK,STAT2,DAG1,MADD,PRKAA2,CDK5R1,C3,RAF1
positive regulation of cellular component organization	GO:0051130	9.23708E-06	1021	487	29	NCK2,ARF6,SPIDR,ADGRB3,NRP1,PLK5,FSCN1,MAD2L1BP,CROCC,SH3GLB1,LPAR3,ANLN,MPHOSPH8,DNM1L,BICD1,MLST8,MPP7,ANO6,FAF1,NPHP4,LTK,DAG1,GBP5,CDK5R1,NEK7,C3,ATF1,NPHP1,PINK1

cell population proliferation	GO:0008283	1.03481E-05	1940	310	32	NCK2,CHRNA7,PRKX,GINS1,SKI,BMPER,MEIS2,SOX5,NRP1,SDCBP2,CTBP1,XDH,PHB2,COPS2,INSM1,EAPP,KCNA1,REG1A,NKX2-8,ST18,WDR6,RBBP4,PCM1,PODN,SCN5A,NELL1,NPR3,STAMBP,BTBD10,ADORA2A,MED1,CDC25A
regulation of cell death	GO:0010941	1.15033E-05	1575	487	37	NCK2,FOXA1,CHRNA7,NRP1,BNIP3L,PLK5,PHB2,ZC3H8,ARL6IP5,ALOX12,ARAF,HDAC6,RNPS1,WNK3,DNM1L,TXNDC12,STAMBP,BTBD10,IP6K2,ANO6,FAF1,ADORA2A,MED1,NAA38,MAL,LTK,CREB3L1,MADD,RARB,PRKAA2,CDK5R1,MNT,ZMYND11,RAF1,RPS6KA3,KRT18,PINK1
regulation of programmed cell death	GO:0043067	1.26371E-05	1439	487	35	NCK2,FOXA1,NRP1,BNIP3L,PLK5,PHB2,ZC3H8,ARL6IP5,ALOX12,ARAF,HDAC6,RNPS1,WNK3,DNM1L,TXNDC12,STAMBP,IP6K2,ANO6,FAF1,ADORA2A,MED1,NAA38,MAL,LTK,CREB3L1,MADD,RARB,PRKAA2,CDK5R1,MNT,ZMYND11,RAF1,RPS6KA3,KRT18,PINK1
cell development	GO:0048468	1.30582E-05	2079	475	43	NCK2,FOXA1,ZPBP,CHRNA7,ARF6,UBA6,SKI,SH3GL2,BFSP1,ADGRB3,NRP1,TAOK1,PLK5,INSM1,LPAR3,NKX2-8,CPNE6,RNF112,NEXN,GPRIN2,HDAC6,PCM1,CELSR2,PTPRO,ULK4,ADORA2A,MED1,NPHP4,TMEM108,LZTS1,USP9X,OGDH,BLOC1S6,LTK,PLPPR4,DAG1,SZT2,RARB,RNF8,CDK5R1,C3,ATF1,ZMPSTE24
animal organ development	GO:0048513	1.60948E-05	3494	489	61	POLR1B,FOXA1,PRKX,NLGN4X,ARF6,UBA6,ITGB8,SKI,BMPER,DRC1,BFSP1,MACROD2,MEIS2,SOX5,RBM24,NRP1,XDH,SLC39A3,ARMC6,PHB2,COPS2,EOMES,ZC3H8,INSM1,KCNA1,CTNS,ALOX12,ANLN,NKX2-8,PRPSAP2,RNF112,EPOR,PTCHD1,PCM1,CELSR2,SCN5A,NELL1,PTPRO,ANO6,MED1,NPHP4,NSD2,HECTD1,PLAC1,VIT,CSDE1,TMEM108,SHROOM2,OGDH,PLPPR4,DAG1,SZT2,RARB,CDK5R1,POLE,RAF1,CALCRL,ZMPSTE24,HPCA,NPHP1,H4C8
vesicle-mediated transport	GO:0016192	1.64808E-05	1597	487	37	ADORA2B,NLGN4X,ARF6,FCHO1,SH3GL2,TVP23B,ICAM3,SYP,LDLRAD3,NRP1,PRAM1,UBQLN2,OSBPL5,SIRPB1,SIRPA,CPNE6,COMMD1,DNM1L,BICD1,RAB26,ANO6,EXOC6B,ADORA2A,BAIAP3,CD320,TMEM108,BLOC1S6,MARCHF2,CHMP3,EHD3,CDK5R1,C3,CALCRL,TRAPPC1,HPCA,KRT18,PINK1
protein catabolic process	GO:0030163	1.67447E-05	985	487	28	APOB,UBA6,USP49,USP24,BNIP3L,NEMF,UBQLN2,ARAF,COMMD1,HDAC6,NELL1,NSFL1C,RNF144A,RAB26,SVIP,UBXN2B,RNF216,FAF1,KLHDC1,HECTD1,FHIT,USP9X,FBXO33,MARCHF2,PSME2,RNF8,ZMPSTE24,PINK1
transmembrane transport	GO:0055085	1.81149E-05	1531	487	36	BEST1,CHRNA7,TMEM38A,SLC44A5,CHRNA2,NDUFS7,SLC38A1,SLC39A3,PHB2,SLC15A3,SLC17A2,TRPC4,AQP4,ARL6IP5,SLC25A23,KCNA1,CTNS,SLC30A2,PIRT,COMMD1,LASP1,SCN5A,KCNS3,WNK3,KCNA4,ANO6,SLC5A10,SHROOM2,SCN8A,ABCA4,SLC25A44,EHD3,C3,ZMPSTE24,HPCA,PINK1

biological process involved in interspecies interaction between organisms	GO:0044419	1.82314E-05	1634	478	37	APOB,ITGB8,CXCL6,SPAG11A,MACROD2,DGKB,NRP1,BNIP3L,PHB2,SLC15A3,CNR2,AQP4,SIRPA,REG1A,C8B,CFP,ANO6,RNF216,IL36RN,THBD,MED1,DEFB129,REG4,C5,MYO1C,TENT5A,MARCHF2,STAT2,CHMP3,LSM5,DAG1,GBP5,TBXA2R,C3,ZMYND11,ZMPSTE24,RPS6KA3
regulation of cellular localization	GO:0060341	1.90956E-05	805	487	25	ADORA2B,ARF6,TMEM38A,SPIDR,CEP72,BNIP3L,CROCC,SH3GLB1,COMMD1,PCM1,SCP2,NSFL1C,WNK3,BICD1,SVIP,UBXN2B,BAIAP3,HECTD1,NDUFAF2,MYO1C,CHMP3,DAG1,CDK5R1,HPCA,PINK1
cell activation	GO:0001775	2.16226E-05	1082	193	18	ADORA2B,HLA-DQA2,NCK2,ITGB8,CXCL6,VWF,FCHO1,BMPER,TEC,DGKB,PRAM1,PHB2,EOMES,CNR2,SIRPB1,ZC3H8,SIRPA,ALOX12
negative regulation of cellular biosynthetic process	GO:0031327	2.22168E-05	1581	454	35	NCK2,FOXA1,TRIM33,SKI,MEIS2,RBM24,CTBP1,PHB2,COPS2,EOMES,ZC3H8,INSM1,HIC1,EAPP,SIRPA,CTNS,GLIS1,HDAC6,MPHOSPH8,RCOR2,TNRC6A,MED1,NSD2,ZNF366,KDM1B,USP9X,ZNF318,PER1,SCML2,CREB3L1,RARB,RNF8,CDK5R1,MNT,ZMYND11
negative regulation of transcription, DNA-templated	GO:0045892	2.23793E-05	1284	454	31	NCK2,FOXA1,TRIM33,SKI,MEIS2,CTBP1,PHB2,COPS2,EOMES,ZC3H8,INSM1,HIC1,EAPP,GLIS1,HDAC6,MPHOSPH8,RCOR2,MED1,NSD2,ZNF366,KDM1B,USP9X,ZNF318,PER1,SCML2,CREB3L1,RARB,RNF8,CDK5R1,MNT,ZMYND11
negative regulation of nucleic acid-templated transcription	GO:1903507	2.31839E-05	1286	454	31	NCK2,FOXA1,TRIM33,SKI,MEIS2,CTBP1,PHB2,COPS2,EOMES,ZC3H8,INSM1,HIC1,EAPP,GLIS1,HDAC6,MPHOSPH8,RCOR2,MED1,NSD2,ZNF366,KDM1B,USP9X,ZNF318,PER1,SCML2,CREB3L1,RARB,RNF8,CDK5R1,MNT,ZMYND11
nitrogen compound transport	GO:0071705	2.33143E-05	2278	492	46	APOB,ARF6,SLC44A5,HOMER3,TVP23B,DHRS1,RANBP2,SLC38A1,BNIP3L,TIMM10,ZDHHC4,PHB2,SLC15A3,TRPC4,GRM2,SH3GLB1,ARL6IP5,SLC25A23,CTNS,UNC50,COMMD1,HDAC6,TANGO2,PCM1,DNM1L,RAB26,SVIP,EXOC6B,ADORA2A,MED1,BAIAP3,CD320,NDUFAF2,MYO1C,BLOC1S6,CHMP3,SLC25A44,EHD3,SMG7,CDK5R1,RAF1,CALCRL,HPCA,KRT18,PINK1,SNX31
negative regulation of RNA biosynthetic process	GO:1902679	2.40156E-05	1288	454	31	NCK2,FOXA1,TRIM33,SKI,MEIS2,CTBP1,PHB2,COPS2,EOMES,ZC3H8,INSM1,HIC1,EAPP,GLIS1,HDAC6,MPHOSPH8,RCOR2,MED1,NSD2,ZNF366,KDM1B,USP9X,ZNF318,PER1,SCML2,CREB3L1,RARB,RNF8,CDK5R1,MNT,ZMYND11
inorganic ion transmembrane transport	GO:0098660	2.46593E-05	850	400	23	BEST1,CHRNA7,TMEM38A,NDUFS7,SLC39A3,PHB2,SLC15A3,SLC17A2,TRPC4,SLC25A23,KCNA1,CTNS,SLC30A2,COMMD1,SCN5A,KCNS3,WNK3,KCNA4,ANO6,SLC5A10,SHROOM2,SCN8A,EHD3

regulation of apoptotic process	GO:0042981	2.61501E-05	1411	487	34	NCK2,FOXA1,NRP1,BNIP3L,PLK5,PHB2,ZC3H8,ARL6IP5,ALOX12,ARAF,RNPS1,WNK3,DNM1L,TXNDC12,STAMBP,IP6K2,ANO6,FAF1,ADORA2A,MED1,NAA38,MAL,LTK,CREB3L1,MADD,RARB,PRKAA2,CDK5R1,MNT,ZMYND11,RAF1,RPS6KA3,KRT18,PINK1
inorganic cation transmembrane transport	GO:0098662	2.75701E-05	785	400	22	CHRNA7,TMEM38A,NDUFS7,SLC39A3,PHB2,SLC15A3,SLC17A2,TRPC4,SLC25A23,KCNA1,CTNS,SLC30A2,COMMD1,SCN5A,KCNS3,WNK3,KCNA4,ANO6,SLC5A10,SHROOM2,SCN8A,EHD3
anterograde trans-synaptic signaling	GO:0098916	2.96026E-05	704	487	23	ADORA2B,CHRNA7,NLGN4X,CHRNA2,SYP,DGKB,GRM2,CNR2,KCNA1,LPAR3,CPNE6,CHAT,PTCHD1,RAB26,ADORA2A,BAIAP3,TMEM108,GRID2IP,LZTS1,BLOC1S6,PLPPR4,RPS6KA3,PINK1
chemical synaptic transmission	GO:0007268	2.96026E-05	704	487	23	ADORA2B,CHRNA7,NLGN4X,CHRNA2,SYP,DGKB,GRM2,CNR2,KCNA1,LPAR3,CPNE6,CHAT,PTCHD1,RAB26,ADORA2A,BAIAP3,TMEM108,GRID2IP,LZTS1,BLOC1S6,PLPPR4,RPS6KA3,PINK1
organonitrogen compound biosynthetic process	GO:1901566	3.02295E-05	1698	490	38	ADORA2B,NCK2,APOB,SMS,SLC44A5,CDA,ADCY4,RBM24,GSPT1,ZDHHC4,CARS1,NEMF,MOCS1,INSM1,PRPSAP2,MARS1,CHAC2,CHAT,TNRC6A,EXTL3,SVIP,ALG5,CSDE1,ALG8,CMPK1,DPY19L1,PER1,CHST12,RPL15,AGXT2,CARNS1,SUCLA2,PLPP2,GALNT14,HPCA,RPS6KA3,PINK1,LGSN
negative regulation of biosynthetic process	GO:0009890	3.4758E-05	1610	454	35	NCK2,FOXA1,TRIM33,SKI,MEIS2,RBM24,CTBP1,PHB2,COPS2,EOMES,ZC3H8,INSM1,HIC1,EAPP,SIRPA,CTNS,GLIS1,HDAC6,MPHOSPH8,RCOR2,TNRC6A,MED1,NSD2,ZNF366,KDM1B,USP9X,ZNF318,PER1,SCML2,CREB3L1,RARB,RNF8,CDK5R1,MNT,ZMYND11
response to biotic stimulus	GO:0009607	3.54601E-05	1529	478	35	APOB,ITGB8,CXCL6,SPAG11A,CDA,MACROD2,DGKB,BNIP3L,PLK5,PHB2,SLC15A3,CNR2,AQP4,SIRPA,REG1A,C8B,CFP,RNF216,IL36RN,THBD,MED1,DEFB129,REG4,C5,MYO1C,TENT5A,MARCHF2,STAT2,LSM5,GBP5,TBXA2R,C3,ZMYND11,ZMPSTE24,RPS6KA3
cell part morphogenesis	GO:0032990	4.05923E-05	657	446	21	CHRNA7,SH3GL2,ADGRB3,NRP1,TAOK1,LPAR3,NKX2-8,CPNE6,NEXN,HDAC6,CELSR2,DNM1L,PTPRO,ADORA2A,TMEM108,LZTS1,USP9X,PLPPR4,DAG1,SZT2,CDK5R1
positive regulation of cellular component biogenesis	GO:0044089	4.06037E-05	493	487	19	NCK2,ARF6,SPIDR,ADGRB3,NRP1,FSCN1,CROCC,SH3GLB1,ANLN,MPHOSPH8,MLST8,MPP7,FAF1,NPHP4,DAG1,GBP5,CDK5R1,NPHP1,PINK1

regulation of developmental process	GO:0050793	4.35831E-05	2421	489	47	APOB,FOXA1,CHRNA7,PRKX,ARF6,ITGB8,SKI,BMPER,MEIS2,SOX5,ADGRB3,RBM24,NRP1,XDH,PHB2,STRIP1,ZC3H8,INSM1,GLIS1,LPAR3,CPNE6,RNF112,HDAC6,PCM1,CELSR2,NELL1,DNM1L,ANO6,MED1,NSD2,LZTS1,C5,BLOC1S6,LTK,STAT2,DAG1,CREB3L1,TBXA2R,RARB,CDK5R1,C3,RAF1,FMNL3,ZMPSTE24,RPS6KA3,PINK1,H4C8
establishment of protein localization	GO:0045184	4.522E-05	1942	492	41	APOB,ARF6,HOMER3,TVP23B,SPIDR,DHRS1,RANBP2,BNIP3L,TIMM10,ZDHHC4,PHB2,SLC15A3,SH3GLB1,ARL6IP5,CTNS,UNC50,COMMD1,HDAC6,TANGO2,PCM1,DNM1L,RAB26,SVIP,EXOC6B,ADORA2A,MED1,BAIAP3,NSD2,NDUFAF2,MYO1C,MAL,BLOC1S6,CHMP3,EHD3,CDK5R1,RAF1,CALCRL,HPCA,KRT18,PINK1,SNX31
response to growth factor	GO:0070848	5.28996E-05	681	131	12	TRIM33,ARF6,TMEM204,ITGB8,SKI,SH3GL2,BMPER,MEIS2,SOX5,NRP1,XDH,PLK5
regulation of cellular catabolic process	GO:0031329	5.47353E-05	850	487	25	RBM24,BNIP3L,PHB2,UBQLN2,SH3GLB1,ARAF,COMMD1,WDR6,HDAC6,TNRC6A,NELL1,DNM1L,RNF144A,SVIP,FAF1,HECTD1,FHIT,LZTS1,TENT5A,MARCHF2,PSME2,PRKAA2,CDK5R1,ZMPSTE24,PINK1
modification-dependent protein catabolic process	GO:0019941	5.70069E-05	671	487	22	UBA6,USP49,USP24,NEMF,UBQLN2,ARAF,COMMD1,HDAC6,NSFL1C,RNF144A,SVIP,UBXN2B,RNF216,FAF1,KLHDC1,HECTD1,FHIT,USP9X,FBXO33,RNF8,ZMPSTE24,PINK1
response to external biotic stimulus	GO:0043207	6.22481E-05	1491	478	34	APOB,ITGB8,CXCL6,SPAG11A,CDA,MACROD2,DGKB,BNIP3L,PHB2,SLC15A3,CNR2,AQP4,SIRPA,REG1A,C8B,CFP,RNF216,IL36RN,THBD,MED1,DEFB129,REG4,C5,MYO1C,TENT5A,MARCHF2,STAT2,LSM5,GBP5,TBXA2R,C3,ZMYND11,ZMPSTE24,RPS6KA3
cation transmembrane transport	GO:0098655	6.54404E-05	897	400	23	CHRNA7,TMEM38A,NDUFS7,SLC39A3,PHB2,SLC15A3,SLC17A2,TRPC4,SLC25A23,KCNA1,CTNS,SLC30A2,PIRT,COMMD1,SCN5A,KCNS3,WNK3,KCNA4,ANO6,SLC5A10,SHROOM2,SCN8A,EHD3
cell migration	GO:0016477	7.09666E-05	1454	469	33	NCK2,PRKX,ARF6,ITGB8,CXCL6,BMPER,NRP1,FSCN1,PHB2,CNR2,INSM1,SIRPA,ANLN,NEXN,HDAC6,PCM1,PODN,CELSR2,DNM1L,PTPRO,ULK4,ANO6,SHROOM2,C5,FRMD5,MYO1C,USP9X,OGDH,DAG1,TBXA2R,CDK5R1,FMNL3,LIMA1
neuron projection morphogenesis	GO:0048812	7.36993E-05	619	446	20	CHRNA7,SH3GL2,ADGRB3,NRP1,TAOK1,LPAR3,NKX2-8,CPNE6,NEXN,HDAC6,CELSR2,PTPRO,ADORA2A,TMEM108,LZTS1,USP9X,PLPPR4,DAG1,SZT2,CDK5R1
modification-dependent macromolecule catabolic process	GO:0043632	7.56994E-05	682	487	22	UBA6,USP49,USP24,NEMF,UBQLN2,ARAF,COMMD1,HDAC6,NSFL1C,RNF144A,SVIP,UBXN2B,RNF216,FAF1,KLHDC1,HECTD1,FHIT,USP9X,FBXO33,RNF8,ZMPSTE24,PINK1

cellular component morphogenesis	GO:0032989	8.2898E-05	749	446	22	ZPBP,CHRNA7,SH3GL2,ADGRB3,NRP1,TAOK1,LPAR3,NKX2-8,CPNE6,NEXN,HDAC6,CELSR2,DNM1L,PTPRO,ADORA2A,TMEM108,LZTS1,USP9X,PLPPR4,DAG1,SZT2,CDK5R1
response to oxygen-containing compound	GO:1901700	8.772E-05	1631	487	36	APOB,FOXA1,CHRNA7,CXCL6,CDA,SPIDR,DSG1,PHB2,GRM2,CNR2,AQP4,SIRPA,REG1A,RNF112,MARS1,HDAC6,IP6K2,IL36RN,THBD,MED1,BAIAP3,NDUFAF2,MYO1C,LTK,STAT2,PER1,DAG1,TBXA2R,EPM2AIP1,PRKAA2,CDK5R1,RAF1,CALCRL,HPCA,RPS6KA3,PINK1
negative regulation of macromolecule biosynthetic process	GO:0010558	9.65846E-05	1523	454	33	NCK2,FOXA1,TRIM33,SKI,MEIS2,RBM24,CTBP1,PHB2,COPS2,EOMES,ZC3H8,INSM1,HIC1,EAPP,GLIS1,HDAC6,MPHOSPH8,RCOR2,TNRC6A,MED1,NSD2,ZNF366,KDM1B,USP9X,ZNF318,PER1,SCML2,CREB3L1,RARB,RNF8,CDK5R1,MNT,ZMYND11
regulation of cellular component biogenesis	GO:0044087	9.7319E-05	940	487	26	NCK2,ARF6,SPIDR,ADGRB3,NRP1,FSCN1,CROCC,UBQLN2,SH3GLB1,INSM1,ANLN,HDAC6,MPHOSPH8,MLST8,MPP7,SVIP,FAF1,NPHP4,MYO1C,DAG1,GBP5,PRKAA2,CDK5R1,RAF1,NPHP1,PINK1
ubiquitin-dependent protein catabolic process	GO:0006511	0.000101219	660	386	19	UBA6,USP49,USP24,NEMF,UBQLN2,ARAF,COMMD1,HDAC6,NSFL1C,RNF144A,SVIP,UBXN2B,RNF216,FAF1,KLHDC1,HECTD1,FHIT,USP9X,FBXO33
transcription by RNA polymerase II	GO:0006366	0.000102812	2634	493	49	NCK2,FOXA1,TRIM33,ZNF286A,SKI,ZNF561,TADA3,ZNF506,ZNF121,MEIS2,SOX5,CTBP1,COPS2,EOMES,ZNF90,ZC3H8,INSM1,HIC1,EAPP,GLIS1,NKX2-8,ST18,SHOX,RCOR2,ZNF721,EBF4,MED1,NSD2,ZNF366,ZNF43,KDM1B,USP9X,PPIL4,ZSCAN29,STAT2,PER1,TCF7L1,CREB3L1,ZNF676,RARB,RNF8,MNT,ZMYND11,RAF1,ATF1,RPS6KA3,CREB5,ZNF143,ZNF320
regulation of protein phosphorylation	GO:0001932	0.00010605	1076	487	28	NCK2,CHRNA7,KLHL31,SH3GL2,BMPER,TADA3,TEC,NRP1,TAOK1,XDH,PHB2,INSM1,SIRPA,ARAF,HDAC6,WNK3,PTPRO,MLST8,ADORA2A,CDC25A,PPIL4,STAT2,DAG1,MADD,CDK5R1,C3,RAF1,PINK1
plasma membrane bounded cell projection morphogenesis	GO:0120039	0.00010854	634	446	20	CHRNA7,SH3GL2,ADGRB3,NRP1,TAOK1,LPAR3,NKX2-8,CPNE6,NEXN,HDAC6,CELSR2,PTPRO,ADORA2A,TMEM108,LZTS1,USP9X,PLPPR4,DAG1,SZT2,CDK5R1
establishment of localization in cell	GO:0051649	0.000114464	2405	492	46	ADORA2B,NLGN4X,ARF6,TMEM38A,HOMER3,DHRS1,RANBP2,SYP,SDCBP2,BNIP3L,TIMM10,ZDHHC4,NEMF,PRAM1,PHB2,SH3GLB1,EML4,HDAC6,PCM1,SCN5A,SCP2,NSFL1C,BICD1,NOP9,SVIP,UBXN2B,EXOC6B,ADORA2A,MED1,BAIAP3,NDUFAF2,TMEM108,SHROOM2,MYO1C,BLOC1S6,CHMP3,EHD3,SMG7,CDK5R1,RAF1,CILK1,ZMPSTE24,TRAPPC1,HPCA,PINK1,SNX31

cell projection morphogenesis	GO:0048858	0.000120107	638	446	20	CHRNA7,SH3GL2,ADGRB3,NRP1,TAOK1,LPAR3,NKX2-8,CPNE6,NEXN,HDAC6,CELSR2,PTPRO,ADORA2A,TMEM108,LZTS1,USP9X,PLPPR4,DAG1,SZT2,CDK5R1
proteolysis involved in cellular protein catabolic process	GO:0051603	0.000135474	766	487	23	UBA6,USP49,USP24,NEMF,UBQLN2,ARAF,COMMD1,HDAC6,NSFL1C,RNF144A,SVIP,UBXN2B,RNF216,FAF1,KLHDC1,HECTD1,FHIT,USP9X,FBXO33,PSME2,RNF8,ZMPSTE24,PINK1
regulation of body fluid levels	GO:0050878	0.000173217	387	306	13	VWF,TEC,DGKB,XDH,AQP4,ALOX12,CELSR2,NPR3,PTPRO,ANO6,THBD,ADORA2A,MED1
regulation of protein localization to centrosome	GO:1904779	0.000180209	10	291	4	CEP72,NSFL1C,BICD1,UBXN2B
response to other organism	GO:0051707	0.00018818	1488	478	33	APOB,ITGB8,CXCL6,SPAG11A,MACROD2,DGKB,BNIP3L,PHB2,SLC15A3,CNR2,AQP4,SIRPA,REG1A,C8B,CFP,RNF216,IL36RN,THBD,MED1,DEFB129,REG4,C5,MYO1C,TENT5A,MARCHF2,STAT2,LSM5,GBP5,TBXA2R,C3,ZMYND11,ZMPSTE24,RPS6KA3
regulation of transcription by RNA polymerase II	GO:0006357	0.000200774	2529	493	47	NCK2,FOXA1,TRIM33,ZNF286A,SKI,ZNF561,TADA3,ZNF506,ZNF121,MEIS2,SOX5,CTBP1,COPS2,EOMES,ZNF90,ZC3H8,INSM1,HIC1,EAPP,GLIS1,NKX2-8,ST18,SHOX,RCOR2,ZNF721,EBF4,MED1,NSD2,ZNF366,ZNF43,KDM1B,USP9X,PPIL4,ZSCAN29,STAT2,PER1,TCF7L1,CREB3L1,ZNF676,RARB,MNT,RAF1,ATF1,RPS6KA3,CREB5,ZNF143,ZNF320
protein transport	GO:0015031	0.000223566	1829	492	38	APOB,ARF6,HOMER3,TVP23B,DHRS1,RANBP2,BNIP3L,TIMM10,ZDHHC4,PHB2,SLC15A3,SH3GLB1,ARL6IP5,CTNS,UNC50,COMMD1,HDAC6,TANGO2,PCM1,DNM1L,RAB26,SVIP,EXOC6B,ADORA2A,MED1,BAIAP3,NDUFAF2,MYO1C,BLOC1S6,CHMP3,EHD3,CDK5R1,RAF1,CALCRL,HPCA,KRT18,PINK1,SNX31
regulation of catabolic process	GO:0009894	0.000259972	989	487	26	RBM24,BNIP3L,PHB2,UBQLN2,SH3GLB1,ARAF,COMMD1,WDR6,HDAC6,TNRC6A,NELL1,DNM1L,RNF144A,RAB26,SVIP,FAF1,HECTD1,FHIT,LZTS1,TENT5A,MARCHF2,PSME2,PRKAA2,CDK5R1,ZMPSTE24,PINK1
head development	GO:0060322	0.000262431	812	477	23	NLGN4X,UBA6,SKI,MACROD2,MEIS2,NRP1,EOMES,KCNA1,CTNS,EPOR,PTCHD1,PCM1,CELSR2,SCN5A,MED1,TMEM108,SHROOM2,OGDH,SZT2,RARB,CDK5R1,RAF1,HPCA
peptidyl-amino acid modification	GO:0018193	0.000272424	1267	487	30	NCK2,TPGS2,PRKX,TADA3,RANBP2,TEC,NRP1,CTBP1,ZDHHC4,ARAF,EEF2KMT,HDAC6,WNK3,MLST8,VCPKMT,NSD2,ALG5,FKBP6,ALG8,DPY19L1,PPIL4,LTK,PER1,PRKAA2,CDK5R1,CSKMT,RAF1,ZMPSTE24,RPS6KA3,PINK1

immune system process	GO:0002376	0.000283591	2748	489	49	ADORA2B,HLA-DQA2,NCK2,PRKX,ITGB8,CXCL6,FCHO1,SPAG11A,MEIS2,TEC,SLC39A3,ARMC6,PRAM1,PHB2,SLC15A3,EOMES,CNR2,SIRPB1,AQP4,ZC3H8,SIRPA,REG1A,C8B,ANLN,FCGR1B,DNM1L,PTPRO,CFP,ANO6,IL36RN,ADORA2A,MED1,NSD2,HECTD1,CD320,C5,MYO1C,BLOC1S6,MARCHF2,STAT2,PSME2,GBP5,RNF8,C3,IGKV6-21,RAF1,ZMPSTE24,RPS6KA3,H4C8
cellular response to growth factor stimulus	GO:0071363	0.000324525	654	131	11	TRIM33,ARF6,TMEM204,ITGB8,SKI,SH3GL2,BMPER,SOX5,NRP1,XDH,PLK5
regulation of ion transport	GO:0043269	0.000330674	682	487	21	BEST1,TMEM38A,HOMER3,PHB2,GRM2,ARL6IP5,KCNA1,PIRT,COMMD1,SCN5A,KCNS3,WNK3,KCNA4,ANO6,ADORA2A,SCN8A,EHD3,PER1,ZMPSTE24,HPCA,PINK1
positive regulation of cellular protein metabolic process	GO:0032270	0.000331719	1361	458	30	NCK2,FANCM,CHRNA7,TEC,NRP1,TAOK1,CTBP1,XDH,PHB2,UBQLN2,ARL6IP5,ARAF,ST18,COMMD1,HDAC6,WNK3,RNF144A,MLST8,SVIP,FAF1,HECTD1,FBXO33,MARCHF2,PSME2,DAG1,MADD,PRKAA2,CDK5R1,C3,RAF1
gene expression	GO:0010467	0.000357715	8909	493	114	POLR1B,ADORA2B,NCK2,APOB,FOXA1,LMF1,CHRNA7,TRIM33,USP49,ZNF286A,ITGB8,CXCL6,SKI,ZNF561,SH3GL2,TOE1,TSEN2,TADA3,HOMER3,ZNF506,ZNF121,MEIS2,SOX5,RBM24,LDLRAD3,GSPT1,CTBP1,CARS1,XDH,NEMF,PHB2,COPS2,RNU6-137P,EOMES,GRM2,AQP4,ZNF90,ZC3H8,INSM1,HIC1,EAPP,SNORA59A,SIRPA,TRMT2A,GLIS1,NKX2-8,MARS1,ST18,COMMD1,WDR6,SHOX,TEFM,HDAC6,MIR99AHG,PERM1,MPHOSPH8,RCOR2,RNPS1,CELSR2,TNRC6A,NELL1,ZNF721,LINC00273,DNM1L,BICD1,EBF4,NOP9,RNF216,IL36RN,FAF1,ADORA2A,MED1,NSD2,ZNF366,SRRM2,FKBP6,CSDE1,ZNF43,KDM1B,C5,USP9X,PPIL4,TENT5A,ZSCAN29,MIR22HG,ZNF318,STAT2,MIR124-1HG,PER1,RPL15,TCF7L1,SCML2,LSM5,SMG7,CREB3L1,GBP5,ZNF676,RARB,RNF8,PRKAA2,CDK5R1,MNT,C3,ZMYND11,CSKMT,RAF1,ATF1,ZMPSTE24,RPS6KA3,CREB5,PINK1,ZNF143,H4C8,ZNF320
brain development	GO:0007420	0.000379102	765	477	22	NLGN4X,UBA6,SKI,MACROD2,MEIS2,NRP1,EOMES,KCNA1,CTNS,EPOR,PTCHD1,PCM1,CELSR2,SCN5A,MED1,TMEM108,SHROOM2,OGDH,SZT2,RARB,CDK5R1,HPCA
organelle assembly	GO:0070925	0.000509652	891	487	24	ZPBP,DRC1,CEP72,FSCN1,CROCC,UBQLN2,SH3GLB1,ANLN,HDAC6,PCM1,CELSR2,NSFL1C,BICD1,GOLGA8B,UBXN2B,DHX30,CSDE1,CHMP3,EHD3,STAG1,PRKAA2,NEK7,CILK1,PINK1

mitotic cell cycle	GO:0000278	0.000512678	895	485	24	FOXA1,GINS1,TADA3,TTC19,MEIS2,TAOK1,GSPT1,PLK5,MAD2L1BP,PHB2,EML4,ANLN,NSFL1C,STAMBP,UBXN2B,CDC25A,LZTS1,CHMP3,PSME2,STAG1,NEK7,POLE,ZMPSTE24,ZWILCH
positive regulation of transport	GO:0051050	0.000519955	892	487	24	ADORA2B,ARF6,KCTD7,EEPD1,SH3GLB1,SIRPB1,SIRPA,KCNA1,PIRT,PCM1,SCN5A,SCP2,WNK3,DNM1L,BICD1,ANO6,ADORA2A,BAIAP3,MYO1C,EHD3,CDK5R1,C3,HPCA,PINK1
cell adhesion	GO:0007155	0.000589132	1480	51	10	HLA-DQA2,HAPLN1,NCK2,FOXA1,PRKX,NLGN4X,ARF6,ITGB8,VWF,FCHO1
biological adhesion	GO:0022610	0.000615033	1487	51	10	HLA-DQA2,HAPLN1,NCK2,FOXA1,PRKX,NLGN4X,ARF6,ITGB8,VWF,FCHO1
nucleic acid metabolic process	GO:0090304	0.000677352	7476	494	99	POLR1B,NCK2,FANCM,FOXA1,TRIM33,GINS1,USP49,ZNF286A,SKI,ZNF561,INO80B,TOE1,SMC6,TSEN2,TADA3,ZNF506,ZNF121,SPIDR,MEIS2,SOX5,RBM24,TAOK1,GSPT1,CTBP1,EEPD1,CARS1,POLN,PHB2,COPS2,RNU6-137P,EOMES,ZNF90,ZC3H8,INSM1,HIC1,EAPP,SNORA59A,TRMT2A,GLIS1,NKX2-8,MARS1,ST18,COMMD1,WDR6,SHOX,TEFM,HDAC6,PERM1,MPHOSPH8,RCOR2,RNPS1,CELSR2,TNRC6A,ZNF721,BICD1,EBF4,NOP9,FAF1,ADORA2A,MED1,NSD2,ZNF366,SRRM2,FKBP6,CSDE1,ZNF43,KDM1B,USP9X,PPIL4,TENT5A,ZSCAN29,ZNF318,STAT2,PER1,TCF7L1,SCML2,LSM5,SMG7,CREB3L1,ZNF676,RARB,RNF8,CDK5R1,NEK7,MNT,ZMYND11,CSKMT,POLE,RAF1,HARBI1,ATF1,ZMPSTE24,RPS6KA3,CREB5,PINK1,ZNF143,H4C8,ZNF320,FAM168A
G protein-coupled receptor signaling pathway	GO:0007186	0.000778846	1245	465	28	ADORA2B,CXCL6,HOMER3,ADCY4,ADGRB3,SYP,DGKB,OR51E1,UBQLN2,GRM2,CNR2,LPAR3,CELSR2,NPR3,OR7E24,BICD1,ADORA2A,BAIAP3,RGS11,OR52I1,GNG7,C5,OR5BS1P,PLPPR4,TBXA2R,CDK5R1,C3,CALCRL
cellular component assembly	GO:0022607	0.000813126	4556	489	68	HLA-DQA2,NCK2,APOB,ZPBP,NLGN4X,ARF6,FCHO1,DRC1,TTC19,SPIDR,DHRS1,ADGRB3,NDUFS7,NRP1,DSG1,CEP72,KCTD7,XDH,FSCN1,RNU6-137P,CROCC,UBQLN2,SH3GLB1,AQP4,INSM1,KCNA1,LPAR3,ANLN,RNF112,RBBP4,HDAC6,MPHOSPH8,PCM1,CELSR2,KCNS3,NSFL1C,KCNA4,DNM1L,BICD1,PTPRO,MLST8,MPP7,GOLGA8B,SVIP,UBXN2B,ANO6,FAF1,DHX30,NPHP4,COX19,NDUFAF2,CSDE1,LGI2,MYO1C,CHMP3,EHD3,DAG1,GBP5,STAG1,PRKAA2,CDK5R1,NEK7,RAF1,LIMA1,CILK1,NPHP1,PINK1,H4C8
cell morphogenesis	GO:0000902	0.00085769	1010	475	25	CHRNA7,SH3GL2,ADGRB3,NRP1,TAOK1,SLC39A3,STRIP1,LPAR3,NKX2-8,CPNE6,NEXN,HDAC6,CELSR2,PTPRO,ADORA2A,MED1,TMEM108,LZTS1,USP9X,PLPPR4,DAG1,SZT2,CDK5R1,FMNL3,ZMPSTE24
protein localization to centrosome	GO:0071539	0.000872218	33	291	5	CEP72,PCM1,NSFL1C,BICD1,UBXN2B

regulation of proteolysis	GO:0030162	0.000951838	728	487	21	LDLRAD3,XDH,UBQLN2,ARL6IP5,ARAF,ST18,COMMD1,HDAC6,RNF144A,SVIP,ADORA2A,A2ML1,HECTD1,FHIT,C5,PSME2,C3,RAF1,ITIH5,RPS6KA3,PINK1
positive regulation of protein-containing complex assembly	GO:0031334	0.000968886	195	487	11	NCK2,ARF6,SPIDR,FSCN1,SH3GLB1,MLST8,MPP7,FAF1,GBP5,CDK5R1,PINK1
circulatory system development	GO:0072359	0.000995283	1089	475	26	APOB,CHRNA7,PRKX,TMEM204,ITGB8,BMPER,DRC1,ADGRB3,RBM24,NRP1,XDH,EOMES,EPOR,SCN5A,MED1,NSD2,HECTD1,C5,DAG1,CREB3L1,TBXA2R,RARB,C3,FMNL3,CALCRL,ZMPSTE24
chemical homeostasis	GO:0048878	0.001013858	1177	469	27	APOB,FOXA1,CHRNA7,TMEM38A,KCTD7,TRPC4,GRM2,AQP4,SLC25A23,KCNA1,LPAR3,ALOX12,SLC30A2,COMMD9,COMMD1,SCN5A,WNK3,HEPH,ADORA2A,BAIAP3,COX19,NDUFAF2,TBXA2R,PRKAA2,RAF1,CTRC,LIMA1
regulation of stress-activated protein kinase signaling cascade	GO:0070302	0.001019263	196	487	11	KLHL31,TAOK1,XDH,ARL6IP5,SIRPA,RELL1,ULK4,PER1,ZMYND11,ZMPSTE24,PINK1
endomembrane system organization	GO:0010256	0.0010998	535	400	16	ZPBP,TMEM38A,SYP,PLK5,SH3GLB1,RNF112,TANGO2,TJAP1,NSFL1C,GOLGA8B,UBXN2B,ANO6,BAIAP3,ATP8B3,CHMP3,EHD3
negative regulation of multicellular organismal process	GO:0051241	0.001142736	996	301	19	ADORA2B,FOXA1,CHRNA7,SKI,HOMER3,ADGRB3,NRP1,PHB2,AQP4,ZC3H8,SIRPA,ALOX12,IP6K1,PCM1,NPR3,PTPRO,RNF216,IL36RN,THBD
cellular component biogenesis	GO:0044085	0.001150858	4801	316	51	POLR1B,HLA-DQA2,NCK2,APOB,ZPBP,NLGN4X,ARF6,FCHO1,DRC1,TTC19,SPIDR,DHRS1,ADGRB3,NDUFS7,NRP1,DSG1,CEP72,KCTD7,XDH,FSCN1,RNU6-137P,CROCC,UBQLN2,SH3GLB1,AQP4,INSM1,KCNA1,LPAR3,ANLN,RNF112,RBBP4,HDAC6,MPHOSPH8,PCM1,CELSR2,KCNS3,NSFL1C,KCNA4,DNM1L,BICD1,PTPRO,NOP9,MLST8,MPP7,GOLGA8B,SVIP,UBXN2B,ANO6,FAF1,DHX30,NPHP4
protein localization to microtubule organizing center	GO:1905508	0.001183828	35	291	5	CEP72,PCM1,NSFL1C,BICD1,UBXN2B
positive regulation of protein metabolic process	GO:0051247	0.001199506	1448	458	30	NCK2,FANCM,CHRNA7,TEC,NRP1,TAOK1,CTBP1,XDH,PHB2,UBQLN2,ARL6IP5,ARAF,ST18,COMMD1,HDAC6,WNK3,RNF144A,MLST8,SVIP,FAF1,HECTD1,FBXO33,MARCHF2,PSME2,DAG1,MADD,PRKAA2,CDK5R1,C3,RAF1

positive regulation of intracellular signal transduction	GO:1902533	0.001200499	971	280	18	ADORA2B,NCK2,CHRNA7,VWF,BMPER,NRP1,TAOK1,XDH,PHB2,SLC15A3,ARL6IP5,HIC1,CTNS,LPAR3,C10ORF71,DNM1L,RELL1,MLST8
positive regulation of signal transduction	GO:0009967	0.001214191	1495	374	27	ADORA2B,NCK2,FOXA1,CHRNA7,SKI,VWF,BMPER,NRP1,TAOK1,XDH,PHB2,SLC15A3,ARL6IP5,HIC1,CTNS,LPAR3,C10ORF71,DNM1L,RELL1,MLST8,FAF1,ADORA2A,MED1,FRMD1,TMEM108,MYO1C,MAL
reproductive process	GO:0022414	0.001217775	1521	484	32	POLR1B,APOB,FANCM,FOXA1,ZPBP,ITGB8,SPAG11A,SPATA6L,SLC38A1,DSG1,PHB2,EOMES,AQP4,EPOR,EFCAB1,THBD,MED1,CDC25A,NPHP4,HECTD1,ATP8B3,PLAC1,FKBP6,CSDE1,KDM1B,TSGA10,USP9X,ZNF318,RNF8,C3,NPHP1,SPATA16
cellular response to endogenous stimulus	GO:0071495	0.001266537	1310	93	12	APOB,FOXA1,TRIM33,ARF6,ITGB8,SKI,SH3GL2,TMEM38A,BMPER,TADA3,SPIDR,SOX5
reproduction	GO:0000003	0.001270055	1524	484	32	POLR1B,APOB,FANCM,FOXA1,ZPBP,ITGB8,SPAG11A,SPATA6L,SLC38A1,DSG1,PHB2,EOMES,AQP4,EPOR,EFCAB1,THBD,MED1,CDC25A,NPHP4,HECTD1,ATP8B3,PLAC1,FKBP6,CSDE1,KDM1B,TSGA10,USP9X,ZNF318,RNF8,C3,NPHP1,SPATA16
sodium ion transport	GO:0006814	0.001582765	246	405	11	SLC38A1,SLC17A2,COMMD9,COMMD1,SCN5A,WNK3,ANO6,SLC5A10,SHROOM2,SCN8A,PER1
stress-activated protein kinase signaling cascade	GO:0031098	0.001588512	249	487	12	ADORA2B,KLHL31,TAOK1,XDH,ARL6IP5,SIRPA,RELL1,ULK4,PER1,ZMYND11,ZMPSTE24,PINK1
positive regulation of nucleobase-containing compound metabolic process	GO:0045935	0.001637122	1897	494	37	ADORA2B,NCK2,FOXA1,SKI,TADA3,SPIDR,MEIS2,RBM24,EOMES,ZC3H8,EAPP,GLIS1,NKX2-8,MARS1,ST18,SHOX,TNRC6A,ZNF721,MED1,NSD2,ZNF43,KDM1B,USP9X,ZNF318,PER1,CREB3L1,RARB,RNF8,PRKAA2,NEK7,RAF1,ATF1,RPS6KA3,CREB5,PINK1,ZNF143,FAM168A
proteasome-mediated ubiquitin-dependent protein catabolic process	GO:0043161	0.001686651	438	386	14	NEMF,UBQLN2,ARAF,COMMD1,NSFL1C,RNF144A,SVIP,UBXN2B,RNF216,FAF1,KLHDC1,HECTD1,FHIT,FBXO33
regulation of protein-containing complex assembly	GO:0043254	0.001823364	402	487	15	NCK2,ARF6,SPIDR,FSCN1,SH3GLB1,INSM1,HDAC6,MLST8,MPP7,SVIP,FAF1,GBP5,CDK5R1,RAF1,PINK1
regulation of vesicle-mediated transport	GO:0060627	0.001960631	527	477	17	ADORA2B,ARF6,SYP,NRP1,PRAM1,UBQLN2,SIRPB1,SIRPA,BICD1,RAB26,ANO6,ADORA2A,BAIAP3,CHMP3,CDK5R1,C3,HPCA

microtubule cytoskeleton organization	GO:0000226	0.001976138	627	447	18	DRC1,TAOK1,CEP72,CROCC,EML4,HDAC6,PCM1,NSFL1C,BICD1,ULK4,GOLGA8B,UBXN2B,CHMP3,DAG1,STAG1,PRKAA2,CDK5R1,NEK7
regulation of cellular protein catabolic process	GO:1903362	0.002466421	260	401	11	UBQLN2,ARAF,COMMD1,NELL1,RNF144A,SVIP,FAF1,HECTD1,FHIT,MARCHF2,PSME2
cellular macromolecule biosynthetic process	GO:0034645	0.002507104	1564	487	32	NCK2,APOB,FANCM,GINS1,RBM24,FAM111B,GSPT1,ZDHHC4,CARS1,POLN,NEMF,MARS1,RBBP4,TNRC6A,EXTL3,SVIP,FAF1,ALG5,CSDE1,ALG8,DPY19L1,PER1,CHST12,RPL15,EPM2AIP1,NEK7,POLE,GALNT14,ATF1,ZMPSTE24,RPS6KA3,PINK1
positive regulation of programmed cell death	GO:0043068	0.002629951	513	446	16	NCK2,FOXA1,BNIP3L,ZC3H8,ARL6IP5,HDAC6,RNPS1,DNM1L,IP6K2,ANO6,FAF1,ADORA2A,MAL,LTK,RARB,CDK5R1
proteasomal protein catabolic process	GO:0010498	0.002632162	503	401	15	NEMF,UBQLN2,ARAF,COMMD1,NSFL1C,RNF144A,SVIP,UBXN2B,RNF216,FAF1,KLHDC1,HECTD1,FHIT,FBXO33,PSME2
response to endogenous stimulus	GO:0009719	0.002666917	1551	445	30	APOB,FOXA1,TRIM33,ARF6,ITGB8,SKI,SH3GL2,TMEM38A,BMPER,TADA3,SPIDR,SOX5,DSG1,PHB2,AQP4,REG1A,MARS1,HDAC6,MED1,ZNF366,TMEM108,MYO1C,USP9X,STAT2,PER1,DAG1,CREB3L1,EPM2AIP1,RARB,PRKAA2
protein localization to organelle	GO:0033365	0.00275935	1105	316	20	ARF6,PDIA2,SPIDR,DHRS1,RANBP2,NRP1,CEP72,BNIP3L,TIMM10,PHB2,CROCC,SH3GLB1,PCM1,C12ORF66,NSFL1C,DNM1L,BICD1,UBXN2B,MED1,NPHP4
cellular response to DNA damage stimulus	GO:0006974	0.002940402	807	133	11	FANCM,UBA6,INO80B,SMC6,SPIDR,MACROD2,RBM24,TAOK1,EEPD1,PLK5,POLN
protein deacetylation	GO:0006476	0.002986739	102	487	8	SKI,TADA3,CTBP1,HDAC6,RCOR2,PER1,PRKAA2,PINK1
apoptotic signaling pathway	GO:0097190	0.0030217	593	487	18	NCK2,NRP1,BNIP3L,ARL6IP5,HIC1,DNM1L,TXNDC12,FAF1,ADORA2A,FHIT,MAL,CREB3L1,MADD,MNT,ZMYND11,RAF1,KRT18,PINK1
system process	GO:0003008	0.003037085	2243	483	40	ADORA2B,BEST1,CHRNA7,NLGN4X,UBA6,SH3GL2,TMEM38A,MEIS2,ADGRB3,CHRNA2,MYH13,OR51E1,SLC38A1,TIMM10,CNR2,AQP4,ARL6IP5,KCNA1,CTNS,PTCHD1,SCN5A,NPR3,OR7E24,DNM1L,PTPRO,TPM4,ADORA2A,OR52I1,TMEM108,SCN8A,OR5BS1P,ABCA4,EHD3,PDE6D,DAG1,TBXA2R,CALCRL,LIMA1,ZMPSTE24,NPHP1
regulation of membrane potential	GO:0042391	0.003037097	419	487	15	CHRNA7,NLGN4X,CHRNA2,KCTD7,CNR2,ARL6IP5,KCNA1,CTNS,SCN5A,ADORA2A,TMEM108,SCN8A,EHD3,ZMPSTE24,PINK1

regulation of ion transmembrane transport	GO:0034765	0.003114273	476	487	16	TMEM38A,PHB2,ARL6IP5,KCNA1,PIRT,COMMD1,SCN5A,KCNS3,WNK3,KCNA4,ANO6,SCN8A,EHD3,ZMPSTE24,HPCA,PINK1
regulation of immune system process	GO:0002682	0.003221831	1423	489	30	ADORA2B,HLA-DQA2,NCK2,CXCL6,FCHO1,MEIS2,TEC,PRAM1,PHB2,SLC15A3,CNR2,SIRPB1,ZC3H8,SIRPA,C8B,FCGR1B,DNM1L,CFP,ANO6,ADORA2A,MED1,NSD2,CD320,C5,BLOC1S6,STAT2,GBP5,C3,RPS6KA3,H4C8
modulation of chemical synaptic transmission	GO:0050804	0.003621822	436	414	14	ADORA2B,CHRNA7,NLGN4X,SYP,DGKB,GRM2,CNR2,RAB26,ADORA2A,BAIAP3,TMEM108,GRID2IP,LZTS1,PLPPR4
MAPK cascade	GO:0000165	0.003669253	793	487	21	ADORA2B,CHRNA7,KLHL31,BMPER,NRP1,TAOK1,XDH,PHB2,ARL6IP5,SIRPA,LPAR3,ARAF,RELL1,ULK4,PER1,DAG1,MADD,ZMYND11,RAF1,RASA4B,PINK1
regulation of trans-synaptic signaling	GO:0099177	0.003719131	437	414	14	ADORA2B,CHRNA7,NLGN4X,SYP,DGKB,GRM2,CNR2,RAB26,ADORA2A,BAIAP3,TMEM108,GRID2IP,LZTS1,PLPPR4
positive regulation of cell communication	GO:0010647	0.003928844	1689	374	28	ADORA2B,NCK2,FOXA1,CHRNA7,SKI,VWF,BMPER,NRP1,TAOK1,XDH,PHB2,SLC15A3,ARL6IP5,HIC1,CTNS,LPAR3,C10ORF71,DNM1L,RELL1,MLST8,FAF1,ADORA2A,MED1,BAIAP3,FRMD1,TMEM108,MYO1C,MAL
negative regulation of protein metabolic process	GO:0051248	0.004011812	1022	478	24	NCK2,CHRNA7,KLHL31,SKI,SH3GL2,RBM24,CTBP1,XDH,INSM1,SIRPA,HDAC6,TNRC6A,NELL1,PTPRO,SVIP,ADORA2A,A2ML1,FHIT,C5,PRKAA2,C3,RAF1,ITIH5,RPS6KA3
positive regulation of signaling	GO:0023056	0.004108587	1693	374	28	ADORA2B,NCK2,FOXA1,CHRNA7,SKI,VWF,BMPER,NRP1,TAOK1,XDH,PHB2,SLC15A3,ARL6IP5,HIC1,CTNS,LPAR3,C10ORF71,DNM1L,RELL1,MLST8,FAF1,ADORA2A,MED1,BAIAP3,FRMD1,TMEM108,MYO1C,MAL
regulation of cell cycle	GO:0051726	0.004282403	1011	485	24	FOXA1,MEIS2,TAOK1,CTBP1,PLK5,MAD2L1BP,PHB2,SH3GLB1,INSM1,ANLN,RNF112,MS4A3,NSFL1C,UBXN2B,MED1,CDC25A,CHMP3,PSME2,MADD,CDK5R1,NEK7,MNT,ZMPSTE24,ZWILCH
organophosphate metabolic process	GO:0019637	0.004366514	938	487	23	ADORA2B,SLC44A5,CDA,ADCY4,DGKB,XDH,PHB2,MOCS1,OSBPL5,IP6K1,PRPSAP2,CHAT,SCP2,IP6K2,FHIT,CMPK1,OGDH,PLPPR4,SUCLA2,PLPP2,PRKAA2,HPCA,PINK1
negative regulation of cellular protein metabolic process	GO:0032269	0.0044577	957	478	23	NCK2,KLHL31,SKI,SH3GL2,RBM24,CTBP1,XDH,INSM1,SIRPA,HDAC6,TNRC6A,NELL1,PTPRO,SVIP,ADORA2A,A2ML1,FHIT,C5,PRKAA2,C3,RAF1,ITIH5,RPS6KA3
mitotic cell cycle process	GO:1903047	0.004820317	744	485	20	GINS1,TTC19,TAOK1,GSPT1,PLK5,MAD2L1BP,PHB2,EML4,ANLN,NSFL1C,STAMBP,UBXN2B,CDC25A,LZTS1,CHMP3,PSME2,STAG1,POLE,ZMPSTE24,ZWILCH

establishment of organelle localization	GO:0051656	0.004896187	409	390	13	ADORA2B,CROCC,EML4,HDAC6,PCM1,NSFL1C,BICD1,NOP9,UBXN2B,EXOC6B,SHROOM2,MYO1C,BLOC1S6
plasma membrane bounded cell projection assembly	GO:0120031	0.005191843	548	293	13	NCK2,ARF6,DRC1,NRP1,FSCN1,CROCC,LPAR3,ANLN,HDAC6,PCM1,CELSR2,PTPRO,ANO6
cell junction organization	GO:0034330	0.005364639	687	483	19	CHRNA7,NLGN4X,ARF6,ADGRB3,DGKB,NRP1,DSG1,FSCN1,HDAC6,PTPRO,MPP7,NPHP4,TMEM108,LGI2,MYO1C,DAG1,CDK5R1,C3,NPHP1
regulation of autophagy	GO:0010506	0.005462708	331	487	13	BNIP3L,PHB2,UBQLN2,SH3GLB1,WDR6,HDAC6,DNM1L,SVIP,LZTS1,PRKAA2,CDK5R1,ZMPSTE24,PINK1
regulation of transmembrane transport	GO:0034762	0.005976613	561	487	17	TMEM38A,PHB2,ARL6IP5,KCNA1,PIRT,COMMD1,SCN5A,KCNS3,WNK3,KCNA4,ANO6,SCN8A,EHD3,C3,ZMPSTE24,HPCA,PINK1
negative regulation of cell population proliferation	GO:0008285	0.006031409	707	306	15	NCK2,SKI,CTBP1,XDH,PHB2,INSM1,NKX2-8,ST18,WDR6,RBBP4,PODN,NELL1,NPR3,ADORA2A,MED1
protein deacylation	GO:0035601	0.006460355	113	487	8	SKI,TADA3,CTBP1,HDAC6,RCOR2,PER1,PRKAA2,PINK1
regulation of stress-activated MAPK cascade	GO:0032872	0.0067911	193	487	10	KLHL31,TAOK1,XDH,ARL6IP5,SIRPA,RELL1,ULK4,PER1,ZMYND11,PINK1
cell projection assembly	GO:0030031	0.006818428	562	293	13	NCK2,ARF6,DRC1,NRP1,FSCN1,CROCC,LPAR3,ANLN,HDAC6,PCM1,CELSR2,PTPRO,ANO6
cellular component disassembly	GO:0022411	0.007030719	449	487	15	NCK2,ARF6,TAOK1,GSPT1,BNIP3L,PLK5,FSCN1,PHB2,HDAC6,DNM1L,CHMP3,C3,LIMA1,ZMPSTE24,PINK1
negative regulation of intracellular signal transduction	GO:1902532	0.007301626	509	487	16	KLHL31,HOMER3,XDH,MAD2L1BP,SIRPA,C12ORF66,TXNDC12,STAMBP,PER1,DAG1,CREB3L1,SZT2,PRKAA2,ZMYND11,RASA4B,PINK1
stress-activated MAPK cascade	GO:0051403	0.007912001	242	487	11	ADORA2B,KLHL31,TAOK1,XDH,ARL6IP5,SIRPA,RELL1,ULK4,PER1,ZMYND11,PINK1
histone deacetylation	GO:0016575	0.008264129	83	487	7	SKI,TADA3,CTBP1,HDAC6,RCOR2,PER1,PINK1
response to abiotic stimulus	GO:0009628	0.008432861	1121	487	25	BEST1,CHRNA7,SPIDR,MEIS2,BNIP3L,PHB2,AQP4,SLC25A23,KCNA1,CTNS,PIRT,WNK3,THBD,CDC25A,NPHP4,ABCA4,PER1,DAG1,RNF8,RAF1,ZMPSTE24,HPCA,TBL2,NPHP1,PINK1
dendrite development	GO:0016358	0.00890699	235	112	6	NCK2,CHRNA7,ARF6,UBA6,ADGRB3,NRP1
macromolecule deacylation	GO:0098732	0.008922532	118	487	8	SKI,TADA3,CTBP1,HDAC6,RCOR2,PER1,PRKAA2,PINK1

tube morphogenesis	GO:0035239	0.009020016	849	142	11	APOB,FOXA1,CHRNA7,PRKX,ITGB8,SKI,BMPER,ADGRB3,NRP1,XDH,PHB2
positive regulation of apoptotic process	GO:0043065	0.009109154	501	446	15	NCK2,FOXA1,BNIP3L,ZC3H8,ARL6IP5,RNPS1,DNM1L,IP6K2,ANO6,FAF1,ADORA2A,MAL,LTK,RARB,CDK5R1
leukocyte activation	GO:0045321	0.009151991	947	180	13	ADORA2B,HLA-DQA2,NCK2,ITGB8,CXCL6,FCHO1,PRAM1,PHB2,EOMES,CNR2,SIRPB1,ZC3H8,SIRPA
regulation of establishment of protein localization	GO:0070201	0.010016728	522	487	16	ARF6,SPIDR,BNIP3L,SH3GLB1,HDAC6,PCM1,DNM1L,SVIP,ADORA2A,BAIAP3,NSD2,NDUFAF2,MYO1C,CDK5R1,HPCA,PINK1
regulation of cell activation	GO:0050865	0.010306475	659	343	15	ADORA2B,HLA-DQA2,NCK2,FCHO1,TEC,PRAM1,CNR2,SIRPB1,ZC3H8,SIRPA,ALOX12,THBD,ADORA2A,NSD2,CD320
macroautophagy	GO:0016236	0.010480949	299	487	12	BNIP3L,PHB2,UBQLN2,SH3GLB1,HDAC6,NSFL1C,UBXN2B,LZTS1,CHMP3,PRKAA2,CDK5R1,PINK1
positive regulation of cell death	GO:0010942	0.010676629	573	446	16	NCK2,FOXA1,BNIP3L,ZC3H8,ARL6IP5,HDAC6,RNPS1,DNM1L,IP6K2,ANO6,FAF1,ADORA2A,MAL,LTK,RARB,CDK5R1
protein localization to microtubule cytoskeleton	GO:0072698	0.010715295	54	291	5	CEP72,PCM1,NSFL1C,BICD1,UBXN2B
complement activation, alternative pathway	GO:0006957	0.011353003	17	452	4	C8B,CFP,C5,C3
secretion	GO:0046903	0.011377064	924	345	18	ADORA2B,ARF6,TVP23B,SYP,XDH,PRAM1,TRPC4,GRM2,TANGO2,CELSR2,NPR3,DNM1L,RAB26,EXOC6B,ADORA2A,MED1,BAIAP3,NDUFAF2
forebrain development	GO:0030900	0.011454252	387	446	13	UBA6,SKI,NRP1,EOMES,KCNA1,PTCHD1,PCM1,SCN5A,TMEM108,OGDH,SZT2,RARB,CDK5R1
regulation of anatomical structure morphogenesis	GO:0022603	0.011820709	926	487	22	CHRNA7,ITGB8,BMPER,ADGRB3,NRP1,PHB2,STRIP1,LPAR3,CPNE6,CELSR2,DNM1L,LZTS1,C5,STAT2,DAG1,CREB3L1,TBXA2R,CDK5R1,C3,FMNL3,ZMPSTE24,PINK1
calcium ion transport	GO:0006816	0.012246034	421	477	14	BEST1,CHRNA7,TMEM38A,HOMER3,TRPC4,SLC25A23,WNK3,DNM1L,ANO6,ADORA2A,EHD3,CALCRL,ZMPSTE24,HPCA
cellular response to external stimulus	GO:0071496	0.012369401	309	479	12	ADORA2B,CDA,MYH13,SH3GLB1,MARS1,TNRC6A,C12ORF66,MED1,DAG1,SZT2,PRKAA2,TBL2

process utilizing autophagic mechanism	GO:0061919	0.012984997	533	487	16	BNIP3L,PHB2,UBQLN2,SH3GLB1,WDR6,HDAC6,NSFL1C,DNM1L,SVIP,UBXN2B,LZTS1,CHMP3,PRKAA2,CDK5R1,ZMPSTE24,PINK1
autophagy	GO:0006914	0.012984997	533	487	16	BNIP3L,PHB2,UBQLN2,SH3GLB1,WDR6,HDAC6,NSFL1C,DNM1L,SVIP,UBXN2B,LZTS1,CHMP3,PRKAA2,CDK5R1,ZMPSTE24,PINK1
regulation of apoptotic signaling pathway	GO:2001233	0.013106628	359	487	13	NCK2,NRP1,DNM1L,TXNDC12,FAF1,ADORA2A,MAL,CREB3L1,MADD,MNT,ZMYND11,RAF1,PINK1
negative regulation of transcription by RNA polymerase II	GO:0000122	0.013229856	936	449	21	NCK2,FOXA1,TRIM33,SKI,MEIS2,CTBP1,COPS2,EOMES,ZC3H8,INSM1,HIC1,GLIS1,MED1,NSD2,ZNF366,KDM1B,USP9X,PER1,CREB3L1,RARB,MNT
response to bacterium	GO:0009617	0.014296302	744	478	19	APOB,CXCL6,SPAG11A,MACROD2,DGKB,CNR2,SIRPA,CFP,IL36RN,THBD,DEFB129,REG4,TENT5A,MARCHF2,LSM5,GBP5,TBXA2R,C3,RPS6KA3
TOR signaling	GO:0031929	0.014497447	126	487	8	CTNS,ARAF,C12ORF66,MLST8,SZT2,PRKAA2,ZMPSTE24,PINK1
Golgi organization	GO:0007030	0.014536467	153	400	8	PLK5,TANGO2,TJAP1,NSFL1C,GOLGA8B,UBXN2B,ATP8B3,EHD3
protein localization to cytoskeleton	GO:0044380	0.0152871	58	291	5	CEP72,PCM1,NSFL1C,BICD1,UBXN2B
regulation of MAPK cascade	GO:0043408	0.015461691	668	487	18	CHRNA7,KLHL31,BMPER,NRP1,TAOK1,XDH,PHB2,ARL6IP5,SIRPA,LPAR3,RELL1,ULK4,PER1,DAG1,MADD,ZMYND11,RAF1,PINK1
regulation of intracellular steroid hormone receptor signaling pathway	GO:0033143	0.0164846	73	405	6	FOXA1,PHB2,HDAC6,MED1,ZNF366,PER1
learning or memory	GO:0007611	0.017484274	263	238	8	CHRNA7,NLGN4X,UBA6,MEIS2,ADGRB3,ARL6IP5,CTNS,PTCHD1
localization within membrane	GO:0051668	0.018229242	883	486	21	ARF6,DHRS1,TIMM10,ZDHHC4,PRAM1,OSBPL5,SH3GLB1,ARL6IP5,COMMD1,SCP2,WNK3,RAB26,EXOC6B,HECTD1,MYO1C,MAL,EHD3,DAG1,CDK5R1,HPCA,KRT18
response to organic cyclic compound	GO:0014070	0.018603243	901	477	21	APOB,FOXA1,TMEM38A,TADA3,CDA,SPIDR,DSG1,PHB2,GRM2,AQP4,ARL6IP5,HDAC6,THBD,ADORA2A,MED1,ZNF366,PER1,DAG1,TBXA2R,ATF1,HPCA
microtubule-based movement	GO:0007018	0.019019108	396	390	12	APOB,DRC1,AQP4,HDAC6,DNHD1,PCM1,CELSR2,EFCAB1,BICD1,NPHP4,TMEM108,BLOC1S6
intracellular estrogen receptor signaling pathway	GO:0030520	0.019136966	55	322	5	FOXA1,TADA3,PHB2,MED1,ZNF366
negative regulation of JNK cascade	GO:0046329	0.019284516	37	487	5	KLHL31,SIRPA,PER1,ZMYND11,PINK1

regulation of cell population proliferation	GO:0042127	0.020184445	1648	488	31	NCK2,CHRNA7,SKI,MEIS2,NRP1,CTBP1,XDH,PHB2,INSM1,EAPP,REG1A,NKX2-8,ST18,WDR6,RBBP4,PODN,SCN5A,NELL1,NPR3,STAMBP,ADORA2A,MED1,CD320,LTK,STAT2,RARB,MNT,RAF1,CALCRL,ZMPSTE24,ZNF143
positive regulation of molecular function	GO:0044093	0.020244599	1491	487	29	ADORA2B,FOXA1,CHRNA7,SKI,RANBP2,NRP1,TAOK1,KCTD7,XDH,PHB2,ARL6IP5,KCNA1,PIRT,ST18,HDAC6,NPR3,WNK3,MLST8,ARHGAP29,LTK,EHD3,PSME2,DAG1,MADD,EPM2AIP1,CDK5R1,NEK7,HPCA,PINK1
organelle localization	GO:0051640	0.021234375	541	390	14	ADORA2B,CROCC,EML4,HDAC6,PCM1,NSFL1C,DNM1L,BICD1,NOP9,UBXN2B,EXOC6B,SHROOM2,MYO1C,BLOC1S6
regulation of cell adhesion	GO:0030155	0.021242619	733	369	16	HLA-DQA2,NCK2,FOXA1,PRKX,FCHO1,NRP1,SIRPB1,ZC3H8,SIRPA,ALOX12,CELSR2,PTPRO,FAF1,ADORA2A,VIT,FRMD5
regulation of organelle organization	GO:0033043	0.021307936	1109	487	24	NCK2,ARF6,NRP1,TAOK1,BNIP3L,FSCN1,MAD2L1BP,CROCC,UBQLN2,SH3GLB1,NEXN,HDAC6,MPHOSPH8,DNM1L,BICD1,MLST8,STAT2,EHD3,PRKAA2,CDK5R1,NEK7,LIMA1,ZWILCH,PINK1
sodium ion transmembrane transport	GO:0035725	0.022354148	173	375	8	SLC17A2,COMMD1,SCN5A,WNK3,ANO6,SLC5A10,SHROOM2,SCN8A
chromosome organization	GO:0051276	0.022415769	1034	489	23	FANCM,FOXA1,USP49,INO80B,SMC6,MAD2L1BP,PHB2,EML4,RBBP4,HDAC6,MPHOSPH8,NSD2,RAD54L2,KDM1B,TCF7L1,STAG1,RNF8,PRKAA2,NEK7,ZMYND11,ZMPSTE24,ZWILCH,H4C8
intracellular steroid hormone receptor signaling pathway	GO:0030518	0.022956993	116	405	7	FOXA1,TADA3,PHB2,HDAC6,MED1,ZNF366,PER1
membrane organization	GO:0061024	0.02313708	825	487	20	APOB,NLGN4X,SYP,BNIP3L,TIMM10,OSBPL5,SH3GLB1,SCP2,NSFL1C,DNM1L,UBXN2B,ANO6,ATP8B3,MAL,ABCA4,BLOC1S6,CHMP3,C3,ZMPSTE24,PINK1
postsynapse organization	GO:0099173	0.023455631	163	112	5	CHRNA7,NLGN4X,ARF6,DGKB,NRP1
anatomical structure formation involved in morphogenesis	GO:0048646	0.023811338	1123	60	8	POLR1B,FOXA1,ZPBP,CHRNA7,PRKX,ITGB8,SKI,BMPER
endocytosis	GO:0006897	0.024204649	638	477	17	NLGN4X,ARF6,FCHO1,SH3GL2,SYP,LDLRAD3,UBQLN2,DNM1L,BICD1,ANO6,CD320,TMEM108,MARCHF2,EHD3,C3,CALCRL,HPCA
vasculature development	GO:0001944	0.024358847	724	465	18	APOB,CHRNA7,PRKX,TMEM204,ITGB8,BMPER,ADGRB3,NRP1,XDH,MED1,HECTD1,C5,DAG1,CREB3L1,TBXA2R,C3,FMNL3,CALCRL

cell-cell adhesion	GO:0098609	0.024420981	888	210	13	HLA-DQA2,NCK2,FOXA1,NLGN4X,FCHO1,ICAM3,DSG1,SIRPB1,AQP4,ZC3H8,SIRPA,ALOX12,NEXN
regulation of proteasomal ubiquitin-dependent protein catabolic process	GO:0032434	0.026370659	138	347	7	UBQLN2,ARAF,COMMD1,RNF144A,SVIP,HECTD1,FHIT
organic anion transport	GO:0015711	0.026582776	361	184	8	BEST1,SLC38A1,SLC17A2,TRPC4,GRM2,ARL6IP5,SLC25A23,CTNS
regulation of response to external stimulus	GO:0032101	0.026609245	927	475	21	ADORA2B,CXCL6,NRP1,CNR2,SIRPA,AOAH,ALOX12,DNM1L,PTPRO,ANO6,RNF216,THBD,ADORA2A,MED1,C5,STAT2,GBP5,TBXA2R,C3,CALCRL,ZMPSTE24
intracellular transport	GO:0046907	0.027913168	1826	492	33	ARF6,HOMER3,DHRS1,RANBP2,SDCBP2,BNIP3L,TIMM10,ZDHHC4,NEMF,PHB2,SH3GLB1,HDAC6,PCM1,SCP2,BICD1,NOP9,SVIP,EXOC6B,MED1,BAIAP3,TMEM108,MYO1C,BLOC1S6,CHMP3,EHD3,SMG7,CDK5R1,CILK1,ZMPSTE24,TRAPPC1,HPCA,PINK1,SNX31
regulation of protein catabolic process	GO:0042176	0.028350695	401	401	12	UBQLN2,ARAF,COMMD1,NELL1,RNF144A,RAB26,SVIP,FAF1,HECTD1,FHIT,MARCHF2,PSME2
adenylate cyclase-modulating G protein-coupled receptor signaling pathway	GO:0007188	0.029510719	239	465	10	ADORA2B,ADCY4,ADGRB3,GRM2,CNR2,LPAR3,NPR3,ADORA2A,TBXA2R,CALCRL
bleb assembly	GO:0032060	0.030676654	11	293	3	LPAR3,ANLN,ANO6
regulation of TOR signaling	GO:0032006	0.0312637	104	475	7	CTNS,ARAF,C12ORF66,MLST8,SZT2,PRKAA2,ZMPSTE24
behavior	GO:0007610	0.031608958	602	316	13	CHRNA7,NLGN4X,UBA6,MEIS2,ADGRB3,GRM2,ARL6IP5,CTNS,PIRT,PTCHD1,PCM1,ADORA2A,NPHP4
RNA metabolic process	GO:0016070	0.032516843	6948	493	87	POLR1B,NCK2,FOXA1,TRIM33,USP49,ZNF286A,SKI,ZNF561,TOE1,TSEN2,TADA3,ZNF506,ZNF121,MEIS2,SOX5,RBM24,GSPT1,CTBP1,CARS1,PHB2,COPS2,RNU6-137P,EOMES,ZNF90,ZC3H8,INSM1,HIC1,EAPP,SNORA59A,TRMT2A,GLIS1,NKX2-8,MARS1,ST18,COMMD1,WDR6,SHOX,TEFM,HDAC6,PERM1,MPHOSPH8,RCOR2,RNPS1,CELSR2,TNRC6A,ZNF721,BICD1,EBF4,NOP9,FAF1,ADORA2A,MED1,NSD2,ZNF366,SRRM2,FKBP6,CSDE1,ZNF43,KDM1B,USP9X,PPIL4,TENT5A,ZSCAN29,ZNF318,STAT2,PER1,TCF7L1,SCML2,LSM5,SMG7,CREB3

						L1,ZNF676,RARB,RNF8,CDK5R1,MNT,ZMYND11,CSKMT,RAF1,ATF1,ZMPSTE24,RPS6KA3,CREB5,PINK1,ZNF143,H4C8,ZNF320
dendritic spine development	GO:0060996	0.032606072	94	33	3	NCK2,ARF6,UBA6
synapse organization	GO:0050808	0.032878856	422	452	13	CHRNA7,NLGN4X,ARF6,ADGRB3,DGKB,NRP1,HDAC6,PTPRO,TMEM108,LGI2,DAG1,CDK5R1,C3
cell morphogenesis involved in neuron differentiation	GO:0048667	0.032962448	559	446	15	CHRNA7,ADGRB3,NRP1,LPAR3,NKX2-8,NEXN,HDAC6,CELSR2,PTPRO,LZTS1,USP9X,PLPPR4,DAG1,SZT2,CDK5R1
telencephalon development	GO:0021537	0.034865522	254	446	10	UBA6,SKI,EOMES,KCNA1,SCN5A,TMEM108,OGDH,SZT2,RARB,CDK5R1
positive regulation of protein localization	GO:1903829	0.03570611	454	487	14	ARF6,CROCC,SH3GLB1,PCM1,WNK3,DNM1L,BICD1,ADORA2A,BAIAP3,MYO1C,PRKAA2,CDK5R1,HPCA,PINK1
blood vessel morphogenesis	GO:0048514	0.036861671	608	465	16	APOB,CHRNA7,PRKX,ITGB8,BMPER,ADGRB3,NRP1,XDH,MED1,C5,DAG1,CREB3L1,TBXA2R,C3,FMNL3,CALCRL
positive regulation of cell-cell adhesion	GO:0022409	0.037140752	284	12	3	HLA-DQA2,NCK2,FOXA1
positive regulation of immune system process	GO:0002684	0.03742146	920	452	20	ADORA2B,HLA-DQA2,NCK2,FCHO1,TEC,PRAM1,PHB2,SIRPB1,SIRPA,C8B,DNM1L,CFP,ANO6,MED1,NSD2,CD320,C5,BLOC1S6,GBP5,C3
tube development	GO:0035295	0.037884152	1047	465	22	APOB,FOXA1,CHRNA7,PRKX,ITGB8,SKI,BMPER,ADGRB3,NRP1,XDH,PHB2,NKX2-8,MED1,HECTD1,C5,DAG1,CREB3L1,TBXA2R,RARB,C3,FMNL3,CALCRL
negative regulation of protein localization to centrosome	GO:1904780	0.039860562	2	291	2	NSFL1C,UBXN2B
negative regulation of BMP signaling pathway	GO:0030514	0.0399118	55	60	3	TRIM33,SKI,BMPER
cellular response to extracellular stimulus	GO:0031668	0.042251879	242	479	10	ADORA2B,MYH13,SH3GLB1,MARS1,TNRC6A,C12ORF66,MED1,SZT2,PRKAA2,TBL2
ion homeostasis	GO:0050801	0.04232412	783	326	15	CHRNA7,TMEM38A,KCTD7,TRPC4,GRM2,SLC25A23,KCNA1,LPAR3,SLC30A2,COMMD1,SCN5A,WNK3,HEPH,ADORA2A,COX19

growth	GO:0040007	0.04238132	951	478	21	NLGN4X,GINS1,SH3GL2,CDA,TEC,NRP1,COPS2,LPAR3,CPNE6,HDAC6,EXTL3,IP6K2,MED1,TMEM108,USP9X,ST7L,DAG1,RARB,CDK5R1,ZMPSTE24,RPS6KA3
adenylate cyclase-activating G protein-coupled receptor signaling pathway	GO:0007189	0.042757289	153	465	8	ADORA2B,ADCY4,ADGRB3,CNR2,LPAR3,ADORA2A,TBXA2R,CALCRL
positive regulation of leukocyte activation	GO:0002696	0.042852009	410	9	3	ADORA2B,HLA-DQA2,NCK2
regulation of proteasomal protein catabolic process	GO:0061136	0.045794202	192	487	9	UBQLN2,ARAF,COMMD1,RNF144A,SVIP,HECTD1,FHIT,PSME2,PINK1
negative regulation of apoptotic process	GO:0043066	0.045902305	866	487	20	NRP1,BNIP3L,PHB2,ALOX12,ARAF,WNK3,TXNDC12,STAMBP,ADORA2A,MED1,NAA38,LTK,CREB3L1,PRKAA2,MNT,ZMYND11,RAF1,RPS6KA3,KRT18,PINK1
positive regulation of cell activation	GO:0050867	0.046365873	421	9	3	ADORA2B,HLA-DQA2,NCK2
regulation of multicellular organismal development	GO:2000026	0.046740808	1315	489	26	CHRNA7,PRKX,ITGB8,SKI,BMPER,MEIS2,SOX5,ADGRB3,NRP1,XDH,ZC3H8,LPAR3,RNF112,PCM1,NELL1,ANO6,MED1,C5,DAG1,CREB3L1,TBXA2R,RARB,CDK5R1,C3,ZMPSTE24,H4C8
defense response	GO:0006952	0.047632079	1687	475	30	ADORA2B,CXCL6,SPAG11A,BNIP3L,PLK5,PHB2,SLC15A3,CNR2,AQP4,SIRPA,AOAH,C8B,CFP,ANO6,RNF216,IL36RN,ADORA2A,MED1,DEFB129,C5,MYO1C,MARCHF2,STAT2,PER1,GBP5,TBXA2R,C3,ZMYND11,CALCRL,ZMPSTE24
positive regulation of phosphorylation	GO:0042327	0.049174396	799	487	19	CHRNA7,RANBP2,TEC,NRP1,TAOK1,PHB2,ARAF,HDAC6,WNK3,MLST8,BTBD10,LTK,DAG1,MADD,PRKAA2,CDK5R1,C3,RAF1,PINK1
blood vessel development	GO:0001568	0.049691571	693	465	17	APOB,CHRNA7,PRKX,ITGB8,BMPER,ADGRB3,NRP1,XDH,MED1,HECTD1,C5,DAG1,CREB3L1,TBXA2R,C3,FMNL3,CALCRL

Table S5: KEGG pathway analysis

KEGG signalling pathways significantly enrichment with upregulated DEGs

source	term_name	term_id	adjusted_p_value	term_size	query_size	intersection_size	intersections
KEGG	KEGG root term	KEGG:00000	3.72E-15	8014	282	91	RSPO4,TECR,OCLN,SLC6A13,COA5,PLEKHM2,BAMBI,KCNQ5,ZFP36L2,OPTN,ACTB,FHL2,HAP1,MEF2C,AGMAT,WNT7A,SLCO1C1,RPL19,ERLEC1,GYG2,SCARB2,ABCC8,ABCB5,STAT5B,PPP1R1A,ITPR2,TEAD4,OR1E1,IGFBP3,PRKAR2A,GRM7,PTGFR,EDA2R,GCK,PDHB,OR8H2,ZNF208,NTN1,TXNDC5,HDAC9,SNX32,UGT2B28,NFATC4,REST,NRG1,GUCY2D,RHOBTB1,MYLK2,HERC2,PEX12,B3GALT6,AQP7,ADRA1D,UBE3A,ARAP2,RRM2,VANGL1,HSD11B1L,MRPL4,ZNF12,MAPK13,ICAM1,CELA2B,BMP2,SLC26A1,HS3ST2,FLNB,DKK4,TAS2R10,ALG1,ECI1,ZNF726,ZNF430,TPSAB1,CLCN4,BAG2,CCNH,PADI4,LPAR2,GMPR,DHFR,NDUFB10,SLC4A4,WIPI2,TAF4B,SLC5A7,GAMT,MUS81,HNRNPA2B1,GNG5,MICALL2
KEGG	Wnt signaling pathway	KEGG:04310	0.002156713	167	213	6	RSPO4,BAMBI,WNT7A,NFATC4,VANGL1,DKK4
KEGG	Oxytocin signaling pathway	KEGG:04921	0.002597233	154	144	5	ACTB,MEF2C,ITPR2,NFATC4,MYLK2
KEGG	cGMP-PKG signaling pathway	KEGG:04022	0.007071489	166	165	5	MEF2C,ITPR2,NFATC4,MYLK2,ADRA1D
KEGG	Cellular senescence	KEGG:04218	0.011009818	156	193	5	ZFP36L2,ITPR2,IGFBP3,NFATC4,MAPK13
KEGG	Amyotrophic lateral sclerosis	KEGG:05014	0.02140012	362	279	8	OPTN,ACTB,HAP1,NRG1,MAPK13,NDUFB10,WIPI2,HNRNPA2B1
KEGG	Leukocyte transendothelial migration	KEGG:04670	0.037843931	114	194	4	OCLN,ACTB,MAPK13,ICAM1
KEGG	Growth hormone synthesis, secretion and action	KEGG:04935	0.043633679	119	193	4	STAT5B,ITPR2,IGFBP3,MAPK13

KEGG signalling pathways significantly enrichment with downregulated DEGs

source	term_name	term_id	adjusted_p_value	term_size	query_size	intersection_size	intersections
KEGG	KEGG root term	KEGG:00000	3.23E-24	8014	493	154	POLR1B,ADORA2B,HLA-DQA2,NCK2,APOB,FANCM,SIKE1,CHRNA7,NLGN4X,SMS,ARF6,UBA6,CCDC6,ZNF286A,ANTXR2,ITGB8,CXCL6,VWF,ZNF561,SH3GL2,SLC44A5,TADA3,HOMER3,ZNF506,CDA,ICAM3,RANBP2,ADCY4,CHRNA2,NDUFS7,TEC,DGKB,OR51E1,SLC38A1,NRP1,TAOK1,DSG1,GSPT1,BNIP3L,CTBP1,CARS1,XDH,SLC39A3,POLN,FSCN1,SDHB,MOCS1,UBQLN2,OSBPL5,TRPC4,GRM2,CNR2,SH3GLB1,SIRPB1,AQP4,ZNF90,PGA5,EML4,SIRPA,CTNS,C8B,LPAR3,ALOX12,IP6K1,ARAF,MARS1,CHAC2,EPOR,RBBP4,HDAC6,CHAT,RNPS1,TJAP1,SCN5A,SCP2,EXTL3,OR7E24,NSFL1C,ZNF721,KCNA4,HEPH,GALK2,DNM1L,CFP,MLST8,TXNDC12,STAMBP,SVIP,IP6K2,TPM4,IL36RN,FAF1,THBD,ADORA2A,MED1,AMY2B,CDC25A,BAIAP3,REG4,NSD2,FRMD1,COX19,ALG5,OR52I1,NDUFAF2,FHIT,ZNF43,ALG8,CMPK1,GNG7,C5,MYO1C,ABCA4,NCEH1,OGDH,STAT2,CHMP3,EHD3,PSME2,PER1,CHST12,RPL15,AGXT2,TCF7L1,LSM5,PDE6D,DAG1,SMG7,CARNS1,CREB3L1,GBP5,TBXA2R,ZNF676,RARB,SUCLA2,STAG1,PLPP2,PRKAA2,CDK5R1,NEK7,C3,POLE,RAF1,RASA4B,CALCRL,GALNT14,ATF1,ZMPSTE24,RPS6KA3,CREB5,KRT18,PINK1,H4C8,ZNF320
KEGG	Thermogenesis	KEGG:04714	0.00128482	232	480	10	ADCY4,NDUFS7,SDHB,MLST8,COX19,NDUFAF2,CREB3L1,PRKAA2,RPS6KA3,CREB5
KEGG	Alcoholism	KEGG:05034	0.001646826	187	489	9	ADORA2B,ARAF,HDAC6,ADORA2A,GNG7,CREB3L1,RAF1,CREB5,H4C8
KEGG	Metabolic pathways	KEGG:01100	0.001703889	1526	443	27	SMS,CDA,ADCY4,NDUFS7,DGKB,XDH,SDHB,MOCS1,ALOX12,MARS1,CHAC2,SCP2,EXTL3,GALK2,TXNDC12,AMY2B,NSD2,ALG5,FHIT,ALG8,CMPK1,OGDH,AGXT2,PDE6D,CARNS1,SUCLA2,PLPP2
KEGG	Cell adhesion molecules	KEGG:04514	0.005092435	146	90	4	HLA-DQA2,NLGN4X,ITGB8,ICAM3
KEGG	Neuroactive ligand-receptor interaction	KEGG:04080	0.00693362	352	465	11	ADORA2B,CHRNA7,CHRNA2,GRM2,CNR2,LPAR3,ADORA2A,C5,TBXA2R,C3,CALCRL
KEGG	Phospholipase D signaling pathway	KEGG:04072	0.007852479	147	191	5	ARF6,ADCY4,DGKB,GRM2,LPAR3
KEGG	Cortisol synthesis and secretion	KEGG:04927	0.011347473	64	480	5	ADCY4,KCNA4,NCEH1,CREB3L1,CREB5

KEGG	Herpes simplex virus 1 infection	KEGG:05168	0.015418016	491	493	13	HLA-DQA2,ZNF286A,ZNF561,ZNF506,ZNF90,ZNF721,CFP,ZNF43,C5,STAT2,ZNF676,C3,ZNF320
KEGG	Non-small cell lung cancer	KEGG:05223	0.015946813	72	458	5	EML4,ARAF,FHIT,RARB,RAF1
KEGG	Glutamatergic synapse	KEGG:04724	0.017631603	114	159	4	HOMER3,ADCY4,SLC38A1,GRM2
KEGG	Cholinergic synapse	KEGG:04725	0.020982481	113	480	6	CHRNA7,ADCY4,CHAT,GNG7,CREB3L1,CREB5
KEGG	Complement and coagulation cascades	KEGG:04610	0.032539168	85	452	5	VWF,C8B,THBD,C5,C3
KEGG	PI3K-Akt signaling pathway	KEGG:04151	0.039835751	353	480	10	ITGB8,VWF,LPAR3,EPOR,MLST8,GNG7,CREB3L1,PRKAA2,RAF1,CREB5
KEGG	Vascular smooth muscle contraction	KEGG:04270	0.0440698	134	465	6	ADORA2B,ADCY4,ARAF,ADORA2A,RAF1,CALCRL
KEGG	Cocaine addiction	KEGG:05030	0.045063537	49	480	4	GRM2,CREB3L1,CDK5R1,CREB5

Table S6: 37 GA-associated genes detected by Eidem et al 2016.

Gnene symbols	Gene names
KLF15	Kruppel-like factor 15
RGAG1	retrotransposon gag domain containing 1
BMP2	bone morphogenetic protein 2
TRIM63	tripartite motif containing 63, E3 ubiquitin protein ligase
ADAMTS15	ADAM metallopeptidase with thrombospondin type 1 motif, 15
SH3PXD2B	SH3 and PX domains 2B
DNASE1L3	deoxyribonuclease I-like 3
TIMP3	TIMP metallopeptidase inhibitor 3
DQX1	DEAQ box RNA-dependent ATPase 1
ELMO3	engulfment and cell motility 3
EFNB1	ephrin-B1
SLC25A4	solute carrier family 25 (mitochondrial carrier; adenine nucleotide translocator), member 4
CLMP	CXADR-like membrane protein
HAND2	hand and neural crest derivatives expressed 2
AMOT	angiomotin
BRPF1	bromodomain and PHD finger containing, 1
FUCA2	fucosidase, alpha-L-2, plasma
DCTN1	dynactin 1
TFDP1	transcription factor Dp-1
RFWD3	ring finger and WD repeat domain 3
MIOS	missing oocyte, meiosis regulator, homolog (Drosophila)
PPIP5K2	diphosphoinositol pentakisphosphate kinase 2
LDB2	LIM domain binding 2
APLNR	apelin receptor

FAM65B	family with sequence similarity 65, member B
CCND1	cyclin D1
KL	klotho
MFSD4	major facilitator superfamily domain containing 4
BAALC	brain and acute leukemia, cytoplasmic
FCN1	ficolin (collagen/fibrinogen domain containing) 1
TMEM56	transmembrane protein 56
ICA1	islet cell autoantigen 1, 69kDa
BMX	BMX non-receptor tyrosine kinase
POF1B	premature ovarian failure, 1B
DBH	dopamine beta-hydroxylase (dopamine beta-monooxygenase)
INSL6	insulin-like 6
APOB	apolipoprotein B

Table S7: KEGG signalling pathway enrichment after removal of GA-associated genes from the DEGs

Signalling pathways enrichment with upregulated DEGs

source	term_name	term_id	adjusted_p_value	term_size	query_size	intersection_size	intersections
KEGG	Oxytocin signaling pathway	KEGG:04921	0.0021162	148	144	5	ACTB,MEF2C,ITPR2,NFATC4,MYLK2
KEGG	cGMP-PKG signaling pathway	KEGG:04022	0.0060336	161	165	5	MEF2C,ITPR2,NFATC4,MYLK2,ADRA1D
KEGG	Wnt signaling pathway	KEGG:04310	0.00864	162	177	5	RSPO4,BAMBI,WNT7A,NFATC4,VANGL1
KEGG	Leukocyte transendothelial migration	KEGG:04670	0.0325879	110	194	4	OCLN,ACTB,MAPK13,ICAM1
KEGG	Growth hormone synthesis, secretion and action	KEGG:04935	0.0353844	113	193	4	STAT5B,ITPR2,IGFBP3,MAPK13
KEGG	Platelet activation	KEGG:04611	0.0443802	120	193	4	ACTB,ITPR2,MYLK2,MAPK13
KEGG	Proteoglycans in cancer	KEGG:05205	0.0458351	200	206	5	ACTB,WNT7A,ITPR2,MAPK13,FLNB

Signalling pathways enrichment with downregulated DEGs

source	term_name	term_id	adjusted_p_value	term_size	query_size	intersection_size	intersections
KEGG	Cell adhesion molecules	KEGG:04514	0.0047412	143	89	4	HLA-DQA2,NLGN4X,ITGB8,ICAM3
KEGG	Phospholipase D signaling pathway	KEGG:04072	0.0059009	139	188	5	ARF6,ADCY4,DGKB,GRM2,LPAR3
KEGG	Alcoholism	KEGG:05034	0.0072676	177	485	8	ARAF,HDAC6,ADORA2A,GNG7,CREB3L1,RAF1, CREB5,H4C8
KEGG	Glutamatergic synapse	KEGG:04724	0.0145527	109	156	4	HOMER3,ADCY4,SLC38A1,GRM2
KEGG	Non-small cell lung cancer	KEGG:05223	0.0151126	71	454	5	EML4,ARAF,FHIT,RARB,RAF1
KEGG	Cholinergic synapse	KEGG:04725	0.0165464	108	476	6	CHRNA7,ADCY4,CHAT,GNG7,CREB3L1,CREB5
KEGG	Thermogenesis	KEGG:04714	0.0187228	207	476	8	ADCY4,NDUFS7,SDHB,MLST8,CREB3L1,PRKAA 2,RPS6KA3,CREB5
KEGG	Neuroactive ligand-receptor interaction	KEGG:04080	0.020815	336	461	10	CHRNA7,CHRNA2,GRM2,CNR2,LPAR3,ADORA 2A,C5,TBXA2R,C3,CALCRL
KEGG	Complement and coagulation cascades	KEGG:04610	0.0295079	83	448	5	VWF,C8B,THBD,C5,C3
KEGG	PI3K-Akt signaling pathway	KEGG:04151	0.0307619	342	476	10	ITGB8,VWF,LPAR3,EPOR,MLST8,GNG7,CREB3L 1,PRKAA2,RAF1,CREB5
KEGG	Herpes simplex virus 1 infection	KEGG:05168	0.0412903	477	489	12	HLA-DQA2, ZNF561, ZNF506, ZNF90, ZNF721, CFP,ZNF43,C5,STAT2,ZNF676,C3,ZNF320
KEGG	Cocaine addiction	KEGG:05030	0.042545	48	476	4	GRM2,CREB3L1,CDK5R1,CREB5
KEGG	Staphylococcus aureus infection	KEGG:05150	0.0434372	84	482	5	HLA-DQA2,DSG1,C5,C3,KRT18

Table S8: Significantly detected DEGs in Male and Female preterm placentas.

Significant DEGs in Male preterm placentas compared to Male term placentas (FDR < 0.05)

ENSEMBL	SYMBOL	logFC	F	FDR
ENSG00000126218	F10	6.027587958	25.70167188	0.009818209
ENSG00000154654	NCAM2	5.377392114	15.46242621	0.04120425
ENSG00000113889	KNG1	5.202789577	15.87760587	0.038388483
ENSG00000228980	LINC01205	4.973071999	19.27883894	0.021540274
ENSG00000138755	CXCL9	4.86522219	15.51814404	0.036998047
ENSG00000163492	CCDC141	4.848182528	17.49169136	0.029331439
ENSG00000125144	MT1G	4.770631216	21.31891691	0.016410942
ENSG00000091704	CPA1	4.625063243	16.89483058	0.032117288
ENSG00000205364	MT1M	4.532663956	30.925658	0.00520962
ENSG00000260549	MT1L	4.526809366	28.50250967	0.006782645
ENSG00000225526	MKRN2OS	4.47742322	16.39315282	0.034522293
ENSG00000185482	STAC3	4.473379915	19.40799639	0.019380243
ENSG00000202111	VTRNA1-2	4.466120751	16.79106037	0.029868825
ENSG00000243056	EIF4EBP3	4.407417084	17.8948334	0.027643536
ENSG00000158315	RHBDL2	4.396451857	16.72466983	0.032593074
ENSG00000143839	REN	4.162099971	15.40264713	0.037990636
ENSG00000100453	GZMB	4.106647985	14.73717086	0.042655835
ENSG00000199990	VTRNA1-1	4.020223234	19.88823802	0.01923776
ENSG00000169245	CXCL10	3.842269212	14.74640895	0.042655835
ENSG00000170667	RASA4B	3.841761171	14.24138803	0.049517264
ENSG00000163827	LRRC2	3.814342927	14.37591639	0.045605285
ENSG00000167618	LAIR2	3.785161153	14.28768056	0.045930606
ENSG00000073754	CD5L	3.699672526	28.70066098	0.006782645
ENSG00000213977	TAX1BP3	3.668054661	19.46019856	0.01923776

ENSG00000244020	MT1HL1	3.63845012	21.43180831	0.016408944
ENSG00000170412	GPRC5C	3.593770115	15.3936222	0.037990636
ENSG00000100362	PVALB	3.535745744	15.8352546	0.034522293
ENSG00000223797	ENTPD3-AS1	3.522283068	15.10994281	0.040506557
ENSG00000197721	CR1L	3.499578799	14.12320358	0.046325431
ENSG00000197119	SLC25A29	3.492952474	13.9509197	0.04771177
ENSG00000100319	ZMAT5	3.480697365	19.51304207	0.01923776
ENSG00000109072	VTN	3.450078491	25.20570491	0.009818209
ENSG00000155465	SLC7A7	3.447772917	21.59271142	0.015991585
ENSG00000106809	OGN	3.445209876	15.04445102	0.040798993
ENSG00000260260	SNHG19	3.438257057	15.03847349	0.040798993
NA	LINC02315	3.408380223	14.36730303	0.045605285
ENSG00000165682	CLEC1B	3.278623398	16.00229594	0.033510002
ENSG00000079689	SCGN	3.247692449	15.68486196	0.03563082
ENSG00000162444	RBP7	3.220224706	23.93648735	0.010250796
ENSG00000099256	PRTFDC1	3.213713903	14.04228018	0.0467162
ENSG00000007312	CD79B	3.18364762	14.06319729	0.046562823
ENSG00000145850	TIMD4	3.17708206	20.13802908	0.018672308
ENSG00000117090	SLAMF1	3.158605282	15.98697873	0.033545329
ENSG00000177700	POLR2L	3.152374772	27.16324337	0.008813043
ENSG00000143819	EPHX1	3.144213554	16.37854416	0.032117288
ENSG00000163507	CIP2A	3.081273889	18.73577762	0.021540274
ENSG00000125148	MT2A	3.069572951	37.94073237	0.002967634
ENSG00000163737	PF4	3.051561626	23.88561716	0.010250796
ENSG00000108107	RPL28	3.01393538	31.42027242	0.00520962
ENSG00000108622	ICAM2	2.97692529	19.03575467	0.020374352
ENSG00000106245	BUD31	2.96960239	28.02138814	0.007171924
ENSG00000213215	OR2F1	2.956773218	14.47353417	0.044966516
ENSG00000148677	ANKRD1	2.889895218	18.15832664	0.023201505

ENSG00000148335	NTMT1	2.886340993	16.17661925	0.032579468
ENSG00000145708	CRHBP	2.8776441	13.9589205	0.047706029
ENSG00000067365	METTL22	2.864781323	15.0408895	0.040798993
ENSG00000167613	LAIR1	2.852927906	19.47536295	0.01923776
ENSG00000005075	POLR2J	2.793054139	32.40050498	0.00520962
ENSG00000111678	C12orf57	2.762605287	26.07958173	0.009084809
ENSG00000077348	EXOSC5	2.736804078	14.93816761	0.04120425
ENSG00000125744	RTN2	2.724202094	16.32736258	0.032117288
ENSG00000166741	NNMT	2.707101345	38.12852418	0.002967634
ENSG00000177646	ACAD9	2.700328552	15.07682189	0.040798993
ENSG00000110063	DCPS	2.676790934	21.33548967	0.016410942
ENSG00000267645	POLR2J2	2.638757551	33.27748945	0.00520962
ENSG00000185420	SMYD3	2.636450206	19.65954964	0.01923776
ENSG00000245910	SNHG6	2.610820514	23.26560885	0.011235459
ENSG00000174886	NDUFA11	2.589510819	16.17845707	0.032579468
ENSG00000118785	SPP1	2.583567911	23.36434922	0.011235459
ENSG00000101473	ACOT8	2.561371062	15.8438285	0.034522293
ENSG00000177674	AGTRAP	2.555886166	17.73903716	0.025102995
ENSG00000214783	POLR2J4	2.551414852	28.42094891	0.006782645
ENSG00000196381	ZNF781	2.538041824	18.72888519	0.021540274
ENSG00000104894	CD37	2.51057987	14.55214547	0.044550496
ENSG00000163736	PPBP	2.496166448	24.17452677	0.009819527
ENSG00000100526	CDKN3	2.486001434	21.07999681	0.016578188
ENSG00000160948	VPS28	2.468040735	21.63903705	0.015991585
ENSG00000114023	FAM162A	2.45559034	31.23911984	0.00520962
ENSG00000100865	CINP	2.442530732	20.03520118	0.018897179
ENSG00000125995	ROMO1	2.441019371	22.50925375	0.012702001
ENSG00000164253	WDR41	2.440686923	16.2051574	0.032579468
ENSG00000175792	RUVBL1	2.43570301	19.72333457	0.01923776

ENSG00000123892	RAB38	2.414963942	17.45149809	0.02637687
ENSG00000106333	PCOLCE	2.412483601	16.71673628	0.030020509
ENSG00000008517	IL32	2.393032356	16.71829074	0.030020509
NA	SNAR-A13	2.386393596	16.01651031	0.033510002
ENSG00000100097	LGALS1	2.375405172	30.7259118	0.00520962
ENSG00000179085	DPM3	2.374175009	14.1660672	0.045930606
ENSG00000105258	POLR2I	2.370071878	14.69566057	0.043198069
ENSG00000109906	ZBTB16	2.36970425	19.56977101	0.01923776
ENSG00000169715	MT1E	2.368508332	25.82098528	0.009084809
ENSG00000150782	IL18	2.353352537	16.653091	0.030276765
NA	SNAR-A6	2.347427256	15.00754956	0.040976455
ENSG00000241644	INMT	2.316727785	18.53264483	0.021540274
ENSG00000011600	TYROBP	2.311446627	18.56296322	0.021540274
ENSG00000168961	LGALS9	2.303403669	15.59083733	0.036290782
NA	SNAR-A7	2.300970873	16.35828881	0.032117288
ENSG00000134202	GSTM3	2.292225826	21.25082058	0.016410942
ENSG00000117543	DPH5	2.283870333	24.16830852	0.009819527
ENSG00000104872	PIH1D1	2.282387172	25.80443104	0.009084809
ENSG00000063177	RPL18	2.27910017	16.12143388	0.032758123
NA	SNAR-A5	2.277957513	14.01575548	0.047014359
ENSG00000100142	POLR2F	2.277288688	23.14890027	0.01141973
ENSG00000125611	CHCHD5	2.268952725	20.59234177	0.01815745
NA	SNAR-A10	2.263270634	14.63059375	0.043944644
ENSG00000244038	DDOST	2.255761441	20.68624642	0.018087314
ENSG00000145681	HAPLN1	2.255179619	18.01228679	0.023944676
NA	SNAR-A14	2.251273121	15.63386678	0.036013073
NA	SNAR-A8	2.245781969	14.05832735	0.046562823
ENSG00000106268	NUDT1	2.237317698	21.41775877	0.016408944
NA	SNAR-A3	2.231861038	14.79989794	0.042230345

NA	SNAR-A12	2.226650777	14.01017166	0.047014359
ENSG00000079150	FKBP7	2.225733279	14.29019206	0.045930606
NA	SNAR-A11	2.217530727	14.20553131	0.045930606
ENSG00000163106	HPGDS	2.211747482	22.01509841	0.014328579
NA	SNAR-A4	2.20500322	13.93396366	0.047889888
NA	SNAR-A1	2.195069945	14.41083094	0.045169052
ENSG00000237765	FAM200B	2.186747613	17.72571572	0.025102995
ENSG00000172757	CFL1	2.180324454	16.4293923	0.031851742
ENSG00000105379	ETFB	2.177858808	26.77042934	0.008877352
ENSG00000196177	ACADSB	2.17704284	15.46682425	0.037576737
ENSG00000103066	PLA2G15	2.176467946	14.86594546	0.041643805
ENSG00000119599	DCAF4	2.154184465	26.79897701	0.008877352
ENSG00000100297	MCM5	2.151272871	22.4757792	0.012702001
ENSG00000169689	CENPX	2.149909226	19.1713065	0.019816836
ENSG00000130255	RPL36	2.149356807	25.853482	0.009084809
ENSG00000029639	TFB1M	2.147604255	18.7568622	0.021540274
ENSG00000155659	VSIG4	2.144132755	16.75608465	0.029917601
ENSG00000158234	FAIM	2.143937343	15.12240494	0.040506557
ENSG00000205544	TMEM256	2.140747241	21.06230759	0.016578188
ENSG00000141504	SAT2	2.140518421	14.25863316	0.045930606
ENSG00000237289	CKMT1B	2.130957366	19.28591906	0.019691844
ENSG00000102007	PLP2	2.128928684	15.03156385	0.040798993
ENSG00000211445	GPX3	2.108234025	19.59674725	0.01923776
ENSG00000213523	SRA1	2.106253252	22.35136928	0.01300486
ENSG00000068079	IFI35	2.098354327	19.16322833	0.019816836
ENSG00000149131	SERPING1	2.097223849	19.53302625	0.01923776
ENSG00000060762	MPC1	2.090466803	20.54709846	0.01815745
ENSG00000163017	ACTG2	2.076988979	14.60526797	0.043944644
ENSG00000264230	ANXA8L1	2.075106988	24.52595895	0.009818209

ENSG00000109911	ELP4	2.064486794	14.8680135	0.041643805
ENSG00000083845	RPS5	2.062072866	28.77701655	0.006782645
ENSG00000162511	LAPTM5	2.059170388	18.52368088	0.021540274
ENSG00000064490	RFXANK	2.05213104	13.86370669	0.048810756
ENSG00000123297	TSFM	2.051790707	20.17249929	0.018672308
ENSG00000165704	HPRT1	2.04963137	16.92775401	0.029042167
ENSG00000176046	NUPR1	2.029688513	22.54442886	0.012702001
ENSG00000213465	ARL2	2.023019574	15.12739859	0.040506557
ENSG00000188257	PLA2G2A	2.019065572	19.80575074	0.01923776
ENSG00000156587	UBE2L6	2.014137715	23.7126009	0.010597067
ENSG00000163001	CFAP36	2.009562257	16.2593959	0.032238421
ENSG00000170291	ELP5	1.994564208	17.57289725	0.025975521
ENSG00000132386	SERPINF1	1.99049753	24.53890517	0.009818209
ENSG00000265190	ANXA8	1.990330444	23.08348354	0.011427023
ENSG00000146066	HIGD2A	1.9807652	24.41831482	0.009818209
ENSG00000173171	MTX1	1.972231094	17.12524745	0.028380419
NA	TP53TG1	1.968323399	16.02417121	0.033510002
ENSG00000166681	BEX3	1.967918048	18.48821036	0.021540274
ENSG00000128789	PSMG2	1.958917851	19.83848394	0.01923776
ENSG00000197406	DIO3	1.957860476	19.58343515	0.01923776
ENSG00000239672	NME1	1.950607607	25.38143454	0.009818209
ENSG00000101464	PIGU	1.944489859	14.31648724	0.045930606
ENSG00000137198	GMPR	1.941038175	14.0030545	0.047014359
ENSG00000164081	TEX264	1.940512169	18.82518157	0.021408434
ENSG00000169627	BOLA2B	1.935488522	22.96509269	0.011647202
ENSG00000197345	MRPL21	1.935009346	24.46055926	0.009818209
ENSG00000142089	IFITM3	1.924281951	24.71186087	0.009818209
ENSG00000019582	CD74	1.920170101	14.81529533	0.042156469
ENSG00000131055	COX4I2	1.916817717	19.31134823	0.019691844

ENSG00000125821	DTD1	1.90100916	15.21121832	0.039756936
ENSG00000100365	NCF4	1.895298987	15.72986944	0.035338058
ENSG00000107796	ACTA2	1.886134972	14.96811002	0.04120425
ENSG00000184047	DIABLO	1.880674538	16.7563934	0.029917601
ENSG00000100417	PMM1	1.877009454	15.22054942	0.039756936
ENSG00000173369	C1QB	1.873820776	20.26347087	0.018552904
ENSG00000135698	MPHOSPH6	1.852729624	20.50511054	0.01815745
ENSG00000119650	IFT43	1.851318057	19.38431544	0.019380243
ENSG00000149591	TAGLN	1.850756264	16.98944539	0.028945528
ENSG00000104325	DECR1	1.845933851	16.26261779	0.032238421
ENSG00000137720	C11orf1	1.845439255	17.66561198	0.025357428
ENSG00000168255	POLR2J3	1.8403075	20.51497726	0.01815745
ENSG00000048162	NOP16	1.8291415	16.52266524	0.031248288
ENSG00000151465	CDC123	1.819344032	25.17526822	0.009818209
ENSG00000154719	MRPL39	1.818566741	19.52896539	0.01923776
ENSG00000079277	MKNK1	1.808672496	17.48154066	0.026255957
ENSG00000148671	ADIRF	1.806872662	18.84317628	0.021408434
ENSG00000105220	GPI	1.802728837	21.19773159	0.016410942
ENSG00000150779	TIMM8B	1.78772094	17.10231215	0.028380419
ENSG00000162493	PDPN	1.784177261	18.09561825	0.023390949
ENSG00000158869	FCER1G	1.778339959	16.97258314	0.028945528
ENSG00000166347	CYB5A	1.777071773	20.47456419	0.01815745
ENSG00000006625	GGCT	1.774025904	24.38213688	0.009818209
ENSG00000105193	RPS16	1.772038334	20.7747961	0.018062661
ENSG00000166803	PCLAF	1.770790462	15.82628909	0.034522293
ENSG00000110934	BIN2	1.758389435	14.22979181	0.045930606
ENSG00000183765	CHEK2	1.756605381	14.53193142	0.044636818
ENSG00000124802	EEF1E1	1.752347932	14.10511705	0.046325431
ENSG00000274070	CASTOR2	1.7514352	19.05054077	0.020374352

ENSG00000175768	TOMM5	1.751186703	26.31884547	0.009084809
ENSG00000205629	LCMT1	1.749323345	24.33142382	0.009818209
ENSG00000157379	DHRS1	1.74845703	14.66554322	0.043551747
ENSG00000183011	NAA38	1.735269581	20.0582587	0.018897179
ENSG00000110077	MS4A6A	1.72729503	16.16555066	0.032579468
ENSG00000198805	PNP	1.723666768	23.46304556	0.011235459
ENSG00000093010	COMT	1.708684126	14.43009361	0.045169052
ENSG00000166171	DPCD	1.706995602	14.15617639	0.045930606
ENSG00000102265	TIMP1	1.706831753	14.93749054	0.04120425
ENSG00000126768	TIMM17B	1.704485187	14.87068691	0.041643805
ENSG00000177575	CD163	1.704078294	15.92876043	0.033977867
ENSG00000090266	NDUFB2	1.697994562	22.56973459	0.012702001
ENSG00000151500	THYN1	1.697082466	17.92782255	0.024345326
ENSG00000163344	PMVK	1.696395523	16.94832065	0.028990916
ENSG00000175324	LSM1	1.695464729	18.59500358	0.021540274
ENSG00000149925	ALDOA	1.691386528	14.18509425	0.045930606
NA	SNAR-E	1.689098505	14.09983255	0.046325431
ENSG00000103415	HMOX2	1.682984196	18.51560952	0.021540274
ENSG00000265354	TIMM23	1.681292652	15.54933437	0.036715604
ENSG00000137474	MYO7A	1.675621146	14.45198696	0.045036961
ENSG00000178980	SELENOW	1.663199327	17.248787	0.027643536
ENSG00000186197	EDARADD	1.653395813	21.18052116	0.016410942
ENSG00000135838	NPL	1.651786315	16.88735958	0.029313643
ENSG00000168899	VAMP5	1.651030266	18.46495713	0.021540274
ENSG00000161281	COX7A1	1.650395518	14.8471174	0.041791814
ENSG00000241553	ARPC4	1.645308666	18.47300619	0.021540274
ENSG00000120306	CYSTM1	1.623069154	20.34818141	0.018483098
ENSG00000160049	DFFA	1.622155712	14.23520026	0.045930606
ENSG00000263327	TAPT1-AS1	1.619801799	18.35840676	0.021886034

ENSG00000103363	ELOB	1.619208523	17.77450569	0.025102995
ENSG00000166451	CENPN	1.6152966	16.99467887	0.028945528
ENSG00000100442	FKBP3	1.613623778	16.81050939	0.029823926
ENSG00000100575	TIMM9	1.607699112	17.73497682	0.025102995
ENSG00000120885	CLU	1.607459086	17.23953262	0.027643536
ENSG00000183844	FAM3B	1.604140141	15.88772669	0.03435539
ENSG00000149806	FAU	1.591744474	18.12807423	0.023285816
ENSG00000134291	TMEM106C	1.591508919	16.69656082	0.030079427
ENSG00000167815	PRDX2	1.590320087	19.58904319	0.01923776
ENSG00000236552	RPL13AP5	1.588991268	16.37686669	0.032117288
ENSG00000130830	MPP1	1.588634952	17.56433563	0.025975521
ENSG00000100823	APEX1	1.586757742	16.58385489	0.030698307
ENSG00000158042	MRPL17	1.58183128	17.10937552	0.028380419
ENSG00000090263	MRPS33	1.577915889	19.93306067	0.01923776
ENSG00000239900	ADSL	1.577543125	20.42193365	0.018249203
ENSG00000155366	RHOC	1.573900348	18.39274835	0.021886034
ENSG00000136522	MRPL47	1.565670834	19.62482525	0.01923776
ENSG00000164258	NDUFS4	1.565144389	19.85303884	0.01923776
ENSG00000075914	EXOSC7	1.562833149	20.73165602	0.018065633
ENSG00000243749	TMEM35B	1.56083778	15.74186938	0.035337202
ENSG00000138175	ARL3	1.552533392	14.3414641	0.04591675
ENSG00000173418	NAA20	1.551186153	18.61348986	0.021540274
ENSG00000170791	CHCHD7	1.549218017	17.84941476	0.024882561
ENSG00000125445	MRPS7	1.546172941	16.23325338	0.032395873
ENSG00000017427	IGF1	1.5401999	14.62371962	0.043944644
ENSG00000167900	TK1	1.539449337	14.4090494	0.045169052
ENSG00000175183	CSRP2	1.534369053	16.49169559	0.031445532
ENSG00000164172	MOCS2	1.533864739	14.6137528	0.043944644
ENSG00000109861	CTSC	1.533012734	15.43405656	0.037725127

ENSG00000175110	MRPS22	1.531077812	14.3137891	0.045930606
ENSG00000084207	GSTP1	1.517029821	14.24184736	0.045930606
ENSG00000171858	RPS21	1.516417729	17.52458648	0.026010215
ENSG00000152413	HOMER1	1.512535188	18.35810655	0.021886034
ENSG00000143353	LYPLAL1	1.50817369	17.11084043	0.028380419
ENSG00000154518	ATP5MC3	1.496710549	16.96846338	0.028945528
ENSG00000169189	NSMCE1	1.495643658	17.52887658	0.026010215
ENSG00000167515	TRAPPC2L	1.492017959	15.71564235	0.035370184
ENSG00000155368	DBI	1.489577811	15.2668935	0.03923368
ENSG00000242485	MRPL20	1.484195043	19.25823428	0.019693266
ENSG00000100348	TXN2	1.481879502	14.89182008	0.041643805
ENSG00000112304	ACOT13	1.481672007	15.44983351	0.037659347
ENSG00000124172	ATP5F1E	1.481160177	15.67290888	0.035633475
ENSG00000177556	ATOX1	1.474565495	18.52393767	0.021540274
ENSG00000177410	ZFAS1	1.469200149	16.00597332	0.033510002
ENSG00000160752	FDPS	1.46597615	15.31223802	0.038781822
ENSG00000125375	DMAC2L	1.463711311	15.19221017	0.039884611
ENSG00000162244	RPL29	1.459358347	14.78176514	0.042230345
ENSG00000137486	ARRB1	1.452733892	15.93563597	0.033977867
ENSG00000180353	HCLS1	1.450423771	17.71125827	0.025102995
ENSG00000112576	CCND3	1.446573175	17.31087096	0.027576063
ENSG00000091164	TXNL1	1.434157343	14.18621957	0.045930606
ENSG00000169567	HINT1	1.42854628	14.78915309	0.042230345
ENSG00000183336	BOLA2	1.417661554	14.57652231	0.044284413
ENSG00000175061	SNHG29	1.414945904	14.16910725	0.045930606
ENSG00000117448	AKR1A1	1.412321876	16.64959854	0.030276765
ENSG00000124570	SERPINB6	1.411912897	17.29775042	0.027576063
ENSG00000140307	GTF2A2	1.410932347	16.31721561	0.032117288
ENSG00000104687	GSR	1.409343611	14.06805037	0.046562823

ENSG00000129235	TXNDC17	1.385573325	16.44931907	0.031783201
ENSG00000139343	SNRPF	1.379332569	14.52191993	0.044636818
ENSG00000137288	UQCC2	1.376134224	15.02889647	0.040798993
ENSG00000138772	ANXA3	1.374530817	13.89916517	0.048267037
ENSG00000228474	OST4	1.371571916	14.46490471	0.044966516
ENSG00000100211	CBY1	1.370483341	16.27491279	0.032238421
ENSG00000141552	ANAPC11	1.367689913	14.47897441	0.044966516
ENSG00000084623	EIF3I	1.355740693	15.30753051	0.038781822
ENSG00000170899	GSTA4	1.344002979	15.76844095	0.035138563
ENSG00000115685	PPP1R7	1.341672501	14.20672278	0.045930606
ENSG00000266472	MRPS21	1.335737896	14.93261136	0.04120425
ENSG00000149100	EIF3M	1.323686293	14.95170623	0.04120425
ENSG00000233954	UQCRHL	1.314494289	14.4122614	0.045169052
ENSG00000159199	ATP5MC1	1.307426569	15.11536597	0.040506557
ENSG00000244184	LOC101559451	1.305363971	14.25827091	0.045930606
ENSG00000164707	SLC13A4	1.291598007	13.81442352	0.049517264
ENSG00000126432	PRDX5	1.288178962	14.09476945	0.046325431
ENSG00000164405	UQCRQ	1.271659907	13.92171723	0.04797839
ENSG00000102243	VGLL1	1.27150328	14.30268358	0.045930606
ENSG00000188243	COMMD6	1.269618754	14.15795857	0.045930606
ENSG00000166136	NDUFB8	1.263231103	14.51058435	0.044636818
ENSG00000082438	COBLL1	-1.306858198	13.78641946	0.049896701
ENSG00000110723	EXPH5	-1.32000663	15.61877708	0.03606125
ENSG00000112144	CILK1	-1.41536201	14.17116084	0.045930606
ENSG00000178202	POGLUT3	-1.48064593	16.62624074	0.030376124
ENSG00000154822	PLCL2	-1.482231129	17.73132712	0.025102995
ENSG00000254087	LYN	-1.601009353	17.04795652	0.028787931
ENSG00000122691	TWIST1	-1.636875978	16.30994954	0.032117288
ENSG00000108389	MTMR4	-1.674635971	18.67607359	0.021540274

ENSG00000154429	CCSAP	-1.677681383	16.11232219	0.032758123
ENSG00000162998	FRZB	-1.742753881	14.11114965	0.046325431
ENSG00000134247	PTGFRN	-1.784891137	20.30766701	0.018504159
ENSG00000139514	SLC7A1	-1.803487297	18.27080006	0.022428491
ENSG00000253138	LINC00967	-1.815493915	17.9834613	0.024023734
ENSG00000122641	INHBA	-1.819826199	14.25535281	0.045930606
ENSG00000197329	PELI1	-1.914529714	14.32459643	0.045930606
ENSG00000003989	SLC7A2	-1.949762644	23.26205064	0.011235459
ENSG00000152463	OLAH	-2.006877995	19.23257644	0.019693266
ENSG00000147606	SLC26A7	-2.020978708	18.93144757	0.020969741
ENSG00000164627	KIF6	-2.498981706	14.51108603	0.044636818
ENSG00000197176	LINC02291	-2.694219214	24.93957561	0.009818209
ENSG00000175445	LPL	-2.821425535	29.04683761	0.006782645
ENSG00000153093	ACOXL	-2.834178111	14.19453635	0.045930606
ENSG00000147571	CRH	-2.855973551	25.95870127	0.009084809
ENSG00000185499	MUC1	-3.061143046	32.06778797	0.00520962
ENSG00000140465	CYP1A1	-5.529873483	20.18664192	0.018672308

Significant DEGs in Female preterm placentas compared to Female term placentas (FDR < 0.05)

**ENSEMBL**	**SYMBOL**	**logFC**	**F**	**FDR**
ENSG00000091428	RAPGEF4	6.017810676	19.1783132	0.011315985
ENSG00000132321	IQCA1	5.817852235	20.66130899	0.008419134
ENSG00000137821	LRRC49	5.627597482	18.33280556	0.012774909
ENSG00000106541	AGR2	5.571008527	18.29763589	0.012862276
ENSG00000166863	TAC3	5.470248134	90.75126951	3.75E-10
ENSG00000177335	C8orf31	5.1692117	16.53520756	0.020455261
ENSG00000148346	LCN2	5.088954004	15.76829878	0.023837879

ENSG00000281103	TRG-AS1	4.950137863	13.09592894	0.043479242
ENSG00000100206	DMC1	4.85478865	13.35951805	0.040983518
ENSG00000204382	XAGE1B	4.523439418	18.87776136	0.011518638
ENSG00000204379	XAGE1A	4.481605479	18.49018135	0.012487383
ENSG00000137860	SLC28A2	4.241693519	19.58534338	0.010152785
ENSG00000165092	ALDH1A1	4.101338495	29.66707666	0.001296771
NA	SNAR-H	4.081253508	12.82565572	0.046125345
ENSG00000105499	PLA2G4C	3.932819531	13.05539305	0.043127791
ENSG00000012223	LTF	3.928269709	33.67876009	0.000402192
ENSG00000133048	CHI3L1	3.888477202	18.37097874	0.012581856
ENSG00000134802	SLC43A3	3.760815851	21.11415863	0.006949707
NA	LINC01116	3.719765834	13.23839847	0.041328629
ENSG00000162639	HENMT1	3.679015459	25.54439684	0.003051738
ENSG00000149292	TTC12	3.647261304	24.36896642	0.003949627
ENSG00000147041	SYTL5	3.624106589	13.44861414	0.039522879
ENSG00000118271	TTR	3.512073074	16.31214565	0.020851953
ENSG00000143156	NME7	3.472260406	34.27659717	0.000402192
ENSG00000284770	TBCE	3.352922189	35.85710141	0.000266769
ENSG00000066185	ZMYND12	3.276150876	13.01301776	0.043479242
ENSG00000105989	WNT2	3.198773201	46.07414215	3.12E-05
ENSG00000143819	EPHX1	3.178056302	26.73528296	0.002302518
ENSG00000199990	VTRNA1-1	3.15750268	14.99298877	0.027944898
ENSG00000117091	CD48	3.144248809	13.14769342	0.042064063
ENSG00000075213	SEMA3A	3.048037789	15.55961943	0.024337717
ENSG00000163803	PLB1	3.033096385	19.900965	0.009620894
ENSG00000115590	IL1R2	3.004120513	27.26825963	0.002266802
ENSG00000173210	ABLIM3	2.941675225	20.33966329	0.008554739
ENSG00000175356	SCUBE2	2.935045558	14.90746485	0.028209599
ENSG00000135218	CD36	2.922469008	40.54913413	9.43E-05

ENSG00000169495	HTRA4	2.849316321	26.96399813	0.002273019
ENSG00000123352	SPATS2	2.831315298	13.87492022	0.036173818
ENSG00000134460	IL2RA	2.817294383	12.81296163	0.046125345
ENSG00000165819	METTL3	2.691183246	15.15622082	0.027234384
ENSG00000186652	PRG2	2.689320719	14.3332299	0.031852959
ENSG00000164253	WDR41	2.666233746	21.28841518	0.006754552
ENSG00000095970	TREM2	2.648724787	13.07804049	0.042926848
ENSG00000116791	CRYZ	2.631661388	19.36637552	0.010601477
ENSG00000140105	WARS1	2.630467747	37.04850333	2.47E-04
ENSG00000137509	PRCP	2.621653311	42.34921191	6.93E-05
ENSG00000164494	PDSS2	2.588148999	19.7731932	0.009680142
ENSG00000175287	PHYHD1	2.580051327	17.24366373	0.016338235
ENSG00000205403	CFI	2.569222507	17.85194646	0.013714408
ENSG00000166741	NNMT	2.558502903	31.14921125	0.000910085
ENSG00000121289	CEP89	2.542732169	19.22814524	0.010922758
ENSG00000136250	AOAH	2.522331922	15.78539123	0.023413994
ENSG00000113272	THG1L	2.485735183	21.7536331	0.00620309
ENSG00000101310	SEC23B	2.470070395	36.01115586	0.000266769
ENSG00000211448	DIO2	2.46481536	15.44758728	0.024978346
ENSG00000150995	ITPR1	2.453829763	14.18241258	0.03370048
ENSG00000143194	MAEL	2.453767248	15.3187204	0.025822399
ENSG00000115840	SLC25A12	2.447301841	16.07345581	0.021667593
ENSG00000237289	CKMT1B	2.43709709	19.25080205	0.010922758
ENSG00000188257	PLA2G2A	2.43178554	33.97334082	0.000402192
ENSG00000164116	GUCY1A1	2.430616388	16.55835045	0.019645241
ENSG00000095485	CWF19L1	2.425500369	18.31153409	0.012581856
ENSG00000128581	IFT22	2.422381188	15.77544663	0.023413994
ENSG00000165704	HPRT1	2.414478361	13.10284371	0.042662209
ENSG00000145088	EAF2	2.407109323	13.62004839	0.038347664

ENSG00000048740	CELF2	2.403351284	13.99606784	0.034732922
ENSG00000188677	PARVB	2.390743526	14.63358801	0.030085058
ENSG00000166974	MAPRE2	2.364189294	13.82558675	0.036364502
ENSG00000108622	ICAM2	2.348864069	13.02521212	0.043479242
ENSG00000143493	INTS7	2.346878231	14.73222119	0.029381067
ENSG00000046647	GEMIN8	2.346707904	13.30314103	0.040831954
ENSG00000118785	SPP1	2.31094477	19.80153236	0.009680142
ENSG00000271605	MILR1	2.308742476	13.86602922	0.036195119
ENSG00000103051	COG4	2.286195471	27.23386029	0.002266802
ENSG00000121858	TNFSF10	2.263915543	30.0231792	0.001261504
ENSG00000108691	CCL2	2.263868773	18.24062962	0.01265408
ENSG00000164815	ORC5	2.259725342	17.33979495	0.015993024
ENSG00000166387	PPFIBP2	2.252387578	13.54674248	0.038706197
ENSG00000115271	GCA	2.25002774	21.31332449	0.006754552
ENSG00000127472	PLA2G5	2.242637626	22.41321038	0.005370374
ENSG00000131203	IDO1	2.233776653	27.93810981	0.002061214
ENSG00000250722	SELENOP	2.232934646	29.53247335	0.001296771
ENSG00000132423	COQ3	2.231371283	14.0963473	0.034151631
ENSG00000049167	ERCC8	2.21300794	12.94457291	0.044363495
ENSG00000119725	ZNF410	2.211726003	24.20422182	0.003973079
ENSG00000163221	S100A12	2.210911048	14.43699287	0.031101026
ENSG00000233901	LINC01503	2.193259224	14.70170475	0.029541553
ENSG00000163017	ACTG2	2.184908076	23.44920965	0.004461915
ENSG00000182093	GET1	2.178147693	20.47952931	0.008419134
ENSG00000196628	TCF4	2.177671327	27.50524417	0.002266802
ENSG00000149131	SERPING1	2.172742516	24.23397754	0.003973079
ENSG00000136319	TTC5	2.16821859	15.40870958	0.025127262
ENSG00000108591	DRG2	2.166729292	19.47433522	0.010352795
ENSG00000269893	SNHG8	2.157255016	21.69680781	0.00620309

ENSG00000143374	TARS2	2.152498466	13.49810087	0.039189552
ENSG00000134779	TPGS2	2.152400957	14.95150584	0.028028117
ENSG00000197548	ATG7	2.151855634	25.10547156	0.003369574
ENSG00000136518	ACTL6A	2.141149904	23.60917823	0.004397065
ENSG00000104299	INTS9	2.136940099	17.73610528	0.013896305
ENSG00000189050	RNFT1	2.13411679	14.90256533	0.028209599
ENSG00000136960	ENPP2	2.13051213	25.26250843	0.003283036
ENSG00000138785	INTS12	2.122155995	15.57949576	0.024337717
ENSG00000261371	PECAM1	2.120589322	24.86280197	0.003465091
ENSG00000187049	TMEM216	2.111747084	14.55320937	0.030459709
ENSG00000138801	PAPSS1	2.110744592	16.33184425	0.020829652
ENSG00000149591	TAGLN	2.104890968	23.91387405	0.004103067
ENSG00000198919	DZIP3	2.10309615	14.19761615	0.03359469
ENSG00000143248	RGS5	2.100689256	13.54504589	0.038706197
ENSG00000159596	TMEM69	2.100662921	18.94189568	0.011430726
ENSG00000185215	TNFAIP2	2.093468186	27.07151483	0.002273019
ENSG00000159251	ACTC1	2.091778381	13.85809049	0.036201371
ENSG00000184047	DIABLO	2.088707311	21.1489813	0.006949707
ENSG00000135838	NPL	2.088572963	21.60828126	0.006326025
ENSG00000139437	TCHP	2.085460997	13.20625333	0.041346743
ENSG00000108039	XPNPEP1	2.082873351	22.6782268	0.005200704
NA	SNAR-A6	2.079170412	26.69219664	0.002302518
ENSG00000134057	CCNB1	2.078899777	25.9196006	0.00296945
ENSG00000187778	MCRS1	2.065765811	15.74061502	0.023528763
ENSG00000197635	DPP4	2.059678002	25.69688689	0.003051738
ENSG00000164808	SPIDR	2.054914405	19.57583508	0.010152785
ENSG00000011465	DCN	2.054225104	20.57604458	0.008418178
ENSG00000134202	GSTM3	2.051958328	15.65772471	0.023837879
ENSG00000143320	CRABP2	2.035988084	15.09182561	0.027595506

ENSG00000108448	TRIM16L	2.031545514	17.79438953	0.013779314
ENSG00000132965	ALOX5AP	2.026106235	24.50277958	0.003864686
ENSG00000100865	CINP	2.018466792	16.22147465	0.020925968
ENSG00000146282	RARS2	2.012789068	19.69622011	0.009879459
NA	TP53TG1	2.001161889	14.87375194	0.028284206
ENSG00000053438	NNAT	1.998156006	16.08680219	0.021667593
ENSG00000100220	RTCB	1.985210136	24.07765773	0.00406009
ENSG00000166803	PCLAF	1.985037886	22.57334642	0.005293939
ENSG00000137033	IL33	1.982463657	20.28809936	0.008626393
NA	SNAR-A8	1.981952777	23.48819041	0.004461915
ENSG00000121691	CAT	1.981290537	22.53408115	0.005293939
NA	SNAR-A14	1.979672622	23.09893773	0.004884455
ENSG00000113140	SPARC	1.978875384	17.22067885	0.016389244
ENSG00000091732	ZC3HC1	1.978060757	16.57536526	0.019645241
ENSG00000160948	VPS28	1.97659798	21.93312347	0.005976984
ENSG00000198062	POTEH	1.975779804	16.1145984	0.021633835
ENSG00000267107	PCAT19	1.97290439	21.46516908	0.006597983
ENSG00000144048	DUSP11	1.972564102	18.41709456	0.012581856
ENSG00000197406	DIO3	1.970027728	16.98533941	0.017207854
NA	SNAR-A9	1.96988102	22.99322967	0.004884455
ENSG00000237064	EIF3IP1	1.968343527	21.99774388	0.005976984
ENSG00000107796	ACTA2	1.966853713	18.21190307	0.012707134
ENSG00000115368	WDR75	1.961956468	14.71874489	0.029437646
ENSG00000106603	COA1	1.961825724	20.65026413	0.008282224
ENSG00000119203	CPSF3	1.959563686	22.7050335	0.005200704
ENSG00000164330	EBF1	1.958504159	13.76049924	0.037195581
ENSG00000120656	TAF12	1.952199411	21.91321348	0.005976984
ENSG00000124491	F13A1	1.949946354	22.37049771	0.005370374
ENSG00000196834	POTEI	1.946479676	14.07360106	0.034322521

ENSG00000155659	VSIG4	1.942534208	19.16829295	0.010922758
ENSG00000143546	S100A8	1.938209355	15.04075173	0.027944898
ENSG00000060718	COL11A1	1.93520448	13.17926636	0.041583844
ENSG00000175899	A2M	1.934111625	23.00372174	0.004884455
ENSG00000057608	GDI2	1.928804436	18.11979339	0.012905173
NA	SNAR-A1	1.924941917	21.70076009	0.00620309
ENSG00000222036	POTEM	1.924576546	16.98951772	0.017207854
ENSG00000140740	UQCRC2	1.924385028	23.95639635	0.004103067
NA	SNAR-A2	1.920228075	22.3560431	0.005370374
ENSG00000177058	SLC38A9	1.920179158	23.65493629	0.004397065
NA	SNAR-A7	1.919345243	21.96626896	0.005976984
ENSG00000116857	TMEM9	1.918848076	19.5240342	0.010258644
ENSG00000163359	COL6A3	1.918173475	18.64947134	0.012101772
ENSG00000129226	CD68	1.912681463	19.44928705	0.010352795
ENSG00000185305	ARL15	1.909652882	15.82143292	0.023413994
ENSG00000165457	FOLR2	1.907448886	21.2782461	0.006754552
ENSG00000189058	APOD	1.905553413	15.78782237	0.023413994
ENSG00000152359	POC5	1.900446607	14.47398417	0.031027408
ENSG00000213949	ITGA1	1.89643629	15.7751301	0.023413994
ENSG00000115956	PLEK	1.891584469	13.91656936	0.035747272
ENSG00000128789	PSMG2	1.88622619	24.879904	0.003465091
ENSG00000132541	RIDA	1.880337136	15.69377098	0.023774646
ENSG00000079257	LXN	1.878376848	14.81201695	0.02870961
ENSG00000180353	HCLS1	1.877417521	20.44414505	0.008419134
ENSG00000111144	LTA4H	1.876810484	12.90353996	0.04507753
ENSG00000163001	CFAP36	1.875238705	20.44241952	0.008419134
ENSG00000151445	VIPAS39	1.874274102	13.83185382	0.036364502
NA	SNAR-A3	1.872198276	20.1469199	0.008805018
ENSG00000281000	SNORD3D	1.872086477	17.95735579	0.013210989

ENSG00000222038	POTEJ	1.869626114	13.53603442	0.038706197
ENSG00000139278	GLIPR1	1.867805483	13.72083725	0.037490948
ENSG00000197345	MRPL21	1.864979814	23.15563534	0.004884455
ENSG00000101670	LIPG	1.863690333	16.53506075	0.019645241
ENSG00000112936	C7	1.863112759	21.25618613	0.006754552
ENSG00000188219	POTEE	1.850949909	13.70114269	0.037577032
ENSG00000064490	RFXANK	1.84977318	15.76122827	0.023436564
ENSG00000137944	KYAT3	1.849771636	20.36554537	0.008554739
ENSG00000140092	FBLN5	1.845664105	13.35969523	0.040157453
ENSG00000185684	EP400P1	1.839350631	16.2025714	0.020936832
ENSG00000186141	POLR3C	1.839088831	14.56489006	0.030459709
ENSG00000105379	ETFB	1.833813429	22.87994543	0.005001715
ENSG00000148700	ADD3	1.832787159	18.31722597	0.012581856
ENSG00000196187	TMEM63A	1.831047554	12.95650557	0.044363495
NA	SNAR-A10	1.830369252	19.8736624	0.009620894
NA	SNAR-A11	1.828137723	19.15395415	0.010922758
ENSG00000105220	GPI	1.825913217	19.78718988	0.009680142
ENSG00000103544	VPS35L	1.822253549	18.69992922	0.011956352
ENSG00000136933	RABEPK	1.81853631	14.00260294	0.034732922
ENSG00000156831	NSMCE2	1.817406427	20.1862592	0.008771492
ENSG00000249751	ECSCR	1.81558241	19.14510712	0.010922758
ENSG00000102024	PLS3	1.806577635	16.92062076	0.017581704
ENSG00000221926	TRIM16	1.803416122	15.00233373	0.027944898
ENSG00000123737	EXOSC9	1.800389927	17.35445509	0.015993024
ENSG00000138036	DYNC2LI1	1.799824932	16.58011302	0.019645241
ENSG00000113407	TARS1	1.799606872	18.5969	0.01220942
ENSG00000276168	RN7SL1	1.791446512	16.40064104	0.020455261
ENSG00000163565	IFI16	1.790534599	18.31100653	0.012581856
ENSG00000274012	RN7SL2	1.786598707	16.29421377	0.020851953

NA	SNAR-A5	1.783094374	18.04248874	0.013017131
ENSG00000187164	SHTN1	1.782320127	14.87541797	0.028284206
ENSG00000113734	BNIP1	1.781694909	16.05621706	0.021713485
ENSG00000001497	LAS1L	1.779277158	15.55897117	0.024337717
ENSG00000181458	TMEM45A	1.771655439	18.27316105	0.012581856
ENSG00000131373	HACL1	1.769139046	14.35639427	0.031731186
ENSG00000125148	MT2A	1.769045294	18.75428133	0.011823753
NA	SNAR-A4	1.767288771	18.4068771	0.012581856
ENSG00000175854	SWI5	1.766324979	16.21651412	0.020925968
ENSG00000106565	TMEM176B	1.762682131	18.53477308	0.012363341
ENSG00000166333	ILK	1.756259769	17.53344254	0.014938411
ENSG00000142657	PGD	1.752095067	13.06828541	0.042996316
ENSG00000075945	KIFAP3	1.750638194	16.28953416	0.020851953
ENSG00000138413	IDH1	1.746813087	20.20837351	0.008771492
NA	FCGR2C	1.739082713	18.8328021	0.011627268
ENSG00000075188	NUP37	1.734818705	15.70288948	0.023774646
ENSG00000144741	SLC25A26	1.729472129	13.3879216	0.040157453
ENSG00000154188	ANGPT1	1.72777916	14.57819363	0.030459709
ENSG00000109016	DHRS7B	1.727068274	18.30512329	0.012581856
ENSG00000245910	SNHG6	1.714493289	12.83738335	0.04608051
ENSG00000144895	EIF2A	1.710955057	17.9998866	0.0130715
ENSG00000143226	FCGR2A	1.710602829	19.0982992	0.011027286
NA	SNAR-A13	1.70794737	13.04416399	0.043227324
ENSG00000223572	CKMT1A	1.68909239	14.13080427	0.033991628
ENSG00000134716	CYP2J2	1.68387743	14.14084997	0.033961276
ENSG00000110077	MS4A6A	1.68280425	14.5823942	0.030459709
ENSG00000188313	PLSCR1	1.670170387	17.08770288	0.016897845
ENSG00000112759	SLC29A1	1.661949555	16.08415264	0.021667593
ENSG00000145287	PLAC8	1.661886207	13.54631029	0.038706197

ENSG00000094914	AAAS	1.661069948	15.36810629	0.025385017
ENSG00000196655	TRAPPC4	1.660614036	17.60842267	0.014569955
ENSG00000168542	COL3A1	1.658183283	15.00852149	0.027944898
ENSG00000117419	ERI3	1.654334454	16.00293273	0.022104596
ENSG00000169715	MT1E	1.653691052	14.44778341	0.031069076
ENSG00000101052	IFT52	1.652959453	12.94964533	0.044363495
ENSG00000138663	COPS4	1.64824264	18.37125691	0.012581856
ENSG00000038002	AGA	1.647376818	14.78839694	0.028774977
ENSG00000013275	PSMC4	1.645864405	18.5856201	0.01220942
ENSG00000174156	GSTA3	1.640959258	19.17968258	0.010922758
ENSG00000135899	SP110	1.640938875	17.25364827	0.016338235
ENSG00000198189	HSD17B11	1.640835619	17.74036391	0.013896305
ENSG00000112308	C6orf62	1.639872134	18.2780242	0.012581856
ENSG00000133800	LYVE1	1.639458105	17.14411595	0.016823432
ENSG00000111348	ARHGDIB	1.634901992	17.07217314	0.016900462
ENSG00000101391	CDK5RAP1	1.632345336	12.87947099	0.045450717
ENSG00000138175	ARL3	1.631483207	15.09251975	0.027595506
ENSG00000100749	VRK1	1.629698249	15.80246599	0.023413994
ENSG00000104325	DECR1	1.628853731	14.91088684	0.028209599
ENSG00000163468	CCT3	1.624431945	18.05731378	0.013017131
ENSG00000017427	IGF1	1.624381015	17.82439922	0.013774695
ENSG00000165168	CYBB	1.623176701	13.55945913	0.038706197
ENSG00000118971	CCND2	1.620257104	13.14011332	0.042088953
ENSG00000239900	ADSL	1.61952509	15.92538466	0.022743234
ENSG00000081189	MEF2C	1.616495952	18.02737075	0.013017131
ENSG00000136247	ZDHHC4	1.614826731	13.44940565	0.039522879
ENSG00000159720	ATP6V0D1	1.60977223	18.912579	0.01146081
ENSG00000083845	RPS5	1.603802357	17.78953771	0.013779314
ENSG00000104413	ESRP1	1.603102991	14.09741538	0.034151631

ENSG00000146386	ABRACL	1.599291311	16.86083412	0.017926708
ENSG00000135314	KHDC1	1.596126423	16.73683858	0.018676124
ENSG00000154556	SORBS2	1.592699308	15.47231814	0.024978346
ENSG00000169908	TM4SF1	1.589564333	16.56068235	0.019645241
ENSG00000002933	TMEM176A	1.587492952	13.64444017	0.038174374
ENSG00000133961	NUMB	1.583936296	14.55262623	0.030459709
ENSG00000163131	CTSS	1.579501779	15.0329039	0.027944898
ENSG00000106245	BUD31	1.579353971	13.62340658	0.038347664
ENSG00000240563	L1TD1	1.577453746	14.36018044	0.031731186
ENSG00000100823	APEX1	1.577272445	14.29473772	0.032285964
ENSG00000197614	MFAP5	1.573297462	12.71264117	0.04761947
ENSG00000178980	SELENOW	1.571145086	15.08553592	0.027595506
ENSG00000100353	EIF3D	1.570244174	16.43564516	0.020387174
ENSG00000122884	P4HA1	1.56971239	14.35009607	0.031731186
ENSG00000124784	RIOK1	1.569534741	16.79064415	0.018361754
ENSG00000006715	VPS41	1.564823023	16.27155205	0.020895977
ENSG00000075914	EXOSC7	1.562166215	16.53883681	0.019645241
ENSG00000148484	RSU1	1.560409285	15.45996879	0.024978346
ENSG00000000003	TSPAN6	1.558722131	15.40192615	0.025127262
ENSG00000135778	NTPCR	1.557700964	15.55039104	0.024337717
ENSG00000198522	GPN1	1.552758139	14.02185328	0.03471485
ENSG00000243749	TMEM35B	1.551368441	17.09714801	0.016897845
ENSG00000124570	SERPINB6	1.55032155	17.32529173	0.015993024
ENSG00000100883	SRP54	1.548101201	14.9745443	0.027967035
ENSG00000151465	CDC123	1.545503187	15.45731553	0.024978346
ENSG00000152944	MED21	1.540962049	13.77528052	0.037072504
ENSG00000153140	CETN3	1.538804649	16.99596555	0.017207854
NA	SNORD3B-1	1.537577357	14.00101328	0.034732922
ENSG00000260314	MRC1	1.531459835	15.60259767	0.024255691

NA	SNORD3B-2	1.53050009	13.83076999	0.036364502
ENSG00000108107	RPL28	1.530379545	13.36475877	0.040157453
ENSG00000176102	CSTF3	1.528633779	13.57521395	0.038706197
ENSG00000164172	MOCS2	1.527439779	16.22609807	0.020925968
ENSG00000131055	COX4I2	1.527138432	12.98795721	0.043856516
NA	SNORD3A	1.52324401	13.73784916	0.037353762
ENSG00000125445	MRPS7	1.523199132	15.41183208	0.025127262
ENSG00000127952	STYXL1	1.519879753	16.33468172	0.020829652
ENSG00000130830	MPP1	1.514923285	14.16090788	0.033862873
ENSG00000175183	CSRP2	1.513588003	14.45931315	0.031027408
NA	SNORD3C	1.513497965	13.54250365	0.038706197
ENSG00000198805	PNP	1.513398771	14.55278778	0.030459709
ENSG00000129538	RNASE1	1.512384296	12.83925129	0.04608051
ENSG00000152413	HOMER1	1.508475434	13.73631666	0.037353762
ENSG00000084623	EIF3I	1.507610723	15.87593441	0.02311646
ENSG00000142089	IFITM3	1.503816927	13.70480394	0.037577032
ENSG00000136045	PWP1	1.503398884	15.68018401	0.023796889
ENSG00000072694	FCGR2B	1.503376775	13.43427641	0.039658021
ENSG00000112118	MCM3	1.50315827	14.46033815	0.031027408
ENSG00000105193	RPS16	1.503060155	12.67863731	0.048238321
ENSG00000115486	GGCX	1.501375996	13.3629592	0.040157453
ENSG00000146376	ARHGAP18	1.498862908	14.35282444	0.031731186
NA	ANXA2P2	1.495109532	13.4947358	0.039189552
ENSG00000086696	HSD17B2	1.494165275	13.09785664	0.042662209
ENSG00000144642	RBMS3	1.491085164	17.09267256	0.016897845
ENSG00000107798	LIPA	1.480922375	13.24163984	0.041328629
ENSG00000134291	TMEM106C	1.479460738	13.28322025	0.040983518
ENSG00000138363	ATIC	1.475345556	14.02049455	0.03471485
ENSG00000203875	SNHG5	1.475264081	14.65938704	0.029981069

ENSG00000138279	ANXA7	1.474386268	14.79340769	0.028774977
ENSG00000006625	GGCT	1.473605027	14.64154351	0.030085058
ENSG00000166794	PPIB	1.470539048	12.81672905	0.046125345
ENSG00000139684	ESD	1.466255972	14.52513981	0.030715047
ENSG00000111481	COPZ1	1.464799425	14.83687381	0.028512432
ENSG00000143995	MEIS1	1.464605407	13.48701648	0.039204399
ENSG00000099797	TECR	1.453695019	14.11216679	0.034151631
ENSG00000113851	CRBN	1.44959089	14.46478759	0.031027408
ENSG00000139329	LUM	1.445922593	12.81158202	0.046125345
ENSG00000135698	MPHOSPH6	1.445747839	13.37372415	0.040157453
ENSG00000106066	CPVL	1.445746174	13.18170046	0.041583844
ENSG00000153015	CWC27	1.445459132	13.88743552	0.036094145
ENSG00000169764	UGP2	1.436475098	13.92722846	0.035699245
ENSG00000134333	LDHA	1.436388807	13.54511188	0.038706197
ENSG00000133026	MYH10	1.435638902	13.36143039	0.040157453
ENSG00000186197	EDARADD	1.431788262	13.34812519	0.040247597
ENSG00000121766	ZCCHC17	1.431315562	14.89861761	0.028209599
ENSG00000022277	RTF2	1.430575341	14.02866298	0.03471485
ENSG00000213614	HEXA	1.427179781	12.71324861	0.04761947
ENSG00000105053	VRK3	1.415218529	13.20451513	0.041346743
ENSG00000164692	COL1A2	1.411141324	13.66178948	0.038000287
ENSG00000205629	LCMT1	1.407855841	12.71802832	0.04761947
ENSG00000125991	ERGIC3	1.404882473	13.33565233	0.040354847
ENSG00000175110	MRPS22	1.403831286	13.21203703	0.041346743
ENSG00000114956	DGUOK	1.403232179	13.66463004	0.038000287
ENSG00000122862	SRGN	1.403072922	13.23745784	0.041328629
ENSG00000116171	SCP2	1.40232676	14.48382759	0.031027408
ENSG00000102893	PHKB	1.38861427	13.28145717	0.040983518
ENSG00000100348	TXN2	1.385964268	14.96612123	0.027967035

ENSG00000116729	WLS	1.385299585	12.75155762	0.047154628
ENSG00000091140	DLD	1.383792147	13.4165127	0.039855797
ENSG00000084072	PPIE	1.383474226	14.06937495	0.034322521
ENSG00000100528	CNIH1	1.377389982	13.21629095	0.041346743
ENSG00000166226	CCT2	1.370415059	13.20932308	0.041346743
ENSG00000143870	PDIA6	1.339859803	12.76379815	0.047017197
ENSG00000009844	VTA1	1.333155881	13.57114725	0.038706197
ENSG00000126821	SGPP1	-1.443101912	14.8631398	0.028299536
ENSG00000189143	CLDN4	-1.451761212	14.98740785	0.027944898
ENSG00000168003	SLC3A2	-1.523247116	14.35812714	0.031731186
ENSG00000122691	TWIST1	-1.526206968	15.09319806	0.027595506
ENSG00000144369	FAM171B	-1.546325035	15.30511686	0.025857737
ENSG00000259207	ITGB3	-1.641354415	14.15541401	0.033862873
ENSG00000127928	GNGT1	-1.763642138	13.46445476	0.03948373
ENSG00000165929	TC2N	-1.790536532	15.19518387	0.026892739
ENSG00000213145	CRIP1	-1.810010351	15.0177989	0.027944898
ENSG00000121898	CPXM2	-1.966788551	16.2220015	0.020925968
ENSG00000163347	CLDN1	-2.21168575	25.62288892	0.003051738
ENSG00000241104	LOC284344	-2.222646943	18.04610997	0.013017131
ENSG00000187094	CCK	-2.430438503	28.31878083	0.001904821
ENSG00000165272	AQP3	-2.848767259	18.74839009	0.011823753
ENSG00000189334	S100A14	-3.772126824	15.20104217	0.026892739
ENSG00000144820	ADGRG7	-6.189865738	19.13965643	0.011384983

Table S9: GO terms with biological process significantly enriched with upregulated DEGs in Male preterm placentas

term_name	term_id	adjusted_p_value	term_size	query_size	intersection_size	intersections
detoxification	GO:0098754	8.05E-12	135	285	14	MT1G,MT1M,MT1HL1,MT2A,MT1E,GSTM3,GPX3,SELENOW,PRDX2,GSTP1,AKR1A1,GSR,TXNDC17,PRDX5
detoxification of copper ion	GO:0010273	2.55E-08	14	89	5	MT1G,MT1M,MT1HL1,MT2A,MT1E
stress response to copper ion	GO:1990169	2.55E-08	14	89	5	MT1G,MT1M,MT1HL1,MT2A,MT1E
detoxification of inorganic compound	GO:0061687	5.56E-08	16	89	5	MT1G,MT1M,MT1HL1,MT2A,MT1E
stress response to metal ion	GO:0097501	1.09E-07	18	89	5	MT1G,MT1M,MT1HL1,MT2A,MT1E
cytoplasmic translation	GO:0002181	2.40E-07	148	280	11	RPL28,DPH5,RPL18,RPL36,RPS5,RPS16,FAU,RPS21,RPL29,EIF3I,EIF3M
cellular response to zinc ion	GO:0071294	4.25E-07	23	89	5	MT1G,MT1M,MT1HL1,MT2A,MT1E
response to cadmium ion	GO:0046686	7.18E-07	58	172	7	MT1G,MT1M,MT1HL1,MT2A,MT1E,NUDT1,GPI
cellular response to copper ion	GO:0071280	8.27E-07	26	89	5	MT1G,MT1M,MT1HL1,MT2A,MT1E
cellular response to cadmium ion	GO:0071276	4.69E-06	36	89	5	MT1G,MT1M,MT1HL1,MT2A,MT1E
aerobic respiration	GO:0009060	4.80E-06	192	286	11	NUPR1,ARL2,COX4I2,NDUFB2,COX7A1,ADSL,NDUFS4,ATP5F1E,UQCC2,UQCRHL,UQCRQ
cellular detoxification	GO:1990748	6.10E-06	110	285	9	GSTM3,GPX3,SELENOW,PRDX2,GSTP1,AKR1A1,GSR,TXNDC17,PRDX5
cellular zinc ion homeostasis	GO:0006882	6.23E-06	38	89	5	MT1G,MT1M,MT1HL1,MT2A,MT1E
zinc ion homeostasis	GO:0055069	8.15E-06	40	89	5	MT1G,MT1M,MT1HL1,MT2A,MT1E
response to copper ion	GO:0046688	8.15E-06	40	89	5	MT1G,MT1M,MT1HL1,MT2A,MT1E
cellular response to toxic substance	GO:0097237	1.14E-05	118	285	9	GSTM3,GPX3,SELENOW,PRDX2,GSTP1,AKR1A1,GSR,TXNDC17,PRDX5
cell killing	GO:0001906	1.15E-05	174	133	8	GZMB,CD5L,CR1L,ROMO1,IL18,TYROBP,LGALS9,HPRT1
electron transport chain	GO:0022900	2.83E-05	176	286	10	ETFB,COX4I2,CYB5A,NDUFB2,COX7A1,NDUFS4,AKR1A1,GSR,UQCRHL,UQCRQ
cellular response to metal ion	GO:0071248	3.04E-05	190	89	7	MT1G,MT1M,RASA4B,MT1HL1,MT2A,CRHBP,MT1E
response to zinc ion	GO:0010043	3.17E-05	52	89	5	MT1G,MT1M,MT1HL1,MT2A,MT1E
oxidative phosphorylation	GO:0006119	5.94E-05	142	286	9	NUPR1,COX4I2,NDUFB2,COX7A1,NDUFS4,ATP5F1E,UQCC2,UQCRHL,UQCRQ
granulocyte chemotaxis	GO:0071621	6.06E-05	129	222	8	CXCL9,CXCL10,SLAMF1,PF4,PPBP,CD74,FCER1G,MPP1
purine-containing compound biosynthetic process	GO:0072522	8.33E-05	200	282	10	PRTFDC1,HPRT1,NME1,PNP,ALDOA,ADSL,ATP5MC3,ATP5F1E,DMAC2L,ATP5MC1
negative regulation of cellular amide metabolic process	GO:0034249	0.000169283	194	236	9	EIF4EBP3,GZMB,EXOSC5,RTN2,DCPS,LSM1,CLU,EXOSC7,IGF1

antimicrobial humoral immune response mediated by antimicrobial peptide	GO:0061844	0.000176677	82	79	5	CXCL9,CXCL10,PF4,PPBP,ROMO1
regulation of complement activation	GO:0030449	0.000254644	22	124	4	CD5L,CR1L,VSIG4,SERPING1
granulocyte migration	GO:0097530	0.000265053	156	222	8	CXCL9,CXCL10,SLAMF1,PF4,PPBP,CD74,FCER1G,MPP1
neutrophil chemotaxis	GO:0030593	0.000297519	106	222	7	CXCL9,CXCL10,PF4,PPBP,CD74,FCER1G,MPP1
cellular modified amino acid metabolic process	GO:0006575	0.000600707	192	277	9	GSTM3,HPGDS,PLA2G15,CKMT1B,PLA2G2A,DIO3,GSTP1,GSR,GSTA4
establishment of protein localization to mitochondrion	GO:0072655	0.000701022	124	215	7	GZMB,ROMO1,TIMM8B,TOMM5,TIMM17B,TIMM23,TIMM9
cellular oxidant detoxification	GO:0098869	0.000756944	95	285	7	GPX3,SELENOW,PRDX2,GSTP1,GSR,TXNDC17,PRDX5
protein localization to mitochondrion	GO:0070585	0.000917167	129	215	7	GZMB,ROMO1,TIMM8B,TOMM5,TIMM17B,TIMM23,TIMM9
positive regulation of leukocyte differentiation	GO:1902107	0.000971304	158	260	8	PF4,ZBTB16,IL18,TYROBP,LGALS9,CD74,PNP,HCLS1
positive regulation of hemopoiesis	GO:1903708	0.000971304	158	260	8	PF4,ZBTB16,IL18,TYROBP,LGALS9,CD74,PNP,HCLS1
neutrophil migration	GO:1990266	0.00113749	129	222	7	CXCL9,CXCL10,PF4,PPBP,CD74,FCER1G,MPP1
cell redox homeostasis	GO:0045454	0.001359961	35	285	5	PRDX2,APEX1,TXN2,GSR,PRDX5
antimicrobial humoral response	GO:0019730	0.001397798	124	79	5	CXCL9,CXCL10,PF4,PPBP,ROMO1
purine ribonucleoside triphosphate biosynthetic process	GO:0009206	0.001446448	66	282	6	NME1,ALDOA,ATP5MC3,ATP5F1E,DMAC2L,ATP5MC1
purine nucleoside triphosphate biosynthetic process	GO:0009145	0.001582633	67	282	6	NME1,ALDOA,ATP5MC3,ATP5F1E,DMAC2L,ATP5MC1
cellular transition metal ion homeostasis	GO:0046916	0.001599152	113	89	5	MT1G,MT1M,MT1HL1,MT2A,MT1E
neutrophil activation	GO:0042119	0.00165295	38	272	5	IL18,TYROBP,PLA2G2A,FCER1G,ANXA3
nucleoside diphosphate metabolic process	GO:0009132	0.001713216	129	236	7	NUPR1,ARL2,NME1,GPI,ALDOA,MPP1,IGF1
myoblast differentiation	GO:0045445	0.001858999	85	122	5	CXCL9,CXCL10,LGALS1,IL18,SRA1
purine ribonucleoside salvage	GO:0006166	0.002253216	7	189	3	PRTFDC1,HPRT1,PNP
ribonucleoside triphosphate biosynthetic process	GO:0009201	0.002430648	72	282	6	NME1,ALDOA,ATP5MC3,ATP5F1E,DMAC2L,ATP5MC1
purine ribonucleotide biosynthetic process	GO:0009152	0.002840649	168	282	8	HPRT1,NME1,ALDOA,ADSL,ATP5MC3,ATP5F1E,DMAC2L,ATP5MC1
positive regulation of calcium ion transmembrane transport	GO:1904427	0.003344892	72	19	3	CXCL9,STAC3,CXCL10
granulocyte activation	GO:0036230	0.003499635	44	272	5	IL18,TYROBP,PLA2G2A,FCER1G,ANXA3

protein targeting to mitochondrion	GO:0006626	0.003511221	100	215	6	ROMO1,TIMM8B,TOMM5,TIMM17B,TIMM23,TIMM9
purine nucleoside metabolic process	GO:0042278	0.00379804	28	189	4	PRTFDC1,NUDT1,HPRT1,PNP
transition metal ion homeostasis	GO:0055076	0.004292282	138	89	5	MT1G,MT1M,MT1HL1,MT2A,MT1E
purine ribonucleoside triphosphate metabolic process	GO:0009205	0.004533637	80	282	6	NME1,ALDOA,ATP5MC3,ATP5F1E,DMAC2L,ATP5MC1
ribonucleotide biosynthetic process	GO:0009260	0.005167322	182	282	8	HPRT1,NME1,ALDOA,ADSL,ATP5MC3,ATP5F1E,DMAC2L,ATP5MC1
nucleoside salvage	GO:0043174	0.005383222	9	189	3	PRTFDC1,HPRT1,PNP
regulation of humoral immune response	GO:0002920	0.005471875	46	124	4	CD5L,CR1L,VSIG4,SERPING1
nucleoside triphosphate biosynthetic process	GO:0009142	0.005629386	83	282	6	NME1,ALDOA,ATP5MC3,ATP5F1E,DMAC2L,ATP5MC1
glycosyl compound metabolic process	GO:1901657	0.005927506	89	265	6	PRTFDC1,NUDT1,HPRT1,PNP,TK1,AKR1A1
nucleoside diphosphate phosphorylation	GO:0006165	0.006372756	101	236	6	NUPR1,ARL2,NME1,GPI,ALDOA,IGF1
purine nucleoside triphosphate metabolic process	GO:0009144	0.006473017	85	282	6	NME1,ALDOA,ATP5MC3,ATP5F1E,DMAC2L,ATP5MC1
negative regulation of complement activation	GO:0045916	0.006515583	14	124	3	CR1L,VSIG4,SERPING1
nucleotide phosphorylation	GO:0046939	0.006749455	102	236	6	NUPR1,ARL2,NME1,GPI,ALDOA,IGF1
ribose phosphate biosynthetic process	GO:0046390	0.006838279	189	282	8	HPRT1,NME1,ALDOA,ADSL,ATP5MC3,ATP5F1E,DMAC2L,ATP5MC1
macrophage activation	GO:0042116	0.007019325	101	240	6	TYROBP,VSIG4,IFI35,CD74,CLU,CTSC
purine nucleotide biosynthetic process	GO:0006164	0.007110778	190	282	8	HPRT1,NME1,ALDOA,ADSL,ATP5MC3,ATP5F1E,DMAC2L,ATP5MC1
protein tetramerization	GO:0051262	0.00718135	89	274	6	HPRT1,ALDOA,TK1,HOMER1,ACOT13,CBY1
ribonucleoside triphosphate metabolic process	GO:0009199	0.007416843	87	282	6	NME1,ALDOA,ATP5MC3,ATP5F1E,DMAC2L,ATP5MC1
purine nucleoside diphosphate metabolic process	GO:0009135	0.007557299	104	236	6	NUPR1,ARL2,GPI,ALDOA,MPP1,IGF1
purine ribonucleoside diphosphate metabolic process	GO:0009179	0.007557299	104	236	6	NUPR1,ARL2,GPI,ALDOA,MPP1,IGF1
positive regulation of viral entry into host cell	GO:0046598	0.007576944	12	154	3	LGALS1,LGALS9,CD74
positive regulation by symbiont of entry into host	GO:0075294	0.007576944	12	154	3	LGALS1,LGALS9,CD74
aerobic electron transport chain	GO:0019646	0.008040642	87	286	6	COX4I2,NDUFB2,COX7A1,NDUFS4,UQCRHL,UQCRQ

negative regulation of immune effector process	GO:0002698	0.008241974	113	124	5	CR1L,SLAMF1,LGALS9,VSIG4,SERPING1
protein insertion into mitochondrial membrane	GO:0051204	0.008430434	30	215	4	GZMB,ROMO1,TIMM8B,TIMM9
tumor necrosis factor production	GO:0032640	0.008522939	160	242	7	SLAMF1,PF4,TYROBP,LGALS9,CLU,IGF1,GSTP1
regulation of tumor necrosis factor production	GO:0032680	0.008522939	160	242	7	SLAMF1,PF4,TYROBP,LGALS9,CLU,IGF1,GSTP1
chemokine-mediated signaling pathway	GO:0070098	0.00906344	87	74	4	CXCL9,CXCL10,PF4,PPBP
regulation of myoblast differentiation	GO:0045661	0.009818325	54	122	4	CXCL9,CXCL10,IL18,SRA1
glutathione derivative metabolic process	GO:1901685	0.009914343	8	265	3	GSTM3,GSTP1,AKR1A1
glutathione derivative biosynthetic process	GO:1901687	0.009914343	8	265	3	GSTM3,GSTP1,AKR1A1
myeloid cell activation involved in immune response	GO:0002275	0.01007239	95	272	6	SLAMF1,TYROBP,LGALS9,IFI35,FCER1G,ANXA3
tumor necrosis factor superfamily cytokine production	GO:0071706	0.010439603	165	242	7	SLAMF1,PF4,TYROBP,LGALS9,CLU,IGF1,GSTP1
regulation of tumor necrosis factor superfamily cytokine production	GO:1903555	0.010439603	165	242	7	SLAMF1,PF4,TYROBP,LGALS9,CLU,IGF1,GSTP1
ribonucleoside diphosphate metabolic process	GO:0009185	0.010464984	110	236	6	NUPR1,ARL2,GPI,ALDOA,MPP1,IGF1
mitochondrial transmembrane transport	GO:1990542	0.011257543	104	253	6	SLC25A29,ROMO1,MPC1,TIMM17B,TIMM23,ATP5F1E
regulation of viral entry into host cell	GO:0046596	0.011828275	45	154	4	LGALS1,LGALS9,IFITM3,CD74
amine metabolic process	GO:0009308	0.01216361	140	190	6	SLC7A7,NNMT,INMT,SAT2,HPRT1,COMT
nucleoside metabolic process	GO:0009116	0.012679278	65	237	5	PRTFDC1,NUDT1,HPRT1,PNP,TK1
ATP biosynthetic process	GO:0006754	0.01283507	55	282	5	ALDOA,ATP5MC3,ATP5F1E,DMAC2L,ATP5MC1
protein homotetramerization	GO:0051289	0.013339331	57	274	5	HPRT1,ALDOA,TK1,ACOT13,CBY1
mitochondrial ATP synthesis coupled electron transport	GO:0042775	0.013414687	95	286	6	COX4I2,NDUFB2,COX7A1,NDUFS4,UQCRHL,UQCRQ
ATP synthesis coupled electron transport	GO:0042773	0.013414687	95	286	6	COX4I2,NDUFB2,COX7A1,NDUFS4,UQCRHL,UQCRQ
response to chemokine	GO:1990868	0.013416177	96	74	4	CXCL9,CXCL10,PF4,PPBP
cellular response to chemokine	GO:1990869	0.013416177	96	74	4	CXCL9,CXCL10,PF4,PPBP
regulation of complement-dependent cytotoxicity	GO:1903659	0.013643912	8	29	2	CD5L,CR1L

ribonucleoside biosynthetic process	GO:0042455	0.014003259	12	189	3	PRTFDC1,HPRT1,PNP
purine nucleoside biosynthetic process	GO:0042451	0.014003259	12	189	3	PRTFDC1,HPRT1,PNP
purine ribonucleoside biosynthetic process	GO:0046129	0.014003259	12	189	3	PRTFDC1,HPRT1,PNP
establishment of protein localization to mitochondrial membrane	GO:0090151	0.014110777	34	215	4	GZMB,ROMO1,TIMM8B,TIMM9
negative regulation of humoral immune response	GO:0002921	0.014520408	18	124	3	CR1L,VSIG4,SERPING1
myotube differentiation	GO:0014902	0.014836096	118	19	3	CXCL9,STAC3,CXCL10
leukocyte mediated cytotoxicity	GO:0001909	0.014952995	119	133	5	GZMB,IL18,TYROBP,LGALS9,HPRT1
complement activation	GO:0006956	0.015123483	128	216	6	CD5L,CR1L,VSIG4,SERPING1,C1QB,CLU
positive regulation of calcium ion transport	GO:0051928	0.015997534	121	19	3	CXCL9,STAC3,CXCL10
purine-containing compound salvage	GO:0043101	0.018162985	13	189	3	PRTFDC1,HPRT1,PNP
proton transmembrane transport	GO:1902600	0.018769285	155	282	7	COX4I2,CYB5A,COX7A1,ATP5MC3,ATP5F1E,DMAC2L,ATP5MC1
positive regulation of macrophage derived foam cell differentiation	GO:0010744	0.019156489	18	136	3	PF4,IL18,PLA2G2A
protein insertion into mitochondrial inner membrane	GO:0045039	0.020594848	12	215	3	ROMO1,TIMM8B,TIMM9
modulation by symbiont of entry into host	GO:0052372	0.021201023	52	154	4	LGALS1,LGALS9,IFITM3,CD74
regulation of neuroinflammatory response	GO:0150077	0.021761131	34	240	4	IL18,NUPR1,IGF1,CTSC
complement-dependent cytotoxicity	GO:0097278	0.021914882	10	29	2	CD5L,CR1L
glutathione metabolic process	GO:0006749	0.023090419	63	277	5	GSTM3,HPGDS,GSTP1,GSR,GSTA4
regulation of syncytium formation by plasma membrane fusion	GO:0060142	0.023488159	28	92	3	CXCL9,CXCL10,TYROBP
regulation of aerobic respiration	GO:1903715	0.024030702	31	271	4	NUPR1,ARL2,COX7A1,UQCC2
positive regulation of peptidase activity	GO:0010952	0.024450941	189	159	6	FAM162A,PCOLCE,LGALS9,LAPTM5,BEX3,DIABLO
inner mitochondrial membrane organization	GO:0007007	0.024685873	39	215	4	ROMO1,MTX1,TIMM8B,TIMM9

nucleoside triphosphate metabolic process	GO:0009141	0.02593255	108	282	6	NME1,ALDOA,ATP5MC3,ATP5F1E,DMAC2L,ATP5MC1
positive regulation of T cell differentiation	GO:0045582	0.026006441	94	189	5	ZBTB16,IL18,LGALS9,CD74,PNP
positive regulation of NIK/NF-kappaB signaling	GO:1901224	0.028226912	66	130	4	IL18,LGALS9,IFI35,LAPTM5
mitochondrial gene expression	GO:0140053	0.028557409	111	279	6	TFB1M,TSFM,MRPL47,MRPS7,UQCC2,MRPS21
positive regulation of cation transmembrane transport	GO:1904064	0.02925382	148	19	3	CXCL9,STAC3,CXCL10
protein alkylation	GO:0008213	0.02955851	190	96	5	NTMT1,METTL22,NNMT,SMYD3,PIH1D1
protein methylation	GO:0006479	0.02955851	190	96	5	NTMT1,METTL22,NNMT,SMYD3,PIH1D1
regulation of cell killing	GO:0031341	0.030621102	94	93	4	CD5L,CR1L,TYROBP,LGALS9
regulation of calcium ion transmembrane transport	GO:1903169	0.032310136	153	19	3	CXCL9,STAC3,CXCL10
exonucleolytic catabolism of deadenylated mRNA	GO:0043928	0.032662529	13	230	3	EXOSC5,DCPS,EXOSC7
nucleoside monophosphate metabolic process	GO:0009123	0.033111349	79	237	5	HPRT1,PNP,MPP1,ADSL,TK1
neuroinflammatory response	GO:0150076	0.034214461	38	240	4	IL18,NUPR1,IGF1,CTSC
regulation of biological process involved in symbiotic interaction	GO:0043903	0.035159422	59	154	4	LGALS1,LGALS9,IFITM3,CD74
nucleoside biosynthetic process	GO:0009163	0.035322734	16	189	3	PRTFDC1,HPRT1,PNP
positive regulation of alpha-beta T cell differentiation	GO:0046638	0.037368267	49	189	4	ZBTB16,IL18,LGALS9,PNP
positive regulation of lymphocyte proliferation	GO:0050671	0.038392389	138	236	6	SLAMF1,IL18,LGALS9,CD74,PNP,IGF1
negative regulation of myoblast differentiation	GO:0045662	0.038583874	25	122	3	CXCL10,IL18,SRA1
glycolytic process	GO:0006096	0.038914576	82	236	5	NUPR1,ARL2,GPI,ALDOA,IGF1
regulation of fibroblast proliferation	GO:0048145	0.03891921	80	242	5	NUPR1,CD74,NDUFS4,IGF1,GSTP1
regulation of myoblast fusion	GO:1901739	0.038933778	20	19	2	CXCL9,CXCL10
positive regulation of ion transmembrane transport	GO:0034767	0.039039804	163	19	3	CXCL9,STAC3,CXCL10
respiratory electron transport chain	GO:0022904	0.040184007	115	286	6	COX4I2,NDUFB2,COX7A1,NDUFS4,UQCRHL,UQCRQ
purine ribonucleoside monophosphate metabolic process	GO:0009167	0.04051374	42	226	4	HPRT1,PNP,MPP1,ADSL
ATP generation from ADP	GO:0006757	0.041280229	83	236	5	NUPR1,ARL2,GPI,ALDOA,IGF1

fibroblast proliferation	GO:0048144	0.041347244	81	242	5	NUPR1,CD74,NDUFS4,IGF1,GSTP1
positive regulation of mononuclear cell proliferation	GO:0032946	0.041654558	140	236	6	SLAMF1,IL18,LGALS9,CD74,PNP,IGF1
nucleobase-containing small molecule biosynthetic process	GO:0034404	0.042794751	17	189	3	PRTFDC1,HPRT1,PNP
regulation of T cell differentiation	GO:0045580	0.04637565	153	220	6	ZBTB16,IL18,LGALS9,CD74,PNP,PRDX2
positive regulation of cysteine-type endopeptidase activity involved in apoptotic process	GO:0043280	0.046817076	126	159	5	FAM162A,LGALS9,LAPTM5,BEX3,DIABLO
purine nucleoside monophosphate metabolic process	GO:0009126	0.048853622	44	226	4	HPRT1,PNP,MPP1,ADSL
myotube cell development	GO:0014904	0.049615129	41	244	4	STAC3,SMYD3,IGF1,HOMER1

Table S10: GO terms with biological process significantly enriched with upregulated DEGs in Female preterm placentas.

term_name	term_id	adjusted_p_value	term_size	query_size	intersection_size	intersections
nucleotide biosynthetic process	GO:0009165	3.14E-07	253	337	14	NME7,TREM2,NNMT,SLC25A12,GUCY1A1,HPRT1,IDO1,PAPSS1,ADSL,PNP,ATIC,TECR,DGUOK,DLD
nucleoside phosphate biosynthetic process	GO:1901293	3.66E-07	256	337	14	NME7,TREM2,NNMT,SLC25A12,GUCY1A1,HPRT1,IDO1,PAPSS1,ADSL,PNP,ATIC,TECR,DGUOK,DLD
purine-containing compound biosynthetic process	GO:0072522	2.53626E-06	200	337	12	NME7,TREM2,SLC25A12,GUCY1A1,HPRT1,PAPSS1,ADSL,PNP,ATIC,TECR,DGUOK,DLD
positive regulation of defense response	GO:0031349	3.12139E-06	281	244	12	TREM2,PLA2G2A,PLA2G5,IDO1,S100A12,ALOX5AP,IL33,S100A8,POLR3C,IFI16,PLSCR1,MEF2C
detoxification	GO:0098754	3.57479E-06	135	310	10	ALDH1A1,CD36,GSTM3,ALOX5AP,CAT,FBLN5,MT2A,MT1E,SELENOW,ESD
purine ribonucleotide biosynthetic process	GO:0009152	4.93101E-06	168	337	11	NME7,TREM2,SLC25A12,GUCY1A1,HPRT1,PAPSS1,ADSL,ATIC,TECR,DGUOK,DLD
cellular lipid catabolic process	GO:0044242	5.88271E-06	218	333	12	PLA2G4C,PLB1,AOAH,PLA2G5,ENPP2,LIPG,ETFB,HACL1,IDH1,DECR1,HEXA,SCP2
ribonucleotide biosynthetic process	GO:0009260	1.14075E-05	182	337	11	NME7,TREM2,SLC25A12,GUCY1A1,HPRT1,PAPSS1,ADSL,ATIC,TECR,DGUOK,DLD
olefinic compound metabolic process	GO:0120254	1.44522E-05	153	28	5	ALDH1A1,PLA2G4C,TTR,EPHX1,PLB1
ribose phosphate biosynthetic process	GO:0046390	1.68958E-05	189	337	11	NME7,TREM2,SLC25A12,GUCY1A1,HPRT1,PAPSS1,ADSL,ATIC,TECR,DGUOK,DLD

purine nucleotide biosynthetic process	GO:0006164	1.78472E-05	190	337	11	NME7,TREM2,SLC25A12,GUCY1A1,HPRT1,PAPSS1,ADSL,ATIC,TECR,DGUOK,DLD
negative regulation of defense response	GO:0031348	3.42974E-05	231	295	11	IL2RA,METTL3,TREM2,AOAH,SERPING1,VSIG4,A2M,IFI16,IGF1,TSPAN6,FCGR2B
neutrophil migration	GO:1990266	3.79287E-05	129	309	9	CCL2,S100A12,PECAM1,DPP4,S100A8,ITGA1,SRP54,MPP1,PPIB
phospholipid catabolic process	GO:0009395	5.11922E-05	54	206	6	PLA2G4C,PLB1,PLA2G5,ENPP2,LIPG,IDH1
positive regulation of inflammatory response	GO:0050729	5.28277E-05	131	145	7	TREM2,PLA2G2A,IDO1,S100A12,ALOX5AP,IL33,S100A8
response to toxic substance	GO:0009636	6.21009E-05	233	310	11	ALDH1A1,EPHX1,CD36,GSTM3,ALOX5AP,CAT,FBLN5,MT2A,MT1E,SELENOW,ESD
hormone metabolic process	GO:0042445	7.86109E-05	229	134	8	ALDH1A1,TTR,PLB1,DIO2,SPP1,DPP4,CRABP2,DIO3
hormone catabolic process	GO:0042447	0.000108172	11	231	4	DIO2,SPP1,DIO3,HSD17B11
neutrophil chemotaxis	GO:0030593	0.000126079	106	309	8	CCL2,S100A12,DPP4,S100A8,ITGA1,SRP54,MPP1,PPIB
nucleic acid phosphodiester bond hydrolysis	GO:0090305	0.000152023	272	290	11	ENPP2,CPSF3,RIDA,EXOSC9,LAS1L,PLSCR1,ERI3,APEX1,EXOSC7,CSTF3,RNASE1
cellular detoxification	GO:1990748	0.000172537	110	310	8	ALDH1A1,CD36,GSTM3,ALOX5AP,CAT,FBLN5,SELENOW,ESD
mesenchyme migration	GO:0090131	0.000181891	5	136	3	ACTG2,ACTC1,ACTA2
granulocyte migration	GO:0097530	0.000195997	156	309	9	CCL2,S100A12,PECAM1,DPP4,S100A8,ITGA1,SRP54,MPP1,PPIB
purine nucleoside monophosphate metabolic process	GO:0009126	0.00023827	44	331	6	HPRT1,ADSL,MPP1,PNP,ATIC,DGUOK
tissue homeostasis	GO:0001894	0.000254086	274	303	11	SLC28A2,ALDH1A1,LTF,SPP1,PECAM1,POTEI,POTEJ,POTEE,ANGPT1,HOMER1,LIPA
phosphatidylcholine metabolic process	GO:0046470	0.000274067	77	97	5	PLA2G4C,PLB1,PLA2G2A,PLA2G5,ENPP2
cellular response to toxic substance	GO:0097237	0.00029751	118	310	8	ALDH1A1,CD36,GSTM3,ALOX5AP,CAT,FBLN5,SELENOW,ESD
positive regulation of ERK1 and ERK2 cascade	GO:0070374	0.0003026	211	240	9	CHI3L1,CD36,TREM2,PLA2G2A,CCL2,PLA2G5,ACTA2,ANGPT1,IGF1
regulation of innate immune response	GO:0045088	0.000309761	234	217	9	METTL3,TREM2,PLA2G5,SERPING1,VSIG4,A2M,POLR3C,IFI16,PLSCR1
tRNA metabolic process	GO:0006399	0.000373865	196	265	9	WARS1,THG1L,TARS2,RARS2,RTCB,EXOSC9,TARS1,CDK5RAP1,EXOSC7
regulation of complement activation	GO:0030449	0.000382424	22	147	4	TREM2,SERPING1,VSIG4,A2M
negative regulation of cell migration	GO:0030336	0.000408006	267	258	10	SEMA3A,CCL2,DPP4,DCN,IL33,APOD,COL3A1,ARHGDIB,MEF2C,APEX1

glycerophospholipid catabolic process	GO:0046475	0.000448644	34	97	4	PLA2G4C,PLB1,PLA2G5,ENPP2
negative regulation of immune response	GO:0050777	0.000469483	181	295	9	METTL3,TREM2,SERPING1,IL33,VSIG4,A2M,IFI16,COL3A1,FCGR2B
myeloid leukocyte migration	GO:0097529	0.00048016	227	309	10	TREM2,CCL2,S100A12,PECAM1,DPP4,S100A8,ITGA1,SRP54,MPP1,PPIB
epithelial cell migration	GO:0010631	0.000555406	292	244	10	SEMA3A,PRCP,MAPRE2,ENPP2,DPP4,DCN,SPARC,GPI,ANGPT1,MEF2C
granulocyte chemotaxis	GO:0071621	0.000576668	129	309	8	CCL2,S100A12,DPP4,S100A8,ITGA1,SRP54,MPP1,PPIB
epithelium migration	GO:0090132	0.000609822	295	244	10	SEMA3A,PRCP,MAPRE2,ENPP2,DPP4,DCN,SPARC,GPI,ANGPT1,MEF2C
regulation of epithelial cell migration	GO:0010632	0.000620275	226	244	9	SEMA3A,PRCP,MAPRE2,ENPP2,DCN,SPARC,GPI,ANGPT1,MEF2C
negative regulation of cytokine production	GO:0001818	0.000653239	275	332	11	LTF,IL1R2,TREM2,IDO1,IL33,VSIG4,APOD,ANGPT1,IGF1,FCGR2B,SRGN
negative regulation of cell motility	GO:2000146	0.000695421	283	258	10	SEMA3A,CCL2,DPP4,DCN,IL33,APOD,COL3A1,ARHGDIB,MEF2C,APEX1
snRNA processing	GO:0016180	0.000720988	35	265	5	INTS7,INTS9,INTS12,EXOSC9,EXOSC7
negative regulation of response to biotic stimulus	GO:0002832	0.000783789	106	267	7	LTF,METTL3,SERPING1,VSIG4,A2M,IFI16,TSPAN6
negative regulation of cellular component movement	GO:0051271	0.00089651	291	258	10	SEMA3A,CCL2,DPP4,DCN,IL33,APOD,COL3A1,ARHGDIB,MEF2C,APEX1
cellular carbohydrate metabolic process	GO:0044262	0.00098563	285	334	11	NNMT,AOAH,SLC25A12,COQ3,PLEK,PGD,IDH1,IGF1,UGP2,LCMT1,PHKB
cellular modified amino acid metabolic process	GO:0006575	0.00101827	192	305	9	DIO2,CKMT1B,PLA2G2A,GSTM3,DIO3,IDH1,PLSCR1,GSTA3,ATIC
positive regulation of DNA-binding transcription factor activity	GO:0051091	0.001160908	266	164	8	LTF,WNT2,CD36,S100A12,CAT,TAF12,S100A8,HCLS1
phosphatidylcholine acyl-chain remodeling	GO:0036151	0.001183092	19	56	3	PLA2G4C,PLB1,PLA2G2A
positive regulation of phosphatidylinositol 3-kinase signaling	GO:0014068	0.001254551	79	240	6	TREM2,DCN,CAT,HCLS1,ANGPT1,IGF1
positive regulation of growth	GO:0045927	0.001340314	254	309	10	AGR2,WNT2,CRABP2,DIO3,S100A8,EXOSC9,SHTN1,IGF1,MEF2C,PPIB
RNA phosphodiester bond hydrolysis	GO:0090501	0.001447685	155	290	8	CPSF3,RIDA,EXOSC9,ERI3,APEX1,EXOSC7,CSTF3,RNASE1
cellular hormone metabolic process	GO:0034754	0.00151319	136	333	8	ALDH1A1,TTR,PLB1,SPP1,CRABP2,HSD17B11,HSD17B2,SCP2

organophosphate catabolic process	GO:0046434	0.001792652	160	289	8	PLA2G4C,PLB1,HPRT1,PLA2G5,ENPP2,LIPG,IDH1,PNP
negative regulation of dendritic cell differentiation	GO:2001199	0.001871463	5	295	3	TMEM176B,TMEM176A,FCGR2B
maturation of 5.8S rRNA	GO:0000460	0.001981096	36	316	5	EXOSC9,LAS1L,ERI3,EXOSC7,MPHOSPH6
glycerolipid catabolic process	GO:0046503	0.002271946	67	171	5	PLA2G4C,PLB1,PLA2G5,ENPP2,LIPG
regulation of plasma lipoprotein particle levels	GO:0097006	0.002363741	70	303	6	CD36,TREM2,PLA2G2A,PLA2G5,LIPG,LIPA
positive regulation of phagocytosis	GO:0050766	0.002375274	65	76	4	CD36,TREM2,CCL2,PLA2G5
positive regulation of macrophage derived foam cell differentiation	GO:0010744	0.002519059	18	76	3	CD36,PLA2G2A,PLA2G5
isoprenoid metabolic process	GO:0006720	0.002587831	118	43	4	ALDH1A1,TTR,PLB1,PDSS2
negative regulation of humoral immune response	GO:0002921	0.002597638	18	295	4	SERPING1,VSIG4,A2M,FCGR2B
IMP metabolic process	GO:0046040	0.002964758	18	305	4	HPRT1,ADSL,PNP,ATIC
retinol metabolic process	GO:0042572	0.002992033	51	28	3	ALDH1A1,TTR,PLB1
complement activation	GO:0006956	0.003094927	128	172	6	TREM2,CFI,SERPING1,VSIG4,A2M,C7
cold-induced thermogenesis	GO:0106106	0.003154831	146	238	7	LCN2,ALDH1A1,CD36,DIO2,DHRS7B,PLAC8,DECR1
regulation of cold-induced thermogenesis	GO:0120161	0.003154831	146	238	7	LCN2,ALDH1A1,CD36,DIO2,DHRS7B,PLAC8,DECR1
production of molecular mediator involved in inflammatory response	GO:0002532	0.003188078	78	157	5	IL1R2,CD36,TREM2,ALOX5AP,APOD
positive regulation of developmental growth	GO:0048639	0.003388928	163	309	8	AGR2,WNT2,CRABP2,DIO3,SHTN1,IGF1,MEF2C,PPIB
RNA 3'-end processing	GO:0031123	0.003454719	126	280	7	INTS7,INTS12,CPSF3,EXOSC9,ERI3,EXOSC7,CSTF3
regulation of ERK1 and ERK2 cascade	GO:0070372	0.003493547	298	76	6	CHI3L1,CD36,TREM2,PLA2G2A,CCL2,PLA2G5
phosphatidylcholine catabolic process	GO:0034638	0.00362012	16	97	3	PLB1,PLA2G5,ENPP2
purine ribonucleoside monophosphate metabolic process	GO:0009167	0.003667122	42	305	5	HPRT1,ADSL,MPP1,PNP,ATIC
hemostasis	GO:0007599	0.003750996	230	298	9	CD36,SERPING1,F13A1,PLEK,GPI,ILK,PLSCR1,COL3A1,GGCX
collagen fibril organization	GO:0030199	0.003989741	71	327	6	COL11A1,VIPAS39,COL3A1,P4HA1,LUM,COL1A2
ncRNA 3'-end processing	GO:0043628	0.004031491	49	265	5	INTS7,INTS12,EXOSC9,ERI3,EXOSC7
amino sugar catabolic process	GO:0046348	0.004070909	15	108	3	ALDH1A1,CHI3L1,NPL

cellular response to oxidised low-density lipoprotein particle stimulus	GO:0140052	0.004318968	11	154	3	CD36,TREM2,CD68
cellular response to xenobiotic stimulus	GO:0071466	0.004652581	151	244	7	EPHX1,CRYZ,S100A12,GSTM3,CYP2J2,GSTA3,MEF2C
negative regulation of innate immune response	GO:0045824	0.004761677	69	193	5	METTL3,SERPING1,VSIG4,A2M,IFI16
response to nutrient	GO:0007584	0.004941385	153	243	7	SPP1,CAT,A2M,FOLR2,LIPG,CYBB,ADSL
regulation of humoral immune response	GO:0002920	0.004943232	46	295	5	TREM2,SERPING1,VSIG4,A2M,FCGR2B
rRNA processing	GO:0006364	0.00505247	225	316	9	WDR75,EXOSC9,LAS1L,ERI3,RIOK1,EXOSC7,PWP1,RPS16,MPHOSPH6
retina homeostasis	GO:0001895	0.005462782	79	173	5	SLC28A2,LTF,POTEI,POTEJ,POTEE
adaptive thermogenesis	GO:1990845	0.005559816	159	238	7	LCN2,ALDH1A1,CD36,DIO2,DHRS7B,PLAC8,DECR1
xenobiotic metabolic process	GO:0006805	0.005689886	107	229	6	EPHX1,CRYZ,S100A12,GSTM3,CYP2J2,GSTA3
response to cadmium ion	GO:0046686	0.005917648	58	241	5	CAT,GPI,MT2A,MT1E,CYBB
ATP metabolic process	GO:0046034	0.006118589	276	337	10	TREM2,SLC25A12,CCNB1,UQCRC2,GPI,IGF1,COX4I2,LDHA,DGUOK,DLD
tumor necrosis factor production	GO:0032640	0.006292285	160	241	7	LTF,CD36,TREM2,IL33,ANGPT1,IGF1,CYBB
regulation of tumor necrosis factor production	GO:0032680	0.006292285	160	241	7	LTF,CD36,TREM2,IL33,ANGPT1,IGF1,CYBB
carbohydrate derivative catabolic process	GO:1901136	0.007325596	172	325	8	ALDH1A1,CHI3L1,AOAH,HPRT1,NPL,LYVE1,PNP,HEXA
icosanoid metabolic process	GO:0006690	0.007337914	119	215	6	PLA2G4C,EPHX1,PLA2G5,ALOX5AP,LTA4H,CYP2J2
carboxylic acid catabolic process	GO:0046395	0.0076636	236	238	8	IDO1,NPL,RIDA,KYAT3,ETFB,HACL1,PGD,DECR1
tumor necrosis factor superfamily cytokine production	GO:0071706	0.007707855	165	241	7	LTF,CD36,TREM2,IL33,ANGPT1,IGF1,CYBB
regulation of tumor necrosis factor superfamily cytokine production	GO:1903555	0.007707855	165	241	7	LTF,CD36,TREM2,IL33,ANGPT1,IGF1,CYBB
nucleoside monophosphate metabolic process	GO:0009123	0.008009321	79	331	6	HPRT1,ADSL,MPP1,PNP,ATIC,DGUOK
negative regulation of complement activation	GO:0045916	0.008241161	14	147	3	SERPING1,VSIG4,A2M
organic acid catabolic process	GO:0016054	0.00866877	240	238	8	IDO1,NPL,RIDA,KYAT3,ETFB,HACL1,PGD,DECR1
regulation of interleukin-1 production	GO:0032652	0.00874008	110	240	6	IL1R2,CD36,TREM2,TRIM16,IFI16,IGF1

interleukin-1 production	GO:0032612	0.00874008	110	240	6	IL1R2,CD36,TREM2,TRIM16,IFI16,IGF1
regulation of phosphatidylinositol 3-kinase signaling	GO:0014066	0.00874008	110	240	6	TREM2,DCN,CAT,HCLS1,ANGPT1,IGF1
neuron apoptotic process	GO:0051402	0.009486659	237	244	8	TREM2,CCL2,DIABLO,DIO3,ITGA1,GPI,ANGPT1,MEF2C
purine nucleoside monophosphate biosynthetic process	GO:0009127	0.009634338	22	331	4	HPRT1,ADSL,ATIC,DGUOK
regulation of phagocytosis	GO:0050764	0.009978543	93	76	4	CD36,TREM2,CCL2,PLA2G5
exonucleolytic trimming involved in rRNA processing	GO:0000459	0.011245819	9	265	3	EXOSC9,ERI3,EXOSC7
exonucleolytic trimming to generate mature 3'-end of 5.8S rRNA from tricistronic rRNA transcript (SSU-rRNA, 5.8S rRNA, LSU-rRNA)	GO:0000467	0.011245819	9	265	3	EXOSC9,ERI3,EXOSC7
temperature homeostasis	GO:0001659	0.011259194	177	238	7	LCN2,ALDH1A1,CD36,DIO2,DHRS7B,PLAC8,DECR1
electron transport chain	GO:0022900	0.011302593	176	337	8	SLC25A12,IDO1,CCNB1,ETFB,CYBB,COX4I2,DGUOK,DLD
negative regulation of leukocyte migration	GO:0002686	0.011548029	47	157	4	CCL2,DPP4,IL33,APOD
positive regulation of chondrocyte proliferation	GO:1902732	0.011853156	8	31	2	LTF,SCUBE2
positive regulation of osteoblast differentiation	GO:0045669	0.01194547	66	244	5	LTF,SCUBE2,ILK,IGF1,MEF2C
cellular respiration	GO:0045333	0.012488635	236	337	9	SLC25A12,CCNB1,CAT,UQCRC2,IDH1,ADSL,COX4I2,DGUOK, DLD
defense response to fungus	GO:0050832	0.012680638	52	145	4	LTF,PLA2G5,S100A12,S100A8
retinoid metabolic process	GO:0001523	0.013552899	84	28	3	ALDH1A1,TTR,PLB1
regulation of macrophage derived foam cell differentiation	GO:0010743	0.013716811	31	76	3	CD36,PLA2G2A,PLA2G5
GMP metabolic process	GO:0046037	0.014038191	26	305	4	HPRT1,ADSL,MPP1,ATIC
snRNA metabolic process	GO:0016073	0.014146783	63	265	5	INTS7,INTS9,INTS12,EXOSC9,EXOSC7
viral genome replication	GO:0019079	0.014556332	130	338	7	LTF,CCL2,IFI16,PLSCR1,IFITM3,PPIB,PPIE
snRNA 3'-end processing	GO:0034472	0.014590414	30	265	4	INTS7,INTS12,EXOSC9,EXOSC7
cytoplasmic translation	GO:0002181	0.014728075	148	297	7	METTL3,DRG2,RPS5,EIF3D,RPL28,EIF3I,RPS16
diterpenoid metabolic process	GO:0016101	0.015062618	87	28	3	ALDH1A1,TTR,PLB1

rRNA 3'-end processing	GO:0031125	0.016014173	10	265	3	EXOSC9,ERI3,EXOSC7
tRNA aminoacylation for protein translation	GO:0006418	0.01746005	43	191	4	WARS1,TARS2,RARS2,TARS1
positive regulation of peptidyl-tyrosine phosphorylation	GO:0050731	0.017612005	188	240	7	CD36,TREM2,ENPP2,PECAM1,HCLS1,ANGPT1,IGF1
rRNA metabolic process	GO:0016072	0.017783559	263	316	9	WDR75,EXOSC9,LAS1L,ERI3,RIOK1,EXOSC7,PWP1,RPS16,MPHOSPH6
amyloid-beta clearance by cellular catabolic process	GO:0150094	0.018878748	8	39	2	CD36,TREM2
regulation of leukocyte differentiation	GO:1902105	0.018974104	284	295	9	LTF,IL2RA,METTL3,TREM2,HCLS1,TMEM176B,TMEM176A,PNP,FCGR2B
cellular response to molecule of bacterial origin	GO:0071219	0.019168832	216	295	8	LTF,CD36,TREM2,CCL2,CD68,MEF2C,MRC1,FCGR2B
negative regulation of endopeptidase activity	GO:0010951	0.020880134	240	193	7	LTF,SERPING1,A2M,COL6A3,LXN,GPI,IFI16
regulation of protein localization to nucleus	GO:1900180	0.021303809	137	340	7	CD36,MCRS1,APOD,HCLS1,ANGPT1,CCT3,CCT2
aerobic respiration	GO:0009060	0.021308997	192	337	8	CCNB1,CAT,UQCRC2,IDH1,ADSL,COX4I2,DGUOK,DLD
terpenoid metabolic process	GO:0006721	0.02154154	98	28	3	ALDH1A1,TTR,PLB1
macrophage derived foam cell differentiation	GO:0010742	0.021691514	36	76	3	CD36,PLA2G2A,PLA2G5
foam cell differentiation	GO:0090077	0.021691514	36	76	3	CD36,PLA2G2A,PLA2G5
ribonucleoside monophosphate metabolic process	GO:0009161	0.021886637	60	305	5	HPRT1,ADSL,MPP1,PNP,ATIC
regulation of toll-like receptor signaling pathway	GO:0034121	0.022562284	71	39	3	LTF,CD36,TREM2
negative regulation of epithelial cell proliferation	GO:0050680	0.022920032	128	244	6	EAF2,CCL2,SPARC,RIDA,IFT52,MEF2C
ether metabolic process	GO:0018904	0.023719107	27	335	4	PLA2G4C,EPHX1,DHRS7B,TXN2
negative regulation of protein import into nucleus	GO:0042308	0.023972041	14	210	3	CD36,APOD,ANGPT1
negative regulation of protein import	GO:1904590	0.023972041	14	210	3	CD36,APOD,ANGPT1
negative regulation of cytokine-mediated signaling pathway	GO:0001960	0.024530714	73	39	3	IL1R2,METTL3,TREM2
tRNA aminoacylation	GO:0043039	0.024991254	47	191	4	WARS1,TARS2,RARS2,TARS1
primary alcohol metabolic process	GO:0034308	0.025009479	103	28	3	ALDH1A1,TTR,PLB1

negative regulation of peptidase activity	GO:0010466	0.025794146	248	193	7	LTF,SERPING1,A2M,COL6A3,LXN,GPI,IFI16
myeloid leukocyte activation	GO:0002274	0.026575639	226	295	8	TREM2,PLA2G2A,MILR1,S100A12,IL33,VSIG4,PLSCR1,FCGR2B
blood coagulation	GO:0007596	0.026805357	224	298	8	CD36,SERPING1,F13A1,PLEK,ILK,PLSCR1,COL3A1,GGCX
amino acid activation	GO:0043038	0.027197104	48	191	4	WARS1,TARS2,RARS2,TARS1
response to fungus	GO:0009620	0.027344624	63	145	4	LTF,PLA2G5,S100A12,S100A8
phosphatidylglycerol metabolic process	GO:0046471	0.027690542	39	76	3	PLB1,PLA2G2A,PLA2G5
negative regulation of response to cytokine stimulus	GO:0060761	0.028800494	77	39	3	IL1R2,METTL3,TREM2
regulation of dendritic cell differentiation	GO:2001198	0.030225716	11	295	3	TMEM176B,TMEM176A,FCGR2B
cellular response to lipoteichoic acid	GO:0071223	0.030316512	10	39	2	CD36,TREM2
response to lipoteichoic acid	GO:0070391	0.030316512	10	39	2	CD36,TREM2
coagulation	GO:0050817	0.031411896	229	298	8	CD36,SERPING1,F13A1,PLEK,ILK,PLSCR1,COL3A1,GGCX
negative regulation of immune effector process	GO:0002698	0.032834621	113	295	6	SERPING1,IL33,VSIG4,A2M,ANGPT1,FCGR2B
acute inflammatory response	GO:0002526	0.032834621	113	295	6	PRCP,ALOX5AP,S100A8,A2M,PLSCR1,FCGR2B
IMP biosynthetic process	GO:0006188	0.033383436	11	305	3	HPRT1,ADSL,ATIC
lipid storage	GO:0019915	0.034791297	82	39	3	PLA2G4C,CD36,TREM2
leukotriene biosynthetic process	GO:0019370	0.036063952	20	165	3	PLA2G5,ALOX5AP,LTA4H
transition metal ion homeostasis	GO:0055076	0.03677877	138	246	6	LCN2,LTF,S100A8,MT2A,MT1E,ATP6V0D1
response to xenobiotic stimulus	GO:0009410	0.037466413	242	289	8	EPHX1,CRYZ,S100A12,GSTM3,CYP2J2,GSTA3,MEF2C,PNP
regulation of autophagic cell death	GO:1904092	0.038904264	5	93	2	TREM2,ATG7
regulation of mRNA catabolic process	GO:0061013	0.039451099	193	265	7	METTL3,TTC5,RIDA,EXOSC9,PSMC4,APEX1,EXOSC7
positive regulation of high-density lipoprotein particle clearance	GO:0010983	0.039699241	3	171	2	TREM2,LIPG
phosphatidylinositol 3-kinase signaling	GO:0014065	0.040721408	144	240	6	TREM2,DCN,CAT,HCLS1,ANGPT1,IGF1
regulation of I-kappaB kinase/NF-kappaB signaling	GO:0043122	0.041186341	253	82	5	LTF,CD36,TREM2,TNFSF10,S100A12
protein homotetramerization	GO:0051289	0.042919191	57	60	3	CRYZ,THG1L,HPRT1

cell recognition	GO:0008037	0.043427698	224	156	6	SEMA3A,CD36,TREM2,PLA2G5,PECAM1,FOLR2
endothelial cell migration	GO:0043542	0.045104923	214	244	7	PRCP,DPP4,DCN,SPARC,GPI,ANGPT1,MEF2C
cellular response to biotic stimulus	GO:0071216	0.045982764	244	295	8	LTF,CD36,TREM2,CCL2,CD68,MEF2C,MRC1,FCGR2B
cell-matrix adhesion	GO:0007160	0.047609437	226	233	7	CD36,APOD,ITGA1,FBLN5,ILK,COL3A1,LYVE1
purine ribonucleoside triphosphate biosynthetic process	GO:0009206	0.048572533	66	54	3	NME7,TREM2,SLC25A12
leukocyte chemotaxis	GO:0030595	0.048904101	235	309	8	CCL2,S100A12,DPP4,S100A8,ITGA1,SRP54,MPP1,PPIB
threonyl-tRNA aminoacylation	GO:0006435	0.049548417	3	191	2	TARS2,TARS1

Table S11: KEGG signalling pathways significantly enriched with upregulated DEGs in Male preterm placentas.

term_name	term_id	adjusted_p_value	term_size	query_size	intersection_size	intersections
Ribosome	KEGG:03010	2.72E-11	153	279	13	RPL28,RPL18,RPL36,RPS5,MRPL21,RPS16,FAU,MRPL17,MRPS7,RPS21,MRPL20,RPL29,MRPS21
Complement and coagulation cascades	KEGG:04610	5.96E-08	85	216	8	F10,KNG1,CR1L,VTN,VSIG4,SERPING1,C1QB,CLU
Oxidative phosphorylation	KEGG:00190	6.82E-08	134	286	10	NDUFA11,COX4I2,NDUFB2,COX7A1,NDUFS4,ATP5MC3,ATP5F1E,UQCRHL,ATP5MC1,UQCRQ
RNA polymerase	KEGG:03020	1.91E-07	34	98	5	POLR2L,POLR2J,POLR2J2,POLR2I,POLR2F
Mineral absorption	KEGG:04978	4.64E-07	60	254	7	MT1G,MT1M,MT1HL1,MT2A,MT1E,HMOX2,ATOX1
Glutathione metabolism	KEGG:00480	5.84E-07	57	277	7	GSTM3,HPGDS,GPX3,GGCT,GSTP1,GSR,GSTA4
Viral protein interaction with cytokine and cytokine receptor	KEGG:04061	2.81E-05	98	90	5	CXCL9,CXCL10,PF4,PPBP,IL18
Drug metabolism - other enzymes	KEGG:00983	0.000128114	79	277	6	GSTM3,HPRT1,NME1,TK1,GSTP1,GSTA4
Cytosolic DNA-sensing pathway	KEGG:04623	0.000241737	62	98	4	CXCL10,POLR2L,IL18,POLR2F
Non-alcoholic fatty liver disease	KEGG:04932	0.000693839	155	286	7	NDUFA11,COX4I2,NDUFB2,COX7A1,NDUFS4,UQCRHL,UQCRQ
RNA degradation	KEGG:03018	0.000967218	79	230	5	EXOSC5,DCPS,MPHOSPH6,LSM1,EXOSC7
Chemical carcinogenesis - DNA adducts	KEGG:05204	0.001134456	68	277	5	EPHX1,GSTM3,HPGDS,GSTP1,GSTA4
Metabolism of xenobiotics by cytochrome P450	KEGG:00980	0.001604931	73	277	5	EPHX1,GSTM3,HPGDS,GSTP1,GSTA4
HIF-1 signaling pathway	KEGG:04066	0.005168865	109	236	5	MKNK1,TIMP1,ALDOA,ELOB,IGF1
Chemokine signaling pathway	KEGG:04062	0.006581769	190	74	4	CXCL9,CXCL10,PF4,PPBP
Purine metabolism	KEGG:00230	0.008065326	125	226	5	HPRT1,NME1,GMPR,PNP,ADSL

Tuberculosis	KEGG:05152	0.01181611	175	175	5	CR1L,IL18,RFXANK,CD74,FCER1G
Toll-like receptor signaling pathway	KEGG:04620	0.016445148	102	69	3	CXCL9,CXCL10,SPP1
Drug metabolism - cytochrome P450	KEGG:00982	0.020065691	68	277	4	GSTM3,HPGDS,GSTP1,GSTA4
Arachidonic acid metabolism	KEGG:00590	0.026592915	61	136	3	HPGDS,GPX3,PLA2G2A
Natural killer cell mediated cytotoxicity	KEGG:04650	0.033903788	123	175	4	GZMB,ICAM2,TYROBP,FCER1G
Biosynthesis of cofactors	KEGG:01240	0.042740669	153	265	5	NME1,PMM1,ADSL,MOCS2,AKR1A1
Amino sugar and nucleotide sugar metabolism	KEGG:00520	0.046083048	49	205	3	PMM1,GPI,NPL

Table S12: KEGG signalling pathways significantly enriched with upregulated DEGs in Female preterm placentas.

term_name	term_id	adjusted_p_value	term_size	query_size	intersection_size	intersections
Ether lipid metabolism	KEGG:00565	1.22959E-06	49	97	5	PLA2G4C,PLB1,PLA2G2A,PLA2G5,ENPP2
Vascular smooth muscle contraction	KEGG:04270	1.78187E-06	134	136	7	PLA2G4C,ITPR1,PLA2G2A,GUCY1A1,PLA2G5,ACTG2,ACTA2
alpha-Linolenic acid metabolism	KEGG:00592	2.06833E-06	25	76	4	PLA2G4C,PLB1,PLA2G2A,PLA2G5
Purine metabolism	KEGG:00230	2.33131E-06	125	331	9	NME7,GUCY1A1,HPRT1,PAPSS1,ADSL,NTPCR,PNP,ATIC,DGUOK
Linoleic acid metabolism	KEGG:00591	3.86887E-06	29	76	4	PLA2G4C,PLB1,PLA2G2A,PLA2G5
Arachidonic acid metabolism	KEGG:00590	6.23868E-06	61	215	6	PLA2G4C,PLB1,PLA2G2A,PLA2G5,LTA4H,CYP2J2
Complement and coagulation cascades	KEGG:04610	1.2373E-05	85	172	6	CFI,SERPING1,F13A1,VSIG4,A2M,C7
Biosynthesis of cofactors	KEGG:01240	0.00018002	153	337	8	NME7,IDO1,COQ3,ADSL,MOCS2,GGCX,UGP2,DLD
Glycerophospholipid metabolism	KEGG:00564	0.00055162	98	76	4	PLA2G4C,PLB1,PLA2G2A,PLA2G5
Phagosome	KEGG:04145	0.000605371	147	295	7	CD36,FCGR2A,CYBB,ATP6V0D1,CTSS,MRC1,FCGR2B
Glutathione metabolism	KEGG:00480	0.000799286	57	308	5	GSTM3,PGD,IDH1,GSTA3,GGCT
Aminoacyl-tRNA biosynthesis	KEGG:00970	0.000860101	44	191	4	WARS1,TARS2,RARS2,TARS1
Fluid shear stress and atherosclerosis	KEGG:05418	0.000911528	138	335	7	IL1R2,CCL2,PECAM1,GSTM3,GSTA3,MEF2C,TXN2
Focal adhesion	KEGG:04510	0.001035029	200	327	8	PARVB,SPP1,COL6A3,ITGA1,ILK,IGF1,CCND2,COL1A2
Coronavirus disease - COVID-19	KEGG:05171	0.001465206	231	297	8	CCL2,F13A1,C7,FCGR2A,CYBB,RPS5,RPL28,RPS16
Protein digestion and absorption	KEGG:04974	0.00155053	103	327	6	PRCP,DPP4,COL11A1,COL6A3,COL3A1,COL1A2

Fat digestion and absorption	KEGG:04975	0.001672645	43	76	3	CD36,PLA2G2A,PLA2G5
Hematopoietic cell lineage	KEGG:04640	0.001746075	95	35	3	IL1R2,CD36,IL2RA
Long-term depression	KEGG:04730	0.001831582	59	57	3	PLA2G4C,ITPR1,GUCY1A1
FoxO signaling pathway	KEGG:04068	0.003145296	131	291	6	TNFSF10,CCNB1,CAT,IGF1,CCND2,HOMER1
Carbon metabolism	KEGG:01200	0.003418444	115	337	6	CAT,GPI,PGD,IDH1,ESD,DLD
Platelet activation	KEGG:04611	0.004410851	124	327	6	PLA2G4C,ITPR1,GUCY1A1,FCGR2A,COL3A1,COL1A2
Malaria	KEGG:05144	0.005470816	49	99	3	CD36,CCL2,PECAM1
Tryptophan metabolism	KEGG:00380	0.005920052	41	337	4	IDO1,CAT,KYAT3,DLD
Lysosome	KEGG:04142	0.006035261	132	325	6	CD68,AGA,ATP6V0D1,CTSS,LIPA,HEXA
ECM-receptor interaction	KEGG:04512	0.006561507	88	159	4	CD36,SPP1,COL6A3,ITGA1
Ovarian steroidogenesis	KEGG:04913	0.009082969	51	301	4	PLA2G4C,CYP2J2,IGF1,HSD17B2
Lipid and atherosclerosis	KEGG:05417	0.009437494	214	72	4	CD36,ITPR1,TNFSF10,CCL2
Proteoglycans in cancer	KEGG:05205	0.009681355	205	327	7	WNT2,ITPR1,DCN,HCLS1,IGF1,LUM,COL1A2
Amino sugar and nucleotide sugar metabolism	KEGG:00520	0.01041626	49	325	4	NPL,GPI,UGP2,HEXA
Tuberculosis	KEGG:05152	0.01665933	175	295	6	RFXANK,FCGR2A,ATP6V0D1,CTSS,MRC1,FCGR2B
Viral protein interaction with cytokine and cytokine receptor	KEGG:04061	0.016731925	98	72	3	IL2RA,TNFSF10,CCL2
Drug metabolism - other enzymes	KEGG:00983	0.017508917	79	229	4	NME7,HPRT1,GSTM3,GSTA3
Pancreatic secretion	KEGG:04972	0.022083633	102	76	3	ITPR1,PLA2G2A,PLA2G5
Cytokine-cytokine receptor interaction	KEGG:04060	0.028044183	293	126	5	IL1R2,IL2RA,TNFSF10,CCL2,IL33
Phospholipase D signaling pathway	KEGG:04072	0.028547259	147	12	2	RAPGEF4,PLA2G4C
Natural killer cell mediated cytotoxicity	KEGG:04650	0.031287933	123	71	3	CD48,ICAM2,TNFSF10
Oxytocin signaling pathway	KEGG:04921	0.031595793	154	57	3	PLA2G4C,ITPR1,GUCY1A1
Chemical carcinogenesis - reactive oxygen species	KEGG:05208	0.046418237	222	282	6	EPHX1,GSTM3,CAT,UQCRC2,GSTA3,COX4I2
Inflammatory mediator regulation of TRP channels	KEGG:04750	0.047478581	98	240	4	PLA2G4C,ITPR1,CYP2J2,IGF1

Table S13: miRNA targets detection analysis

miRNA	UP_genes	count_unique
hsa-miR-20a-5p	BAMBI, BMP2, BMP2, CELF2, CRAMP1, CRAMP1, CRAMP1, CRAMP1, KLHL28, KLHL28, RAPGEFL1, RAPGEFL1, RAPGEFL1, RAPGEFL1, RAPGEFL1, RAPGEFL1, REST, RHOBTB1, RHOBTB1, RHOBTB1, RHOBTB1, RHOBTB1, RHOBTB1, RRM2, RRM2, RRM2, RRM2, SCARB2, VANGL1	11
hsa-miR-23a-3p	CELF2, CELF2, CELF2, CELF2, CELF2, CELF2, CELF2, CELF2, CELF2, CELF2, CELF2, CELF2, CELF2, CELF2, CELF2, CELF2, CELF2, CELF2, CELF2, CPD, CPD, CPD, CPD, CPD, CPD, CPD, CPD, CPD, HNRNPA2B1, HNRNPA2B1, HNRNPA2B1, HNRNPA2B1, HNRNPA2B1, HNRNPA2B1, LHFPL2, LHFPL2, LHFPL2, LHFPL2, MEF2C, MEF2C, MEF2C, MEF2C, MEF2C, MEF2C, MEF2C, MEF2C, MEF2C, MEF2C, MEF2C, MEF2C, N4BP2L2, N4BP2L2, OTUB1, OTUB1, SLC4A4, SLC4A4, SLC4A4, SLC4A4, SLC4A4, SLC4A4, SLC4A4, TTLL7, ZNF430, ZNF430	10
hsa-miR-300	ANKRD28, ANKRD28, CELF2, CELF2, CELF2, CELF2, CELF2, CELF2, CELF2, CELF2, CELF2, CELF2, CPD, CPD, CPD, CPD, CPD, CPD, CPD, CPD, CPD, CPD, CPD, CPD, MAST4, MAST4, MAST4, MEF2C, MEF2C, MEF2C, MEF2C, MEF2C, PMP22, RAPGEFL1, RAPGEFL1, TAF4B, UBE3A, UBE3A, UBE3A, UBE3A, UBE3A, UBE3A, UBE3A, UBE3A, ZNF12, ZNF12, ZNF12, ZNF12, ZNF12, ZNF12, ZNF12, ZNF12, ZNF12, ZNF12, ZNF12, ZNF12	10
hsa-miR-424-5p	CLCN4, CLCN4, COPS7A, COPS7A, COPS7A, COPS7A, COPS7A, COPS7A, COPS7A, COPS7A, CPD, CPD, CPD, CPD, DMRT2, DMRT2, DMRT2, FLNB, FLNB, FLNB, OTUB1, OTUB1, PRKAR2A, PRKAR2A, PRKAR2A, PRKAR2A, PRKAR2A, PRKAR2A, RAPGEFL1, TRAM2, WIPI2, WIPI2, WIPI2, WIPI2, WIPI2	10
hsa-miR-497-5p	CLCN4, CLCN4, COPS7A, COPS7A, COPS7A, COPS7A, COPS7A, COPS7A, COPS7A, CPD, CPD, CPD, CPD, CRAMP1, CRAMP1, CRAMP1, CRAMP1, DMRT2, DMRT2, DMRT2, DMRT2, FLNB, FLNB, FLNB, NFATC4, NFATC4, NFATC4, NFATC4, NFATC4, NFATC4, NFATC4, OTUB1, OTUB1, PRKAR2A, PRKAR2A, PRKAR2A, PRKAR2A, PRKAR2A, RAPGEFL1	10
hsa-miR-101-3p	ANKRD28, ANKRD28, ANKRD28, ANKRD28, ANKRD28, ANKRD28, CELF2, EMP1, EMP1, EMP1, EMP1, HDAC9, HDAC9, MAST4, SLC4A4, SLC4A4, SLC4A4, SNX13, VANGL1, VANGL1, VANGL1, VANGL1, VANGL1, VANGL1, VANGL1, VANGL1, WWC3, WWC3	9
hsa-miR-106a-5p	BAMBI, CELF2, CLSTN1, CLSTN1, CLSTN1, CRAMP1, CRAMP1, CRAMP1, CRAMP1, CRAMP1, CRAMP1, RAPGEFL1, RAPGEFL1, RAPGEFL1, RAPGEFL1, RAPGEFL1, RAPGEFL1, REST, RHOBTB1, RHOBTB1, RHOBTB1, RHOBTB1, RHOBTB1, RHOBTB1, RRM2, RRM2, RRM2, RRM2, VANGL1, VANGL1, VANGL1, VANGL1, VANGL1, VANGL1, VANGL1, VANGL1, VANGL1, VANGL1, VANGL1, VANGL1	9

hsa-miR-106b-5p	BAMBI, CELF2, CRAMP1, CRAMP1, CRAMP1, CRAMP1, CRAMP1, CRAMP1, RAPGEFL1, RAPGEFL1, RAPGEFL1, RAPGEFL1, RAPGEFL1, RAPGEFL1, REST, RHOBTB1, RHOBTB1, RHOBTB1, RHOBTB1, RHOBTB1, RHOBTB1, RRM2, RRM2, RRM2, RRM2, SCARB2, SCARB2, SCARB2, SCARB2, SCARB2, SCARB2, SCARB2, SCARB2, SCARB2, SCARB2, SCARB2, SCARB2, SCARB2, SCARB2, SCARB2, SCARB2, VANGL1, VANGL1, VANGL1, VANGL1, VANGL1, VANGL1, VANGL1, VANGL1, VANGL1, VANGL1, VANGL1, VANGL1, VANGL1, VANGL1, VANGL1, VANGL1, VANGL1, VANGL1	9
hsa-miR-23b-3p	CELF2, CPD, CPD, CPD, CPD, CPD, CPD, CPD, CPD, CPD, CPD, CPD, CPD, HNRNPA2B1, HNRNPA2B1, HNRNPA2B1, HNRNPA2B1, HNRNPA2B1, HNRNPA2B1, LHFPL2, LHFPL2, LHFPL2, LHFPL2, MEF2C, MEF2C, MEF2C, MEF2C, MEF2C, MEF2C, MEF2C, MEF2C, MEF2C, MEF2C, MEF2C, MEF2C, MEF2C, N4BP2L2, N4BP2L2, OTUB1, OTUB1, SLC4A4, SLC4A4, SLC4A4, SLC4A4, SLC4A4, SLC4A4, SLC4A4, TTLL7	9
hsa-miR-27b-3p	BAG2, BAG2, BAG2, BAG2, CELF2, CELF2, CELF2, CELF2, CELF2, CELF2, ERLEC1, ERLEC1, LETMD1, MEF2C, MEF2C, MEF2C, MEF2C, MEF2C, MEF2C, MEF2C, MEF2C, MEF2C, NR1D2, NR1D2, NRG1, NRG1, NRG1, NRG1, NRG1, NRG1, NRG1, NRG1, NRG1, NRG1, VANGL1, VANGL1, VANGL1, VANGL1, VANGL1, VANGL1, VANGL1, VANGL1, VANGL1, VANGL1, VANGL1, VANGL1, VANGL1, VANGL1, VANGL1, VANGL1, VANGL1, VANGL1, ZFP36L2, ZFP36L2	9
hsa-miR-340-5p	ANKRD28, ANKRD28, CPD, CPD, CPD, EMP1, HDAC9, HDAC9, HDAC9, HDAC9, HDAC9, HNRNPA2B1, HNRNPA2B1, HNRNPA2B1, HNRNPA2B1, HNRNPA2B1, HNRNPA2B1, HNRNPA2B1, HNRNPA2B1, HNRNPA2B1, HNRNPA2B1, HNRNPA2B1, HNRNPA2B1, HNRNPA2B1, HNRNPA2B1, HNRNPA2B1, HNRNPA2B1, OCLN, OCLN, OCLN, OCLN, OCLN, OCLN, REST, REST, REST, REST, REST, REST, REST, REST, REST, REST, REST, REST, REST, REST, SNX13, SNX13, TTLL7, TTLL7, TTLL7	9
hsa-miR-485-5p	ELP2, ELP2, ELP2, GPRC5A, IGFBP3, IGFBP3, NFATC4, RBM23, RBM23, RBM23, RBM23, RBM23, RBM23, RRM2, RRM2, RRM2, RRM2, RRM2, RRM2, RRM2, RRM2, TXNDC5, VANGL1, VANGL1, VANGL1, VANGL1, WWC3, WWC3, WWC3	9
hsa-miR-493-5p	CELF2, CELF2, CELF2, CELF2, CELF2, CELF2, CELF2, CELF2, CELF2, CELF2, CELF2, CELF2, CELF2, CELF2, CELF2, CLSTN1, CLSTN1, CLSTN1, DIP2C, DIP2C, FUBP1, FUBP1, FUBP1, FUBP1, MEF2C, OTUB1, OTUB1, OTUB1, OTUB1, OTUB1, OTUB1, PMP22, SRPX, SRPX, SRPX, SRPX, TRAM2	9

hsa-miR-495-3p	CELF2, DNAAF5, DNAAF5, GPRC5A, HDAC9, HDAC9, LGALSL, LGALSL, LGALSL, LGALSL, MAST4, SNX13, SNX13, UBE3A, WIPI2, WIPI2, WIPI2, WIPI2, WIPI2, WIPI2, WIPI2, WIPI2, WIPI2	9
hsa-miR-512-3p	CELF2, CELF2, CRAMP1, CRAMP1, CRAMP1, CRAMP1, CRAMP1, CRAMP1, CRAMP1, CRAMP1, CRAMP1, CRAMP1, EDA2R, EDA2R, EDA2R, KLHL28, KLHL28, LETMD1, PRKAR2A, PRKAR2A, PRKAR2A, PRKAR2A, PRKAR2A, VANGL1, VANGL1, VANGL1, VANGL1, YPEL5, YPEL5, YPEL5, YPEL5, ZNF12, ZNF12, ZNF12, ZNF12	9
hsa-miR-520a-3p	BAMBI, CELF2, DYRK4, DYRK4, DYRK4, KLHL28, KLHL28, LETMD1, LETMD1, LETMD1, LETMD1, LETMD1, LETMD1, LETMD1, LETMD1, MEF2C, MEF2C, RAPGEFL1, RAPGEFL1, RAPGEFL1, REST, REST, REST, REST, REST, REST, REST, UBE3A, UBE3A, UBE3A, UBE3A, UBE3A, UBE3A, UBE3A	9
hsa-miR-520c-3p	BAMBI, CELF2, DYRK4, DYRK4, DYRK4, KLHL28, KLHL28, LETMD1, LETMD1, LETMD1, LETMD1, LETMD1, LETMD1, LETMD1, MEF2C, RAPGEFL1, RAPGEFL1, RAPGEFL1, REST, REST, REST, REST, REST, REST, REST, UBE3A, UBE3A, UBE3A, UBE3A, UBE3A, UBE3A, UBE3A	9
hsa-miR-93-5p	ANKRD28, ANKRD28, ARAP2, BAMBI, CELF2, CRAMP1, CRAMP1, CRAMP1, CRAMP1, RAPGEFL1, RAPGEFL1, RAPGEFL1, REST, REST, REST, REST, REST, REST, REST, REST, REST, REST, REST, REST, REST, REST, RRM2, RRM2, RRM2, RRM2, VANGL1, VANGL1, VANGL1, VANGL1, VANGL1, VANGL1, VANGL1, VANGL1, VANGL1, VANGL1, VANGL1, VANGL1, VANGL1, VANGL1, VANGL1, VANGL1	9
hsa-miR-15a-5p	CLCN4, CLCN4, COPS7A, COPS7A, COPS7A, COPS7A, COPS7A, COPS7A, COPS7A, CPD, CPD, CPD, CPD, DMRT2, DMRT2, DMRT2, FLNB, FLNB, FLNB, PRKAR2A, PRKAR2A, PRKAR2A, PRKAR2A, PRKAR2A, RAPGEFL1, WIPI2, WIPI2, WIPI2, WIPI2, WIPI2	8
hsa-miR-182-5p	CELF2, CELF2, CELF2, CELF2, CELF2, CELF2, CELF2, CELF2, DENND2C, DENND2C, HDAC9, HDAC9, HDAC9, HDAC9, HDAC9, HDAC9, HDAC9, HDAC9, HDAC9, KIAA1217, KIAA1217, KIAA1217, KIAA1217, KIAA1217, KIAA1217, KIAA1217, KIAA1217, KIAA1217, KIAA1217, KIAA1217, KIAA1217, KIAA1217, KIAA1217, MAST4, MAST4, OPTN, OPTN, OPTN, OPTN, TAF4B, TAF4B, WIPI2, WIPI2, WIPI2, WIPI2, WIPI2, WIPI2, WIPI2, WIPI2, WIPI2, WIPI2, WIPI2, WIPI2	8
hsa-miR-186-5p	ANKRD28, ANKRD28, DIP2C, DIP2C, DIP2C, DIP2C, DIP2C, DIP2C, DIP2C, DIP2C, EIF3M, EIF3M, GPRC5A, GPRC5A, IGFBP3, IGFBP3, IGFBP3, IGFBP3, IGFBP3, IGFBP3, MAST4, MEF2C, MEF2C, MEF2C, MEF2C, MEF2C, MEF2C, MEF2C, MEF2C, MEF2C, MEF2C, MEF2C, MEF2C, VANGL1, VANGL1, VANGL1, VANGL1, VANGL1, VANGL1	8

hsa-miR-195-5p	CLCN4, CLCN4, COPS7A, COPS7A, COPS7A, COPS7A, COPS7A, COPS7A, COPS7A, COPS7A, CPD, CPD, CPD, CPD, DMRT2, DMRT2, DMRT2, FLNB, FLNB, FLNB, PRKAR2A, PRKAR2A, PRKAR2A, PRKAR2A, PRKAR2A, RAPGEFL1, WIPI2, WIPI2, WIPI2, WIPI2, WIPI2	8
hsa-miR-20b-5p	BAMBI, CELF2, CRAMP1, CRAMP1, CRAMP1, CRAMP1, KLHL28, KLHL28, KLHL28, KLHL28, RAPGEFL1, RAPGEFL1, RAPGEFL1, REST, REST, REST, REST, REST, REST, REST, REST, REST, REST, REST, REST, REST, REST, RRM2, RRM2, RRM2, RRM2, VANGL1, VANGL1, VANGL1, VANGL1, VANGL1, VANGL1, VANGL1, VANGL1, VANGL1, VANGL1, VANGL1, VANGL1	8
hsa-miR-27a-3p	BAG2, BAG2, BAG2, BAG2, CELF2, CELF2, CELF2, CELF2, CELF2, CELF2, ERLEC1, ERLEC1, LETMD1, MEF2C, MEF2C, MEF2C, MEF2C, MEF2C, MEF2C, MEF2C, MEF2C, MEF2C, MEF2C, NR1D2, NR1D2, NRG1, NRG1, NRG1, NRG1, NRG1, NRG1, NRG1, VANGL1, VANGL1, VANGL1, VANGL1, VANGL1, VANGL1, VANGL1, VANGL1, VANGL1, VANGL1, VANGL1, VANGL1, VANGL1, VANGL1, VANGL1, VANGL1, VANGL1, VANGL1	8
hsa-miR-29b-3p	CELF2, CELF2, CELF2, CELF2, CELF2, CELF2, CELF2, CELF2, EMP1, KLHL28, NFATC4, NFATC4, NFATC4, NFATC4, NFATC4, NFATC4, NFATC4, NFATC4, NFATC4, NFATC4, NFATC4, NFATC4, NFATC4, NFATC4, NFATC4, RAPGEFL1, RAPGEFL1, REST, REST, REST, REST, REST, REST, REST, TRAM2, VANGL1, VANGL1, VANGL1, VANGL1, VANGL1, VANGL1, VANGL1, VANGL1, VANGL1, VANGL1, VANGL1, VANGL1, VANGL1, VANGL1, VANGL1, VANGL1	8
hsa-miR-302a-3p	BAMBI, CELF2, DYRK4, DYRK4, DYRK4, KLHL28, KLHL28, KLHL28, LETMD1, LETMD1, LETMD1, LETMD1, LETMD1, LETMD1, LETMD1, LETMD1, LETMD1, LETMD1, LETMD1, MEF2C, MEF2C, RAPGEFL1, RAPGEFL1, RAPGEFL1, UBE3A	8
hsa-miR-302c-3p	BAMBI, CELF2, DYRK4, DYRK4, DYRK4, LETMD1, LETMD1, LETMD1, LETMD1, LETMD1, LETMD1, LETMD1, LETMD1, LETMD1, LETMD1, LETMD1, MEF2C, MEF2C, MEF2C, MEF2C, MEF2C, MEF2C, MEF2C, MEF2C, MEF2C, MEF2C, MEF2C, MEF2C, MEF2C, MEF2C, MEF2C, MEF2C, MEF2C, RAPGEFL1, RAPGEFL1, RAPGEFL1, REST, REST, REST, REST, REST, REST, REST, UBE3A, UBE3A, UBE3A, UBE3A, UBE3A, UBE3A, UBE3A, UBE3A, UBE3A, UBE3A, UBE3A, UBE3A, UBE3A, UBE3A	8
hsa-miR-302e	BAMBI, CELF2, DYRK4, DYRK4, DYRK4, KLHL28, KLHL28, LETMD1, LETMD1, LETMD1, LETMD1, LETMD1, LETMD1, LETMD1, LETMD1, LETMD1, LETMD1, LETMD1, RAPGEFL1, RAPGEFL1, RAPGEFL1, REST, REST, REST, REST, REST, REST, REST, UBE3A, UBE3A, UBE3A, UBE3A, UBE3A, UBE3A, UBE3A	8
hsa-miR-372-3p	BAMBI, CELF2, DYRK4, DYRK4, DYRK4, LETMD1, LETMD1, LETMD1, LETMD1, LETMD1, LETMD1, LETMD1, LETMD1, MEF2C, MEF2C, MEF2C, MEF2C, MEF2C, MEF2C, MEF2C, MEF2C, MEF2C, MEF2C, MEF2C, MEF2C, MEF2C, MEF2C, MEF2C, MEF2C, MEF2C, RAPGEFL1, RAPGEFL1, RAPGEFL1, REST, REST, REST, REST, REST, REST, REST, REST, REST, REST, REST, REST, REST, REST, VANGL1, VANGL1, VANGL1, VANGL1, VANGL1, VANGL1, VANGL1, VANGL1	8

hsa-miR-524-5p	CENPA, CENPA, DIP2C, DIP2C, KIAA1217, KIAA1217, KIAA1217, KIAA1217, KIAA1217, KIAA1217, KIAA1217, KIAA1217, MAST4, MEF2C, OCLN, OCLN, OCLN, RBM23, RBM23, RBM23, RBM23, RBM23, RBM23, RBM23, RBM23, RBM23, RBM23, RBM23, RBM23, RBM23, RBM23, RBM23, RBM23, RBM23, RBM23, WWC3, WWC3, WWC3, WWC3, WWC3, WWC3	8
hsa-miR-526b-3p	ARAP2, BAMBI, CELF2, CELF2, CELF2, CELF2, KLHL28, KLHL28, RAPGEFL1, RAPGEFL1, RAPGEFL1, REST, RRM2, RRM2, RRM2, RRM2, VANGL1, VANGL1, VANGL1, VANGL1, VANGL1, VANGL1, VANGL1, VANGL1	8
hsa-miR-1252-5p	CELF2, CPD, CPD, CRAMP1, CRAMP1, CRAMP1, CRAMP1, CRAMP1, CRAMP1, CRAMP1, CRAMP1, CRAMP1, CRAMP1, GPRC5A, GPRC5A, GPRC5A, GPRC5A, GPRC5A, GPRC5A, GPRC5A, GPRC5A, GPRC5A, GPRC5A, HNRNPA2B1, KIAA1217, KIAA1217, KIAA1217, KIAA1217, KIAA1217, KIAA1217, KIAA1217, KIAA1217, KIAA1217, N4BP2L2, N4BP2L2, N4BP2L2, N4BP2L2, N4BP2L2, N4BP2L2	7
hsa-miR-128-3p	BAG2, BAG2, BAG2, BAG2, CPD, CPD, ERLEC1, ERLEC1, ERLEC1, ERLEC1, ERLEC1, ERLEC1, ERLEC1, ERLEC1, ERLEC1, ERLEC1, ERLEC1, ERLEC1, ERLEC1, ERLEC1, ERLEC1, LETMD1, LETMD1, LETMD1, LETMD1, NR1D2, TCEANC2, TCEANC2, TCEANC2, VANGL1	7
hsa-miR-1323	CELF2, HNRNPA2B1, MEF2C, MEF2C, MEF2C, PARP11, PARP11, PARP11, SLC4A4, SLC4A4, SLC4A4, SLC4A4, SLC4A4, SLC4A4, SLC4A4, SLC4A4, VANGL1, VANGL1, VANGL1, VANGL1, VANGL1, VANGL1, VANGL1, VANGL1, VANGL1, VANGL1, VANGL1, VANGL1, VANGL1, VANGL1, VANGL1, VANGL1, VANGL1, VANGL1, YPEL5, YPEL5, YPEL5, YPEL5	7
hsa-miR-135a-5p	CPD, CPD, CPD, CRAMP1, CRAMP1, EMP1, KLHL28, MEF2C, MEF2C, MEF2C, MEF2C, MEF2C, MEF2C, MEF2C, MEF2C, MEF2C, MEF2C, MEF2C, MEF2C, MEF2C, MEF2C, MEF2C, MEF2C, MEF2C, MEF2C, MEF2C, RBM23, ZNF430, ZNF430	7

hsa-miR-144-3p	ANKRD28, ANKRD28, CELF2, HDAC9, HDAC9, HDAC9, HDAC9, HDAC9, HIVEP3, HIVEP3, HIVEP3, HIVEP3, HIVEP3, HIVEP3, KIAA1217, KIAA1217, KIAA1217, KIAA1217, KIAA1217, KIAA1217, KIAA1217, SLC4A4, SLC4A4, SLC4A4, SLC4A4, SLC4A4, SLC4A4, VANGL1	7
hsa-miR-15b-5p	COPS7A, COPS7A, COPS7A, COPS7A, COPS7A, COPS7A, COPS7A, CPD, CPD, CPD, CPD, CPD, CPD, DMRT2, DMRT2, DMRT2, DMRT2, FLNB, FLNB, FLNB, PRKAR2A, PRKAR2A, PRKAR2A, PRKAR2A, PRKAR2A, RAPGEFL1, WIPI2, WIPI2, WIPI2, WIPI2, WIPI2	7
hsa-miR-16-5p	COPS7A, COPS7A, COPS7A, COPS7A, COPS7A, COPS7A, COPS7A, COPS7A, CPD, CPD, CPD, CPD, DMRT2, DMRT2, DMRT2, DMRT2, FLNB, FLNB, FLNB, PRKAR2A, PRKAR2A, PRKAR2A, PRKAR2A, PRKAR2A, RAPGEFL1, WIPI2, WIPI2, WIPI2, WIPI2, WIPI2	7
hsa-miR-17-5p	BAMBI, CELF2, CRAMP1, CRAMP1, CRAMP1, CRAMP1, CRAMP1, CRAMP1, RAPGEFL1, RAPGEFL1, RAPGEFL1, REST, REST, REST, REST, REST, REST, REST, REST, REST, REST, REST, REST, REST, REST, RRM2, RRM2, RRM2, RRM2, VANGL1, VANGL1, VANGL1, VANGL1, VANGL1, VANGL1, VANGL1, VANGL1	7
hsa-miR-185-5p	CELF2, CELF2, CELF2, CELF2, CELF2, CELF2, CELF2, CELF2, CELF2, CELF2, CELF2, CELF2, CELF2, CELF2, CELF2, CELF2, CELF2, CLSTN1, CLSTN1, CLSTN1, CRAMP1, EDA2R, EDA2R, EDA2R, EDA2R, EDA2R, EDA2R, MAPK13, MAPK13, MAPK13, MAPK13, MAPK13, MAPK13, MAPK13, STAT5B, STAT5B, STAT5B, VANGL1	7
hsa-miR-19a-3p	BAMBI, CPD, CPD, CPD, CPD, FAM234A, FAM234A, KIAA1217, KIAA1217, KIAA1217, KIAA1217, KIAA1217, KIAA1217, KIAA1217, LGALSL, LGALSL, LGALSL, LGALSL, RAPGEFL1, RAPGEFL1, RAPGEFL1, REST, REST, REST, REST, REST, REST, REST	7
hsa-miR-19b-3p	BAMBI, CPD, CPD, FAM234A, FAM234A, KIAA1217, KIAA1217, KIAA1217, KIAA1217, KIAA1217, KIAA1217, KIAA1217, LGALSL, LGALSL, LGALSL, LGALSL, RAPGEFL1, RAPGEFL1, RAPGEFL1, REST, REST, REST, REST, REST, REST, REST	7
hsa-miR-29a-3p	CELF2, CELF2, CELF2, CELF2, CELF2, CELF2, CELF2, CELF2, EMP1, NFATC4, NFATC4, NFATC4, NFATC4, NFATC4, NFATC4, NFATC4, NFATC4, NFATC4, NFATC4, NFATC4, NFATC4, NFATC4, NFATC4, RAPGEFL1, RAPGEFL1, REST, REST, REST, REST, REST, REST, REST, REST, REST, REST, REST, REST, REST, REST, TRAM2, VANGL1, VANGL1, VANGL1, VANGL1, VANGL1, VANGL1, VANGL1, VANGL1, VANGL1, VANGL1, VANGL1, VANGL1, VANGL1, VANGL1, VANGL1, VANGL1	7

hsa-miR-302b-3p	BAMBI, CELF2, LETMD1, LETMD1, LETMD1, LETMD1, LETMD1, LETMD1, LETMD1, LETMD1, LETMD1, LETMD1, LETMD1, MEF2C, MEF2C, MEF2C, MEF2C, MEF2C, MEF2C, MEF2C, MEF2C, MEF2C, MEF2C, MEF2C, MEF2C, MEF2C, MEF2C, MEF2C, MEF2C, MEF2C, MEF2C, RAPGEFL1, RAPGEFL1, RAPGEFL1, REST, REST, REST, REST, REST, REST, REST, UBE3A	7
hsa-miR-302d-3p	BAMBI, CELF2, DYRK4, LETMD1, LETMD1, LETMD1, LETMD1, LETMD1, LETMD1, LETMD1, LETMD1, LETMD1, LETMD1, LETMD1, MEF2C, MEF2C, MEF2C, MEF2C, MEF2C, MEF2C, MEF2C, MEF2C, MEF2C, MEF2C, MEF2C, MEF2C, MEF2C, MEF2C, MEF2C, MEF2C, MEF2C, RAPGEFL1, RAPGEFL1, RAPGEFL1, UBE3A, UBE3A, UBE3A, UBE3A, UBE3A, UBE3A, UBE3A, UBE3A, UBE3A, UBE3A, UBE3A, UBE3A, UBE3A, UBE3A	7
hsa-miR-30a-5p	CELF2, CELF2, ERLEC1, ERLEC1, ERLEC1, ERLEC1, HDAC9, KLHL28, KLHL28, KLHL28, MAST4, MAST4, TAF4B, TAF4B, TXNDC5, TXNDC5	7
hsa-miR-373-3p	CELF2, DYRK4, DYRK4, DYRK4, KLHL28, KLHL28, MEF2C, RAPGEFL1, RAPGEFL1, RAPGEFL1, REST, REST, REST, REST, REST, REST, REST, UBE3A, UBE3A, UBE3A, UBE3A, UBE3A, UBE3A, UBE3A, UBE3A, UBE3A, UBE3A, UBE3A, UBE3A, UBE3A, UBE3A	7
hsa-miR-381-3p	ANKRD28, ANKRD28, CPD, CPD, CPD, CPD, CPD, CPD, MEF2C, MEF2C, MEF2C, MEF2C, MEF2C, PMP22, PMP22, RAPGEFL1, RAPGEFL1, TAF4B, ZNF12, ZNF12, ZNF12, ZNF12, ZNF12, ZNF12, ZNF12, ZNF12, ZNF12, ZNF12, ZNF12, ZNF12	7
hsa-miR-448	COPS7A, COPS7A, COPS7A, COPS7A, COPS7A, COPS7A, COPS7A, MAST4, MAST4, MEF2C, N4BP2L2, REST, TMEM171, TMEM171, TTLL7, TTLL7	7
hsa-miR-519d-3p	BAMBI, CRAMP1, CRAMP1, CRAMP1, CRAMP1, RAPGEFL1, RAPGEFL1, RAPGEFL1, RAPGEFL1, RAPGEFL1, RAPGEFL1, REST, RHOBTB1, RHOBTB1, RHOBTB1, RHOBTB1, RHOBTB1, RHOBTB1, RRM2, RRM2, RRM2, RRM2, VANGL1, VANGL1, VANGL1, VANGL1, VANGL1, VANGL1	7
hsa-miR-520d-3p	BAMBI, CELF2, DYRK4, DYRK4, DYRK4, MEF2C, MEF2C, RAPGEFL1, RAPGEFL1, RAPGEFL1, REST, REST, REST, REST, REST, REST, REST, UBE3A	7
hsa-miR-520d-5p	CENPA, CENPA, KIAA1217, KIAA1217, KIAA1217, KIAA1217, KIAA1217, KIAA1217, KIAA1217, KIAA1217, MAST4, MEF2C, MEF2C, MEF2C, OCLN, OCLN, OCLN, RBM23, RBM23, RBM23, RBM23, RBM23, RBM23, RBM23, RBM23, WWC3, WWC3, WWC3	7
hsa-miR-579-3p	ANKRD28, ANKRD28, ANKRD28, ANKRD28, ANKRD28, ANKRD28, ANKRD28, ANKRD28, CPD, CPD, CPD, CPD, CPD, CPD, CPD, CPD, FUBP1, FUBP1, HDAC9, HDAC9, HDAC9, HDAC9, HDAC9, RHOBTB1, RHOBTB1, RHOBTB1, RHOBTB1, SNX13, SNX13, UBE3A, UBE3A, UBE3A, UBE3A, UBE3A, UBE3A, UBE3A, UBE3A, UBE3A, UBE3A, UBE3A, UBE3A, UBE3A, UBE3A, UBE3A, UBE3A, UBE3A	7

hsa-miR-9-5p	KIAA1217, KIAA1217, KIAA1217, KIAA1217, KIAA1217, KIAA1217, KIAA1217, LGALSL, LGALSL, PMP22, REST, REST, REST, REST, REST, REST, REST, REST, REST, REST, REST, REST, REST, REST, SNX7, SNX7, UBE3A, UBE3A, UBE3A, UBE3A, UBE3A, UBE3A, UBE3A, VANGL1, VANGL1, VANGL1, VANGL1, VANGL1, VANGL1, VANGL1, VANGL1, VANGL1, VANGL1, VANGL1, VANGL1, VANGL1, VANGL1, VANGL1, VANGL1, VANGL1, VANGL1	7
hsa-miR-96-5p	CELF2, CELF2, CELF2, CELF2, CELF2, DENND2C, DENND2C, DENND2C, GPRC5A, GPRC5A, HDAC9, HDAC9, HDAC9, HDAC9, HDAC9, KIAA1217, KIAA1217, SNX7, SNX7, WIPI2, WIPI2, WIPI2, WIPI2, WIPI2, WIPI2	7
hsa-miR-124-3p	CPD, CPD, CPD, CPD, CPD, CPD, FAM234A, FAM234A, TRAM2, TRAM2, TRAM2, UBE3A, VANGL1, VANGL1, VANGL1, VANGL1, VANGL1, VANGL1, VANGL1, VANGL1, VANGL1, VANGL1, WIPI2	6
hsa-miR-181b-5p	BAG2, CPD, CPD, CPD, CPD, DIP2C, DIP2C, KCNQ5, KCNQ5, KCNQ5, KCNQ5, KCNQ5, KCNQ5, KCNQ5, KCNQ5, KCNQ5, KCNQ5, KCNQ5, KCNQ5, KCNQ5, KCNQ5, LGALSL, LGALSL, NR1D2	6
hsa-miR-200a-3p	BAG2, DNAAF5, DNAAF5, DNAAF5, DNAAF5, DNAAF5, DNAAF5, LGALSL, LGALSL, MAST4, MAST4, MAST4, UBE3A, YPEL5, YPEL5, YPEL5, YPEL5, YPEL5, YPEL5, YPEL5, YPEL5, YPEL5, YPEL5, YPEL5, YPEL5, YPEL5, YPEL5, YPEL5, YPEL5, YPEL5, YPEL5	6
hsa-miR-29c-3p	CELF2, CELF2, CELF2, CELF2, CELF2, CELF2, CELF2, CELF2, EMP1, EMP1, NFATC4, NFATC4, NFATC4, NFATC4, NFATC4, NFATC4, NFATC4, NFATC4, NFATC4, NFATC4, NFATC4, NFATC4, NFATC4, NFATC4, RAPGEFL1, RAPGEFL1, REST, REST, REST, REST, REST, REST, REST, VANGL1, VANGL1, VANGL1, VANGL1, VANGL1, VANGL1, VANGL1, VANGL1, VANGL1, VANGL1, VANGL1, VANGL1, VANGL1, VANGL1, VANGL1, VANGL1	6
hsa-miR-377-3p	ANKRD28, ANKRD28, CELF2, CELF2, CELF2, CELF2, CELF2, CELF2, CELF2, HNRNPA2B1, OCLN, OCLN, OCLN, OCLN, OCLN, OCLN, OCLN, OCLN, OCLN, OCLN, OCLN, OCLN, SNX13, SNX13, TTLL7	6
hsa-miR-520a-5p	CELF2, CELF2, CELF2, CELF2, CELF2, CELF2, CELF2, CELF2, CELF2, CELF2, DHFR, DHFR, DHFR, DHFR, NFATC4, NFATC4, NFATC4, NFATC4, NFATC4, NFATC4, NFATC4, NFATC4, NFATC4, NFATC4, NFATC4, NFATC4, NFATC4, NFATC4, NFATC4, RAPGEFL1, RAPGEFL1, RAPGEFL1, RAPGEFL1, SNX7, SNX7, ZFP36L2	6
hsa-miR-520f-3p	CELF2, CPD, HNRNPA2B1, HNRNPA2B1, HNRNPA2B1, HNRNPA2B1, LETMD1, LETMD1, LETMD1, LETMD1, LETMD1, LETMD1, LETMD1, LETMD1, LETMD1, RAPGEFL1, RAPGEFL1, RAPGEFL1, SCARB2, SCARB2, SCARB2, SCARB2, SCARB2, SCARB2, SCARB2, SCARB2, SCARB2, SCARB2	6
hsa-miR-582-5p	CELF2, CELF2, CELF2, CELF2, CELF2, CELF2, CELF2, CELF2, CELF2, CELF2, CPD, CPD, CPD, CPD, CPD, CPD, SLC4A4, SLC4A4, SLC4A4, UBE3A, VANGL1, VANGL1, VANGL1, VANGL1, VANGL1, VANGL1, VANGL1, VANGL1, ZFP36L2, ZFP36L2	6
hsa-miR-654-5p	BMP2, BMP2, DNAAF5, DNAAF5, DNAAF5, FAM234A, FLNB, FLNB, FLNB, PALM, VANGL1, VANGL1, VANGL1, VANGL1	6
hsa-let-7e-5p	CPD, CPD, RNF170, RNF170, RRM2, RRM2, RRM2, RRM2, RRM2, RRM2, RRM2, RRM2, RRM2, RRM2, RRM2, RRM2, SLC4A4, SLC4A4, SLC4A4, WIPI2	5
hsa-miR-103a-3p	COPS7A, COPS7A, COPS7A, COPS7A, COPS7A, COPS7A, COPS7A, COPS7A, CPD, HERC2, PRKAR2A, RAPGEFL1, RAPGEFL1, RAPGEFL1, RAPGEFL1, RAPGEFL1, RAPGEFL1, RAPGEFL1, RAPGEFL1, RAPGEFL1, RAPGEFL1, RAPGEFL1, RAPGEFL1, RAPGEFL1, RAPGEFL1, RAPGEFL1	5

hsa-miR-105-5p	CELF2, CELF2, CELF2, CELF2, CELF2, CELF2, CELF2, CELF2, CELF2, CELF2, MEF2C, MEF2C, MEF2C, MEF2C, MEF2C, MEF2C, MEF2C, MEF2C, MEF2C, MEF2C, MEF2C, MEF2C, REST, RHOBTB1, RHOBTB1, RHOBTB1, RHOBTB1, YPEL5, YPEL5, YPEL5, YPEL5, YPEL5, YPEL5, YPEL5, YPEL5	5
hsa-miR-107	COPS7A, COPS7A, COPS7A, COPS7A, COPS7A, COPS7A, COPS7A, COPS7A, CPD, HERC2, PRKAR2A, RAPGEFL1, RAPGEFL1, RAPGEFL1, RAPGEFL1, RAPGEFL1, RAPGEFL1, RAPGEFL1, RAPGEFL1, RAPGEFL1, RAPGEFL1, RAPGEFL1, RAPGEFL1, RAPGEFL1, RAPGEFL1, RAPGEFL1	5
hsa-miR-1271-5p	CELF2, CELF2, CELF2, CELF2, CELF2, HDAC9, HDAC9, HDAC9, HDAC9, HDAC9, KIAA1217, KIAA1217, KIAA1217, KIAA1217, KIAA1217, KIAA1217, KIAA1217, KIAA1217, KIAA1217, SNX7, SNX7, WIPI2, WIPI2, WIPI2, WIPI2, WIPI2, WIPI2	5
hsa-miR-1286	ICAM1, ICAM1, LETMD1, LETMD1, LETMD1, LETMD1, LETMD1, LETMD1, LETMD1, LETMD1, RAPGEFL1, RAPGEFL1, RAPGEFL1, RAPGEFL1, RAPGEFL1, RAPGEFL1, RAPGEFL1, RAPGEFL1, RAPGEFL1, RAPGEFL1, RAPGEFL1, RAPGEFL1, RAPGEFL1, RAPGEFL1, RAPGEFL1, VANGL1, VANGL1, VANGL1, VANGL1, VANGL1, VANGL1, VANGL1, VANGL1, VANGL1, VANGL1, VANGL1, VANGL1, VANGL1, VANGL1, VANGL1, VANGL1, VANGL1, VANGL1, WIPI2	5
hsa-miR-130a-5p	CELF2, CELF2, HNRNPA2B1, MEF2C, SLC4A4, SLC4A4, SLC4A4, SLC4A4, SLC4A4, SLC4A4, SLC4A4, SLC4A4, SLC4A4, STAT5B, STAT5B	5
hsa-miR-132-3p	FKBP2, FKBP2, FKBP2, LGALSL, LGALSL, LGALSL, LGALSL, LGALSL, LGALSL, LGALSL, LGALSL, MEF2C, SNX13, TAF4B	5
hsa-miR-135b-5p	CPD, CPD, CPD, CPD, CPD, CPD, CPD, CPD, CPD, EMP1, MEF2C, RBM23, ZNF430	5
hsa-miR-140-5p	BAG2, BAG2, BMP2, CELF2, ERLEC1, ERLEC1, ERLEC1, REST	5
hsa-miR-141-3p	BAG2, BAG2, DNAAF5, DNAAF5, DNAAF5, DNAAF5, LGALSL, LGALSL, UBE3A, YPEL5, YPEL5, YPEL5, YPEL5, YPEL5, YPEL5, YPEL5, YPEL5, YPEL5, YPEL5, YPEL5, YPEL5, YPEL5, YPEL5, YPEL5, YPEL5, YPEL5, YPEL5	5
hsa-miR-145-5p	ACTB, FLNB, FLNB, FLNB, FLNB, FLNB, FLNB, GPRC5A, GPRC5A, GPRC5A, GPRC5A, GPRC5A, GPRC5A, GPRC5A, KLHL28, KLHL28, KLHL28, KLHL28, KLHL28, KLHL28, KLHL28, KLHL28, PMP22	5
hsa-miR-148a-3p	CPD, EMP1, EMP1, EMP1, EMP1, FAM234A, FAM234A, FAM234A, FAM234A, FAM234A, FAM234A, FAM234A, FAM234A, KIAA1217, KIAA1217, KIAA1217, KIAA1217, KIAA1217, KIAA1217, KIAA1217, KIAA1217, TAF4B, TAF4B	5

hsa-miR-181a-5p	BAG2, BAG2, CPD, CPD, CPD, CPD, DIP2C, DIP2C, KCNQ5, KCNQ5, KCNQ5, KCNQ5, KCNQ5, KCNQ5, KCNQ5, NR1D2	5
hsa-miR-181c-5p	BAG2, CPD, CPD, CPD, CPD, DIP2C, DIP2C, DIP2C, DIP2C, KCNQ5, KCNQ5, KCNQ5, KCNQ5, KCNQ5, KCNQ5, KCNQ5, NR1D2	5
hsa-miR-212-3p	HDAC9, HDAC9, LGALSL, LGALSL, LGALSL, LGALSL, LGALSL, LGALSL, LGALSL, LGALSL, LGALSL, LGALSL, LGALSL, LGALSL, MEF2C, SNX13, TAF4B	5
hsa-miR-214-3p	CPD, CPD, CPD, CPD, DENND2C, DENND2C, DENND2C, NTN1, NTN1, NTN1, NTN1, NTN1, RAPGEFL1, RAPGEFL1, RAPGEFL1, RAPGEFL1, RAPGEFL1, RAPGEFL1, RAPGEFL1, RAPGEFL1, RAPGEFL1, VANGL1	5
hsa-miR-22-3p	CYB5D1, CYB5D1, CYB5D1, PALM, PALM, PALM, PALM, PALM, PALM, PALM, PALM, RAPGEFL1, RAPGEFL1, RAPGEFL1, RAPGEFL1, RAPGEFL1, RAPGEFL1, RBM23, RBM23, RBM23, RBM23, RBM23, RBM23, ZFP36L2	5
hsa-miR-23c	CELF2, CELF2, CELF2, CELF2, CELF2, CELF2, CELF2, CELF2, CELF2, CELF2, CELF2, CELF2, CELF2, CELF2, CELF2, CELF2, CELF2, CPD, CPD, MEF2C, MEF2C, MEF2C, MEF2C, MEF2C, MEF2C, MEF2C, MEF2C, MEF2C, MEF2C, MEF2C, MEF2C, OTUB1, OTUB1, SLC4A4, SLC4A4, SLC4A4, SLC4A4	5
hsa-miR-26a-5p	KLHL28, MEF2C, REST, REST, REST, REST, REST, REST, REST, UBE3A, ZFP36L2	5
hsa-miR-299-5p	EMP1, EMP1, EMP1, EMP1, EMP1, EMP1, KIAA1217, KIAA1217, KIAA1217, KIAA1217, KIAA1217, KIAA1217, KIAA1217, LHFPL2, LHFPL2, LHFPL2, LHFPL2, LHFPL2, LHFPL2, LHFPL2, LHFPL2, REST, REST, REST, REST, REST, REST, REST, REST, REST, VANGL1, VANGL1, VANGL1, VANGL1	5
hsa-miR-30d-5p	CELF2, CELF2, HDAC9, MAST4, MAST4, TXNDC5, YPEL5, YPEL5, YPEL5, YPEL5, YPEL5	5
hsa-miR-32-5p	CELF2, CELF2, CELF2, CELF2, CELF2, CELF2, CELF2, CELF2, CELF2, FHL2, FHL2, FHL2, FHL2, MAST4, REST, REST, REST, REST, REST, REST, REST, REST, REST, REST, REST, REST, REST, REST, REST, TRAM2	5
hsa-miR-330-5p	GPRC5A, GPRC5A, GPRC5A, GPRC5A, NTN1, NTN1, NTN1, NTN1, NTN1, NTN1, NTN1, NTN1, NTN1, NTN1, NTN1, NTN1, PALM, STAT5B, STAT5B, STAT5B, STAT5B, TCEANC2, TCEANC2, TCEANC2, TCEANC2, TCEANC2, TCEANC2, TCEANC2, TCEANC2	5
hsa-miR-34c-5p	CPD, KIAA1217, KIAA1217, KIAA1217, KIAA1217, KIAA1217, KIAA1217, KIAA1217, SNX13, SNX13, TAF4B, TAF4B, WWC3, WWC3, WWC3, WWC3, WWC3, WWC3	5
hsa-miR-3681-3p	BAG2, BAG2, CPD, CPD, NR1D2, TCEANC2, TCEANC2, VANGL1, VANGL1, VANGL1, VANGL1, VANGL1, VANGL1, VANGL1, VANGL1, VANGL1, VANGL1, VANGL1, VANGL1, VANGL1, VANGL1, VANGL1, VANGL1, VANGL1, VANGL1	5
hsa-miR-506-3p	CPD, CPD, CPD, CPD, CPD, CPD, FAM234A, FAM234A, HNRNPA2B1, HNRNPA2B1, HNRNPA2B1, HNRNPA2B1, HNRNPA2B1, HNRNPA2B1, HNRNPA2B1, HNRNPA2B1, HNRNPA2B1, HNRNPA2B1, HNRNPA2B1, HNRNPA2B1, SNX13, VANGL1	5
hsa-miR-515-5p	FLNB, FLNB, FLNB, FLNB, FLNB, FLNB, NR1D2, NR1D2, NR1D2, SNX13, SNX13, SNX13, SNX13, ZFAND4, ZFAND4, ZFAND4, ZNF430	5
hsa-miR-532-3p	CAVIN1, CAVIN1, CAVIN1, CAVIN1, CAVIN1, EMP1, EMP1, EMP1, EMP1, LETMD1, LETMD1, LETMD1, LETMD1, LETMD1, LETMD1, LETMD1, LETMD1, RAPGEFL1, RAPGEFL1, RAPGEFL1, RAPGEFL1, RAPGEFL1, RRM2, RRM2, RRM2, RRM2, RRM2, RRM2, RRM2, RRM2	5
hsa-miR-541-3p	BMP2, BMP2, DNAAF5, DNAAF5, DNAAF5, FAM234A, FLNB, FLNB, FLNB, PALM	5

hsa-miR-543	CELF2, CPD, CPD, N4BP2L2, N4BP2L2, N4BP2L2, N4BP2L2, N4BP2L2, N4BP2L2, NR1D2, NR1D2, VANGL1, VANGL1, VANGL1, VANGL1, VANGL1, VANGL1, VANGL1, VANGL1	5
hsa-miR-671-5p	CYB5D1, GPRC5A, HIVEP3, HIVEP3, HIVEP3, LGALSL, LGALSL, LGALSL, LGALSL, LGALSL, LGALSL, TRAM2, TRAM2, TRAM2, TRAM2, TRAM2, TRAM2, TRAM2, TRAM2, TRAM2, TRAM2, TRAM2, TRAM2	5
hsa-let-7b-5p	CPD, RNF170, RNF170, RRM2, RRM2, RRM2, RRM2, SLC4A4, SLC4A4, SLC4A4	4
hsa-let-7f-5p	CPD, CPD, RNF170, RNF170, RRM2, RRM2, RRM2, RRM2, WIPI2, WIPI2	4
hsa-let-7g-5p	ANKRD28, ANKRD28, CPD, CPD, CPD, RRM2, RRM2, RRM2, RRM2, WIPI2	4
hsa-miR-125a-5p	CYB5D1, CYB5D1, RAPGEFL1, RAPGEFL1, REST, REST, REST, REST, REST, REST, REST, REST, REST, REST, ZNF12, ZNF12	4
hsa-miR-125b-5p	CYB5D1, CYB5D1, CYB5D1, CYB5D1, RAPGEFL1, RAPGEFL1, REST, REST, REST, REST, REST, REST, REST, ZNF12, ZNF12	4
hsa-miR-129-5p	CELF2, CELF2, CELF2, CELF2, CELF2, CELF2, CELF2, CELF2, CELF2, CELF2, EMP1, EMP1, EMP1, EMP1, EMP1, EMP1, EMP1, EMP1, EMP1, EMP1, EMP1, EMP1, HDAC9, HDAC9, HDAC9, UBE3A	4
hsa-miR-1301-3p	CELF2, CELF2, CELF2, CELF2, CELF2, DIP2C, DIP2C, HNRNPA2B1, HNRNPA2B1, NDUFB10, NDUFB10	4
hsa-miR-130a-3p	FAM234A, FAM234A, FAM234A, FAM234A, LGALSL, LGALSL, LGALSL, LGALSL, TAF4B, TAF4B, VANGL1, VANGL1, VANGL1, VANGL1	4
hsa-miR-130b-3p	DIP2C, DIP2C, FAM234A, FAM234A, FAM234A, FAM234A, LGALSL, LGALSL, LGALSL, LGALSL, TAF4B, TAF4B	4
hsa-miR-1321	HNRNPA2B1, HNRNPA2B1, HNRNPA2B1, HNRNPA2B1, PMP22, RAPGEFL1, RAPGEFL1, RAPGEFL1, RAPGEFL1, RAPGEFL1, RAPGEFL1, RAPGEFL1, RAPGEFL1, RAPGEFL1, RAPGEFL1, RAPGEFL1, RAPGEFL1, RHBDL3, RHBDL3, RHBDL3, RHBDL3	4
hsa-miR-142-5p	ANKRD28, ANKRD28, CPD, CPD, CPD, CPD, FHL2, FHL2, KIAA1217, KIAA1217, KIAA1217, KIAA1217, KIAA1217, KIAA1217, KIAA1217, KIAA1217, KIAA1217, KIAA1217	4
hsa-miR-147a	BAG2, BAG2, BAG2, BAG2, EMP1, EMP1, EMP1, EMP1, EMP1, EMP1, EMP1, EMP1, EMP1, EMP1, EMP1, EMP1, LHFPL2, LHFPL2, SLC26A1, SLC26A1	4
hsa-miR-148b-3p	EMP1, EMP1, EMP1, EMP1, FAM234A, FAM234A, FAM234A, FAM234A, FAM234A, FAM234A, FAM234A, FAM234A, KIAA1217, KIAA1217, KIAA1217, KIAA1217, KIAA1217, KIAA1217, KIAA1217, TAF4B, TAF4B	4
hsa-miR-152-3p	CPD, FAM234A, FAM234A, FAM234A, FAM234A, KIAA1217, KIAA1217, KIAA1217, KIAA1217, KIAA1217, KIAA1217, KIAA1217, TAF4B, TAF4B	4
hsa-miR-153-3p	CELF2, CELF2, CELF2, CELF2, CELF2, CELF2, CELF2, CELF2, CELF2, CELF2, CELF2, CELF2, CELF2, CELF2, MEF2C, MEF2C, MEF2C, MEF2C, MEF2C, MEF2C, MEF2C, MEF2C, MEF2C, MEF2C, MEF2C, MEF2C, MEF2C, MEF2C, MEF2C, MEF2C, MEF2C, MEF2C, MEF2C, REST, REST, REST, REST, REST, REST, REST, REST, REST, REST, SLC4A4, SLC4A4, SLC4A4, SLC4A4, SLC4A4, SLC4A4, SLC4A4, SLC4A4, SLC4A4, SLC4A4, SLC4A4, SLC4A4, SLC4A4	4
hsa-miR-183-5p	CELF2, CELF2, CELF2, CELF2, CELF2, CELF2, CELF2, LHFPL2, LHFPL2, LHFPL2, LHFPL2, VANGL1, VANGL1, VANGL1, VANGL1, VANGL1, VANGL1, VANGL1, VANGL1, YPEL5, YPEL5, YPEL5, YPEL5, YPEL5	4

hsa-miR-194-5p	ANKRD28, ANKRD28, ANKRD28, ANKRD28, KIAA1217, KIAA1217, KIAA1217, KIAA1217, KIAA1217, KIAA1217, KIAA1217, MAST4, MAST4, MAST4, MEF2C, MEF2C, MEF2C, MEF2C, MEF2C	4
hsa-miR-196b-5p	CELF2, CELF2, CELF2, CELF2, CELF2, CELF2, CELF2, CELF2, CELF2, CPD, CPD, CPD, CPD, CPD, CPD, HDAC9, HDAC9, HDAC9, HDAC9, HDAC9, HDAC9, HDAC9, HDAC9, MAST4, MAST4, MAST4, MAST4	4
hsa-miR-199a-5p	GPRC5A, GPRC5A, GPRC5A, GPRC5A, OPTN, OPTN, OPTN, OPTN, RBM23, RBM23, RBM23, RBM23, RBM23, WIPI2, WIPI2, WIPI2, WIPI2, WIPI2, WIPI2	4
hsa-miR-199b-5p	GPRC5A, GPRC5A, GPRC5A, LHFPL2, LHFPL2, LHFPL2, LHFPL2, LHFPL2, LHFPL2, OPTN, OPTN, OPTN, OPTN, WIPI2, WIPI2, WIPI2, WIPI2, WIPI2	4
hsa-miR-200c-3p	FUBP1, FUBP1, NRG1, NRG1, OCLN, OCLN, OCLN, OCLN, OCLN, OCLN, STXBP6, STXBP6	4
hsa-miR-204-5p	ARAP2, CPD, HNRNPA2B1, HNRNPA2B1, HNRNPA2B1, HNRNPA2B1, HNRNPA2B1, HNRNPA2B1, HNRNPA2B1, HNRNPA2B1, HNRNPA2B1, HNRNPA2B1, HNRNPA2B1, HNRNPA2B1, HNRNPA2B1, HNRNPA2B1, WWC3, WWC3	4
hsa-miR-211-5p	ARAP2, CPD, HNRNPA2B1, HNRNPA2B1, HNRNPA2B1, HNRNPA2B1, HNRNPA2B1, HNRNPA2B1, HNRNPA2B1, HNRNPA2B1, HNRNPA2B1, HNRNPA2B1, HNRNPA2B1, HNRNPA2B1, WWC3, WWC3	4
hsa-miR-301b-3p	FAM234A, FAM234A, FAM234A, FAM234A, LGALSL, LGALSL, LGALSL, LGALSL, TAF4B, TAF4B, VANGL1, VANGL1, VANGL1, VANGL1	4
hsa-miR-30c-5p	HDAC9, HDAC9, HDAC9, HDAC9, HDAC9, HDAC9, KLHL28, KLHL28, KLHL28, KLHL28, KLHL28, KLHL28, TAF4B, TAF4B, TXNDC5, TXNDC5	4
hsa-miR-30e-5p	HDAC9, MAST4, MAST4, TAF4B, TAF4B, TXNDC5, TXNDC5	4
hsa-miR-320b	CAVIN1, CAVIN1, CPD, CPD, CPD, CPD, CPD, CPD, CPD, CPD, CPD, CPD, CPD, CPD, CPD, CPD, CPD, NR1D2, RAPGEFL1, RAPGEFL1	4
hsa-miR-320c	CAVIN1, CAVIN1, CPD, CPD, CPD, CPD, CPD, CPD, CPD, CPD, CPD, CPD, CPD, CPD, CPD, CPD, CPD, NR1D2, RAPGEFL1, RAPGEFL1	4
hsa-miR-320d	CAVIN1, CPD, CPD, CPD, CPD, CPD, CPD, CPD, CPD, CPD, CPD, NR1D2, RAPGEFL1, RAPGEFL1	4
hsa-miR-339-5p	CELF2, CELF2, ICAM1, ICAM1, ICAM1, ICAM1, MAST4, MAST4, MAST4, UBE3A	4
hsa-miR-34a-5p	CPD, KIAA1217, KIAA1217, KIAA1217, KIAA1217, KIAA1217, KIAA1217, KIAA1217, LCMT2, MAPK13, MAPK13, MAPK13, MAPK13, MAPK13, MAPK13	4
hsa-miR-362-3p	ELAVL4, ELAVL4, MEF2C, PARP11, PARP11, PARP11, VANGL1, VANGL1, VANGL1, VANGL1, VANGL1, VANGL1	4
hsa-miR-367-3p	CELF2, CELF2, CELF2, CELF2, CELF2, CELF2, CELF2, CELF2, CELF2, CELF2, CELF2, FHL2, FHL2, FHL2, FHL2, MAST4, REST, REST, REST, REST, REST, REST, REST	4
hsa-miR-423-5p	CRAMP1, CRAMP1, CRAMP1, CRAMP1, NFATC4, NFATC4, NFATC4, NFATC4, NFATC4, NFATC4, NFATC4, NFATC4, NFATC4, NFATC4, NFATC4, NFATC4, NFATC4, NFATC4, NFATC4, NFATC4, RAPGEFL1, RAPGEFL1, RAPGEFL1, RAPGEFL1, RAPGEFL1, RAPGEFL1, RAPGEFL1, RAPGEFL1, RAPGEFL1, RAPGEFL1, RAPGEFL1, RAPGEFL1, STAT5B	4
hsa-miR-429	ANKRD28, ANKRD28, FUBP1, FUBP1, FUBP1, FUBP1, NRG1, NRG1, NRG1, NRG1, NRG1, NRG1, NRG1, NRG1, NRG1, NRG1, NRG1, OCLN, OCLN, OCLN	4
hsa-miR-452-5p	ANKRD28, ANKRD28, CELF2, MEF2C, MEF2C, MEF2C, MEF2C, MEF2C, MEF2C, MEF2C, MEF2C, MEF2C, MEF2C, MEF2C, MEF2C, MEF2C, MEF2C, MEF2C, UBE3A	4

hsa-miR-454-3p	ANKRD28, ANKRD28, FAM234A, FAM234A, LGALSL, LGALSL, LGALSL, LGALSL, TAF4B, TAF4B	4
hsa-miR-4731-5p	CRAMP1, CRAMP1, CRAMP1, CRAMP1, DHFR, HIVEP3, HIVEP3, HIVEP3, NFATC4	4
hsa-miR-494-3p	CELF2, CELF2, CELF2, CELF2, CELF2, CELF2, CPD, CPD, CPD, SNX13, SNX13, SNX13, SNX13, SNX13, SNX13, SNX13, SNX13, WWC3	4
hsa-miR-513b-5p	CELF2, HNRNPA2B1, HNRNPA2B1, HNRNPA2B1, HNRNPA2B1, HNRNPA2B1, HNRNPA2B1, HNRNPA2B1, HNRNPA2B1, HNRNPA2B1, HNRNPA2B1, LHFPL2, LHFPL2, WWC3	4
hsa-miR-522-3p	CELF2, CPD, CPD, CPD, CPD, PRKAR2A, SCARB2, SCARB2, SCARB2, SCARB2, SCARB2, SCARB2	4
hsa-miR-525-5p	CELF2, CELF2, CELF2, CELF2, CELF2, CELF2, CELF2, CELF2, CELF2, CELF2, DHFR, DHFR, DHFR, DHFR, NFATC4, NFATC4, NFATC4, NFATC4, NFATC4, NFATC4, NFATC4, NFATC4, NFATC4, NFATC4, NFATC4, NFATC4, NFATC4, NFATC4, NFATC4, NFATC4, RAPGEFL1, RAPGEFL1, RAPGEFL1, RAPGEFL1	4
hsa-miR-548o-3p	CELF2, HNRNPA2B1, HNRNPA2B1, HNRNPA2B1, HNRNPA2B1, SLC4A4, SLC4A4, SLC4A4, SLC4A4, SLC4A4, SLC4A4, VANGL1	4
hsa-miR-5688	CELF2, MAST4, MAST4, VANGL1, VANGL1, WIPI2, WIPI2, WIPI2, WIPI2, WIPI2, WIPI2, WIPI2, WIPI2	4
hsa-miR-708-5p	OTUB1, OTUB1, OTUB1, RAPGEFL1, RBM23, SCARB2, SCARB2, SCARB2, SCARB2, SCARB2, SCARB2	4
hsa-miR-760	COPS7A, HNRNPA2B1, TRAM2, TRAM2, TRAM2, YPEL5, YPEL5, YPEL5, YPEL5, YPEL5, YPEL5, YPEL5, YPEL5, YPEL5, YPEL5	4
hsa-miR-92b-3p	FHL2, FHL2, MAST4, REST, REST, REST, REST, REST, REST, REST, REST, REST, REST, ZNF430, ZNF430, ZNF430, ZNF430	4
hsa-miR-98-5p	CPD, CPD, CPD, RRM2, RRM2, RRM2, RRM2, RRM2, RRM2, RRM2, RRM2, SLC4A4, SLC4A4, SLC4A4, WIPI2	4
hsa-let-7a-5p	CPD, CPD, RRM2, RRM2, RRM2, RRM2, RRM2, RRM2, RRM2, RRM2, SLC4A4, SLC4A4, SLC4A4	3
hsa-let-7c-5p	CPD, CPD, RRM2, RRM2, RRM2, RRM2, RRM2, RRM2, RRM2, RRM2, SLC4A4, SLC4A4, SLC4A4	3
hsa-let-7d-5p	CPD, CPD, RNF170, RNF170, RRM2, RRM2, RRM2, RRM2, RRM2, RRM2, RRM2, RRM2	3
hsa-let-7i-5p	CPD, RRM2, RRM2, RRM2, RRM2, SLC4A4, SLC4A4, SLC4A4	3

hsa-miR-10a-5p	CELF2, CELF2, CELF2, CELF2, LGALSL, LGALSL, TMEM167B, TMEM167B	3
hsa-miR-10b-5p	CELF2, CELF2, CELF2, CELF2, LGALSL, LGALSL, TMEM167B, TMEM167B	3
hsa-miR-1197	CAVIN1, CLSTN1, CLSTN1, CLSTN1, CLSTN1, CLSTN1, CLSTN1, CLSTN1, CLSTN1, CLSTN1, CLSTN1, CLSTN1, CLSTN1, CLSTN1, CLSTN1, CLSTN1, CLSTN1, CLSTN1, KLF8, KLF8	3
hsa-miR-122-5p	DENND2C, DENND2C, OCLN, OCLN, OCLN, ZNF430, ZNF430	3
hsa-miR-1294	CELF2, CELF2, CELF2, CELF2, CELF2, CELF2, CELF2, CELF2, CELF2, CELF2, CELF2, CELF2, CELF2, CELF2, CELF2, CELF2, CELF2, CELF2, RRM2, RRM2, RRM2, RRM2, RRM2, RRM2, RRM2, RRM2, TRAM2	3
hsa-miR-142-3p	CELF2, CELF2, CELF2, CELF2, CELF2, CELF2, CELF2, CELF2, CELF2, CELF2, COPS7A, COPS7A, COPS7A, COPS7A, COPS7A, COPS7A, NR1D2	3
hsa-miR-143-3p	CELF2, CELF2, CELF2, CELF2, CPD, WWC3, WWC3	3
hsa-miR-150-5p	CPD, CPD, NR1D2, NR1D2, RHOBTB1, RHOBTB1	3
hsa-miR-196a-5p	CELF2, CELF2, CELF2, CELF2, CELF2, CELF2, CELF2, CELF2, CELF2, CPD, CPD, CPD, CPD, CPD, CPD, HDAC9, HDAC9, HDAC9, HDAC9, HDAC9, HDAC9, HDAC9, HDAC9, HDAC9, HDAC9	3
hsa-miR-200b-3p	FUBP1, FUBP1, FUBP1, FUBP1, NRG1, NRG1, NRG1, NRG1, NRG1, NRG1, NRG1, NRG1, OCLN, OCLN, OCLN	3
hsa-miR-205-5p	ACTB, ACTB, LHFPL2, LHFPL2, TRAM2	3
hsa-miR-216a-3p	BAG2, BAG2, CPD, CPD, VANGL1, VANGL1, VANGL1, VANGL1, VANGL1, VANGL1, VANGL1, VANGL1, VANGL1, VANGL1, VANGL1, VANGL1, VANGL1, VANGL1, VANGL1, VANGL1	3
hsa-miR-218-5p	MAST4, MAST4, MAST4, SNX13, UBE3A, UBE3A, UBE3A, UBE3A, UBE3A, UBE3A, UBE3A, UBE3A, UBE3A, UBE3A, UBE3A	3
hsa-miR-223-3p	BAG2, BAG2, ERLEC1, ERLEC1, ERLEC1, MEF2C, MEF2C, MEF2C, MEF2C, MEF2C, MEF2C, MEF2C, MEF2C, MEF2C, MEF2C, MEF2C, MEF2C	3
hsa-miR-24-3p	LHFPL2, LHFPL2, LHFPL2, LHFPL2, MRPL4, MRPL4, MRPL4, MRPL4, MRPL4, SNX13, SNX13	3
hsa-miR-25-3p	FHL2, FHL2, MAST4, REST, REST, REST, REST, REST, REST, REST, REST, REST, REST	3
hsa-miR-26b-5p	MEF2C, REST, REST, REST, REST, REST, REST, REST, UBE3A, UBE3A, UBE3A, UBE3A, UBE3A, UBE3A, UBE3A, UBE3A	3
hsa-miR-296-5p	FLNB, FLNB, FLNB, GMPR, ZFP36L2	3
hsa-miR-301a-3p	FAM234A, FAM234A, FAM234A, FAM234A, LGALSL, LGALSL, LGALSL, LGALSL, TAF4B, TAF4B	3
hsa-miR-3064-5p	ELAVL4, ELAVL4, ELAVL4, ELAVL4, ELAVL4, ELAVL4, ELAVL4, ELAVL4, HNRNPA2B1, HNRNPA2B1, HNRNPA2B1, HNRNPA2B1, RRM2, RRM2, RRM2, RRM2	3
hsa-miR-30b-5p	HDAC9, HDAC9, HDAC9, HDAC9, HDAC9, HDAC9, HDAC9, HDAC9, HDAC9, HDAC9, KLHL28, KLHL28, KLHL28, TXNDC5	3
hsa-miR-31-5p	PRKAR2A, RHOBTB1, RHOBTB1, TRAM2, TRAM2, TRAM2, TRAM2	3
hsa-miR-326	NTN1, NTN1, NTN1, NTN1, NTN1, NTN1, NTN1, NTN1, PALM, TCEANC2, TCEANC2, TCEANC2, TCEANC2, TCEANC2, TCEANC2, TCEANC2, TCEANC2, TCEANC2, TCEANC2	3
hsa-miR-329-3p	CELF2, CELF2, CELF2, CELF2, CELF2, MEF2C, VANGL1, VANGL1, VANGL1, VANGL1, VANGL1, VANGL1	3
hsa-miR-330-3p	CPD, CPD, CPD, MEF2C, ZNF726, ZNF726	3

hsa-miR-363-3p	FHL2, FHL2, FHL2, FHL2, MAST4, MAST4, REST, REST, REST	3
hsa-miR-369-3p	CELF2, CELF2, CELF2, CELF2, CELF2, CELF2, CELF2, CELF2, CELF2, CELF2, CTBS, CTBS, TTLL7, TTLL7	3
hsa-miR-370-3p	RAPGEFL1, RAPGEFL1, RAPGEFL1, RAPGEFL1, RAPGEFL1, RAPGEFL1, RAPGEFL1, RAPGEFL1, RAPGEFL1, RAPGEFL1, SLC4A4, SLC4A4, SLC4A4, VANGL1	3
hsa-miR-371a-5p	ARAP2, ARAP2, CELF2, LHFPL2, LHFPL2, LHFPL2, LHFPL2, LHFPL2, LHFPL2	3
hsa-miR-376b-3p	COPS7A, COPS7A, COPS7A, RAPGEFL1, RAPGEFL1, RAPGEFL1, RAPGEFL1, RAPGEFL1, RAPGEFL1, SNX13, SNX13	3
hsa-miR-425-5p	ELAVL4, ELAVL4, ELAVL4, ELAVL4, TFAP2B, WWC3, WWC3, WWC3, WWC3	3
hsa-miR-4458	CPD, RRM2, RRM2, RRM2, RRM2, SLC4A4, SLC4A4, SLC4A4	3
hsa-miR-449a	CPD, KIAA1217, KIAA1217, KIAA1217, KIAA1217, KIAA1217, KIAA1217, KIAA1217, TAF4B, TAF4B	3
hsa-miR-449b-5p	CPD, KIAA1217, KIAA1217, KIAA1217, KIAA1217, KIAA1217, KIAA1217, KIAA1217, TAF4B, TAF4B	3
hsa-miR-4500	CPD, RRM2, RRM2, RRM2, RRM2, SLC4A4, SLC4A4, SLC4A4	3
hsa-miR-486-5p	CELF2, CELF2, CELF2, CELF2, CELF2, CELF2, CELF2, CELF2, CELF2, CELF2, CELF2, CELF2, CELF2, CELF2, CELF2, CELF2, CELF2, CELF2, EMP1, EMP1, EMP1, EMP1, EMP1, EMP1, EMP1, EMP1, VANGL1, VANGL1, VANGL1, VANGL1	3
hsa-miR-488-3p	ANKRD28, ANKRD28, ANKRD28, ANKRD28, ANKRD28, ANKRD28, ERLEC1, ERLEC1, VANGL1, VANGL1, VANGL1, VANGL1	3
hsa-miR-491-5p	DHFR, DHFR, DHFR, DHFR, SLC6A17, SLC6A17, SLC6A17, SLC6A17, VANGL1, VANGL1, VANGL1, VANGL1, VANGL1, VANGL1	3
hsa-miR-499a-5p	CELF2, CELF2, CELF2, CELF2, CELF2, CELF2, CELF2, CELF2, CELF2, CELF2, CELF2, CELF2, CELF2, CELF2, CELF2, CELF2, HNRNPA2B1, HNRNPA2B1, HNRNPA2B1, HNRNPA2B1, HNRNPA2B1, MAST4	3
hsa-miR-5047	CELF2, CELF2, CELF2, CELF2, CELF2, NDUFB10, REST, REST, REST, REST, REST, REST, REST, REST, REST, REST, REST, REST, REST, REST	3
hsa-miR-513a-5p	CELF2, HIVEP3, HIVEP3, HIVEP3, PTGFR	3
hsa-miR-519a-3p	CELF2, UBE3A, VANGL1, VANGL1, VANGL1, VANGL1, VANGL1, VANGL1, VANGL1, VANGL1, VANGL1, VANGL1, VANGL1, VANGL1, VANGL1, VANGL1, VANGL1, VANGL1, VANGL1, VANGL1, VANGL1, VANGL1	3

hsa-miR-519b-3p	CELF2, UBE3A, VANGL1, VANGL1, VANGL1, VANGL1, VANGL1, VANGL1, VANGL1, VANGL1, VANGL1, VANGL1, VANGL1, VANGL1, VANGL1, VANGL1, VANGL1, VANGL1	3
hsa-miR-519c-3p	CELF2, UBE3A, VANGL1, VANGL1, VANGL1, VANGL1, VANGL1, VANGL1, VANGL1, VANGL1, VANGL1, VANGL1, VANGL1, VANGL1, VANGL1, VANGL1, VANGL1, VANGL1	3
hsa-miR-5590-3p	BAMBI, BMP2, VANGL1, VANGL1, VANGL1, VANGL1, VANGL1, VANGL1, VANGL1, VANGL1	3
hsa-miR-580-3p	CPD, CPD, CPD, CPD, CPD, CPD, CPD, CPD, CPD, CPD, CPD, CPD, SNX13, SNX13, SNX13, SNX13, TRAM2, TRAM2, TRAM2, TRAM2	3
hsa-miR-588	ALG1, ALG1, ALG1, FLNB, FLNB, FLNB, LHFPL2, LHFPL2, LHFPL2, LHFPL2	3
hsa-miR-624-3p	HDAC9, HDAC9, HDAC9, HDAC9, HDAC9, HDAC9, HDAC9, HDAC9, HDAC9, KIAA1217, KIAA1217, KIAA1217, KIAA1217, KIAA1217, KIAA1217, KIAA1217, VANGL1, VANGL1, VANGL1, VANGL1	3
hsa-miR-761	CPD, CPD, RAPGEFL1, RAPGEFL1, RAPGEFL1, VANGL1, VANGL1, VANGL1, VANGL1, VANGL1, VANGL1, VANGL1, VANGL1, VANGL1, VANGL1, VANGL1, VANGL1	3
hsa-miR-766-5p	GPRC5A, GPRC5A, GPRC5A, ICAM1, ICAM1, ICAM1, PRKAR2A, PRKAR2A	3
hsa-miR-873-5p	NR1D2, PRKAR2A, PRKAR2A, PRKAR2A, RAPGEFL1	3
hsa-miR-888-5p	CELF2, CELF2, CELF2, CELF2, CELF2, CELF2, CELF2, CELF2, CELF2, CELF2, CELF2, CELF2, SLC4A4, SLC4A4, SLC4A4, TCEANC2, TCEANC2, TCEANC2	3
hsa-miR-92a-3p	FHL2, FHL2, FHL2, FHL2, MAST4, REST, REST, REST, REST, REST, REST, REST, REST, REST, REST	3
hsa-miR-942-5p	BAMBI, CELF2, CELF2, CELF2, CELF2, CELF2, CELF2, CELF2, CELF2, CELF2, CELF2, CELF2, CELF2, CELF2, CELF2, CELF2, CELF2, CELF2, CELF2, CELF2, STRAP, STRAP	3
hsa-miR-1-3p	FUBP1, ZFP36L2, ZFP36L2, ZFP36L2, ZFP36L2, ZFP36L2, ZFP36L2	2
hsa-miR-1224-5p	CELF2, TRAM2	2
hsa-miR-1269a	NR1D2, VANGL1, VANGL1, VANGL1, VANGL1, VANGL1, VANGL1, VANGL1, VANGL1, VANGL1, VANGL1, VANGL1, VANGL1	2
hsa-miR-1276	BMP2, VANGL1, VANGL1, VANGL1, VANGL1, VANGL1, VANGL1, VANGL1, VANGL1, VANGL1, VANGL1, VANGL1, VANGL1, VANGL1, VANGL1, VANGL1, VANGL1	2
hsa-miR-1277-5p	BMP2, BMP2, NTN1	2
hsa-miR-1297	REST, REST, REST, REST, REST, REST, REST, UBE3A, UBE3A, UBE3A, UBE3A, UBE3A, UBE3A, UBE3A, UBE3A, UBE3A, UBE3A	2

hsa-miR-133a-3p	CPD, CPD, TRAM2, TRAM2, TRAM2, TRAM2	2
hsa-miR-133b	CPD, CPD, TRAM2, TRAM2, TRAM2, TRAM2	2
hsa-miR-138-5p	MAST4, RHBDL3, RHBDL3, RHBDL3, RHBDL3, RHBDL3, RHBDL3, RHBDL3, RHBDL3, RHBDL3	2
hsa-miR-139-5p	HIVEP3, HIVEP3, HIVEP3, HIVEP3, HIVEP3, HIVEP3, PRKAR2A	2
hsa-miR-140-3p	CRAMP1, CRAMP1, CRAMP1, CRAMP1, PRKAR2A, PRKAR2A	2
hsa-miR-149-5p	CYB5D1, PRKAR2A, PRKAR2A	2
hsa-miR-193a-3p	CELF2, CELF2, CLSTN1, CLSTN1, CLSTN1	2
hsa-miR-193b-3p	CELF2, CELF2, CLSTN1, CLSTN1, CLSTN1	2
hsa-miR-197-3p	IGFBP3, IGFBP3, IGFBP3, TCEANC2, TCEANC2, TCEANC2, TCEANC2	2
hsa-miR-199a-3p	CELF2, CELF2, CELF2, CELF2, CELF2, VANGL1, VANGL1	2
hsa-miR-199b-3p	CELF2, CELF2, CELF2, CELF2, CELF2, VANGL1, VANGL1	2
hsa-miR-206	FUBP1, ZFP36L2, ZFP36L2, ZFP36L2, ZFP36L2, ZFP36L2, ZFP36L2, ZFP36L2, ZFP36L2	2
hsa-miR-2115-3p	CYB5D1, PMP22	2
hsa-miR-219a-5p	CELF2, CELF2, CELF2, CELF2, CELF2, LGALSL, LGALSL, LGALSL, LGALSL	2
hsa-miR-224-3p	CPD, CPD, SCARB2, SCARB2, SCARB2, SCARB2, SCARB2, SCARB2	2
hsa-miR-224-5p	LGALSL, LGALSL, LGALSL, LGALSL, OCLN, OCLN, OCLN	2
hsa-miR-2467-3p	OTUB1, PALM, PALM, PALM, PALM, PALM, PALM, PALM, PALM, PALM, PALM, PALM, PALM	2
hsa-miR-28-3p	CPD, CPD, HNRNPA2B1, HNRNPA2B1	2
hsa-miR-28-5p	OTUB1, OTUB1, OTUB1, OTUB1, RAPGEFL1	2
hsa-miR-338-3p	LGALSL, LGALSL, SLC4A4, SLC4A4, SLC4A4	2
hsa-miR-33a-5p	CELF2, CELF2, CELF2, CELF2, CELF2, CELF2, CELF2, CELF2, CELF2, CELF2, CELF2, CELF2, CELF2, CELF2, CELF2, CELF2, CELF2, CELF2, NTN1	2
hsa-miR-33b-5p	CELF2, CELF2, CELF2, CELF2, CELF2, NTN1	2
hsa-miR-342-3p	CELF2, CELF2, CELF2, CELF2, RRM2, RRM2, RRM2, RRM2, RRM2, RRM2, RRM2, RRM2, RRM2, RRM2, RRM2, RRM2, RRM2, RRM2, RRM2, RRM2	2
hsa-miR-361-5p	HDAC9, HDAC9, HDAC9, HDAC9, HDAC9, HDAC9, HDAC9, HDAC9, HDAC9, HDAC9, HDAC9, HDAC9, ZNF12, ZNF12, ZNF12, ZNF12, ZNF12, ZNF12	2
hsa-miR-3666	FAM234A, FAM234A, LGALSL, LGALSL	2
hsa-miR-376a-3p	COPS7A, COPS7A, COPS7A, RAPGEFL1, RAPGEFL1, RAPGEFL1, RAPGEFL1, RAPGEFL1, RAPGEFL1	2
hsa-miR-376c-3p	EMP1, WWC3, WWC3, WWC3	2
hsa-miR-378a-3p	VANGL1, ZFP36L2, ZFP36L2	2
hsa-miR-378b	VANGL1, VANGL1, VANGL1, VANGL1, VANGL1, VANGL1, ZFP36L2	2

hsa-miR-378c	VANGL1, VANGL1, VANGL1, VANGL1, VANGL1, VANGL1, VANGL1, VANGL1, ZFP36L2, ZFP36L2	2
hsa-miR-378d	VANGL1, VANGL1, VANGL1, VANGL1, ZFP36L2, ZFP36L2	2
hsa-miR-378e	VANGL1, VANGL1, VANGL1, VANGL1, ZFP36L2, ZFP36L2	2
hsa-miR-378f	VANGL1, VANGL1, VANGL1, VANGL1, ZFP36L2	2
hsa-miR-378h	VANGL1, VANGL1, VANGL1, VANGL1, ZFP36L2	2
hsa-miR-384	CPD, TCEANC2, TCEANC2	2
hsa-miR-422a	VANGL1, ZFP36L2	2
hsa-miR-4295	FAM234A, FAM234A, LGALSL, LGALSL, LGALSL, LGALSL	2
hsa-miR-431-5p	CELF2, CELF2, CELF2, CELF2, CELF2, CELF2, CELF2, CELF2, CELF2, CYB5D1, CYB5D1	2
hsa-miR-4319	RAPGEFL1, RAPGEFL1, REST, REST, REST, REST, REST, REST, REST	2
hsa-miR-432-5p	CENPA, CENPA, EDA2R, EDA2R, EDA2R	2
hsa-miR-449c-5p	FAM234A, FAM234A, FAM234A, TRAM2, TRAM2	2
hsa-miR-450b-5p	MEF2C, STAT5B, STAT5B	2
hsa-miR-4756-5p	RAPGEFL1, RAPGEFL1, RAPGEFL1, RAPGEFL1, RAPGEFL1, RAPGEFL1, RAPGEFL1, RHBDL3, RHBDL3, RHBDL3, RHBDL3, RHBDL3, RHBDL3, RHBDL3, RHBDL3	2
hsa-miR-4782-3p	REST, REST, REST, REST, REST, REST, REST, UBE3A	2
hsa-miR-489-3p	CELF2, CELF2, CELF2, CELF2, CELF2, CELF2, CELF2, CELF2, CELF2, CELF2, YPEL5, YPEL5, YPEL5, YPEL5, YPEL5	2
hsa-miR-5010-5p	TRAM2, VANGL1, VANGL1, VANGL1, VANGL1, VANGL1, VANGL1, VANGL1, VANGL1, VANGL1, VANGL1, VANGL1, VANGL1	2
hsa-miR-503-5p	KLF8, KLF8, WIPI2, WIPI2, WIPI2, WIPI2, WIPI2, WIPI2	2
hsa-miR-510-5p	GPRC5A, GPRC5A, GPRC5A, SNX13	2
hsa-miR-513c-5p	CELF2, PARP11, PARP11, PARP11	2
hsa-miR-5194	CRAMP1, CRAMP1, CRAMP1, CRAMP1, CRAMP1, CRAMP1, TRAM2	2
hsa-miR-5195-3p	FLNB, FLNB, FLNB, GPRC5A, GPRC5A, GPRC5A	2
hsa-miR-542-3p	TCEANC2, TCEANC2, TCEANC2, TCEANC2, TCEANC2, YPEL5	2
hsa-miR-552-3p	HIVEP3, HIVEP3, HIVEP3, VANGL1, VANGL1, VANGL1, VANGL1, VANGL1, VANGL1, VANGL1, VANGL1, VANGL1, VANGL1	2
hsa-miR-589-5p	CAVIN1, CAVIN1, CAVIN1, CAVIN1, PRKAR2A	2
hsa-miR-613	HNRNPA2B1, HNRNPA2B1, HNRNPA2B1, HNRNPA2B1, HNRNPA2B1, HNRNPA2B1, HNRNPA2B1, HNRNPA2B1, ZFP36L2, ZFP36L2	2
hsa-miR-625-5p	TRAM2, YPEL5, YPEL5, YPEL5, YPEL5	2
hsa-miR-641	CPD, CPD, CPD, CPD, CPD, CPD, KIAA1217, KIAA1217, KIAA1217, KIAA1217, KIAA1217, KIAA1217, KIAA1217	2
hsa-miR-642a-5p	PALM, PALM, PALM, PALM, STRAP	2

hsa-miR-650	KRT7, LETMD1, LETMD1, LETMD1, LETMD1, LETMD1, LETMD1, LETMD1, LETMD1	2
hsa-miR-651-5p	CELF2, CELF2, CELF2, CELF2, CELF2, CELF2, CELF2, CELF2, CELF2, CELF2, CYB5D1, CYB5D1, CYB5D1, CYB5D1	2
hsa-miR-654-3p	EMP1, EMP1, EMP1, EMP1, EMP1, EMP1, EMP1, EMP1, EMP1, EMP1, HNRNPA2B1, HNRNPA2B1, HNRNPA2B1, HNRNPA2B1, HNRNPA2B1, HNRNPA2B1, HNRNPA2B1, HNRNPA2B1	2
hsa-miR-655-3p	CELF2, CELF2, CELF2, CELF2, CELF2, CELF2, CELF2, CELF2, CELF2, CELF2, LGALSL, LGALSL, LGALSL, LGALSL	2
hsa-miR-663a	MRPL4, MRPL4, WWC3, WWC3	2
hsa-miR-665	CYB5D1, CYB5D1, CYB5D1, CYB5D1, CYB5D1, PALM	2
hsa-miR-7-5p	KLHL28, KLHL28, KLHL28, RBM23, RBM23, RBM23	2
hsa-miR-1179	MEF2C, MEF2C, MEF2C, MEF2C, MEF2C	1
hsa-miR-1185-5p	CELF2, CELF2, CELF2, CELF2, CELF2, CELF2, CELF2, CELF2, CELF2, CELF2, CELF2, CELF2, CELF2, CELF2, CELF2, CELF2, CELF2, CELF2, CELF2, CELF2	1
hsa-miR-1269b	VANGL1, VANGL1, VANGL1, VANGL1, VANGL1, VANGL1	1
hsa-miR-1287-5p	HIVEP3, HIVEP3, HIVEP3, HIVEP3, HIVEP3, HIVEP3, HIVEP3, HIVEP3, HIVEP3, HIVEP3, HIVEP3, HIVEP3	1
hsa-miR-129-1-3p	KIAA1217, KIAA1217, KIAA1217, KIAA1217, KIAA1217, KIAA1217, KIAA1217, KIAA1217, KIAA1217	1
hsa-miR-129-2-3p	KIAA1217, KIAA1217, KIAA1217, KIAA1217, KIAA1217, KIAA1217, KIAA1217, KIAA1217, KIAA1217	1
hsa-miR-1306-5p	LCMT2	1
hsa-miR-134-5p	CELF2, CELF2, CELF2, CELF2, CELF2	1
hsa-miR-136-5p	CPD	1
hsa-miR-154-5p	SLC16A1, SLC16A1	1
hsa-miR-155-5p	CELF2, CELF2, CELF2, CELF2, CELF2	1
hsa-miR-181d-5p	DIP2C, DIP2C	1
hsa-miR-188-5p	UBE3A, UBE3A	1
hsa-miR-18a-5p	HDAC9, HDAC9, HDAC9, HDAC9, HDAC9	1
hsa-miR-18b-5p	HDAC9, HDAC9, HDAC9	1
hsa-miR-190a-5p	HNRNPA2B1	1
hsa-miR-193a-5p	NR1D2	1
hsa-miR-21-5p	MEF2C, MEF2C	1
hsa-miR-212-5p	TCEANC2, TCEANC2, TCEANC2	1
hsa-miR-219a-2-3p	DHFR	1
hsa-miR-221-3p	LHFPL2, LHFPL2	1
hsa-miR-222-3p	LHFPL2, LHFPL2	1
hsa-miR-2355-5p	MEF2C, MEF2C, MEF2C, MEF2C, MEF2C	1
hsa-miR-296-3p	VANGL1, VANGL1, VANGL1, VANGL1	1

hsa-miR-299-3p	PMP22, PMP22, PMP22, PMP22, PMP22, PMP22, PMP22, PMP22, PMP22, PMP22, PMP22, PMP22, PMP22, PMP22, PMP22, PMP22, PMP22, PMP22, PMP22, PMP22	1
hsa-miR-3139	OTUB1, OTUB1, OTUB1	1
hsa-miR-3150a-3p	SLC6A17, SLC6A17, SLC6A17, SLC6A17, SLC6A17	1
hsa-miR-3150b-3p	GPRC5A, GPRC5A, GPRC5A, GPRC5A	1
hsa-miR-3167	ERLEC1, ERLEC1	1
hsa-miR-324-3p	TWF2	1
hsa-miR-325	TTLL7	1
hsa-miR-328-3p	NFATC4, NFATC4, NFATC4, NFATC4, NFATC4, NFATC4, NFATC4, NFATC4	1
hsa-miR-331-3p	TCEANC2	1
hsa-miR-335-5p	KLHL28	1
hsa-miR-345-3p	HNRNPA2B1, HNRNPA2B1, HNRNPA2B1, HNRNPA2B1, HNRNPA2B1	1
hsa-miR-345-5p	CYB5D1	1
hsa-miR-346	ERLEC1, ERLEC1, ERLEC1	1
hsa-miR-34b-5p	TRAM2	1
hsa-miR-361-3p	EMP1, EMP1, EMP1, EMP1, EMP1, EMP1, EMP1, EMP1, EMP1, EMP1, EMP1, EMP1, EMP1, EMP1	1
hsa-miR-3619-5p	RAPGEFL1, RAPGEFL1, RAPGEFL1, RAPGEFL1, RAPGEFL1, RAPGEFL1, RAPGEFL1, RAPGEFL1, RAPGEFL1, RAPGEFL1, RAPGEFL1, RAPGEFL1, RAPGEFL1, RAPGEFL1	1
hsa-miR-3679-5p	CELF2, CELF2, CELF2, CELF2, CELF2, CELF2, CELF2, CELF2, CELF2	1
hsa-miR-371a-3p	NR1D2	1
hsa-miR-374a-3p	HNRNPA2B1, HNRNPA2B1, HNRNPA2B1, HNRNPA2B1, HNRNPA2B1, HNRNPA2B1, HNRNPA2B1, HNRNPA2B1	1
hsa-miR-374a-5p	TTLL7	1
hsa-miR-374b-5p	TTLL7	1
hsa-miR-374c-5p	CELF2, CELF2, CELF2, CELF2, CELF2, CELF2, CELF2, CELF2, CELF2, CELF2, CELF2, CELF2	1
hsa-miR-378i	VANGL1, VANGL1, VANGL1, VANGL1, VANGL1, VANGL1	1
hsa-miR-383-5p	STRAP, STRAP	1
hsa-miR-410-3p	CPD, CPD	1
hsa-miR-411-3p	LGALSL, LGALSL	1
hsa-miR-421	YPEL5, YPEL5, YPEL5, YPEL5, YPEL5, YPEL5, YPEL5, YPEL5, YPEL5, YPEL5, YPEL5, YPEL5, YPEL5, YPEL5, YPEL5, YPEL5	1
hsa-miR-423-3p	LGALSL, LGALSL	1
hsa-miR-4262	KCNQ5, KCNQ5	1
hsa-miR-4306	CRAMP1, CRAMP1, CRAMP1, CRAMP1, CRAMP1, CRAMP1	1
hsa-miR-4428	YPEL5, YPEL5, YPEL5, YPEL5, YPEL5	1
hsa-miR-4429	CPD, CPD, CPD, CPD, CPD, CPD, CPD, CPD, CPD, CPD, CPD, CPD	1
hsa-miR-4465	REST, REST, REST, REST, REST, REST, REST	1

hsa-miR-4640-5p	CRAMP1, CRAMP1, CRAMP1, CRAMP1, CRAMP1, CRAMP1	1
hsa-miR-4644	VANGL1, VANGL1, VANGL1, VANGL1, VANGL1, VANGL1, VANGL1, VANGL1, VANGL1, VANGL1, VANGL1, VANGL1, VANGL1, VANGL1	1
hsa-miR-4676-3p	CELF2, CELF2, CELF2, CELF2, CELF2	1
hsa-miR-4739	EMP1, EMP1	1
hsa-miR-4766-5p	HNRNPA2B1, HNRNPA2B1, HNRNPA2B1, HNRNPA2B1, HNRNPA2B1, HNRNPA2B1, HNRNPA2B1, HNRNPA2B1, HNRNPA2B1, HNRNPA2B1, HNRNPA2B1, HNRNPA2B1, HNRNPA2B1, HNRNPA2B1, HNRNPA2B1, HNRNPA2B1	1
hsa-miR-4770	WWC3	1
hsa-miR-483-3p	ICAM1, ICAM1	1
hsa-miR-485-3p	CPD, CPD, CPD, CPD, CPD, CPD	1
hsa-miR-487a-3p	CPD, CPD	1
hsa-miR-490-3p	WWC3	1
hsa-miR-493-3p	TCEANC2	1
hsa-miR-500a-3p	NR1D2	1
hsa-miR-501-3p	NR1D2	1
hsa-miR-509-3p	SNX13	1
hsa-miR-514a-3p	NR1D2	1
hsa-miR-514a-5p	TCEANC2, TCEANC2, TCEANC2	1
hsa-miR-516b-5p	PMP22, PMP22	1
hsa-miR-526b-5p	HNRNPA2B1, HNRNPA2B1, HNRNPA2B1, HNRNPA2B1	1
hsa-miR-532-5p	CPD, CPD	1
hsa-miR-539-3p	CPD, CPD, CPD	1
hsa-miR-541-5p	UBE3A, UBE3A	1
hsa-miR-545-3p	CELF2, CELF2	1
hsa-miR-545-5p	CELF2, CELF2	1
hsa-miR-551b-3p	MEF2C, MEF2C, MEF2C, MEF2C, MEF2C, MEF2C	1
hsa-miR-556-3p	CENPA, CENPA, CENPA, CENPA	1
hsa-miR-582-3p	EBPL, EBPL	1
hsa-miR-584-5p	NR1D2, NR1D2, NR1D2, NR1D2, NR1D2, NR1D2	1
hsa-miR-590-5p	MEF2C, MEF2C, MEF2C, MEF2C, MEF2C, MEF2C, MEF2C, MEF2C, MEF2C, MEF2C, MEF2C, MEF2C, MEF2C, MEF2C, MEF2C, MEF2C	1
hsa-miR-6088	WWC3, WWC3	1
hsa-miR-616-3p	CELF2, CELF2	1
hsa-miR-628-5p	PRKAR2A, PRKAR2A, PRKAR2A, PRKAR2A, PRKAR2A, PRKAR2A, PRKAR2A, PRKAR2A, PRKAR2A	1

hsa-miR-629-5p	CYB5D1, CYB5D1	1
hsa-miR-6504-5p	HNRNPA2B1	1
hsa-miR-652-3p	ZNF430, ZNF430, ZNF430	1
hsa-miR-653-5p	TRAM2, TRAM2, TRAM2, TRAM2, TRAM2, TRAM2	1
hsa-miR-656-3p	SNX13, SNX13	1
hsa-miR-660-5p	TFAP2B, TFAP2B, TFAP2B	1
hsa-miR-668-3p	EMP1, EMP1	1
hsa-miR-670-5p	FKBP2, FKBP2, FKBP2	1
hsa-miR-744-5p	CRAMP1, CRAMP1, CRAMP1, CRAMP1	1
hsa-miR-758-3p	STAT5B, STAT5B, STAT5B	1
hsa-miR-769-5p	YPEL5, YPEL5, YPEL5, YPEL5, YPEL5	1
hsa-miR-876-5p	ERLEC1, ERLEC1	1
hsa-miR-885-5p	NR1D2	1
hsa-miR-892c-3p	CELF2, CELF2, CELF2, CELF2, CELF2, CELF2, CELF2, CELF2, CELF2, CELF2, CELF2, CELF2, CELF2, CELF2	1
hsa-miR-9-3p	KIAA1217, KIAA1217, KIAA1217, KIAA1217, KIAA1217, KIAA1217, KIAA1217	1
hsa-miR-934	LCMT2	1
**miRNA**	**Down_genes**	**count_unique**
hsa-miR-106a-5p	ANO6, ANO6, ANO6, ANO6, ANO6, BNIP3L, BNIP3L, BNIP3L, BNIP3L, BNIP3L, BNIP3L, BNIP3L, BNIP3L, BNIP3L, BNIP3L, BNIP3L, BNIP3L, BNIP3L, BNIP3L, BTBD10, CELSR2, CMPK1, COPS2, CREB5, CREB5, CREB5, CREB5, CXCL6, EHD3, FAM168A, FAM168A, FAM177A1, FAM177A1, FAM177A1, FAM177A1, IP6K1, IP6K1, IP6K1, ITGB8, ITGB8, ITGB8, ITGB8, ITGB8, ITGB8, ITGB8, ITGB8, ITGB8, ITGB8, ITGB8, ITGB8, ITGB8, ITGB8, ITGB8, ITGB8, ITGB8, ITGB8, LDLRAD3, NCEH1, NCEH1, NCEH1, NCEH1, NCEH1, NCEH1, NCEH1, NCEH1, NCEH1, NCEH1, NCEH1, NCEH1, NSD2, RARB, RARB, RARB, RNF24, RNF24, RNF24, RNF24, RPS6KA3, SCML2, SCML2, SCML2, SIKE1, SIKE1, SIRPA, SIRPA, SIRPA, SIRPA, SIRPA, SIRPA, SIRPA, SIRPA, TAOK1, TAOK1, TAOK1, TAOK1, TAOK1, TAOK1, TAOK1, TAOK1, TAOK1, TCF7L1, TCF7L1, TCF7L1, TCF7L1, TCF7L1, TCF7L1, TNRC6A, TNRC6A, TNRC6A, TNRC6A, TNRC6A, TNRC6A, TNRC6A, TNRC6A, TNRC6A, TRIM33, TRIM33, TRIM33, TRIM33, TRIM33, TRIM33, TRIM33, TRIM33, TVP23B, TVP23B, TVP23B, WNK3, WNK3, WNK3, WNK3, ZNF320, ZNF320, ZNF320, ZNF320, ZNF320, ZNF320, ZNF320	29
hsa-miR-424-5p	ABTB2, ADORA2A, ADORA2A, ADORA2A, ADORA2A, ARL6IP5, ARL6IP5, ARL6IP5, BICD1, BICD1, BICD1, BICD1, BICD1, BICD1, CCDC6, CCDC6, CCDC6, CCDC6, CDC25A, CDC25A, CDC25A, CDC25A, CDC25A, CDC25A, CDK5R1, CDK5R1, CDK5R1, CDK5R1, CDK5R1, COMMD1, COMMD1, COMMD1, COMMD1, COMMD1, COPS2, COPS2, COPS2, CREB3L1, CREB3L1, EXTL3, EXTL3, FBXO33, MADD, MADD, MADD, MADD, MADD, MADD, MADD, MADD, MADD, MED1, MED1, MNT, MNT, MYO1C, MYO1C, MYO1C, MYO1C, MYO1C, MYO1C, MYO1C, NSD2, PCNX1, PCNX1, PCNX1, RARB, RARB, RARB, RBM24, RBM24, RBM24, RBM24, RBM24, RBM24, RNF24, RNF24, RNF24, RNF24, RNF8, SCN8A, SCN8A, SCN8A, SCN8A, SCN8A, SCN8A, SCN8A, SCN8A, SCN8A, SH3GL2, SH3GL2, SH3GL2, SKI, SKI, SKI, SKI, SKI, SKI, SKI, SKI, SOX5, SOX5, SOX5, TVP23B, TVP23B, USP9X, USP9X, USP9X, USP9X, USP9X, USP9X	28

hsa-miR-20a-5p	ANO6, ANO6, ANO6, ANO6, ANO6, BNIP3L, BNIP3L, BTBD10, CELSR2, CMPK1, COPS2, CREB5, CREB5, CREB5, CREB5, CREB5, CREB5, CREB5, CREB5, CXCL6, EHD3, FAM177A1, FAM177A1, FAM177A1, FAM177A1, IP6K1, IP6K1, IP6K1, ITGB8, LDLRAD3, NCEH1, NCEH1, NCEH1, NCEH1, NCEH1, NCEH1, NCEH1, NCEH1, NCEH1, NCEH1, NCEH1, NCEH1, NSD2, NSD2, NSD2, NSD2, NSD2, NSD2, NSD2, NSD2, NSD2, NSD2, NSD2, NSD2, NSD2, NSD2, NSD2, NSD2, NSD2, NSD2, RNF24, RNF24, RNF24, RNF24, RNF24, RNF24, RNF24, RNF24, RPS6KA3, SCML2, SCML2, SCML2, SIKE1, SIKE1, SIKE1, SIKE1, SIRPA, SIRPA, SIRPA, SIRPA, SIRPA, SIRPA, SIRPA, SIRPA, SKI, TAOK1, TAOK1, TAOK1, TAOK1, TAOK1, TAOK1, TAOK1, TAOK1, TAOK1, TAOK1, TAOK1, TCF7L1, TCF7L1, TCF7L1, TCF7L1, TCF7L1, TCF7L1, TNRC6A, TNRC6A, TNRC6A, TNRC6A, TNRC6A, TNRC6A, TNRC6A, TNRC6A, TNRC6A, TVP23B, TVP23B, TVP23B, WNK3, WNK3, WNK3, WNK3, WNK3, WNK3, WNK3, WNK3, WNK3, WNK3, ZNF320, ZNF320, ZNF320, ZNF320, ZNF320, ZNF320, ZNF320	27
hsa-miR-497-5p	ABTB2, ADORA2A, ADORA2A, ADORA2A, ADORA2A, ARL6IP5, ARL6IP5, BICD1, BICD1, BICD1, BICD1, BICD1, BICD1, CCDC6, CCDC6, CDC25A, CDC25A, CDC25A, CDC25A, CDC25A, CDC25A, CDK5R1, CDK5R1, COMMD1, COMMD1, COMMD1, COMMD1, COMMD1, COPS2, COPS2, EXTL3, EXTL3, FBXO33, FBXO33, MADD, MADD, MADD, MADD, MADD, MADD, MADD, MADD, MADD, MADD, MADD, MADD, MADD, MADD, MADD, MADD, MADD, MADD, MNT, MNT, MYO1C, MYO1C, MYO1C, MYO1C, MYO1C, MYO1C, MYO1C, NSD2, PCNX1, PCNX1, PCNX1, PCNX1, RARB, RARB, RARB, RBM24, RNF24, RNF24, RNF24, RNF24, RNF8, SCN8A, SCN8A, SCN8A, SCN8A, SCN8A, SCN8A, SH3GL2, SH3GL2, SKI, SKI, SKI, SKI, SKI, SKI, SKI, SKI, SOX5, TAOK1, TAOK1, TAOK1, TAOK1, TAOK1, TAOK1, TVP23B, TVP23B, USP9X, USP9X, USP9X, USP9X, USP9X, USP9X	27
hsa-miR-106b-5p	ANO6, ANO6, ANO6, BNIP3L, CELSR2, CMPK1, CMPK1, CMPK1, COPS2, CREB5, CREB5, CREB5, CREB5, CREB5, CREB5, CREB5, CXCL6, EHD3, FAM168A, FAM168A, FAM177A1, FAM177A1, FAM177A1, FAM177A1, IP6K1, IP6K1, IP6K1, ITGB8, LDLRAD3, NCEH1, NCEH1, NCEH1, NCEH1, NCEH1, NCEH1, NCEH1, NCEH1, NCEH1, NCEH1, NCEH1, NCEH1, NSD2, NSD2, NSD2, NSD2, NSD2, NSD2, NSD2, NSD2, NSD2, NSD2, NSD2, NSD2, NSD2, NSD2, NSD2, NSD2, NSD2, RNF24, RNF24, SCML2, SCML2, SCML2, SIKE1, SIKE1, SIRPA, SIRPA, SIRPA, SIRPA, SKI, TAOK1, TCF7L1, TCF7L1, TCF7L1, TCF7L1, TNRC6A, TNRC6A, TNRC6A, TNRC6A, TNRC6A, TNRC6A, TNRC6A, TNRC6A, TNRC6A, TVP23B, TVP23B, TVP23B, WNK3, WNK3, WNK3, WNK3, WNK3, WNK3, ZNF320, ZNF320, ZNF320, ZNF320, ZNF320, ZNF320, ZNF320	26
hsa-miR-15a-5p	ADORA2A, ADORA2A, ADORA2A, ADORA2A, ANLN, ANLN, ANLN, ANLN, ARL6IP5, ARL6IP5, ARL6IP5, BICD1, BICD1, BICD1, BICD1, BICD1, BICD1, BICD1, CCDC6, CCDC6, CDC25A, CDC25A, CDC25A, CDC25A, CDC25A, CDC25A, CDK5R1, CDK5R1, CDK5R1, COPS2, COPS2, COPS2, EXTL3, EXTL3, EXTL3, EXTL3, EXTL3, EXTL3, MADD, MADD, MADD, MADD, MADD, MADD, MADD, MADD, MADD, MNT, MNT, NSD2, PCNX1, PCNX1, PCNX1, PCNX1, RARB, RARB, RARB, RBM24, RBM24, RBM24, RNF24, RNF24, RNF24, RNF24, RPS6KA3, RPS6KA3, RPS6KA3, RPS6KA3, RPS6KA3, RPS6KA3, SCN8A, SCN8A, SCN8A, SCN8A, SCN8A, SCN8A, SCN8A, SCN8A, SCN8A, SH3GL2, SH3GL2, SKI, SKI, SKI, SKI, SKI, SKI, SKI, SKI, SKI, TAOK1, TAOK1, TAOK1, TAOK1, TAOK1, TVP23B, TVP23B, USP9X, USP9X, USP9X, USP9X, USP9X, USP9X	23

hsa-miR-15b-5p	ADORA2A, ADORA2A, ADORA2A, ADORA2A, ANLN, ANLN, ARL6IP5, ARL6IP5, BICD1, BICD1, BICD1, BICD1, BICD1, BICD1, CDC25A, CDC25A, CDC25A, CDC25A, CDC25A, CDC25A, CDK5R1, CDK5R1, CDK5R1, COPS2, COPS2, EXTL3, EXTL3, EXTL3, EXTL3, EXTL3, EXTL3, FBXO33, FBXO33, MED1, MED1, MNT, MNT, NSD2, PCNX1, PCNX1, PCNX1, PCNX1, RARB, RARB, RARB, RBM24, RNF24, RNF24, RNF24, RNF24, RNF24, RNF24, RNF24, RNF24, RNF24, RNF24, RNF24, RNF24, RNF24, RNF24, RNF8, SCN8A, SCN8A, SCN8A, SCN8A, SCN8A, SCN8A, SCN8A, SCN8A, SCN8A, SCN8A, SCN8A, SCN8A, SH3GL2, SH3GL2, SKI, SKI, SKI, SKI, SKI, SKI, SKI, SKI, SKI, TAOK1, TAOK1, TAOK1, TAOK1, TVP23B, TVP23B, USP9X, USP9X, USP9X, USP9X	23
hsa-miR-195-5p	ADORA2A, ADORA2A, ADORA2A, ADORA2A, ANLN, ANLN, BICD1, BICD1, BICD1, BICD1, BICD1, BLOC1S6, BLOC1S6, BLOC1S6, CCDC6, CCDC6, CDC25A, CDC25A, CDC25A, CDC25A, CDC25A, CDC25A, CDK5R1, CDK5R1, COPS2, COPS2, EXTL3, EXTL3, MADD, MADD, MADD, MADD, MADD, MADD, MADD, MADD, MADD, MNT, MNT, NSD2, PCNX1, PCNX1, PCNX1, PCNX1, RARB, RARB, RARB, RBM24, RBM24, RBM24, RBM24, RBM24, RNF24, RNF24, RNF24, RNF24, RNF24, RNF24, RNF24, RNF24, RNF24, RNF24, RNF24, RNF24, RNF24, RNF24, RNF8, SCN8A, SCN8A, SCN8A, SCN8A, SCN8A, SCN8A, SCN8A, SCN8A, SCN8A, SCN8A, SCN8A, SCN8A, SH3GL2, SH3GL2, SKI, SKI, SKI, SKI, SKI, SKI, SKI, SKI, SKI, TAOK1, TAOK1, TAOK1, TAOK1, TVP23B, TVP23B, USP9X, USP9X, USP9X, USP9X, USP9X, USP9X, USP9X, USP9X	23
hsa-miR-16-5p	ADORA2A, ADORA2A, ADORA2A, ADORA2A, BICD1, BICD1, BICD1, BICD1, BICD1, CCDC6, CCDC6, CCDC6, CCDC6, CDC25A, CDC25A, CDC25A, CDC25A, CDC25A, CDC25A, CDK5R1, CDK5R1, COPS2, COPS2, EXTL3, EXTL3, EXTL3, EXTL3, EXTL3, MADD, MADD, MADD, MADD, MADD, MADD, MADD, MADD, MADD, MADD, MADD, MADD, MADD, MADD, MADD, MADD, MADD, MNT, MNT, NSD2, PCNX1, PCNX1, PCNX1, PCNX1, RARB, RARB, RARB, RBM24, RBM24, RBM24, RNF24, RNF24, RNF24, RNF24, RNF24, RNF24, RNF24, RNF24, RNF24, RNF24, RNF24, RNF24, RNF24, RNF24, RNF8, RPS6KA3, RPS6KA3, RPS6KA3, RPS6KA3, RPS6KA3, RPS6KA3, SCN8A, SCN8A, SCN8A, SCN8A, SCN8A, SCN8A, SCN8A, SCN8A, SCN8A, SH3GL2, SH3GL2, SKI, SKI, SKI, SKI, SKI, SKI, SKI, SKI, TAOK1, TAOK1, TAOK1, TAOK1, TAOK1, TAOK1, TAOK1, TAOK1, TAOK1, TAOK1, TAOK1, TAOK1, USP9X, USP9X, USP9X, USP9X, USP9X, USP9X, USP9X, USP9X	21
hsa-miR-520d-3p	ANO6, ANO6, ANO6, ARHGAP29, BNIP3L, BNIP3L, CHMP3, CHMP3, CHMP3, CHMP3, CMPK1, CREB5, FAM168A, FAM168A, FAM177A1, FAM177A1, FAM177A1, FAM177A1, FMNL3, FMNL3, FMNL3, ITGB8, ITGB8, ITGB8, ITGB8, KDM1B, KDM1B, KDM1B, LIMA1, LIMA1, LIMA1, LIMA1, LIMA1, LIMA1, LIMA1, LIMA1, LIMA1, LIMA1, LIMA1, LIMA1, NSD2, NSD2, NSD2, NSD2, NSD2, NSD2, NSD2, NSD2, NSD2, NSD2, RPS6KA3, RPS6KA3, RPS6KA3, SIRPA, SIRPA, SIRPA, SIRPA, SIRPA, SIRPA, SIRPA, SIRPA, SIRPA, SIRPA, SIRPA, SIRPA, SIRPA, SIRPA, SIRPA, SIRPA, TAOK1, TAOK1, TAOK1, TCF7L1, TCF7L1, TCF7L1, TCF7L1, TVP23B, TVP23B, TVP23B, USP24, ZMYND11, ZMYND11, ZMYND11, ZMYND11, ZMYND11, ZMYND11, ZMYND11, ZMYND11, ZMYND11	20
hsa-miR-17-5p	ANO6, ANO6, ANO6, ANO6, ANO6, BNIP3L, BNIP3L, BNIP3L, BNIP3L, BNIP3L, BNIP3L, BNIP3L, BTBD10, CELSR2, CMPK1, CREB5, CREB5, CREB5, CREB5, FAM168A, FAM168A, IP6K1, IP6K1, IP6K1, ITGB8, ITGB8, ITGB8, ITGB8, ITGB8, ITGB8, ITGB8, ITGB8, ITGB8, ITGB8, ITGB8, ITGB8, ITGB8, ITGB8, ITGB8, ITGB8, ITGB8, ITGB8, LDLRAD3, NCEH1, NCEH1, NCEH1, NCEH1, NCEH1, NCEH1, NSD2, RARB, RARB, RARB, RPS6KA3, SCML2, SCML2, SCML2, TAOK1, TAOK1, TAOK1, TAOK1, TAOK1, TAOK1, TAOK1, TAOK1, TAOK1, TCF7L1, TCF7L1, TCF7L1, TNRC6A, TNRC6A, TNRC6A, WNK3, WNK3, WNK3, WNK3	19

hsa-miR-302a-3p	ANO6, ANO6, ANO6, ARHGAP29, BNIP3L, BNIP3L, BNIP3L, BNIP3L, CHMP3, CHMP3, CHMP3, CHMP3, CHMP3, CHMP3, CMPK1, CMPK1, CMPK1, CMPK1, CREB5, CREB5, CREB5, CREB5, CREB5, CREB5, CREB5, CREB5, CREB5, CREB5, CREB5, CREB5, CREB5, CREB5, CREB5, CREB5, CREB5, FAM177A1, FAM177A1, FAM177A1, FAM177A1, IP6K1, IP6K1, IP6K1, ITGB8, ITGB8, ITGB8, ITGB8, KDM1B, KDM1B, KDM1B, KDM1B, KDM1B, KDM1B, KDM1B, KDM1B, KDM1B, KDM1B, LDLRAD3, NSD2, NSD2, NSD2, NSD2, NSD2, NSD2, NSD2, NSD2, NSD2, NSD2, RPS6KA3, RPS6KA3, RPS6KA3, SIRPA, ST7L, ST7L, TAOK1, TCF7L1, TCF7L1, TCF7L1, TVP23B, TVP23B, TVP23B, ZMYND11, ZMYND11, ZMYND11, ZMYND11, ZMYND11, ZMYND11, ZMYND11, ZMYND11, ZMYND11	19
hsa-miR-372-3p	ANO6, ANO6, ANO6, ARHGAP29, BNIP3L, BNIP3L, BNIP3L, BNIP3L, CMPK1, CMPK1, CREB5, CREB5, EHD3, FAM168A, FAM168A, FAM177A1, FAM177A1, FAM177A1, FAM177A1, FMNL3, FMNL3, FMNL3, ITGB8, ITGB8, ITGB8, ITGB8, KDM1B, KDM1B, KDM1B, KDM1B, KDM1B, KDM1B, LIMA1, LIMA1, LIMA1, LIMA1, LIMA1, LIMA1, LIMA1, LIMA1, LIMA1, LIMA1, LIMA1, LIMA1, NSD2, NSD2, NSD2, NSD2, RPS6KA3, RPS6KA3, RPS6KA3, SIRPA, SIRPA, SIRPA, SIRPA, SIRPA, SIRPA, SIRPA, SIRPA, SIRPA, SIRPA, SIRPA, SIRPA, SIRPA, SIRPA, SIRPA, SIRPA, TAOK1, TAOK1, TAOK1, TAOK1, TAOK1, TAOK1, TCF7L1, TCF7L1, TVP23B, TVP23B, TVP23B, ZMYND11, ZMYND11, ZMYND11, ZMYND11, ZMYND11, ZMYND11, ZMYND11, ZMYND11, ZMYND11	19
hsa-miR-429	ANLN, ANLN, ANO6, ANO6, ANO6, ANO6, ANO6, ANO6, CHAC2, CHAC2, CREB5, CREB5, DAG1, DAG1, DAG1, DAG1, DAG1, DAG1, DAG1, FSCN1, GOLGA8B, GOLGA8B, GOLGA8B, GOLGA8B, IP6K1, IP6K1, IP6K1, NSD2, NSD2, NSD2, NSD2, NSD2, NSD2, NXPH1, NXPH1, PCNX1, PCNX1, PPIL4, PPIL4, PPIL4, PPIL4, PRKAA2, PRKAA2, PRKAA2, PRKAA2, PRKAA2, PRKAA2, PRKAA2, PRKAA2, PRKAA2, PRKAA2, PRKAA2, PRKAA2, PRKAA2, PRKAA2, PRKAA2, PRKAA2, SCP2, SCP2, SCP2, SCP2, SOX5, SOX5, SOX5, SOX5, SOX5, SOX5, TAOK1, TAOK1, TMX4, TMX4, UBA6, UNC50, UNC50, UNC50, UNC50, UNC50	19
hsa-miR-101-3p	ANTXR2, BLOC1S6, BLOC1S6, BLOC1S6, BLOC1S6, BLOC1S6, BLOC1S6, CDK5R1, CDK5R1, CHAC2, CHST12, CHST12, CHST12, CHST12, CHST12, CHST12, CHST12, CHST12, CXCL6, CXCL6, DAG1, DAG1, DAG1, DAG1, DAG1, DAG1, DAG1, FAM177A1, FAM177A1, FANCM, FANCM, FANCM, FANCM, ITGB8, ITGB8, KLHDC1, LGI2, LGI2, MPP7, MPP7, NEK7, NEK7, PLPPR4, PLPPR4, SIKE1, SIKE1, SIKE1, SIKE1, SIKE1, SIKE1, STAMBP, STAMBP, STAMBP, STAMBP, STAMBP, STAMBP, ZNF143	18
hsa-miR-20b-5p	ANO6, ANO6, ANO6, ANO6, ANO6, BNIP3L, CELSR2, CMPK1, CMPK1, CREB5, CREB5, CREB5, CREB5, CREB5, CREB5, CREB5, CREB5, IP6K1, IP6K1, IP6K1, ITGB8, LDLRAD3, NCEH1, NCEH1, NCEH1, NCEH1, NCEH1, NCEH1, NSD2, RARB, RARB, RARB, RPS6KA3, RPS6KA3, RPS6KA3, RPS6KA3, RPS6KA3, RPS6KA3, RPS6KA3, RPS6KA3, RPS6KA3, RPS6KA3, SCML2, SCML2, SCML2, SKI, TAOK1, TAOK1, TAOK1, TAOK1, TAOK1, TAOK1, TAOK1, TAOK1, TAOK1, TAOK1, TAOK1, TAOK1, TCF7L1, TCF7L1, TCF7L1, TNRC6A, TNRC6A, TNRC6A, WNK3, WNK3, WNK3, WNK3, WNK3, WNK3, WNK3, WNK3, WNK3, WNK3	18
hsa-miR-520c-3p	ANO6, ANO6, ANO6, ARHGAP29, BNIP3L, BNIP3L, BNIP3L, BNIP3L, CREB5, CREB5, CREB5, CREB5, CREB5, CREB5, FAM168A, FAM168A, FAM177A1, FAM177A1, FAM177A1, FAM177A1, FMNL3, FMNL3, FMNL3, ITGB8, ITGB8, ITGB8, ITGB8, ITGB8, KDM1B, KDM1B, KDM1B, LDLRAD3, LIMA1, LIMA1, LIMA1, LIMA1, LIMA1, LIMA1, LIMA1, LIMA1, LIMA1, LIMA1, LIMA1, LIMA1, NSD2, NSD2, NSD2, NSD2, NSD2, NSD2, NSD2, NSD2, NSD2, NSD2, NSD2, NSD2, NSD2, NSD2, RPS6KA3, RPS6KA3, RPS6KA3, SIRPA, SIRPA, SIRPA, SIRPA, SIRPA, SIRPA, SIRPA, SIRPA, SIRPA, SIRPA, SIRPA, SIRPA, SIRPA, SIRPA, SIRPA, SIRPA, TAOK1, TAOK1, TAOK1, TCF7L1, TCF7L1, TVP23B, TVP23B, TVP23B, ZMYND11, ZMYND11, ZMYND11, ZMYND11, ZMYND11, ZMYND11, ZMYND11, ZMYND11, ZMYND11	18

hsa-miR-182-5p	ANLN, ANLN, ANLN, ANLN, ANTXR2, ARHGAP29, ARHGAP29, ATF1, ATF1, ATF1, BICD1, BICD1, BICD1, BICD1, CREB3L1, CREB3L1, ITGB8, ITGB8, KCTD7, LASP1, LASP1, LASP1, MEIS2, MEIS2, PCNX1, PCNX1, PCNX1, PCNX1, PLPPR4, PLPPR4, PLPPR4, PLPPR4, SIKE1, SIKE1, SIKE1, SIKE1, SIKE1, SIKE1, SIKE1, SIKE1, SIKE1, SIKE1, SIKE1, SIKE1, SIKE1, SIKE1, SIKE1, SIKE1, SOX5, TAOK1, USP24, ZMPSTE24	17
hsa-miR-206	BICD1, EML4, FAM72B, FAM72B, FBXO33, IP6K2, IP6K2, IP6K2, IP6K2, IP6K2, LASP1, MED1, MED1, MED1, MPP7, MPP7, NCK2, NCK2, NCK2, NRP1, NRP1, NRP1, NRP1, PRKX, PRKX, PRKX, SMG7, SMG7, SMG7, SMG7, TPM4, TPM4, TPM4, TPM4, TPM4, TPM4, TPM4, TPM4, TPM4, TPM4, TPM4, TPM4, TPM4, TPM4, TPM4, TPM4, TPM4, UBXN2B, UBXN2B, UNC50, UNC50, UNC50, UNC50, UNC50, WDR6, WDR6, WDR6, WDR6, ZMYND11, ZMYND11, ZMYND11, ZMYND11, ZMYND11, ZMYND11, ZMYND11, ZMYND11, ZMYND11	17
hsa-miR-302d-3p	ANO6, ANO6, ANO6, ARHGAP29, BNIP3L, BNIP3L, BNIP3L, BNIP3L, BNIP3L, BNIP3L, BNIP3L, BNIP3L, BNIP3L, BNIP3L, BNIP3L, BNIP3L, CHMP3, CHMP3, CHMP3, CHMP3, CHMP3, CHMP3, CHMP3, CHMP3, CHMP3, CHMP3, CMPK1, CMPK1, CREB5, CREB5, CREB5, CREB5, CREB5, FAM177A1, FAM177A1, FAM177A1, FAM177A1, ITGB8, ITGB8, ITGB8, ITGB8, ITGB8, KDM1B, KDM1B, KDM1B, KDM1B, KDM1B, LDLRAD3, NSD2, NSD2, NSD2, NSD2, NSD2, NSD2, NSD2, NSD2, NSD2, NSD2, NSD2, NSD2, NSD2, NSD2, RPS6KA3, RPS6KA3, SIRPA, SIRPA, SIRPA, SIRPA, SIRPA, SIRPA, SIRPA, SIRPA, SIRPA, SIRPA, SIRPA, SIRPA, SIRPA, SIRPA, SIRPA, SIRPA, SIRPA, SIRPA, SIRPA, TAOK1, TAOK1, TCF7L1, TVP23B, TVP23B, TVP23B, ZMYND11, ZMYND11, ZMYND11, ZMYND11, ZMYND11, ZMYND11, ZMYND11, ZMYND11, ZMYND11	17
hsa-miR-373-3p	ARHGAP29, BNIP3L, BNIP3L, BNIP3L, BNIP3L, CCDC6, CHMP3, CHMP3, CHMP3, CHMP3, CREB5, CREB5, FAM177A1, FAM177A1, FAM177A1, FAM177A1, ITGB8, ITGB8, ITGB8, KDM1B, KDM1B, KDM1B, KDM1B, KDM1B, LDLRAD3, NSD2, RPS6KA3, RPS6KA3, RPS6KA3, RPS6KA3, SIRPA, SIRPA, SIRPA, SIRPA, SIRPA, SIRPA, SIRPA, SIRPA, TAOK1, TAOK1, TAOK1, TAOK1, TAOK1, TCF7L1, TVP23B, TVP23B, TVP23B, USP24, ZMYND11, ZMYND11, ZMYND11, ZMYND11, ZMYND11, ZMYND11, ZMYND11, ZMYND11, ZMYND11	17
hsa-miR-495-3p	BLOC1S6, BLOC1S6, BLOC1S6, BLOC1S6, BLOC1S6, BLOC1S6, BLOC1S6, BLOC1S6, BLOC1S6, CCDC6, EML4, EML4, FAM72B, FAM72B, FAM72B, FAM72B, LDLRAD3, LDLRAD3, LDLRAD3, LDLRAD3, MACROD2, MACROD2, MACROD2, MACROD2, MACROD2, MACROD2, NSD2, NSD2, NSD2, NSD2, PRKAA2, PRKAA2, PRKAA2, PRKAA2, PRKX, PRKX, PRKX, RPS6KA3, SCP2, SCP2, SCP2, SCP2, SKI, SOX5, SOX5, TAOK1, TAOK1, TAOK1, TAOK1, TAOK1, TAOK1, TMX4, TMX4, UBA6, UBA6, UBA6, UBA6, UBA6, UBA6, UBA6, UBA6, ZMYND11, ZMYND11, ZMYND11, ZMYND11, ZMYND11, ZMYND11, ZMYND11, ZMYND11, ZMYND11, ZMYND11, ZMYND11, ZMYND11, ZMYND11, ZMYND11, ZMYND11, ZMYND11, ZMYND11	17
hsa-miR-93-5p	ANO6, ANO6, ANO6, ANO6, ANO6, ANO6, BNIP3L, CELSR2, CMPK1, CREB5, CREB5, CREB5, CREB5, IP6K1, IP6K1, IP6K1, ITGB8, ITGB8, ITGB8, NCEH1, NCEH1, NCEH1, NSD2, NSD2, NSD2, NSD2, NSD2, NSD2, NSD2, NSD2, NSD2, NSD2, NSD2, NSD2, NSD2, NSD2, NSD2, PCNX1, PCNX1, PCNX1, SCML2, SCML2, SCML2, SKI, TAOK1, TAOK1, TAOK1, TAOK1, TAOK1, TAOK1, TAOK1, TAOK1, TAOK1, TAOK1, TAOK1, TAOK1, TAOK1, TAOK1, TAOK1, TCF7L1, TCF7L1, TCF7L1, TNRC6A, TNRC6A, TNRC6A, USP24, WNK3, WNK3, WNK3, WNK3, WNK3, WNK3	17
hsa-miR-302c-3p	ANO6, ANO6, ANO6, ARHGAP29, BNIP3L, BNIP3L, BNIP3L, BNIP3L, BNIP3L, BNIP3L, CMPK1, CMPK1, CMPK1, CMPK1, CREB5, CREB5, CREB5, CREB5, CREB5, CREB5, CREB5, CREB5, CREB5, CREB5, CREB5, CREB5, FAM177A1, FAM177A1, FAM177A1, FAM177A1, FAM177A1, FAM177A1, ITGB8, ITGB8, ITGB8, ITGB8, ITGB8, ITGB8, KDM1B, KDM1B, KDM1B, KDM1B, KDM1B, NSD2, NSD2, NSD2, NSD2, NSD2, NSD2, NSD2, NSD2, NSD2, NSD2, NSD2, NSD2, NSD2, NSD2, NSD2, NSD2, RPS6KA3, RPS6KA3, SIRPA, TAOK1, TAOK1, TAOK1, TAOK1, TAOK1, TCF7L1, TVP23B, TVP23B, TVP23B, USP24, ZMYND11, ZMYND11, ZMYND11, ZMYND11, ZMYND11, ZMYND11, ZMYND11, ZMYND11, ZMYND11	16

hsa-miR-520a-3p	ANO6, ANO6, ANO6, BNIP3L, BNIP3L, BNIP3L, BNIP3L, CCDC6, CREB5, CREB5, FAM177A1, FAM177A1, FAM177A1, FAM177A1, FMNL3, FMNL3, FMNL3, FMNL3, FMNL3, FMNL3, IP6K1, IP6K1, IP6K1, ITGB8, ITGB8, ITGB8, ITGB8, KDM1B, KDM1B, KDM1B, KDM1B, KDM1B, KDM1B, LIMA1, LIMA1, LIMA1, LIMA1, LIMA1, LIMA1, LIMA1, LIMA1, LIMA1, LIMA1, LIMA1, LIMA1, NSD2, SIRPA, SIRPA, SIRPA, SIRPA, SIRPA, SIRPA, SIRPA, SIRPA, SIRPA, SIRPA, SIRPA, SIRPA, SIRPA, SIRPA, SIRPA, SIRPA, TAOK1, TAOK1, TAOK1, TCF7L1, TCF7L1, TVP23B, TVP23B, TVP23B, ZMYND11, ZMYND11, ZMYND11, ZMYND11, ZMYND11, ZMYND11, ZMYND11, ZMYND11, ZMYND11	16
hsa-miR-9-5p	ANO6, ANO6, ANO6, ANO6, ANO6, ANO6, ANO6, ANO6, ARF6, ATF1, BMPER, BMPER, BMPER, BMPER, BMPER, BMPER, BMPER, BMPER, CCDC6, CCDC6, CCDC6, CCDC6, CCDC6, CCDC6, CCDC6, CCDC6, CCDC6, CCDC6, CCDC6, CCDC6, CREB5, CREB5, EHD3, EHD3, LDLRAD3, LDLRAD3, LDLRAD3, LDLRAD3, NEK7, NRP1, NRP1, NRP1, NRP1, NRP1, NRP1, NRP1, NRP1, NRP1, NRP1, RANBP2, RANBP2, RBM24, RBM24, RBM24, RBM24, RBM24, RBM24, RBM24, RBM24, RBM24, RNF24, RNF24, RNF24, RNF24, RNF24, RNF24, RNF24, RNF24, SH3GLB1, SH3GLB1, SH3GLB1, SH3GLB1, SH3GLB1, SH3GLB1, SHROOM2, SHROOM2, SHROOM2, SHROOM2, SHROOM2, SHROOM2, SHROOM2, SHROOM2, TNRC6A, TNRC6A, TNRC6A, TNRC6A, TNRC6A, TNRC6A, TNRC6A	16
hsa-miR-27a-3p	ADORA2B, ANTXR2, EHD3, GSPT1, HIC1, HIC1, HIC1, HIC1, ITGB8, LASP1, LASP1, LASP1, MAD2L1BP, MAD2L1BP, MPP7, MPP7, PCNX1, PCNX1, PCNX1, PCNX1, PCNX1, PCNX1, PRKAA2, PRKAA2, PTCHD1, PTCHD1, PTCHD1, RNF144A, TAOK1, TAOK1, TAOK1, TAOK1, TMX4, TMX4	15
hsa-miR-302b-3p	ANO6, ANO6, ANO6, ARHGAP29, BNIP3L, BNIP3L, BNIP3L, BNIP3L, BNIP3L, BNIP3L, CMPK1, CMPK1, CMPK1, CMPK1, CREB5, CREB5, CREB5, CREB5, CREB5, CREB5, FAM177A1, FAM177A1, FAM177A1, FAM177A1, ITGB8, ITGB8, ITGB8, ITGB8, KDM1B, KDM1B, KDM1B, KDM1B, KDM1B, NSD2, NSD2, NSD2, NSD2, NSD2, NSD2, NSD2, NSD2, NSD2, NSD2, SIRPA, ST7L, ST7L, TAOK1, TAOK1, TAOK1, TAOK1, TCF7L1, TVP23B, TVP23B, TVP23B, ZMYND11, ZMYND11, ZMYND11, ZMYND11, ZMYND11, ZMYND11, ZMYND11, ZMYND11, ZMYND11	15
hsa-miR-423-5p	ANO6, ANO6, ANO6, ANO6, ANO6, ANO6, CELSR2, CELSR2, CELSR2, CELSR2, CELSR2, CELSR2, CELSR2, CELSR2, CELSR2, CELSR2, CELSR2, CELSR2, CELSR2, CELSR2, CELSR2, FMNL3, FMNL3, FMNL3, FMNL3, FMNL3, FMNL3, LASP1, MLST8, MLST8, MLST8, MLST8, MLST8, MLST8, MNT, MOCS1, MPPED2, NPR3, NPR3, NPR3, NPR3, OGDH, OGDH, PER1, RNF216, RNF216, RNF216, RNF216, RNF216, RNF216, RNF216, RNF216, SIRPA, SIRPA, SIRPA, SIRPA, SIRPA, SIRPA, SKI, SLC25A44, SLC25A44, SLC25A44, SLC25A44	15
hsa-miR-519d-3p	ANO6, ANO6, ANO6, BNIP3L, BNIP3L, BNIP3L, BNIP3L, CELSR2, CMPK1, CREB5, FAM168A, FAM168A, FAM177A1, FAM177A1, FAM177A1, FAM177A1, ITGB8, ITGB8, ITGB8, NSD2, NSD2, NSD2, NSD2, NSD2, NSD2, NSD2, NSD2, NSD2, NSD2, NSD2, NSD2, NSD2, NSD2, NSD2, NSD2, NSD2, SKI, TAOK1, TAOK1, TAOK1, TAOK1, TAOK1, TAOK1, TAOK1, TAOK1, TAOK1, TNRC6A, TNRC6A, TNRC6A, TNRC6A, TNRC6A, TNRC6A, TNRC6A, TNRC6A, TNRC6A, TVP23B, TVP23B, TVP23B, WNK3, WNK3, WNK3, WNK3, WNK3, WNK3, ZNF320, ZNF320, ZNF320, ZNF320, ZNF320	15
hsa-miR-124-3p	CMPK1, CMPK1, CSDE1, CSDE1, CSDE1, CSDE1, CSDE1, CSDE1, CSDE1, CSDE1, CTNS, CTNS, CTNS, CTNS, CTNS, FAM177A1, FAM177A1, LGSN, LGSN, MOCS1, MOCS1, NRP1, PCNX1, PCNX1, PCNX1, RBM24, RBM24, RPS6KA3, SKI, TAOK1, TAOK1, ZMPSTE24, ZMPSTE24, ZNF561, ZNF561	14
hsa-miR-128-3p	ADORA2B, ADORA2B, FAM177A1, FAM177A1, FBXO33, GSPT1, HIC1, HIC1, HIC1, HIC1, HIC1, HIC1, HIC1, HIC1, HIC1, INSM1, LASP1, LASP1, LASP1, LASP1, LDLRAD3, LDLRAD3, MNT, MNT, MPPED2, PCNX1, PCNX1, RARB, RARB, RARB, RNF144A, UBA6	14

hsa-miR-1321	ARL6IP5, ARL6IP5, CREB3L1, CREB3L1, CREB3L1, CREB3L1, CREB3L1, CREB3L1, FMNL3, FMNL3, FMNL3, FMNL3, GINS1, GINS1, LASP1, MLST8, MLST8, MLST8, MLST8, MLST8, MLST8, MLST8, MLST8, MLST8, MLST8, MLST8, MLST8, MNT, MNT, MNT, NDUFS7, NPR3, NPR3, SKI, SKI, SKI, SKI, SKI, SKI, SKI, SKI, SKI, SKI, SKI, SKI, SKI, SKI, SKI, SKI, SKI, SKI, SKI, SKI, SKI, SKI, SKI, SKI, SKI, SKI, SKI, SKI, SLC25A44, SLC25A44, SLC25A44, SLC25A44, SLC25A44, SLC25A44, SLC25A44, SLC25A44, TAOK1, TAOK1, TJAP1, TJAP1, TJAP1, TJAP1, TJAP1, TJAP1, TJAP1, TJAP1, TJAP1, TJAP1, UBQLN2, UBQLN2, UBQLN2, UBQLN2	14
hsa-miR-1323	CDK5R1, CDK5R1, CDK5R1, CDK5R1, CDK5R1, CDK5R1, CMPK1, CMPK1, CMPK1, CMPK1, CMPK1, CMPK1, CMPK1, CMPK1, CMPK1, CMPK1, CMPK1, CMPK1, CSDE1, CSDE1, CSDE1, CSDE1, CSDE1, CSDE1, CSDE1, EEPD1, EEPD1, EEPD1, EML4, EML4, EML4, EML4, EML4, EML4, EML4, EML4, FAM168A, FAM168A, FAM168A, FAM168A, GINS1, GINS1, GINS1, GINS1, GINS1, GINS1, GINS1, GINS1, GINS1, GINS1, GINS1, GINS1, GINS1, GINS1, GINS1, GINS1, GINS1, MPHOSPH8, NPR3, NPR3, NPR3, NPR3, NPR3, NPR3, PRKAA2, PRKAA2, PRKAA2, PRKAA2, PRKAA2, PRKAA2, PRKAA2, PRKAA2, PRKAA2, RNF8, RNF8, RPL15, SOX5, SOX5, SOX5, TAOK1, TAOK1, TAOK1, TAOK1, TAOK1, TAOK1, TAOK1, TAOK1, TAOK1, TAOK1, TAOK1, TAOK1	14
hsa-miR-141-3p	ANO6, ANO6, ANO6, ANO6, ANO6, ANO6, ANO6, ANO6, ANO6, ANO6, ANO6, ANO6, ANO6, ANO6, ANO6, CCDC6, CCDC6, CCDC6, HECTD1, HECTD1, HECTD1, HECTD1, NSD2, NSD2, NSD2, NSD2, NSD2, NSD2, NSD2, NSD2, NSD2, NSD2, NSD2, NSD2, NSD2, PCNX1, PCNX1, POLR1B, POLR1B, POLR1B, POLR1B, POLR1B, POLR1B, POLR1B, POLR1B, POLR1B, POLR1B, RARB, RARB, RARB, SCP2, SCP2, SCP2, SCP2, SOX5, SOX5, STAG1, STAG1, STAG1, STAG1, SUCLA2, SUCLA2, SUCLA2, SUCLA2, SUCLA2, SUCLA2, TNRC6A, TNRC6A, TNRC6A, TNRC6A, TNRC6A, TNRC6A, UBA6, UBA6, UBA6, UBA6, USP9X, USP9X	14
hsa-miR-143-3p	BICD1, BICD1, BICD1, BICD1, BICD1, BICD1, BICD1, BICD1, CREB5, CREB5, EPM2AIP1, EPM2AIP1, FMNL3, FMNL3, FMNL3, FMNL3, LASP1, LASP1, LASP1, NPR3, NPR3, NPR3, NSD2, NSD2, NSD2, NSD2, NSD2, NSD2, PRKX, PRKX, PRKX, RBM24, RBM24, SOX5, TNRC6A, TNRC6A, TTC19, TTC19, TTC19, TTC19, UBXN2B, UBXN2B, UBXN2B, ZWILCH, ZWILCH, ZWILCH	14
hsa-miR-144-3p	ANTXR2, ANTXR2, CALCRL, CHST12, CHST12, CHST12, CHST12, CHST12, CHST12, CHST12, CHST12, COPS2, FMNL3, FMNL3, FMNL3, FMNL3, ITGB8, KLHDC1, MEIS2, MEIS2, MEIS2, MEIS2, NEK7, NEK7, NEK7, NEK7, RARB, RARB, RARB, RARB, RARB, RARB, RARB, RARB, RARB, RARB, RARB, RARB, SUCLA2, SUCLA2, SUCLA2, SUCLA2, TAOK1, TAOK1, TNRC6A, TNRC6A, WNK3, WNK3	14
hsa-miR-200a-3p	ANO6, ANO6, ANO6, ANO6, ANO6, ANO6, ANO6, ANO6, ANO6, ANO6, ANO6, ANO6, ANO6, ANO6, ANO6, CCDC6, CCDC6, CCDC6, CCDC6, CCDC6, CCDC6, HECTD1, HECTD1, HECTD1, HECTD1, NRP1, NSD2, NSD2, NSD2, NSD2, NSD2, NSD2, NSD2, NSD2, POLR1B, POLR1B, POLR1B, POLR1B, POLR1B, RARB, RARB, RARB, SCP2, SCP2, SCP2, SCP2, SOX5, SOX5, STAG1, STAG1, STAG1, STAG1, SUCLA2, SUCLA2, TNRC6A, TNRC6A, TNRC6A, TNRC6A, TNRC6A, TNRC6A, UBA6, UBA6, UBA6, UBA6, USP9X, USP9X	14

hsa-miR-200c-3p	ANLN, ANLN, ANO6, ANO6, ANO6, ANO6, ANO6, ANO6, CHAC2, CHAC2, DAG1, DAG1, DAG1, DAG1, DAG1, DAG1, DAG1, EPM2AIP1, FBXO33, FBXO33, FSCN1, GOLGA8B, GOLGA8B, NSD2, NSD2, NSD2, NSD2, PCNX1, PPIL4, PPIL4, PPIL4, PPIL4, SOX5, SOX5, SOX5, SOX5, SOX5, SOX5, SOX5, SOX5, SOX5, TAOK1, TAOK1, TRIM33, TRIM33, TRIM33, TRIM33	14
hsa-miR-214-3p	ADORA2A, ADORA2A, ADORA2A, ADORA2A, ADORA2A, ADORA2A, ADORA2A, ADORA2A, CELSR2, EXTL3, EXTL3, EXTL3, EXTL3, IP6K1, IP6K1, IP6K1, IP6K1, IP6K1, IP6K1, MNT, MNT, MNT, MOCS1, MOCS1, MOCS1, MOCS1, MOCS1, MOCS1, MOCS1, MOCS1, NOMO1, NOMO1, NOMO1, NOMO1, NSD2, NSD2, NSD2, NSD2, NSD2, NSD2, NSD2, PCNX1, RNF8, RNF8, RNF8, RNF8, RNF8, SCP2, SCP2, SKI, SKI, SKI, SKI, SKI, SKI, TRIM33, TRIM33, TSEN2	14
hsa-miR-23b-3p	ANTXR2, ANTXR2, ANTXR2, BNIP3L, HIC1, HIC1, HIC1, HIC1, MADD, MADD, MADD, MADD, MADD, MADD, MADD, MADD, NEK7, NPR3, NPR3, NSD2, SIRPA, SIRPA, SIRPA, SIRPA, SLC38A1, SMS, TAOK1, TNRC6A, UBA6, UBA6, UBA6, ZNF506, ZNF506, ZNF506	14
hsa-miR-27b-3p	ADORA2B, ANTXR2, EHD3, GSPT1, HIC1, HIC1, HIC1, HIC1, ITGB8, LASP1, LASP1, LASP1, LASP1, LASP1, LASP1, MPP7, MPP7, PCNX1, PCNX1, PCNX1, PCNX1, PCNX1, PCNX1, PCNX1, PRKAA2, PRKAA2, PTCHD1, PTCHD1, PTCHD1, RNF144A, TAOK1, TAOK1, TAOK1, TAOK1, TMX4, TMX4	14
hsa-miR-300	BLOC1S6, CMPK1, CMPK1, DAG1, DAG1, DAG1, DAG1, DAG1, DAG1, DAG1, NRP1, NRP1, NRP1, NRP1, NRP1, RNF144A, SLC38A1, SLC38A1, SLC38A1, SLC38A1, STAG1, TAOK1, TNRC6A, TNRC6A, TNRC6A, TVP23B, TVP23B, UBXN2B, USP24, USP24, USP24, ZNF721, ZNF721, ZWILCH, ZWILCH, ZWILCH, ZWILCH, ZWILCH, ZWILCH, ZWILCH, ZWILCH, ZWILCH, ZWILCH, ZWILCH, ZWILCH	14
hsa-miR-301a-3p	BTBD10, BTBD10, CCDC6, CREB5, CREB5, CXCL6, CXCL6, ITGB8, ITGB8, KCNA4, MPPED2, MPPED2, PCNX1, PCNX1, RARB, RARB, RARB, RPS6KA3, SLC25A44, SLC25A44, SLC25A44, SLC25A44, SLC25A44, SLC25A44, SOX5, SOX5, TNRC6A, TNRC6A, TNRC6A, TNRC6A, TNRC6A, TNRC6A, TNRC6A, TNRC6A, TNRC6A, TNRC6A, WNK3, WNK3	14
hsa-miR-302e	ANO6, ANO6, ANO6, ARHGAP29, BNIP3L, BNIP3L, CMPK1, CMPK1, CREB5, CREB5, CREB5, CREB5, FAM177A1, FAM177A1, IP6K1, IP6K1, IP6K1, ITGB8, ITGB8, ITGB8, ITGB8, KDM1B, KDM1B, KDM1B, KDM1B, KDM1B, KDM1B, NSD2, NSD2, NSD2, NSD2, NSD2, SIRPA, SIRPA, SIRPA, SIRPA, SIRPA, SIRPA, SIRPA, SIRPA, SIRPA, SIRPA, SIRPA, SIRPA, TAOK1, TAOK1, TAOK1, TAOK1, TAOK1, TAOK1, TAOK1, TAOK1, TVP23B, TVP23B, TVP23B, ZMYND11, ZMYND11, ZMYND11, ZMYND11, ZMYND11, ZMYND11, ZMYND11, ZMYND11, ZMYND11	14
hsa-miR-320d	BNIP3L, BNIP3L, BNIP3L, BNIP3L, BNIP3L, BNIP3L, CMPK1, COPS2, COPS2, COPS2, CREB5, GSPT1, GSPT1, NRP1, NRP1, POLR1B, POLR1B, POLR1B, POLR1B, POLR1B, PRKAA2, PRKAA2, RANBP2, RANBP2, RBM24, RBM24, RBM24, RBM24, SMG7, SMG7, SMG7, SMG7, STAMBP, STAMBP, STAMBP, STAMBP, STAMBP, STAMBP, TAOK1, TAOK1, ZWILCH, ZWILCH, ZWILCH	14

hsa-miR-340-5p	ANO6, ANO6, ANO6, ARHGAP29, ARHGAP29, ARHGAP29, ATF1, ATF1, ATF1, ATF1, ATF1, ATF1, ATF1, CALCRL, CALCRL, CALCRL, CALCRL, FAM177A1, FAM177A1, FAM177A1, FAM177A1, FAM177A1, FAM177A1, FANCM, FANCM, FANCM, FANCM, KLHDC1, MYO1C, MYO1C, MYO1C, PCNX1, PCNX1, PCNX1, PCNX1, PCNX1, PCNX1, PRKAA2, RPS6KA3, RPS6KA3, RPS6KA3, RPS6KA3, RPS6KA3, SOX5, TVP23B, TVP23B, TVP23B, TVP23B, TVP23B, TVP23B, TVP23B, TVP23B, TVP23B, TVP23B, TVP23B, TVP23B, UBA6, UBA6	14
hsa-miR-454-3p	CCDC6, CMPK1, CMYA5, CREB5, CREB5, CREB5, CREB5, CXCL6, DSG1, DSG1, DSG1, HECTD1, HECTD1, HECTD1, HECTD1, HECTD1, HECTD1, HECTD1, HECTD1, ITGB8, ITGB8, ITGB8, MPPED2, MPPED2, NRP1, SLC25A44, SLC25A44, SLC25A44, SLC25A44, SLC25A44, SLC25A44, SOX5, SOX5, TNRC6A, TNRC6A, TNRC6A, TNRC6A, TNRC6A, TNRC6A, WNK3, WNK3	14
hsa-miR-522-3p	ARHGAP29, ARHGAP29, ARHGAP29, ARHGAP29, ARHGAP29, ARHGAP29, CMPK1, CMPK1, CMPK1, CREB5, CXCL6, DSG1, DSG1, FOXA1, FOXA1, KCNA1, LSM5, LSM5, NSD2, NSD2, NSD2, NSD2, NSD2, NSD2, NSD2, NSD2, PCNX1, PCNX1, SIKE1, SIKE1, SIKE1, SIKE1, SIKE1, SIKE1, TAOK1, TAOK1, TNRC6A, UBA6, UBA6, UBA6, UBA6	14
hsa-miR-1252-5p	CTNS, CTNS, CTNS, FMNL3, FMNL3, FMNL3, FMNL3, FMNL3, FMNL3, LIMA1, LIMA1, LIMA1, LIMA1, LIMA1, LIMA1, NSD2, NSD2, NSD2, NSD2, NSD2, NSD2, NSD2, NSD2, NSD2, NSD2, PRKX, PRKX, RNF144A, RNF144A, RNF144A, RNF144A, RNF144A, RNF144A, STAMBP, SZT2, SZT2, SZT2, SZT2, TMX4, TMX4, TMX4, TPM4, UBA6, UBA6, UBA6, UBA6, UBA6, UBA6, UBA6, UBA6, UBQLN2, UBQLN2, UBQLN2, UBQLN2, ZMPSTE24	13
hsa-miR-145-5p	ANO6, ANO6, ANO6, ANO6, ANO6, ANO6, ARF6, ARF6, ARF6, ARF6, ARF6, ARF6, ARF6, ARF6, ARHGAP29, ATF1, DAG1, DAG1, DAG1, DAG1, DAG1, DAG1, FSCN1, FSCN1, FSCN1, FSCN1, FSCN1, FSCN1, FSCN1, FSCN1, ITGB8, ITGB8, ITGB8, ITGB8, ITGB8, ITGB8, ITGB8, ITGB8, ITGB8, PRKX, PRPSAP2, PRPSAP2, PRPSAP2, PRPSAP2, PRPSAP2, SIRPA, SIRPA, SIRPA, SIRPA, SIRPA, SIRPA, SIRPA, SIRPA, TAOK1, TAOK1, TAOK1, TAOK1, TAOK1, TAOK1, TPM4, TPM4, TPM4, TPM4, TPM4, TPM4, UBA6, UBA6	13
hsa-miR-181b-5p	ARF6, ARF6, BLOC1S6, BLOC1S6, BLOC1S6, BLOC1S6, BLOC1S6, BLOC1S6, CALCRL, CALCRL, CALCRL, CCDC6, CCDC6, COPS2, COPS2, CREB5, CREB5, CREB5, CREB5, CREB5, CREB5, CREB5, CREB5, CREB5, CREB5, FBXO33, FBXO33, FBXO33, ITGB8, ITGB8, ITGB8, ITGB8, MADD, MADD, MADD, MADD, MADD, MADD, MADD, MADD, NEK7, NEK7, SMG7, SMG7, SMG7, SMG7, SOX5, SOX5, SOX5, SOX5, SOX5, SOX5, TAOK1, TAOK1	13

hsa-miR-24-3p	BNIP3L, FSCN1, HIC1, HIC1, HIC1, MNT, MNT, MNT, MNT, NRP1, NRP1, NSD2, NSD2, NSD2, NSD2, NSD2, NSD2, NSD2, NSD2, NSD2, NSD2, NSD2, NSD2, NSD2, NSD2, NSD2, NSD2, RBBP4, RBBP4, SHROOM2, SHROOM2, SHROOM2, SHROOM2, SHROOM2, SHROOM2, SIRPA, SLC39A3, STAT2, STAT2, STAT2, TAOK1, TRIM33, TRIM33	13
hsa-miR-30a-5p	ATF1, BNIP3L, BNIP3L, BNIP3L, BNIP3L, EML4, KCTD7, KCTD7, KCTD7, KCTD7, KCTD7, KCTD7, MEIS2, MEIS2, NRP1, NRP1, PRKAA2, PRKAA2, PRKAA2, PRKAA2, PRKAA2, PRKAA2, PRKAA2, PRKAA2, PRKAA2, PRKAA2, PRKAA2, PRKAA2, RARB, RARB, RARB, RARB, RARB, RARB, SCN8A, SCN8A, SCN8A, SCN8A, SCN8A, TAOK1, TAOK1, TAOK1, TAOK1, TAOK1, TAOK1, TAOK1, TAOK1, TAOK1, TAOK1, TAOK1, TAOK1, TNRC6A, TVP23B, TVP23B, TVP23B, WNK3, WNK3, WNK3, WNK3	13
hsa-miR-320c	BNIP3L, BNIP3L, BNIP3L, BNIP3L, CMPK1, COPS2, COPS2, COPS2, CREB5, GSPT1, NRP1, NRP1, POLR1B, POLR1B, POLR1B, POLR1B, POLR1B, RANBP2, RANBP2, RBM24, RBM24, RBM24, RBM24, SMG7, SMG7, SMG7, SMG7, STAMBP, STAMBP, STAMBP, STAMBP, STAMBP, STAMBP, TAOK1, TAOK1, ZWILCH, ZWILCH, ZWILCH	13
hsa-miR-520d-5p	ATF1, ATF1, ATF1, ATF1, CREB5, CREB5, CREB5, CREB5, CREB5, CREB5, GINS1, GINS1, GINS1, GINS1, GINS1, GINS1, GINS1, GINS1, GINS1, GINS1, GINS1, GINS1, GINS1, GINS1, GSPT1, GSPT1, GSPT1, GSPT1, NRP1, NRP1, NRP1, NRP1, NRP1, NRP1, NRP1, NRP1, NRP1, NRP1, NRP1, NRP1, NRP1, NRP1, NRP1, NRP1, NRP1, NRP1, NRP1, NSD2, NSD2, NSD2, NSD2, NSD2, NSD2, NSD2, NSD2, NSD2, RANBP2, RANBP2, RANBP2, RANBP2, SIRPA, SIRPA, SVIP, SVIP, TAOK1, TAOK1, TAOK1, TAOK1, TAOK1, TAOK1, TRIM33, TRIM33, TRIM33, TRIM33, TRIM33, TRIM33, TRIM33, TRIM33, UBQLN2, UBQLN2, UBQLN2, USP24, USP24, USP24, USP24	13
hsa-miR-524-5p	ATF1, ATF1, ATF1, ATF1, CREB5, CREB5, CREB5, CREB5, EPM2AIP1, EPM2AIP1, EPM2AIP1, GINS1, GINS1, GINS1, GINS1, GINS1, GINS1, GINS1, GSPT1, GSPT1, GSPT1, GSPT1, NRP1, RNF216, RNF216, SIRPA, SIRPA, STAMBP, STAMBP, STAMBP, STAMBP, STAMBP, STAMBP, STAMBP, STAMBP, STAMBP, STAMBP, STAMBP, STAMBP, SVIP, SVIP, TAOK1, TRIM33, TRIM33, TRIM33, TRIM33, USP24, USP24, USP24, USP24	13
hsa-miR-526b-3p	ANO6, ANO6, ANO6, BNIP3L, BTBD10, CMPK1, CREB5, CREB5, CREB5, CREB5, CREB5, CREB5, CREB5, CREB5, FAM168A, FAM168A, FAM168A, FAM168A, ITGB8, ITGB8, ITGB8, ITGB8, ITGB8, ITGB8, TAOK1, TAOK1, TAOK1, TAOK1, TCF7L1, TCF7L1, TNRC6A, TNRC6A, TNRC6A, TVP23B, TVP23B, TVP23B, USP24, WNK3, WNK3	13
hsa-miR-96-5p	ANLN, ANLN, ANLN, ANLN, ANTXR2, ARHGAP29, CELSR2, EOMES, EOMES, EOMES, EOMES, EPM2AIP1, KCTD7, KCTD7, KCTD7, KCTD7, KCTD7, KCTD7, MYO1C, MYO1C, MYO1C, MYO1C, MYO1C, MYO1C, NEK7, OGDH, OGDH, PLPPR4, PLPPR4, PLPPR4, PLPPR4, SIKE1, SIKE1, SIKE1, SIKE1, SIKE1, SIKE1, SIKE1, SIKE1, TNRC6A, TNRC6A, TNRC6A, TNRC6A, TNRC6A	13
hsa-miR-1-3p	BICD1, FBXO33, LASP1, MED1, MED1, MED1, MPP7, MPP7, NCK2, NCK2, NCK2, NRP1, NRP1, NRP1, NRP1, PRKX, SMG7, SMG7, SMG7, SMG7, TPM4, TPM4, TPM4, TPM4, TPM4, TPM4, TPM4, TPM4, TPM4, TPM4, TPM4, TPM4, TPM4, TPM4, TPM4, TPM4, TPM4, TPM4, UNC50, UNC50, UNC50, UNC50, UNC50, WDR6, WDR6, WDR6, WDR6	12

hsa-miR-130b-3p	CMPK1, CREB5, CREB5, CREB5, CREB5, CXCL6, DSG1, DSG1, DSG1, DSG1, ITGB8, ITGB8, ITGB8, KCNA4, MPPED2, MPPED2, MPPED2, RBM24, RPS6KA3, SLC25A44, SLC25A44, SLC25A44, SLC25A44, SLC25A44, SLC25A44, TNRC6A, TNRC6A, TNRC6A, TNRC6A, TNRC6A, TNRC6A, WNK3, WNK3	12
hsa-miR-181a-5p	ARF6, ARF6, BLOC1S6, BLOC1S6, BLOC1S6, BLOC1S6, BLOC1S6, BLOC1S6, BLOC1S6, BLOC1S6, BLOC1S6, BLOC1S6, BLOC1S6, BLOC1S6, CALCRL, CALCRL, CALCRL, CCDC6, CREB5, CREB5, CREB5, CREB5, CREB5, CREB5, CREB5, FBXO33, FBXO33, FBXO33, ITGB8, ITGB8, ITGB8, ITGB8, MADD, MADD, MADD, MADD, MADD, MADD, MADD, MADD, NEK7, NEK7, SMG7, SMG7, SMG7, SMG7, SOX5, SOX5, SOX5, SOX5, SOX5, SOX5, SOX5, SOX5, TAOK1, TAOK1	12
hsa-miR-181d-5p	ARF6, ARF6, BLOC1S6, BLOC1S6, BLOC1S6, BLOC1S6, BLOC1S6, BLOC1S6, CALCRL, CALCRL, CCDC6, CCDC6, COPS2, COPS2, CREB5, CREB5, CREB5, CREB5, CREB5, CREB5, CREB5, CREB5, CREB5, CREB5, FBXO33, FBXO33, FBXO33, ITGB8, ITGB8, MADD, MADD, MADD, MADD, MADD, MADD, MADD, MADD, NEK7, SMG7, SMG7, SMG7, SMG7, SOX5, SOX5, SOX5, SOX5	12
hsa-miR-185-5p	BICD1, BICD1, BICD1, BICD1, BICD1, CELSR2, CELSR2, CELSR2, CELSR2, CELSR2, CELSR2, CELSR2, CELSR2, CTNS, CTNS, CTNS, CTNS, CTNS, CTNS, CTNS, CTNS, CTNS, CTNS, CTNS, CTNS, CTNS, CTNS, CTNS, CTNS, FAM168A, FAM168A, FAM168A, FAM168A, FAM168A, FMNL3, GINS1, GINS1, GINS1, GINS1, LASP1, LASP1, LASP1, LASP1, LASP1, LASP1, LASP1, LASP1, LASP1, LASP1, LASP1, LASP1, NRP1, NRP1, NRP1, NRP1, NRP1, NRP1, NRP1, NRP1, RNF24, RNF24, RNF24, RNF24, RNF24, RNF24, RNF24, SIRPA, SIRPA, SIRPA, SIRPA, SIRPA, SIRPA, SIRPA, SIRPA, SIRPA, SIRPA, SIRPA, SIRPA, SIRPA, SIRPA, SIRPA, SIRPA, SMG7, TNRC6A, TNRC6A, TNRC6A, TNRC6A, TNRC6A, TNRC6A, TNRC6A, TNRC6A, TNRC6A	12
hsa-miR-19b-3p	CCDC6, DAG1, DAG1, DAG1, DAG1, DAG1, DAG1, HIC1, HIC1, HIC1, ITGB8, ITGB8, ITGB8, ITGB8, KCNA4, MPPED2, MPPED2, SOX5, SOX5, SOX5, SOX5, TAOK1, TAOK1, TAOK1, TAOK1, TAOK1, TAOK1, TRIM33, USP24, ZMYND11, ZNF320, ZNF320, ZNF320	12
hsa-miR-301b-3p	CMPK1, CREB5, CREB5, CREB5, CREB5, CXCL6, ITGB8, ITGB8, ITGB8, KCNA4, MPPED2, MPPED2, PCNX1, PCNX1, RARB, RARB, RARB, SLC25A44, SLC25A44, SLC25A44, SLC25A44, SLC25A44, SLC25A44, SOX5, SOX5, TNRC6A, TNRC6A, TNRC6A, TNRC6A, TNRC6A, TNRC6A, TNRC6A, TNRC6A, TNRC6A, TNRC6A, WNK3, WNK3, WNK3, WNK3	12
hsa-miR-30d-5p	ATF1, EML4, EML4, EML4, EML4, KCTD7, KCTD7, KCTD7, KCTD7, KCTD7, KCTD7, MEIS2, MEIS2, NRP1, NRP1, PRKAA2, PRKAA2, PRKAA2, PRKAA2, PRKAA2, PRKAA2, SCN8A, SCN8A, SCN8A, SCN8A, SCN8A, TAOK1, TAOK1, TAOK1, TAOK1, TAOK1, TAOK1, TAOK1, TAOK1, TNRC6A, TNRC6A, TNRC6A, TNRC6A, TNRC6A, TNRC6A, TNRC6A, TNRC6A, TNRC6A, TNRC6A, TNRC6A, TNRC6A, TNRC6A, TNRC6A, TNRC6A, TNRC6A,	12

	TNRC6A, TNRC6A, TNRC6A, TNRC6A, TNRC6A, TNRC6A, TNRC6A, TNRC6A, TNRC6A, TNRC6A, TNRC6A, TNRC6A, TNRC6A, TNRC6A, TNRC6A, TNRC6A, TVP23B, TVP23B, TVP23B, WNK3, WNK3, WNK3, WNK3, WNK3, WNK3, WNK3, WNK3, ZSCAN29	
hsa-miR-320b	BNIP3L, BNIP3L, BNIP3L, BNIP3L, BNIP3L, BNIP3L, COPS2, COPS2, COPS2, CREB5, GSPT1, GSPT1, NRP1, NRP1, POLR1B, POLR1B, POLR1B, POLR1B, POLR1B, RANBP2, RANBP2, RBM24, RBM24, RBM24, RBM24, SMG7, SMG7, SMG7, SMG7, STAMBP, STAMBP, STAMBP, STAMBP, STAMBP, STAMBP, TAOK1, TAOK1, ZWILCH, ZWILCH, ZWILCH	12
hsa-miR-330-3p	ANTXR2, ANTXR2, CMPK1, CMPK1, CMPK1, CMPK1, CMPK1, CMPK1, CMPK1, CMPK1, CMPK1, CMPK1, CMPK1, CMPK1, DAG1, FAM177A1, FAM177A1, NCEH1, NCEH1, NCEH1, NCEH1, NEK7, NEK7, NPR3, NPR3, NPR3, NPR3, NPR3, NPR3, PRKAA2, PRKAA2, PRKAA2, PRKAA2, PRKAA2, PRKAA2, PRKAA2, PRKAA2, PRKX, PRKX, SCP2, SCP2, SCP2, SCP2, SCP2, SCP2, SCP2, SCP2, SCP2, SCP2, SCP2, SCP2, SCP2, SMG7, SMG7, SMG7, SMG7, SMG7, SMG7, SMG7, TVP23B, TVP23B, TVP23B, TVP23B, TVP23B, TVP23B	12
hsa-miR-506-3p	ANTXR2, ANTXR2, ANTXR2, BLOC1S6, CMPK1, CMPK1, CTNS, CTNS, CTNS, CTNS, CTNS, FAM177A1, FAM177A1, MOCS1, MOCS1, MOCS1, MOCS1, NRP1, PCNX1, PCNX1, PCNX1, RBM24, RBM24, TAOK1, TAOK1, ZMPSTE24, ZMPSTE24, ZNF561, ZNF561, ZNF561, ZNF561	12
hsa-miR-519b-3p	ARHGAP29, ARHGAP29, CELSR2, CMPK1, CMPK1, CREB5, CREB5, CREB5, CXCL6, FANCM, FANCM, FANCM, FANCM, FANCM, FMNL3, NSD2, NSD2, NSD2, NSD2, NSD2, NSD2, NSD2, NSD2, SIKE1, SIKE1, SIKE1, SIKE1, SKI, TAOK1, TAOK1, WNK3, WNK3, WNK3, WNK3, WNK3, WNK3	12
hsa-miR-519c-3p	ARHGAP29, ARHGAP29, ARHGAP29, ARHGAP29, CELSR2, CELSR2, CMPK1, CMPK1, CREB5, CREB5, CREB5, CREB5, CREB5, CREB5, CXCL6, FANCM, FANCM, FANCM, FANCM, FANCM, SIKE1, SIKE1, SKI, TAOK1, TAOK1, TAOK1, TAOK1, TAOK1, TAOK1, TNRC6A, TNRC6A, TNRC6A, UBA6, UBA6, WNK3, WNK3, WNK3, WNK3, WNK3, WNK3	12
hsa-miR-520f-3p	ANO6, ANO6, ANO6, ARF6, CCDC6, CCDC6, CCDC6, CHMP3, CHMP3, CHMP3, CREB5, CREB5, CREB5, CREB5, CREB5, CREB5, ITGB8, ITGB8, ITGB8, ITGB8, ITGB8, ITGB8, KDM1B, KDM1B, KDM1B, KDM1B, KDM1B, KDM1B, KDM1B, KDM1B, KDM1B, KDM1B, KDM1B, KDM1B, KDM1B, KDM1B, KDM1B, KDM1B, RBM24, RBM24, RBM24, RPS6KA3, RPS6KA3, SIRPA, SIRPA, SIRPA, SIRPA, SIRPA, SIRPA, TAOK1, TAOK1, TAOK1, TAOK1, ZMYND11, ZMYND11, ZMYND11, ZMYND11, ZMYND11, ZMYND11, ZMYND11, ZMYND11, ZMYND11, ZMYND11, ZMYND11, ZMYND11, ZMYND11, ZMYND11, ZMYND11, ZMYND11, ZMYND11, ZMYND11	12
hsa-miR-543	ATF1, CMPK1, CMPK1, CMPK1, CREB5, CREB5, CREB5, CREB5, CREB5, CREB5, CREB5, CREB5, CREB5, CREB5, CREB5, CREB5, CREB5, CREB5, CREB5, CREB5, CREB5, CREB5, FANCM, FANCM, FANCM, HEPH, HEPH, HEPH, HEPH, HEPH, HEPH, ITGB8, MEIS2, MEIS2, NEK7, NRP1, NRP1, PRKAA2, PRKAA2, PRKAA2, PRKAA2, PRKAA2, SCP2, SCP2, SCP2, SCP2, SH3GLB1, SH3GLB1, SH3GLB1	12

hsa-miR-19a-3p	CCDC6, DAG1, DAG1, DAG1, DAG1, DAG1, DAG1, HIC1, HIC1, HIC1, ITGB8, ITGB8, ITGB8, ITGB8, MPPED2, MPPED2, SOX5, SOX5, SOX5, SOX5, TAOK1, TAOK1, TAOK1, TAOK1, TAOK1, TAOK1, TRIM33, TRIM33, USP24, ZMYND11, ZNF320, ZNF320, ZNF320, ZNF320, ZNF320	11
hsa-miR-23a-3p	ANTXR2, ANTXR2, ANTXR2, BNIP3L, HIC1, HIC1, HIC1, HIC1, MADD, MADD, MADD, MADD, MADD, MADD, MADD, MADD, NEK7, NSD2, SIRPA, SIRPA, SIRPA, SIRPA, SMS, TNRC6A, TNRC6A, TNRC6A, TNRC6A, TNRC6A, TNRC6A, TNRC6A, TNRC6A, TNRC6A, TNRC6A, TNRC6A, TNRC6A, TNRC6A, TNRC6A, TNRC6A, UBA6, UBA6, UBA6, ZNF506, ZNF506, ZNF506	11
hsa-miR-26a-5p	ARF6, ARF6, EPM2AIP1, EPM2AIP1, EPM2AIP1, ITGB8, ITGB8, NSD2, NSD2, NSD2, NSD2, NSD2, NSD2, NSD2, NSD2, PCNX1, PCNX1, PRKAA2, PRKAA2, PRKAA2, PRKAA2, TAOK1, TNRC6A, TNRC6A, TNRC6A, TRPC4, TRPC4, TRPC4, USP9X, USP9X, USP9X, USP9X, ZNF286A, ZNF286A, ZNF286A, ZNF286A, ZNF286A, ZNF286A, ZNF286A, ZNF286A, ZNF286A, ZNF286A	11
hsa-miR-26b-5p	ARF6, ARF6, EPM2AIP1, EPM2AIP1, EPM2AIP1, EPM2AIP1, EPM2AIP1, EPM2AIP1, ITGB8, ITGB8, NSD2, NSD2, NSD2, NSD2, NSD2, NSD2, NSD2, NSD2, PCNX1, PCNX1, PRKAA2, PRKAA2, PRKAA2, PRKAA2, TAOK1, TNRC6A, TNRC6A, TNRC6A, TRIM33, USP9X, USP9X, USP9X, USP9X, ZNF286A, ZNF286A, ZNF286A, ZNF286A, ZNF286A, ZNF286A, ZNF286A, ZNF286A, ZNF286A, ZNF286A	11
hsa-miR-29a-3p	CMPK1, CMPK1, CREB5, CREB5, CREB5, CREB5, CREB5, CREB5, CREB5, CREB5, CREB5, CREB5, CREB5, CREB5, DAG1, EML4, EML4, EML4, EML4, EML4, EML4, LASP1, LASP1, LASP1, LASP1, LASP1, LASP1, NSD2, NSD2, NSD2, NSD2, NSD2, NSD2, NSD2, NSD2, NSD2, NSD2, NSD2, NSD2, SH3GLB1, SH3GLB1, SH3GLB1, SHROOM2, SHROOM2, SHROOM2, SHROOM2, SIKE1, SIKE1, SKI, SMS, SMS	11
hsa-miR-30c-5p	ATF1, BNIP3L, BNIP3L, BNIP3L, BNIP3L, KCTD7, KCTD7, KCTD7, KCTD7, KCTD7, KCTD7, NRP1, NRP1, PRKAA2, PRKAA2, PRKAA2, PRKAA2, PRKAA2, PRKAA2, PRKAA2, PRKAA2, PRKAA2, PRKAA2, PRKAA2, PRKAA2, RARB, RARB, RARB, RARB, RARB, RARB, SCN8A, SCN8A, SCN8A, SCN8A, SCN8A, SCN8A, SCN8A, SCN8A, STRIP1, STRIP1, TAOK1, TAOK1, TAOK1, TAOK1, TAOK1, TAOK1, TAOK1, TAOK1, TAOK1, TAOK1, TAOK1, TAOK1, TNRC6A, TRIM33	11
hsa-miR-30e-5p	ATF1, BNIP3L, BNIP3L, BNIP3L, BNIP3L, EML4, EML4, MEIS2, MEIS2, PRKAA2, PRKAA2, PRKAA2, PRKAA2, PRKAA2, PRKAA2, RARB, RARB, RARB, RARB, RARB, RARB, RARB, RARB, RARB, RARB, RARB, RARB, SCN8A, SCN8A, SCN8A, SCN8A, SCN8A, TAOK1, TAOK1, TAOK1, TAOK1, TAOK1, TAOK1, TAOK1, TAOK1, TAOK1, TNRC6A, TVP23B, TVP23B, TVP23B, WNK3, WNK3, WNK3, WNK3	11

hsa-miR-326	BSDC1, BSDC1, BSDC1, BSDC1, BSDC1, BSDC1, BSDC1, BSDC1, BSDC1, BSDC1, BSDC1, BSDC1, BSDC1, BSDC1, BSDC1, BSDC1, BSDC1, BSDC1, CDK5R1, CDK5R1, DAG1, FAM168A, FAM168A, FAM168A, FAM168A, FAM168A, FAM168A, FAM168A, FAM168A, ITGB8, ITGB8, LASP1, LASP1, LASP1, LASP1, LASP1, LASP1, LASP1, LASP1, LASP1, MNT, MNT, NRP1, PCNX1, PCNX1, PCNX1, PCNX1, PCNX1, PCNX1, PCNX1, PCNX1, POLE, POLE, SIRPA, SIRPA, SIRPA, SIRPA	11
hsa-miR-34c-5p	CHST12, CHST12, CHST12, CHST12, CHST12, CHST12, CHST12, CHST12, CREB5, CREB5, CREB5, CREB5, FMNL3, FMNL3, ITGB8, MPPED2, MPPED2, MYO1C, MYO1C, MYO1C, MYO1C, SVIP, TAOK1, TAOK1, TTC19, TTC19, TTC19, USP24, USP24, USP24, ZMYND11, ZMYND11, ZMYND11, ZMYND11, ZMYND11, ZMYND11, ZMYND11, ZMYND11, ZMYND11, ZMYND11, ZMYND11, ZMYND11, ZMYND11, ZMYND11, ZMYND11, ZMYND11, ZMYND11, ZMYND11	11
hsa-miR-381-3p	BLOC1S6, CMPK1, CMPK1, DAG1, DAG1, DAG1, DAG1, DAG1, DAG1, DAG1, RNF144A, RNF144A, RPS6KA3, RPS6KA3, SLC38A1, SLC38A1, SLC38A1, SLC38A1, TAOK1, TAOK1, TAOK1, TAOK1, TAOK1, TAOK1, TAOK1, TAOK1, TAOK1, TAOK1, TAOK1, TAOK1, TAOK1, TAOK1, TAOK1, TAOK1, TAOK1, TAOK1, TAOK1, UBXN2B, USP24, USP24, USP24, ZNF721, ZNF721, ZWILCH, ZWILCH, ZWILCH, ZWILCH, ZWILCH, ZWILCH, ZWILCH, ZWILCH, ZWILCH, ZWILCH, ZWILCH, ZWILCH	11
hsa-miR-613	BICD1, EML4, EML4, EML4, EML4, LASP1, LASP1, LASP1, LASP1, LASP1, LASP1, LASP1, LASP1, LASP1, LASP1, LASP1, LASP1, LASP1, LASP1, LASP1, LASP1, LASP1, LASP1, MPP7, MPP7, NCK2, NCK2, NCK2, NRP1, NRP1, NRP1, NRP1, NRP1, SIKE1, SIKE1, SIKE1, SIKE1, SIKE1, SIKE1, SMG7, SMG7, SMG7, SMG7, TPM4, TPM4, TPM4, TPM4, TPM4, TPM4, TPM4, TPM4, TPM4, TPM4, TPM4, TPM4, TPM4, TPM4, TPM4, TPM4, TPM4, UNC50, UNC50, UNC50, UNC50, UNC50, WDR6, WDR6, WDR6, WDR6	11
hsa-let-7b-5p	BLOC1S6, BLOC1S6, BLOC1S6, CDC25A, CDC25A, CDC25A, CDC25A, CDC25A, CDC25A, CTNS, CTNS, CTNS, CTNS, CTNS, HIC1, HIC1, HIC1, HIC1, ITGB8, ITGB8, ITGB8, PRKAA2, PRKAA2, PRKAA2, PRKAA2, RANBP2, RPS6KA3, RPS6KA3, RPS6KA3, RPS6KA3, RPS6KA3, TAOK1, WNK3, WNK3	10
hsa-let-7g-5p	BLOC1S6, BLOC1S6, BLOC1S6, CDC25A, CDC25A, CDC25A, CDC25A, CDC25A, CDC25A, CTNS, CTNS, CTNS, CTNS, CTNS, HIC1, HIC1, HIC1, HIC1, HIC1, HIC1, HIC1, ITGB8, ITGB8, ITGB8, MEIS2, MEIS2, PRKAA2, PRKAA2, PRKAA2, PRKAA2, PRKAA2, PRKAA2, PRKAA2, PRKAA2, RANBP2, RPS6KA3, RPS6KA3, RPS6KA3, TAOK1	10
hsa-let-7i-5p	CDC25A, CDC25A, CDC25A, CDC25A, CDC25A, CDC25A, CTNS, CTNS, CTNS, CTNS, CTNS, HIC1, HIC1, HIC1, HIC1, ITGB8, ITGB8, ITGB8, MEIS2, MEIS2, PRKAA2, PRKAA2, PRKAA2, PRKAA2, PRKAA2, PRKAA2, PRKAA2, PRKAA2, PRKAA2, PRKAA2, PRKAA2, PRKAA2, RANBP2, RNF8, RNF8, RNF8, RNF8, RNF8, RNF8, RNF8, RNF8, RNF8, RNF8, RNF8, RNF8, RNF8, RNF8, RNF8, RPS6KA3, RPS6KA3, RPS6KA3, TAOK1	10
hsa-miR-103a-3p	CDC25A, CDC25A, CDC25A, CDC25A, CDC25A, CDC25A, CDK5R1, CDK5R1, CELSR2, NSD2, RBM24, RBM24, RBM24, RBM24, RBM24, RBM24, RBM24, RBM24, RNF8, RNF8, RNF8, RNF8, SCN8A, SCN8A, SCN8A, SCN8A, SCN8A, SCN8A, SCN8A, SCN8A, SCN8A, SCN8A, SCN8A, SCN8A, SCN8A, SCN8A, SCN8A, SH3GL2, SKI, TSEN2, TSEN2	10
hsa-miR-105-5p	ARHGAP29, ARHGAP29, ARHGAP29, ARHGAP29, ATF1, ATF1, BNIP3L, CMPK1, CMPK1, CMPK1, CMPK1, CMPK1, CMPK1, CMPK1, CMPK1, CMPK1, CMPK1, CREB5, CREB5, CREB5, LDLRAD3, LDLRAD3, LDLRAD3, LDLRAD3, LDLRAD3, LDLRAD3, LDLRAD3, LDLRAD3, LDLRAD3, LDLRAD3, LDLRAD3, LDLRAD3, LDLRAD3, LDLRAD3, LDLRAD3, NEK7, NEK7, NRP1, NRP1, NSD2, NSD2, NSD2, NSD2, NSD2, NSD2, NSD2, NSD2, NSD2, NSD2, NSD2, NSD2, PRKAA2, PRKAA2, PRKAA2, PRKAA2	10
hsa-miR-107	CDC25A, CDC25A, CDC25A, CDC25A, CDC25A, CDC25A, CDK5R1, CDK5R1, CELSR2, NSD2, RBM24, RBM24, RBM24, RBM24, RBM24, RBM24, RBM24, RBM24, RNF8, RNF8, RNF8, RNF8, SCN8A, SCN8A, SCN8A, SCN8A, SCN8A, SCN8A, SCN8A, SCN8A, SCN8A, SCN8A, SCN8A, SCN8A, SCN8A, SCN8A, SCN8A, SH3GL2, SKI, TSEN2, TSEN2	10

hsa-miR-130a-3p	CMPK1, CREB5, CREB5, CREB5, CREB5, CREB5, CREB5, CREB5, CREB5, CREB5, CREB5, CREB5, CREB5, CXCL6, CXCL6, DSG1, DSG1, DSG1, ITGB8, ITGB8, MPPED2, MPPED2, MPPED2, RPS6KA3, SLC25A44, SLC25A44, SLC25A44, SLC25A44, SLC25A44, SLC25A44, TNRC6A, TNRC6A, TNRC6A, TNRC6A, TNRC6A, TNRC6A, WNK3, WNK3	10
hsa-miR-138-5p	ARL6IP5, ARL6IP5, ATF1, BNIP3L, BNIP3L, BNIP3L, BNIP3L, BNIP3L, BNIP3L, BNIP3L, BNIP3L, PER1, PER1, RBBP4, RBBP4, RNF24, RNF24, RNF24, RNF24, RNF24, SLC38A1, SMG7, SMG7, SMG7, SMG7, TAOK1, TAOK1, TAOK1, TAOK1, TAOK1, TAOK1, TAOK1, TAOK1, ZMYND11	10
hsa-miR-142-3p	CCDC6, CCDC6, CCDC6, CCDC6, CREB5, CREB5, CREB5, CREB5, EML4, EML4, EML4, EML4, EML4, EML4, FAM177A1, FAM177A1, HECTD1, HECTD1, HECTD1, HECTD1, ITGB8, ITGB8, ITGB8, ITGB8, ITGB8, ITGB8, ITGB8, ITGB8, NCK2, NCK2, NSFL1C, NSFL1C, NSFL1C, SOX5, TAOK1, TAOK1, TAOK1, TAOK1	10
hsa-miR-148a-3p	CCDC6, CDK5R1, CDK5R1, CDK5R1, CDK5R1, FBXO33, FBXO33, ITGB8, ITGB8, ITGB8, ITGB8, MPPED2, MPPED2, NSD2, NSD2, NSD2, NSD2, NSD2, NSD2, NSD2, NSD2, NSD2, NSD2, NSD2, RBM24, RBM24, SLC25A44, SLC25A44, SLC25A44, SLC25A44, SLC25A44, SLC25A44, SLC25A44, SLC25A44, SLC25A44, SLC25A44, SLC25A44, SLC25A44, SMS, TNRC6A, TNRC6A, TNRC6A, TNRC6A, TNRC6A, TNRC6A	10
hsa-miR-148b-3p	CCDC6, CDK5R1, CDK5R1, CDK5R1, CDK5R1, FBXO33, ITGB8, ITGB8, ITGB8, ITGB8, MPPED2, NSD2, NSD2, NSD2, NSD2, NSD2, NSD2, RBM24, RBM24, RBM24, RBM24, SLC25A44, SLC25A44, SLC25A44, SLC25A44, SLC25A44, SLC25A44, SLC25A44, SLC25A44, SLC25A44, SLC25A44, SLC25A44, SLC25A44, SMS, TNRC6A, TNRC6A, TNRC6A, TNRC6A, TNRC6A, TNRC6A	10
hsa-miR-199b-5p	ARF6, ARF6, EXTL3, FBXO33, KDM1B, KDM1B, RANBP2, RBBP4, RBBP4, RBBP4, RBBP4, SLC25A23, SLC25A23, SLC25A23, SLC25A23, SLC25A23, SLC25A23, SLC25A23, STAG1, STAG1, TAOK1, TAOK1, TAOK1, TAOK1, TAOK1, TTC19	10
hsa-miR-205-5p	CSDE1, FAM168A, FAM168A, FAM168A, FAM168A, FAM177A1, FAM177A1, NCEH1, NCEH1, NCEH1, NSD2, NSD2, NSD2, NSD2, NSFL1C, NSFL1C, NSFL1C, PCNX1, SMG7, SMG7, SMG7, SMG7, SMG7, SMG7, SMG7, SMG7, TAOK1, TTC19	10
hsa-miR-29b-3p	CMPK1, CMPK1, CREB5, CREB5, CREB5, CREB5, CREB5, CREB5, CREB5, CREB5, CREB5, CREB5, CREB5, CREB5, CREB5, CREB5, EML4, EML4, LASP1, LASP1, LASP1, LASP1, LASP1, LASP1, NSD2, SH3GLB1, SH3GLB1, SH3GLB1, SHROOM2, SHROOM2, SHROOM2, SHROOM2, SIKE1, SIKE1, SKI, SMS	10
hsa-miR-29c-3p	CMPK1, CMPK1, CREB5, CREB5, CREB5, CREB5, CREB5, CREB5, CREB5, CREB5, CREB5, CREB5, CREB5, CREB5, CREB5, CREB5, EML4, EML4, EML4, EML4, EML4, EML4, LASP1, LASP1, LASP1, LASP1, LASP1, LASP1, NSD2, NSD2, NSD2, NSD2, NSD2, NSD2, NSD2, NSD2, NSD2, NSD2, NSD2, SH3GLB1, SH3GLB1, SH3GLB1, SHROOM2, SHROOM2, SHROOM2, SHROOM2, SIKE1, SIKE1, SKI, SMS	10
hsa-miR-330-5p	BSDC1, BSDC1, BSDC1, BSDC1, BSDC1, BSDC1, BSDC1, BSDC1, BSDC1, BSDC1, BSDC1, BSDC1, CTNS, CTNS, CTNS, CTNS, CTNS, CTNS, CTNS, CTNS, CTNS, CTNS, CTNS, CTNS, CTNS, DAG1, FAM168A, FAM168A, FAM168A, FAM168A, FAM168A, FAM168A, FAM168A, FAM168A, FAM168A, FAM168A, FAM168A, FAM168A, FAM168A, FSCN1, ITGB8, ITGB8, LASP1, LASP1, LASP1, LASP1, LASP1, LASP1, LASP1, LASP1, LASP1, LASP1, LASP1, LASP1, LASP1, PCNX1, PCNX1, PCNX1, PCNX1, PCNX1, PCNX1, PCNX1, PCNX1, SIRPA, SIRPA, SIRPA, SIRPA, SIRPA, SIRPA, SIRPA, SIRPA, SIRPA, SIRPA, SIRPA, SIRPA, TVP23B, TVP23B	10

hsa-miR-34a-5p	CHST12, CHST12, CHST12, CHST12, CHST12, CHST12, CHST12, CHST12, CHST12, CHST12, CREB5, CREB5, CREB5, CREB5, DAG1, DAG1, DAG1, DAG1, DAG1, DAG1, DNM1L, DNM1L, DNM1L, DNM1L, DNM1L, DNM1L, DNM1L, DNM1L, DNM1L, DNM1L, DNM1L, DNM1L, MPPED2, MPPED2, MPPED2, SLC25A44, SLC25A44, SLC25A44, SLC25A44, SVIP, TTC19, USP24, USP24, USP24, ZMYND11, ZMYND11, ZMYND11, ZMYND11, ZMYND11, ZMYND11, ZMYND11, ZMYND11, ZMYND11, ZMYND11, ZMYND11, ZMYND11, ZMYND11, ZMYND11, ZMYND11, ZMYND11, ZMYND11, ZMYND11	10
hsa-miR-4429	BNIP3L, BNIP3L, CMPK1, COPS2, COPS2, DAG1, DAG1, DAG1, DAG1, DAG1, DAG1, DAG1, GSPT1, GSPT1, GSPT1, RANBP2, RBM24, RBM24, RBM24, RBM24, SMG7, SMG7, SMG7, SMG7, STAMBP, STAMBP, STAMBP, STAMBP, STAMBP, STAMBP, STAMBP, STAMBP, STAMBP, TAOK1	10
hsa-miR-449b-5p	CHST12, CHST12, CHST12, CHST12, CREB5, CREB5, CREB5, CREB5, FMNL3, FMNL3, FMNL3, MPPED2, MPPED2, MYO1C, MYO1C, MYO1C, MYO1C, SVIP, SVIP, TAOK1, TTC19, USP24, USP24, USP24, ZMYND11	10
hsa-miR-4739	CREB3L1, CREB3L1, CREB3L1, CREB3L1, CREB3L1, CREB3L1, GINS1, GINS1, GINS1, LASP1, LDLRAD3, LDLRAD3, LDLRAD3, LDLRAD3, LDLRAD3, LDLRAD3, LDLRAD3, LDLRAD3, MNT, MNT, MNT, MNT, MNT, MNT, NDUFS7, NPR3, NPR3, NPR3, NPR3, NPR3, NPR3, NPR3, NPR3, NPR3, NPR3, SKI, SKI, SKI, SKI, SKI, SKI, SKI, SKI, SKI, SKI, SKI, SKI, SKI, SKI, TJAP1, UBQLN2, UBQLN2, UBQLN2, UBQLN2, UBQLN2, UBQLN2, UBQLN2, UBQLN2, UBQLN2, UBQLN2, UBQLN2, UBQLN2	10
hsa-miR-513a-5p	ANO6, ANO6, ANO6, ANO6, ANO6, ANO6, ANO6, ANO6, BNIP3L, BNIP3L, BNIP3L, BNIP3L, FBXO33, FBXO33, NSD2, NSD2, NSD2, NSD2, NSD2, NSD2, NSD2, NSD2, NSD2, NSD2, PRKAA2, PRKAA2, PRKAA2, PRKAA2, RNF144A, RNF144A, SRRM2, TAOK1, TMX4, TMX4, TMX4, TMX4, WNK3, WNK3	10
hsa-miR-542-3p	ANLN, ANLN, CMPK1, CMPK1, COPS2, COPS2, CREB5, CREB5, CREB5, CREB5, CREB5, CREB5, CREB5, ITGB8, ITGB8, ITGB8, NOMO1, PCNX1, PCNX1, PCNX1, PCNX1, PLPPR4, PLPPR4, RANBP2, RANBP2, RNF216, RNF216	10
hsa-miR-548o-3p	CDK5R1, CDK5R1, CDK5R1, CDK5R1, CMPK1, CMPK1, CMPK1, CMPK1, CMPK1, CMPK1, EEPD1, FAM168A, FAM168A, FAM168A, FAM168A, FAM168A, FAM168A, FAM168A, FAM168A, GINS1, GINS1, GINS1, GINS1, GINS1, GINS1, GINS1, GINS1, GINS1, GINS1, GINS1, GINS1, GINS1, GINS1, GINS1, GINS1, GINS1, GINS1, LDLRAD3, LDLRAD3, LDLRAD3, LDLRAD3, LDLRAD3, LDLRAD3, NRP1, NRP1, NRP1, NRP1, PRKAA2, PRKAA2, PRKAA2, PRKAA2, PRKAA2, RPL15, UBXN2B, UBXN2B, UBXN2B, UBXN2B	10
hsa-miR-5688	ANLN, ANLN, BLOC1S6, BLOC1S6, BLOC1S6, BLOC1S6, BLOC1S6, BLOC1S6, CCDC6, DAG1, DAG1, DAG1, DAG1, DAG1, DAG1, GINS1, GINS1, GINS1, LDLRAD3, LDLRAD3, SKI, SKI, TAOK1, TAOK1, TAOK1, TAOK1, TAOK1, TAOK1, TAOK1, TAOK1, THBD, TNRC6A, TNRC6A, TNRC6A, TNRC6A, TNRC6A	10
hsa-miR-582-5p	FMNL3, INSM1, INSM1, MNT, MNT, MNT, MNT, NEK7, RBM24, RBM24, RBM24, RBM24, RBM24, RBM24, ST7L, ST7L, ST7L, ST7L, TAOK1, TAOK1, TAOK1, TAOK1, TAOK1, TAOK1, TAOK1, TAOK1, TAOK1, TAOK1, TNRC6A, TNRC6A, TNRC6A, TNRC6A, TNRC6A, TNRC6A, TNRC6A, TNRC6A, TNRC6A, TNRC6A, TVP23B, TVP23B, USP24, USP24, USP24, USP24	10
hsa-miR-625-5p	BLOC1S6, BLOC1S6, BLOC1S6, BNIP3L, BNIP3L, BSDC1, BSDC1, BSDC1, DAG1, DAG1, DAG1, DAG1, DAG1, DAG1, DAG1, DAG1, DAG1, DAG1, DAG1, DAG1, GSPT1, GSPT1, LASP1, RNF24, RNF24, RNF24, RNF24, RNF24, RNF24, RNF24, RNF24, SIKE1, SIKE1, TAOK1, TAOK1, TAOK1, TNRC6A, TNRC6A	10

hsa-let-7c-5p	CDC25A, CDC25A, CDC25A, CDC25A, CDC25A, CDC25A, CTNS, CTNS, CTNS, CTNS, CTNS, HIC1, HIC1, HIC1, HIC1, ITGB8, ITGB8, ITGB8, ITGB8, PRKAA2, PRKAA2, PRKAA2, PRKAA2, RANBP2, RPS6KA3, RPS6KA3, TAOK1, WNK3, WNK3	9
hsa-miR-1271-5p	ANLN, ANLN, ANLN, ANLN, CELSR2, EPM2AIP1, GOLGA8B, GOLGA8B, GOLGA8B, GOLGA8B, MYO1C, MYO1C, MYO1C, MYO1C, MYO1C, MYO1C, NEK7, OGDH, OGDH, PLPPR4, PLPPR4, SH3GLB1, SH3GLB1, SH3GLB1, SH3GLB1, SH3GLB1, SH3GLB1, SH3GLB1, SH3GLB1, SH3GLB1, SH3GLB1, SH3GLB1	9
hsa-miR-1297	EPM2AIP1, EPM2AIP1, EPM2AIP1, ITGB8, ITGB8, NSD2, NSD2, NSD2, NSD2, PCNX1, TAOK1, TNRC6A, TNRC6A, TNRC6A, TRIM33, USP9X, USP9X, ZNF286A, ZNF286A, ZNF286A, ZNF286A, ZNF286A	9
hsa-miR-152-3p	CDK5R1, CDK5R1, CDK5R1, CDK5R1, FBXO33, ITGB8, ITGB8, ITGB8, ITGB8, MPPED2, NSD2, NSD2, NSD2, NSD2, NSD2, NSD2, RBM24, RBM24, SLC25A44, SLC25A44, SLC25A44, SLC25A44, SLC25A44, SLC25A44, SLC25A44, SLC25A44, SLC25A44, SLC25A44, SLC25A44, SLC25A44, SMS, TNRC6A, TNRC6A, TNRC6A, TNRC6A, TNRC6A, TNRC6A	9
hsa-miR-181c-5p	ARF6, ARF6, BLOC1S6, BLOC1S6, BLOC1S6, BLOC1S6, BLOC1S6, BLOC1S6, BLOC1S6, BLOC1S6, BLOC1S6, BLOC1S6, BLOC1S6, BLOC1S6, BLOC1S6, BLOC1S6, BLOC1S6, BLOC1S6, BLOC1S6, BLOC1S6, CCDC6, CREB5, CREB5, CREB5, CREB5, CREB5, CREB5, CREB5, CREB5, ITGB8, ITGB8, MADD, MADD, MADD, MADD, MADD, MADD, MADD, MADD, NEK7, SMG7, SMG7, SMG7, SMG7, TAOK1	9
hsa-miR-183-5p	BNIP3L, BNIP3L, BNIP3L, BNIP3L, CDK5R1, CDK5R1, DNM1L, DNM1L, DNM1L, DNM1L, EML4, ITGB8, MACROD2, MACROD2, MACROD2, NCK2, NCK2, NCK2, NCK2, NCK2, NCK2, NRP1, NRP1, NRP1, NRP1, NRP1, NRP1, NRP1, NRP1, OXNAD1, OXNAD1	9
hsa-miR-199a-5p	ARF6, ARF6, EXTL3, KDM1B, KDM1B, RANBP2, RBBP4, RBBP4, RBBP4, SLC25A23, SLC25A23, SLC25A23, SLC25A23, SLC25A23, SLC25A23, SLC25A23, STAG1, TAOK1, TAOK1, TAOK1, TAOK1, TAOK1, TAOK1, TAOK1, TAOK1, TAOK1, TAOK1, TTC19	9
hsa-miR-200b-3p	ANLN, ANLN, ANO6, ANO6, ANO6, ANO6, ANO6, ANO6, FBXO33, FSCN1, NSD2, NSD2, PCNX1, SCP2, SCP2, SCP2, SCP2, SOX5, SOX5, SOX5, SOX5, SOX5, SOX5, TRIM33, TRIM33, TRIM33, TRIM33	9
hsa-miR-23c	CCDC6, MADD, MADD, MADD, MADD, MADD, MADD, MADD, MADD, NEK7, NEK7, NPR3, NPR3, NSD2, NSD2, NSD2, NSD2, NSD2, NSD2, NSD2, NSD2, SIRPA, SIRPA, SIRPA, SIRPA, SMS, TNRC6A, TNRC6A, TNRC6A, TNRC6A, TNRC6A, TNRC6A, TNRC6A, TNRC6A, TNRC6A, TNRC6A, TNRC6A, TNRC6A, TNRC6A, TNRC6A, TNRC6A, WNK3, WNK3	9
hsa-miR-30b-5p	ATF1, BNIP3L, BNIP3L, BNIP3L, BNIP3L, BNIP3L, BNIP3L, BNIP3L, BNIP3L, PRKAA2, PRKAA2, PRKAA2, PRKAA2, PRKAA2, PRKAA2, RARB, RARB, RARB, RARB, RARB, RARB, SCN8A, SCN8A, SCN8A, SCN8A, SCN8A, SCN8A, SCN8A, SCN8A, STRIP1, STRIP1, TAOK1, TAOK1, TAOK1, TAOK1, TAOK1, TAOK1, TAOK1, TAOK1, TAOK1, TAOK1, TAOK1, TAOK1, TAOK1, TAOK1, TAOK1, TNRC6A, TVP23B, TVP23B, TVP23B	9
hsa-miR-33b-5p	ARHGAP29, ARHGAP29, ARHGAP29, CDK5R1, CDK5R1, CDK5R1, CDK5R1, DNM1L, DNM1L, DNM1L, DNM1L, DNM1L, DNM1L, DNM1L, DNM1L, MEIS2, MEIS2, PCM1, PCM1, PCM1, PCM1, PCNX1, PCNX1, SCN8A, SCN8A, SCN8A, SCN8A, SCN8A, SCN8A, SCN8A, SCN8A, SKI, SKI, ZNF286A, ZNF286A, ZNF286A, ZNF286A, ZNF286A, ZNF286A, ZNF286A, ZNF286A, ZNF286A	9
hsa-miR-448	CREB5, CREB5, CREB5, CREB5, EHD3, EHD3, FAM168A, FAM168A, FAM168A, FAM168A, FBXO33, FBXO33, FBXO33, FBXO33, POLR1B, POLR1B, POLR1B, POLR1B, POLR1B, POLR1B, POLR1B, POLR1B, POLR1B, POLR1B, POLR1B, POLR1B, RANBP2, RANBP2, SH3GL2, UBA6, UBA6, UBQLN2	9
hsa-miR-452-5p	ANKRD46, ANKRD46, ANKRD46, COPS2, CREB5, CREB5, CREB5, CREB5, EPM2AIP1, NRP1, NRP1, NRP1, NRP1, NRP1, NRP1, NRP1, NRP1, SCP2, SCP2, SCP2, SCP2, SCP2, SCP2, SCP2, SCP2, SCP2, SCP2, SCP2, SCP2, SOX5, SOX5, TAOK1, TAOK1, TAOK1, TAOK1, UBA6, UBA6	9

hsa-miR-5195-3p	ANO6, ANO6, ANO6, FSCN1, FSCN1, FSCN1, FSCN1, FSCN1, FSCN1, FSCN1, FSCN1, FSCN1, FSCN1, FSCN1, FSCN1, FSCN1, FSCN1, FSCN1, FSCN1, ITGB8, ITGB8, ITGB8, ITGB8, ITGB8, ITGB8, LDLRAD3, PRKX, PRKX, PRKX, PRKX, SIRPA, SIRPA, SIRPA, SIRPA, SIRPA, SIRPA, SIRPA, SIRPA, TAOK1, TAOK1, TAOK1, TAOK1, TAOK1, TAOK1, TPM4, TPM4, TPM4, TPM4, TPM4, TPM4, UBA6	9
hsa-let-7f-5p	BLOC1S6, BLOC1S6, BLOC1S6, BSDC1, BSDC1, CTNS, CTNS, CTNS, CTNS, CTNS, HIC1, HIC1, HIC1, HIC1, HIC1, HIC1, HIC1, ITGB8, ITGB8, ITGB8, ITGB8, PRKAA2, PRKAA2, PRKAA2, PRKAA2, PRKAA2, PRKAA2, RANBP2, RPS6KA3, RPS6KA3	8
hsa-miR-129-5p	CREB5, CSDE1, CSDE1, CSDE1, CSDE1, CSDE1, CSDE1, CSDE1, CSDE1, CSDE1, CSDE1, CSDE1, CSDE1, DSG1, EPM2AIP1, MEIS2, MEIS2, MEIS2, MEIS2, MEIS2, MEIS2, MEIS2, MEIS2, MEIS2, MEIS2, MEIS2, MEIS2, MPP7, MPP7, MPP7, MPP7, MPP7, MPP7, MPP7, MPP7, MPP7, MPP7, MPP7, MPP7, SIKE1, SIKE1, SIKE1, SIKE1, SIKE1, SIKE1, UBA6, UBA6, UBA6, UBA6, UBA6, UBA6, UBA6, UBA6, UBA6, UBA6, UBA6, UBA6, UBA6, UBA6, UBA6, UBA6	8
hsa-miR-132-3p	CREB5, CREB5, DCAF4L1, EML4, EML4, EML4, EML4, EML4, EML4, EML4, EML4, EML4, EML4, PRKAA2, PRKAA2, PRKAA2, PRKAA2, PRKAA2, PRKAA2, PRKAA2, PRKAA2, PRKAA2, PRKAA2, PRKAA2, PRKAA2, PRKAA2, PRKAA2, PRKAA2, PRKAA2, SOX5, TCF7L1, TJAP1, TJAP1, TJAP1, TJAP1, TJAP1, TJAP1, TJAP1, TJAP1, TJAP1, TJAP1, USP9X, USP9X	8
hsa-miR-133a-3p	BNIP3L, CDK5R1, CMPK1, CREB5, LASP1, LASP1, LASP1, LASP1, LASP1, LASP1, LASP1, LASP1, LASP1, LASP1, LASP1, LASP1, RARB, RARB, RARB, TAOK1, TAOK1, TPM4, TPM4, TPM4, TPM4, TPM4, TPM4, TPM4, TPM4, TPM4, TPM4, TPM4, TPM4	8
hsa-miR-133b	BNIP3L, CDK5R1, CMPK1, CREB5, LASP1, LASP1, LASP1, LASP1, LASP1, LASP1, LASP1, LASP1, LASP1, LASP1, LASP1, LASP1, RARB, RARB, RARB, TAOK1, TAOK1, TPM4, TPM4, TPM4, TPM4, TPM4, TPM4, TPM4, TPM4, TPM4, TPM4, TPM4, TPM4	8
hsa-miR-186-5p	ANO6, ANO6, ANO6, ANO6, ANO6, ANO6, ANO6, ANO6, ANO6, ANTXR2, CDK5R1, CDK5R1, CDK5R1, CDK5R1, CDK5R1, CDK5R1, CDK5R1, CDK5R1, CDK5R1, CDK5R1, PCNX1, PCNX1, PCNX1, PCNX1, PCNX1, PCNX1, PCNX1, PCNX1, PCNX1, PRKAA2, PRKAA2, PRKAA2, PRKAA2, RPS6KA3, RPS6KA3, RPS6KA3, RPS6KA3, RPS6KA3, RPS6KA3, RPS6KA3, RPS6KA3, UBA6, UBA6, ZNF320, ZNF320, ZNF320	8
hsa-miR-199a-3p	CDK5R1, CDK5R1, CDK5R1, CELSR2, CELSR2, COPS2, COPS2, EML4, EML4, GOLGA8B, GOLGA8B, GOLGA8B, GOLGA8B, ITGB8, ITGB8, ITGB8, ITGB8, ITGB8, ITGB8, SLC38A1, TAOK1, TAOK1, TAOK1, TAOK1, TAOK1, TAOK1	8
hsa-miR-199b-3p	CDK5R1, CDK5R1, CDK5R1, CELSR2, CELSR2, COPS2, COPS2, EML4, EML4, GOLGA8B, GOLGA8B, GOLGA8B, GOLGA8B, ITGB8, ITGB8, ITGB8, ITGB8, ITGB8, ITGB8, SLC38A1, TAOK1, TAOK1, TAOK1, TAOK1, TAOK1, TAOK1	8
hsa-miR-211-5p	ARHGAP29, CCDC6, CREB5, CREB5, CREB5, CREB5, CREB5, CREB5, CREB5, CREB5, CREB5, MEIS2, MEIS2, RANBP2, RPS6KA3, RPS6KA3, SKI, SKI, SKI, UBA6, UBA6, UBA6, UBA6	8
hsa-miR-218-5p	ARAF, ARF6, CCDC6, CCDC6, CCDC6, LASP1, LASP1, LASP1, LASP1, LASP1, LASP1, LASP1, LASP1, LASP1, LASP1, LASP1, LASP1, LASP1, LASP1, LASP1, LASP1, LASP1, LASP1, LASP1, MPPED2, MPPED2, MPPED2, PRKX, PRKX, RPS6KA3, RPS6KA3, UBQLN2	8
hsa-miR-342-3p	DCAF4L1, EPM2AIP1, EPM2AIP1, GSPT1, LASP1, LASP1, LASP1, LASP1, LASP1, LASP1, LASP1, LASP1, LASP1, LASP1, LASP1, LASP1, NOMO1, PLPPR4, PLPPR4, TAOK1, TAOK1, TAOK1, TNRC6A, TNRC6A	8
hsa-miR-3681-3p	FAM177A1, FAM177A1, FBXO33, GSPT1, INSM1, LDLRAD3, LDLRAD3, MPPED2, PCNX1, PCNX1, RNF144A	8
hsa-miR-449a	CHST12, CHST12, CHST12, CHST12, CREB5, CREB5, CREB5, CREB5, DNM1L, DNM1L, DNM1L, DNM1L, MPPED2, MPPED2, SVIP, SVIP, TTC19, USP24, USP24, USP24, ZMYND11	8
hsa-miR-493-5p	ARHGAP29, FBXO33, MADD, MADD, MADD, MADD, MADD, MADD, MADD, MADD, MED1, MED1, MED1, MED1, NXPH1, NXPH1, NXPH1, NXPH1, NXPH1, NXPH1, PCNX1, PCNX1, SMC6, SMC6, SMC6, SMC6, STAG1, STAG1	8

hsa-miR-512-3p	CMPK1, CREB5, CREB5, CREB5, EEPD1, EEPD1, EEPD1, EEPD1, EEPD1, EEPD1, ITGB8, ITGB8, RPS6KA3, RPS6KA3, STRIP1, STRIP1, TAOK1, TAOK1, TAOK1, TCF7L1	8
hsa-miR-579-3p	DPY19L1, DPY19L1, DPY19L1, DPY19L1, DPY19L1, DPY19L1, LGSN, LGSN, NPR3, NPR3, NPR3, NPR3, NPR3, NRP1, NRP1, NRP1, NRP1, NRP1, NRP1, NRP1, NRP1, NRP1, NRP1, NRP1, NRP1, NRP1, NRP1, NRP1, NRP1, NRP1, NRP1, PRKAA2, STAG1, STAG1, STAG1, SVIP, SVIP, SVIP, SVIP, TAOK1	8
hsa-miR-98-5p	BSDC1, BSDC1, CTNS, CTNS, CTNS, CTNS, CTNS, HIC1, HIC1, HIC1, HIC1, HIC1, HIC1, HIC1, ITGB8, ITGB8, ITGB8, PRKAA2, PRKAA2, PRKAA2, PRKAA2, RANBP2, RPS6KA3, RPS6KA3, RPS6KA3, RPS6KA3, RPS6KA3, TAOK1	8
hsa-let-7d-5p	BLOC1S6, BLOC1S6, BLOC1S6, BSDC1, BSDC1, CTNS, CTNS, CTNS, CTNS, CTNS, HIC1, HIC1, HIC1, HIC1, HIC1, HIC1, ITGB8, ITGB8, ITGB8, ITGB8, ITGB8, RANBP2, RPS6KA3, RPS6KA3, RPS6KA3, RPS6KA3, RPS6KA3	7
hsa-let-7e-5p	BLOC1S6, BLOC1S6, BLOC1S6, CTNS, CTNS, CTNS, CTNS, CTNS, HIC1, HIC1, HIC1, HIC1, HIC1, HIC1, HIC1, ITGB8, ITGB8, ITGB8, ITGB8, ITGB8, PRKAA2, PRKAA2, RANBP2, RPS6KA3, RPS6KA3	7
hsa-miR-1224-5p	ARHGAP29, BICD1, BICD1, BICD1, FMNL3, FMNL3, FMNL3, LASP1, LASP1, LASP1, LASP1, NSD2, NSD2, NSD2, NSD2, NSD2, NSD2, NSD2, NSD2, NSD2, NSD2, TAOK1, TAOK1, TAOK1, TAOK1, THBD, THBD	7
hsa-miR-135b-5p	BMPER, BMPER, BMPER, BMPER, BMPER, BMPER, BMPER, BMPER, CREB5, CREB5, CREB5, CREB5, CREB5, CREB5, DAG1, DAG1, DAG1, DAG1, DAG1, DAG1, RAB26, SCML2, SCML2, SCML2, SCML2, SCML2, SCML2, SCML2, SCML2, SCML2, SCML2, SCML2, SCML2, STAMBP, STAMBP, STAMBP, STAMBP, STAMBP, STAMBP, STAMBP, STAMBP, STAMBP, STAMBP, STAMBP, STAMBP, ZNF286A	7
hsa-miR-140-3p	LRRC7, LRRC7, LRRC7, NOMO1, PRKAA2, PRKAA2, PRKAA2, PRKAA2, PRKAA2, PRKAA2, RARB, RARB, RARB, SIRPA, SIRPA, SIRPA, SIRPA, SIRPA, SIRPA, SIRPA, SIRPA, SIRPA, SIRPA, SIRPA, SIRPA, SIRPA, SIRPA, SIRPA, SIRPA, STAMBP, STAMBP, STAMBP, USP9X, USP9X	7
hsa-miR-140-5p	ANO6, ANO6, CREB3L1, DAG1, DAG1, DAG1, DAG1, DAG1, DAG1, DPY19L1, DPY19L1, DPY19L1, FMNL3, SH3GL2, TPGS2, TPGS2	7
hsa-miR-204-5p	ARHGAP29, CCDC6, CREB5, CREB5, CREB5, CREB5, CREB5, CREB5, CREB5, CREB5, RANBP2, RPS6KA3, RPS6KA3, RPS6KA3, RPS6KA3, SKI, SKI, SKI, UBA6, UBA6, UBA6, UBA6	7
hsa-miR-212-3p	CREB5, CREB5, DCAF4L1, PRKAA2, PRKAA2, PRKAA2, PRKAA2, PRKAA2, PRKAA2, PRKAA2, PRKAA2, PRKAA2, PRKAA2, PRKAA2, PRKAA2, PRKAA2, PRKAA2, PRKAA2, PRKAA2, SOX5, TCF7L1, TJAP1, TJAP1, TJAP1, TJAP1, TJAP1, TJAP1, TJAP1, TJAP1, TJAP1, TJAP1, USP9X, USP9X, USP9X, USP9X	7
hsa-miR-216a-3p	FAM177A1, FAM177A1, FBXO33, GSPT1, MPPED2, PRKX, RNF144A, RNF144A, STAG1	7
hsa-miR-216a-5p	CMPK1, CMPK1, CMPK1, CMPK1, CMPK1, CMPK1, CMPK1, CMPK1, EML4, GINS1, GINS1, GINS1, GINS1, GINS1, GINS1, GINS1, GINS1, GINS1, GINS1, GINS1, GINS1, GINS1, GINS1, GINS1, PCNX1, PCNX1, RPS6KA3, SCP2, SCP2, SCP2, SCP2, ZMYND11, ZMYND11, ZMYND11, ZMYND11, ZMYND11, ZMYND11, ZMYND11, ZMYND11, ZMYND11	7
hsa-miR-299-3p	ARL6IP5, ARL6IP5, RNF24, RNF24, RNF24, RNF24, RNF24, SIRPA, SIRPA, SIRPA, SIRPA, SIRPA, SIRPA, SIRPA, SIRPA, SOX5, SOX5, SOX5, SOX5, TPM4, TPM4, TPM4, TPM4, TPM4, TPM4, TPM4, TPM4, TPM4, TPM4, TPM4, TPM4, TPM4, USP24, ZNF561, ZNF561, ZNF561, ZNF561, ZNF561, ZNF561, ZNF561, ZNF561, ZNF561, ZNF561, ZNF561, ZNF561, ZNF561, ZNF561, ZNF561, ZNF561	7
hsa-miR-32-5p	FBXO33, NOMO1, PLPPR4, PLPPR4, PRKAA2, PRKAA2, PRKAA2, PRKAA2, PRKAA2, RPL15, RPL15, RPL15, RPL15, RPL15, RPL15, TRIM33, ZNF721, ZNF721	7

hsa-miR-369-3p	BLOC1S6, BLOC1S6, BLOC1S6, BLOC1S6, BLOC1S6, BLOC1S6, BLOC1S6, BLOC1S6, BLOC1S6, BLOC1S6, BLOC1S6, BLOC1S6, BLOC1S6, BLOC1S6, BLOC1S6, GALK2, GALK2, GALK2, NSD2, RPS6KA3, RPS6KA3, RPS6KA3, TAOK1, TAOK1, TAOK1, TRIM33, TRIM33, TRIM33, TRIM33, TRIM33, TRIM33, TRIM33, TRIM33, TRIM33, TRIM33, UBA6	7
hsa-miR-377-3p	ARHGAP29, ARHGAP29, LASP1, LASP1, LASP1, LASP1, LASP1, LASP1, LASP1, LASP1, LASP1, LASP1, LASP1, LASP1, LASP1, LASP1, LASP1, LASP1, LASP1, LASP1, MADD, MADD, MADD, MADD, MADD, MADD, MADD, MADD, MADD, MADD, MADD, MADD, MADD, MADD, MADD, MADD, NSD2, NSD2, NSD2, NSD2, NSD2, NSD2, NSD2, NSD2, NSD2, NSD2, NSD2, PLPPR4, PLPPR4, RBBP4, RBBP4, RBBP4, TAOK1, TAOK1, TAOK1	7
hsa-miR-409-3p	MPP7, MPP7, MPP7, MPP7, SKI, SLC38A1, STAG1, STAG1, STAG1, STAG1, STAG1, STAG1, STAG1, STAG1, STAG1, STAG1, STAG1, STAG1, STAG1, TNRC6A, TNRC6A, TNRC6A, TNRC6A, TNRC6A, TRIM33, UBQLN2, UBQLN2, UBQLN2, UBQLN2	7
hsa-miR-421	BLOC1S6, BLOC1S6, BLOC1S6, BLOC1S6, BLOC1S6, BLOC1S6, BLOC1S6, BLOC1S6, BLOC1S6, BNIP3L, BNIP3L, BNIP3L, DCAF4L1, GSPT1, GSPT1, NXPH1, NXPH1, PRKAA2, PRKAA2, PRKAA2, PRKAA2, RELL1	7
hsa-miR-488-3p	DCAF4L1, DCAF4L1, FSCN1, FSCN1, NEXN, NEXN, NEXN, NPR3, NPR3, NPR3, NPR3, NPR3, SCP2, SCP2, SCP2, SCP2, SCP2, TRIM33, WNK3, WNK3	7
hsa-miR-494-3p	EML4, FBXO33, FBXO33, NPR3, NPR3, NRP1, NRP1, TMX4, TRIM33, TRIM33, TRIM33, TRIM33, TRIM33, TRIM33, TRIM33, TRIM33, UBQLN2	7
hsa-miR-519a-3p	ARHGAP29, ARHGAP29, ARHGAP29, CMPK1, CMPK1, CREB5, CXCL6, FANCM, FANCM, FANCM, FANCM, FANCM, RPS6KA3, WNK3, WNK3, WNK3, WNK3	7
hsa-miR-654-5p	CELSR2, CELSR2, CELSR2, CREB3L1, CREB3L1, DAG1, DAG1, DAG1, DAG1, DAG1, DAG1, DAG1, DAG1, DAG1, DAG1, DAG1, DAG1, DAG1, DAG1, EXTL3, MNT, MNT, MNT, MNT, MNT, MNT, SKI, SKI, SKI, SKI, SKI, SKI, TNRC6A, TNRC6A, TNRC6A, TNRC6A, TNRC6A, TNRC6A, TNRC6A, TNRC6A, TNRC6A	7
hsa-miR-665	CDC25A, CDC25A, CDC25A, CDC25A, GINS1, GINS1, IP6K1, IP6K1, IP6K1, KLHL31, LASP1, RPS6KA3, RPS6KA3, RPS6KA3, RPS6KA3, RPS6KA3, RPS6KA3, RPS6KA3, RPS6KA3, RPS6KA3, RPS6KA3, RPS6KA3, RPS6KA3, SIRPA, SIRPA, SIRPA, SIRPA, SIRPA, SIRPA, SIRPA, SIRPA, SIRPA, SIRPA, SIRPA, SIRPA, SIRPA, SIRPA, SIRPA, SIRPA	7
hsa-miR-7-5p	ANO6, ANO6, ANO6, ANO6, ANO6, ANO6, ANO6, ANO6, ANO6, ANO6, ANO6, ANO6, ANO6, ANO6, ANO6, EPM2AIP1, EPM2AIP1, EPM2AIP1, EPM2AIP1, EPM2AIP1, EPM2AIP1, NCEH1, NCEH1, NCEH1, NCEH1, NCEH1, NCEH1, NCEH1, NCEH1, NCEH1, NCEH1, NCEH1, NCEH1, RNF144A, TNRC6A, TNRC6A, TNRC6A, TNRC6A, TNRC6A, TNRC6A, TNRC6A, TNRC6A, TNRC6A, UBXN2B, UBXN2B, UBXN2B, UBXN2B, UBXN2B, UBXN2B, UBXN2B, UBXN2B, UBXN2B, UBXN2B, UBXN2B, UBXN2B, UBXN2B, UBXN2B, UBXN2B, ZSCAN29, ZSCAN29, ZSCAN29, ZSCAN29	7
hsa-miR-944	ANO6, ANO6, ANO6, ANO6, ANO6, ANO6, BICD1, BICD1, BICD1, BICD1, CREB5, CREB5, CREB5, CREB5, CREB5, CREB5, CREB5, CREB5, CREB5, CREB5, CREB5, CREB5, ITGB8, MPP7, MPP7, MPP7, MPP7, PCNX1, PCNX1, PCNX1, PCNX1, SHROOM2, SHROOM2, SHROOM2, SHROOM2	7
hsa-let-7a-5p	BLOC1S6, BLOC1S6, BLOC1S6, CTNS, CTNS, CTNS, CTNS, CTNS, HIC1, HIC1, HIC1, HIC1, ITGB8, ITGB8, ITGB8, ITGB8, ITGB8, PRKAA2, PRKAA2, PRKAA2, PRKAA2, RANBP2	6
hsa-miR-125b-5p	ANO6, ANO6, EBF4, IP6K1, IP6K1, IP6K1, KCNS3, KCNS3, SVIP, ZSCAN29, ZSCAN29, ZSCAN29, ZSCAN29	6
hsa-miR-1276	CREB5, CREB5, CREB5, CREB5, CREB5, CREB5, CREB5, CREB5, CREB5, CREB5, CREB5, CREB5, CREB5, CREB5, CREB5, CREB5, DSG1, EXTL3, EXTL3, EXTL3, EXTL3, EXTL3, EXTL3, EXTL3, EXTL3, SMG7, SMG7, SMG7, SMG7, SMG7, SMG7, SMG7, SMG7, SMG7, SMG7, SMG7, SMG7, SMG7, SMG7, SMG7, SMG7, TNRC6A, TNRC6A, WNK3, WNK3, WNK3, WNK3, WNK3, WNK3, WNK3, WNK3	6

hsa-miR-130a-5p	ANTXR2, BNIP3L, BNIP3L, CCDC6, CCDC6, NSD2, NSD2, NSD2, NSD2, NSD2, NSD2, NSD2, TNRC6A, TNRC6A, TNRC6A, TNRC6A, TNRC6A, TNRC6A, TNRC6A, TNRC6A, TNRC6A, TNRC6A, TNRC6A, TNRC6A, TNRC6A, TNRC6A, TNRC6A, TNRC6A, TNRC6A, TNRC6A, WNK3, WNK3, WNK3, WNK3, WNK3, WNK3, WNK3, WNK3, WNK3, WNK3, WNK3, WNK3, WNK3, WNK3	6
hsa-miR-135a-5p	BMPER, BMPER, BMPER, BMPER, BMPER, BMPER, BMPER, BMPER, BMPER, BMPER, BMPER, CREB5, CREB5, DAG1, DAG1, DAG1, DAG1, DAG1, DAG1, RAB26, STAMBP, STAMBP, STAMBP, STAMBP, STAMBP, STAMBP, STAMBP, STAMBP, STAMBP, ZNF286A, ZNF286A, ZNF286A, ZNF286A, ZNF286A, ZNF286A	6
hsa-miR-142-5p	BICD1, BICD1, FBXO33, NRP1, NRP1, NRP1, NRP1, NRP1, NRP1, NRP1, NRP1, PRKAA2, PRKAA2, STAG1, STAG1, STAG1, TVP23B, TVP23B, TVP23B, TVP23B, TVP23B, TVP23B, TVP23B, TVP23B, TVP23B	6
hsa-miR-153-3p	DPY19L1, DPY19L1, EHD3, EHD3, FAM111B, FAM111B, FAM111B, SLC38A1, TNRC6A, TNRC6A, ZMYND11, ZMYND11, ZMYND11, ZMYND11, ZMYND11, ZMYND11, ZMYND11, ZMYND11	6
hsa-miR-224-5p	CELSR2, CELSR2, CELSR2, DCAF4L1, PCNX1, PCNX1, PCNX1, TNRC6A, USP24, USP24, ZWILCH, ZWILCH, ZWILCH	6
hsa-miR-25-3p	BSDC1, BSDC1, CDK5R1, EML4, EML4, NOMO1, RPL15, RPL15, RPL15, ZNF721, ZNF721	6
hsa-miR-33a-5p	ARHGAP29, ARHGAP29, ARHGAP29, ARHGAP29, ARHGAP29, ARHGAP29, CDK5R1, CDK5R1, CDK5R1, CDK5R1, PCM1, PCM1, PCM1, PCM1, PCNX1, PCNX1, SCN8A, SCN8A, SCN8A, SCN8A, SCN8A, SCN8A, SCN8A, SCN8A, SCN8A, SCN8A, ZNF286A, ZNF286A, ZNF286A, ZNF286A, ZNF286A, ZNF286A, ZNF286A, ZNF286A, ZNF286A	6
hsa-miR-362-3p	BICD1, BICD1, CREB5, CREB5, CREB5, CREB5, CREB5, CREB5, CREB5, CREB5, CREB5, FAM177A1, FAM177A1, NSD2, PCNX1, PCNX1, PRRG3, PRRG3, PRRG3	6
hsa-miR-363-3p	CDK5R1, CDK5R1, EML4, EML4, FBXO33, NOMO1, RPL15, RPL15, RPL15, SVIP, SVIP	6
hsa-miR-3666	CREB5, CREB5, MPPED2, MPPED2, NRP1, SLC25A44, SLC25A44, TNRC6A, TNRC6A, TNRC6A, WNK3, WNK3	6
hsa-miR-371a-5p	BNIP3L, BNIP3L, EPM2AIP1, EPM2AIP1, ITGB8, ITGB8, ITGB8, NCEH1, SIKE1, SIKE1, SIKE1, SIKE1, SIKE1, SIKE1, SIKE1, SIKE1, SIKE1, SIKE1, TJAP1, TJAP1	6
hsa-miR-450b-5p	COPS2, COPS2, COPS2, GSPT1, SKI, SKI, SKI, TAOK1, TAOK1, TAOK1, TAOK1, TNRC6A, TNRC6A, TNRC6A, TNRC6A, TNRC6A, TNRC6A, UBA6, UBA6	6
hsa-miR-485-5p	CELSR2, CELSR2, CELSR2, CELSR2, CREB5, KIAA1328, KIAA1328, KIAA1328, KIAA1328, KIAA1328, KIAA1328, LRRC7, LRRC7, LRRC7, LRRC7, LRRC7, LRRC7, LRRC7, LRRC7, LRRC7, LRRC7, LRRC7, STRIP1, STRIP1, TAOK1, TAOK1, TAOK1, TAOK1, TAOK1, TAOK1, TAOK1, TAOK1	6
hsa-miR-499a-5p	DNM1L, DNM1L, DNM1L, DNM1L, DNM1L, DNM1L, DNM1L, EML4, EML4, EML4, EML4, EML4, EML4, EML4, EML4, EML4, EML4, EPM2AIP1, EPM2AIP1, EPM2AIP1, PRKAA2, PRKAA2, PRKAA2, PRKAA2, PRKAA2, PRKAA2, PRKAA2, PRKAA2, PRKAA2, PRKAA2, PRKAA2, PRKAA2, SOX5, TVP23B, TVP23B, TVP23B, TVP23B	6
hsa-miR-513b-5p	AQP4, AQP4, AQP4, AQP4, AQP4, AQP4, EML4, EML4, INSM1, NEK7, NEK7, NEK7, NSD2, NSD2, NSD2, NSD2, NSD2, PCNX1, PCNX1, PCNX1, PCNX1, PCNX1, PCNX1, PCNX1, PCNX1, PCNX1, PCNX1, PCNX1, PCNX1	6
hsa-miR-515-5p	CREB5, CREB5, EBF4, EBF4, EML4, EML4, MEIS2, MEIS2, NSD2, NSD2, NSD2, NSD2, USP24, USP24	6
hsa-miR-520a-5p	ARF6, NPR3, NPR3, NPR3, NPR3, RNF144A, STAG1, TCF7L1, UBA6	6

hsa-miR-532-5p	MPP7, MPP7, PRKX, RPS6KA3, RPS6KA3, RPS6KA3, RPS6KA3, RPS6KA3, RPS6KA3, RPS6KA3, SRRM2, SRRM2, SRRM2, SRRM2, ZMPSTE24, ZMYND11	6
hsa-miR-588	BAIAP3, BAIAP3, BAIAP3, BAIAP3, BAIAP3, BAIAP3, BAIAP3, BAIAP3, BAIAP3, BAIAP3, BAIAP3, BAIAP3, BAIAP3, BAIAP3, BAIAP3, BAIAP3, BAIAP3, BICD1, BICD1, DPY19L1, DPY19L1, DPY19L1, DPY19L1, DPY19L1, DPY19L1, DPY19L1, DPY19L1, RPS6KA3, RPS6KA3, RPS6KA3, RPS6KA3, RPS6KA3, RPS6KA3, RPS6KA3, RPS6KA3, RPS6KA3, SIRPA, SIRPA, SIRPA, SIRPA, SIRPA, SIRPA, SIRPA, SIRPA, SIRPA, SIRPA, SIRPA, SIRPA, TNRC6A, TNRC6A, TNRC6A, TNRC6A, TNRC6A, TNRC6A	6
hsa-miR-641	CCDC6, CCDC6, CCDC6, NPR3, NPR3, NPR3, NPR3, NPR3, NPR3, NPR3, PRKAA2, PRKAA2, PRKAA2, PRKAA2, PRKAA2, PRKAA2, PRKAA2, PRKAA2, PRKAA2, PRKAA2, PRKAA2, PRKAA2, PRKAA2, PRKAA2, PRKAA2, PRKAA2, PRKX, PRKX, PRKX, SHROOM2, SHROOM2, SHROOM2, SHROOM2, SHROOM2, SHROOM2, SHROOM2, SHROOM2, SHROOM2, SHROOM2, SHROOM2, SHROOM2, SVIP	6
hsa-miR-671-5p	CCDC6, CCDC6, CCDC6, CCDC6, CREB5, EHD3, EHD3, EHD3, EHD3, EHD3, NSD2, NSD2, NSD2, NSD2, NSD2, NSD2, NSD2, NSD2, NSD2, SLC25A44, SLC25A44, SLC25A44, SLC25A44, ZNF561	6
hsa-miR-92a-3p	BSDC1, BSDC1, CDK5R1, CDK5R1, CDK5R1, CDK5R1, NOMO1, PRKAA2, PRKAA2, RPL15, RPL15, RPL15, ZNF721, ZNF721	6
hsa-miR-92b-3p	BSDC1, BSDC1, CDK5R1, CDK5R1, CDK5R1, CDK5R1, NOMO1, PRKAA2, PRKAA2, RPL15, RPL15, RPL15, ZNF721, ZNF721	6
hsa-miR-1197	BLOC1S6, BLOC1S6, BLOC1S6, BLOC1S6, BLOC1S6, BLOC1S6, CTBP1, CTBP1, CTBP1, MAD2L1BP, MAD2L1BP, MADD, TNRC6A, TNRC6A	5
hsa-miR-122-5p	BICD1, BICD1, CDA, CHMP3, CHMP3, CHMP3, TANGO2, TANGO2, TANGO2, TANGO2, TANGO2, TANGO2, TANGO2, TANGO2, TANGO2, TNRC6A, TNRC6A, TNRC6A	5
hsa-miR-1278	FMNL3, FMNL3, POLR1B, POLR1B, POLR1B, POLR1B, POLR1B, POLR1B, POLR1B, POLR1B, POLR1B, POLR1B, RNPS1, RNPS1, RNPS1, RNPS1, RNPS1, RNPS1, TAOK1, TAOK1, TAOK1, TAOK1, TAOK1, TAOK1, TAOK1, TAOK1, TAOK1, TAOK1, TAOK1, TAOK1, TAOK1, TAOK1, ZNF143	5
hsa-miR-1301-3p	COPS2, COPS2, COPS2, COPS2, EML4, EML4, EML4, EML4, EML4, EML4, MACROD2, MACROD2, MACROD2, MACROD2, MACROD2, SMG7, SMG7, SMG7, SMG7, SMG7, SMG7, SMG7, SMG7, SMG7, SMG7, SMG7, SMG7, SMG7, SMG7, ZSCAN29, ZSCAN29	5
hsa-miR-146b-5p	CCDC6, CCDC6, FANCM, FANCM, FANCM, FANCM, FANCM, FANCM, FANCM, FANCM, PRKAA2, PRKAA2, PRKAA2, PRKAA2, PRKAA2, PRKAA2, PRKAA2, PRKAA2, PRKAA2, PRKAA2, PRKAA2, PRKAA2, USP24, USP24, USP24, ZNF506, ZNF506	5
hsa-miR-147a	FOXA1, MNT, MNT, MNT, MNT, NCK2, NCK2, NCK2, NCK2, NCK2, NCK2, NCK2, NCK2, SIRPA, SIRPA, SIRPA, SIRPA, TAOK1, TAOK1	5
hsa-miR-149-5p	COX19, COX19, OXNAD1, OXNAD1, OXNAD1, OXNAD1, RNF24, RNF24, SHROOM2, SHROOM2, SHROOM2, SHROOM2, SHROOM2, SHROOM2, SHROOM2, SHROOM2, STAT2, STAT2	5
hsa-miR-150-5p	BLOC1S6, GINS1, GINS1, GINS1, GINS1, NPR3, NPR3, NPR3, NPR3, NPR3, NPR3, SCP2, SCP2, SCP2, SCP2, SCP2, SCP2, TNRC6A, TNRC6A	5
hsa-miR-155-5p	NEMF, NEMF, SKI, TAOK1, TAOK1, TAOK1, TAOK1, TAOK1, TAOK1, TAOK1, TAOK1, UBXN2B, UBXN2B, USP9X, USP9X	5
hsa-miR-193a-5p	CMPK1, NSFL1C, NSFL1C, NSFL1C, SHROOM2, SHROOM2, SHROOM2, SHROOM2, SLC25A44, SLC25A44, SLC25A44, SLC25A44, TAOK1, TAOK1, TAOK1	5

hsa-miR-216b-5p	CMPK1, CMPK1, FBXO33, PCNX1, PCNX1, TVP23B, TVP23B, TVP23B, TVP23B, TVP23B, ZMYND11	5
hsa-miR-221-3p	BNIP3L, BNIP3L, ITGB8, ITGB8, NXPH1, NXPH1, NXPH1, NXPH1, SLC25A44, SLC25A44, SVIP	5
hsa-miR-3129-5p	CDK5R1, CELSR2, CELSR2, CELSR2, CELSR2, EML4, ITGB8, ITGB8, ITGB8, ITGB8, TAOK1, TAOK1, TAOK1, TAOK1, TAOK1, TAOK1, TAOK1, TAOK1, TAOK1, TAOK1, TAOK1, TAOK1, TAOK1, TAOK1, TAOK1, TAOK1, TAOK1, TAOK1, TAOK1	5
hsa-miR-3163	ANLN, ANLN, ANLN, ANLN, ATF1, CREB5, CREB5, CREB5, CREB5, CREB5, CREB5, CREB5, CREB5, CREB5, CREB5, NEK7, NEK7, NEK7, NEK7, ZNF121, ZNF121, ZNF121, ZNF121, ZNF121, ZNF121, ZNF121, ZNF121, ZNF121, ZNF121, ZNF121, ZNF121, ZNF121, ZNF121, ZNF121, ZNF121	5
hsa-miR-329-3p	BICD1, BICD1, CREB5, CREB5, CREB5, CREB5, CREB5, CREB5, CREB5, CREB5, CREB5, FAM177A1, FAM177A1, NSD2, PRRG3, PRRG3, PRRG3, PRRG3, PRRG3, PRRG3	5
hsa-miR-3619-5p	ADORA2A, ADORA2A, ADORA2A, ADORA2A, ADORA2A, ADORA2A, ADORA2A, ADORA2A, SMG7, SMG7, SMG7, SMG7, SMG7, SMG7, SMG7, SMG7, SMG7, SMG7, SMG7, SMG7, TAOK1, TAOK1, TRIM33, TRIM33, TSEN2, TSEN2	5
hsa-miR-370-3p	BSDC1, BSDC1, BSDC1, BSDC1, BSDC1, BSDC1, BSDC1, BSDC1, BSDC1, BSDC1, BSDC1, BSDC1, BSDC1, BSDC1, BSDC1, BSDC1, POLR1B, POLR1B, POLR1B, POLR1B, POLR1B, POLR1B, POLR1B, POLR1B, POLR1B, POLR1B, TAOK1, TAOK1, TAOK1, TAOK1, TPM4, TPM4, TPM4, TPM4, TPM4, TPM4, ZMYND11, ZMYND11, ZMYND11, ZMYND11, ZMYND11, ZMYND11, ZMYND11, ZMYND11, ZMYND11, ZMYND11	5
hsa-miR-374b-5p	BLOC1S6, BLOC1S6, BLOC1S6, MPP7, MPP7, MPP7, MPP7, MPP7, MPP7, MPP7, MPP7, SVIP, SVIP, TAOK1, TAOK1, TNRC6A, TNRC6A, TNRC6A, TNRC6A, TNRC6A, TNRC6A	5
hsa-miR-376c-3p	KLHDC1, MEIS2, MEIS2, MEIS2, MEIS2, RARB, RARB, RARB, SIKE1, SIKE1, SIKE1, SIKE1, SIKE1, SIKE1, UBA6	5
hsa-miR-382-5p	CCDC6, CCDC6, SH3GLB1, SH3GLB1, SH3GLB1, SIRPA, SIRPA, SOX5, SOX5, SOX5, UBXN2B	5
hsa-miR-4319	EBF4, EPOR, IP6K1, IP6K1, IP6K1, IP6K1, IP6K1, KCNS3, KCNS3, ZSCAN29, ZSCAN29	5
hsa-miR-449c-5p	CREB5, CREB5, DNM1L, DNM1L, DNM1L, DNM1L, DNM1L, DNM1L, DNM1L, DNM1L, RBM24, RBM24, RBM24, TAOK1, TAOK1, TAOK1, TAOK1, TAOK1, TAOK1, TAOK1, TAOK1, ZMPSTE24	5
hsa-miR-4644	BICD1, BICD1, BICD1, BICD1, BICD1, CELSR2, NRP1, NRP1, NRP1, NRP1, NRP1, NRP1, NRP1, NRP1, SIRPA, SIRPA, SIRPA, SIRPA, SIRPA, SIRPA, SIRPA, SIRPA, SIRPA, SIRPA, SMG7, SMG7, SMG7, SMG7, SMG7, SMG7, SMG7, SMG7, SMG7, SMG7, SMG7, SMG7	5
hsa-miR-4756-5p	CREB3L1, CREB3L1, LASP1, LASP1, LASP1, LASP1, LASP1, LASP1, LASP1, LASP1, LASP1, LASP1, LASP1, LASP1, LASP1, LASP1, LASP1, LASP1, LASP1, LASP1, NDUFS7, SKI, SKI, SKI, SKI, SKI, SKI, SKI, SLC25A44, SLC25A44, SLC25A44, SLC25A44	5
hsa-miR-491-5p	CELSR2, OGDH, OGDH, OGDH, OGDH, OGDH, SKI, SKI, SKI, SKI, SKI, SKI, SLC25A23, SLC25A23, SLC25A23, SLC25A23, SLC25A23, SLC25A23, TNRC6A, TNRC6A	5

hsa-miR-5194	CELSR2, CELSR2, CELSR2, CELSR2, CELSR2, CELSR2, CELSR2, CELSR2, CELSR2, LASP1, LASP1, LASP1, MOCS1, MOCS1, MOCS1, SIRPA, SIRPA, SIRPA, SIRPA, SIRPA, SIRPA, SIRPA, SIRPA, SIRPA, SIRPA, SIRPA, SIRPA, SIRPA, SIRPA, SIRPA, SIRPA, SYP	5
hsa-miR-541-3p	CELSR2, CELSR2, CELSR2, CELSR2, CELSR2, CELSR2, EXTL3, EXTL3, LASP1, MNT, MNT, MNT, MNT, MNT, MNT, TNRC6A, TNRC6A, TNRC6A, TNRC6A, TNRC6A, TNRC6A, TNRC6A, TNRC6A, TNRC6A, TNRC6A, TNRC6A, TNRC6A, TNRC6A, TNRC6A, TNRC6A	5
hsa-miR-5590-3p	FAM168A, FAM168A, SCML2, SCML2, SCML2, SOX5, STAG1, TVP23B, TVP23B, TVP23B, TVP23B, TVP23B, TVP23B, TVP23B, TVP23B, TVP23B, TVP23B	5
hsa-miR-574-5p	BICD1, BICD1, BLOC1S6, BLOC1S6, BLOC1S6, CCDC6, CCDC6, CCDC6, CCDC6, NCK2, NCK2, NCK2, NCK2, NCK2, NCK2, NCK2, NCK2, NCK2, NCK2, NCK2, NCK2, NSD2	5
hsa-miR-577	NSD2, NSD2, NSD2, NSD2, NSD2, NSD2, PCNX1, PRKAA2, PRKAA2, PRKAA2, PRKAA2, PRKAA2, PRKAA2, PRKAA2, PRKAA2, PRKAA2, PRKAA2, PRKAA2, PRKAA2, PRKAA2, PRKAA2, PRKAA2, PRKAA2, SVIP, ZMPSTE24, ZMPSTE24, ZMPSTE24, ZMPSTE24, ZMPSTE24, ZMPSTE24	5
hsa-miR-580-3p	FMNL3, NCK2, NCK2, NCK2, RPS6KA3, RPS6KA3, SCP2, SCP2, SCP2, SCP2, SCP2, SOX5, SOX5	5
hsa-miR-582-3p	CSDE1, CSDE1, CSDE1, CSDE1, CSDE1, CSDE1, CSDE1, CSDE1, CSDE1, CSDE1, CSDE1, CSDE1, CSDE1, CSDE1, FBXO33, FBXO33, NEK7, NEK7, NEK7, NEK7, NEK7, NEK7, NEK7, NEK7, TNRC6A, TNRC6A, TNRC6A, TNRC6A, TNRC6A, TNRC6A, TNRC6A, TNRC6A, TNRC6A, TNRC6A, TNRC6A, TNRC6A, TPM4, TPM4, TPM4, TPM4, TPM4, TPM4, TPM4	5
hsa-miR-590-5p	CREB5, CREB5, CREB5, CREB5, DSG1, GINS1, GINS1, SCML2, SCML2, SCML2, SCML2, SKI, SKI	5
hsa-miR-628-5p	ANO6, ANO6, ANO6, BNIP3L, BNIP3L, BNIP3L, BNIP3L, GINS1, RBM24, RBM24, RBM24, UBXN2B, UBXN2B, UBXN2B, UBXN2B, UBXN2B, UBXN2B	5
hsa-miR-650	CREB3L1, CREB3L1, EBF4, EBF4, EBF4, EBF4, EBF4, EBF4, EEPD1, RARB, RARB, RARB, SZT2, SZT2, SZT2, SZT2	5
hsa-miR-761	ADORA2A, ADORA2A, ADORA2A, ADORA2A, ADORA2A, ADORA2A, ADORA2A, ADORA2A, IP6K1, IP6K1, IP6K1, IP6K1, IP6K1, IP6K1, RNF8, RNF8, RNF8, RNF8, RNF8, SKI, SKI, TSEN2, TSEN2, TSEN2	5
hsa-miR-873-5p	FMNL3, FMNL3, FMNL3, FMNL3, LASP1, LASP1, LASP1, LASP1, LASP1, LASP1, LASP1, LASP1, LASP1, LASP1, LASP1, PCNX1, SRRM2, SRRM2, ZNF561, ZNF561, ZNF561, ZNF561, ZNF561, ZNF561	5
hsa-miR-876-5p	BLOC1S6, BLOC1S6, BLOC1S6, BLOC1S6, BLOC1S6, BLOC1S6, BLOC1S6, BLOC1S6, BLOC1S6, BLOC1S6, BLOC1S6, BLOC1S6, CREB5, GLOD4, GLOD4, GLOD4, TAOK1, TAOK1, TAOK1, TAOK1, TAOK1, TAOK1, TAOK1, TAOK1, TAOK1, TAOK1, WDR6, WDR6, WDR6, WDR6	5
hsa-miR-10a-5p	DCAF4L1, DCAF4L1, DCAF4L1, DCAF4L1, DCAF4L1, DCAF4L1, FAM111B, FAM111B, FAM111B, PRKAA2, PRKAA2, PRKAA2, PRKAA2, PRKAA2, PRKAA2, PRKAA2, PRKAA2, PRKAA2, PRKAA2, PRKAA2, PRKAA2, ZMYND11	4
hsa-miR-10b-5p	DCAF4L1, DCAF4L1, DCAF4L1, DCAF4L1, DCAF4L1, DCAF4L1, FAM111B, FAM111B, FAM111B, PRKAA2, PRKAA2, PRKAA2, PRKAA2, PRKAA2, PRKAA2, ZMYND11	4
hsa-miR-125a-5p	ANO6, ANO6, IP6K1, IP6K1, IP6K1, IP6K1, KCNS3, KCNS3, ZSCAN29, ZSCAN29, ZSCAN29, ZSCAN29	4
hsa-miR-1270	BLOC1S6, BLOC1S6, BLOC1S6, BLOC1S6, BLOC1S6, BLOC1S6, CELSR2, CELSR2, COPS2, COPS2, COPS2, SMG7, SMG7, SMG7	4
hsa-miR-1277-5p	ARHGAP29, ARHGAP29, ARHGAP29, NCK2, NCK2, NCK2, PCNX1, PCNX1, PCNX1, SVIP, SVIP, SVIP, SVIP, SVIP, SVIP, SVIP	4
hsa-miR-134-5p	CMPK1, CMPK1, EML4, EML4, EML4, EML4, NSD2, NSD2, NSD2, NSD2, NSD2, NSD2, NSD2, NSD2, NSD2, NSD2, NSD2, UBA6, UBA6	4

hsa-miR-190a-5p	BNIP3L, BNIP3L, BNIP3L, DAG1, DAG1, DAG1, DAG1, DAG1, DAG1, DAG1, DAG1, DAG1, DAG1, DAG1, DAG1, TNRC6A, TNRC6A, TNRC6A, TNRC6A, TNRC6A, TNRC6A, TRIM33, TRIM33, TRIM33, TRIM33, TRIM33, TRIM33, TRIM33, TRIM33	4
hsa-miR-1914-3p	LASP1, LASP1, LASP1, SIRPA, SIRPA, SIRPA, SIRPA, SIRPA, SIRPA, SIRPA, SIRPA, SIRPA, SIRPA, SIRPA, SIRPA, SKI, SYP	4
hsa-miR-194-5p	ARL6IP5, ARL6IP5, ARL6IP5, ARL6IP5, ARL6IP5, ARL6IP5, NRP1, NRP1, NRP1, NRP1, NRP1, NRP1, NRP1, NRP1, NRP1, NRP1, TAOK1, TJAP1, TJAP1, TJAP1, TJAP1, TJAP1, TJAP1, TJAP1, TJAP1, TJAP1	4
hsa-miR-196b-5p	BLOC1S6, BLOC1S6, BLOC1S6, FAM110B, RANBP2, ZMYND11	4
hsa-miR-21-5p	CREB5, CREB5, SCML2, SCML2, SCML2, SCML2, SKI, SOX5	4
hsa-miR-215-5p	CCDC152, CREB5, CREB5, CREB5, GOLGA8B, GOLGA8B, TAOK1, TAOK1, TAOK1	4
hsa-miR-22-3p	DCAF4L1, DCAF4L1, DCAF4L1, LDLRAD3, SKI, SKI, SKI, SMG7, SMG7, SMG7, SMG7, SMG7, SMG7, SMG7, SMG7, SMG7, SMG7, SMG7, SMG7, SMG7, SMG7, SMG7	4
hsa-miR-222-3p	BNIP3L, BNIP3L, NXPH1, NXPH1, SVIP, TAOK1	4
hsa-miR-2682-5p	DNM1L, DNM1L, DNM1L, DNM1L, DNM1L, DNM1L, DNM1L, DNM1L, DNM1L, DNM1L, DNM1L, DNM1L, RBM24, RBM24, RBM24, TAOK1, TAOK1, TAOK1, TAOK1, TAOK1, TAOK1, ZMPSTE24	4
hsa-miR-323a-3p	ARL6IP5, ARL6IP5, ARL6IP5, ARL6IP5, ARL6IP5, ARL6IP5, ARL6IP5, ARL6IP5, ATF1, ATF1, ATF1, NPR3, NPR3, PCNX1	4
hsa-miR-328-3p	HIC1, HIC1, HIC1, HIC1, HIC1, HIC1, HIC1, HIC1, MNT, MNT, MNT, MYO1C, MYO1C, MYO1C, MYO1C, MYO1C, MYO1C, MYO1C, MYO1C, MYO1C, MYO1C, MYO1C, MYO1C, MYO1C, MYO1C, MYO1C, MYO1C, STRIP1, STRIP1, STRIP1, STRIP1	4
hsa-miR-335-5p	PCNX1, PRKAA2, PRKAA2, PRKAA2, PRKAA2, SLC25A44, SLC25A44, ZMPSTE24	4
hsa-miR-338-3p	CELSR2, CELSR2, CHMP3, CHMP3, CHMP3, CHMP3, FAF1, PRKAA2, PRKAA2, PRKAA2, PRKAA2, PRKAA2, PRKAA2, PRKAA2, PRKAA2	4
hsa-miR-365a-3p	CREB5, CREB5, CREB5, CREB5, CREB5, CREB5, IP6K2, IP6K2, IP6K2, IP6K2, UBXN2B, ZMPSTE24, ZMPSTE24	4
hsa-miR-367-3p	EML4, EML4, EML4, EML4, EML4, NOMO1, RPL15, RPL15, RPL15, RPL15, RPL15, ZNF721, ZNF721	4
hsa-miR-380-3p	ARHGAP29, ARHGAP29, DSG1, PRKAA2, PRKAA2, PRKAA2, PRKAA2, PRKAA2, PRKAA2, PRKAA2, PRKAA2, PRKAA2, PRKAA2, PRKAA2, PRKAA2, PRKAA2, PRKAA2, PRKAA2, PRKAA2, TVP23B, TVP23B, TVP23B, TVP23B, TVP23B, TVP23B, TVP23B, TVP23B, TVP23B	4
hsa-miR-410-3p	BLOC1S6, BLOC1S6, BLOC1S6, BLOC1S6, BLOC1S6, BLOC1S6, BLOC1S6, BLOC1S6, BLOC1S6, BLOC1S6, BLOC1S6, BLOC1S6, NEK7, PRKAA2, PRKAA2, PRKAA2, PRKAA2, PRKAA2, PRKAA2, PRKAA2, PRKAA2, PRKAA2, PRKAA2, PRKAA2, PRKAA2, SOX5, SOX5, SOX5, SOX5, SOX5	4
hsa-miR-4306	BICD1, BICD1, BICD1, FMNL3, FMNL3, FMNL3, FMNL3, FMNL3, FMNL3, FMNL3, FMNL3, FMNL3, SIRPA, SIRPA, SIRPA, SIRPA, SIRPA, SIRPA, SIRPA, SIRPA, SMG7, SMG7, SMG7, SMG7, SMG7, SMG7, SMG7, SMG7, SMG7, SMG7, SMG7, SMG7	4
hsa-miR-431-5p	MOCS1, MOCS1, MOCS1, SMG7, SMG7, SMG7, SMG7, SMG7, SMG7, SMG7, SMG7, TAOK1, ZNF143, ZNF143, ZNF143	4
hsa-miR-432-5p	CELSR2, CELSR2, CELSR2, CELSR2, EML4, HEPH, HEPH, HEPH, HEPH, HEPH, HEPH, HEPH, MNT, MNT	4
hsa-miR-433-3p	LSM5, LSM5, LSM5, LSM5, LSM5, LSM5, LSM5, NPR3, NPR3, NPR3, NPR3, NPR3, NPR3, NPR3, NPR3, RNF24, RNF24, RNF24, RNF24, RNF24, RNF24, SH3GLB1, SH3GLB1, SH3GLB1, SH3GLB1	4
hsa-miR-455-3p	CDA, CDA, NSD2, NSD2, NSD2, NSD2, NSD2, NSD2, NSD2, NSD2, RARB, RARB, RARB, TCF7L1	4
hsa-miR-4731-5p	BCL7B, BCL7B, BCL7B, FAM168A, FAM168A, FAM168A, FAM168A, MNT, MNT, TPM4, TPM4, TPM4, TPM4, TPM4, TPM4, TPM4, TPM4, TPM4, TPM4, TPM4, TPM4	4

hsa-miR-4770	NPR3, NPR3, NPR3, RBM24, RBM24, SOX5, SOX5, UBXN2B, UBXN2B, UBXN2B	4
hsa-miR-483-3p	MADD, MADD, MADD, MADD, MADD, MADD, MADD, MADD, MADD, MADD, MADD, MADD, MADD, MADD, MADD, MADD, MADD, SMS, TVP23B, TVP23B, TVP23B, USP24	4
hsa-miR-485-3p	ANO6, ANO6, ANO6, ANO6, ANO6, ANO6, ANO6, ANO6, ANO6, ANO6, ARHGAP29, ARHGAP29, CEP72, TNRC6A, TNRC6A, TNRC6A, TNRC6A, TNRC6A, TNRC6A, TNRC6A, TNRC6A, TNRC6A, TNRC6A, TNRC6A, TNRC6A	4
hsa-miR-525-5p	ARF6, RNF144A, TCF7L1, UBA6	4
hsa-miR-552-3p	FBXO33, FBXO33, FBXO33, FBXO33, STAG1, TAOK1, TAOK1, TAOK1, TAOK1, TAOK1, TAOK1, TAOK1, TAOK1, TAOK1, TAOK1, TOE1	4
hsa-miR-589-5p	DCAF4L1, PRKAA2, PRKAA2, PRKAA2, PRKAA2, PRKAA2, PRKAA2, TNRC6A, USP24	4
hsa-miR-655-3p	PRKAA2, PRKAA2, PRKAA2, PRKAA2, TAOK1, TAOK1, TAOK1, TAOK1, TAOK1, TAOK1, TRIM33, ZMYND11, ZMYND11, ZMYND11, ZMYND11, ZMYND11, ZMYND11	4
hsa-miR-670-5p	CELSR2, CELSR2, RBM24, RBM24, RBM24, RBM24, SVIP, ZSCAN29, ZSCAN29	4
hsa-miR-874-3p	EEPD1, EEPD1, EEPD1, EEPD1, EEPD1, EEPD1, EXTL3, EXTL3, EXTL3, EXTL3, FMNL3, FMNL3, FMNL3, NOP9	4
hsa-miR-888-5p	OXNAD1, OXNAD1, OXNAD1, OXNAD1, OXNAD1, OXNAD1, OXNAD1, RNF8, RNF8, RNF8, RNF8, RNF8, RNF8, RNF8, RNF8, SVIP, TRIM33, TRIM33, TRIM33	4
hsa-miR-1286	NCEH1, NCEH1, NCEH1, NCEH1, NCEH1, NCEH1, SLC25A44, SLC25A44, SLC25A44, SLC25A44, SLC25A44, SLC25A44, SLC25A44, SLC25A44, ZMYND11, ZMYND11, ZMYND11, ZMYND11, ZMYND11, ZMYND11, ZMYND11, ZMYND11, ZMYND11, ZMYND11	3
hsa-miR-139-5p	ITGB8, ITGB8, ITGB8, ITGB8, TAOK1, TAOK1, USP24, USP24	3
hsa-miR-146a-5p	CCDC6, CCDC6, FANCM, FANCM, FANCM, FANCM, FANCM, FANCM, FANCM, FANCM, PRKAA2, PRKAA2, PRKAA2, PRKAA2, PRKAA2, PRKAA2, PRKAA2, PRKAA2, PRKAA2, PRKAA2, PRKAA2, PRKAA2, PRKAA2, PRKAA2, PRKAA2, PRKAA2, PRKAA2, PRKAA2	3
hsa-miR-184	CREB3L1, CTBP1, DAG1, DAG1, DAG1, DAG1, DAG1, DAG1, DAG1, DAG1, DAG1, DAG1, DAG1, DAG1	3
hsa-miR-192-5p	CREB5, CREB5, CREB5, TAOK1, TAOK1, TAOK1, UBXN2B, UBXN2B, UBXN2B, UBXN2B	3
hsa-miR-193a-3p	CELSR2, CELSR2, SCP2, SCP2, SCP2, SCP2, TAOK1, TAOK1	3
hsa-miR-193b-3p	CELSR2, SCP2, SCP2, SCP2, SCP2, TAOK1, TAOK1	3
hsa-miR-196a-5p	BLOC1S6, BLOC1S6, BLOC1S6, BLOC1S6, BLOC1S6, BLOC1S6, BLOC1S6, BLOC1S6, BLOC1S6, RANBP2, RANBP2, ZMYND11	3
hsa-miR-223-3p	CMPK1, COPS2, RANBP2, RANBP2	3
hsa-miR-2467-3p	FAM168A, FAM168A, FAM168A, FAM168A, FAM168A, FAM168A, PCNX1, PCNX1, PCNX1, PCNX1, PCNX1, PCNX1, PCNX1, PCNX1, PCNX1, PCNX1, USP24	3
hsa-miR-28-3p	ANTXR2, CMPK1, CMPK1, CMPK1, CMPK1, CMPK1, CMPK1, ZMPSTE24	3
hsa-miR-28-5p	EPM2AIP1, FMNL3, FMNL3, FMNL3, FMNL3, FMNL3, FMNL3, LASP1, LASP1, LASP1, LASP1	3
hsa-miR-296-3p	CREB5, CREB5, CREB5, CREB5, CREB5, CREB5, CREB5, HIC1, HIC1, HIC1, HIC1, MNT	3

hsa-miR-296-5p	PRKX, SLC25A23, SLC25A23, SMG7	3
hsa-miR-3064-5p	CCDC6, PPIL4, STAT2, STAT2	3
hsa-miR-31-5p	EPM2AIP1, LASP1, NCK2, NCK2, NCK2	3
hsa-miR-3150a-3p	BSDC1, BSDC1, BSDC1, BSDC1, BSDC1, BSDC1, SLC25A23, SMG7, SMG7, SMG7	3
hsa-miR-3184-5p	CELSR2, CELSR2, CELSR2, CELSR2, CELSR2, CELSR2, CELSR2, CELSR2, LASP1, LASP1, LASP1, LASP1, LASP1, LASP1, LASP1, LASP1, LASP1, LASP1, LASP1, LASP1, SYP	3
hsa-miR-34b-5p	CREB5, CREB5, CREB5, DNM1L, DNM1L, DNM1L, DNM1L, DNM1L, DNM1L, DNM1L, DNM1L, TAOK1, TAOK1, TAOK1, TAOK1	3
hsa-miR-361-5p	NRP1, NRP1, NRP1, PLPPR4, PLPPR4, TTC19, TTC19	3
hsa-miR-362-5p	CDK5R1, CDK5R1, EPM2AIP1, EPM2AIP1, NPR3, NPR3, NPR3, NPR3, NPR3, NPR3, NPR3, NPR3, NPR3, NPR3	3
hsa-miR-374a-5p	BLOC1S6, BLOC1S6, BLOC1S6, SVIP, SVIP, SVIP, TNRC6A, TNRC6A, TNRC6A, TNRC6A, TNRC6A, TNRC6A	3
hsa-miR-376b-3p	GINS1, GINS1, GINS1, NRP1, NRP1, NRP1, NRP1, ZMYND11, ZMYND11, ZMYND11, ZMYND11, ZMYND11, ZMYND11, ZMYND11	3
hsa-miR-378a-3p	CDA, NCEH1, NCEH1, NCEH1, PRPSAP2, PRPSAP2, PRPSAP2	3
hsa-miR-378g	FBXO33, FBXO33, PER1, PER1, PER1, TJAP1	3
hsa-miR-422a	CDA, NCEH1, NCEH1, NCEH1, PRPSAP2, PRPSAP2, PRPSAP2	3
hsa-miR-425-5p	CXCL6, CXCL6, CXCL6, CXCL6, FSCN1, UBQLN2	3
hsa-miR-4295	CMPK1, CREB5, CREB5, TNRC6A, TNRC6A, TNRC6A, TNRC6A, TNRC6A, TNRC6A, TNRC6A, TNRC6A, TNRC6A, TNRC6A, TNRC6A, TNRC6A	3
hsa-miR-4436a	SLC39A3, TAOK1, TAOK1, TAOK1, TAOK1, ZMPSTE24	3
hsa-miR-493-3p	EPM2AIP1, EPM2AIP1, NCEH1, NCEH1, NCEH1, NCEH1, NCEH1, NCEH1, TNRC6A, TNRC6A, TNRC6A, TNRC6A, TNRC6A, TNRC6A, TNRC6A, TNRC6A	3
hsa-miR-501-3p	ANO6, ANO6, ANO6, ANO6, ANO6, ANO6, ANO6, ANO6, ANO6, RPL15, RPL15, RPL15, TAOK1	3
hsa-miR-502-3p	ANO6, ANO6, ANO6, ANO6, ANO6, ANO6, ANO6, ANO6, ANO6, CREB5, CREB5, RPL15, RPL15, RPL15	3
hsa-miR-503-5p	MNT, MNT, RNF8, RNF8, SKI, SKI	3
hsa-miR-5047	MACROD2, MACROD2, MACROD2, MACROD2, MACROD2, MACROD2, MACROD2, MACROD2, SMG7, SMG7, SMG7, SMG7, SMG7, SMG7, SMG7, SMG7, SMG7, SMG7, SMG7, ZMYND11, ZMYND11, ZMYND11, ZMYND11, ZMYND11, ZMYND11, ZMYND11, ZMYND11, ZMYND11	3
hsa-miR-505-3p	DNM1L, DNM1L, DNM1L, DNM1L, DNM1L, DNM1L, DNM1L, DNM1L, NCK2, NCK2, NCK2, USP9X, USP9X, USP9X, USP9X	3
hsa-miR-509-3p	KIAA1328, KIAA1328, TRIM33, TRIM33, TRIM33, TRIM33, UBXN2B, UBXN2B	3
hsa-miR-516b-5p	COPS2, COPS2, COPS2, SLC25A23, SYP, SYP	3
hsa-miR-520g-3p	PCNX1, PCNX1, PCNX1, RANBP2, RANBP2, RANBP2, SVIP	3
hsa-miR-532-3p	NPR3, NPR3, NPR3, NPR3, PCNX1, PCNX1, PCNX1, SRRM2, SRRM2, SRRM2, SRRM2, SRRM2, SRRM2	3
hsa-miR-545-3p	COPS2, COPS2, COPS2, COPS2, DPY19L1, DPY19L1, DPY19L1, DPY19L1, DPY19L1, DPY19L1, TAOK1, TAOK1, TAOK1, TAOK1, TAOK1, TAOK1, TAOK1, TAOK1	3
hsa-miR-545-5p	ADORA2B, ADORA2B, BNIP3L, BNIP3L, BNIP3L, BNIP3L, BNIP3L, BNIP3L, BNIP3L, ZMYND11, ZMYND11, ZMYND11, ZMYND11, ZMYND11, ZMYND11, ZMYND11, ZMYND11, ZMYND11, ZMYND11	3

hsa-miR-642a-5p	CREB3L1, MOCS1, MOCS1, NSD2, NSD2, NSD2, NSD2, NSD2, NSD2, NSD2, NSD2, NSD2	3
hsa-miR-653-5p	IP6K2, IP6K2, IP6K2, IP6K2, IP6K2, IP6K2, IP6K2, IP6K2, IP6K2, ZNF286A, ZNF286A, ZNF286A, ZNF286A, ZNF286A, ZNF286A, ZNF286A, ZNF286A, ZNF286A, ZNF286A, ZNF721, ZNF721	3
hsa-miR-663a	KDM1B, KDM1B, SKI, SKI, SKI, TNRC6A, TNRC6A, TNRC6A	3
hsa-miR-664b-3p	NRP1, NRP1, NRP1, NRP1, NRP1, NRP1, NRP1, NRP1, NRP1, NRP1, NRP1, NRP1, NRP1, NRP1, NRP1, NRP1, PRKAA2, PRKAA2, PRKAA2, PRKAA2, PRKAA2, PRKAA2, PRKAA2, PRKAA2, TAOK1	3
hsa-miR-708-5p	EPM2AIP1, EPM2AIP1, FMNL3, FMNL3, FMNL3, FMNL3, FMNL3, FMNL3, HEPH, HEPH, HEPH, HEPH, HEPH	3
hsa-miR-758-3p	PRKAA2, PRKAA2, PRKAA2, PRKAA2, UBA6, UBA6, UBXN2B, UBXN2B, UBXN2B, UBXN2B, UBXN2B, UBXN2B, UBXN2B, UBXN2B	3
hsa-miR-760	CCDC6, CCDC6, CCDC6, CMPK1, DAG1, DAG1, DAG1, DAG1, DAG1, DAG1, DAG1, DAG1, DAG1, DAG1, DAG1, DAG1, DAG1, DAG1	3
hsa-miR-769-5p	CREB5, SH3GL2, ZNF561, ZNF561, ZNF561	3
hsa-miR-877-5p	FAM72B, FAM72B, NSD2, STAMBP, STAMBP, STAMBP, STAMBP, STAMBP, STAMBP	3
hsa-miR-942-5p	DAG1, DAG1, DAG1, DAG1, DAG1, DAG1, DAG1, MYO1C, MYO1C, MYO1C, MYO1C, MYO1C, MYO1C, TAOK1, TAOK1, TAOK1, TAOK1, TAOK1, TAOK1, TAOK1, TAOK1, TAOK1, TAOK1, TAOK1, TAOK1, TAOK1, TAOK1	3
hsa-miR-1193	CDK5R1, LASP1, LASP1, LASP1	2
hsa-miR-1269a	CREB5, CREB5, CREB5, CREB5, CREB5, CREB5, CREB5, CREB5, CREB5, CREB5, CREB5, CREB5, USP9X, USP9X	2
hsa-miR-1287-5p	DSG1, FMNL3, FMNL3, FMNL3, FMNL3, FMNL3, FMNL3, FMNL3, FMNL3, FMNL3, FMNL3, FMNL3, FMNL3, FMNL3, FMNL3, FMNL3	2
hsa-miR-129-1-3p	BNIP3L, BNIP3L, MED1, MED1, MED1	2
hsa-miR-129-2-3p	BNIP3L, BNIP3L, MED1, MED1, MED1	2
hsa-miR-1294	CTNS, CTNS, CTNS, CTNS, CTNS, PRKAA2	2
hsa-miR-1343-3p	CELSR2, CELSR2, CELSR2, CELSR2, LASP1, LASP1, LASP1, LASP1, LASP1, LASP1, LASP1, LASP1, LASP1, LASP1, LASP1, LASP1, LASP1, LASP1, LASP1	2
hsa-miR-136-5p	EXTL3, TNRC6A, TNRC6A, TNRC6A	2
hsa-miR-154-5p	COPS2, TAOK1	2
hsa-miR-188-5p	NSD2, NSD2, NSD2, NSD2, NSD2, NSD2, NSD2, NSD2, NSD2, RPS6KA3	2
hsa-miR-18a-5p	TAOK1, TAOK1, TAOK1, TAOK1, TAOK1, TAOK1, TRPC4	2
hsa-miR-18b-5p	TAOK1, TAOK1, TAOK1, TAOK1, TAOK1, TAOK1, TAOK1, TAOK1, TRPC4	2
hsa-miR-197-3p	TAOK1, TAOK1, ZMYND11, ZMYND11, ZMYND11, ZMYND11, ZMYND11, ZMYND11, ZMYND11, ZMYND11, ZMYND11	2
hsa-miR-212-5p	EPM2AIP1, NRP1, NRP1, NRP1, NRP1, NRP1	2
hsa-miR-2278	INSM1, RNF24	2
hsa-miR-299-5p	BLOC1S6, BLOC1S6, BLOC1S6, MPHOSPH8	2
hsa-miR-3126-5p	LASP1, LASP1, LASP1, LASP1, LASP1, LASP1, LASP1, UBQLN2, UBQLN2	2
hsa-miR-3150b-3p	CELSR2, CELSR2, CELSR2, SRRM2	2

hsa-miR-3180	KDM1B, KDM1B, KDM1B, KDM1B, SKI, SKI, SKI, SKI, SKI, SKI, SKI, SKI, SKI, SKI, SKI, SKI	2
hsa-miR-3180-3p	KDM1B, KDM1B, SKI, SKI, SKI, SKI, SKI, SKI, SKI, SKI, SKI, SKI	2
hsa-miR-339-5p	CDC25A, CDC25A, STRIP1, STRIP1	2
hsa-miR-345-3p	CELSR2, CELSR2, NCK2, NCK2, NCK2, NCK2, NCK2, NCK2	2
hsa-miR-345-5p	SMS, SMS, SMS, SMS, STAMBP, STAMBP, STAMBP	2
hsa-miR-3605-5p	NOP9, NOP9, SMG7, SMG7, SMG7, SMG7	2
hsa-miR-3612	SZT2, SZT2, SZT2, SZT2, TAOK1, TAOK1	2
hsa-miR-376a-3p	GINS1, GINS1, GINS1, NRP1, NRP1, NRP1, NRP1	2
hsa-miR-383-5p	BLOC1S6, BLOC1S6, BLOC1S6, BLOC1S6, BLOC1S6, BLOC1S6, SOX5	2
hsa-miR-3924	RPS6KA3, RPS6KA3, RPS6KA3, RPS6KA3, RPS6KA3, RPS6KA3, RPS6KA3, SIKE1, SIKE1	2
hsa-miR-411-5p	EML4, EML4, EML4, EML4, TRIM33, TRIM33, TRIM33, TRIM33, TRIM33, TRIM33, TRIM33, TRIM33	2
hsa-miR-4262	CCDC6, ITGB8, ITGB8	2
hsa-miR-4458	CTNS, CTNS, CTNS, CTNS, CTNS, RANBP2	2
hsa-miR-4465	PCNX1, TAOK1, TAOK1	2
hsa-miR-455-5p	NCK2, NCK2, NCK2, TAOK1, TAOK1, TAOK1, TAOK1, TAOK1, TAOK1, TAOK1, TAOK1, TAOK1, TAOK1, TAOK1, TAOK1	2
hsa-miR-4640-5p	EBF4, EBF4, EBF4, EBF4, EBF4, NOP9, NOP9, NOP9	2
hsa-miR-4676-3p	DAG1, DAG1, DAG1, DAG1, DAG1, DAG1, DAG1, RPS6KA3, RPS6KA3, RPS6KA3, RPS6KA3	2
hsa-miR-4766-5p	FAM177A1, FAM177A1, SCP2, SCP2, SCP2, SCP2, SCP2	2
hsa-miR-4784	CELSR2, CELSR2, CELSR2, CELSR2, CELSR2, CELSR2, CELSR2, CELSR2, PER1, PER1	2
hsa-miR-487a-3p	CEP72, UBA6, UBA6, UBA6, UBA6	2
hsa-miR-489-3p	COPS2, COPS2, COPS2, TAOK1, TAOK1, TAOK1	2
hsa-miR-490-3p	POLR1B, TAOK1, TAOK1	2
hsa-miR-496	ANO6, ANO6, EML4, EML4, EML4, EML4, EML4, EML4	2
hsa-miR-5000-3p	SLC39A3, ZMPSTE24	2
hsa-miR-500b-5p	CDK5R1, CDK5R1, EPM2AIP1	2
hsa-miR-5094	ITGB8, ITGB8, ITGB8, NRP1, NRP1, NRP1, NRP1, NRP1	2
hsa-miR-510-5p	DNM1L, DNM1L, DNM1L, DNM1L, DNM1L, DNM1L, DNM1L, DNM1L, PCNX1, PCNX1	2
hsa-miR-514a-3p	SYNC, SYNC, SYNC, UBQLN2, UBQLN2	2
hsa-miR-514a-5p	BSDC1, BSDC1, BSDC1, BSDC1, BSDC1, BSDC1, BSDC1, BSDC1, BSDC1, BSDC1, ZMYND11	2
hsa-miR-520h	PCNX1, PCNX1, PCNX1, SVIP	2
hsa-miR-584-5p	RPS6KA3, RPS6KA3, RPS6KA3, STAMBP, STAMBP, STAMBP, STAMBP	2
hsa-miR-599	EPM2AIP1, EPM2AIP1, TXNDC12	2
hsa-miR-6088	NPR3, NPR3, NPR3, RBM24, RBM24	2
hsa-miR-620	CELSR2, CELSR2, COPS2	2

hsa-miR-654-3p	PCNX1, SCN8A, SCN8A, SCN8A	2
hsa-miR-656-3p	NEK7, NEK7, NPR3, NPR3	2
hsa-miR-668-3p	NSD2, NSD2, NSD2, NSD2, NSD2, NSD2, NSD2, NSD2, NSD2, PRKAA2, PRKAA2, PRKAA2, PRKAA2	2
hsa-miR-766-5p	DAG1, DAG1, DAG1, DAG1, DAG1, DAG1, DAG1, SHROOM2, SHROOM2, SHROOM2, SHROOM2, SHROOM2, SHROOM2	2
hsa-miR-802	EPM2AIP1, FOXA1	2
hsa-miR-885-5p	EML4, EML4, EML4, EML4, EML4, EML4, TVP23B, TVP23B, TVP23B, TVP23B, TVP23B, TVP23B, TVP23B, TVP23B, TVP23B	2
hsa-miR-889-3p	NEMF, NEMF, PRKAA2, PRKAA2, PRKAA2, PRKAA2	2
hsa-miR-892c-3p	COPS2, RPS6KA3, RPS6KA3, RPS6KA3, RPS6KA3, RPS6KA3, RPS6KA3, RPS6KA3, RPS6KA3, RPS6KA3, RPS6KA3	2
hsa-miR-934	ANTXR2, TRIM33, TRIM33, TRIM33, TRIM33	2
hsa-miR-100-5p	INSM1	1
hsa-miR-1179	UBA6	1
hsa-miR-1185-5p	ITGB8, ITGB8, ITGB8, ITGB8, ITGB8, ITGB8	1
hsa-miR-1245b-5p	RNF24, RNF24, RNF24, RNF24, RNF24, RNF24, RNF24	1
hsa-miR-1251-5p	TAOK1, TAOK1, TAOK1, TAOK1	1
hsa-miR-127-3p	SCN8A, SCN8A, SCN8A	1
hsa-miR-1298-5p	ZNF561, ZNF561, ZNF561, ZNF561	1
hsa-miR-1913	CELSR2	1
hsa-miR-202-5p	LDLRAD3	1
hsa-miR-214-5p	RBM24, RBM24, RBM24, RBM24, RBM24, RBM24	1
hsa-miR-219a-2-3p	COPS2	1
hsa-miR-219a-5p	RBM24, RBM24, RBM24	1
hsa-miR-2681-3p	OXNAD1, OXNAD1	1
hsa-miR-3118	EML4, EML4	1
hsa-miR-3121-3p	RBM24, RBM24, RBM24	1
hsa-miR-3139	ITGB8	1
hsa-miR-3142	RNF24, RNF24, RNF24, RNF24, RNF24, RNF24, RNF24, RNF24, RNF24, RNF24, RNF24, RNF24, RNF24	1
hsa-miR-3144-3p	TAOK1	1
hsa-miR-3145-3p	PRKAA2, PRKAA2, PRKAA2, PRKAA2, PRKAA2, PRKAA2, PRKAA2, PRKAA2, PRKAA2, PRKAA2	1
hsa-miR-3164	MEIS2, MEIS2	1
hsa-miR-3174	TAOK1, TAOK1, TAOK1	1
hsa-miR-3179	SLC25A44, SLC25A44, SLC25A44, SLC25A44, SLC25A44, SLC25A44	1
hsa-miR-3194-5p	BICD1, BICD1	1
hsa-miR-3196	KDM1B, KDM1B, KDM1B, KDM1B	1
hsa-miR-324-3p	DAG1, DAG1	1
hsa-miR-325	RNF112	1

hsa-miR-331-3p	DAG1, DAG1	1
hsa-miR-361-3p	BSDC1, BSDC1, BSDC1, BSDC1, BSDC1, BSDC1, BSDC1, BSDC1, BSDC1, BSDC1	1
hsa-miR-3613-5p	BTBD10, BTBD10, BTBD10, BTBD10	1
hsa-miR-3622b-5p	RNF144A, RNF144A	1
hsa-miR-365b-3p	CREB5, CREB5, CREB5, CREB5, CREB5, CREB5	1
hsa-miR-371a-3p	FMNL3, FMNL3	1
hsa-miR-374a-3p	PLPPR4, PLPPR4, PLPPR4, PLPPR4	1
hsa-miR-374b-3p	TAOK1, TAOK1, TAOK1, TAOK1	1
hsa-miR-374c-3p	BICD1, BICD1	1
hsa-miR-379-5p	SYNC	1
hsa-miR-3909	NPR3, NPR3, NPR3, NPR3	1
hsa-miR-3918	NOP9, NOP9, NOP9, NOP9, NOP9	1
hsa-miR-409-5p	PRKAA2, PRKAA2, PRKAA2, PRKAA2, PRKAA2, PRKAA2, PRKAA2, PRKAA2	1
hsa-miR-4500	RANBP2	1
hsa-miR-4524a-5p	SKI, SKI	1
hsa-miR-4524b-5p	SKI, SKI	1
hsa-miR-4525	GNG7	1
hsa-miR-4726-5p	NOP9, NOP9	1
hsa-miR-4782-3p	RBM24, RBM24, RBM24	1
hsa-miR-486-5p	SLC38A1	1
hsa-miR-5009-3p	ZMYND11, ZMYND11, ZMYND11, ZMYND11, ZMYND11, ZMYND11, ZMYND11, ZMYND11, ZMYND11, ZMYND11	1
hsa-miR-500a-3p	CCDC6	1
hsa-miR-508-3p	NSD2, NSD2	1
hsa-miR-513c-5p	TAOK1, TAOK1, TAOK1	1
hsa-miR-517a-3p	SMG7, SMG7, SMG7, SMG7	1
hsa-miR-517b-3p	SMG7, SMG7, SMG7, SMG7	1
hsa-miR-517c-3p	SMG7, SMG7, SMG7, SMG7	1
hsa-miR-518d-5p	EPM2AIP1	1
hsa-miR-519b-5p	EPM2AIP1	1
hsa-miR-519c-5p	EPM2AIP1	1
hsa-miR-520c-5p	EPM2AIP1	1
hsa-miR-526b-5p	RARB, RARB, RARB, RARB, RARB, RARB	1
hsa-miR-556-5p	CREB5, CREB5, CREB5, CREB5, CREB5, CREB5, CREB5, CREB5	1
hsa-miR-576-5p	NCEH1, NCEH1	1
hsa-miR-624-3p	RNF8, RNF8	1

hsa-miR-627-5p	USP9X, USP9X, USP9X, USP9X, USP9X, USP9X	1
hsa-miR-629-5p	TRIM33, TRIM33, TRIM33, TRIM33, TRIM33	1
hsa-miR-652-3p	TNRC6A, TNRC6A, TNRC6A	1
hsa-miR-770-5p	DCAF4L1, DCAF4L1	1
hsa-miR-875-5p	SIKE1, SIKE1, SIKE1, SIKE1, SIKE1, SIKE1	1
hsa-miR-9-3p	INSM1, INSM1, INSM1, INSM1	1
hsa-miR-99a-5p	INSM1	1
hsa-miR-99b-5p	INSM1	1
